# Global, regional, and national incidence of six major immune-mediated inflammatory diseases: findings from the global burden of disease study 2019

**DOI:** 10.1016/j.eclinm.2023.102193

**Published:** 2023-09-09

**Authors:** GBD 2019 IMID Collaborators, Dongze Wu, Yingzhao Jin, Yuhan Xing, Melsew Dagne Abate, Mohammadreza Abbasian, Mohsen Abbasi-Kangevari, Zeinab Abbasi-Kangevari, Foad Abd-Allah, Michael Abdelmasseh, Mohammad-Amin Abdollahifar, Deldar Morad Abdulah, Aidin Abedi, Vida Abedi, Hassan Abidi, Richard Gyan Aboagye, Hassan Abolhassani, Katrina Abuabara, Morteza Abyadeh, Isaac Yeboah Addo, Kayode Nelson Adeniji, Abiola Victor Adepoju, Miracle Ayomikun Adesina, Qorinah Estiningtyas Sakilah Adnani, Mohsen Afarideh, Shahin Aghamiri, Antonella Agodi, Anurag Agrawal, Constanza Elizabeth Aguilera Arriagada, Aqeel Ahmad, Danish Ahmad, Sajjad Ahmad, Sohail Ahmad, Ali Ahmadi, Ali Ahmed, Ayman Ahmed, Janardhana P. Aithala, Abdullateef Abiodun Ajadi, Marjan Ajami, Mostafa Akbarzadeh-Khiavi, Fares Alahdab, Mohammad T. AlBataineh, Sharifullah Alemi, Adel Ali Saeed Al-Gheethi, Liaqat Ali, Sheikh Mohammad Alif, Joseph Uy Almazan, Sami Almustanyir, Jaber S. Alqahtani, Ibrahim Alqasmi, Ihsan Ullah Khan Altaf, Nelson Alvis-Guzman, Nelson J. Alvis-Zakzuk, Yaser Mohammed Al-Worafi, Hany Aly, Reza Amani, Hubert Amu, Ganiyu Adeniyi Amusa, Catalina Liliana Andrei, Adnan Ansar, Hossein Ansariniya, Anayochukwu Edward Anyasodor, Jalal Arabloo, Reza Arefnezhad, Judie Arulappan, Mohammad Asghari-Jafarabadi, Tahira Ashraf, Jamila Abdulhamid Atata, Seyyed Shamsadin Athari, Daniel Atlaw, Maha Moh'd Wahbi Atout, Avinash Aujayeb, Asma Tahir Awan, Haleh Ayatollahi, Sina Azadnajafabad, Ahmed Y. Azzam, Alaa Badawi, Ashish D. Badiye, Sara Bagherieh, Atif Amin Baig, Berihun Bantie Bantie, Martina Barchitta, Mainak Bardhan, Suzanne Lyn Barker-Collo, Francesco Barone-Adesi, Kavita Batra, Nebiyou Simegnew Bayileyegn, Amir Hossein Behnoush, Uzma Iqbal Belgaumi, Maryam Bemanalizadeh, Isabela M. Bensenor, Kebede A. Beyene, Akshaya Srikanth Bhagavathula, Pankaj Bhardwaj, Sonu Bhaskar, Ajay Nagesh Bhat, Saeid Bitaraf, Veera R. Bitra, Archith Boloor, Kaustubh Bora, João Silva Botelho, Rachelle Buchbinder, Daniela Calina, Luis Alberto Cámera, Andre F. Carvalho, Jeffrey Shi Kai Chan, Vijay Kumar Chattu, Endeshaw Chekol Abebe, Fatemeh Chichagi, Sungchul Choi, Tzu-Chieh Chou, Dinh-Toi Chu, Kaleb Coberly, Vera Marisa Costa, Rosa A.S. Couto, Natália Cruz-Martins, Omid Dadras, Xiaochen Dai, Giovanni Damiani, Ana Maria Dascalu, Mohsen Dashti, Sisay Abebe Debela, Robert Paul Dellavalle, Andreas K. Demetriades, Alemayehu Anley Demlash, Xinlei Deng, Hardik Dineshbhai Desai, Rupak Desai, Syed Masudur Rahman Dewan, Sourav Dey, Samath Dhamminda Dharmaratne, Daniel Diaz, Mahmoud Dibas, Ricardo Jorge Dinis-Oliveira, Mengistie Diress, Thanh Chi Do, Duy Khanh Doan, Masoud Dodangeh, Milad Dodangeh, Deepa Dongarwar, John Dube, Arkadiusz Marian Dziedzic, Abdelaziz Ed-Dra, Hisham Atan Edinur, Negin Eissazade, Michael Ekholuenetale, Temitope Cyrus Ekundayo, Noha Mousaad Elemam, Muhammed Elhadi, Ahmed O. Elmehrath, Omar Abdelsadek Abdou Elmeligy, Mehdi Emamverdi, Theophilus I. Emeto, Hawi Leul Esayas, Habitu Birhan Eshetu, Farshid Etaee, Adeniyi Francis Fagbamigbe, Shahriar Faghani, Ildar Ravisovich Fakhradiyev, Ali Fatehizadeh, Mobina Fathi, Alireza Feizkhah, Ginenus Fekadu, Mohammad Fereidouni, Seyed-Mohammad Fereshtehnejad, João C. Fernandes, Pietro Ferrara, Getahun Fetensa, Irina Filip, Florian Fischer, Behzad Foroutan, Masoud Foroutan, Takeshi Fukumoto, Balasankar Ganesan, Belete Negese Belete Gemeda, Seyyed-Hadi Ghamari, MohammadReza Ghasemi, Maryam Gholamalizadeh, Tiffany K. Gill, Richard F. Gillum, Mohamad Goldust, Mahaveer Golechha, Pouya Goleij, Davide Golinelli, Houman Goudarzi, Shi-Yang Guan, Yang Guo, Bhawna Gupta, Veer Bala Gupta, Vivek Kumar Gupta, Rasool Haddadi, Najah R. Hadi, Rabih Halwani, Shafiul Haque, Ikramul Hasan, Reza Hashempour, Amr Hassan, Treska S. Hassan, Sara Hassanzadeh, Mohammed Bheser Hassen, Johannes Haubold, Khezar Hayat, Golnaz Heidari, Mohammad Heidari, Reza Heidari-Soureshjani, Claudiu Herteliu, Kamran Hessami, Kamal Hezam, Yuta Hiraike, Ramesh Holla, Mohammad-Salar Hosseini, Hong-Han Huynh, Bing-Fang Hwang, Segun Emmanuel Ibitoye, Irena M. Ilic, Milena D. Ilic, Arad Iranmehr, Farideh Iravanpour, Nahlah Elkudssiah Ismail, Masao Iwagami, Chidozie C.D. Iwu, Louis Jacob, Morteza Jafarinia, Abdollah Jafarzadeh, Kasra Jahankhani, Haitham Jahrami, Mihajlo Jakovljevic, Elham Jamshidi, Chinmay T. Jani, Manthan Dilipkumar Janodia, Sathish Kumar Jayapal, Shubha Jayaram, Jayakumar Jeganathan, Jost B. Jonas, Abel Joseph, Nitin Joseph, Charity Ehimwenma Joshua, K. Vaishali, Billingsley Kaambwa, Ali Kabir, Zubair Kabir, Vidya Kadashetti, Feroze Kaliyadan, Fatemeh Kalroozi, Vineet Kumar Kamal, Amit Kandel, Himal Kandel, Srikanta Kanungo, Jafar Karami, Ibraheem M. Karaye, Hanie Karimi, Hengameh Kasraei, Sina Kazemian, Sewnet Adem Kebede, Leila Keikavoosi-Arani, Mohammad Keykhaei, Yousef Saleh Khader, Himanshu Khajuria, Faham Khamesipour, Ejaz Ahmad Khan, Imteyaz A. Khan, Maseer Khan, Md Jobair Khan, Moien A.B. Khan, Muhammad Arslan Khan, Haitham Khatatbeh, Moawiah Mohammad Khatatbeh, Sorour Khateri, Hamid Reza Khayat Kashani, Min Seo Kim, Adnan Kisa, Sezer Kisa, Hyun Yong Koh, Pavel Kolkhir, Oleksii Korzh, Ashwin Laxmikant Kotnis, Parvaiz A. Koul, Ai Koyanagi, Kewal Krishan, Mohammed Kuddus, Vishnutheertha Vishnutheertha Kulkarni, Narinder Kumar, Satyajit Kundu, Om P. Kurmi, Carlo La Vecchia, Chandrakant Lahariya, Tri Laksono, Judit Lám, Kamaluddin Latief, Paolo Lauriola, Basira Kankia Lawal, Thao Thi Thu Le, Trang Thi Bich Le, Munjae Lee, Seung Won Lee, Wei-Chen Lee, Yo Han Lee, Jacopo Lenzi, Miriam Levi, Wei Li, Virendra S. Ligade, Stephen S. Lim, Gang Liu, Xuefeng Liu, Erand Llanaj, Chun-Han Lo, Vanessa Sintra Machado, Azzam A. Maghazachi, Mansour Adam Mahmoud, Tuan A. Mai, Azeem Majeed, Pantea Majma Sanaye, Omar Mohamed Makram, Elaheh Malakan Rad, Kashish Malhotra, Ahmad Azam Malik, Iram Malik, Tauqeer Hussain Mallhi, Deborah Carvalho Malta, Mohammad Ali Mansournia, Lorenzo Giovanni Mantovani, Miquel Martorell, Sahar Masoudi, Seyedeh Zahra Masoumi, Yasith Mathangasinghe, Elezebeth Mathews, Alexander G. Mathioudakis, Andrea Maugeri, Mahsa Mayeli, John Robert Carabeo Medina, Gebrekiros Gebremichael Meles, José João Mendes, Ritesh G. Menezes, Tomislav Mestrovic, Irmina Maria Michalek, Ana Carolina Micheletti Gomide Nogueira de Sá, Ephrem Tesfaye Mihretie, Le Huu Nhat Minh, Reza Mirfakhraie, Erkin M. Mirrakhimov, Awoke Misganaw, Ashraf Mohamadkhani, Nouh Saad Mohamed, Faezeh Mohammadi, Soheil Mohammadi, Salahuddin Mohammed, Shafiu Mohammed, Syam Mohan, Anita Mohseni, Ali H. Mokdad, Sara Momtazmanesh, Lorenzo Monasta, Mohammad Ali Moni, Md Moniruzzaman, Yousef Moradi, Negar Morovatdar, Ebrahim Mostafavi, Parsa Mousavi, George Duke Mukoro, Admir Mulita, Getaneh Baye Mulu, Efrén Murillo-Zamora, Fungai Musaigwa, Ghulam Mustafa, Sathish Muthu, Firzan Nainu, Vinay Nangia, Sreenivas Narasimha Swamy, Zuhair S. Natto, Perumalsamy Navaraj, Biswa Prakash Nayak, Athare Nazri-Panjaki, Hadush Negash, Mohammad Hadi Nematollahi, Dang H. Nguyen, Hau Thi Hien Nguyen, Hien Quang Nguyen, Phat Tuan Nguyen, Van Thanh Nguyen, Robina Khan Niazi, Taxiarchis Konstantinos Nikolouzakis, Lawrence Achilles Nnyanzi, Mamoona Noreen, Chimezie Igwegbe Nzoputam, Ogochukwu Janet Nzoputam, Bogdan Oancea, In-Hwan Oh, Hassan Okati-Aliabad, Osaretin Christabel Okonji, Patrick Godwin Okwute, Andrew T. Olagunju, Matthew Idowu Olatubi, Isaac Iyinoluwa Olufadewa, Michal Ordak, Nikita Otstavnov, Mayowa O. Owolabi, P.A. Mahesh, Jagadish Rao Padubidri, Anton Pak, Reza Pakzad, Raffaele Palladino, Adrian Pana, Ioannis Pantazopoulos, Paraskevi Papadopoulou, Shahina Pardhan, Ashwaghosha Parthasarathi, Ava Pashaei, Jay Patel, Aslam Ramjan Pathan, Shankargouda Patil, Uttam Paudel, Shrikant Pawar, Paolo Pedersini, Umberto Pensato, David M. Pereira, Jeevan Pereira, Maria Odete Pereira, Renato B. Pereira, Mario F.P. Peres, Arokiasamy Perianayagam, Simone Perna, Ionela-Roxana Petcu, Parmida Sadat Pezeshki, Hoang Tran Pham, Anil K. Philip, Michael A. Piradov, Indrashis Podder, Vivek Podder, Dimitri Poddighe, Elton Junio Sady Prates, Ibrahim Qattea, Amir Radfar, Pourya Raee, Alireza Rafiei, Alberto Raggi, Fakher Rahim, Mehran Rahimi, Mahban Rahimifard, Vafa Rahimi-Movaghar, Md Obaidur Rahman, Mohammad Hifz Ur Rahman, Mosiur Rahman, Muhammad Aziz Rahman, Amir Masoud Rahmani, Mohamed Rahmani, Shayan Rahmani, Vahid Rahmanian, Premkumar Ramasubramani, Nemanja Rancic, Indu Ramachandra Rao, Sina Rashedi, Ahmed Mustafa Rashid, Nakul Ravikumar, Salman Rawaf, Elrashdy Moustafa Mohamed Redwan, Nazila Rezaei, Negar Rezaei, Nima Rezaei, Mohsen Rezaeian, Daniela Ribeiro, Mónica Rodrigues, Jefferson Antonio Buendia Rodriguez, Leonardo Roever, Esperanza Romero-Rodríguez, Aly M.A. Saad, Basema Saddik, Saeid Sadeghian, Umar Saeed, Azam Safary, Mahdi Safdarian, Sher Zaman Safi, Amene Saghazadeh, Dominic Sagoe, Fatemeh Saheb Sharif-Askari, Narjes Saheb Sharif-Askari, Amirhossein Sahebkar, Harihar Sahoo, Mohammad Ali Sahraian, Mirza Rizwan Sajid, Sateesh Sakhamuri, Joseph W. Sakshaug, Mohamed A. Saleh, Leili Salehi, Sana Salehi, Amir Salek Farrokhi, Sara Samadzadeh, Saad Samargandy, Noosha Samieefar, Abdallah M. Samy, Nima Sanadgol, Rama Krishna Sanjeev, Monika Sawhney, Ganesh Kumar Saya, Art Schuermans, Subramanian Senthilkumaran, Sadaf G. Sepanlou, Yashendra Sethi, Mahan Shafie, Humaira Shah, Izza Shahid, Samiah Shahid, Masood Ali Shaikh, Sadaf Sharfaei, Manoj Sharma, Maryam Shayan, Hatem Samir Shehata, Aziz Sheikh, Jeevan K. Shetty, Jae Il Shin, Reza Shirkoohi, Nebiyu Aniley Shitaye, K.M. Shivakumar, Velizar Shivarov, Parnian Shobeiri, Soraya Siabani, Migbar Mekonnen Sibhat, Emmanuel Edwar Siddig, Colin R. Simpson, Ehsan Sinaei, Harpreet Singh, Inderbir Singh, Jasvinder A. Singh, Paramdeep Singh, Surjit Singh, Md Shahjahan Siraj, Abdullah Al Mamun Sohag, Ranjan Solanki, Solikhah Solikhah, Yonatan Solomon, Mohammad Sadegh Soltani-Zangbar, Jing Sun, Mindy D. Szeto, Rafael Tabarés-Seisdedos, Seyyed Mohammad Tabatabaei, Mohammad Tabish, Ensiyeh Taheri, Azin Tahvildari, Iman M. Talaat, Jacques J.L. Lukenze Tamuzi, Ker-Kan Tan, Nathan Y. Tat, Razieh Tavakoli Oliaee, Arian Tavasol, Mohamad-Hani Temsah, Pugazhenthan Thangaraju, Samar Tharwat, Nigusie Selomon Tibebu, Jansje Henny Vera Ticoalu, Tala Tillawi, Tenaw Yimer Tiruye, Amir Tiyuri, Marcos Roberto Tovani-Palone, Manjari Tripathi, Guesh Mebrahtom Tsegay, Abdul Rohim Tualeka, Sree Sudha Ty, Chukwudi S. Ubah, Saif Ullah, Sana Ullah, Muhammad Umair, Srikanth Umakanthan, Era Upadhyay, Seyed Mohammad Vahabi, Asokan Govindaraj Vaithinathan, Sahel Valadan Tahbaz, Rohollah Valizadeh, Shoban Babu Varthya, Tommi Juhani Vasankari, Narayanaswamy Venketasubramanian, Georgios-Ioannis Verras, Jorge Hugo Villafañe, Vasily Vlassov, Danh Cao Vo, Yasir Waheed, Abdul Waris, Brhane Gebrehiwot Welegebrial, Ronny Westerman, Dakshitha Praneeth Wickramasinghe, Nuwan Darshana Wickramasinghe, Barbara Willekens, Beshada Zerfu Woldegeorgis, Melat Woldemariam, Hong Xiao, Dereje Y. Yada, Galal Yahya, Lin Yang, Fereshteh Yazdanpanah, Dong Keon Yon, Naohiro Yonemoto, Yuyi You, Mazyar Zahir, Syed Saoud Zaidi, Moein Zangiabadian, Iman Zare, Mohammad A. Zeineddine, Dawit T. Zemedikun, Naod Gebrekrstos Zeru, Chen Zhang, Hanqing Zhao, Chenwen Zhong, Magdalena Zielińska, Mohammad Zoladl, Alimuddin Zumla, Cui Guo, Lai-shan Tam

**Keywords:** Immune-mediated inflammatory disease, Incidence, Global burden of disease study, Trend

## Abstract

**Background:**

The causes for immune-mediated inflammatory diseases (IMIDs) are diverse and the incidence trends of IMIDs from specific causes are rarely studied. The study aims to investigate the pattern and trend of IMIDs from 1990 to 2019.

**Methods:**

We collected detailed information on six major causes of IMIDs, including asthma, inflammatory bowel disease, multiple sclerosis, rheumatoid arthritis, psoriasis, and atopic dermatitis, between 1990 and 2019, derived from the Global Burden of Disease study in 2019. The average annual percent change (AAPC) in number of incidents and age standardized incidence rate (ASR) on IMIDs, by sex, age, region, and causes, were calculated to quantify the temporal trends.

**Findings:**

In 2019, rheumatoid arthritis, atopic dermatitis, asthma, multiple sclerosis, psoriasis, inflammatory bowel disease accounted 1.59%, 36.17%, 54.71%, 0.09%, 6.84%, 0.60% of overall new IMIDs cases, respectively. The ASR of IMIDs showed substantial regional and global variation with the highest in High SDI region, High-income North America, and United States of America. Throughout human lifespan, the age distribution of incident cases from six IMIDs was quite different. Globally, incident cases of IMIDs increased with an AAPC of 0.68 and the ASR decreased with an AAPC of −0.34 from 1990 to 2019. The incident cases increased across six IMIDs, the ASR of rheumatoid arthritis increased (0.21, 95% CI 0.18, 0.25), while the ASR of asthma (AAPC = −0.41), inflammatory bowel disease (AAPC = −0.72), multiple sclerosis (AAPC = −0.26), psoriasis (AAPC = −0.77), and atopic dermatitis (AAPC = −0.15) decreased. The ASR of overall and six individual IMID increased with SDI at regional and global level. Countries with higher ASR in 1990 experienced a more rapid decrease in ASR.

**Interpretation:**

The incidence patterns of IMIDs varied considerably across the world. Innovative prevention and integrative management strategy are urgently needed to mitigate the increasing ASR of rheumatoid arthritis and upsurging new cases of other five IMIDs, respectively.

**Funding:**

The Global Burden of Disease Study is funded by the 10.13039/100000865Bill and Melinda Gates Foundation. The project funded by Scientific Research Fund of Sichuan Academy of Medical Sciences & Sichuan Provincial People's Hospital (2022QN38).

**Research in context**

**Evidence before this study**

We conducted a systematic search of the Medline and EMBASE databases from their inception to January 9, 2023, using the keywords “immune-mediated inflammatory disease”, “incidence”, “trend”, “trend analysis”, “rheumatoid arthritis”, “atopic dermatitis”, “asthma”, “multiple sclerosis”, “inflammatory bowel disease”, and “psoriasis”. Although the burden of immune-mediated inflammatory diseases (IMIDs) is increasing globally, few studies have focused on the most up-to-date incidence trends of IMIDs on a global scale. Most studies have been limited to a single cause, country, or population, or have considered the trend by a single factor. To our knowledge, a comprehensive analysis of the pattern and trends of IMID incidence has not been reported. Therefore, this study aims to fill this gap by providing a comprehensive and up-to-date assessment of the global burden of six major IMIDs, including asthma, inflammatory bowel disease, multiple sclerosis, rheumatoid arthritis, psoriasis, and atopic dermatitis, from 1990 to 2019.

**Added value of this study**

Our study presents a comprehensive analysis of the temporal trends of IMIDs by gender, age, cause, region, and country, and their association with the socio-demographic index (SDI) across the world. Our findings reveal a wide variation in the incidence of IMIDs, and the age-standardized rate (ASR) of overall and six individual IMIDs increased with SDI across 21 Global Burden of Disease regions and 204 countries and territories. Globally, the number of incident cases of IMIDs increased, while the age-standardized rate decreased from 1990 to 2019. Throughout human lifespan, the age distribution of incident cases from six IMIDs was quite different. Among the six IMIDs studied, incident cases increased, and the ASR of rheumatoid arthritis increased, while the ASR of asthma, inflammatory bowel disease, multiple sclerosis, psoriasis, and atopic dermatitis decreased. We identified several at-risk populations for increasing trends in patients with IMIDs, including those with rheumatoid arthritis, people aged 60 years or older, and those from high-income countries. Our study provides valuable insights into the global burden of IMIDs and can inform future public health policies aimed at reducing their impact.

**Implications of all the available evidence**

The magnitude of incident cases of IMIDs has increased significantly over the past few decades. As a result, there is an urgent need for an integrative management strategy to address the increasing ASR of rheumatoid arthritis and the upsurge in new cases of the other five IMIDs studied. Furthermore, future analyses of IMID trends should also consider the potential impact of the COVID-19 pandemic on incidence rates. One Health after the COVID-19 pandemic is an opportunity to focus efforts and resources on IMIDs, which can strengthen multisectoral coordination mechanisms.

## Introduction

Immune-mediated inflammatory diseases (IMIDs) encompass a heterogeneous group of disorders affecting various organs and tissues, including the skin (psoriasis [PsO] and atopic dermatitis [AD]), and the joints (rheumatoid arthritis [RA] and psoriatic arthritis [PsA], internal lumen (inflammatory bowel disease [IBD] and asthma) and white matter and gray matter (multiple sclerosis [MS]).^1^ Patients with IMID have a higher likelihood of developing another IMID and often present with comorbidities, such as cardiovascular, psychiatric, and peripheral artery disorders.^2^, ^3^, ^4^, ^5^, ^6^ The evolving understanding of the shared underlying pathogenesis of these clinically diverse diseases has led to a transition from organ-based to molecular-based classification, which was initiated by insights into associated key immune and inflammatory pathways and the development of cytokine targeted therapy, including monoclonal and bispecific antibodies, small interfering RNA (siRNA) therapeutics and chimeric antigen receptor (CAR)-T cell therapy.^7^, ^8^, ^9^

Over the past three decades, there has been a remarkable increase in human life expectancy and healthy life expectancy.^10^ Higher life expectancy at age 70 has led to a greater proportion of years spent in ill health at that age.^11^ Healthcare access and quality disparities persist worldwide, the Healthcare Access and Quality Index increased globally from 1990 to 2019, low-SDI countries had a significantly lower overall index of 30.7 compared to high-SDI countries with an index of 83.4.^12^ In 2019, the median physician density was ten times higher in high-SDI countries compared to low-SDI countries.^13^ Given that IMIDs represent a significant health concern, a refined trend analysis of IMIDs will aid in identifying and addressing the underlying causes of disparities in the diagnosis, treatment, and management of these diseases.

The Global Burden of Diseases (GBD), Injuries, and Risk Factors Study provided a systematic approach to assess the burden of IMIDs in 204 countries and territories, offering a unique opportunity to understand the underlying trends across the past three decades.^14^ In this study, we focused primarily on six major IMIDs, chosen due to the emergence of novel drugs and treatment strategies over the past few decades. Given the evolving healthcare needs of patients with IMIDs over their lifespan and medical advancements, this study aimed to i) estimate the pattern and trend of IMIDs incidence across the lifespan, ii) identify the global, regional, and national trends in IMIDs incidence from 1990 to 2019, and iii) determine the driving forces behind these trends.

## Methods

### Data sources

The GBD 2019 study is the most comprehensive and up-to-date source of epidemiological data, providing estimates for 369 diseases and injuries across 204 countries and territories from 1990 to 2019.^14^^,^^15^ Using standardized tools and a Bayesian framework, the study provides a detailed estimation of the incidence of IMIDs across all regions of the world. The accompanying GBD 2019 publications describe the data inputs, processing, synthesis, and final models used to estimate the disease burden of IMIDs.^11^^,^^15^ The GBD 2019 synthesizes a great number of input sources to estimate the incidence of IMIDs. The Data Input Sources Tool in Global Health Data Exchange (http://ghdx.healthdata.org/gbd-2019/data-input-sources) provides access to input sources for specific GBD components, causes and risks, and locations. The estimates and methods used in this study are publicly available from the Institute for Health Metrics and Evaluation website, including the GBD Compare tool (https://vizhub.healthdata.org/gbd-compare/) and the GBD Results Tool (http://ghdx.healthdata.org/gbd-results-tool).

### Data collection

Annual incident cases and age standardized incidences of IMIDs from 1990 to 2019, by sex, region, country, and cause (asthma, IBD, MS, RA, psoriasis, AD), were collected from the Global Health Data Exchange (GHDx) query tool (https://vizhub.healthdata.org/gbd-results/). Data from a total of 204 countries and territories were categorized into 5 regions in terms of socio-demographic index (SDI), including low, low-middle, middle, high-middle, and high and were separated into 21 regions in terms of geography.

### Socio-demographic index

The SDI is a composite index of socio-demographic development status strongly correlated with health outcomes, which is the geometric mean of 0–1 indices of total fertility rate in those under 25 years old, mean education for those age 15 years or older, and lag-distributed income per capita.^10^

### Statistical analysis

The study aimed to analyze the patterns and trends of major IMIDs using age-standardized incidence rate (ASR) and incident cases. The temporal trend was evaluated using a join-point regression model, and the average annual percent change (AAPC) was calculated for the study period. An increasing trend was determined if both the AAPC estimate and the lower boundary of its 95% confidence interval (CI) were >0, while a decreasing trend was established if both the AAPC estimate, and the upper boundary of its 95% CI were <0. Otherwise, the ASR was considered stable over time. The Join-point analysis of entire range (1990–2019), and three segment ranges (1990–1999, 2000–2009, 2010–2019) were used to reflect the full and local trend of IMIDs. To investigate the factors influencing AAPCs, the association between AAPCs and ASRs (1990) and SDI (2019) was assessed at the national level. All statistical analyses were conducted using Join-point Regression Program (Version 4.8.0.1, Statistical Methodology and Applications Branch, Surveillance Research Program, National Cancer Institute).^16^ A significance level of p < 0.05, at a two-tailed level, was used to determine statistical significance.

### Ethics statement

This study was produced as part of the GBD Collaborator Network and in accordance with the GBD Protocol (IHME ID 4239-GBD2019-042,022). For GBD studies, a waiver of informed consent was reviewed and approved by the Institutional Review Board of the University of Washington (https://www.healthdata.org/gbd/2019).

### Role of the funding source

The funders of this study had no role in study design, data collection, data analysis, data interpretation, and writing of the manuscript.

## Results

### The age effect on incidence of overall immune-mediated inflammatory disease

**Fig. 1:**
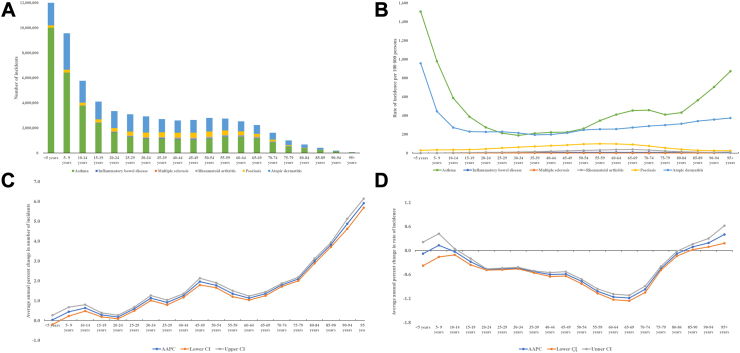
Cross sectional and longitudinal trend of incidence attributable to overall immune-mediated inflammatory diseases throughout human lifespan. The number of incident cases attributable to overall immune-mediated inflammatory diseases throughout human lifespan in 2019 (A), The rate of incidence attributable to overall immune-mediated inflammatory diseases throughout human lifespan in 2019 (B), The average annual percent change in number of indecent cases attributable to overall immune-mediated inflammatory diseases throughout human lifespan, 1990–2019 (C), The average annual percent change in rate of incidence attributable to overall immune-mediated inflammatory diseases throughout human lifespan, 1990–2019 (D).

Throughout human lifespan, the age distribution of incident cases from six IMIDs was quite different (sTable S1, Fig. 1A and B). Most incident cases were observed in individuals under the age of 25 for AD, in the age group of 20–59 years for IBD, in individuals aged 15–54 years for MS, among adults aged 30–69 years for RA, in individuals under the age of 69 for psoriasis. MS and RA did not affect children under the age of 5, while AD and asthma most frequently affected children under the age of 5. The age-specific-rate was highest among children under the age of 5, decreased with age, but increased again for individuals over the age of 80 for asthma and AD, increasing with age and plateauing at 40–44 years for IBD, increased rapidly, peaked at 25–29 years, and quickly turned to a decrease for MS, slowly increased, peaked at 65–69 years, and quickly turned to a decrease for RA, slowly increased, peaked at 55-55 years, and slowly turned to a decrease for psoriasis (sTable S2, sFigure S1, sFigure S2).

Throughout human lifespan, the AAPC of overall IMIDs new cases decreased with age, the AAPC of overall IMIDs incidence rate increased before 10 years, decreased during 10–65 yeas, reached nadir at 65–69 years, then turned to increase (sTable S1, Fig. 1C and D). Specifically, the AAPC of incident cases attributable to six individual IMIDs universally increased with age (sFigure S3). In contrast, the AAPC of incidence rate decreased with age, reached trough at 65–69 years, and turn to increase for asthma; fluctuant decrease with age for inflammatory bowel disease; fluctuant decrease with age, reached trough at 50–54 years, then turned to increase for MS; remained stale between 10 and 79 years, then turn to decrease for RA; slowly decrease, reached trough at 40–44 years, then turned to increase for psoriasis; fluctuant increase with age, peaked at 50–54 years, slightly decrease, reached trough at 75–79 years, then turn to increase for AD (sTable S2, sFigure S4).

### Global and regional incidence of overall immune-mediated inflammatory disease

In 2019, the global incidence of overall IMIDs was approximately 67,586,168 cases, with an ASR of 908.69 per 100,000 population. Throughout 5 SDI regions, 21 GBD region and 204 countries, the highest ASR was found in the High SDI region, High-income North America, United States of America, and the highest incident cases were recorded in Middle SDI region, South Asia, China, respectively (Table 1).

**Table 1:** The incident cases and age-standardized rate of incidence attributable to immune-mediated inflammatory diseases according to gender, cause, socio-demographic index regions, global burden of disease regions, and its temporal trends from 1990 to 2019.

	1990	2019	1990–1999	2000–2009	2010–2019	1990–2019
	N (95%CI)	N (95% CI)	AAPC (95% CI)	AAPC (95% CI)	AAPC (95% CI)	AAPC (95% CI)
**Age standardized rate**						
Overall	1013.74 (888.58–1169.42)	908.69 (786.07–1057.33)	−0.91 (−0.96, −0.87)	−0.28 (−0.37, −0.19)	0.19 (−0.17, 0.54)	−0.34 (−0.46, −0.22)
Male	936.49 (807.69–1098.63)	844.27 (717.13–1004.97)	−0.90 (−0.96, −0.84)	−0.13 (−0.33, 0.06)	0.11 (−0.26, 0.48)	−0.29 (−0.43, −0.15)
Female	1092.27 (966.58–1243.79)	973.24 (851.86–1115.20)	−0.93 (−0.97, −0.90)	−0.39 (−0.50, −0.27)	0.20 (0.11, 0.29)	−0.38 (−0.43, −0.33)
*Cause*						
Asthma	580.09 (474.68–715.04)	504.28 (400.64–633.26)	−1.47 (−1.55, −1.40)	−0.32 (−0.58, −0.07)	0.54 (0.05, 1.02)	−0.41 (−0.59, −0.23)
Inflammatory bowel disease	6.10 (5.35–6.96)	4.97 (4.43–5.59)	−1.65 (−1.71, −1.59)	−0.16 (−0.29, −0.03)	−0.32 (−0.39, −0.26)	−0.72 (−0.77, −0.67)
Multiple sclerosis	0.80 (0.70–0.90)	0.74 (0.65–0.83)	−0.68 (−0.71, −0.65)	−0.12 (−0.13, −0.11)	0.00 (−0.03, 0.03)	−0.26 (−0.27, −0.24)
Rheumatoid arthritis	12.21 (11.13–13.38)	13.00 (11.83–14.27)	0.27 (0.23, 0.31)	0.41 (0.40, 0.42)	−0.08 (−0.19, 0.02)	0.21 (0.18, 0.25)
Psoriasis	72.24 (69.70–74.72)	57.78 (55.76–59.71)	−0.72 (−0.72, −0.71)	−0.76 (−0.76, −0.75)	−0.84 (−0.85,−0.83)	−0.77 (−0.78, −0.76)
Atopic dermatitis	342.30 (327.04–358.42)	327.91 (312.76–343.67)	−0.04 (−0.06, −0.03)	−0.15 (−0.16, −0.14)	−0.27 (−0.28, −0.27)	−0.15 (−0.16, −0.14)
*SDI region*						
High SDI	1505.32 (1289.94–1773.48)	1441.66 (1225.91–1685.83)	−1.09 (−1.20, −0.98)	0.31 (0.20, 0.41)	0.24 (0.15, 0.33)	−0.14 (−0.21, −0.08)
High−middle SDI	1013.46 (878.70–1180.79)	931.65 (794.68–1094.39)	−0.61 (−0.84, −0.38)	−0.60 (−0.74, −0.45)	0.61 (0.32, 0.89)	−0.23 (−0.37, −0.09)
Middle SDI	965.01 (839.49–1123.07)	903.46 (775.97–1062.31)	−0.69 (−0.74, −0.65)	−0.38 (−0.43,−0.32)	0.40 (−0.05, 0.84)	−0.23 (−0.37, −0.09)
Low−middle SDI	843.92 (744.71–963.28)	766.86 (672.61–884.99)	−0.90 (−0.97, −0.84)	0.00 (−0.17, 0.17)	0.04 (−0.33, 0.42)	−0.27 (−0.41, −0.14)
Low SDI	859.27 (741.80–999.00)	798.54 (682.39–936.25)	−0.64 (−0.72, −0.56)	−0.06 (−0.14, 0.02)	0.07 (−0.19, 0.34)	−0.22 (−0.31, −0.13)
*GBD region*						
East Asia	894.11 (774.31–1047.27)	843.98 (726.72–1002.12)	−1.09 (−1.21, −0.98)	−0.77 (−0.91, −0.62)	1.12 (−0.44, 2.71)	−0.26 (−0.76, 0.24)
Southeast Asia	1137.98 (1007.04–1297.84)	1110.15 (974.02–1281.29)	−0.30 (−0.33, −0.27)	0.01 (−0.04, 0.05)	0.04 (−0.03, 0.11)	−0.10 (−0.13, −0.07)
Oceania	1312.62 (1158.42–1477.09)	1194.09 (1060.93–1340.98)	−0.11 (−0.14, −0.08)	−0.60 (−0.63, −0.57)	−0.22 (−0.28, −0.16)	−0.32 (−0.35, −0.29)
Central Asia	1116.28 (961.86–1299.42)	1077.72 (915.85–1273.98)	−0.16 (−0.18,−0.13)	−0.35 (−0.41,−0.30)	0.19 (0.14, 0.23)	−0.11 (−0.14,−0.09)
Central Europe	1011.85 (871.77–1191.64)	895.63 (746.27–1085.60)	−0.37 (−0.45, −0.30)	−0.69 (−0.73, −0.65)	−0.13 (−0.22, −0.03)	−0.42 (−0.46, −0.37)
Eastern Europe	998.65 (841.63–1192.99)	803.47 (658.87–977.26)	−0.68 (−0.80, −0.57)	−1.54 (−1.66, −1.42)	0.16 (−0.04, 0.36)	−0.73 (−0.82,−0.64)
High−income Asia Pacific	1466.17 (1281.85–1695.46)	1168.98 (995.26–1378.25)	−1.18 (−1.33, −1.03)	−1.43 (−1.54, −1.32)	0.29 (0.18, 0.40)	−0.84 (−0.91, −0.76)
Australasia	1338.01 (1138.99–1551.76)	1164.09 (984.84–1379.40)	0.24 (−0.01, 0.50)	−1.70 (−1.84, −1.55)	−0.07 (−0.19, 0.05)	−0.49 (−0.60, −0.38)
Western Europe	1353.20 (1210.93–1519.95)	1229.29 (1072.90–1402.16)	−0.70 (−0.97,−0.43)	−0.24 (−0.28,−0.21)	−0.09 (−0.29, 0.11)	−0.34 (−0.44,−0.23)
Southern Latin America	1255.43 (1101.48–1453.59)	1263.50 (1076.81–1496.65)	0.12 (0.00, 0.23)	0.00 (−0.01, 0.01)	0.00 (−0.01, 0.01)	0.03 (−0.01, 0.06)
High−income North America	1826.13 (1487.93–2270.75)	1911.37 (1603.78–2271.19)	−1.82 (−2.03, −1.61)	1.47 (1.32, 1.62)	0.69 (0.55, 0.82)	0.21 (0.10, 0.31)
Caribbean	1355.04 (1125.45–1626.43)	1283.19 (1060.07–1549.93)	−0.29 (−0.31, −0.28)	−0.29 (−0.31, −0.28)	0.12 (0.02, 0.22)	−0.17 (−0.20, −0.13)
Andean Latin America	1293.82 (1042.51–1583.94)	1118.89 (903.69–1402.55)	−0.68 (−0.73, −0.63)	−1.09 (−1.13, −1.06)	0.37 (0.31, 0.43)	−0.51 (−0.54, −0.48)
Central Latin America	982.24 (810.15–1192.05)	845.25 (673.58–1055.32)	−1.05 (−1.19,−0.92)	−0.66 (−0.78,−0.54)	0.25 (0.21, 0.28)	−0.53 (−0.59,−0.47)
Tropical Latin America	1476.43 (1178.40–1839.26)	1341.33 (1049.19–1666.04)	−0.48 (−0.66, −0.30)	−0.94 (−1.02, −0.87)	0.55 (0.35, 0.75)	−0.34 (−0.44, −0.25)
North Africa and Middle East	897.41 (768.64–1049.74)	851.41 (718.70–1007.79)	−0.29 (−0.32,−0.26)	−0.47 (−0.49,−0.45)	0.31 (0.22, 0.39)	−0.18 (−0.21,−0.15)
South Asia	733.14 (649.63–827.25)	672.56 (596.87–760.22)	−1.57 (−2.02,−1.13)	0.85 (0.19, 1.51)	−0.28 (−0.57, 0.00)	−0.34 (−0.61,−0.07)
Central Sub−Saharan Africa	816.85 (695.52–958.17)	741.82 (627.48–883.02)	−0.34 (−0.35, −0.33)	−0.49 (−0.57, −0.40)	−0.14 (−0.19, −0.09)	−0.33 (−0.36, −0.30)
Eastern Sub−Saharan Africa	943.32 (793.17–1131.35)	852.92 (708.08–1030.73)	−0.57 (−0.64, −0.50)	−0.48 (−0.50, −0.46)	0.06 (0.00, 0.13)	−0.34 (−0.37,−0.31)
Southern Sub−Saharan Africa	779.75 (633.85–944.95)	709.23 (576.11–868.27)	0.35 (−0.07, 0.76)	−3.54 (−4.77, −2.31)	3.28 (2.74, 3.83)	−0.06 (−0.53, 0.41)
Western Sub−Saharan Africa	789.05 (666.42–944.59)	715.11 (599.63–870.18)	−0.65 (−0.98, −0.32)	−0.41 (−0.47, −0.35)	0.00 (−0.20, 0.20)	−0.36 (−0.48, −0.23)
**Number of incident cases**						
Overall	55,906,499 (48,383,433–65,460,874)	67,586,168 (58,788,402–77,980,783)	0.13 (0.09, 0.17)	0.75 (0.63, 0.88)	1.13 (0.90, 1.37)	0.68 (0.59, 0.77)
Male	26,180,219 (22,235,358–31,447,992)	31,442,524 (26,888,533–37,097,214)	0.05 (−0.01, 0.11)	0.85 (0.67, 1.03)	1.07 (0.74, 1.41)	0.68 (0.55, 0.80)
Female	29,726,280 (26,136,610–34,237,346)	36,143,644 (31,908,458–40,987,243)	0.17 (0.14, 0.20)	0.67 (0.64, 0.70)	1.23 (1.14, 1.32)	0.68 (0.65, 0.72)
*Cause*						
Asthma	32,163,213 (25,752,792–40,513,127)	36,979,267 (29,601,976–45,928,112)	−0.52 (−0.60, −0.45)	0.61 (0.38, 0.84)	1.48 (1.04, 1.91)	0.53 (0.37, 0.70)
Inflammatory bowel disease	293,572 (257,425–336,651)	404,552 (360,521–456,478)	0.43 (0.30, 0.56)	1.67 (1.62, 1.71)	1.27 (1.21, 1.34)	1.11 (1.06, 1.16)
Multiple sclerosis	41,854 (36,306–47,445)	59,345 (51,818–66,943)	1.14 (1.10, 1.17)	1.36 (1.35, 1.37)	1.11 (1.08, 1.14)	1.21 (1.20, 1.23)
Rheumatoid arthritis	567,463 (519,417–621,415)	1,074,391 (975,502–1,179,332)	2.31 (2.28, 2.34)	2.47 (2.46, 2.48)	1.84 (1.75, 1.93)	2.22 (2.19, 2.25)
Psoriasis	3,653,236 (3,527,023–3,778,791)	4,622,594 (4,458,904–4,780,771)	0.98 (0.97, 1.00)	0.85 (0.84, 0.85)	0.61 (0.60, 0.63)	0.81 (0.80, 0.82)
Atopic dermatitis	19,187,161 (18,290,469–20,163,445)	24,446,018 (23,339,682–25,569,146)	0.93 (0.89, 0.97)	0.86 (0.86, 0.87)	0.72 (0.70, 0.74)	0.84 (0.82, 0.86)
*SDI region*						
High SDI	11,308,057 (9,894,762–13,026,205)	12,088,206 (10,695,628–13,675,720)	−0.87 (−0.96, −0.77)	0.79 (0.65, 0.93)	0.69 (0.60, 0.78)	0.25 (0.18, 0.32)
High−middle SDI	11,390,468 (9,919,040–13,277,351)	11,576,149 (10,234,863–13,168,363)	−0.38 (−0.61, −0.15)	−0.29 (−0.39, −0.19)	1.03 (0.82, 1.24)	0.09 (−0.02, 0.21)
Middle SDI	17,217,439 (14,695,850–20,511,806)	20,163,835 (17,515,593–23,332,662)	0.22 (−0.01, 0.46)	0.30 (0.20, 0.41)	1.29 (1.07, 1.50)	0.59 (0.47, 0.70)
Low−middle SDI	10,430,368 (9,006,974–12,270,814)	13,307,082 (11,596,864–15,359,307)	0.47 (0.42, 0.52)	1.26 (1.10, 1.41)	0.87 (0.58, 1.16)	0.90 (0.79, 1.01)
Low SDI	5,522,262 (4,623,631–6,700,074)	10,402,167 (8,603,854–12,719,419)	2.01 (1.93, 2.10)	2.69 (2.57, 2.81)	2.04 (1.74, 2.33)	2.25 (2.15, 2.36)
*GBD region*						
East Asia	10,690,192 (9,252,978–12,581,924)	11,270,729 (10,033,558–12,788,729)	−0.26 (−0.85, 0.34)	−0.58 (−0.84, −0.32)	1.83 (1.37, 2.29)	0.28 (0.00, 0.56)
Southeast Asia	5,520,353 (4,796,120–6,444,135)	7,160,601 (6,320,548–8,165,612)	0.91 (0.86, 0.95)	1.03 (0.96, 1.10)	0.74 (0.65, 0.83)	0.88 (0.84, 0.92)
Oceania	88,198 (75,687–102,467)	162,617 (142,217–186,085)	2.53 (2.48, 2.57)	1.72 (1.65, 1.78)	2.25 (2.09, 2.41)	2.15 (2.10, 2.21)
Central Asia	878,383 (742,184–1,047,408)	1,019,637 (864,469–1,210,312)	−0.38 (−0.50, −0.26)	0.17 (−0.09, 0.43)	1.83 (1.78, 1.89)	0.53 (0.44, 0.63)
Central Europe	1,207,366 (1,049,354–1,402,317)	869,178 (755,103–1,000,998)	−1.15 (−1.26,−1.05)	−1.53 (−1.58,−1.49)	−0.67 (−0.80, −0.53)	−1.14 (−1.20, −1.09)
Eastern Europe	2,162,990 (1,846,212–2,545,314)	1,382,432 (1,179,409–1,627,520)	−2.27 (−2.51, −2.04)	−2.37 (−2.46, −2.29)	0.20 (−0.03, 0.43)	−1.51 (−1.63,−1.39)
High−income Asia Pacific	2,258,416 (1,988,004–2,583,898)	1,691,690 (1,513,016–1,891,154)	−1.07 (−1.16, −0.97)	−1.86 (−1.96, −1.75)	0.01 (−0.06, 0.08)	−1.03 (−1.09, −0.98)
Australasia	243,038 (209,273–278,595)	286,422 (249,096–330,809)	1.00 (0.84, 1.16)	−0.31 (−0.41, −0.20)	1.01 (0.95, 1.06)	0.57 (0.50, 0.64)
Western Europe	4,763,790 (4,308,445–5,272,713)	4,565,937 (4,109,690–5,053,466)	−0.69 (−0.87, −0.51)	0.24 (0.13, 0.35)	−0.03 (−0.11, 0.05)	−0.19 (−0.26,−0.11)
Southern Latin America	633,000 (553,280–736,366)	775,180 (671,334–903,964)	0.79 (0.62, 0.95)	0.58 (0.51, 0.64)	0.82 (0.76, 0.87)	0.72 (0.66, 0.78)
High−income North America	4,662,400 (3,905,771–5,676,648)	5,910,905 (5,129,336–6,821,981)	−1.17 (−1.45, −0.89)	2.25 (2.05, 2.44)	1.17 (1.00, 1.34)	0.85 (0.71, 0.99)
Caribbean	524,424 (432,880–632,393)	557,437 (464,673–667,717)	0.22 (0.18, 0.27)	0.01 (−0.03, 0.04)	0.36 (0.10, 0.62)	0.19 (0.11, 0.27)
Andean Latin America	627,462 (495,373–777,248)	711,504 (575,085–893,222)	0.23 (0.14, 0.31)	−0.52 (−0.60, −0.45)	1.82 (1.75, 1.89)	0.44 (0.39, 0.49)
Central Latin America	1,926,142 (1,547,193–2,378,778)	1,998,679 (1,604,735–2,490,472)	0.14 (−0.10, 0.38)	−0.13 (−0.22, −0.04)	0.45 (0.42, 0.48)	0.13 (0.05, 0.21)
Tropical Latin America	2,534,797 (1,993,882–3,218,798)	2,557,679 (2,044,355–3,124,198)	−0.01 (−0.17, 0.14)	−0.54 (−0.61, −0.48)	0.72 (0.56, 0.87)	0.01 (−0.07, 0.09)
North Africa and Middle East	3,496,870 (2,902,019–4,224,006)	5,108,802 (4,286,740–6,082,702)	1.39 (1.31, 1.46)	1.02 (0.99, 1.05)	1.59 (1.49, 1.69)	1.32 (1.28, 1.36)
South Asia	8,465,092 (7,460,147–9,802,521)	11,614,829 (10,330,682–13,169,438)	−0.02 (−0.48, 0.43)	2.61 (1.95, 3.28)	0.65 (0.37, 0.93)	1.07 (0.79, 1.34)
Central Sub-Saharan Africa	574,128 (463,728–704,268)	1,163,850 (945,928–1,431,971)	2.50 (2.47, 2.52)	2.55 (2.52, 2.58)	2.36 (2.32, 2.41)	2.47 (2.45, 2.49)
Eastern Sub-Saharan Africa	2,337,638 (1,878,499–2,929,628)	4,284,839 (3,413,453–5,375,525)	1.98 (1.84, 2.13)	2.12 (2.07, 2.16)	2.28 (2.14, 2.41)	2.12 (2.05, 2.19)
Southern Sub-Saharan Africa	450,736 (356,336–561,184)	554,527 (447,968–685,629)	1.91 (1.42, 2.39)	−3.15 (−4.52, −1.75)	4.57 (3.98, 5.17)	1.01 (0.48, 1.55)
Western Sub-Saharan Africa	1,861,085 (1,503,020–2,320,718)	3,938,693 (3,155,961–4,963,753)	2.39 (2.26, 2.52)	2.99 (2.87, 3.11)	2.49 (2.46, 2.53)	2.59 (2.52, 2.65)

**Fig. 2:**
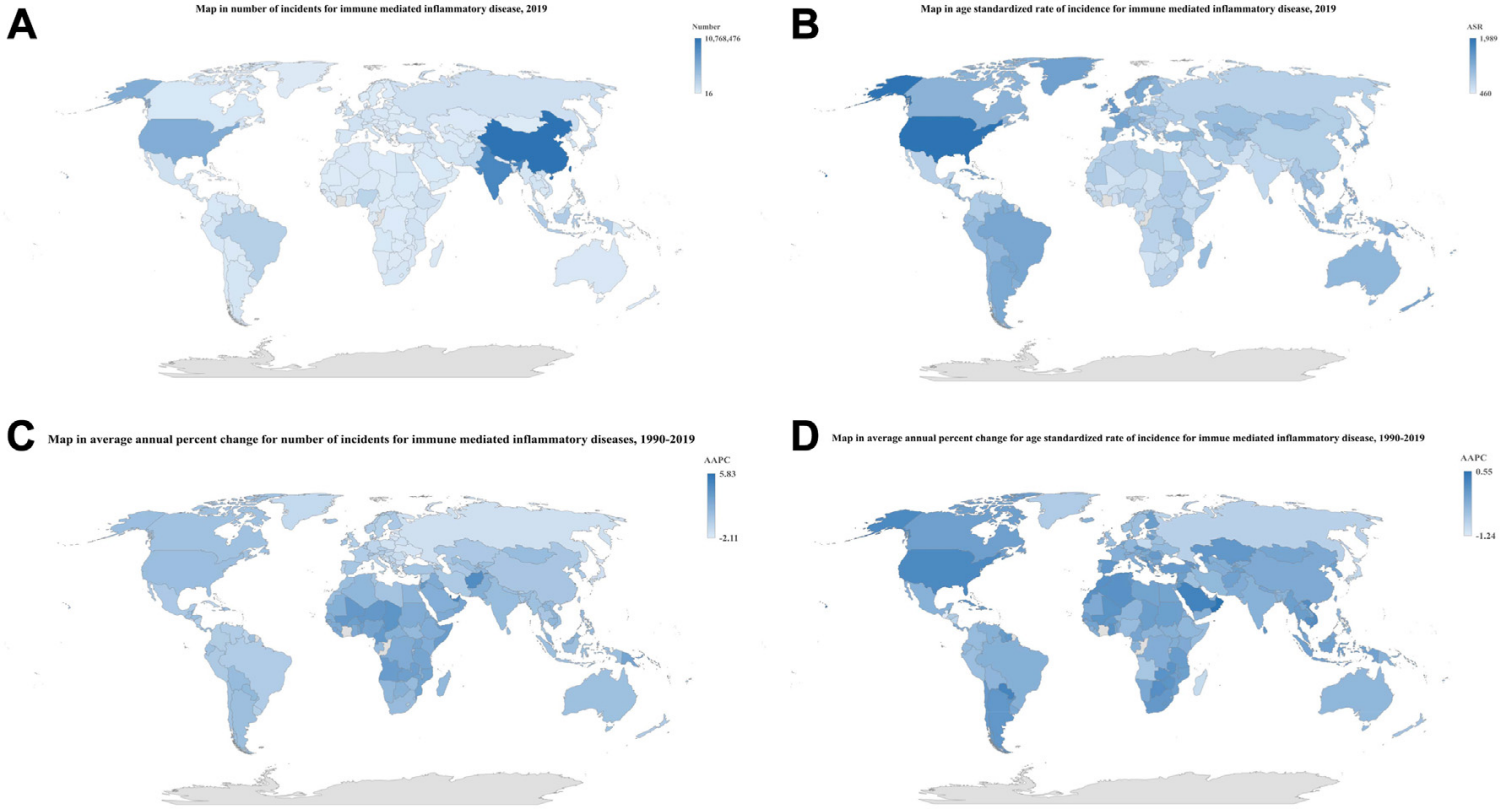
The global map of incidence attributable to overall immune-mediated inflammatory diseases in 204 countries and territories for both sexes combined. The global map in number of incident cases attributable to overall immune-mediated inflammatory diseases, 2019 (A). The global map in age standardized rate of incidence attributable to overall immune-mediated inflammatory diseases, 2019 (B), The global map in average annual percent change in number of incident cases attributable to overall immune-mediated inflammatory diseases, 1990–2019 (C). The global map in average annual percent change in age standardized rate of incidence attributable to overall immune-mediated inflammatory diseases, 1990–2019 (D).

From 1990 to 2019, the global incident cases increased with an AAPC of 0.68, but the ASRs decreased with an AAPC of −0.34. The ASRs decreased in all five SDI regions from 1990 to 2019, with the quickest decline in the Low-middle SDI region (AAPC = −0.27). Across the regional and national region, the most rapid increase of ASR was observed in High-income North America (AAPC = 0.21) and Oman (AAPC = 0.55), the most rapid increases of incident cases were Western Sub-Saharan Africa (AAPC = 2.59) and Qatar (AAPC = 5.83) (Table 1, Fig. 2C and D, sFigure S5, sTable S3).

### Incidence of six immune-mediated inflammatory disease according to gender and proportion

**Fig. 3:**
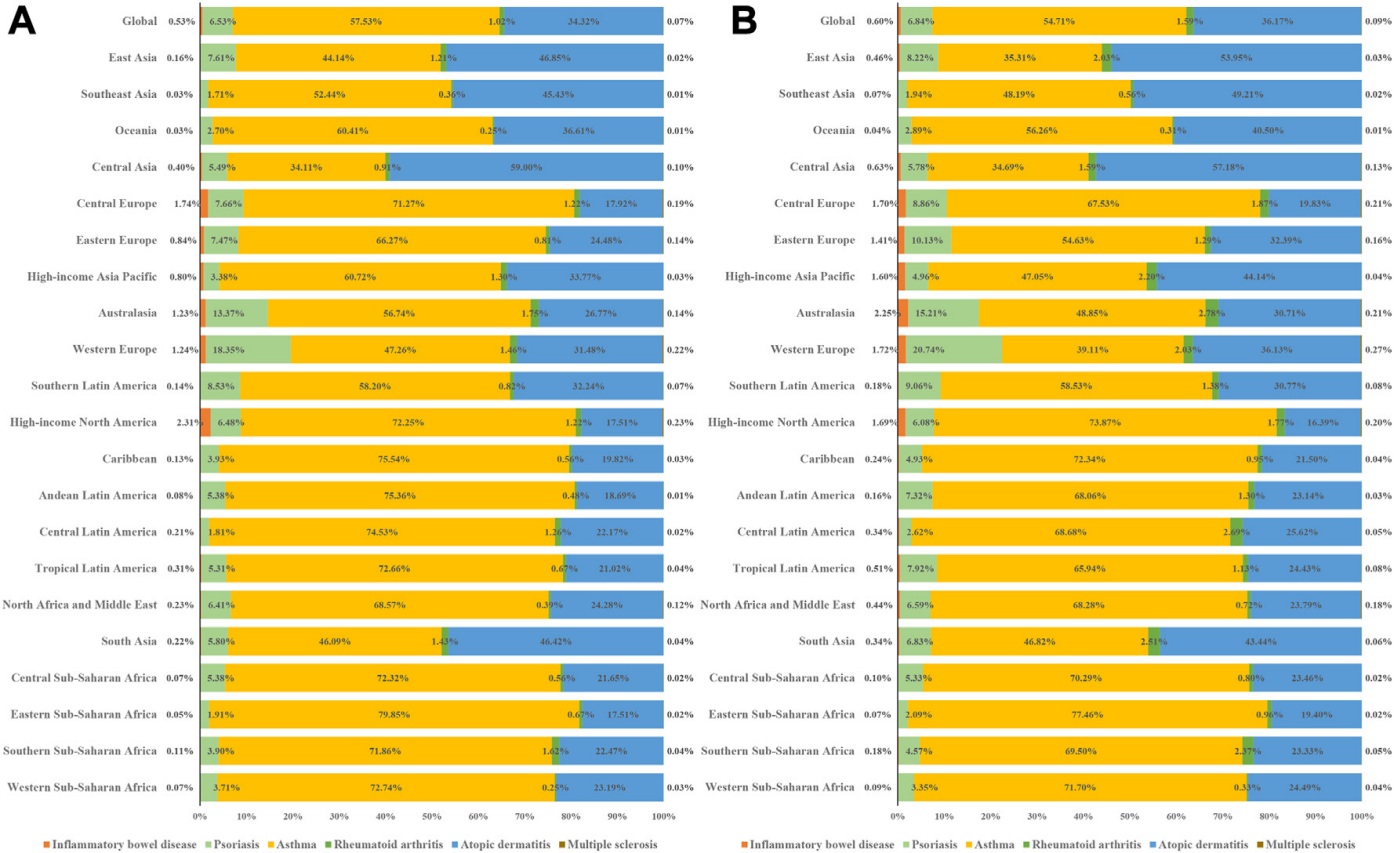
Contribution of incident cases from six individual to overall immune-mediated inflammatory diseases, both sexes, globally and by region, 1990 and 2019. Contribution of incident cases from six individual to overall immune-mediated inflammatory diseases, both sexes, globally and by region, 1990 (A), Contribution of incident cases from six individual to overall immune-mediated inflammatory diseases, both sexes, globally and by region, 2019 (B).

Compared with males, females have more than twice likelihood to develop RA, have modestly higher likelihood to develop AD, have comparable possibility to develop asthma, have more considerable likelihood to develop MS, have similar possibility to develop psoriasis, has slight lower likelihood to IBD. (sFigure S6). In 1990 and 2019, RA, AD, asthma, MS, psoriasis, IBD accounted for 1.02%, 34.32%, 57.53%, 0.07%, 6.53%, 0.53% and 1.59%, 36.17%, 54.71%, 0.09%, 6.84%, 0.60% of overall new IMIDs cases, respectively. In 2019, this proportion exceeded 2.51% for RA in South Asia, comprised as much as 0.27% for MS in certain high-SDI regions, such as Western Europe, reached 1.70% for IBD in Central Europe (Table 1, Fig. 3).

### Incidence of six immune-mediated inflammatory disease according to global, SDI and GBD region

From 1990 to 2019, the global ASR significantly increased for RA (AAPC = 0.21), albeit the ASRs significantly decreased for AD (AAPC = −0.15), asthma (AAPC = −0.41), MS (AAPC = −0.26), psoriasis (AAPC = −0.77), IBD (AAPC = −0.32). Among the six IMIDs, the global new cases increased the fastest for RA (AAPC = 2.22), followed by MS (AAPC = 1.21), IBD (AAPC = 1.11), AD (AAPC = 0.84), psoriasis (AAPC = 0.81), asthma (AAPC = 0.53) (Table 1).

The ASR increased across 5 SDI regions for RA, decreased in four out of five SDI regions for AD except for High-middle SDI, was only decreased in the High-middle SDI region for MS, generally decreased across the 5 SDI regions for asthma and psoriasis, increased fastest in middle SDI regions for IBD. The incident cases generally increased across the five SDI regions for RA, AD, MS, psoriasis, IBD, increased in all five regions except High-middle SDI for asthma (sTable S4).

Of the 21 geographical regions, the most significant increase in ASR was observed in Andean Latin America (AAPC = 1.36) for RA, in Eastern Europe (AAPC = 0.07) for AD, in High-income North America (AAPC = 0.37) for asthma, in Australasia (AAPC = 1.05) for MS, in East Asia (AAPC = 2.48) for IBD. The ASR of psoriasis unanimously decreased among the 21 regions, with the highest decrement observed in North Africa and the Middle East (AAPC = −0.90). The incident cases increased across 21, 18, 12, 21, 19, 21 regions for RA, AD, asthma, MS, psoriasis, IBD, with highest increase in Andean Latin America (AAPC = 3.91), Western Sub-Saharan Africa (AAPC = 2.82), Western Sub-Saharan Africa (AAPC = 2.51), Western Sub-Saharan Africa (AAPC = 3.61), Eastern Sub-Saharan Africa (AAPC = 2.42), Central Sub-Saharan Africa (AAPC = 3.90), respectively (sTable S5, sFigure S7, sFigure S8).

### Incidence of six immune-mediated inflammatory disease according to countries and territories

At the national level, the highest ASR of RA, AD, asthma, MS, psoriasis, IBD were observed in Ireland, Mongolia, United States of America, Sweden, France, Canada, while the highest incident cases were recorded in India, China, India, United States of America, China, United States of America for RA, AD, asthma, MS, psoriasis, IBD, respectively. The fastest increasing trend in ASR of RA, AD, asthma, MS, IBD were Peru (AAPC = 1.43), Kenya (AAPC = 0.16), Oman (AAPC = 0.93), Taiwan (Province of China) (AAPC = 1.55), Taiwan (Province of China) (AAPC = 3.20), respectively. The ASR of psoriasis remained stable in Japan and Somalia but decreased in other 202 countries and territories from 1990 to 2019. The most significant decline in ASR was observed in Equatorial Guinea (AAPC = −1.66). The most significant increase of new cases across six IMIDs was observed in Qatar (AAPC: RA = 8.32, AD = 5.85, asthma = 5.68, MS = 8.82, psoriasis = 6.14, IBD = 8.24) (sTable S6, sFigure S9–S12).

### The association between ASR, SDI and AAPC

**Fig. 4:**
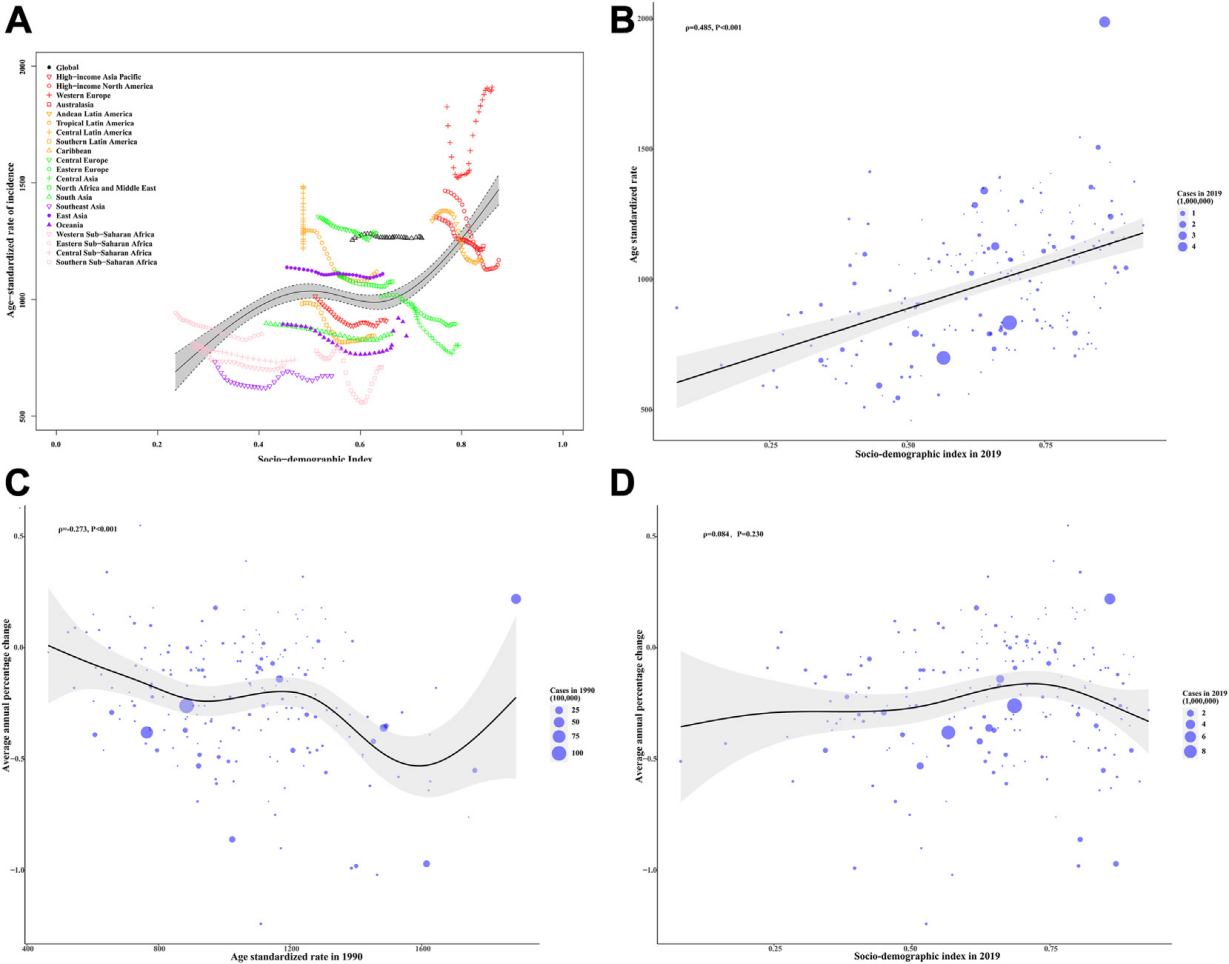
The association between age standardized rate of incidence, socio-demographic index, average annual percent change across global burden of disease regions and countries and territories. Age standardized rate of incidence attributable to overall immune-mediated inflammatory diseases per 100,000 persons for socio-demographic index by 21 global burden of diseaseregions, 2019 (A). Black line represents expected values based on socio-demographic index and disease rates across 21 global burden of disease regions; each point shows observed age standardized rate of incidence for specified global burden of diseaseregion in 2019. Age standardized rate of incidence attributable to overall immune-mediated inflammatory diseases per 100,000 persons for socio-demographic index by 204 countries and territories, 2019(B). Black line represents expected values based on socio-demographic index and disease across 204 countries and territories, each point shows observed age standardized rate of incidence for specified country in 2019. The correlation between average annual percent change and age standardized rate of incidence attributable to overall immune-mediated inflammatory in 1990 across 204 countries and territories (C). The size of circle is increased with the incident cases of immune-mediated inflammatory diseases. The ρ indices and p values were derived from Pearson correlation analysis. The correlation between average annual percent change and socio-demographic index attributable to overall immune-mediated inflammatory in 2019 across 204 countries and territories (D). The size of circle is increased with the incident cases of immune-mediated inflammatory diseases. The ρindices and p values were derived from Pearson correlation analysis.

In 2019, the ASR of overall IMIDs increased with the SDI across 21 regions and 204 countries and territories (Fig. 4A and B). This increasing trend was also observed for six individual IMIDs at the regional and global levels (sFigure S13, sFigure S14).

From 1990 to 2019, countries with higher ASR in 1990 showed a more rapid decrease in ASR of overall IMIDs for an ASR below 1600 per 100,000 (Fig. 4-C). The ASR of IMIDs in 1990 reflects the disease reservoir at baseline, while the SDI in 2019 can serve as a surrogate for the level and availability of healthcare in each country. This decreasing trend was also found in four IMIDs, with the most notable being RA. However, the trend was reversed in psoriasis and MS (sFigure S15).

Throughout all regions and countries, those with higher SDI have experienced a more rapid increase in ASR of overall IMIDs from 1990 to 2019 (Fig. 4D). The SDI in 2019 serves as a surrogate for the level and availability of healthcare in each country. A similar increasing trend was also found for IBD, MS, and psoriasis, while a reverse trend was observed for asthma, RA, and AD at the global level (sFigure S16).

## Discussion

This study provides a comprehensive estimation of the incidence of IMIDs and investigates their temporal trend by gender, age, SDI, and global-regional-national levels for the first time. The magnitude of incident cases of IMIDs increased, probably driven by population growth and ageing, societal development, interaction between genetic and environmental factors. The global population has risen from 5.3 billion in 1990 to 7.7 billion in 2019, the population aged 70–79 years increased by 115.4%, aged 80–94 years increased by 164.7%, and ≥95 years increased by 363.7%, respectively.^11^

The ideal efforts to prevent the onset or redirect the course of IMIDs should focus on modifying environmental or behavioral factors.^17^ The accumulation of environmental exposures and lifestyle factors that can trigger genetic predisposition underlying immune response over time.^18^, ^19^, ^20^ Exposure to environmental air pollution above the threshold for human protection was associated with a 10% higher risk of developing IMIDs.^21^ The hygiene hypothesis postulates that the increase in the incidence of IMIDs was caused by the reduced exposure to infectious agents, probably explains the rising development of IMID's in low-middle-SDI countries, where there has been a steady decline in microbes and parasites over the past thirty years.^22^ For example, exposure to agricultural farming and poultry is associated with the asthma-protective effect in the rural area.^23^ Indeed, hygiene hypothesis cannot fully explain autoimmunity, there is no strong evidence linking the hygiene hypothesis to rheumatoid arthritis.^24^ In addition, industrial PM2.5 associated with the risk of systemic autoimmune rheumatic diseases and air pollution may be a trigger factor for psoriasis flare.^25^^,^^26^ However, the impact of lifestyle changes in preventing the development of systemic autoimmunity in rheumatoid arthritis, such as smoking cessation, dietary changes, weight reduction, has been partially established.^27^ Furthermore, the rise in overweigh and obesity has paralleled the increase in the incidence of IMIDs, which might be explained by the overstimulation of T lymphocytes by nutrient- and energy-sensing pathways and loss of self-tolerance by metabolic overload.^28^

Preventive strategy is urgently needed to address the rising ASR of RA. Currently available treatment did not shown to prevent the development of RA in individuals at high risk, early treatment with rituximab and abatacept only delayed onset of full-blown RA.^29^ Similarly, the TREAT EARLIER study found that early intervention with methotrexate and glucocorticoid treatment did not prevent the development of clinical arthritis.^30^ Ongoing investigations into preventive interventions that interfere with altered activation of the adaptive immune system, such as ARIAA (EudraCT 2014-000555-93) and APIPPRA (EudraCT 2013-003413-18), may provide further insight into the feasibility of preventing RA in the future.^31^^,^^32^

A reoriented management strategy and more targeted drugs are needed to control the disease burden from the rapid increase in incident cases of IMIDs as traditional therapeutic modalities, including biologic and small-molecule therapies, is not a concern in patients with IMIDs. New methods are urgently needed to choose tailored formulation and frequency of administration with the highest probability of acceptance and to limit unnecessary use of medication.^33^, ^34^, ^35^ The Allergic March story tell us which subtype of AD are at risk progresses to asthma, which might be enhanced when allergic sensitization presents at early life,^36^^,^^37^ long term study was needed to investigate whether the new biologics that interact with classic type 2 inflammatory process truly interrupt the atopic march.^38^ The 2021 asthma recommendation from the Global Initiative for Asthma (GINA) emphasizes the use of symptoms and side-effect preventive modifiers instead of relying on side-effect prone and regular use of relievers.^39^ Whether modern steroid-sparing anti-inflammatory treatments are disease modifiers remain controversy as the therapeutic effect of allergen immunotherapy is modest and therapeutic effects of biologics targeting IgE, IL-5, IL-4, IL-13 are maintained in in adults with established asthma.^40^, ^41^, ^42^ Although biologic agents can be effective add-on therapies for patients with type 2–high severe asthma, no biologic drugs are currently available for type 2-low severe asthma.^43^ However, the recent success of tezepelumab, which targets the epithelial alarmin thymic stromal lymphopoietin, is an encouraging development.^44^

Appropriate disease-modifying therapies should be considered in patients with MS and comorbid autoimmune diseases due to the autoimmunity partially overlap with other autoimmune mediated disorders.^45^ More innovative therapies are needed to prevent neurodegeneration and reverse structural damage.^46^ The domain-driven treatment approach aims to address all active domains of psoriatic disease and related conditions.^47^ Larger and longer trials are needed to assess the comparative efficacy and safety of tapinarof (1%) and roflumilast cream (0.3%) in patients with skin psoriasis,.^48^^,^^49^ While head-to-head trials have shown dual blockade of IL-17A and IL-17F to be superior to biologic targeting IL-17A, IL-12/23, TNF-α in patients with psoriasis, further data is required to confirm whether this finding replicated in patients with PsA.^50^^,^^51^ Considering the potential benefits of small molecules over monoclonal antibodies, the next leap forward in treating psoriasis and PsA might be small molecule modulators targeting IL-17A/IL-17RA.^52^ Exciting preliminary data confirms this leap, as deucravacitinib demonstrated superiority over apremilast in patients with psoriasis,^53^ and upadacitinib was superior to adalimumab in patients with PsA.^54^ Although currently available therapeutic armamentarium resulting in somewhat durable remission in patents with ulcerative colitis, the treatment goal of corticosteroid-free clinical remission was hardly achieved with biologic and small-molecule therapies targeting TNF-α, 4β7 integrin, JAK, S1P, TYK2, etc.^55^ Although the effect on small bowel lesions remain unclear, risankizumab represents a promising and favorable option for patients with Crohn's disease who still have unmet needs.^56^

Emerging novel therapeutic modality opens a window on exit strategies of biologic therapy as both patients and clinicians hope to avoid the undesirable consequences of long-term biologic therapy. Effective improvement in AD have been observed in mesenchymal stem cell (MSC) based therapy, although more research is needed to determine optimal dosages, ideal administration routes and standard methods.^57^ A phase 1/2a single-arm study showed that intravenous infusions of umbilical cord mesenchymal stem cells were could partially be effective in treating psoriasis.^58^ Autologous haemopoietic stem cell transplantation (aHSCT), allogeneic neural stem-cell-based therapy, and CAR-Tregs targeting myelin oligodendrocyte glycoprotein were effective for inducing remissions of active relapsing remitting MS, although long follow-ups and head-to-head comparisons with the most effective disease-modifying treatments are necessary to understand how to position them for the management of patients with aggressive MS.^59^, ^60^, ^61^, ^62^ Novel vaccines that prevent EBV infection or targeting EBV would be expected to prevent most new cases or represent a novel treatment strategy for MS.^63^ Although the failure of Seres Therapeutics' microbiome-based candidate SER-287 in ulcerative colitis casting a shadow over the emerging field,^64^ more data on functional effects of individual and groups of microbes on the mucosal immune system might lead to new microbiota-based therapies.^65^^,^^66^ Local treatment with adipose-derived mesenchymal stem cells (Cx601) added on to established treatments for Crohn's disease represents a novel and minimally invasive alternative for complex perianal fistulas.^67^^,^^68^ The long-term efficacy and time frame for retreatment are currently being investigated in the INSPIRE trial (EUPAS24267).^55^

Future analyses of the IMIDs should prioritize examining the direct and indirect effects of the COVID-19 pandemic. While the pandemic threatens healthcare access and quality gains achieved at all ages, it poses a particularly grave risk to older individuals who account for most COVID-19 deaths.^69^ However, the pandemic has also catalyzed innovation in the provision of health care, including an expansion in the use of telemedicine.^70^ Recent data strongly suggest that patients with IMID require a third SARS-CoV-2 vaccination, future study should ascertain whether fourth and beyond doses should be given as new boosters become available.^71^^,^^72^ Immunocompromising therapies for IMIDs, such as TNF-α inhibitors, are not associated with a significantly greater risk of SARS-CoV-2 or severe sequelae and may even be associated with a lower risk of adverse COVID-19 outcomes.^73^^,^^74^ In addition to the impact of immune-modulatory medications that were used in severe cases of COVID-19, on the developing, recurring, or improving the IMIDS, it is necessary to capture the trends at different stages of the pandemic. For example, there may be a surge in incidents as the post-COVID-19 era progresses and diagnoses return to normal levels. The increased attention to One Health after the COVID-19 pandemic is an opportunity to focus efforts and resources on IMIDs, which can strengthen multisectoral coordination mechanisms at national, regional, and global levels.^75^^,^^76^

The previous analyses of the GBD study have highlighted its limitations.^11^^,^^15^ The major limitation of the analysis of the incidence of six IMIDs is the sources vary substantially and out-of-sample modelling data where primary data are not available. Although the data for the modelling on the incidence of IMIDs comes from scientific literature, national surveys, claims data, data were excluded if they violated established regional trends and age distributions, if they led to overestimation of subnational pseudo-random effects and poor model fit. The GBD study tried to include all available data to modeling the global-regional-national incidence of IMIDs but part data were marked as outliers and excluded if they were implausibly high or low relative to global or regional patterns, substantially conflicted with established age or temporal patterns, significantly conflicted with other data sources conducted from the same locations or locations with similar socio-demographic index. Five additional limitations have been identified. Firstly, the current estimates of the incidence of IMIDs do not reflect the impact of the COVID-19 pandemic. Secondly, underreporting of IMID incidence in low- and middle-income countries may occur due to inadequate reporting mechanisms and infrastructure in some regions. However, the incidence rates of some countries may have been overestimated as they were based on data from major cities. Thirdly, the study does not include a comprehensive list of IMIDs, such as systemic lupus erythematosus, scleroderma, and primary Sjogren's syndrome, Muckle Wells syndrome. Fourthly, the physician density, healthcare access, the quality of medical training might influence the diagnosis of different IMIDs, especially the incidence of IMIDs might overestimate in developed countries and underestimate in developing countries. Fifthly, as inpatients with IMIDs are severely affected patients, further analysis of inpatient data with IMIDs could reflect refractory disease burden and difference of disease burden between inpatient and outpatient.

We recommend four areas of work that need priority in future research. Firstly, it should incorporate how intercept interventions impact the incidence of new cases. Secondly, it should explore the reasons behind these epidemiological transitions. Thirdly, it is vital to train health care providers in the use of up-to-date therapeutics. Finally, it should prioritize investments and cost-effective healthcare to address the substantial unmet healthcare needs.

## Contributors

Dongze Wu, Yingzhao Jin, Cui Guo, and Lai-shan Tam had full access to all the data in the study and directly accessed and verified the underlying data reported in the manuscript. All authors had access to, reviewed estimates, and agree to submit the manuscript. Please see appendix (Authors’ contributions) for more detailed information about individual author contributions to the research, divided into the following categories: managing the overall research enterprise; writing the first draft of the manuscript; primary responsibility for applying analytical methods to produce estimates; primary responsibility for seeking, cataloguing, extracting, or cleaning data; designing or coding figures and tables; providing data or critical feedback on data sources; developing methods or computational machinery; providing critical feedback on methods or results; drafting the manuscript or revising it critically for important intellectual content; and managing the estimation or publications process.

## Data sharing statement

Data used for the analyses are publicly available from the Institute of Health Metrics and Evaluation (http://www.healthdata.org/; http://ghdx.healthdata.org/gbd-results-tool).

## Declaration of interests

K Abuabara reports grants or contracts from Pfizer and Cosmetique Internacional SNC to their institution, University of California San Francisco; consulting fees from TARGET RWE; outside the submitted work. S Bhaskar reports leadership or fiduciary role in other board, society, committee or advocacy group, paid or unpaid, with the Rotary Club or Sydney as Board Director and Chair of Youth, with Rotary District 9675 as Chair of Diversity, Equity and Inclusion, and with Global Hub Health Germany as Founding Member and Co-manager, all outside the submitted work. R Buchbinder reports grants from Australian National Health and Medical Research Council (NHMRC), Arthritis Australia, Cabrini Foundation, HCF Foundation, Australian Department of Health to their institution; royalties or licenses from UptoDate as personal payments for a chapter on plantar fasciitis; all outside the submitted work. A K Demetriades reports leadership or fiduciary role in other board, society, committee or advocacy group, paid or unpaid, with European Association of Neurosurgical Societies (EANS) as President and with Global Neuro Foundation as Board Member, all outside the submitted work. I Filip and A Radfar report payment or honoraria for lectures, presentations, speakers bureaus, manuscript writing or educational events from Avicenna Medical and Clinical Research Institute. T Fukumoto reports payment or honoraria for lectures, presentations, speakers bureaus, manuscript writing or educational events from AbbVie, Eli Lilly, Sanofi, Pfizer, Maruho, Novartis, Taiho, Sun Pharma, UCB, and Janssen Pharma, all outside the submitted work. C Herteliu reports a research grant from Romanian Ministry of Research Innovation and Digitalization, MCID, for project titled “Enhancing institutional performance through development of infrastructure and transdisciplinary research ecosystem within socio-economic domain–PERFECTIS,” project number ID-585-CTR-42-PFE-2021, outside the submitted work. N Ismail reports leadership or fiduciary role in other board, society, committee or advocacy group, unpaid, with the Malaysian Academy of Pharmacy as council member and bursar, outside the submitted work. K Krishan reports non-financial support from UGC Centre of Advanced Study, CAS II, Department of Anthropology, Panjab University, Chandigarh, India, outside the submitted work. V Shivarov reports a pending Bulgarian patent for Possible SARS-CoV-2 preimmune epitopes; stock or stock options in ICON PLC through restricted stock units; other financial interests from PRAHS/ICON PLC through their salary; all outside the submitted work. C R Simpson reports research grants from MBIE (NZ), HRC (NZ), Ministry of Health (NZ), MRC (UK), HDRUK, and CSO (UK) to their institution, all outside the submitted work. J A Singh reports consulting fees from Crealta/Horizon, Medisys, Fidia, PK Med, Two Labs Inc., Adept Field Solutions, Clinical Care Options, Clearview Healthcare Partners, Putnam Associates, Focus Forward, Navigant Consulting, Spherix, MedIQ, Jupiter Life Science, UBM, Trio Health, Medscape, WebMD, Practice Point Communications, the National Institutes of Health, and the American College of Rheumatology all as personal payments; payment or honoraria for speakers' bureaus from Simply Speaking; support for attending meetings or travel from the steering committee of OMERACT; unpaid participation on a Data Safety Monitoring Board or Advisory Board with the US Food and Drug Administration Arthritis Advisory Committee; leadership or fiduciary role in board, society, committee or advocacy group, paid or unpaid, with OMERACT as a steering committee member, with the Veterans Affairs Rheumatology Field Advisory Committee as Chair (unpaid), and with the UAB Cochrane Musculoskeletal Group Satellite Center on Network Meta-analysis and editor and director (unpaid); stock or stock options in Atai Life Sciences, Kintara Therapeutics, Intelligent Biosolutions, Acumen Pharmaceutical, TPT Global Tech, Vaxart Pharmaceuticals, Atyu Biopharma, Adaptimmune Therapeutics, GeoVax Labs, Pieris Pharmaceuticals, Enzolytics Inc., Seres Therapeutics, Tonix Pharmaceuticals Holding Corp., and Charlotte's Web Holdings, Inc., and previously owned stock options in Amarin, Viking, and Moderna Pharmaceuticals; all outside the submitted work. M Zielińska reports other financial or non-financial interests as an employee of AstraZeneca, outside the submitted work. E Upadhyay reports a published patent for A system and method of reusable filters for anti-pollution mask, A system and method for electricity generation through crop stubble by using microbial fuel cells, A system for disposed personal protection equipment (PPE) into biofuel through pyrolysis and method, A novel herbal pharmaceutical aid for formulation of gel and method thereof, and reports leadship for Joint Secretary of Indian Meteorological Society, Jaipur Chapter, India, Member Secretary-DSTPURSE Program.

## References

[1] McInnes I.B., Gravallese E.M. (2021). Immune-mediated inflammatory disease therapeutics: past, present and future. Nat Rev Immunol.

[2] El-Gabalawy H., Guenther L.C., Bernstein C.N. (2010). Epidemiology of immune-mediated inflammatory diseases: incidence, prevalence, natural history, and comorbidities. J Rheumatol Suppl.

[3] Baena-Diez J.M., Garcia-Gil M., Comas-Cufi M. (2018). Association between chronic immune-mediated inflammatory diseases and cardiovascular risk. Heart.

[4] Marrie R.A., Walld R., Bolton J.M. (2018). Psychiatric comorbidity increases mortality in immune-mediated inflammatory diseases. Gen Hosp Psychiatry.

[5] Aguero F., Gonzalez-Zobl G., Baena-Diez J.M. (2015). Prevalence of lower extremity peripheral arterial disease in individuals with chronic immune mediated inflammatory disorders. Atherosclerosis.

[6] Conrad N., Verbeke G., Molenberghs G. (2022). Autoimmune diseases and cardiovascular risk: a population-based study on 19 autoimmune diseases and 12 cardiovascular diseases in 22 million individuals in the UK. Lancet.

[7] Schett G., McInnes I.B., Neurath M.F. (2021). Reframing immune-mediated inflammatory diseases through signature cytokine hubs. N Engl J Med.

[8] Sargazi S., Arshad R., Ghamari R. (2022). siRNA-based nanotherapeutics as emerging modalities for immune-mediated diseases: a preliminary review. Cell Biol Int.

[9] Zmievskaya E., Valiullina A., Ganeeva I., Petukhov A., Rizvanov A., Bulatov E. (2021). Application of CAR-T cell therapy beyond Oncology: autoimmune diseases and viral infections. Biomedicines.

[10] Collaborators, G.B.D.D. (2020). Global age-sex-specific fertility, mortality, healthy life expectancy (HALE), and population estimates in 204 countries and territories, 1950-2019: a comprehensive demographic analysis for the Global Burden of Disease Study 2019. Lancet.

[11] Collaborators, G.B.D.A. (2022). Global, regional, and national burden of diseases and injuries for adults 70 years and older: systematic analysis for the Global Burden of Disease 2019 Study. BMJ.

[12] (2022). Access, G.B.D.H. and C. Quality, Assessing performance of the Healthcare Access and Quality Index, overall and by select age groups, for 204 countries and territories, 1990-2019: a systematic analysis from the Global Burden of Disease Study 2019. Lancet Glob Health.

[13] Collaborators, G.B.D.H.R.f.H. (2022). Measuring the availability of human resources for health and its relationship to universal health coverage for 204 countries and territories from 1990 to 2019: a systematic analysis for the Global Burden of Disease Study 2019. Lancet.

[14] Diseases, G.B.D. and C. Injuries (2020). Global burden of 369 diseases and injuries in 204 countries and territories, 1990-2019: a systematic analysis for the Global Burden of Disease Study 2019. Lancet.

[15] Collaborators, G.B.D.R.F. (2020). Global burden of 87 risk factors in 204 countries and territories, 1990-2019: a systematic analysis for the Global Burden of Disease Study 2019. Lancet.

[16] Clegg L.X., Hankey B.F., Tiwari R., Feuer E.J., Edwards B.K. (2009). Estimating average annual per cent change in trend analysis. Stat Med.

[17] Bieber K., Hundt J.E., Yu X. (2023). Autoimmune pre-disease. Autoimmun Rev.

[18] Forbes J.D., Chen C.Y., Knox N.C. (2018). A comparative study of the gut microbiota in immune-mediated inflammatory diseases-does a common dysbiosis exist?. Microbiome.

[19] Vetrano S., Bouma G., Benschop R.J. (2022). ImmUniverse Consortium: multi-omics integrative approach in personalized medicine for immune-mediated inflammatory diseases. Front Immunol.

[20] David T., Ling S.F., Barton A. (2018). Genetics of immune-mediated inflammatory diseases. Clin Exp Immunol.

[21] Adami G., Pontalti M., Cattani G. (2022). Association between long-term exposure to air pollution and immune-mediated diseases: a population-based cohort study. RMD Open.

[22] Bach J.F. (2018). The hygiene hypothesis in autoimmunity: the role of pathogens and commensals. Nat Rev Immunol.

[23] Xing Y., Wang M.H., Leung T.F. (2022). Poultry exposure and environmental protection against asthma in rural children. Allergy.

[24] Murdaca G., Greco M., Borro M., Gangemi S. (2021). Hygiene hypothesis and autoimmune diseases: a narrative review of clinical evidences and mechanisms. Autoimmun Rev.

[25] Bellinato F., Adami G., Vaienti S. (2022). Association between short-term exposure to environmental air pollution and psoriasis flare. JAMA Dermatol.

[26] Zhao N., Smargiassi A., Chen H., Widdifield J., Bernatsky S. (2023). Systemic autoimmune rheumatic diseases and multiple industrial air pollutant emissions: a large general population Canadian cohort analysis. Environ Int.

[27] Petrovska N., Prajzlerova K., Vencovsky J., Senolt L., Filkova M. (2021). The pre-clinical phase of rheumatoid arthritis: from risk factors to prevention of arthritis. Autoimmun Rev.

[28] Matarese G. (2023). The link between obesity and autoimmunity. Science.

[29] Frazzei G., Musters A., de Vries N., Tas S.W., van Vollenhoven R.F. (2023). Prevention of rheumatoid arthritis: a systematic literature review of preventive strategies in at-risk individuals. Autoimmun Rev.

[30] Krijbolder D.I., Verstappen M., van Dijk B.T. (2022). Intervention with methotrexate in patients with arthralgia at risk of rheumatoid arthritis to reduce the development of persistent arthritis and its disease burden (TREAT EARLIER): a randomised, double-blind, placebo-controlled, proof-of-concept trial. Lancet.

[31] Rech J., Schett G. (2022). Towards preventive treatment of rheumatoid arthritis. Lancet.

[32] Hensvold A., Klareskog L. (2021). Towards prevention of autoimmune diseases: the example of rheumatoid arthritis. Eur J Immunol.

[33] Reich K., Thyssen J.P., Blauvelt A. (2022). Efficacy and safety of abrocitinib versus dupilumab in adults with moderate-to-severe atopic dermatitis: a randomised, double-blind, multicentre phase 3 trial. Lancet.

[34] Mennini M., Di Nardo G., Fiocchi A.G. (2022). Atopic dermatitis: time for tailored therapy. Lancet.

[35] Thyssen J.P., Schmid-Grendelmeier P. (2022). Long-term disease control in atopic dermatitis using biologics. Lancet.

[36] Tran M.M., Lefebvre D.L., Dharma C. (2018). Predicting the atopic march: results from the Canadian healthy infant longitudinal development study. J Allergy Clin Immunol.

[37] Paller A.S., Spergel J.M., Mina-Osorio P., Irvine A.D. (2019). The atopic march and atopic multimorbidity: many trajectories, many pathways. J Allergy Clin Immunol.

[38] Spergel J.M., Du Toit G., Davis C.M. (2023). Might biologics serve to interrupt the atopic march?. J Allergy Clin Immunol.

[39] Reddel H.K., Bacharier L.B., Bateman E.D. (2022). Global Initiative for Asthma Strategy 2021: executive summary and rationale for key changes. Eur Respir J.

[40] Lommatzsch M., Brusselle G.G., Canonica G.W. (2022). Disease-modifying anti-asthmatic drugs. Lancet.

[41] Pfaar O., Creticos P.S., Kleine-Tebbe J., Canonica G.W., Palomares O., Schulke S. (2021). One hundred ten years of allergen immunotherapy: a Broad look into the future. J Allergy Clin Immunol Pract.

[42] Felson D.T., Smolen J.S., Wells G. (2011). American College of Rheumatology/European League against Rheumatism provisional definition of remission in rheumatoid arthritis for clinical trials. Ann Rheum Dis.

[43] Brusselle G.G., Koppelman G.H. (2022). Biologic therapies for severe asthma. N Engl J Med.

[44] Menzies-Gow A., Corren J., Bourdin A. (2021). Tezepelumab in adults and adolescents with severe, uncontrolled asthma. N Engl J Med.

[45] Konen F.F., Mohn N., Witte T. (2023). Treatment of autoimmunity: the impact of disease-modifying therapies in multiple sclerosis and comorbid autoimmune disorders. Autoimmun Rev.

[46] Bierhansl L., Hartung H.P., Aktas O., Ruck T., Roden M., Meuth S.G. (2022). Thinking outside the box: non-canonical targets in multiple sclerosis. Nat Rev Drug Discov.

[47] Coates L.C., Soriano E.R., Corp N. (2022). Group for research and assessment of psoriasis and psoriatic arthritis (GRAPPA): updated treatment recommendations for psoriatic arthritis 2021. Nat Rev Rheumatol.

[48] Lebwohl M.G., Stein Gold L., Strober B. (2021). Phase 3 trials of tapinarof cream for plaque psoriasis. N Engl J Med.

[49] Lebwohl M.G., Kircik L.H., Moore A.Y. (2022). Effect of roflumilast cream vs vehicle cream on chronic plaque psoriasis: the DERMIS-1 and DERMIS-2 randomized clinical trials. JAMA.

[50] Huang W.W., Feldman S.R. (2021). The next quantum leap forward? Bimekizumab for psoriasis. Lancet.

[51] Queiro R. (2023). Bimekizumab in psoriatic arthritis: a great leap forward?. Lancet.

[52] Zhang B., Domling A. (2022). Small molecule modulators of IL-17A/IL-17RA: a patent review (2013-2021). Expert Opin Ther Pat.

[53] Strober B., Thaci D., Sofen H. (2023). Deucravacitinib versus placebo and apremilast in moderate to severe plaque psoriasis: efficacy and safety results from the 52-week, randomized, double-blinded, phase 3 Program fOr Evaluation of TYK2 inhibitor psoriasis second trial. J Am Acad Dermatol.

[54] McInnes I.B., Anderson J.K., Magrey M. (2021). Trial of upadacitinib and adalimumab for psoriatic arthritis. N Engl J Med.

[55] Baumgart D.C., Le Berre C. (2021). Newer biologic and small-molecule therapies for inflammatory bowel disease. N Engl J Med.

[56] Hibi T. (2022). Risankizumab for Crohn's disease. Lancet.

[57] Najera J., Hao J. (2022). Recent advance in mesenchymal stem cells therapy for atopic dermatitis. J Cell Biochem.

[58] Cheng L., Wang S., Peng C. (2022). Human umbilical cord mesenchymal stem cells for psoriasis: a phase 1/2a, single-arm study. Signal Transduct Target Ther.

[59] Fransson M., Piras E., Burman J. (2012). CAR/FoxP3-engineered T regulatory cells target the CNS and suppress EAE upon intranasal delivery. J Neuroinflammation.

[60] Nash R.A., Hutton G.J., Racke M.K. (2017). High-dose immunosuppressive therapy and autologous HCT for relapsing-remitting MS. Neurology.

[61] Scolding N.J., Pasquini M., Reingold S.C. (2017). Cell-based therapeutic strategies for multiple sclerosis. Brain.

[62] Genchi A., Brambilla E., Sangalli F. (2023). Neural stem cell transplantation in patients with progressive multiple sclerosis: an open-label, phase 1 study. Nat Med.

[63] Bjornevik K., Munz C., Cohen J.I., Ascherio A. (2023). Epstein-Barr virus as a leading cause of multiple sclerosis: mechanisms and implications. Nat Rev Neurol.

[64] Mullard A. (2021). Failure of seres's phase II ulcerative colitis programme renews microbiome concerns. Nat Rev Drug Discov.

[65] Chang J.T. (2020). Pathophysiology of inflammatory bowel diseases. N Engl J Med.

[66] Federici S., Kredo-Russo S., Valdes-Mas R. (2022). Targeted suppression of human IBD-associated gut microbiota commensals by phage consortia for treatment of intestinal inflammation. Cell.

[67] Panes J., Garcia-Olmo D., Van Assche G. (2016). Expanded allogeneic adipose-derived mesenchymal stem cells (Cx601) for complex perianal fistulas in Crohn’s disease: a phase 3 randomised, double-blind controlled trial. Lancet.

[68] Panes J., Garcia-Olmo D., Van Assche G. (2018). Long-term efficacy and safety of stem cell therapy (Cx601) for complex perianal fistulas in patients with crohn’s disease. Gastroenterology.

[69] Migliori G.B., Thong P.M., Akkerman O. (2020). Worldwide effects of coronavirus disease pandemic on tuberculosis Services, january-april 2020. Emerg Infect Dis.

[70] Ting D.S.W., Carin L., Dzau V., Wong T.Y. (2020). Digital technology and COVID-19. Nat Med.

[71] Jena A., Mishra S., Deepak P. (2022). Response to SARS-CoV-2 vaccination in immune mediated inflammatory diseases: systematic review and meta-analysis. Autoimmun Rev.

[72] Simon D., Tascilar K., Fagni F. (2022). Efficacy and safety of SARS-CoV-2 revaccination in non-responders with immune-mediated inflammatory disease. Ann Rheum Dis.

[73] Izadi Z., Brenner E.J., Mahil S.K. (2021). Association between tumor necrosis factor inhibitors and the risk of hospitalization or death among patients with immune-mediated inflammatory disease and COVID-19. JAMA Netw Open.

[74] Veenstra J., Buechler C.R., Robinson G. (2020). Antecedent immunosuppressive therapy for immune-mediated inflammatory diseases in the setting of a COVID-19 outbreak. J Am Acad Dermatol.

[75] Mwatondo A., Rahman-Shepherd A., Hollmann L. (2023). A global analysis of one health networks and the proliferation of One health collaborations. Lancet.

[76] Zinsstag J., Kaiser-Grolimund A., Heitz-Tokpa K. (2023). Advancing One human-animal-environment Health for global health security: what does the evidence say?. Lancet.

