# Supplementary material for: Global, regional, and national incidence of six major immune-mediated inflammatory diseases: findings from the global burden of disease study 2019
Source: eClinicalMedicine. 2023 Sep 9;64:102193. doi: 10.1016/j.eclinm.2023.102193 (PMC10507198; doi:10.1016/j.eclinm.2023.102193)
Supplement: Appendix [file mmc1.pdf]

Appendix

Table of content

Authors' contributions

Part 1. Providing data or critical feedback on data sources

Part 2. Developing methods or computational machinery

Part 3. Providing critical feedback on methods or results

Part 4. Drafting the work or revising it critically for important intellectual content

Part 5. Managing the estimation or publications process

Supplementary figures

sFigure 1. The number of incident cases attributable to six immune mediated inflammatory diseases throughout human lifespan in 2019

sFigure 2. The rate of incidence attributable to six immune mediated inflammatory diseases throughout human lifespan in 2019

sFigure 3. The average annual percent change in number of incidents attributable to six immune mediated inflammatory diseases throughout human lifespan, 1990-2019

sFigure 4. The average annual percent change in rate of incidence attributable to six immune mediated inflammatory diseases throughout human lifespan, 1990-2019

sFigure 5. The incident cases and age-standardized rate of incidence attributable to overall immune mediated inflammatory diseases according to GBD regions in 2019

sFigure 6. The incident cases and age-standardized rate of incidence attributable to overall immune mediated inflammatory diseases according to gender in 2019

sFigure 7. The incident cases and age-standardized rate of incidence attributable to six immune mediated inflammatory diseases according to GBD regions in 2019

sFigure 8. The average annual percent change in number of incidents and age standardized rate of incidence attributable to six immune mediated inflammatory diseases across 21 GBD regions, 1990-2019

sFigure 9. The global map in number of incidents attributable to six immune mediated inflammatory diseases in 204 countries and territories, 2019

sFigure 10. The global map in age standardized rate of incidence attributable to six immune mediated inflammatory diseases in 204 countries and territories, 2019

sFigure 11. The global map in average annual percent change in number of incidence attributable to six immune mediated inflammatory diseases in 204 countries and territories, 1990-2019

sFigure 12. The global map in average annual percent change in age standardized rate of incidence attributable to six immune mediated inflammatory diseases in 204 countries and territories, 1990-2019

sFigure 13. Age standardized rate of incidence attributable to six immune mediated inflammatory diseases per 100 000 persons for sociodemographic index by 21 GBD regions, 2019

sFigure 14. Age standardized rate of incidence attributable to six immune mediated inflammatory diseases per 100 000 persons for sociodemographic index by 204 countries and territories, 2019

sFigure 15. The correlation between average annual percent change and age standardized rate of incidence attributable to six immune mediated inflammatory in 1990 across 204 countries and territories

sFigure 16. The correlation between average annual percent change and socio-demographic index attributable to six immune mediated inflammatory in 2019 across 204 countries and territories

Supplementary table

sTable 1. The incident cases and rate of incidence attributable to overall immune mediated inflammatory diseases across 20 age groups, and its temporal trends from 1990 to 2019

sTable 2. The incident cases and rate of incidence attributable to six immune mediated inflammatory diseases across 20 age groups, and its temporal trends from 1990 to 2019

sTable 3. The incident cases and age-standardized rate of incidence attributable to overall immune mediated inflammatory diseases according to 204 countries and territories and its temporal trends from 1990 to 2019

sTable 4. The incident cases and age-standardized rate of incidence attributable to six immune mediated inflammatory diseases according to SDI regions and its temporal trends from 1990 to 2019.

sTable 5. The incident cases and age-standardized rate of incidence attributable to six immune mediated inflammatory diseases according to GBD regions and its temporal trends from 1990 to 2019.

sTable 6. The incident cases and age-standardized rate of incidence attributable to six immune mediated inflammatory diseases according to countries and territories and its temporal trends from 1990 to 2019.

Authors' contributions

Part 1. Providing data or critical feedback on data sources

Melsew Dagne Abate, Mohammadreza Abbasian, Aidin Abedi, Hassan Abidi, Richard Gyan Aboagye, Hassan Abolhassani, Qorinah Estiningtyas Sakilah Adnani, Mohsen Afarideh, Danish Ahmad, Sajjad Ahmad, Sohail Ahmad, Ali Ahmadi, Ayman Ahmed, Janardhana P Aithala, Abdullateef Abiodun Ajadi, Fares Alahdab, Mohammad T AlBataineh, Liaqat Ali, Sheikh Mohammad Alif, Joseph Uy Almazan, Sami Almustanyir, Jaber S Alqahtani, Ihsan Ullah Khan Altaf, Nelson Alvis-Guzman, Nelson J Alvis-Zakzuk, Hany Aly, Hubert Amu, Ganiyu Adeniyi Amusa, Jalal Arabloo, Reza Arefnezhad, Judie Arulappan, Tahira Ashraf, Jamila Abdulhamid Atata, Seyyed Shamsadin Athari, Maha Moh'd Wahbi Atout, Ahmed Y Azzam, Alaa Badawi, Ashish D Badiye, Sara Bagherieh, Atif Amin Baig, Mainak Bardhan, Nebiyou Simegnew Bayileyegn, Uzma Iqbal Belgaumi, Akshaya Srikanth Bhagavathula, Sonu Bhaskar, Ajay Nagesh Bhat, Archith Boloor, João Silva Botelho, Luis Alberto Cámera, Vijay Kumar Chattu, Dinh-Toi Chu, Rosa A S Couto, Natália Cruz-Martins, Xiaochen Dai, Giovanni Damiani, Ana Maria Dascalu, Andreas K Demetriades, Hardik Dineshbhai Desai, Rupak Desai, Sourav Dey, Samath Dhamminda Dharmaratne, Thanh Chi Do, Masoud Dodangeh, Milad Dodangeh, Michael Ekholuenetale, Temitope Cyrus Ekundayo, Ahmed O Elmehrath, Hawi Leul Esayas, Adeniyi Francis Fagbamigbe, Ildar Ravisovich Fakhradiyev, Ali Fatehizadeh, Alireza Feizkhah, Ginenus Fekadu, Seyed-Mohammad Fereshtehnejad, João C Fernandes, Pietro Ferrara, Takeshi Fukumoto, Maryam Gholamalizadeh, Mahaveer Golechha, Pouya Goleij, Vivek Kumar Gupta, Rabih Halwani, Shafiul Haque, Ikramul Hasan, Reza Hashempour, Treska S Hassan, Johannes Haubold, Reza Heidari-Soureshjani, Claudiu Herteliu, Kamran Hessami, Hong-Han Huynh, Segun Emmanuel Ibitoye, Farideh Iravanpour, Nahlah Elkudssiah Ismail, Morteza Jafarinia, Haitham Jahrami, Mihajlo Jakovljevic, Manthan Dilipkumar Janodia, Sathish Kumar Jayapal, Shubha Jayaram, Jost B Jonas, Abel Joseph, Nitin Joseph, Charity Ehimwenma Joshua, Billingsley Kaambwa, Zubair Kabir, Vidya Kadashetti, Feroze Kaliyadan, Himal Kandel, Ibraheem M Karaye, Yousef Saleh Khader, Himanshu Khajuria, Faham Khamesipour, Imteyaz A Khan, Maseer Khan, Moien AB Khan, Muhammad Arslan Khan, Sorour Khateri, Adnan Kisa, Sezer Kisa, Hyun Yong Koh, Pavel Kolkhir, Oleksii Korzh, Chandrakant Lahariya, Tri Laksono, Kamaluddin Latief, Basira Kankia Lawal, Thao Thi Thu Le, Seung Won Lee, Yo Han Lee, Wei Li, Virendra S Ligade, Stephen S Lim, Gang Liu, Xuefeng Liu, Vanessa Sintra Machado, Azzam A Maghazachi, Tuan A Mai, Pantea Majma Sanaye, Kashish Malhotra, Deborah Carvalho Malta, Mohammad Ali Mansournia, Ritesh G Menezes, Irmina Maria Michalek, Le Huu Nhat Minh, Erkin M Mirrakhimov, Awoke Misganaw, Soheil Mohammadi, Salahuddin Mohammed, Shafiu Mohammed, Ali H Mokdad, Sara Momtazmanesh, Mohammad Ali Moni, Yousef Moradi, Ebrahim Mostafavi, George Duke Mukoro, Admir Mulita, Getaneh Baye Mulu, Efrén Murillo-Zamora, Ghulam Mustafa, Sreenivas Narasimha Swamy, Zuhair S Natto, Biswa Prakash Nayak, Dang H Nguyen, Hien Quang Nguyen, Phat Tuan Nguyen, Van Thanh Nguyen, Robina Khan Niazi, Taxiarchis Konstantinos Nikolouzakis, Lawrence Achilles Nnyanzi, Bogdan Oancea, Osaretin Christabel Okonji, Patrick Godwin Okwute, Andrew T Olagunju, Michal Ordak, Mayowa O Owolabi, Mahesh P A, Adrian Pana, Shahina Pardhan, Jay Patel, Shankargouda Patil, Uttam Paudel, Shrikant Pawar, Paolo Pedersini, David M Pereira, Jeevan Pereira, Renato B Pereira, Mario F P Peres, Arokiasamy Perianayagam, Simone Perna, Hoang Tran Pham, Anil K Philip, Vivek Podder, Elton Junio Sady Prates, Fakher Rahim, Mehran Rahimi, Vafa Rahimi-Movaghar, Amir Masoud Rahmani, Shayan Rahmani, Nemanja Rancic, Sina Rashedi, Ahmed Mustafa Rashid, Salman Rawaf, Elrashdy Moustafa Mohamed Redwan, Nima Rezaei, Jefferson Antonio Buendia Rodriguez, Leonardo Roever, Esperanza Romero-Rodríguez, Aly M A Saad, Basema Saddik, Umar Saeed, Narjes Saheb Sharif-Askari, Mohammad Ali Sahraian, Mirza Rizwan Sajid, Sateesh Sakhamuri, Mohamed A Saleh, Sara Samadzadeh, Abdallah M Samy, Monika Sawhney, Yashendra Sethi, Samiah Shahid, Masood Ali Shaikh, Maryam Shayan, Jae Il Shin, K M Shivakumar, Parnian Shobeiri, Soraya Siabani, Inderbir Singh, Jasvinder A Singh, Paramdeep Singh, Solikhah Solikhah, Yonatan Solomon, Mohammad Sadegh Soltani-Zangbar, Jing Sun, Mindy D Szeto, Rafael Tabarés-Seisdedos, Mohammad Tabish, Lai-shan Tam, Ker-Kan Tan, Mohamad-Hani Temsah, Pugazhenthan Thangaraju, Nigusie Selomon Tibebu, Jansje Henny Vera Ticoalu, Tala Tillawi, Amir Tiyuri, Marcos Roberto Tovani-Palone, Manjari Tripathi, Guesh Mebrahtom Tsegay, Abdul Rohim Tualeka, Saif Ullah, Sana Ullah, Muhammad Umair, Srikanth Umakanthan, Era Upadhyay, Sahel Valadan Tahbaz, Shoban Babu Varthya, Tommi Juhani Vasankari, Narayanaswamy Venketasubramanian, Georgios-Ioannis Verras, Vasily Vlassov, Yasir Waheed, Ronny Westerman, Dakshitha Praneeth Wickramasinghe, Beshada Zerfu Woldegeorgis, Dongze Wu, Dereje Y Yada, Galal Yahya,

Dong Keon Yon, Naohiro Yonemoto, Iman Zare, Mohammad A Zeineddine, Naod Gebrekrstos Zeru, Magdalena Zielińska, Mohammad Zoladl.

Part 2. Developing methods or computational machinery

Qorinah Estiningtyas Sakilah Adnani, Ali Ahmadi, Ibrahim Alqasmi, Hubert Amu, Ahmed Y Azzam, Akshaya Srikanth Bhagavathula, Dinh-Toi Chu, Kaleb Coberly, Rosa A S Couto, Xiaochen Dai, Hardik Dineshbhai Desai, Thanh Chi Do, Ali Fatehizadeh, Mohammad Heidari, Hong-Han Huynh, Farideh Iravanpour, Morteza Jafarinia, Sathish Kumar Jayapal, Leila Keikavoosi-Arani, Sorour Khateri, Adnan Kisa, Thao Thi Thu Le, Tuan A Mai, Seyedeh Zahra Masoumi, Le Huu Nhat Minh, Ali H Mokdad, Mohammad Ali Moni, Yousef Moradi, Admir Mulita, Dang H Nguyen, Hien Quang Nguyen, Phat Tuan Nguyen, Van Thanh Nguyen, Hoang Tran Pham, Amir Masoud Rahmani, Premkumar Ramasubramani, Umar Saeed, Abdallah M Samy, Ganesh Kumar Saya, Razieh Tavakoli Oliaee, Ronny Westerman, Melat Woldemariam, Dongze Wu, Naohiro Yonemoto.

Part 3. Providing critical feedback on methods or results

Melsew Dagne Abate, Mohammadreza Abbasian, Michael Abdelmasseh, Mohammad-Amin Abdollahifar, Deldar Morad Abdulah, Aidin Abedi, Vida Abedi, Hassan Abidi, Richard Gyan Aboagye, Hassan Abolhassani, Katrina Abuabara, Isaac Yeboah Addo, Kayode Nelson Adeniji, Abiola Victor Adepoju, Miracle Ayomikun Adesina, Qorinah Estiningtyas Sakilah Adnani, Mohsen Afarideh, Shahin Aghamiri, Antonella Agodi, Anurag Agrawal, Constanza Elizabeth Aguilera Arriagada, Aqeel Ahmad, Danish Ahmad, Sajjad Ahmad, Sohail Ahmad, Ali Ahmadi, Ali Ahmed, Ayman Ahmed, Janardhana P Aithala, Abdullateef Abiodun Ajadi, Mostafa Akbarzadeh-Khiavi, Fares Alahdab, Mohammad T AlBataineh, Sharifullah Alemi, Adel Ali Saeed Al-Gheethi, Liaqat Ali, Sheikh Mohammad Alif, Sami Almustanyir, Jaber S Alqahtani, Ihsan Ullah Khan Altaf, Nelson Alvis-Guzman, Nelson J Alvis-Zakzuk, Hany Aly, Reza Amani, Hubert Amu, Ganiyu Adeniyi Amusa, Catalina Liliana Andrei, Anayochukwu Edward Anyasodor, Jalal Arabloo, Judie Arulappan, Mohammad Asghari-Jafarabadi, Tahira Ashraf, Jamila Abdulhamid Atata, Seyyed Shamsadin Athari, Daniel Atlaw, Maha Moh'd Wahbi Atout, Avinash Aujayeb, Asma Tahir Awan, Haleh Ayatollahi, Sina Azadnajafabad, Ahmed Y Azzam, Alaa Badawi, Ashish D Badiye, Sara Bagherieh, Atif Amin Baig, Berihun Bantie Bantie, Martina Barchitta, Mainak Bardhan, Suzanne Lyn Barker-Collo, Kavita Batra, Nebiyou Simegnew Bayileyegn, Amir Hossein Behnoush, Uzma Iqbal Belgaumi, Maryam Bemanalizadeh, Isabela M Bensenor, Akshaya Srikanth Bhagavathula, Pankaj Bhardwaj, Sonu Bhaskar, Ajay Nagesh Bhat, Veera R Bitra, Archith Boloor, Kaustubh Bora, João Silva Botelho, Rachelle Buchbinder, Luis Alberto Cámera, Andre F Carvalho, Vijay Kumar Chattu, Endeshaw Chekol Abebe, Fatemeh Chichagi, Sungchul Choi, Tzu-Chieh Chou, Dinh-Toi Chu, Vera Marisa Costa, Rosa A S Couto, Natália Cruz-Martins, Omid Dadras, Xiaochen Dai, Giovanni Damiani, Ana Maria Dascalu, Mohsen Dashti, Sisay Abebe Debela, Andreas K Demetriades, Alemayehu Anley Demlash, Xinlei Deng, Hardik Dineshbhai Desai, Rupak Desai, Sourav Dey, Samath Dhamminda Dharmaratne, Daniel Diaz, Mahmoud Dibas, Ricardo Jorge Dinis-Oliveira, Mengistie Diress, Thanh Chi Do, Duy Khanh Doan, Masoud Dodangeh, Milad Dodangeh, Deepa Dongarwar, Arkadiusz Marian Dziedzic, Abdelaziz Ed-Dra, Hisham Atan Edinur, Negin Eissazade, Michael Ekholuenetale, Temitope Cyrus Ekundayo, Noha Mousaad Elemam, Muhammed Elhadi, Ahmed O Elmehrath, Omar Abdelsadek Abdou Elmeligy, Mehdi Emamverdi, Hawi Leul Esayas, Habitu Birhan Eshetu, Farshid Etaee, Adeniyi Francis Fagbamigbe, Shahriar Faghani, Ildar Ravisovich Fakhradiyev, Ali Fatehizadeh, Mobina Fathi, Alireza Feizkhah, Mohammad Fereidouni, Seyed-Mohammad Fereshtehnejad, João C Fernandes, Pietro Ferrara, Getahun Fetensa, Irina Filip, Florian Fischer, Behzad Foroutan, Masoud Foroutan, Takeshi Fukumoto, Balasankar Ganesan, Belete Negese Belete Gemeda, MohammadReza Ghasemi, Mohamad Goldust, Mahaveer Golechha, Davide Golinelli, Houman Goudarzi, Shi-Yang Guan, Cui Guo, Bhawna Gupta, Vivek Kumar Gupta, Rasool Haddadi, Najah R Hadi, Rabih Halwani, Shafiul Haque, Ikramul Hasan, Reza Hashempour, Treska S Hassan, Sara Hassanzadeh, Mohammed Bheser Hassen, Johannes Haubold, Khezar Hayat, Golnaz Heidari, Mohammad Heidari, Reza Heidari-Soureshjani, Claudiu Herteliu, Kamran Hessami, Kamal Hezam, Yuta Hiraike, Ramesh Holla, Mohammad-Salar Hosseini, Hong-Han Huynh, Bing-Fang Hwang, Segun Emmanuel Ibitoye, Irena M Ilic, Milena D Ilic, Farideh Iravanpour, Nahlah Elkudssiah Ismail, Masao Iwagami, Chidozie C D Iwu, Louis Jacob, Morteza Jafarinia, Abdollah Jafarzadeh, Haitham Jahrami, Mihajlo Jakovljevic, Elham Jamshidi, Chinmay T Jani, Sathish Kumar Jayapal, Shubha Jayaram, Jayakumar Jeganathan, Jost B Jonas, Abel Joseph, Nitin Joseph, Charity Ehimwenma Joshua, Vaishali K, Billingsley Kaambwa, Ali Kabir, Zubair Kabir, Vidya Kadashetti, Feroze Kaliyadan, fatemeh kalroozi, Vineet Kumar Kamal, Himal

Kandel, Srikanta Kanungo, Jafar Karami, Ibraheem M Karaye, Hanie Karimi, Sina Kazemian, Sewnet Adem Kebede, Leila Keikavoosi-Arani, Mohammad Keykhaei, Yousef Saleh Khader, Himanshu Khajuria, Faham Khamesipour, Ejaz Ahmad Khan, Imteyaz A Khan, Maseer Khan, Md Jobair Khan, Moien AB Khan, Muhammad Arslan Khan, Haitham Khatatbeh, Moawiah Mohammad Khatatbeh, Sorour Khateri, Min Seo Kim, Adnan Kisa, Sezer Kisa, Hyun Yong Koh, Pavel Kolkhir, Oleksii Korzh, Ashwin Laxmikant Kotnis, Ai Koyanagi, Mohammed Kuddus, Narinder Kumar, Satyajit Kundu, Om P Kurmi, Carlo La Vecchia, Chandrakant Lahariya, Tri Laksono, Judit Lám, Kamaluddin Latief, Paolo Lauriola, Basira Kankia Lawal, Thao Thi Thu Le, Seung Won Lee, Wei-Chen Lee, Yo Han Lee, Jacopo Lenzi, Wei Li, Virendra S Ligade, Stephen S Lim, Gang Liu, Xuefeng Liu, Erand Llanaj, Chun-Han Lo, Vanessa Sintra Machado, Azzam A Maghazachi, Mansour Adam Mahmoud, Tuan A Mai, Azeem Majeed, Omar Mohamed Makram, Elaheh Malakan Rad, Kashish Malhotra, Ahmad Azam Malik, Iram Malik, Tauqeer Hussain Mallhi, Deborah Carvalho Malta, Mohammad Ali Mansournia, Lorenzo Giovanni Mantovani, Miquel Martorell, Sahar Masoudi, Yasith Mathangasinghe, Elezebeth Mathews, Alexander G Mathioudakis, Andrea Maugeri, Mahsa Mayeli, John Robert Carabeo Medina, Gebrekiros Gebremichael Meles, Ritesh G Menezes, Tomislav Mestrovic, Irmina Maria Michalek, Ana Carolina Micheletti Gomide Nogueira de Sá, Ephrem Tesfaye Mihretie, Le Huu Nhat Minh, Reza Mirfakhraie, Erkin M Mirrakhimov, Awoke Misganaw, Ashraf Mohamadkhani, Nouh Saad Mohamed, Faezeh Mohammadi, Soheil Mohammadi, Salahuddin Mohammed, Shafiu Mohammed, Syam Mohan, Anita Mohseni, Ali H Mokdad, Sara Momtazmanesh, Mohammad Ali Moni, Md Moniruzzaman, Yousef Moradi, Negar Morovatdar, Ebrahim Mostafavi, George Duke Mukoro, Admir Mulita, Getaneh Baye Mulu, Efrén Murillo-Zamora, Fungai Musaigwa, Ghulam Mustafa, Sathish Muthu, Firzan Nainu, Vinay Nangia, Sreenivas Narasimha Swamy, Zuhair S Natto, Navaraj Perumalsamy, Biswa Prakash Nayak, Athare Nazri-Panjaki, Hadush Negash, Dang H Nguyen, Hau Thi Hien Nguyen, Hien Quang Nguyen, Phat Tuan Nguyen, Van Thanh Nguyen, Robina Khan Niazi, Taxiarchis Konstantinos Nikolouzakis, Lawrence Achilles Nnyanzi, Mamoona Noreen, Chimezie Igwegbe Nzoputam, Ogochukwu Janet Nzoputam, Bogdan Oancea, Hassan Okati-Aliabad, Osaretin Christabel Okonji, Patrick Godwin Okwute, Andrew T Olagunju, Matthew Idowu Olatubi, Isaac Iyinoluwa Olufadewa, Michal Ordak, Nikita Otstavnov, Mayowa O Owolabi, Mahesh P A, Jagadish Rao Padubidri, Anton Pak, Reza Pakzad, Raffaele Palladino, Adrian Pana, Ioannis Pantazopoulos, Paraskevi Papadopoulou, Shahina Pardhan, Ashwaghosha Parthasarathi, Ava Pashaei, Jay Patel, Aslam Ramjan Pathan, Shankargouda Patil, Uttam Paudel, Shrikant Pawar, Paolo Pedersini, Umberto Pensato, David M Pereira, Jeevan Pereira, Maria Odete Pereira, Renato B Pereira, Mario F P Peres, Arokiasamy Perianayagam, Ionela-Roxana Petcu, Parmida Sadat Pezeshki, Hoang Tran Pham, Anil K Philip, Michael A Piradov, Indrashis Podder, Vivek Podder, Elton Junio Sady Prates, Ibrahim Qattea, Amir Radfar, Pourya Raee, Alireza Rafiei, Fakher Rahim, Mehran Rahimi, Vafa Rahimi-Movaghar, Md Obaidur Rahman, Mosiur Rahman, Muhammad Aziz Rahman, Amir Masoud Rahmani, Mohamed Rahmani, Vahid Rahmanian, Premkumar Ramasubramani, Nemanja Rancic, Sina Rashedi, Ahmed Mustafa Rashid, Nakul Ravikumar, Salman Rawaf, Elrashdy Moustafa Mohamed Redwan, Nazila Rezaei, Negar Rezaei, Mohsen Rezaeian, Daniela Ribeiro, Mónica Rodrigues, Jefferson Antonio Buendia Rodriguez, Leonardo Roever, Esperanza Romero-Rodríguez, Aly M A Saad, Basema Saddik, Saeid Sadeghian, Umar Saeed, Azam Safary, Mahdi Safdarian, Sher Zaman Safi, Amene Saghazadeh, Fatemeh Saheb Sharif-Askari, Narjes Saheb Sharif-Askari, Harihar Sahoo, Mohammad Ali Sahraian, Mirza Rizwan Sajid, Sateesh Sakhamuri, Joseph W Sakshaug, Mohamed A Saleh, Sana Salehi, Amir Salek Farrokhi, Sara Samadzadeh, Noosha Samieefar, Abdallah M Samy, Nima Sanadgol, Rama Krishna Sanjeev, Monika Sawhney, Ganesh Kumar Saya, Art Schuermans, Subramanian Senthilkumaran, Sadaf G Sepanlou, Yashendra Sethi, Mahan Shafie, Humaira Shah, Izza Shahid, Samiah Shahid, Masood Ali Shaikh, Sadaf Sharfaei, Maryam Shayan, Hatem Samir Shehata, Aziz Sheikh, Jeevan K Shetty, Jae Il Shin, Reza Shirkoohi, Nebiyu Aniley Shitaye, K M Shivakumar, Parnian Shobeiri, Soraya Siabani, Migbar Mekonnen Sibhat, Emmanuel Edwar Siddig, Ehsan Sinaei, Inderbir Singh, Jasvinder A Singh, Paramdeep Singh, Surjit Singh, Md Shahjahan Siraj, Abdullah Al Mamun Sohag, Yonatan Solomon, Mohammad Sadegh Soltani-Zangbar, Jing Sun, Mindy D Szeto, Rafael Tabarés-Seisdedos, Seyyed Mohammad Tabatabaei, Mohammad Tabish, Ensiyeh Taheri, Azin Tahvildari, Iman M Talaat, Lai-shan Tam, Jacques JL Lukenze Tamuzi, Ker-Kan Tan, Razieh Tavakoli Oliaee, Arian Tavasol, Mohamad-Hani Temsah, Pugazhenthan Thangaraju, Samar Tharwat, Jansje Henny Vera Ticoalu, Tala Tillawi, Tenaw Yimer Tiruye, Amir Tiyuri, Marcos Roberto Tovani-Palone, Manjari Tripathi, Guesh Mebrahtom Tsegay, Sree Sudha Ty, Chukwudi S Ubah, Saif Ullah, Sana Ullah, Srikanth Umakanthan, Era Upadhyay, Seyed Mohammad Vahabi, Asokan Govindaraj Vaithinathan, Sahel

Valadan Tahbaz, Rohollah Valizadeh, Shoban Babu Varthya, Narayanaswamy Venketasubramanian, Georgios-Ioannis Verras, Jorge Hugo Villafañe, Vasily Vlassov, Danh Cao Vo, Yasir Waheed, Abdul Waris, Brhane Gebrehiwot Welegebrial, Ronny Westerman, Dakshitha Praneeth Wickramasinghe, Nuwan Darshana Wickramasinghe, Barbara Willekens, Beshada Zerfu Woldegeorgis, Dongze Wu, Yuhan Xing, Galal Yahya, Lin Yang, Fereshteh Yazdanpanah, Dong Keon Yon, Naohiro Yonemoto, Yuyi You, Mazyar Zahir, Syed Saoud Zaidi, Moein Zangiabadian, Mohammad A Zeineddine, Dawit T Zemedikun, Naod Gebrekrstos Zeru, Chen Zhang, Hanqing Zhao, Chenwen Zhong, Magdalena Zielińska, Mohammad Zoladl, Alimuddin Zumla.

Part 4. Drafting the work or revising it critically for important intellectual content

Melsew Dagne Abate, Mohammadreza Abbasian, Mohsen Abbasi-Kangevari, Zeinab Abbasi-Kangevari, Foad Abd-Allah, Michael Abdelmasseh, Aidin Abedi, Hassan Abidi, Hassan Abolhassani, Katrina Abuabara, Morteza Abyadeh, Isaac Yeboah Addo, Kayode Nelson Adeniji, Abiola Victor Adepoju, Qorinah Estiningtyas Sakilah Adnani, Antonella Agodi, Constanza Elizabeth Aguilera Arriagada, Danish Ahmad, Ali Ahmadi, Ali Ahmadi, Ali Ahmed, Ali Ahmed, Ayman Ahmed, Janardhana P Aithala, Abdullateef Abiodun Ajadi, Marjan Ajami, Fares Alahdab, Mohammad T AlBataineh, Liaqat Ali, Sami Almustanyir, Jaber S Alqahtani, Ihsan Ullah Khan Altaf, Nelson Alvis-Guzman, Nelson J Alvis-Zakzuk, Yaser Mohammed Al-Worafi, Hany Aly, Reza Amani, Hubert Amu, Ganiyu Adeniyi Amusa, Catalina Liliana Andrei, Adnan Ansar, Hossein Ansariniya, Anayochukwu Edward Anyasodor, Jalal Arabloo, Judie Arulappan, Jamila Abdulhamid Atata, Seyyed Shamsadin Athari, Daniel Atlaw, Maha Moh'd Wahbi Atout, Avinash Aujayeb, Sina Azadnajafabad, Ahmed Y Azzam, Alaa Badawi, Ashish D Badiye, Sara Bagherieh, Atif Amin Baig, Martina Barchitta, Mainak Bardhan, Suzanne Lyn Barker-Collo, Francesco Barone-Adesi, Kavita Batra, Amir Hossein Behnoush, Uzma Iqbal Belgaumi, Maryam Bemanalizadeh, Isabela M Bensenor, Kebede A Beyene, Akshaya Srikanth Bhagavathula, Sonu Bhaskar, Ajay Nagesh Bhat, Saeid Bitaraf, Veera R Bitra, Kaustubh Bora, João Silva Botelho, Daniela Calina, Luis Alberto Cámera, Andre F Carvalho, Jeffrey Shi Kai Chan, Vijay Kumar Chattu, Fatemeh Chichagi, Sungchul Choi, Dinh-Toi Chu, Vera Marisa Costa, Rosa A S Couto, Natália Cruz-Martins, Giovanni Damiani, Robert Paul Dellavalle, Andreas K Demetriades, Hardik Dineshbhai Desai, Syed Masudur Rahman Dewan, Sourav Dey, Samath Dhamminda Dharmaratne, Daniel Diaz, Mahmoud Dibas, Ricardo Jorge Dinis-Oliveira, Thanh Chi Do, Masoud Dodangeh, Milad Dodangeh, Deepa Dongarwar, John Dube, Arkadiusz Marian Dziedzic, Negin Eissazade, Noha Mousaad Elemam, Muhammed Elhadi, Ahmed O Elmehrath, Omar Abdelsadek Abdou Elmeligy, Mehdi Emamverdi, Theophilus I Emeto, Habitu Birhan Eshetu, Farshid Etaee, Adeniyi Francis Fagbamigbe, Shahriar Faghani, Ali Fatehizadeh, Mobina Fathi, Mohammad Fereidouni, Seyed-Mohammad Fereshtehnejad, João C Fernandes, Pietro Ferrara, Getahun Fetensa, Irina Filip, Florian Fischer, Masoud Foroutan, Takeshi Fukumoto, Balasankar Ganesan, Seyyed-Hadi Ghamari, MohammadReza Ghasemi, Tiffany K Gill, Richard F Gillum, Mohamad Goldust, Davide Golinelli, Shi-Yang Guan, Cui Guo, Bhawna Gupta, Veer Bala Gupta, Vivek Kumar Gupta, Najah Hadi, Najah R Hadi, Rabih Halwani, Shafiul Haque, Ikramul Hasan, Amr Hassan, Treska S Hassan, Sara Hassanzadeh, Johannes Haubold, Khezar Hayat, Golnaz Heidari, Claudiu Herteliu, Kamran Hessami, Kamal Hezam, Yuta Hiraike, Ramesh Holla, Mohammad-Salar Hosseini, Hong-Han Huynh, Segun Emmanuel Ibitoye, Irena M Ilic, Milena D Ilic, Arad Iranmehr, Farideh Iravanpour, Nahlah Elkudssiah Ismail, Chidozie C D Iwu, Louis Jacob, Morteza Jafarinia, Abdollah Jafarzadeh, Kasra Jahankhani, Haitham Jahrami, Mihajlo Jakovljevic, Chinmay T Jani, Manthan Dilipkumar Janodia, Sathish Kumar Jayapal, Shubha Jayaram, Yingzhao Jin, Jost B Jonas, Abel Joseph, Nitin Joseph, Ali Kabir, Vidya Kadashetti, Feroze Kaliyadan, fatemeh kalroozi, Amit Kandel, Himal Kandel, Jafar Karami, Hanie Karimi, Hengameh Kasraei, Leila Keikavoosi-Arani, Yousef Saleh Khader, Himanshu Khajuria, Ejaz Ahmad Khan, Imteyaz A Khan, Maseer Khan, Md Jobair Khan, Moien Khan, Moien AB Khan, Muhammad Arslan Khan, Haitham Khatatbeh, Moawiah Mohammad Khatatbeh, Sorour Khateri, Hamid Reza Khayat Kashani, Min Seo Kim, Adnan Kisa, Sezer Kisa, Hyun Yong Koh, Pavel Kolkhir, Oleksii Korzh, Ashwin Laxmikant Kotnis, Parvaiz A Koul, Ai Koyanagi, Kewal Krishan, Mohammed Kuddus, Vishnutheertha Vishnutheertha Kulkarni, Narinder Kumar, Om P Kurmi, Carlo La Vecchia, Chandrakant Lahariya, Judit Lám, Kamaluddin Latief, Paolo Lauriola, Basira Kankia Lawal, Thao Thi Thu Le, Trang Thi Bich Le, Munjae Lee, Seung Won Lee, Jacopo Lenzi, Miriam Levi, Erand Llanaj, Chun-Han Lo, Vanessa Sintra Machado, Mansour Adam Mahmoud, Tuan A Mai, Azeem Majeed, Omar Mohamed Makram, Elaheh Malakan Rad, Ahmad Azam Malik, Iram Malik, Tauqeer Hussain Mallhi, Deborah Carvalho Malta,

Lorenzo Giovanni Mantovani, Miquel Martorell, Yasith Mathangasinghe, Elezebeth Mathews, Alexander G Mathioudakis, Andrea Maugeri, Mahsa Mayeli, Gebrekiros Gebremichael Meles, José João Mendes, Ritesh G Menezes, Tomislav Mestrovic, Irmina Maria Michalek, Ana Carolina Micheletti Gomide Nogueira de Sá, Ephrem Tesfaye Mihretie, Le Huu Nhat Minh, Reza Mirfakhraie, Awoke Misganaw, Faezeh Mohammadi, Salahuddin Mohammed, Shafiu Mohammed, Ali H Mokdad, Sara Momtazmanesh, Lorenzo Monasta, Mohammad Ali Moni, Negar Morovatdar, Ebrahim Mostafavi, Parsa Mousavi, George Duke Mukoro, Fungai Musaigwa, Sathish Muthu, Firzan Nainu, Sreenivas Narasimha Swamy, Zuhair S Natto, Biswa Prakash Nayak, Hadush Negash, Mohammad Hadi Nematollahi, Dang H Nguyen, Hau Thi Hien Nguyen, Hien Quang Nguyen, Phat Tuan Nguyen, Van Thanh Nguyen, Robina Khan Niazi, Taxiarchis Konstantinos Nikolouzakis, Lawrence Achilles Nnyanzi, Mamoona Noreen, Chimezie Igwegbe Nzoputam, Ogochukwu Janet Nzoputam, Bogdan Oancea, In-Hwan Oh, Osaretin Christabel Okonji, Patrick Godwin Okwute, Andrew T Olagunju, Matthew Idowu Olatubi, Michal Ordak, Nikita Otstavnov, Mayowa O Owolabi, Mahesh P A, Jagadish Rao Padubidri, Anton Pak, Reza Pakzad, Raffaele Palladino, Adrian Pana, Ioannis Pantazopoulos, Paraskevi Papadopoulou, Shahina Pardhan, Ashwaghosha Parthasarathi, Ava Pashaei, Jay Patel, Shankargouda Patil, Uttam Paudel, Shrikant Pawar, Paolo Pedersini, Umberto Pensato, David M Pereira, Jeevan Pereira, Maria Odete Pereira, Renato B Pereira, Mario F P Peres, Arokiasamy Perianayagam, Ionela-Roxana Petcu, Parmida Sadat Pezeshki, Hoang Tran Pham, Anil K Philip, Indrashis Podder, Vivek Podder, Dimitri Poddighe, Elton Junio Sady Prates, Amir Radfar, Alireza Rafiei, Alberto Raggi, Fakher Rahim, Mehran Rahimi, Mahban Rahimifard, Vafa Rahimi-Movaghar, Mohammad Hifz Ur Rahman, Shayan Rahmani, Premkumar Ramasubramani, Nemanja Rancic, Indu Ramachandra Rao, Nakul Ravikumar, Salman Rawaf, Elrashdy Moustafa Mohamed Redwan, Nazila Rezaei, Nima Rezaei, Daniela Ribeiro, Mónica Rodrigues, Jefferson Antonio Buendia Rodriguez, Leonardo Roever, Aly M A Saad, Basema Saddik, Umar Saeed, Mahdi Safdarian, Sher Zaman Safi, Dominic Sagoe, Fatemeh Saheb Sharif-Askari, Narjes Saheb Sharif-Askari, Amirhossein Sahebkar, Mohammad Ali Sahraian, Mirza Rizwan Sajid, Sateesh Sakhamuri, leili salehi, Amir Salek Farrokhi, Sara Samadzadeh, Saad Samargandy, Noosha Samieefar, Abdallah M Samy, Nima Sanadgol, Ganesh Kumar Saya, Art Schuermans, Sadaf G Sepanlou, Yashendra Sethi, Mahan Shafie, Izza Shahid, Samiah Shahid, Manoj Sharma, Jeevan K Shetty, K M Shivakumar, Velizar Shivarov, Parnian Shobeiri, Migbar Mekonnen Sibhat, Emmanuel Edwar Siddig, Colin R Simpson, Harpreet Singh, Jasvinder A Singh, Paramdeep Singh, Abdullah Al Mamun Sohag, Ranjan Solanki, Solikhah Solikhah, Yonatan Solomon, Seyyed Mohammad Tabatabaei, Mohammad Tabish, Ensiyeh Taheri, Iman M Talaat, Lai-shan Tam, Jacques JL Lukenze Tamuzi, Ker-Kan Tan, Nathan Y Tat, Razieh Tavakoli Oliaee, Arian Tavasol, Mohamad-Hani Temsah, Pugazhenthan Thangaraju, Samar Tharwat, Jansje Henny Vera Ticoalu, Tala Tillawi, Tenaw Yimer Tiruye, Marcos Roberto Tovani-Palone, Guesh Mebrahtom Tsegay, Sree Sudha Ty, Chukwudi S Ubah, Saif Ullah, Muhammad Umair, Srikanth Umakanthan, Era Upadhyay, Asokan Govindaraj Vaithinathan, Sahel Valadan Tahbaz, Shoban Babu Varthya, Tommi Juhani Vasankari, Narayanaswamy Venketasubramanian, Georgios-Ioannis Verras, Jorge Hugo Villafañe, Vasily Vlassov, Danh Cao Vo, Ronny Westerman, Dakshitha Praneeth Wickramasinghe, Nuwan Darshana Wickramasinghe, Barbara Willekens, Beshada Zerfu Woldegeorgis, Dongze Wu, Hong Xiao, Yuhan Xing, Galal Yahya, Lin Yang, Dong Keon Yon, Mazyar Zahir, Syed Saoud Zaidi, Iman Zare, Dawit T Zemedikun, Naod Gebrekrstos Zeru, Hanqing Zhao, Chenwen Zhong, Magdalena Zielińska, Mohammad Zoladl, Alimuddin Zumla.

Part 5. Managing the estimation or publications process

Melsew Dagne Abate, Ali Ahmadi, Ganiyu Adeniyi Amusa, Judie Arulappan, Ahmed Y Azzam, Rosa A S Couto, Thanh Chi Do, Ali Fatehizadeh, Yang Guo, Hong-Han Huynh, Farideh Iravanpour, Morteza Jafarinia, Sathish Kumar Jayapal, Jafar Karami, Chandrakant Lahariya, Thao Thi Thu Le, Tuan A Mai, Le Huu Nhat Minh, Ali H Mokdad, George Duke Mukoro, Getaneh Baye Mulu, Hien Quang Nguyen, Phat Tuan Nguyen, Van Thanh Nguyen, Mahesh P A, Hoang Tran Pham, Nemanja Rancic, Nima Rezaei, Aly M A Saad, Abdallah M Samy, Shoban Babu Varthya, Dongze Wu, Dong Keon Yon.

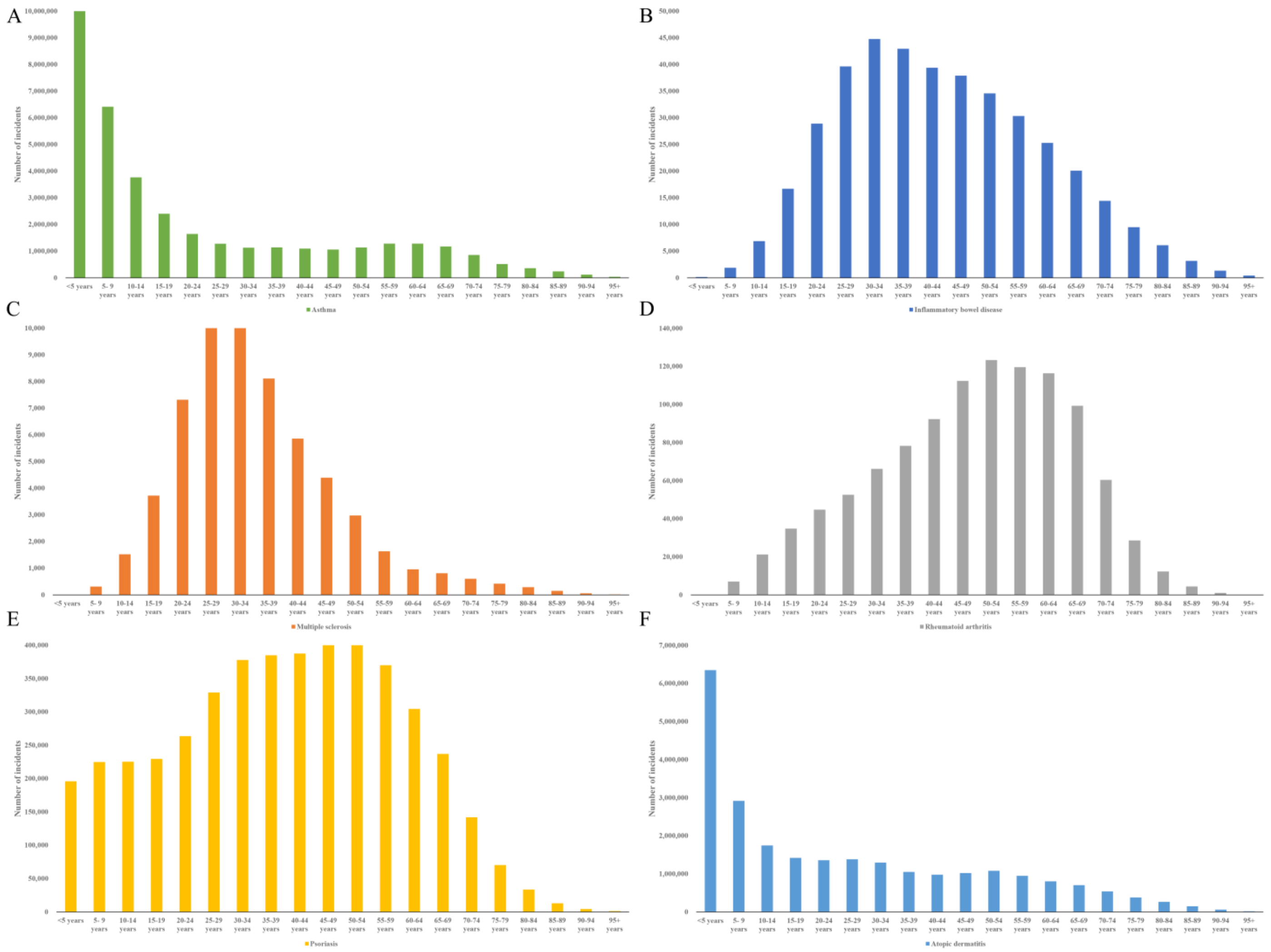

sFigure 1. The number of incident cases attributable to six immune mediated inflammatory diseases throughout human lifespan in 2019

The number of incident cases attributable to asthma throughout human lifespan in 2019 (A),

The number of incident cases attributable to inflammatory bowel disease throughout human lifespan in 2019 (B),

The number of incident cases attributable to multiple sclerosis throughout human lifespan in 2019 (C),

The number of incident cases attributable to rheumatoid arthritis throughout human lifespan in 2019 (D),

The number of incident cases attributable to psoriasis throughout human lifespan in 2019(E),

The number of incident cases attributable to atopic dermatitis throughout human lifespan in 2019 (F).

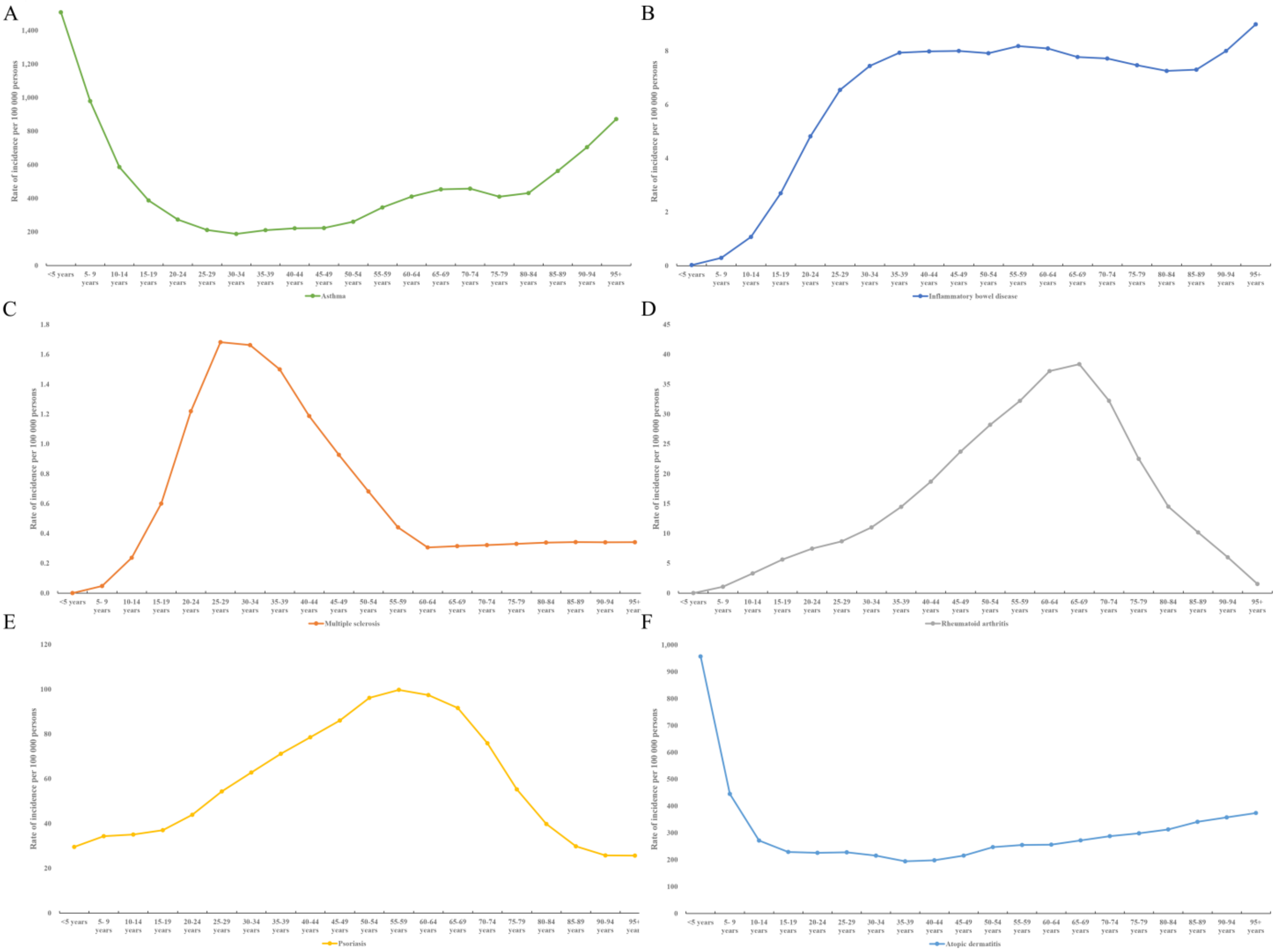

sFigure 2. The rate of incidence attributable to six immune mediated inflammatory diseases throughout human lifespan in 2019

The rate of incident cases attributable to asthma throughout human lifespan in 2019 (A),

The rate of incident cases attributable to inflammatory bowel disease throughout human lifespan in 2019 (B),

The rate of incident cases attributable to multiple sclerosis throughout human lifespan in 2019 (C),

The rate of incident cases attributable to rheumatoid arthritis throughout human lifespan in 2019 (D),

The rate of incident cases attributable to psoriasis throughout human lifespan in 2019 (E),

The rate of incident cases attributable to atopic dermatitis throughout human lifespan in 2019 (F),

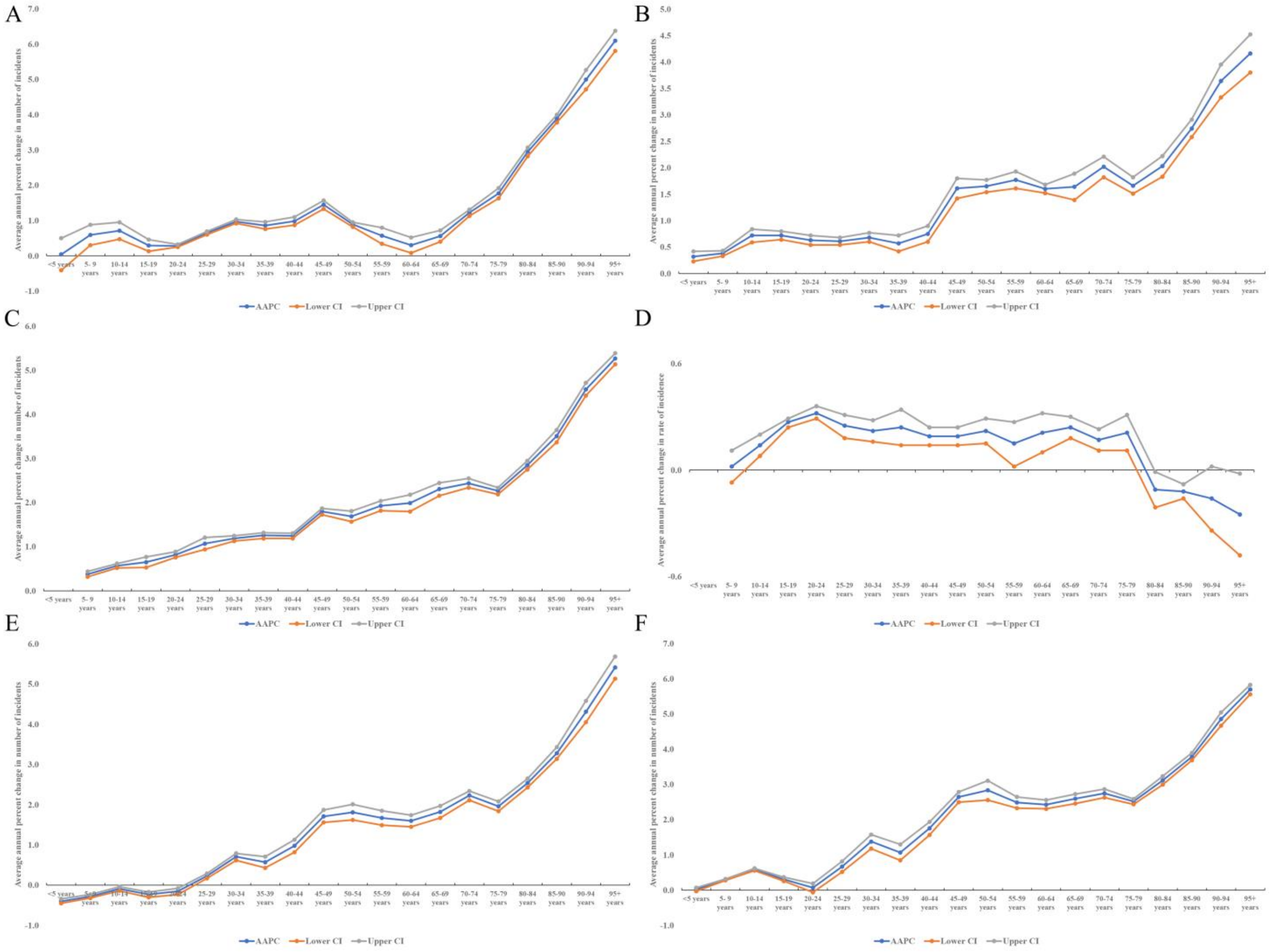

sFigure 3. The average annual percent change in number of incidents attributable to six immune mediated inflammatory diseases throughout human lifespan, 1990-2019

The average annual percent change in number of incident cases attributable to asthma throughout human lifespan, 1990-2019 (A),

The average annual percent change in number of incident cases attributable to inflammatory bowel disease throughout human lifespan, 1990-2019(B),

The average annual percent change in number of incident cases attributable to multiple sclerosis throughout human lifespan, 1990-2019 (C),

The average annual percent change in number of incident cases attributable to rheumatoid arthritis throughout human lifespan, 1990-2019(D),

The average annual percent change in number of incident cases attributable to psoriasis throughout human lifespan, 1990-2019(E),

The average annual percent change in number of incident cases attributable to atopic dermatitis throughout human lifespan, 1990-2019 (F).

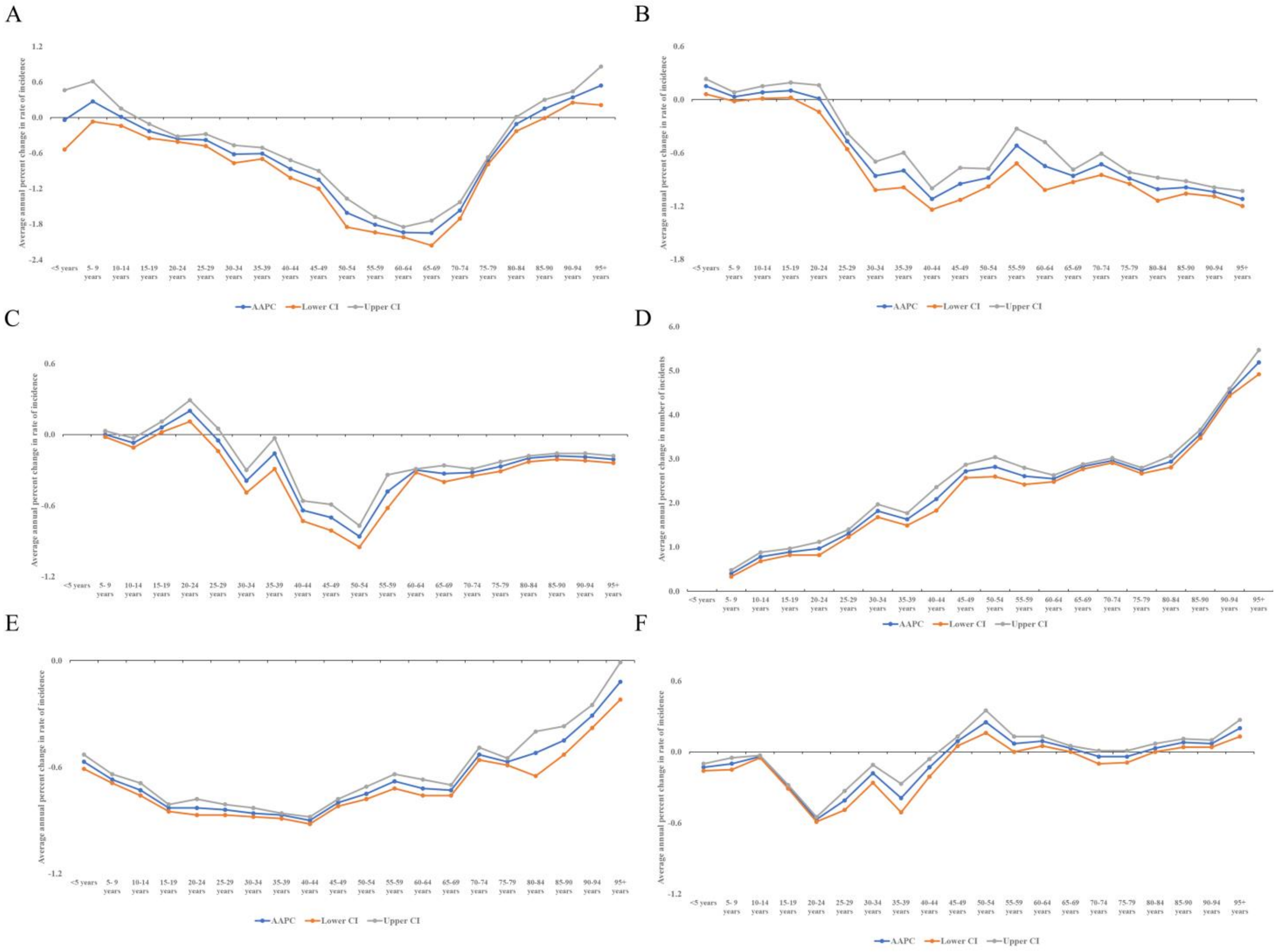

sFigure 4. The average annual percent change in rate of incidence attributable to six immune mediated inflammatory diseases throughout human lifespan, 1990-2019

The average annual percent change in rate of incidence attributable to asthma throughout human lifespan, 1990-2019 (A),

The average annual percent change in rate of incidence attributable to inflammatory bowel disease throughout human lifespan, 1990-2019(B),

The average annual percent change in rate of incidence attributable to multiple sclerosis throughout human lifespan, 1990-2019(C),

The average annual percent change in rate of incidence attributable to rheumatoid arthritis throughout human lifespan, 1990-2019 (D),

The average annual percent change in rate of incidence attributable to psoriasis throughout human lifespan, 1990-2019(E),

The average annual percent change in rate of incidence attributable to atopic dermatitis throughout human lifespan, 1990-2019 (F),

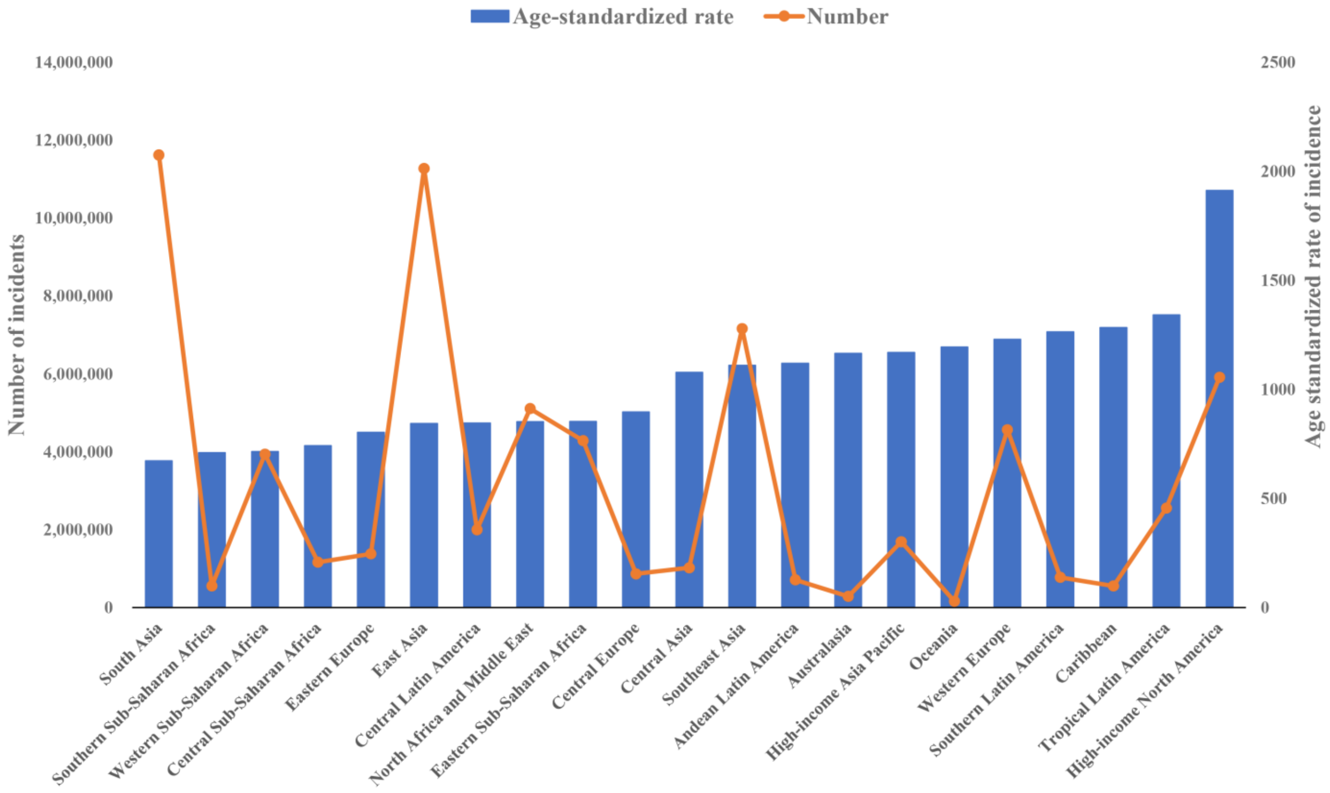

sFigure 5. The incident cases and age-standardized rate of incidence attributable to overall immune mediated inflammatory diseases according to GBD regions in 2019

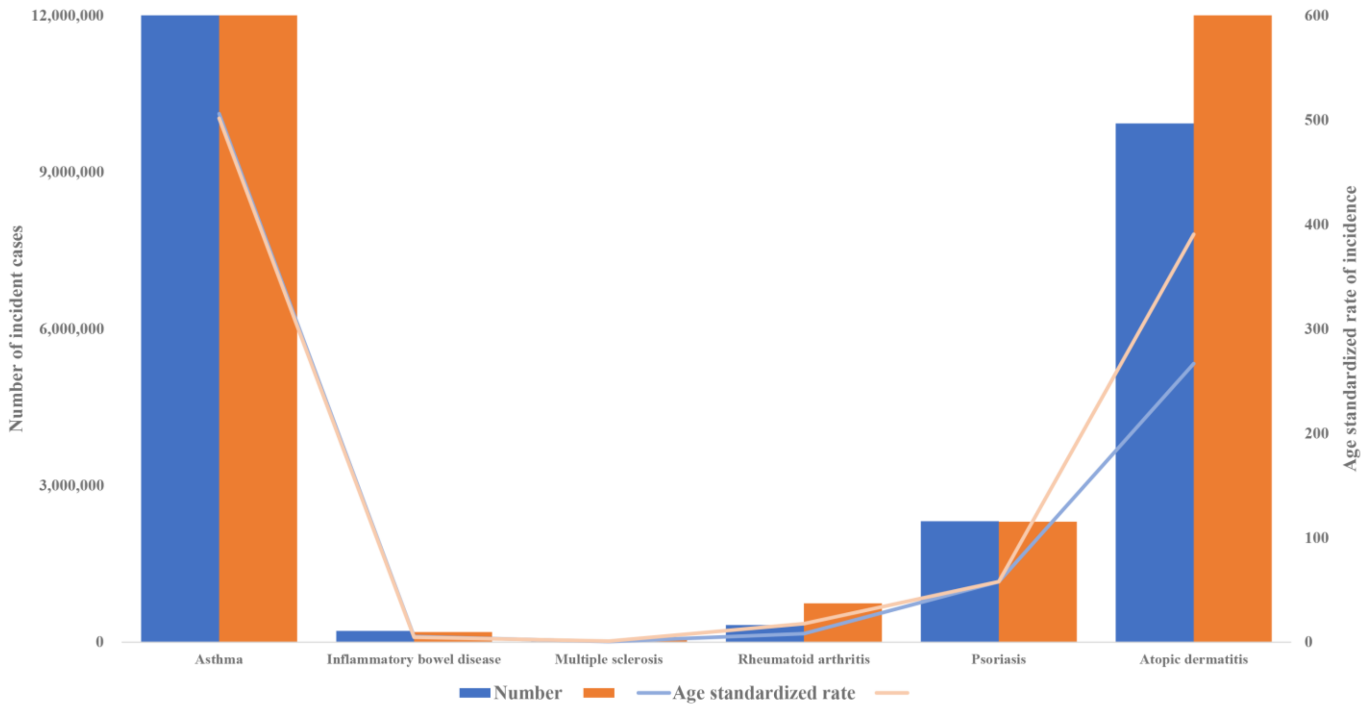

sFigure 6. The incident cases and age-standardized rate of incidence attributable to overall immune mediated inflammatory diseases according to gender in 2019

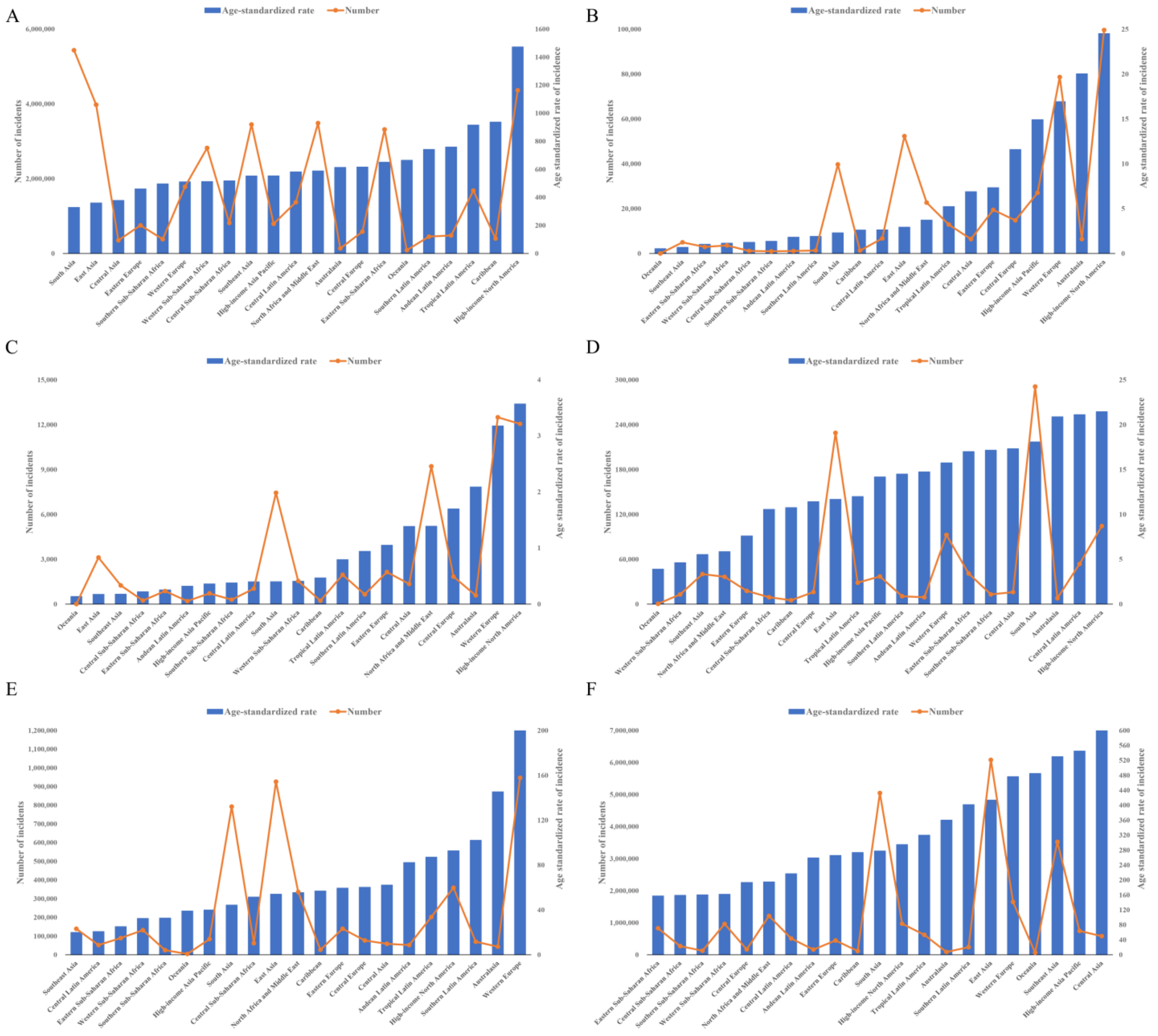

sFigure 7. The incident cases and age-standardized rate of incidence attributable to six immune mediated inflammatory diseases according to GBD regions in 2019

The incident cases and age-standardized rate of incidence attributable to asthma according to GBD regions(A),

The incident cases and age-standardized rate of incidence attributable to inflammatory bowel disease according to GBD regions (B),

The incident cases and age-standardized rate of incidence attributable to multiple sclerosis according to GBD regions (C),

The incident cases and age-standardized rate of incidence attributable to rheumatoid arthritis according to GBD regions (D),

The incident cases and age-standardized rate of incidence attributable to psoriasis according to GBD regions (E),

The incident cases and age-standardized rate of incidence attributable to atopic dermatitis according to GBD regions (F).

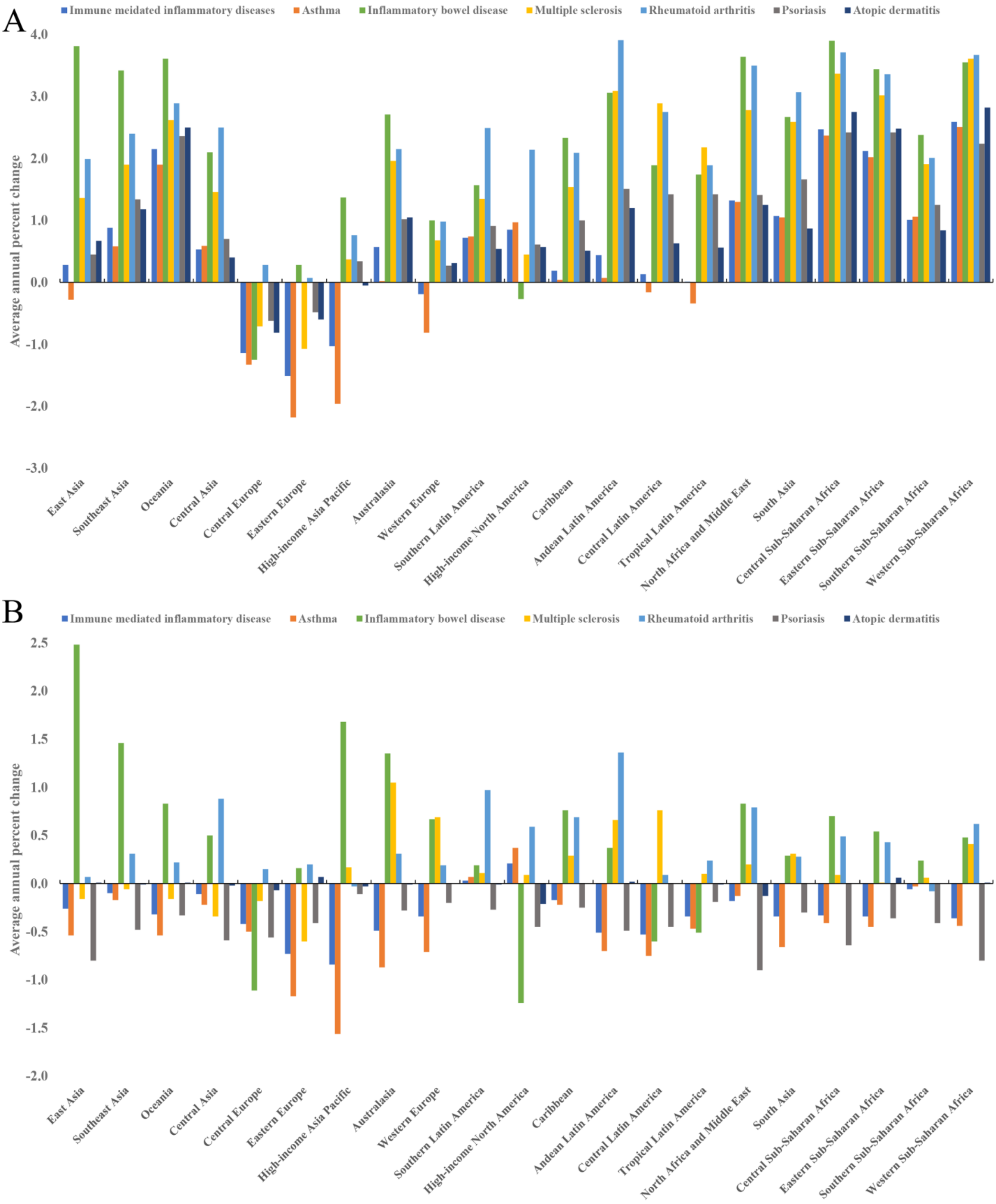

sFigure 8. The average annual percent change in number of incidents and age standardized rate of incidence attributable to six immune mediated inflammatory diseases across 21 GBD regions, 1990-2019

The average annual percent change in number of incidents attributable to six immune mediated inflammatory diseases across 21 GBD regions, 1990-2019 (A)

The average annual percent change in age standardized rate of incidence attributable to six immune mediated inflammatory diseases across 21 GBD regions, 1990-2019 (B)

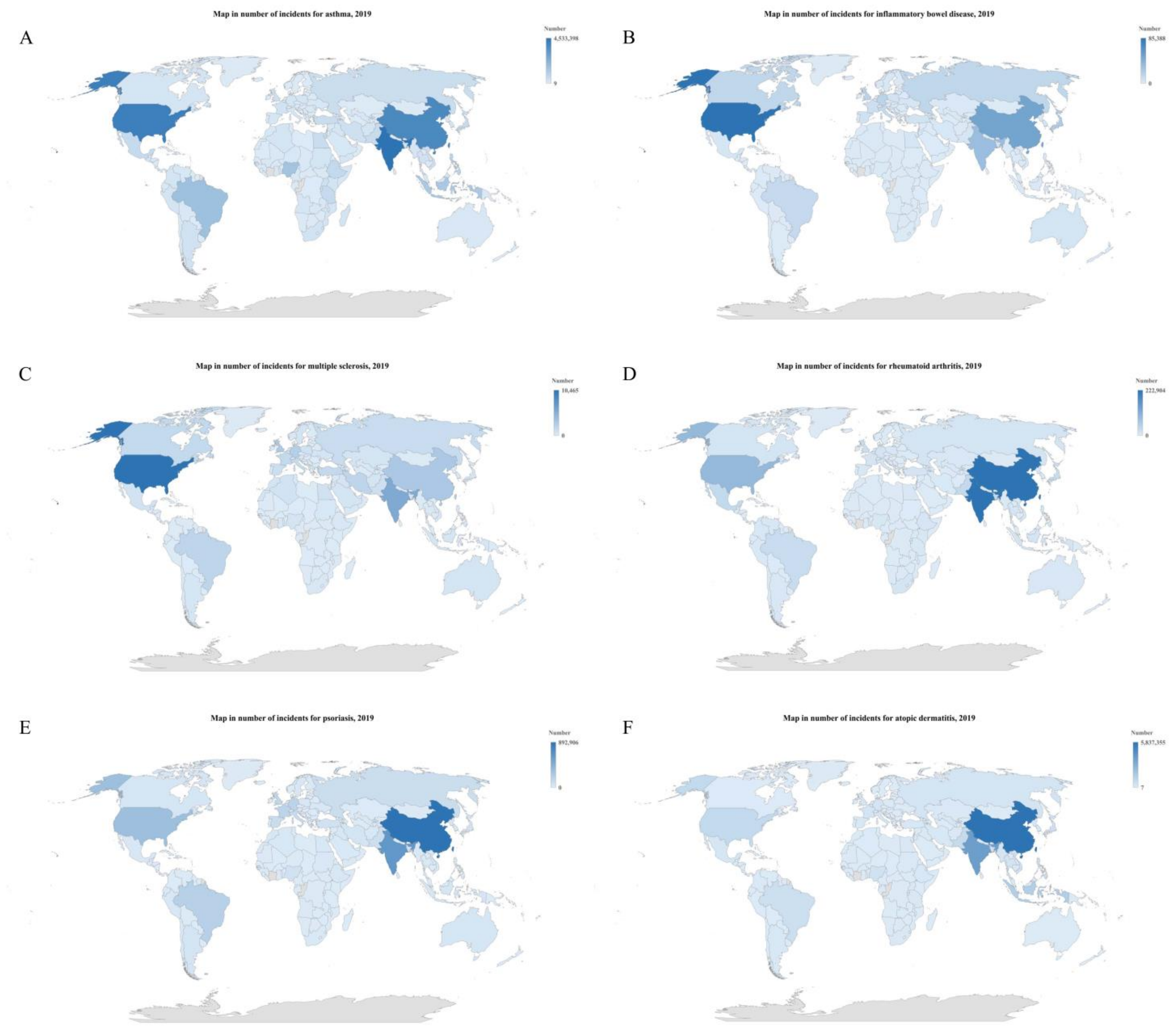

sFigure 9. The global map in number of incidents attributable to six immune mediated inflammatory diseases in 204 countries and territories, 2019

The global map in number of incident cases attributable to asthma, 2019 (A),

The global map in number of incident cases attributable to inflammatory bowel disease, 2019 (B),

The global map in number of incident cases attributable to multiple sclerosis, 2019 (C),

The global map in number of incident cases attributable to rheumatoid arthritis, 2019 (D),

The global map in number of incident cases attributable to psoriasis, 2019 (E),

The global map in number of incident cases attributable to atopic dermatitis, 2019 (F).

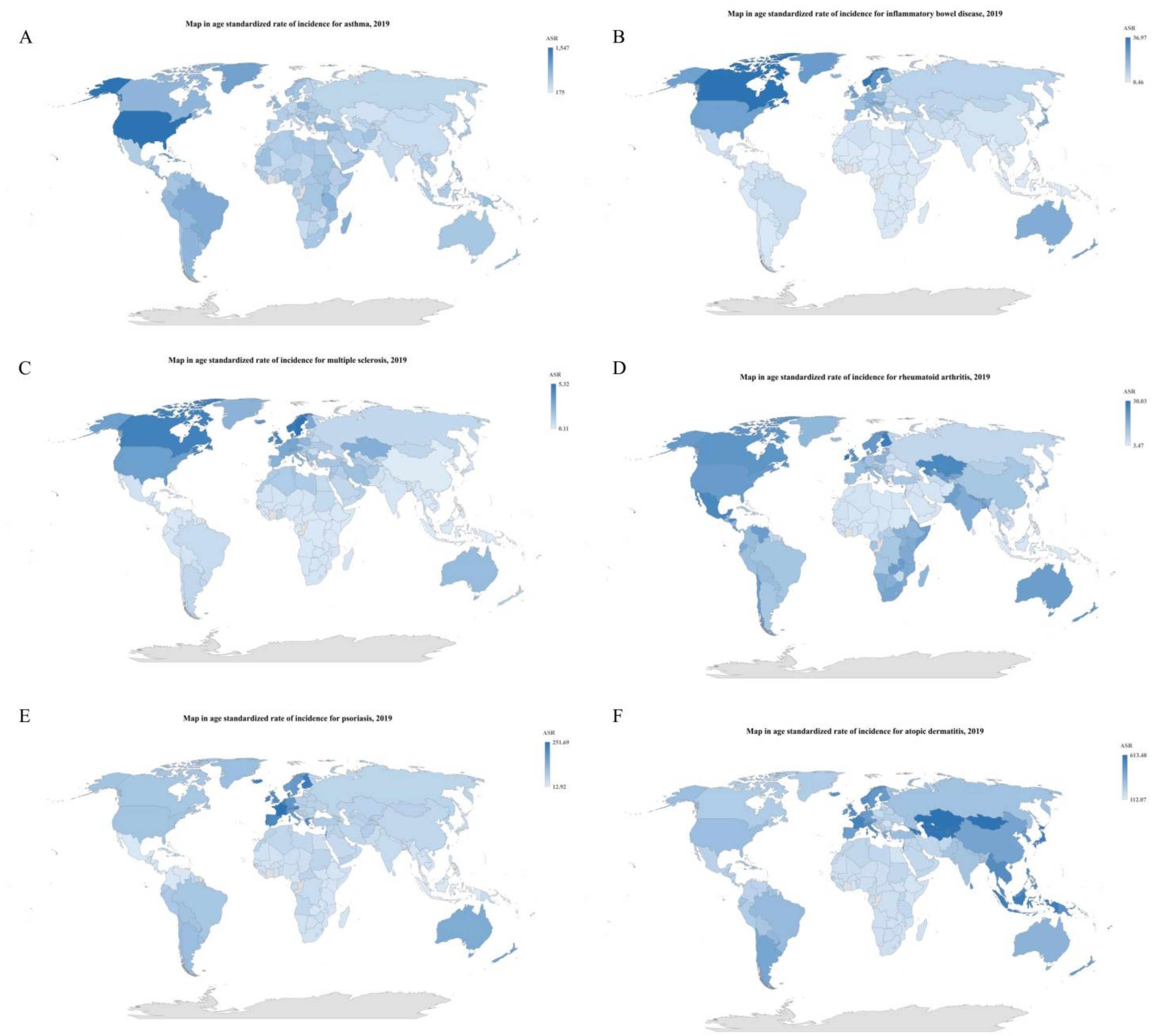

sFigure 10. The global map in age standardized rate of incidence attributable to six immune mediated inflammatory diseases in 204 countries and territories, 2019

The global map in age standardized rate of incidence attributable to asthma, 2019 (A),

The global map in age standardized rate of incidence attributable to inflammatory bowel disease, 2019 (B),

The global map in age standardized rate of incidence attributable to multiple sclerosis, 2019 (C),

The global map in age standardized rate of incidence attributable to rheumatoid arthritis, 2019 (D),

The global map in age standardized rate of incidence attributable to psoriasis, 2019 (E),

The global map in age standardized rate of incidence attributable to atopic dermatitis, 2019 (F).

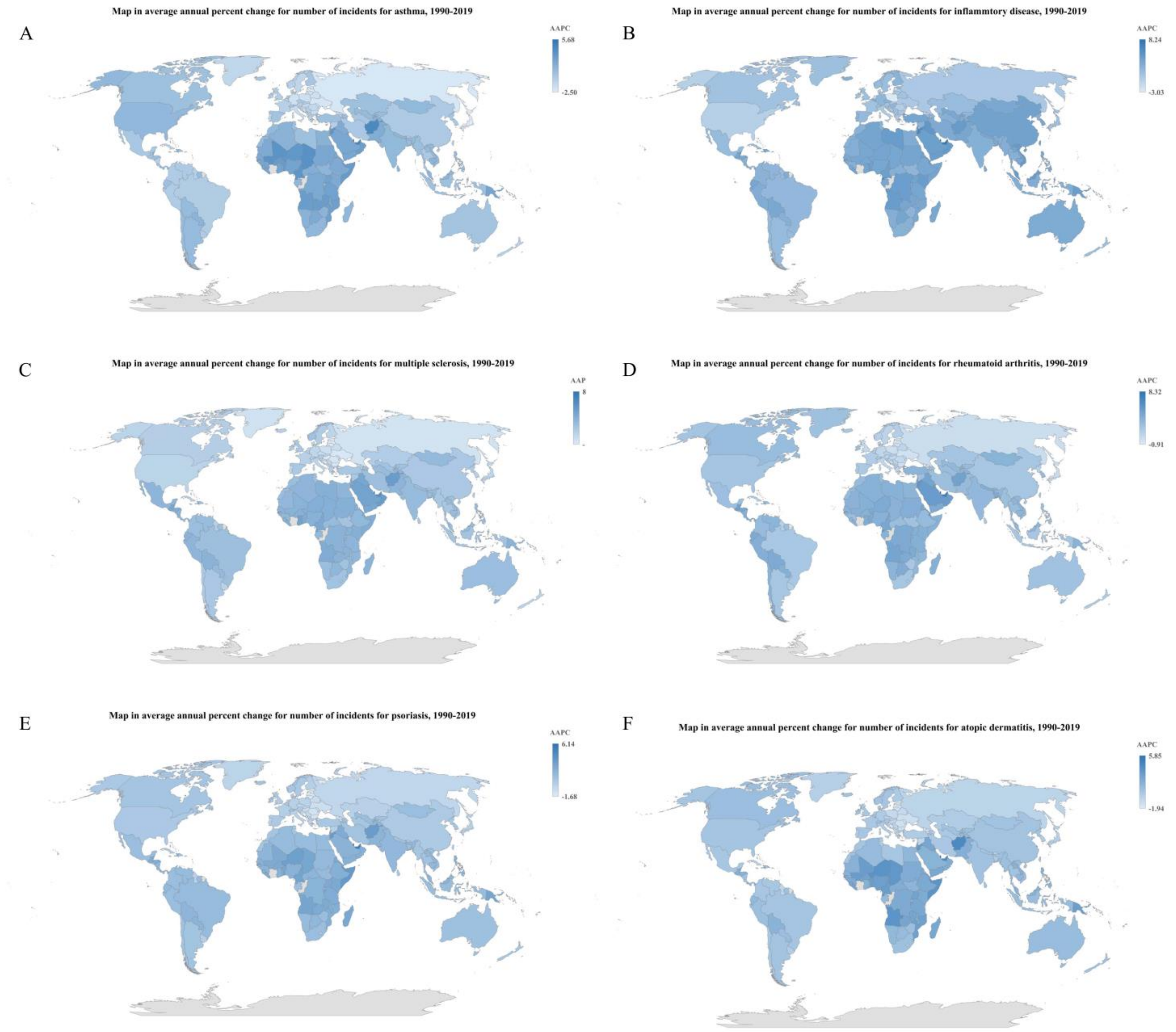

sFigure 11. The global map in average annual percent change in number of incidence attributable to six immune mediated inflammatory diseases in 204 countries and territories, 1990-2019

The global map in average annual percent change in number of incident cases attributable to asthma, 1990-2019 (A),

The global map in average annual percent change in number of incident cases attributable to inflammatory bowel disease, 1990-2019 (B),

The global map in average annual percent change in number of incident cases attributable to multiple sclerosis, 1990-2019 (C),

The global map in average annual percent change in number of incident cases attributable to rheumatoid arthritis, 1990-2019 (D),

The global map in average annual percent change in number of incident cases attributable to psoriasis, 1990-2019 (E),

The global map in average annual percent change in number of incident cases attributable to atopic dermatitis, 1990-2019 (F).

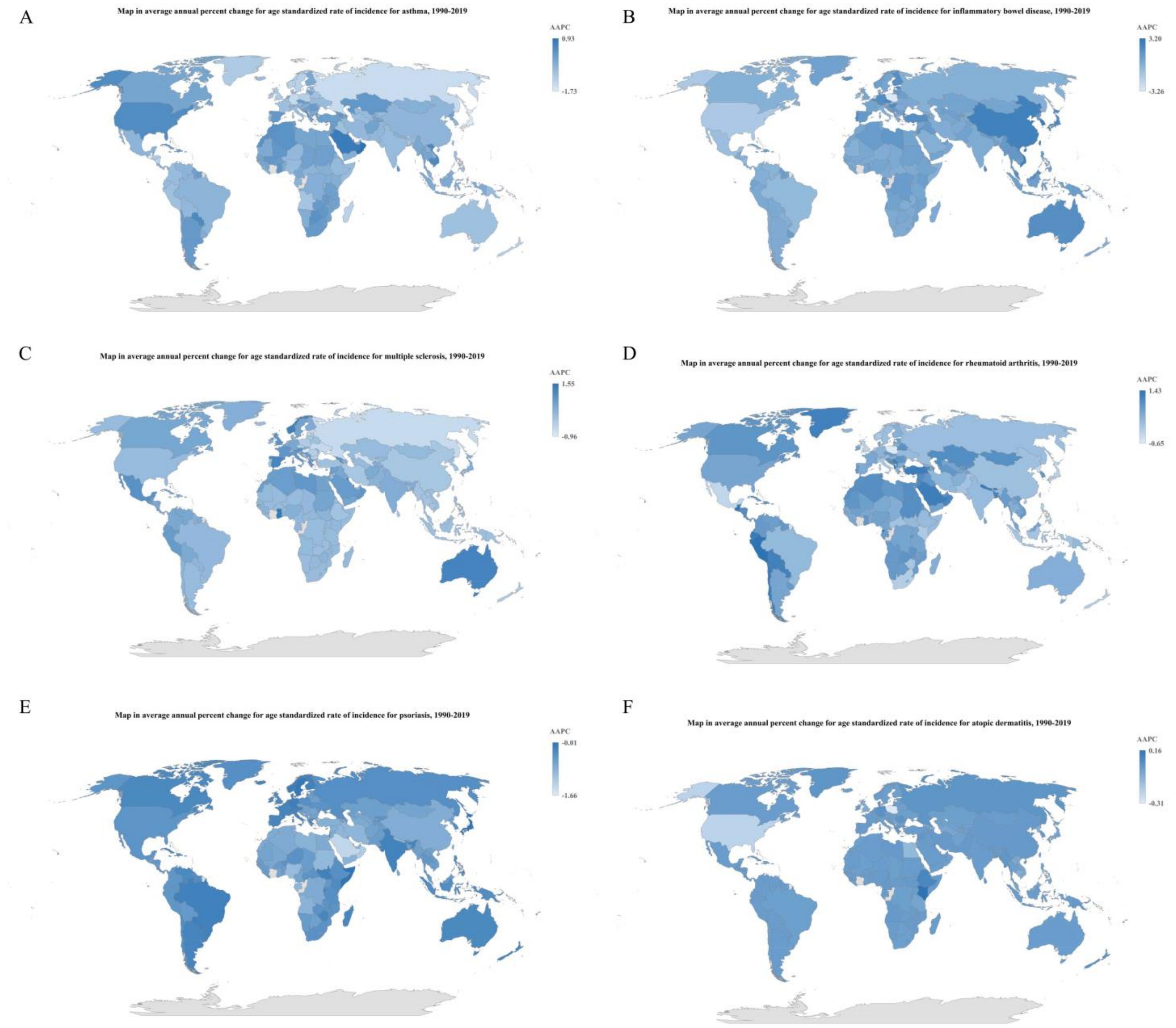

sFigure 12. The global map in average annual percent change in age standardized rate of incidence attributable to six immune mediated inflammatory diseases in 204 countries and territories, 1990-2019

The global map in average annual percent change in age standardized rate of incidence attributable to asthma, 1990-2019 (A),

The global map in average annual percent change in age standardized rate of incidence attributable to inflammatory bowel disease, 1990-2019 (B),

The global map in average annual percent change in age standardized rate of incidence attributable to multiple sclerosis, 1990-2019 (C),

The global map in average annual percent change in age standardized rate of incidence attributable to rheumatoid arthritis, 1990-2019 (D),

The global map in average annual percent change in age standardized rate of incidence attributable to psoriasis, 1990-2019 (E),

The global map in average annual percent change in age standardized rate of incidence attributable to atopic dermatitis, 1990-2019 (F).

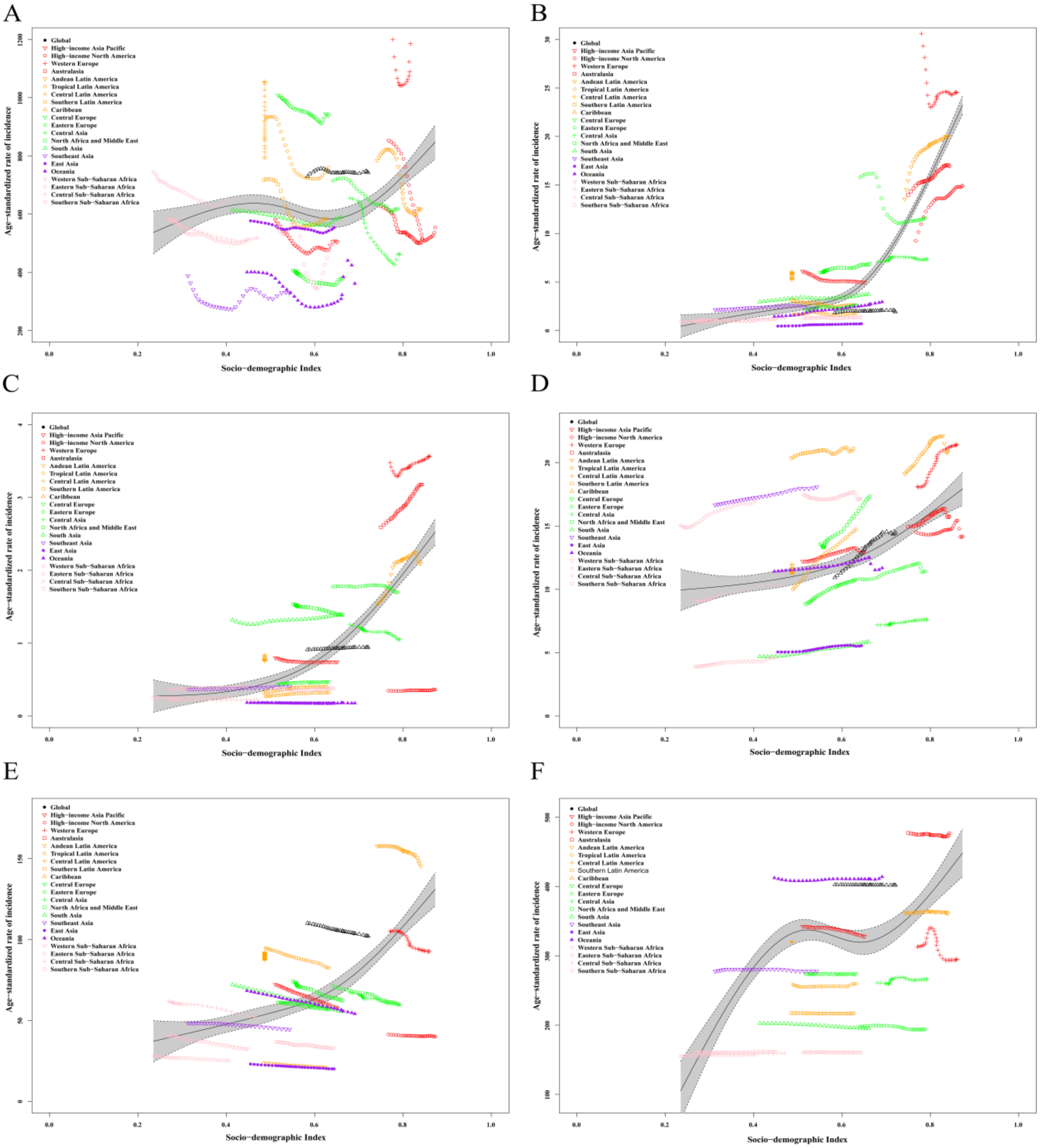

sFigure 13. Age standardized rate of incidence attributable to six immune mediated inflammatory diseases per 100 000 persons for sociodemographic index by 21 GBD regions, 2019

Age standardized rate of incidence attributable to asthma per 100 000 persons for sociodemographic index by 21 GBD regions, 2019 (A), Age standardized rate of incidence attributable to inflammatory bowel disease per 100 000 persons for sociodemographic index by 21 GBD regions, 2019 (B), Age standardized rate of incidence attributable to multiple sclerosis per 100 000 persons for sociodemographic index by 21 GBD regions, 2019 (C), Age standardized rate of incidence attributable to rheumatoid arthritis per 100 000 persons for sociodemographic index by 21 GBD regions, 2019 (D), Age standardized rate of incidence attributable to psoriasis per 100 000 persons for sociodemographic index by 21 GBD regions, 2019 (E), Age standardized rate of incidence attributable to atopic dermatitis per 100 000 persons for sociodemographic index by 21 GBD regions, 2019 (F). Black line represents expected values based on sociodemographic index and disease rates across 21 Global Burden of Disease regions; each point shows observed age standardized rate of incidence for specified Global Burden of Disease region in 2019.

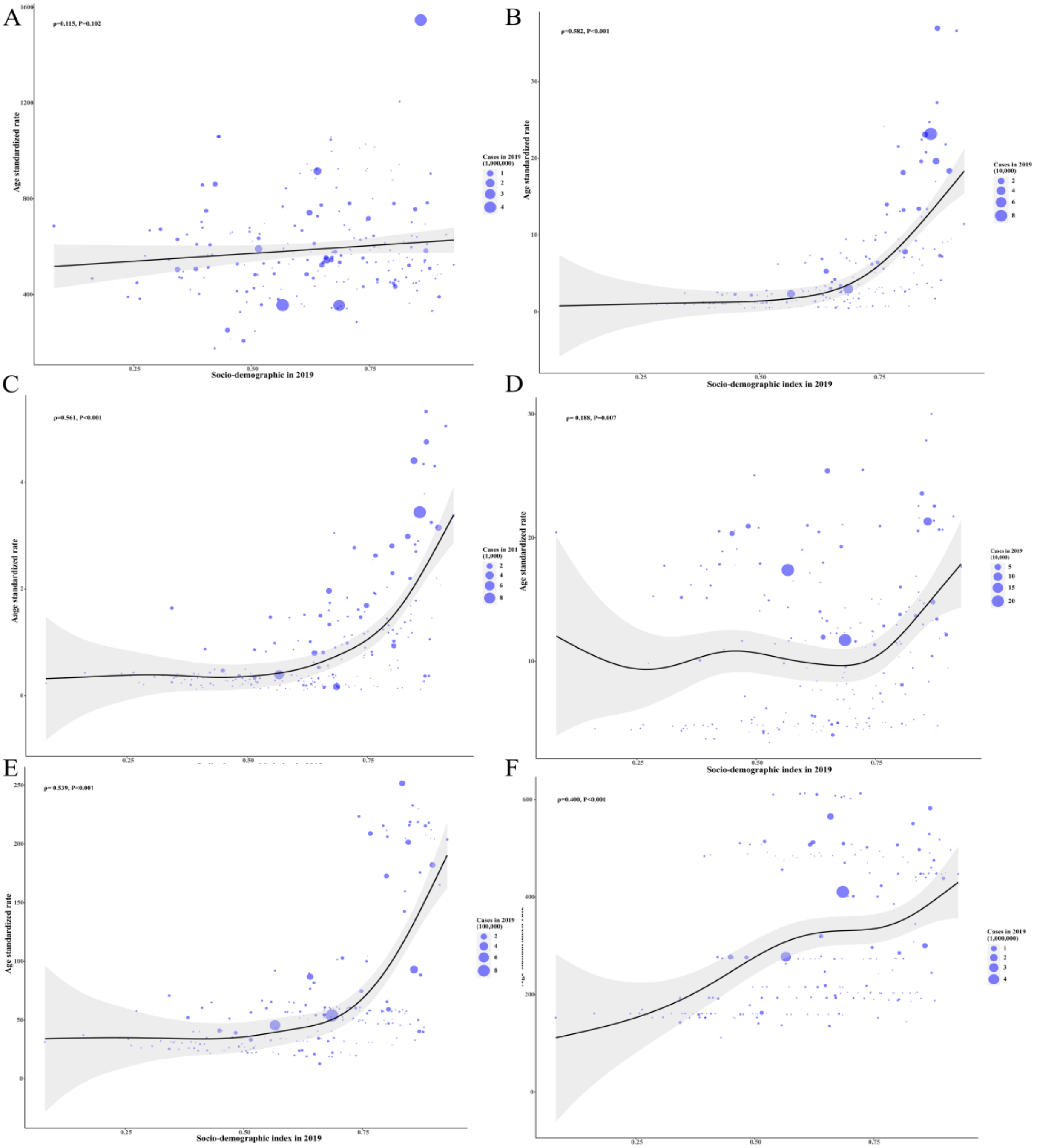

sFigure 14. Age standardized rate of incidence attributable to six immune mediated inflammatory diseases per 100 000 persons for sociodemographic index by 204 countries and territories, 2019

Age standardized rate of incidence attributable to asthma per 100 000 persons for sociodemographic index by 204 countries and territories, 2019 (A),Age standardized rate of incidence attributable to inflammatory bowel disease per 100 000 persons for sociodemographic index by 204 countries and territories, 2019 (B), Age standardized rate of incidence attributable to multiple sclerosis per 100 000 persons for sociodemographic index by 204 countries and territories, 2019 (C),Age standardized rate of incidence attributable to rheumatoid arthritis per 100 000 persons for sociodemographic index by 204 countries and territories, 2019 (D), Age standardized rate of incidence attributable to psoriasis per 100 000 persons for sociodemographic index by 204 countries and territories, 2019 (E), Age standardized rate of incidence attributable to atopic dermatitis per 100 000 persons for sociodemographic index by 204 countries and territories, 2019 (F), Black line represents expected values based on sociodemographic index and disease across 204 countries and territories, each point shows observed age standardized rate of incidence for specified country in 2019.

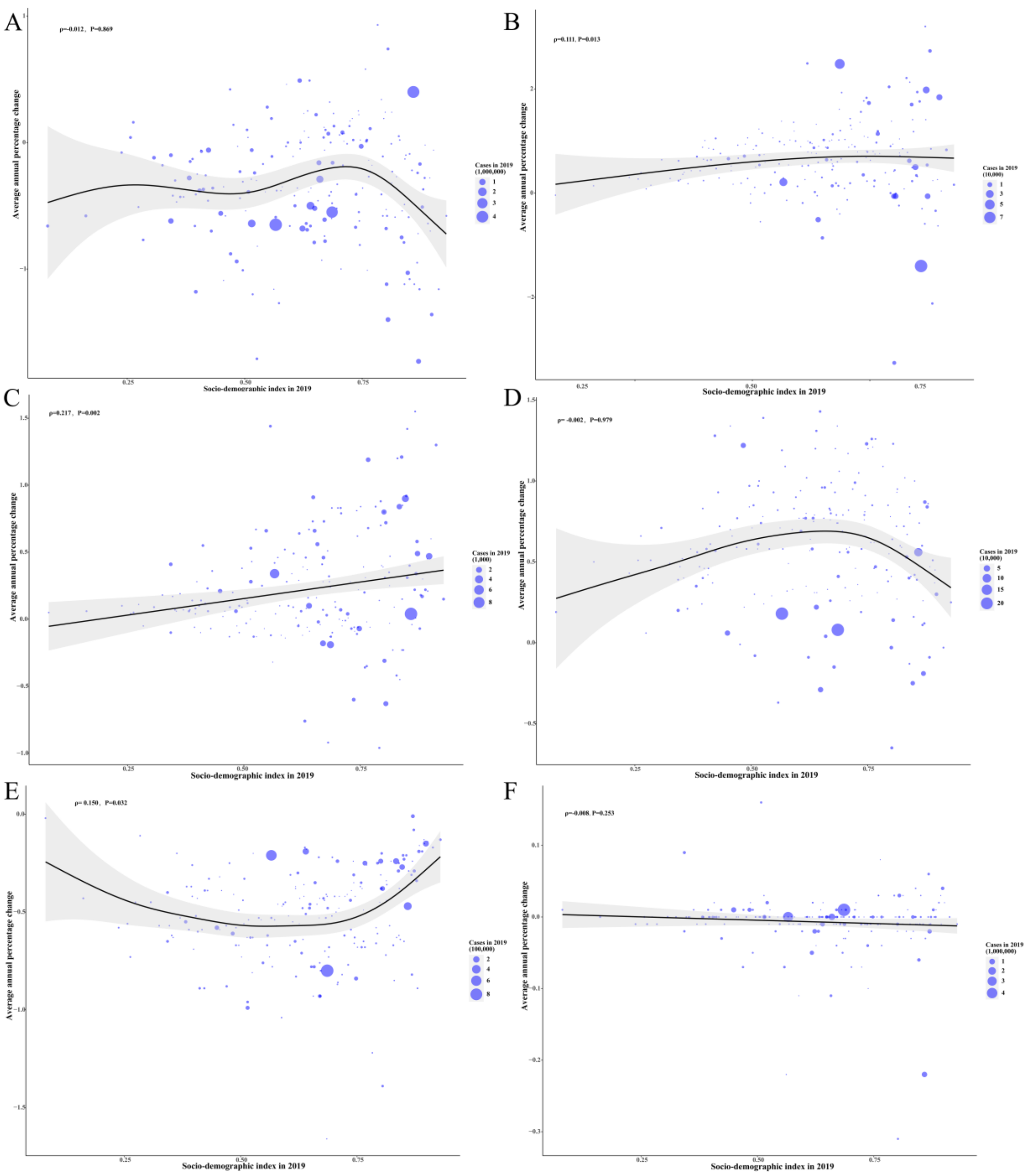

sFigure 15. The correlation between average annual percent change and age standardized rate of incidence attributable to six immune mediated inflammatory in 1990 across 204 countries and territories

The correlation between average annual percent change and age standardized rate of incidence attributable to asthma in 1990 across 204 countries and territories (A), The correlation between average annual percent change and age standardized rate of incidence attributable to inflammatory bowel disease in 1990 across 204 countries and territories (B), The correlation between average annual percent change and age standardized rate of incidence attributable to multiple sclerosis in 1990 across 204 countries and territories (C), The correlation between average annual percent change and age standardized rate of incidence attributable to rheumatoid arthritis in 1990 across 204 countries and territories (D), The correlation between average annual percent change and age standardized rate of incidence attributable to psoriasis in 1990 across 204 countries and territories (E), The correlation between average annual percent change and age standardized rate of incidence attributable to atopic dermatitis in 1990 across 204 countries and territories (F). The size of circle is increased with the incident cases of immune mediated inflammatory diseases. The ρ indices and p values were derived from Pearson correlation analysis.

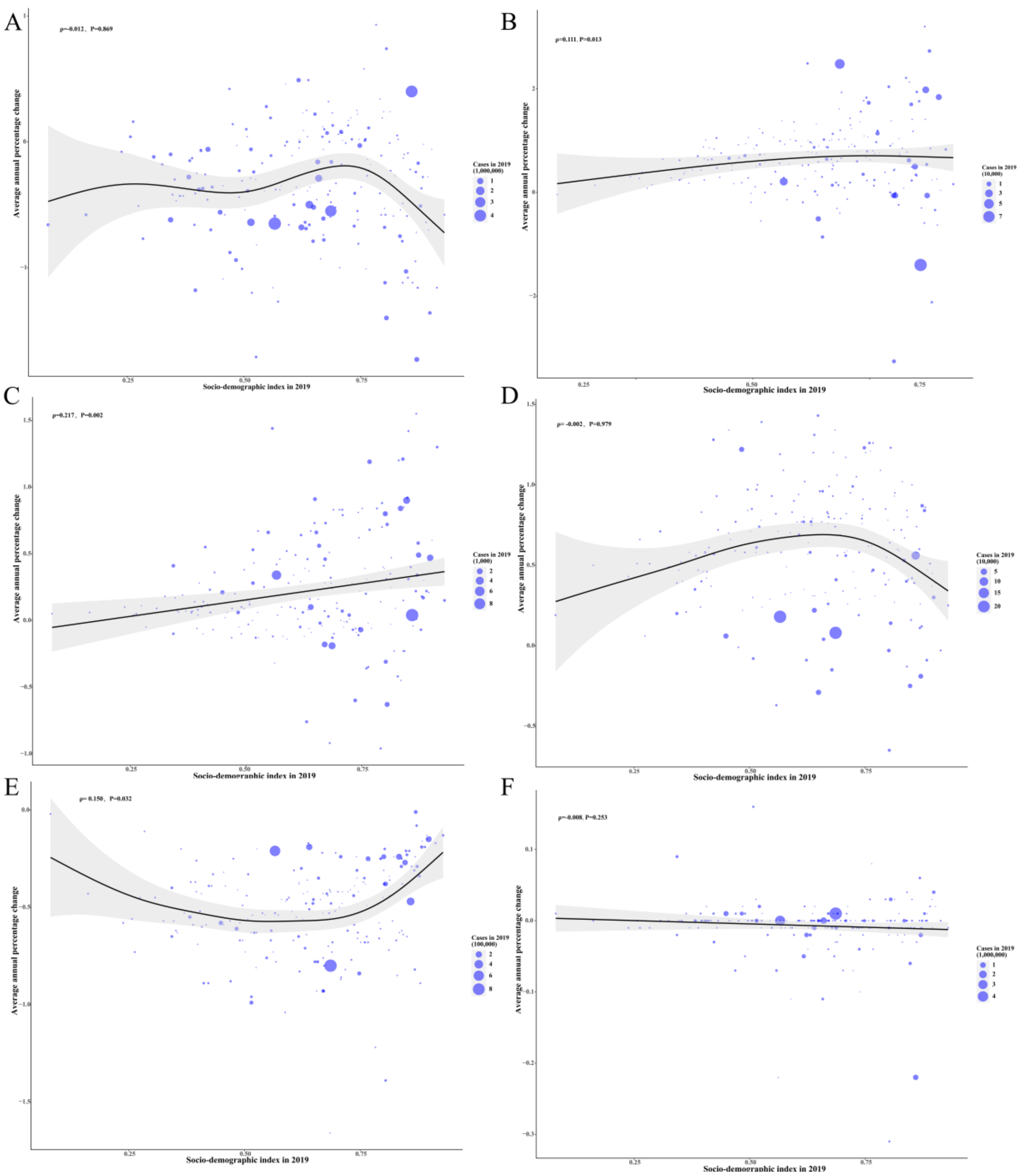

sFigure 16. The correlation between average annual percent change and socio-demographic index attributable to six immune mediated inflammatory in 2019 across 204 countries and territories

The correlation between average annual percent change and socio-demographic index attributable to asthma in 2019 across 204 countries and territories (A), The correlation between average annual percent change and socio-demographic index attributable to inflammatory bowel disease in 2019 across 204 countries and territories (B), The correlation between average annual percent change and socio-demographic index attributable to multiple sclerosis in 2019 across 204 countries and territories (C), The correlation between average annual percent change and socio-demographic index attributable to rheumatoid arthritis in 2019 across 204 countries and territories (D), The correlation between average annual percent change and socio-demographic index attributable to psoriasis in 2019 across 204 countries and territories (E), The correlation between average annual percent change and socio-demographic index attributable to atopic dermatitis in 2019 across 204 countries and territories (F). The size of circle is increased with the incident cases of immune mediated inflammatory diseases. The ρ indices and p values were derived from Pearson correlation analysis.

sTable 1. The incident cases and rate of incidence attributable to overall immune mediated inflammatory diseases across 20 age groups, and its temporal trends from 1990 to 2019

	1990		2019		1990-1999	2000-2009	2010-2019	1990-2019	
	N(95%CI)	Rank	N(95%CI)	Rank	AAPC(95%CI)	AAPC(95%CI)	AAPC(95%CI)	AAPC(95%CI)	Rank
**Rate**									
<5 years	2,636.03(1,951.96-3,566.38)	1	2,496.39(1,842.16-3,392.48)	1	-0.95(-1.08,-0.82)	0.28(-0.14,0.69)	0.32(-0.45,1.09)	-0.08(-0.38,0.21)	6
5- 9 years	1,448.88(909.32-2,177.48)	2	1,461.23(914.22-2,212.18)	2	-0.80(-0.98,-0.62)	0.66(0.46,0.86)	0.57(-0.32,1.46)	0.13(-0.16,0.42)	3
10-14 years	906.48(534.82-1,313.47)	10	898.33(527.74-1,301.27)	6	-0.53(-0.64,-0.42)	-0.24(-0.27,-0.20)	0.71(0.51,0.91)	-0.03(-0.11,0.04)	5
15-19 years	722.89(500.98-997.29)	14	662.82(454.87-915.71)	13	-0.33(-0.40,-0.25)	-0.77(-0.86,-0.67)	0.24(0.02,0.47)	-0.28(-0.36,-0.20)	8
20-24 years	640.33(430.92-913.63)	16	557.52(368.91-803.26)	16	-0.47(-0.48,-0.45)	-0.71(-0.73,-0.69)	-0.25(-0.28,-0.23)	-0.47(-0.49,-0.46)	12
25-29 years	582.97(419.45-771.75)	19	510.74(367.34-674.87)	18	-0.61(-0.63,-0.58)	-0.67(-0.69,-0.65)	-0.06(-0.09,-0.03)	-0.46(-0.48,-0.44)	11
30-34 years	553.88(391.80-745.40)	20	486.38(347.28-650.25)	20	-0.64(-0.68,-0.61)	-0.72(-0.74,-0.70)	0.07(0.03,0.12)	-0.44(-0.46,-0.42)	9
35-39 years	583.69(401.26-826.28)	18	500.43(348.52-705.93)	19	-0.92(-0.96,-0.89)	-0.68(-0.71,-0.65)	0.01(-0.05,0.06)	-0.53(-0.56,-0.51)	13
40-44 years	623.90(432.86-871.95)	17	526.34(370.58-723.97)	17	-1.04(-1.08,-1.00)	-0.70(-0.76,-0.64)	-0.02(-0.16,0.12)	-0.60(-0.65,-0.55)	15
45-49 years	657.27(423.46-959.41)	15	557.82(374.24-791.22)	15	-0.87(-1.01,-0.74)	-0.71(-0.77,-0.64)	-0.05(-0.12,0.01)	-0.59(-0.64,-0.53)	14
50-54 years	799.40(561.86-1,086.71)	13	640.85(458.32-860.75)	14	-1.34(-1.38,-1.31)	-0.86(-0.99,-0.74)	-0.10(-0.19,0.00)	-0.77(-0.83,-0.72)	16
55-59 years	1,002.69(649.36-1,425.19)	8	741.83(505.84-1,031.77)	12	-2.02(-2.08,-1.95)	-0.92(-1.03,-0.81)	-0.06(-0.18,0.05)	-1.02(-1.07,-0.96)	18
60-64 years	1,141.76(791.12-1,531.91)	4	810.02(573.09-1,086.87)	9	-2.24(-2.30,-2.18)	-1.04(-1.17,-0.91)	-0.14(-0.31,0.04)	-1.16(-1.23,-1.09)	19
65-69 years	1,221.90(856.82-1,775.40)	3	864.56(615.60-1,234.48)	7	-2.03(-2.06,-2.00)	-1.23(-1.27,-1.19)	-0.31(-0.52,-0.11)	-1.19(-1.26,-1.12)	20
70-74 years	1,135.73(749.16-1,667.56)	5	862.61(598.05-1,216.89)	8	-1.54(-1.69,-1.39)	-1.22(-1.37,-1.07)	-0.14(-0.22,-0.06)	-0.97(-1.05,-0.89)	17
75-79 years	901.02(652.56-1,195.10)	11	794.52(583.33-1,047.14)	11	-0.53(-0.59,-0.47)	-0.74(-0.77,-0.70)	-0.02(-0.14,0.10)	-0.45(-0.49,-0.40)	10
80-84 years	820.51(568.91-1,157.12)	12	806.46(588.09-1,077.73)	10	0.22(0.15,0.30)	-0.35(-0.40,-0.30)	-0.04(-0.21,0.12)	-0.08(-0.14,-0.02)	7
85-90 years	922.43(696.71-1,204.07)	9	952.86(740.75-1,201.65)	5	0.02(-0.05,0.10)	0.43(0.32,0.54)	-0.13(-0.31,0.06)	0.09(0.02,0.16)	4
90-94 years	1,037.20(724.46-1,423.52)	7	1,103.16(796.24-1,463.06)	4	-0.13(-0.22,-0.04)	1.19(0.87,1.50)	-0.37(-0.44,-0.30)	0.19(0.09,0.30)	2
95+ years	1,135.64(654.11-1,701.07)	6	1,283.43(794.17-1,831.07)	3	-0.25(-0.46,-0.05)	1.97(1.32,2.62)	-0.35(-0.52,-0.17)	0.40(0.18,0.62)	1
**Number**									
<5 years	16,662,759(12,338,677-22,543,654)	1	16,547,167(12,210,642-22,486,820)	1	-1.12(-1.29,-0.94)	0.65(0.04,1.28)	0.63(0.31,0.94)	0.03(-0.20,0.26)	20
5- 9 years	8,478,300(5,321,021-12,741,812)	2	9,566,704(5,985,443-14,483,251)	2	-0.48(-0.60,-0.36)	0.72(0.43,1.02)	1.06(0.44,1.68)	0.44(0.22,0.67)	17
10-14 years	4,864,999(2,870,301-7,049,241)	3	5,768,970(3,389,072-8,356,583)	3	1.20(1.07,1.33)	-0.70(-0.90,-0.49)	1.33(0.88,1.79)	0.63(0.47,0.80)	15
15-19 years	3,756,174(2,603,098-5,181,969)	4	4,106,455(2,818,099-5,673,169)	4	0.49(0.27,0.70)	0.19(0.11,0.28)	0.15(-0.04,0.34)	0.28(0.18,0.38)	18
20-24 years	3,154,768(2,123,026-4,501,246)	5	3,345,898(2,213,995-4,820,734)	5	-0.18(-0.26,-0.09)	1.19(1.11,1.27)	-0.57(-0.83,-0.32)	0.17(0.08,0.26)	19
25-29 years	2,581,639(1,857,487-3,417,662)	6	3,092,408(2,224,163-4,086,139)	6	0.62(0.46,0.77)	0.33(0.25,0.42)	0.84(0.62,1.07)	0.59(0.49,0.68)	16
30-34 years	2,135,902(1,510,899-2,874,481)	7	2,926,733(2,089,724-3,912,752)	7	1.93(1.78,2.09)	-0.22(-0.33,-0.12)	2.00(1.65,2.35)	1.13(1.01,1.26)	12
35-39 years	2,058,936(1,415,418-2,914,626)	8	2,707,196(1,885,392-3,818,909)	10	1.03(0.84,1.22)	0.65(0.53,0.77)	0.95(0.73,1.17)	0.91(0.79,1.03)	14
40-44 years	1,786,185(1,239,248-2,496,313)	11	2,597,206(1,828,598-3,572,394)	12	1.65(1.48,1.83)	1.83(1.66,1.99)	0.53(0.47,0.60)	1.26(1.17,1.35)	11
45-49 years	1,527,559(984,154-2,229,764)	13	2,642,941(1,773,137-3,748,824)	11	2.90(2.64,3.16)	1.46(1.25,1.67)	1.54(1.27,1.81)	1.96(1.79,2.13)	6
50-54 years	1,699,448(1,194,470-2,310,244)	12	2,799,344(2,002,029-3,759,895)	8	0.51(0.30,0.72)	2.05(1.85,2.24)	2.74(2.57,2.91)	1.77(1.65,1.90)	8
55-59 years	1,859,139(1,204,018-2,642,517)	9	2,752,269(1,876,738-3,827,984)	9	-0.99(-1.12,-0.87)	2.91(2.75,3.06)	2.21(1.77,2.64)	1.35(1.20,1.50)	9
60-64 years	1,834,164(1,270,882-2,460,905)	10	2,531,612(1,791,112-3,396,847)	13	-0.86(-1.04,-0.67)	1.19(1.05,1.33)	2.89(2.76,3.02)	1.13(1.03,1.23)	13

65-69 years	1,508,907(1,058,074-2,192,414)	14	2,235,603(1,591,844-3,192,152)	14	0.14(-0.05,0.34)	0.30(0.18,0.42)	4.00(3.87,4.12)	1.35(1.25,1.44)	10
70-74 years	959,846(633,143-1,409,313)	15	1,613,827(1,118,873-2,276,647)	15	1.94(1.81,2.07)	0.95(0.88,1.02)	2.67(2.50,2.85)	1.79(1.71,1.87)	7
75-79 years	552,406(400,082-732,704)	16	1,009,465(741,137-1,330,429)	16	1.81(1.55,2.07)	2.16(2.12,2.20)	2.16(2.12,2.20)	2.09(2.00,2.18)	5
80-84 years	288,979(200,368-407,534)	17	680,834(496,481-909,843)	17	1.83(1.50,2.17)	4.11(3.97,4.25)	2.94(2.83,3.05)	3.00(2.88,3.12)	4
85-90 years	138,998(104,986-181,437)	18	414,310(322,086-522,486)	18	3.48(3.31,3.65)	4.12(3.91,4.33)	3.84(3.70,3.98)	3.82(3.71,3.93)	3
90-94 years	45,699(31,920-62,720)	19	185,966(134,226-246,636)	19	4.94(4.82,5.06)	3.95(3.40,4.50)	5.59(5.11,6.07)	4.88(4.62,5.13)	2
95+ years	11,691(6,734-17,512)	20	61,261(37,907-87,401)	20	5.80(5.46,6.15)	7.34(7.12,7.56)	4.82(4.31,5.34)	5.91(5.68,6.14)	1

sTable 2. The incident cases and rate of incidence attributable to six immune mediated inflammatory diseases across 20 age groups, and its temporal trends from 1990 to 2019

	1990		2019		1990-1999	2000-2009	2010-2019	1990-2019	
	N(95%CI)	Rank	N(95%CI)	Rank	AAPC(95%CI)	AAPC(95%CI)	AAPC(95%CI)	AAPC(95%CI)	Rank
**Rate**									
Asthma									
<5 years	1,607.51(1,021.25-2,432.93)	1	1,509.37(947.96-2,305.76)	1	-1.50(-1.73,-1.28)	0.62（-0.09,1.33)	0.57(-0.73,1.89)	-0.04(-0.54,0.46)	6
5- 9 years	950.58(479.63-1,598.96)	2	980.25(499.97-1,654.75)	2	-1.18(-1.41,-0.94)	1.03（0.64,1.42)	0.97(0.01,1.94)	0.27(-0.07,0.61)	3
10-14 years	583.93(265.12-932.45)	9	586.95(268.78-934.63)	5	-0.79(-1.04,-0.55)	-0.37（-0.43,-0.31)	1.27(0.89,1.65)	0.01(-0.14,0.15)	5
15-19 years	417.55(244.95-639.20)	13	387.86(227.90-590.65)	12	-0.31(-0.42,-0.20)	-1.17（-1.32,-1.02)	0.76(0.44,1.09)	-0.23(-0.35,-0.11)	8
20-24 years	306.06(148.42-522.84)	15	274.17(132.12-468.66)	14	-0.24(-0.28,-0.20)	-1.30（-1.35,-1.25)	0.56(0.43,0.69)	-0.36(-0.41,-0.32)	9
25-29 years	239.97(133.00-369.01)	19	211.70(120.50-323.89)	18	-0.90(-0.97,-0.83)	-0.58（-0.75,-0.40)	0.45(0.22,0.69)	-0.38(-0.48,-0.28)	10
30-34 years	222.69(115.69-356.70)	20	188.29(99.92-299.11)	20	-1.99(-2.12,-1.86)	-0.49（-0.84,-0.14)	0.70(0.41,0.98)	-0.62(-0.77,-0.47)	12
35-39 years	252.03(126.00-434.76)	18	210.97(110.31-362.04)	19	-1.71(-1.88,-1.55)	-1.13（-1.24,-1.02)	1.19(1.03,1.36)	-0.61(-0.70,-0.51)	11
40-44 years	284.14(145.83-476.35)	17	221.85(114.99-368.57)	17	-2.03(-2.07,-1.98)	-1.76（-2.02,-1.50)	1.33(1.06,1.60)	-0.87(-1.02,-0.72)	14
45-49 years	303.72(130.77-544.86)	16	223.57(99.62-397.07)	16	-2.07(-2.42,-1.73)	-1.26（-1.36,-1.16)	0.50(0.26,0.75)	-1.05(-1.20,-0.90)	15
50-54 years	409.74(235.01-627.15)	14	260.63(140.55-412.11)	15	-2.95(-3.06,-2.84)	-1.71（-1.89,-1.52)	-0.18(-0.88,0.53)	-1.61(-1.85,-1.37)	17
55-59 years	591.39(302.82-944.42)	8	346.10(172.62-568.07)	13	-3.49(-3.60,-3.39)	-2.07（-2.41,-1.73)	0.27(0.10,0.44)	-1.81(-1.94,-1.68)	18
60-64 years	726.61(444.61-1,047.26)	5	410.67(241.23-619.51)	10	-3.65(-3.74,-3.56)	-2.06（-2.18,-1.95)	-0.06(-0.29,0.17)	-1.94(-2.02,-1.85)	19
65-69 years	791.77(497.08-1,269.87)	3	454.17(273.54-755.50)	8	-3.21(-3.29,-3.12)	-2.04（-2.16,-1.92)	-0.60(-1.24,0.05)	-1.95(-2.16,-1.74)	20
70-74 years	716.71(398.55-1,175.83)	6	458.23(261.23-743.81)	7	-2.38(-2.61,-2.16)	-1.99（-2.30,-1.67)	-0.32(-0.47,-0.17)	-1.57(-1.71,-1.43)	16
75-79 years	504.12(319.58-728.77)	11	410.24(260.54-597.06)	11	-0.96(-1.05,-0.87)	-1.27（-1.31,-1.23)	0.12(-0.04,0.28)	-0.73(-0.79,-0.67)	13
80-84 years	438.90(253.58-704.25)	12	431.59(276.28-634.23)	9	0.28(0.10,0.46)	-0.55（-0.69,-0.41)	-0.05(-0.29,0.19)	-0.11(-0.23,0.01)	7
85-90 years	533.52(366.68-747.94)	10	563.78(409.17-747.50)	6	-0.08(-0.41,0.25)	0.77（0.47,1.07)	-0.14(-0.22,-0.06)	0.15(-0.01,0.30)	4
90-94 years	640.76(390.77-956.91)	7	705.40(462.52-996.72)	4	-0.26(-0.35,-0.16)	1.75（1.62,1.88)	-0.27(-0.52,-0.03)	0.34(0.25,0.44)	2
95+ years	740.63(366.16-1,185.69)	4	872.73(498.76-1,291.67)	3	-0.46(-0.78,-0.14)	2.78（1.81,3.76)	-0.45(-0.71,-0.19)	0.54(0.21,0.86)	1
Inflammatory bowel disease									
<5 years	0.02(0.01-0.03)	20	0.02(0.02-0.03)	20	0.73(0.48,0.97)	-0.03(-0.06,0.00)	-0.22(-0.31,-0.13)	0.15(0.06,0.23)	1
5- 9 years	0.29(0.22-0.37)	19	0.29(0.22-0.38)	19	1.47(1.34,1.61)	-0.31(-0.33,-0.29)	-0.98(-1.04,-0.93)	0.03(-0.02,0.08)	4
10-14 years	1.04(0.84-1.31)	18	1.07(0.87-1.34)	18	0.96(0.89,1.04)	0.08(-0.03,0.19)	-0.84(-0.96,-0.72)	0.08(0.01,0.15)	3
15-19 years	2.60(2.09-3.20)	17	2.70(2.20-3.27)	17	1.44(1.34,1.55)	-0.22(-0.40,-0.03)	-0.81(-0.91,-0.72)	0.10(0.02,0.19)	2
20-24 years	4.85(3.96-5.92)	16	4.82(4.02-5.76)	16	0.85(0.49,1.21)	-0.53(-0.78,-0.28)	-0.29(-0.40,-0.18)	0.01(-0.14,0.16)	5
25-29 years	7.46(6.00-9.21)	15	6.55(5.33-7.94)	15	-0.72(-0.89,-0.55)	-0.21(-0.41,-0.01)	-0.47(-0.54,-0.41)	-0.47(-0.56,-0.38)	6
30-34 years	9.51(7.43-11.79)	14	7.44(5.88-9.10)	14	-2.30(-2.74,-1.85)	0.00(-0.08,0.08)	-0.46(-0.67,-0.25)	-0.86(-1.02,-0.70)	11
35-39 years	10.12(7.97-12.58)	5	7.94(6.40-9.70)	13	-1.83(-2.13,-1.53)	-0.44(-0.95,0.08)	0.04(-0.03,0.10)	-0.80(-0.99,-0.60)	10
40-44 years	10.98(8.58-13.70)	2	7.98(6.34-9.88)	12	-2.65(-2.78,-2.52)	-0.73(-1.01,-0.44)	-0.13(-0.20,-0.07)	-1.12(-1.24,-1.00)	19
45-49 years	10.49(8.17-13.10)	4	8.00(6.36-9.91)	11	-2.14(-2.55,-1.74)	0.13(-0.18,0.45)	-0.59(-0.71,-0.47)	-0.95(-1.13,-0.77)	15
50-54 years	10.11(8.04-12.78)	6	7.92(6.36-9.89)	10	-1.83(-1.96,-1.70)	-0.18(-0.36,-0.01)	-0.83(-0.97,-0.70)	-0.88(-0.98,-0.78)	13
55-59 years	9.83(7.75-12.37)	9	8.18(6.57-10.18)	9	-1.90(-2.09,-1.72)	0.28(-0.19,0.75)	0.24(0.03,0.45)	-0.52(-0.72,-0.33)	7

60-64 years	10.01(7.75-12.65)	7	8.09(6.36-10.13)	8	-2.68(-2.91,-2.44)	0.51(-0.29,1.32)	-0.22(-0.42,-0.03)	-0.75(-1.02,-0.48)	9
65-69 years	9.96(7.75-12.67)	8	7.77(6.07-9.82)	7	-3.03(-3.12,-2.93)	0.50(0.45,0.55)	0.08(-0.11,0.27)	-0.86(-0.93,-0.79)	12
70-74 years	9.57(7.27-12.40)	12	7.72(5.98-9.93)	6	-2.30(-2.62,-1.98)	-0.41(-0.57,-0.26)	0.45(0.39,0.51)	-0.73(-0.85,-0.61)	8
75-79 years	9.68(7.45-12.45)	11	7.47(5.73-9.75)	5	-2.13(-2.31,-1.95)	-0.76(-0.82,-0.70)	0.21(0.13,0.29)	-0.89(-0.95,-0.82)	14
80-84 years	9.55(7.33-12.25)	13	7.26(5.52-9.42)	4	-1.81(-2.09,-1.52)	-0.12(-0.35,0.10)	-0.93(-1.01,-0.84)	-1.01(-1.14,-0.88)	17
85-90 years	9.80(7.48-12.74)	10	7.30(5.55-9.56)	3	-1.45(-1.53,-1.37)	-0.28(-0.39,-0.17)	-1.29(-1.42,-1.17)	-0.99(-1.06,-0.92)	16
90-94 years	10.83(8.04-14.19)	3	8.00(5.91-10.57)	2	-2.00(-2.14,-1.87)	-0.19(-0.22,-0.16)	-1.11(-1.18,-1.04)	-1.04(-1.09,-0.99)	18
95+ years	12.42(8.74-16.88)	1	8.99(6.31-12.48)	1	-1.78(-1.98,-1.58)	-0.33(-0.40,-0.27)	-1.07(-1.19,-0.94)	-1.12(-1.20,-1.03)	20
Multiple sclerosis									
<5 years	0.00(0.00-0.00)	20	0.00(0.00-0.00)	20					
5- 9 years	0.05(0.02-0.10)	19	0.05(0.02-0.10)	19	-0.14(-0.19,-0.10)	0.01(-0.01,0.02)	0.11(0.05,0.17)	0.00(-0.02,0.03)	3
10-14 years	0.24(0.13-0.40)	18	0.24(0.13-0.38)	18	-0.62(-0.73,-0.50)	0.14(0.13,0.16)	0.22(0.17,0.28)	-0.07(-0.11,-0.03)	5
15-19 years	0.59(0.38-0.89)	8	0.60(0.39-0.88)	8	0.41(0.36,0.47)	-0.18(-0.28,-0.08)	-0.01(-0.06,0.05)	0.06(0.02,0.11)	2
20-24 years	1.16(0.71-1.82)	5	1.22(0.78-1.86)	4	0.56(0.39,0.73)	-0.27(-0.43,-0.10)	0.21(0.11,0.31)	0.20(0.11,0.29)	1
25-29 years	1.71(1.14-2.35)	2	1.68(1.13-2.27)	1	-0.57(-0.75,-0.39)	0.58(0.52,0.64)	-0.15(-0.38,0.07)	-0.05(-0.14,0.05)	4
30-34 years	1.84(1.14-2.61)	1	1.66(1.04-2.30)	2	-1.60(-1.74,-1.47)	0.35(0.21,0.49)	-0.13(-0.31,0.04)	-0.39(-0.49,-0.30)	15
35-39 years	1.61(1.01-2.21)	3	1.50(0.96-2.05)	3	-0.54(-0.73,-0.35)	-0.62(-0.88,-0.35)	0.77(0.63,0.91)	-0.16(-0.29,-0.03)	6
40-44 years	1.41(0.83-2.01)	4	1.19(0.70-1.68)	5	-1.31(-1.48,-1.15)	-1.09(-1.24,-0.94)	0.29(0.20,0.39)	-0.64(-0.73,-0.56)	17
45-49 years	1.14(0.75-1.64)	6	0.93(0.60-1.35)	6	-1.20(-1.30,-1.10)	-0.22(-0.47,0.04)	-0.54(-0.72,-0.35)	-0.70(-0.81,-0.59)	18
50-54 years	0.87(0.48-1.37)	7	0.68(0.37-1.10)	7	-1.38(-1.51,-1.25)	-0.51(-0.61,-0.41)	-0.82(-0.96,-0.68)	-0.86(-0.95,-0.77)	19
55-59 years	0.51(0.35-0.69)	9	0.44(0.30-0.60)	9	-0.82(-0.93,-0.72)	-0.60(-0.71,-0.48)	0.04(-0.37,0.45)	-0.48(-0.62,-0.34)	16
60-64 years	0.33(0.22-0.43)	17	0.31(0.19-0.41)	17	-0.50(-0.50,-0.49)	-0.36(-0.37,-0.35)	-0.05(-0.09,-0.01)	-0.30(-0.32,-0.29)	12
65-69 years	0.34(0.24-0.43)	16	0.32(0.21-0.40)	16	-0.45(-0.49,-0.41)	-0.32(-0.54,-0.10)	-0.22(-0.27,-0.17)	-0.33(-0.40,-0.26)	14
70-74 years	0.35(0.23-0.44)	15	0.32(0.21-0.42)	15	-0.40(-0.49,-0.31)	-0.13(-0.17,-0.09)	-0.43(-0.46,-0.40)	-0.32(-0.35,-0.29)	13
75-79 years	0.36(0.26-0.44)	14	0.33(0.24-0.42)	14	-0.43(-0.54,-0.31)	-0.08(-0.10,-0.05)	-0.34(-0.39,-0.28)	-0.27(-0.31,-0.23)	11
80-84 years	0.36(0.28-0.43)	13	0.34(0.26-0.41)	13	-0.40(-0.47,-0.33)	-0.07(-0.08,-0.06)	-0.17(-0.21,-0.14)	-0.20(-0.23,-0.18)	9
85-90 years	0.36(0.27-0.44)	11	0.34(0.26-0.42)	10	-0.40(-0.48,-0.33)	-0.06(-0.10,-0.03)	-0.11(-0.13,-0.10)	-0.18(-0.21,-0.16)	7
90-94 years	0.36(0.24-0.45)	12	0.34(0.23-0.43)	12	-0.40(-0.46,-0.34)	0.02(-0.02,0.05)	-0.15(-0.18,-0.12)	-0.19(-0.22,-0.16)	8
95+ years	0.36(0.21-0.46)	10	0.34(0.20-0.44)	11	-0.51(-0.55,-0.48)	-0.07(-0.12,-0.02)	-0.04(-0.10,0.03)	-0.21(-0.24,-0.18)	10
Rheumatoid arthritis									
<5 years	0.00(0.00-0.00)	20	0.00(0.00-0.00)	20					
5- 9 years	1.06(0.62-1.66)	19	1.07(0.63-1.69)	19	0.18(-0.07,0.43)	0.19(0.14,0.23)	-0.34(-0.47,-0.21)	0.02(-0.07,0.11)	15
10-14 years	3.15(2.35-4.06)	17	3.30(2.46-4.28)	17	0.25(0.13,0.36)	0.29(0.23,0.35)	-0.17(-0.28,-0.06)	0.14(0.08,0.20)	14
15-19 years	5.17(3.64-7.27)	16	5.62(3.96-7.93)	16	0.39(0.38,0.40)	0.39(0.38,0.40)	-0.01(-0.09,0.07)	0.27(0.24,0.29)	2
20-24 years	6.76(5.12-8.75)	14	7.45(5.64-9.71)	14	0.46(0.44,0.48)	0.46(0.44,0.48)	0.02(-0.09,0.13)	0.32(0.29,0.36)	1
25-29 years	8.02(5.39-10.74)	13	8.68(5.81-11.62)	13	0.25(0.12,0.38)	0.49(0.45,0.53)	-0.06(-0.20,0.08)	0.25(0.18,0.31)	3
30-34 years	10.27(7.77-13.16)	12	11.00(8.25-14.19)	11	0.22(0.16,0.28)	0.40(0.34,0.46)	0.00(-0.15,0.14)	0.22(0.16,0.28)	6
35-39 years	13.55(9.42-18.14)	10	14.46(9.99-19.45)	10	0.30(0.28,0.32)	0.30(0.28,0.32)	0.10(-0.23,0.43)	0.24(0.14,0.34)	4

40-44 years	17.67(13.43-22.29)	8	18.70(14.06-23.79)	8	0.17(0.13,0.22)	0.43(0.42,0.45)	-0.09(-0.25,0.07)	0.19(0.14,0.24)	10
45-49 years	22.40(16.08-30.31)	6	23.72(16.86-32.07)	6	0.23(0.20,0.26)	0.45(0.43,0.47)	-0.16(-0.31,0.00)	0.19(0.14,0.24)	11
50-54 years	26.36(20.29-32.96)	5	28.22(21.55-35.61)	5	0.65(0.54,0.76)	0.28(0.23,0.33)	-0.28(-0.45,-0.11)	0.22(0.15,0.29)	7
55-59 years	30.57(21.49-40.51)	4	32.23(22.56-42.97)	4	0.41(0.16,0.65)	0.32(0.22,0.42)	-0.43(-0.63,-0.23)	0.15(0.02,0.27)	13
60-64 years	34.65(25.73-44.38)	2	37.22(27.60-48.08)	2	0.48(0.38,0.58)	0.72(0.50,0.94)	-0.55(-0.76,-0.35)	0.21(0.10,0.32)	8
65-69 years	35.81(26.48-46.01)	1	38.38(28.25-49.26)	1	0.07(-0.04,0.18)	0.75(0.66,0.83)	-0.23(-0.31,-0.16)	0.24(0.18,0.30)	5
70-74 years	30.63(21.89-40.64)	3	32.24(22.70-43.00)	3	-0.60(-0.66,-0.54)	0.46(0.42,0.50)	0.59(0.41,0.77)	0.17(0.11,0.23)	12
75-79 years	21.21(17.46-25.11)	7	22.50(18.38-26.93)	7	0.12(-0.05,0.28)	-0.09(-0.16,-0.01)	0.68(0.46,0.90)	0.21(0.11,0.31)	9
80-84 years	14.86(11.48-18.78)	9	14.50(11.07-18.57)	9	0.20(-0.01,0.42)	-0.68(-0.85,-0.50)	0.02(-0.05,0.09)	-0.11(-0.21,-0.01)	16
85-90 years	10.60(8.20-13.42)	11	10.18(7.78-13.00)	12	0.25(0.23,0.28)	-0.29(-0.33,-0.25)	-0.33(-0.45,-0.22)	-0.12(-0.16,-0.08)	17
90-94 years	6.38(4.96-8.02)	15	6.01(4.60-7.64)	15	0.32(0.16,0.48)	-0.06(-0.22,0.10)	-0.81(-1.33,-0.29)	-0.16(-0.34,0.02)	18
95+ years	1.65(1.28-2.06)	18	1.54(1.18-1.96)	18	0.40(0.22,0.59)	-0.09(-0.19,0.02)	-1.10(-1.79,-0.42)	-0.25(-0.48,-0.02)	19
Psoriasis									
<5 years	34.84(31.48-38.02)	17	29.55(26.70-32.21)	18	-0.38(-0.44,-0.31)	-0.76(-0.80,-0.73)	-0.58(-0.64,-0.51)	-0.57(-0.61,-0.53)	6
5- 9 years	41.77(36.96-46.51)	16	34.35(30.24-38.30)	16	-0.43(-0.50,-0.36)	-0.91(-0.96,-0.87)	-0.68(-0.71,-0.65)	-0.67(-0.69,-0.64)	8
10-14 years	43.28(37.70-49.12)	15	35.08(30.47-39.87)	15	-0.66(-0.67,-0.64)	-0.76(-0.78,-0.75)	-0.76(-0.86,-0.65)	-0.73(-0.76,-0.69)	11
15-19 years	47.17(40.70-53.59)	13	37.05(32.05-42.21)	14	-0.86(-0.87,-0.84)	-0.73(-0.76,-0.70)	-0.88(-0.93,-0.83)	-0.83(-0.85,-0.81)	15
20-24 years	55.65(48.06-64.05)	12	43.94(37.84-50.72)	12	-0.94(-0.99,-0.89)	-0.79(-0.83,-0.75)	-0.77(-0.89,-0.64)	-0.83(-0.87,-0.78)	16
25-29 years	69.30(60.66-79.44)	10	54.34(47.54-62.35)	11	-1.01(-1.03,-0.99)	-0.75(-0.82,-0.69)	-0.74(-0.81,-0.67)	-0.84(-0.87,-0.81)	17
30-34 years	80.43(70.17-91.36)	9	62.80(54.78-71.26)	9	-0.82(-0.90,-0.74)	-0.90(-0.93,-0.87)	-0.84(-0.86,-0.81)	-0.86(-0.88,-0.83)	18
35-39 years	91.70(80.32-103.81)	7	71.16(62.34-80.60)	8	-0.70(-0.72,-0.67)	-0.90(-0.92,-0.88)	-1.03(-1.05,-1.00)	-0.87(-0.89,-0.86)	19
40-44 years	102.02(89.02-115.17)	6	78.56(68.63-88.39)	6	-0.82(-0.84,-0.81)	-0.67(-0.70,-0.64)	-1.19(-1.23,-1.16)	-0.90(-0.92,-0.88)	20
45-49 years	108.76(94.97-123.49)	5	86.07(74.95-97.85)	5	-0.60(-0.65,-0.55)	-0.75(-0.77,-0.74)	-1.01(-1.04,-0.99)	-0.80(-0.82,-0.78)	14
50-54 years	119.86(104.84-136.59)	3	96.15(83.87-109.88)	3	-0.43(-0.47,-0.40)	-0.96(-1.03,-0.89)	-0.89(-0.96,-0.82)	-0.75(-0.78,-0.71)	13
55-59 years	121.89(107.16-137.98)	1	99.75(87.83-112.77)	1	-0.61(-0.64,-0.57)	-0.71(-0.83,-0.59)	-0.67(-0.71,-0.63)	-0.68(-0.72,-0.64)	9
60-64 years	120.11(104.76-136.12)	2	97.42(85.18-110.67)	2	-0.87(-0.92,-0.83)	-0.48(-0.51,-0.45)	-0.85(-0.98,-0.72)	-0.72(-0.76,-0.67)	10
65-69 years	113.68(98.82-128.42)	4	91.66(80.00-103.59)	4	-1.04(-1.09,-1.00)	-0.58(-0.61,-0.55)	-0.64(-0.72,-0.56)	-0.73(-0.76,-0.70)	12
70-74 years	88.44(76.28-100.79)	8	75.90(65.40-86.36)	7	-0.55(-0.65,-0.45)	-0.59(-0.62,-0.56)	-0.43(-0.48,-0.38)	-0.53(-0.56,-0.49)	5
75-79 years	65.20(55.78-75.23)	11	55.29(47.52-63.66)	10	-0.55(-0.61,-0.49)	-0.65(-0.68,-0.63)	-0.50(-0.52,-0.48)	-0.57(-0.59,-0.55)	7
80-84 years	46.18(39.28-53.56)	14	39.81(33.89-46.33)	13	-0.93(-1.22,-0.64)	-0.19(-0.38,0.00)	-0.57(-0.61,-0.52)	-0.52(-0.65,-0.40)	4
85-90 years	33.69(28.53-39.40)	18	29.82(25.19-34.73)	17	-0.22(-0.31,-0.14)	-0.36(-0.58,-0.14)	-0.66(-0.72,-0.60)	-0.45(-0.53,-0.37)	3
90-94 years	28.40(23.69-33.18)	19	25.75(21.52-30.06)	19	0.00(-0.03,0.04)	-0.66(-0.78,-0.55)	-0.40(-0.53,-0.27)	-0.31(-0.38,-0.25)	2
95+ years	26.67(21.41-32.57)	20	25.66(20.47-31.50)	20	0.05(-0.15,0.24)	0.18(0.11,0.25)	-0.53(-0.74,-0.31)	-0.12(-0.22,-0.01)	1
Atopic dermatitis									
<5 years	993.65(899.21-1,095.40)	1	957.45(867.48-1,054.47)	1	-0.14(-0.16,-0.12)	-0.20(-0.26,-0.15)	-0.04(-0.11,0.03)	-0.13(-0.16,-0.10)	14
5- 9 years	455.14(391.88-529.88)	2	445.21(383.15-516.97)	2	0.04(-0.03,0.11)	0.05(-0.08,0.18)	-0.39(-0.43,-0.35)	-0.10(-0.15,-0.05)	13
10-14 years	274.84(228.68-326.13)	9	271.69(225.04-320.76)	10	-0.03(-0.04,-0.03)	0.09(0.09,0.10)	-0.20(-0.22,-0.18)	-0.04(-0.05,-0.03)	10
15-19 years	249.82(209.21-293.15)	14	229.00(188.37-270.76)	14	-0.32(-0.34,-0.30)	-0.14(-0.15,-0.12)	-0.43(-0.45,-0.41)	-0.30(-0.31,-0.28)	17

20-24 years	265.86(224.65-310.26)	11	225.92(188.52-266.55)	16	-0.67(-0.69,-0.65)	-0.09(-0.13,-0.05)	-1.03(-1.07,-0.99)	-0.57(-0.59,-0.55)	20
25-29 years	256.51(213.25-301.01)	12	227.80(187.04-266.80)	15	-0.20(-0.33,-0.06)	-0.81(-0.86,-0.77)	-0.26(-0.44,-0.08)	-0.41(-0.49,-0.33)	19
30-34 years	229.13(189.61-269.79)	17	215.19(177.42-254.28)	18	0.74(0.60,0.88)	-0.95(-1.03,-0.87)	-0.15(-0.11,0.02)	-0.18(-0.26,-0.11)	16
35-39 years	214.68(176.55-254.78)	18	194.40(158.52-232.09)	20	-0.12(-0.28,0.03)	-0.25(-0.54,0.04)	-0.91(-0.98,-0.84)	-0.39(-0.51,-0.27)	18
40-44 years	207.69(175.18-242.42)	20	198.06(165.85-231.66)	19	0.18(0.05,0.31)	0.40(0.27,0.52)	-0.82(-0.89,-0.76)	-0.13(-0.21,-0.06)	15
45-49 years	210.77(172.73-246.00)	19	215.53(175.85-252.98)	17	0.52(0.48,0.56)	-0.18(-0.26,-0.09)	-0.22(-0.28,-0.15)	0.09(0.05,0.13)	3
50-54 years	232.47(193.21-275.86)	16	247.25(205.62-292.17)	13	0.44(0.40,0.48)	0.03(-0.04,0.11)	0.38(0.10,0.65)	0.25(0.16,0.35)	1
55-59 years	248.49(209.79-289.23)	15	255.13(215.96-297.18)	12	-0.05(-0.12,0.03)	0.34(0.31,0.37)	-0.14(-0.33,0.04)	0.07(0.00,0.13)	6
60-64 years	250.04(208.05-291.06)	13	256.32(212.54-298.08)	11	0.11(0.06,0.17)	0.17(0.13,0.20)	0.02(-0.07,0.11)	0.09(0.05,0.13)	4
65-69 years	270.34(226.45-318.00)	10	272.26(227.52-315.91)	9	-0.12(-0.16,-0.07)	-0.12(-0.17,-0.08)	0.27(0.25,0.29)	0.03(0.00,0.05)	8
70-74 years	290.02(244.94-337.46)	8	288.20(242.54-333.35)	8	-0.09(-0.11,-0.06)	-0.16(-0.24,-0.08)	0.16(0.08,0.24)	-0.04(-0.10,0.01)	11
75-79 years	300.45(252.04-353.10)	7	298.69(250.92-349.33)	7	0.23(0.18,0.28)	-0.16(-0.29,-0.04)	-0.23(-0.28,-0.17)	-0.04(-0.09,0.01)	12
80-84 years	310.66(256.96-367.85)	6	312.96(261.07-368.77)	6	0.29(0.27,0.31)	-0.08(-0.12,-0.04)	-0.10(-0.19,0.00)	0.03(0.00,0.07)	9
85-90 years	334.45(285.56-390.13)	5	341.43(292.81-396.44)	5	0.33(0.31,0.35)	0.16(0.10,0.22)	-0.22(-0.29,-0.14)	0.08(0.04,0.11)	5
90-94 years	350.47(296.76-410.77)	4	357.66(301.46-417.64)	4	0.23(0.19,0.28)	0.27(0.24,0.31)	-0.33(-0.38,-0.27)	0.07(0.04,0.10)	7
95+ years	353.92(256.31-463.40)	3	374.17(267.26-493.01)	3	0.22(0.19,0.26)	0.45(0.23,0.67)	-0.06(-0.10,-0.03)	0.20(0.13,0.27)	2
**Number of incident cases**									
Asthma									
<5 years	10,161,359(6,455,506-15,378,937)	1	10,004,724(6,283,513-15,283,571)	1	-1.57(-1.84,-1.31)	1.01(-0.32,2.34)	0.82(0.30,1.34)	0.04(-0.41,0.50)	20
5- 9 years	5,562,422(2,806,587-9,356,499)	2	6,417,739(3,273,311-10,833,682)	2	-0.84(-0.99,-0.68)	1.06(0.67,1.45)	1.50(0.70,2.30)	0.59(0.30,0.88)	14
10-14 years	3,133,916(1,422,898-5,004,360)	3	3,769,324(1,726,065-6,002,079)	3	1.06(0.87,1.25)	-0.87(-1.17,-0.57)	1.88(1.23,2.54)	0.71(0.47,0.95)	12
15-19 years	2,169,583(1,272,772-3,321,297)	4	2,402,948(1,411,948-3,659,320)	4	0.45(0.10,0.80)	-0.13(-0.29,0.02)	0.58(0.32,0.85)	0.29(0.13,0.46)	18
20-24 years	1,507,890(731,246-2,575,884)	5	1,645,434(792,918-2,812,667)	5	0.06(0.02,0.11)	0.59(0.55,0.62)	0.14(0.03,0.24)	0.28(0.25,0.32)	19
25-29 years	1,062,690(588,987-1,634,115)	8	1,281,779(729,569-1,961,082)	8	0.28(0.21,0.35)	0.32(0.27,0.38)	1.43(1.34,1.53)	0.65(0.60,0.69)	13
30-34 years	858,763(446,120-1,375,539)	12	1,133,008(601,239-1,799,833)	12	0.55(0.46,0.64)	0.01(-0.03,0.06)	2.46(2.34,2.58)	0.97(0.92,1.03)	9
35-39 years	889,031(444,438-1,533,570)	10	1,141,320(596,745-1,958,526)	10	0.20(0.16,0.24)	0.31(0.24,0.37)	2.21(1.89,2.52)	0.86(0.76,0.96)	11
40-44 years	813,465(417,495-1,363,757)	13	1,094,700(567,432-1,818,707)	13	0.63(0.40,0.86)	0.76(0.68,0.83)	1.76(1.55,1.97)	0.98(0.87,1.10)	8
45-49 years	705,875(303,915-1,266,307)	14	1,059,301(472,020-1,881,306)	14	1.68(1.48,1.88)	0.66(0.54,0.77)	2.17(1.92,2.43)	1.45(1.33,1.57)	6
50-54 years	871,060(499,617-1,333,259)	11	1,138,467(613,965-1,800,162)	11	-1.08(-1.20,-0.95)	1.22(1.15,1.28)	2.48(2.35,2.60)	0.89(0.82,0.95)	10
55-59 years	1,096,537(561,477-1,751,094)	7	1,284,061(640,453-2,107,612)	6	-2.38(-2.72,-2.04)	1.66(1.55,1.78)	2.73(2.18,3.28)	0.57(0.34,0.80)	15
60-64 years	1,167,255(714,242-1,682,343)	6	1,283,484(753,916-1,936,200)	7	-2.14(-2.24,-2.05)	0.19(-0.43,0.81)	2.77(2.57,2.97)	0.30(0.08,0.52)	17
65-69 years	977,746(613,841-1,568,146)	9	1,174,412(707,333-1,953,588)	9	-0.81(-1.09,-0.53)	-0.64(-0.92,-0.37)	3.50(3.28,3.72)	0.56(0.40,0.72)	16
70-74 years	605,720(336,830-993,737)	15	857,280(488,723-1,391,581)	15	1.07(0.95,1.19)	0.17(0.11,0.24)	2.60(2.36,2.84)	1.22(1.13,1.31)	7
75-79 years	309,070(195,933-446,803)	16	521,221(331,020-758,592)	16	1.36(1.02,1.70)	1.65(1.41,1.88)	2.17(2.06,2.29)	1.77(1.63,1.92)	5
80-84 years	154,580(89,310-248,034)	17	364,360(233,245-535,433)	17	1.91(1.57,2.25)	3.77(3.69,3.84)	2.98(2.84,3.13)	2.95(2.82,3.07)	4
85-90 years	80,395(55,254-112,705)	18	245,135(177,910-325,019)	18	3.42(3.20,3.63)	4.27(4.12,4.42)	3.94(3.80,4.08)	3.89(3.78,4.00)	3
90-94 years	28,232(17,217-42,161)	19	118,914(77,970-168,024)	19	4.70(4.50,4.91)	4.65(3.83,5.47)	5.61(5.40,5.81)	5.00(4.72,5.27)	2
95+ years	7,624(3,769-12,206)	20	41,657(23,807-61,654)	20	5.51(5.08,5.95)	7.84(7.49,8.19)	5.03(4.42,5.64)	6.10(5.81,6.38)	1

Inflammatory bowel disease									
<5 years	143(93-205)	19	157(103-225)	20	0.51(0.42,0.61)	0.37(0.14,0.59)	0.03(-0.10,0.16)	0.32(0.23,0.42)	20
5- 9 years	1,691(1,306-2,157)	16	1,896(1,450-2,456)	17	1.90(1.84,1.96)	-0.16(-0.23,-0.09)	-0.47(-0.57,-0.36)	0.38(0.33,0.43)	19
10-14 years	5,578(4,492-7,012)	14	6,888(5,571-8,607)	14	2.71(2.62,2.79)	-0.36(-0.74,0.01)	-0.27(-0.35,-0.20)	0.72(0.59,0.84)	13
15-19 years	13,485(10,856-16,615)	10	16,718(13,620-20,286)	11	2.33(2.14,2.53)	0.57(0.44,0.70)	-0.78(-0.84,-0.72)	0.72(0.64,0.80)	14
20-24 years	23,877(19,515-29,145)	6	28,911(24,106-34,576)	8	1.27(1.01,1.53)	1.19(1.15,1.24)	-0.69(-0.78,-0.60)	0.63(0.54,0.72)	16
25-29 years	33,043(26,576-40,798)	3	39,642(32,289-48,092)	3	0.56(0.45,0.67)	0.71(0.63,0.80)	0.55(0.41,0.70)	0.61(0.54,0.68)	17
30-34 years	36,680(28,641-45,452)	1	44,770(35,357-54,787)	1	0.26(0.03,0.49)	0.43(0.34,0.52)	1.33(1.26,1.40)	0.68(0.60,0.77)	15
35-39 years	35,702(28,112-44,369)	2	42,937(34,623-52,499)	2	-0.01(-0.17,0.16)	1.06(0.85,1.28)	0.77(0.41,1.12)	0.57(0.42,0.72)	18
40-44 years	31,426(24,576-39,230)	4	39,392(31,307-48,740)	4	0.13(-0.29,0.56)	1.80(1.66,1.93)	0.33(0.17,0.49)	0.75(0.60,0.90)	12
45-49 years	24,370(18,979-30,454)	5	37,906(30,116-46,956)	5	1.56(1.12,2.01)	2.22(2.07,2.38)	1.20(0.97,1.43)	1.61(1.42,1.80)	10
50-54 years	21,496(17,083-27,172)	7	34,583(27,777-43,204)	6	0.07(-0.12,0.26)	2.61(2.53,2.70)	1.94(1.76,2.12)	1.65(1.54,1.77)	8
55-59 years	18,230(14,375-22,927)	8	30,351(24,378-37,753)	7	-0.78(-0.91,-0.64)	4.03(3.65,4.40)	2.30(2.11,2.49)	1.77(1.61,1.93)	6
60-64 years	16,079(12,455-20,327)	9	25,296(19,874-31,648)	9	-1.23(-1.35,-1.12)	2.69(2.55,2.82)	2.98(2.87,3.09)	1.60(1.52,1.68)	11
65-69 years	12,299(9,572-15,650)	11	20,101(15,698-25,394)	10	-0.91(-1.15,-0.67)	1.89(1.65,2.13)	4.27(3.55,4.99)	1.64(1.39,1.89)	9
70-74 years	8,089(6,142-10,479)	12	14,438(11,180-18,586)	12	1.33(1.00,1.67)	1.52(1.37,1.67)	3.34(2.91,3.76)	2.02(1.82,2.21)	5
75-79 years	5,937(4,569-7,634)	13	9,487(7,278-12,383)	13	0.18(-0.06,0.42)	2.19(1.89,2.48)	2.58(2.41,2.75)	1.66(1.51,1.82)	7
80-84 years	3,363(2,583-4,316)	15	6,126(4,658-7,950)	15	-0.49(-1.03,0.06)	4.15(3.92,4.38)	2.17(2.02,2.31)	2.03(1.83,2.22)	4
85-90 years	1,477(1,126-1,920)	17	3,175(2,412-4,155)	16	1.93(1.65,2.22)	3.40(3.15,3.65)	2.82(2.59,3.05)	2.74(2.58,2.91)	3
90-94 years	477(354-625)	18	1,349(996-1,781)	18	3.41(2.79,4.04)	2.72(2.56,2.88)	4.64(3.96,5.33)	3.64(3.33,3.95)	2
95+ years	128(90-174)	20	429(301-596)	19	4.04(3.24,4.85)	4.82(4.34,5.31)	3.85(3.56,4.14)	4.16(3.80,4.52)	1
Multiple sclerosis									
<5 years	0(0-0)	20	0(0-0)	20					
5- 9 years	277(102-573)	14	310(114-642)	15	0.37(0.28,0.46)	0.17(0.11,0.22)	0.64(0.50,0.78)	0.38(0.32,0.44)	19
10-14 years	1,296(685-2,135)	9	1,524(826-2,462)	10	1.08(1.03,1.12)	-0.20(-0.28,-0.12)	0.78(0.69,0.88)	0.57(0.52,0.62)	18
15-19 years	3,087(1,954-4,626)	6	3,720(2,439-5,428)	7	1.11(0.84,1.39)	0.77(0.67,0.86)	0.07(-0.13,0.27)	0.65(0.53,0.77)	17
20-24 years	5,702(3,493-8,985)	3	7,316(4,671-11,144)	4	0.92(0.82,1.03)	1.54(1.45,1.63)	-0.15(-0.24,-0.05)	0.82(0.76,0.89)	16
25-29 years	7,560(5,042-10,406)	1	10,186(6,816-13,757)	1	0.80(0.72,0.89)	1.64(1.36,1.92)	0.78(0.48,1.09)	1.07(0.94,1.21)	15
30-34 years	7,110(4,404-10,066)	2	10,005(6,266-13,848)	2	0.95(0.85,1.06)	0.89(0.85,0.93)	1.65(1.53,1.78)	1.19(1.13,1.25)	14
35-39 years	5,674(3,560-7,799)	4	8,116(5,212-11,099)	3	1.41(1.22,1.59)	0.84(0.79,0.88)	1.62(1.53,1.72)	1.26(1.19,1.32)	12
40-44 years	4,045(2,373-5,744)	5	5,857(3,447-8,268)	5	1.35(1.26,1.45)	1.50(1.38,1.63)	0.86(0.81,0.91)	1.25(1.19,1.31)	13
45-49 years	2,647(1,735-3,816)	7	4,394(2,846-6,398)	6	2.43(2.22,2.64)	1.93(1.89,1.96)	1.02(0.96,1.09)	1.80(1.73,1.87)	10
50-54 years	1,840(1,015-2,906)	8	2,978(1,605-4,790)	8	0.47(0.21,0.73)	2.38(2.27,2.49)	1.98(1.90,2.05)	1.69(1.57,1.81)	11
55-59 years	938(651-1,275)	10	1,637(1,116-2,240)	9	0.33(0.14,0.52)	3.32(3.09,3.55)	2.41(2.33,2.48)	1.93(1.82,2.04)	9
60-64 years	537(352-690)	11	957(609-1,273)	11	0.82(0.75,0.89)	2.03(1.88,2.17)	3.00(2.46,3.53)	1.99(1.80,2.18)	8
65-69 years	426(292-525)	12	815(543-1,037)	12	1.92(1.57,2.26)	1.25(1.15,1.35)	4.00(3.75,4.26)	2.31(2.16,2.45)	6
70-74 years	297(196-375)	13	603(387-795)	13	3.16(3.03,3.29)	1.96(1.88,2.04)	2.32(2.02,2.62)	2.44(2.34,2.55)	5
75-79 years	218(157-267)	15	419(299-530)	14	1.87(1.73,2.01)	2.95(2.90,3.01)	1.81(1.66,1.96)	2.27(2.19,2.34)	7

80-84 years	126(97-152)	16	286(220-350)	16	1.19(0.91,1.47)	4.43(4.31,4.55)	2.79(2.69,2.90)	2.85(2.75,2.95)	4
85-90 years	54(41-66)	17	149(112-181)	17	3.06(2.86,3.26)	3.45(3.23,3.67)	4.08(3.84,4.31)	3.51(3.37,3.65)	3
90-94 years	16(11-20)	18	57(39-72)	18	4.74(4.52,4.97)	3.01(2.65,3.38)	5.94(5.80,6.08)	4.57(4.43,4.72)	2
95+ years	4(2-5)	19	16(10-21)	19	5.49(5.40,5.57)	5.23(5.09,5.37)	5.07(4.73,5.41)	5.27(5.14,5.39)	1
Rheumatoid arthritis									
<5 years	0(0-0)	20	0(0-0)	20					
5- 9 years	6,185(3,629-9,718)	15	7,005(4,115-11,093)	16	0.73(0.62,0.83)	0.32(0.24,0.40)	0.20(0.06,0.34)	0.40(0.33,0.48)	19
10-14 years	16,925(12,632-21,811)	13	21,202(15,766-27,497)	14	1.86(1.62,2.10)	-0.05(-0.23,0.14)	0.43(0.35,0.51)	0.78(0.68,0.88)	18
15-19 years	26,867(18,936-37,774)	11	34,799(24,516-49,150)	12	1.34(1.21,1.47)	1.22(1.11,1.34)	0.09(-0.01,0.19)	0.89(0.82,0.97)	17
20-24 years	33,294(25,224-43,122)	10	44,708(33,845-58,270)	11	0.79(0.68,0.90)	2.38(2.25,2.51)	-0.38(-0.82,0.07)	0.97(0.82,1.12)	16
25-29 years	35,526(23,890-47,556)	9	52,549(35,159-70,334)	10	1.53(1.36,1.70)	1.45(1.38,1.52)	0.88(0.70,1.05)	1.31(1.23,1.40)	15
30-34 years	39,592(29,944-50,730)	8	66,206(49,617-85,382)	8	2.73(2.51,2.96)	0.88(0.62,1.14)	1.89(1.74,2.04)	1.82(1.68,1.97)	13
35-39 years	47,803(33,216-64,001)	6	78,205(54,040-105,229)	7	2.19(1.97,2.42)	1.72(1.42,2.03)	0.85(0.74,0.96)	1.63(1.49,1.77)	14
40-44 years	50,592(38,447-63,827)	5	92,256(69,396-117,397)	6	2.90(2.38,3.42)	3.19(2.77,3.61)	0.45(0.17,0.73)	2.09(1.83,2.36)	12
45-49 years	52,052(37,360-70,443)	4	112,381(79,898-151,949)	4	4.11(3.94,4.28)	2.42(2.11,2.73)	1.39(1.14,1.64)	2.72(2.57,2.87)	9
50-54 years	56,036(43,129-70,068)	2	123,281(94,137-155,551)	1	2.50(2.02,2.97)	3.27(3.03,3.50)	2.52(2.36,2.67)	2.82(2.60,3.04)	7
55-59 years	56,690(39,843-75,111)	1	119,568(83,690-159,428)	2	1.54(1.37,1.71)	4.35(4.17,4.52)	1.89(1.34,2.44)	2.61(2.42,2.80)	10
60-64 years	55,664(41,327-71,295)	3	116,326(86,251-150,255)	3	1.97(1.94,2.00)	2.99(2.91,3.08)	2.49(2.28,2.69)	2.55(2.48,2.63)	11
65-69 years	44,216(32,696-56,814)	7	99,235(73,056-127,372)	5	2.38(2.30,2.45)	2.27(2.25,2.30)	3.96(3.82,4.10)	2.83(2.77,2.88)	6
70-74 years	25,888(18,503-34,345)	12	60,322(42,470-80,450)	9	3.05(2.96,3.15)	2.55(2.52,2.58)	3.38(3.23,3.53)	2.96(2.91,3.02)	4
75-79 years	13,003(10,704-15,398)	14	28,590(23,349-34,209)	13	2.44(2.28,2.60)	2.89(2.79,3.00)	2.74(2.68,2.81)	2.74(2.67,2.80)	8
80-84 years	5,233(4,042-6,613)	16	12,245(9,345-15,673)	15	1.75(1.61,1.88)	3.75(3.65,3.86)	3.14(2.77,3.52)	2.94(2.81,3.07)	5
85-90 years	1,597(1,235-2,022)	17	4,427(3,381-5,654)	17	3.86(3.75,3.98)	3.27(3.05,3.48)	3.67(3.56,3.79)	3.56(3.47,3.66)	3
90-94 years	281(218-354)	18	1,013(775-1,289)	18	5.41(5.23,5.58)	2.98(2.85,3.12)	5.05(4.96,5.15)	4.51(4.43,4.59)	2
95+ years	17(13-21)	19	73(56-93)	19	6.46(6.27,6.65)	5.10(4.91,5.29)	3.91(3.09,4.73)	5.19(4.92,5.47)	1
Psoriasis									
<5 years	220,212(199,004-240,311)	12	195,900(176,999-213,529)	14	-0.47(-0.65,-0.29)	-0.38(-0.41,-0.34)	-0.34(-0.37,-0.31)	-0.40(-0.45,-0.34)	20
5- 9 years	244,422(216,269-272,185)	9	224,918(197,971-250,738)	13	0.04(-0.06,0.14)	-0.70(-0.75,-0.64)	-0.11(-0.15,-0.06)	-0.28(-0.32,-0.23)	19
10-14 years	232,271(202,318-263,608)	10	225,277(195,665-256,049)	12	1.06(1.01,1.10)	-1.26(-1.32,-1.20)	-0.16(-0.29,-0.03)	-0.09(-0.14,-0.04)	16
15-19 years	245,082(211,500-278,449)	8	229,544(198,573-261,516)	11	0.09(-0.03,0.20)	0.11(0.03,0.19)	-0.87(-0.97,-0.77)	-0.23(-0.30,-0.17)	18
20-24 years	274,187(236,758-315,552)	5	263,679(227,069-304,388)	9	-0.55(-0.76,-0.34)	1.23(1.15,1.31)	-1.20(-1.29,-1.12)	-0.15(-0.23,-0.08)	17
25-29 years	306,869(268,611-351,779)	3	329,019(287,837-377,490)	7	0.28(0.16,0.40)	0.22(0.17,0.27)	0.18(0.06,0.31)	0.23(0.17,0.29)	15
30-34 years	310,175(270,601-352,324)	2	377,898(329,625-428,804)	5	1.69(1.55,1.83)	-0.39(-0.55,-0.24)	1.04(0.93,1.16)	0.71(0.62,0.79)	13
35-39 years	323,456(283,337-366,168)	1	384,945(337,243-436,008)	4	1.23(1.01,1.45)	0.45(0.33,0.57)	-0.05(-0.34,0.25)	0.57(0.43,0.71)	14
40-44 years	292,067(254,845-329,721)	4	387,668(338,659-436,174)	3	1.85(1.55,2.16)	1.92(1.66,2.19)	-0.57(-0.71,-0.43)	0.98(0.82,1.13)	12
45-49 years	252,758(220,720-287,012)	7	407,782(355,091-463,612)	2	3.28(3.08,3.47)	1.27(1.02,1.52)	0.60(0.35,0.85)	1.71(1.56,1.87)	9
50-54 years	254,805(222,877-290,379)	6	420,014(366,360-479,954)	1	1.35(1.02,1.69)	2.01(1.72,2.29)	1.94(1.68,2.21)	1.81(1.62,2.01)	8
55-59 years	226,007(198,693-255,828)	11	370,094(325,853-418,393)	6	0.56(0.20,0.92)	3.17(2.82,3.51)	1.49(1.35,1.63)	1.67(1.49,1.85)	10

60-64 years	192,952(168,294-218,675)	13	304,459(266,203-345,872)	8	0.60(0.54,0.66)	1.86(1.69,2.02)	2.14(1.77,2.51)	1.60(1.45,1.74)	11
65-69 years	140,387(122,032-158,586)	14	237,012(206,877-267,873)	10	1.25(1.01,1.49)	0.96(0.89,1.03)	3.47(3.06,3.88)	1.82(1.67,1.97)	7
70-74 years	74,746(64,464-85,180)	15	141,995(122,359-161,577)	15	3.01(2.79,3.24)	1.37(1.30,1.44)	2.42(2.18,2.66)	2.23(2.11,2.34)	5
75-79 years	39,974(34,196-46,123)	16	70,252(60,382-80,878)	16	1.69(1.43,1.95)	2.30(2.22,2.38)	1.62(1.40,1.84)	1.96(1.84,2.08)	6
80-84 years	16,265(13,835-18,863)	17	33,605(28,611-39,109)	17	0.53(0.24,0.82)	4.12(3.97,4.26)	2.68(2.62,2.74)	2.54(2.43,2.65)	4
85-90 years	5,077(4,299-5,937)	18	12,967(10,954-15,102)	18	3.35(3.19,3.51)	3.36(2.99,3.73)	3.51(3.38,3.65)	3.28(3.14,3.43)	3
90-94 years	1,251(1,044-1,462)	19	4,341(3,628-5,067)	19	4.92(4.71,5.12)	2.25(1.83,2.67)	5.48(4.86,6.10)	4.31(4.05,4.58)	2
95+ years	275(220-335)	20	1,225(977-1,503)	20	6.21(6.05,6.38)	5.48(5.14,5.82)	4.47(3.72,5.24)	5.41(5.13,5.68)	1
Atopic dermatitis									
<5 years	6,281,045(5,684,074-6,924,202)	1	6,346,386(5,750,027-6,989,495)	1	-0.35(-0.42,-0.29)	0.18(0.09,0.28)	0.18(0.10,0.25)	0.02(-0.03,0.07)	20
5- 9 years	2,663,303(2,293,129-3,100,679)	2	2,914,836(2,508,482-3,384,640)	2	0.54(0.50,0.58)	0.21(0.18,0.23)	0.20(0.17,0.23)	0.30(0.28,0.32)	18
10-14 years	1,475,014(1,227,276-1,750,315)	3	1,744,755(1,445,179-2,059,889)	3	1.68(1.63,1.73)	-0.36(-0.43,-0.28)	0.37(0.32,0.41)	0.59(0.56,0.62)	16
15-19 years	1,298,070(1,087,081-1,523,209)	5	1,418,726(1,167,003-1,677,468)	4	0.62(0.51,0.72)	0.66(0.61,0.72)	-0.38(-0.49,-0.27)	0.31(0.26,0.37)	17
20-24 years	1,309,818(1,106,789-1,528,558)	4	1,355,849(1,131,386-1,599,689)	6	-0.39(-0.50,-0.28)	1.96(1.60,2.32)	-1.54(-1.68,-1.41)	0.07(-0.06,0.19)	19
25-29 years	1,135,950(944,382-1,333,009)	6	1,379,234(1,132,493-1,615,384)	5	1.08(0.80,1.36)	0.24(0.14,0.35)	0.64(0.33,0.95)	0.67(0.52,0.82)	15
30-34 years	883,582(731,188-1,040,370)	7	1,294,847(1,067,620-1,530,099)	7	3.21(2.93,3.48)	-0.40(-0.75,-0.05)	1.72(1.46,1.97)	1.38(1.18,1.58)	13
35-39 years	757,269(622,755-898,719)	8	1,051,673(857,530-1,255,548)	9	1.82(1.46,2.17)	1.08(0.89,1.27)	0.18(-0.31,0.66)	1.07(0.85,1.30)	14
40-44 years	594,589(501,512-694,034)	9	977,332(818,357-1,143,108)	11	2.88(2.53,3.23)	3.00(2.68,3.31)	-0.21(-0.37,-0.05)	1.76(1.57,1.94)	12
45-49 years	489,857(401,445-571,732)	11	1,021,178(833,167-1,198,603)	10	4.28(4.16,4.41)	1.94(1.61,2.28)	1.39(1.19,1.60)	2.65(2.50,2.79)	7
50-54 years	494,211(410,748-586,461)	10	1,080,021(898,186-1,276,234)	8	2.24(1.79,2.69)	3.00(2.59,3.40)	3.39(3.03,3.75)	2.84(2.56,3.11)	5
55-59 years	460,737(388,978-536,282)	12	946,557(801,248-1,102,558)	12	1.10(0.98,1.23)	4.33(4.16,4.50)	1.97(1.53,2.42)	2.49(2.33,2.65)	10
60-64 years	401,677(334,211-467,575)	13	801,090(664,259-931,599)	13	1.59(1.46,1.72)	2.37(2.09,2.66)	3.19(2.96,3.43)	2.43(2.31,2.56)	11
65-69 years	333,833(279,642-392,691)	14	704,029(588,338-816,887)	14	2.15(1.99,2.32)	1.47(1.39,1.56)	4.44(4.05,4.82)	2.60(2.46,2.73)	8
70-74 years	245,106(207,008-285,198)	15	539,189(453,753-623,657)	15	3.59(3.32,3.85)	1.76(1.70,1.82)	3.00(2.76,3.24)	2.75(2.63,2.87)	6
75-79 years	184,204(154,522-216,480)	16	379,496(318,809-443,836)	16	2.52(2.35,2.68)	2.81(2.75,2.87)	2.00(1.89,2.11)	2.52(2.44,2.59)	9
80-84 years	109,412(90,502-129,555)	17	264,211(220,401-311,328)	17	1.81(1.48,2.14)	4.29(4.14,4.44)	3.07(3.00,3.14)	3.12(3.00,3.24)	4
85-90 years	50,398(43,030-58,788)	18	148,457(127,316-172,375)	18	3.91(3.78,4.04)	3.70(3.46,3.94)	3.84(3.72,3.97)	3.79(3.69,3.90)	3
90-94 years	15,442(13,075-18,099)	19	60,293(50,818-70,403)	19	5.37(5.05,5.70)	3.37(2.94,3.80)	5.77(5.56,5.97)	4.86(4.67,5.05)	2
95+ years	3,643(2,639-4,770)	20	17,860(12,757-23,533)	20	6.26(6.18,6.33)	5.79(5.61,5.97)	5.01(4.65,5.37)	5.70(5.56,5.83)	1

sTable 3. The incident cases and age-standardized rate of incidence attributable to overall immune mediated inflammatory diseases according to 204 countries and territories and its temporal trends from 1990 to 2019

	1990		2019		1990-1999	2000-2009	2010-2019	1990-2019	
	N(95%CI)	Rank	N(95%CI)	Rank	AAPC(95%CI)	AAPC(95%CI)	AAPC(95%CI)	AAPC(95%CI)	Rank
**Age standardized rate**									
China	885.33(766.00-1,039.30)	150	835.34(719.22-992.26)	141	-1.15(-1.27,-1.04)	-0.80(-0.94,-0.66)	1.21(-0.34,2.79)	-0.26(-0.75,0.24)	112
Democratic People's Republic of Korea	1,099.92(948.88-1,280.90)	94	1,068.49(927.62-1,236.72)	82	0.31(0.02,0.60)	-0.33(-0.37,-0.30)	-0.24(-0.26,-0.21)	-0.11(-0.21,-0.02)	78
Taiwan (Province of China)	1,176.56(1,023.32-1,367.21)	66	1,135.56(969.86-1,362.88)	55	0.55(0.17,0.94)	0.11(-0.40,0.63)	-1.21(-1.45,-0.97)	-0.23(-0.45,-0.01)	108
Cambodia	932.57(825.41-1,055.98)	133	958.14(838.90-1,104.82)	109	-0.34(-0.40,-0.28)	0.15(0.06,0.24)	0.56(0.46,0.66)	0.12(0.07,0.17)	17
Indonesia	1,167.81(1,037.36-1,326.19)	70	1,128.38(994.17-1,290.41)	59	-0.65(-0.74,-0.56)	0.37(0.25,0.49)	-0.16(-0.47,0.14)	-0.14(-0.25,-0.04)	85
Lao People's Democratic Republic	969.43(863.13-1,092.68)	124	910.17(811.20-1,030.84)	117	-0.34(-0.38,-0.31)	-0.30(-0.32,-0.28)	0.01(-0.03,0.05)	-0.22(-0.24,-0.20)	102
Malaysia	1,017.25(906.09-1,147.79)	115	961.48(834.27-1,132.70)	108	-0.35(-0.41,-0.29)	-0.05(-0.28,0.18)	-0.19(-0.48,0.10)	-0.22(-0.34,-0.09)	103
Maldives	1,134.53(990.61-1,313.25)	79	926.34(793.53-1,087.94)	115	-0.74(-0.79,-0.69)	-1.00(-1.02,-0.97)	-0.25(-0.31,-0.19)	-0.69(-0.72,-0.66)	194
Myanmar	933.48(844.13-1,037.15)	132	906.45(817.29-1,015.91)	119	-0.28(-0.30,-0.26)	-0.17(-0.18,-0.16)	0.17(0.15,0.20)	-0.10(-0.11,-0.09)	70
Philippines	1,453.40(1,245.55-1,732.66)	14	1,286.24(1,111.69-1,518.09)	20	-0.70(-0.74,-0.67)	-0.31(-0.32,-0.30)	-0.22(-0.26,-0.18)	-0.42(-0.44,-0.40)	160
Sri Lanka	1,058.39(942.39-1,186.47)	105	1,075.34(951.30-1,227.65)	79	0.18(0.16,0.19)	-0.03(-0.07,0.00)	0.03(-0.01,0.06)	0.05(0.03,0.07)	27
Thailand	1,106.99(982.09-1,259.73)	92	1,077.90(928.09-1,265.20)	78	-0.04(-0.10,0.01)	-0.60(-0.82,-0.37)	0.54(0.15,0.94)	-0.09(-0.25,0.06)	65
Timor-Leste	1,066.37(937.16-1,218.69)	102	1,023.55(902.02-1,169.32)	99	-0.28(-0.33,-0.24)	-0.27(-0.29,-0.24)	0.17(0.13,0.22)	-0.14(-0.16,-0.11)	86
Viet Nam	973.03(857.33-1,115.70)	123	1,025.03(897.62-1,187.94)	97	0.45(0.29,0.61)	-0.27(-0.43,-0.11)	0.39(0.28,0.51)	0.18(0.09,0.27)	6
Fiji	1,241.28(1,101.15-1,395.78)	47	1,065.15(940.49-1,215.33)	84	-0.39(-0.45,-0.32)	-1.02(-1.07,-0.96)	-0.15(-0.26,-0.04)	-0.53(-0.58,-0.49)	181
Kiribati	1,420.38(1,255.15-1,598.83)	18	1,230.12(1,096.92-1,380.69)	33	-0.33(-0.35,-0.30)	-0.68(-0.69,-0.67)	-0.44(-0.47,-0.42)	-0.49(-0.51,-0.48)	175
Marshall Islands	1,130.82(1,003.94-1,271.05)	81	1,037.11(897.95-1,196.29)	92	-0.74(-1.00,-0.47)	-0.57(-0.62,-0.53)	0.63(0.40,0.86)	-0.25(-0.37,-0.14)	111
Micronesia (Federated States of)	1,122.47(988.17-1,264.60)	83	1,053.36(901.08-1,222.85)	89	-0.56(-0.59,-0.54)	-0.56(-0.59,-0.54)	0.64(0.03,1.27)	-0.19(-0.38,0.00)	99
Papua New Guinea	1,380.22(1,214.53-1,550.26)	23	1,233.24(1,097.47-1,382.15)	32	-0.04(-0.07,-0.01)	-0.70(-0.76,-0.63)	-0.44(-0.47,-0.41)	-0.40(-0.43,-0.37)	158
Samoa	1,080.31(944.20-1,234.84)	99	1,024.84(881.68-1,196.60)	98	-0.52(-0.54,-0.49)	-0.36(-0.36,-0.35)	0.41(0.33,0.48)	-0.17(-0.20,-0.15)	90
Solomon Islands	1,129.09(988.04-1,278.85)	82	1,100.59(952.58-1,282.07)	70	-0.47(-0.88,-0.06)	-0.46(-0.56,-0.36)	0.92(0.68,1.16)	-0.04(-0.19,0.12)	52
Tonga	1,217.82(1,053.90-1,427.41)	55	1,130.65(963.45-1,328.99)	56	-0.31(-0.57,-0.05)	-0.73(-0.82,-0.64)	0.40(0.22,0.58)	-0.22(-0.34,-0.11)	104
Vanuatu	1,084.97(953.95-1,226.48)	98	1,057.55(877.28-1,252.91)	86	-0.66(-0.71,-0.62)	-0.66(-0.71,-0.62)	1.44(0.46,2.44)	-0.02(-0.32,0.29)	45
Armenia	1,032.17(875.95-1,216.66)	111	1,034.67(864.38-1,229.92)	93	-0.20(-0.27,-0.13)	-0.03(-0.04,-0.01)	0.29(0.24,0.35)	0.02(-0.01,0.05)	32
Azerbaijan	1,048.62(896.28-1,233.87)	107	1,023.12(866.40-1,210.69)	100	-0.26(-0.29,-0.23)	-0.20(-0.24,-0.16)	0.29(0.16,0.42)	-0.06(-0.11,0.00)	59
Georgia	1,040.41(900.02-1,214.18)	109	1,019.87(855.18-1,217.02)	101	-0.33(-0.38,-0.27)	0.10(-0.18,0.39)	0.06(-0.06,0.17)	-0.06(-0.16,0.04)	60
Kazakhstan	995.85(859.03-1,153.82)	117	995.24(851.94-1,163.41)	104	-0.38(-0.44,-0.33)	-0.01(-0.03,0.01)	0.44(0.38,0.50)	0.02(-0.01,0.04)	33
Kyrgyzstan	1,188.53(1,023.45-1,392.98)	62	1,098.41(926.53-1,304.71)	71	-0.50(-0.58,-0.42)	-0.34(-0.37,-0.30)	0.04(-0.08,0.15)	-0.27(-0.32,-0.23)	119
Mongolia	1,115.47(962.88-1,292.75)	86	1,057.50(899.64-1,244.44)	87	-0.31(-0.35,-0.27)	-0.28(-0.29,-0.26)	0.06(0.03,0.09)	-0.18(-0.20,-0.16)	94
Tajikistan	1,114.95(956.82-1,297.74)	87	1,080.92(916.92-1,273.14)	77	-0.29(-0.41,-0.18)	-0.21(-0.23,-0.18)	0.20(0.12,0.29)	-0.10(-0.15,-0.05)	71
Turkmenistan	1,133.49(977.33-1,327.81)	80	1,027.52(862.18-1,224.74)	96	-0.67(-0.70,-0.63)	-0.57(-0.66,-0.48)	0.28(0.21,0.35)	-0.32(-0.36,-0.28)	136
Uzbekistan	1,276.87(1,094.04-1,489.03)	39	1,171.22(997.69-1,384.03)	45	-0.17(-0.21,-0.14)	-0.68(-0.72,-0.64)	0.00(-0.06,0.06)	-0.31(-0.33,-0.28)	133
Albania	713.88(626.23-834.75)	185	738.57(627.32-879.45)	168	-0.41(-0.52,-0.31)	0.58(0.39,0.76)	0.23(-0.26,0.73)	0.14(-0.04,0.31)	14

Bosnia and Herzegovina	937.08(797.57-1,114.23)	131	924.14(774.65-1,114.69)	116	-0.09(-0.17,-0.02)	-0.08(-0.23,0.08)	0.00(-0.02,0.02)	-0.05(-0.11,0.01)	54
Bulgaria	870.74(746.24-1,026.99)	153	816.38(679.02-978.62)	147	-0.56(-0.80,-0.32)	-0.15(-0.23,-0.07)	0.09(0.03,0.16)	-0.22(-0.32,-0.12)	105
Croatia	988.97(837.75-1,169.13)	118	874.01(734.37-1,045.79)	128	-0.63(-0.78,-0.47)	-0.60(-0.66,-0.54)	0.09(-0.08,0.26)	-0.42(-0.50,-0.34)	161
Czechia	759.41(638.87-906.92)	177	753.01(630.11-905.79)	165	-0.30(-0.47,-0.14)	-0.08(-0.15,-0.02)	0.46(0.32,0.60)	0.00(-0.08,0.08)	39
Hungary	905.98(769.95-1,070.72)	143	844.57(709.83-1,008.97)	136	-0.58(-0.86,-0.29)	0.06(-0.26,0.38)	-0.19(-0.36,-0.02)	-0.26(-0.41,-0.11)	113
North Macedonia	1,138.87(946.06-1,368.20)	78	989.27(844.99-1,168.83)	105	-0.13(-0.35,0.08)	-1.26(-1.50,-1.01)	-0.04(-0.09,0.01)	-0.49(-0.60,-0.38)	176
Montenegro	783.89(654.30-940.01)	166	817.62(681.32-993.61)	146	-0.16(-0.36,0.04)	0.07(0.02,0.11)	0.67(0.49,0.86)	0.18(0.10,0.27)	7
Poland	1,400.60(1,196.48-1,640.21)	19	1,066.75(878.45-1,306.26)	83	-0.77(-0.94,-0.59)	-1.49(-1.57,-1.41)	-0.59(-0.67,-0.51)	-0.98(-1.06,-0.91)	201
Romania	829.27(698.22-1,004.79)	160	823.90(676.57-1,005.77)	143	0.15(0.06,0.25)	-0.13(-0.23,-0.03)	-0.03(-0.12,0.06)	-0.02(-0.08,0.04)	46
Serbia	776.78(666.21-913.10)	169	736.50(621.50-877.51)	169	-0.09(-0.13,-0.05)	-0.36(-0.38,-0.35)	-0.05(-0.09,0.00)	-0.18(-0.20,-0.16)	95
Slovakia	748.73(635.21-888.20)	178	735.03(615.52-878.37)	171	-0.26(-0.41,-0.12)	-0.01(-0.05,0.03)	0.18(0.05,0.30)	-0.05(-0.12,0.02)	55
Slovenia	1,044.30(892.95-1,231.17)	108	933.96(777.74-1,128.27)	111	-0.43(-0.52,-0.33)	-0.54(-0.56,-0.52)	-0.10(-0.20,0.00)	-0.37(-0.41,-0.33)	150
Belarus	973.88(819.22-1,156.26)	122	813.19(664.08-998.74)	148	-0.79(-0.93,-0.65)	-1.25(-1.35,-1.14)	0.37(0.18,0.56)	-0.59(-0.68,-0.50)	187
Estonia	830.76(711.32-961.43)	159	754.90(627.10-901.28)	163	-0.22(-0.29,-0.15)	-1.40(-1.59,-1.21)	0.95(0.58,1.33)	-0.30(-0.44,-0.16)	128
Latvia	844.46(728.98-995.58)	155	734.71(591.82-905.12)	173	-1.26(-1.31,-1.21)	0.03(-0.09,0.15)	-0.04(-0.14,0.07)	-0.45(-0.51,-0.40)	165
Lithuania	765.10(653.18-898.11)	174	735.43(599.08-890.02)	170	-0.91(-0.98,-0.83)	0.54(0.31,0.76)	0.18(0.02,0.34)	-0.10(-0.19,-0.01)	72
Republic of Moldova	817.52(687.58-986.41)	161	730.66(596.36-899.36)	176	-0.49(-0.53,-0.45)	-0.71(-0.76,-0.65)	0.11(0.02,0.19)	-0.37(-0.41,-0.33)	151
Russian Federation	1,024.08(869.23-1,224.68)	114	795.83(658.56-961.28)	155	-0.69(-0.80,-0.57)	-1.81(-1.92,-1.70)	0.05(-0.17,0.27)	-0.86(-0.95,-0.77)	198
Ukraine	982.98(796.50-1,195.29)	120	844.36(672.02-1,054.86)	137	-0.66(-0.80,-0.51)	-1.18(-1.33,-1.03)	0.49(0.27,0.72)	-0.49(-0.60,-0.38)	177
Brunei Darussalam	1,191.79(1,030.46-1,390.85)	61	1,145.03(986.93-1,337.80)	52	0.25(0.12,0.38)	-0.43(-0.47,-0.39)	-0.20(-0.29,-0.11)	-0.15(-0.20,-0.10)	87
Japan	1,614.93(1,396.77-1,896.75)	8	1,241.81(1,054.64-1,475.33)	31	-1.23(-1.41,-1.06)	-1.98(-2.14,-1.81)	0.56(0.44,0.69)	-0.97(-1.06,-0.87)	200
Republic of Korea	1,118.48(978.01-1,290.74)	85	1,047.20(895.43-1,235.07)	90	-0.50(-0.64,-0.36)	0.02(-0.15,0.19)	-0.17(-0.21,-0.14)	-0.26(-0.33,-0.18)	114
Singapore	1,183.74(1,021.46-1,365.44)	64	985.96(828.17-1,184.04)	106	-1.03(-1.11,-0.96)	-0.67(-0.72,-0.62)	-0.11(-0.14,-0.08)	-0.63(-0.66,-0.60)	192
Australia	1,281.61(1,079.48-1,493.79)	37	1,129.09(949.19-1,341.98)	58	0.53(0.43,0.64)	-1.94(-2.25,-1.63)	0.05(-0.09,0.18)	-0.46(-0.58,-0.35)	168
New Zealand	1,621.31(1,370.19-1,916.85)	7	1,350.11(1,131.25-1,623.44)	14	-0.99(-1.10,-0.88)	-0.64(-0.72,-0.56)	-0.20(-0.24,-0.16)	-0.64(-0.69,-0.60)	193
Andorra	1,207.71(1,055.09-1,385.04)	58	1,164.41(1,010.52-1,340.25)	46	-0.15(-0.17,-0.13)	-0.25(-0.26,-0.24)	0.03(0.00,0.07)	-0.12(-0.14,-0.11)	79
Austria	1,237.31(1,106.06-1,393.55)	50	1,150.15(1,006.78-1,311.69)	50	-0.42(-0.52,-0.31)	-0.08(-0.15,-0.01)	-0.28(-0.35,-0.21)	-0.27(-0.32,-0.22)	120
Belgium	1,252.85(1,123.13-1,410.10)	43	1,115.58(975.32-1,266.91)	62	-1.17(-1.39,-0.94)	0.32(0.18,0.46)	-0.34(-0.45,-0.22)	-0.45(-0.55,-0.36)	166
Cyprus	1,253.57(1,090.42-1,444.60)	42	1,214.02(1,047.30-1,406.41)	39	-0.45(-0.53,-0.36)	-0.02(-0.04,-0.01)	0.13(0.11,0.15)	-0.10(-0.13,-0.07)	73
Denmark	1,341.27(1,183.76-1,532.24)	27	1,251.08(1,082.77-1,444.89)	29	-0.34(-0.40,-0.28)	-0.12(-0.28,0.03)	-0.38(-0.69,-0.08)	-0.27(-0.38,-0.16)	121
Finland	1,226.54(1,091.00-1,387.99)	51	1,193.22(1,050.59-1,353.60)	42	0.01(-0.01,0.03)	-0.04(-0.09,0.00)	-0.23(-0.26,-0.19)	-0.09(-0.11,-0.07)	66
France	1,492.07(1,332.65-1,696.75)	11	1,355.17(1,185.70-1,548.51)	11	-0.58(-0.63,-0.53)	-0.35(-0.45,-0.26)	-0.08(-0.28,0.11)	-0.35(-0.42,-0.28)	145
Germany	1,208.39(1,074.29-1,353.27)	57	1,045.66(910.66-1,197.48)	91	-1.63(-1.74,-1.51)	0.13(-0.02,0.28)	-0.01(-0.32,0.30)	-0.46(-0.58,-0.35)	169
Greece	1,098.71(965.62-1,260.97)	95	1,074.41(928.00-1,237.89)	80	-0.37(-0.47,-0.28)	0.28(0.24,0.33)	-0.18(-0.22,-0.14)	-0.08(-0.12,-0.05)	63
Iceland	1,624.88(1,389.11-1,894.08)	6	1,450.20(1,243.88-1,700.39)	4	-0.28(-0.51,-0.04)	-0.77(-0.89,-0.65)	-0.14(-0.30,0.02)	-0.39(-0.50,-0.28)	156
Ireland	1,420.80(1,252.76-1,615.07)	17	1,245.90(1,085.04-1,424.30)	30	-0.57(-0.64,-0.49)	-0.33(-0.44,-0.22)	-0.56(-0.86,-0.25)	-0.48(-0.59,-0.38)	173
Israel	1,204.39(1,062.03-1,368.96)	59	1,114.24(971.02-1,270.24)	64	-0.52(-0.57,-0.46)	-0.23(-0.25,-0.21)	0.00(-0.03,0.04)	-0.27(-0.29,-0.25)	122
Italy	1,250.42(1,116.83-1,405.65)	45	1,163.04(1,004.85-1,332.75)	47	-0.42(-0.68,-0.17)	-0.14(-0.63,0.35)	-0.28(-0.48,-0.08)	-0.30(-0.49,-0.11)	129

Luxembourg	1,341.65(1,166.29-1,571.04)	26	1,217.03(1,055.67-1,396.37)	36	-0.17(-0.25,-0.08)	-0.56(-0.58,-0.54)	-0.29(-0.36,-0.22)	-0.35(-0.39,-0.32)	146
Malta	1,329.63(1,183.03-1,500.60)	28	1,223.01(1,063.49-1,402.79)	34	-0.42(-0.49,-0.35)	-0.24(-0.25,-0.22)	-0.18(-0.27,-0.08)	-0.29(-0.33,-0.26)	124
Netherlands	1,077.54(972.27-1,198.03)	100	1,027.71(920.49-1,144.95)	95	0.00(-0.15,0.16)	-0.56(-0.60,-0.51)	0.18(0.13,0.22)	-0.16(-0.21,-0.10)	88
Norway	1,626.01(1,420.56-1,870.93)	5	1,375.92(1,179.95-1,599.10)	9	-0.53(-0.61,-0.46)	-0.69(-0.78,-0.60)	-0.52(-0.81,-0.22)	-0.60(-0.72,-0.48)	188
Portugal	1,435.49(1,267.36-1,649.16)	16	1,332.76(1,156.18-1,530.66)	17	-0.71(-0.95,-0.46)	0.64(0.50,0.78)	-0.60(-0.71,-0.50)	-0.31(-0.41,-0.20)	134
Spain	1,119.04(985.81-1,281.99)	84	1,125.86(979.83-1,296.43)	60	-0.39(-0.43,-0.36)	0.59(0.55,0.64)	-0.18(-0.31,-0.04)	0.02(-0.03,0.06)	34
Sweden	1,529.45(1,338.95-1,759.50)	10	1,302.05(1,118.44-1,520.81)	18	-1.57(-1.64,-1.50)	-0.73(-0.87,-0.60)	0.64(0.28,1.00)	-0.58(-0.71,-0.46)	186
Switzerland	1,311.29(1,149.64-1,498.92)	29	1,209.06(1,051.21-1,400.62)	41	-0.33(-0.34,-0.31)	-0.49(-0.50,-0.48)	-0.02(-0.05,0.01)	-0.28(-0.30,-0.27)	123
United Kingdom	1,761.41(1,523.29-2,026.38)	2	1,507.40(1,293.90-1,749.50)	3	-0.66(-1.17,-0.14)	-0.66(-0.74,-0.58)	-0.32(-0.80,0.15)	-0.55(-0.78,-0.33)	184
Argentina	1,285.81(1,121.73-1,491.97)	36	1,300.65(1,103.18-1,545.49)	19	0.14(0.09,0.19)	0.17(0.14,0.19)	-0.18(-0.21,-0.14)	0.03(0.01,0.05)	29
Chile	1,184.06(1,033.03-1,372.13)	63	1,171.50(1,002.71-1,386.85)	44	0.05(-0.01,0.11)	-0.41(-0.48,-0.35)	0.38(0.27,0.48)	-0.01(-0.06,0.04)	44
Uruguay	1,277.09(1,106.20-1,488.07)	38	1,210.32(1,031.86-1,432.73)	40	-0.36(-0.43,-0.29)	-0.25(-0.50,0.01)	0.03(-0.08,0.15)	-0.21(-0.30,-0.11)	101
Canada	1,246.44(1,053.58-1,489.75)	46	1,181.52(990.31-1,411.66)	43	0.23(-0.10,0.55)	0.39(0.27,0.51)	-0.83(-1.21,-0.45)	-0.12(-0.30,0.05)	80
United States of America	1,886.73(1,532.77-2,353.14)	1	1,988.77(1,669.58-2,365.69)	1	-1.99(-2.25,-1.72)	1.47(1.32,1.63)	0.88(0.69,1.07)	0.22(0.08,0.35)	5
Antigua and Barbuda	1,217.77(992.05-1,512.26)	56	1,258.20(1,012.24-1,543.43)	26	-0.05(-0.26,0.17)	0.00(-0.07,0.07)	0.50(0.33,0.66)	0.15(0.05,0.25)	12
Bahamas	1,222.72(999.74-1,511.82)	53	1,257.62(1,010.36-1,550.98)	27	0.02(-0.18,0.22)	-0.07(-0.13,-0.01)	0.44(0.30,0.58)	0.13(0.05,0.22)	15
Barbados	1,295.19(1,081.77-1,563.47)	35	1,372.22(1,112.34-1,689.18)	10	0.07(0.02,0.12)	0.56(0.46,0.65)	-0.12(-0.37,0.12)	0.18(0.10,0.27)	8
Belize	1,299.92(1,065.66-1,572.10)	33	1,251.78(1,012.05-1,529.42)	28	-0.08(-0.37,0.21)	-0.31(-0.36,-0.26)	0.16(-0.10,0.42)	-0.09(-0.22,0.03)	67
Cuba	1,389.49(1,131.86-1,695.51)	20	1,391.28(1,119.30-1,713.93)	8	0.03(-0.02,0.08)	-0.15(-0.26,-0.04)	0.21(0.11,0.30)	0.03(-0.02,0.08)	30
Dominica	1,307.33(1,079.60-1,581.93)	31	1,351.04(1,102.03-1,645.67)	13	0.48(0.38,0.58)	-0.01(-0.05,0.03)	-0.08(-0.17,0.00)	0.13(0.08,0.18)	16
Dominican Republic	1,054.09(878.98-1,248.08)	106	1,029.71(848.57-1,260.20)	94	-1.05(-1.21,-0.88)	0.55(0.41,0.70)	0.45(0.09,0.82)	-0.02(-0.16,0.13)	47
Grenada	1,368.12(1,106.38-1,678.39)	24	1,396.33(1,135.24-1,726.54)	7	0.03(-0.01,0.07)	-0.06(-0.08,-0.04)	0.26(0.22,0.31)	0.07(0.05,0.09)	23
Guyana	1,305.84(1,084.63-1,566.48)	32	1,282.56(1,054.65-1,559.92)	22	0.16(0.04,0.28)	-0.44(-0.48,-0.39)	0.18(0.09,0.27)	-0.05(-0.11,0.00)	56
Haiti	1,540.60(1,274.10-1,832.14)	9	1,414.52(1,173.72-1,693.36)	5	-0.27(-0.32,-0.22)	-0.58(-0.61,-0.55)	0.04(-0.02,0.09)	-0.29(-0.32,-0.25)	125
Jamaica	1,381.78(1,131.74-1,693.69)	22	1,264.12(1,026.36-1,559.03)	24	-0.18(-0.31,-0.06)	-0.67(-0.70,-0.65)	0.03(-0.33,0.39)	-0.30(-0.42,-0.19)	130
Saint Lucia	1,361.73(1,124.24-1,645.10)	25	1,341.03(1,092.66-1,633.57)	16	0.24(0.20,0.27)	-0.42(-0.44,-0.40)	0.09(-0.07,0.24)	-0.06(-0.11,-0.01)	61
Saint Vincent and the Grenadines	1,252.12(1,030.05-1,531.82)	44	1,259.20(1,023.02-1,537.27)	25	-0.19(-0.31,-0.07)	-0.09(-0.17,0.00)	0.47(0.32,0.61)	0.05(-0.02,0.13)	28
Suriname	1,218.18(997.74-1,503.70)	54	1,220.75(987.85-1,506.62)	35	-0.15(-0.16,-0.14)	-0.15(-0.16,-0.14)	0.38(0.23,0.53)	0.02(-0.03,0.06)	35
Trinidad and Tobago	1,065.57(883.46-1,293.29)	103	1,141.44(917.37-1,418.92)	53	-0.12(-0.20,-0.03)	-0.12(-0.20,-0.03)	1.52(1.00,2.04)	0.39(0.22,0.56)	2
Bolivia (Plurinational State of)	1,298.23(1,054.78-1,563.32)	34	1,140.41(927.51-1,423.83)	54	-0.61(-0.74,-0.48)	-0.73(-0.78,-0.67)	0.15(0.03,0.28)	-0.44(-0.50,-0.37)	164
Ecuador	1,259.93(1,014.93-1,549.56)	41	1,103.91(888.22-1,385.76)	68	-1.05(-1.39,-0.70)	-0.67(-1.04,-0.30)	0.53(0.44,0.61)	-0.47(-0.65,-0.28)	172
Peru	1,308.65(1,047.41-1,616.64)	30	1,119.59(897.18-1,417.64)	61	-0.56(-0.65,-0.47)	-1.49(-1.55,-1.42)	0.46(0.30,0.61)	-0.56(-0.63,-0.49)	185
Colombia	1,012.33(828.08-1,236.65)	116	864.31(685.31-1,080.70)	131	-1.27(-1.96,-0.58)	-0.49(-0.74,-0.24)	0.26(0.17,0.36)	-0.51(-0.75,-0.27)	178
Costa Rica	1,226.03(995.21-1,496.04)	52	1,106.14(881.31-1,380.41)	67	0.63(0.50,0.76)	-1.39(-1.88,-0.89)	-0.05(-0.24,0.15)	-0.32(-0.50,-0.14)	137
El Salvador	1,464.63(1,167.56-1,807.97)	13	1,095.95(885.67-1,365.77)	73	-1.11(-1.23,-0.99)	-1.51(-1.59,-1.43)	-0.24(-0.33,-0.15)	-1.02(-1.08,-0.95)	203
Guatemala	1,111.09(896.70-1,341.52)	91	786.34(634.30-981.30)	161	-1.54(-1.76,-1.32)	-1.84(-2.31,-1.37)	-0.25(-0.32,-0.19)	-1.24(-1.41,-1.06)	204
Honduras	1,154.41(928.46-1,407.86)	76	929.04(752.18-1,150.73)	112	-0.62(-1.01,-0.22)	-1.22(-1.28,-1.16)	-0.31(-0.47,-0.15)	-0.75(-0.89,-0.62)	196
Mexico	881.22(724.18-1,086.79)	152	792.47(622.69-996.78)	159	-1.35(-1.54,-1.16)	-0.39(-0.56,-0.21)	0.80(0.74,0.85)	-0.37(-0.46,-0.28)	152

Nicaragua	1,171.69(945.34-1,451.31)	67	908.67(727.14-1,135.00)	118	-1.17(-1.70,-0.64)	-0.95(-1.19,-0.70)	-0.53(-0.60,-0.46)	-0.90(-1.10,-0.71)	199
Panama	1,111.18(906.85-1,349.14)	90	999.46(799.19-1,248.65)	103	-0.21(-0.36,-0.07)	-0.61(-0.68,-0.54)	-0.21(-0.37,-0.05)	-0.37(-0.45,-0.29)	153
Venezuela (Bolivarian Republic of)	1,027.74(831.01-1,267.84)	113	927.61(741.02-1,150.44)	114	-0.38(-0.49,-0.28)	-0.61(-0.65,-0.57)	0.06(-0.02,0.14)	-0.36(-0.41,-0.31)	147
Brazil	1,484.43(1,182.98-1,853.39)	12	1,341.54(1,047.19-1,669.74)	15	-0.50(-0.68,-0.31)	-0.99(-1.07,-0.90)	0.56(0.35,0.76)	-0.36(-0.46,-0.26)	148
Paraguay	1,238.66(1,008.41-1,525.94)	48	1,351.08(1,079.05-1,684.92)	12	0.26(0.25,0.28)	0.26(0.25,0.28)	0.44(0.34,0.55)	0.32(0.28,0.35)	4
Algeria	776.27(667.09-909.14)	170	793.16(660.57-953.54)	158	-0.34(-0.42,-0.26)	0.00(-0.23,0.23)	0.72(0.59,0.86)	0.10(0.01,0.19)	19
Bahrain	917.31(775.53-1,089.26)	141	839.44(696.27-1,009.49)	139	-0.14(-0.22,-0.05)	-0.71(-0.74,-0.68)	-0.04(-0.10,0.03)	-0.30(-0.34,-0.26)	131
Egypt	776.05(648.60-929.24)	171	734.99(611.04-885.96)	172	-0.56(-0.62,-0.51)	-0.41(-0.44,-0.39)	0.51(0.45,0.57)	-0.17(-0.20,-0.14)	91
Iran (Islamic Republic of)	924.91(780.70-1,094.00)	135	807.10(668.18-967.32)	152	-0.18(-0.23,-0.12)	-0.98(-1.02,-0.93)	-0.18(-0.23,-0.14)	-0.48(-0.51,-0.45)	174
Iraq	976.17(826.58-1,149.20)	121	818.72(680.39-993.40)	144	-0.71(-0.72,-0.69)	-0.74(-0.75,-0.73)	-0.34(-0.36,-0.32)	-0.61(-0.62,-0.60)	190
Jordan	898.88(751.56-1,081.48)	144	886.70(725.46-1,081.55)	124	-0.03(-0.11,0.05)	-0.27(-0.29,-0.24)	0.21(0.13,0.30)	-0.02(-0.06,0.02)	48
Kuwait	914.86(761.00-1,095.56)	142	891.52(718.43-1,098.30)	123	-0.52(-0.55,-0.49)	0.10(0.01,0.19)	0.11(-0.06,0.29)	-0.09(-0.16,-0.03)	68
Lebanon	930.34(790.13-1,109.13)	134	929.03(774.12-1,118.03)	113	-0.10(-0.15,-0.06)	-0.14(-0.16,-0.11)	0.29(0.23,0.36)	0.01(-0.02,0.04)	37
Libya	886.79(748.80-1,040.38)	149	841.78(703.40-997.40)	138	-0.43(-0.53,-0.34)	-0.22(-0.26,-0.18)	0.19(0.09,0.30)	-0.17(-0.22,-0.12)	92
Morocco	726.74(623.95-845.23)	183	749.30(634.97-892.56)	166	-0.05(-0.09,0.00)	-0.10(-0.12,-0.07)	0.52(0.46,0.57)	0.11(0.08,0.14)	18
Palestine	836.95(708.93-989.60)	157	807.64(670.19-970.84)	151	-0.73(-0.82,-0.63)	-0.10(-0.14,-0.06)	0.55(0.44,0.65)	-0.10(-0.15,-0.05)	74
Oman	744.37(625.77-887.84)	179	861.41(706.54-1,049.20)	132	-0.35(-0.44,-0.25)	1.06(0.92,1.19)	0.98(0.57,1.40)	0.55(0.41,0.69)	1
Qatar	806.21(663.27-981.77)	164	797.03(655.43-970.45)	154	0.11(0.06,0.15)	-0.53(-0.59,-0.47)	0.38(0.15,0.60)	-0.03(-0.11,0.04)	50
Saudi Arabia	642.65(550.32-748.72)	192	708.04(584.71-851.43)	178	-0.25(-0.36,-0.14)	0.67(0.50,0.84)	0.51(0.20,0.82)	0.34(0.22,0.46)	3
Syrian Arab Republic	814.71(700.49-948.18)	162	805.98(683.88-948.69)	153	-1.04(-1.21,-0.88)	0.82(0.39,1.25)	-0.24(-0.62,0.14)	-0.10(-0.31,0.10)	75
Tunisia	832.99(715.45-984.38)	158	836.06(700.92-1,001.02)	140	0.02(-0.06,0.11)	-0.26(-0.31,-0.21)	0.30(0.23,0.37)	0.01(-0.03,0.06)	38
Turkey	1,146.72(986.32-1,326.49)	77	1,110.17(955.20-1,289.13)	65	-0.03(-0.12,0.06)	-1.09(-1.36,-0.82)	1.09(0.97,1.22)	-0.07(-0.18,0.04)	62
United Arab Emirates	1,194.73(1,004.52-1,404.42)	60	1,147.84(962.37-1,367.29)	51	0.06(-0.26,0.39)	1.27(-0.74,3.32)	-2.32(-3.10,-1.53)	-0.32(-1.03,0.40)	138
Yemen	959.86(829.47-1,114.13)	126	869.61(730.61-1,029.28)	129	-0.47(-0.52,-0.41)	-0.55(-0.59,-0.51)	0.08(0.00,0.15)	-0.33(-0.36,-0.29)	142
Afghanistan	940.44(813.93-1,081.45)	129	904.04(776.96-1,055.49)	120	-0.39(-0.46,-0.31)	-0.18(-0.20,-0.17)	0.19(0.14,0.24)	-0.13(-0.16,-0.10)	83
Bangladesh	606.42(544.32-677.83)	197	547.00(489.69-616.13)	201	-0.13(-0.24,-0.01)	-0.80(-0.86,-0.74)	-0.17(-0.23,-0.10)	-0.39(-0.44,-0.33)	157
Bhutan	632.54(567.21-708.91)	193	555.11(497.92-625.52)	200	-0.31(-0.36,-0.27)	-0.73(-0.76,-0.69)	-0.30(-0.37,-0.23)	-0.46(-0.49,-0.43)	170
India	764.63(674.80-868.07)	175	700.39(617.67-793.96)	179	-1.98(-2.54,-1.41)	1.27(0.43,2.12)	-0.38(-0.76,0.00)	-0.38(-0.72,-0.03)	155
Nepal	543.17(490.86-604.43)	202	511.02(458.42-572.95)	203	-0.16(-0.21,-0.11)	-0.69(-0.80,-0.58)	0.39(0.21,0.57)	-0.18(-0.25,-0.11)	96
Pakistan	657.47(586.93-737.66)	190	594.07(532.96-668.33)	195	-0.07(-0.37,0.24)	-0.70(-0.89,-0.51)	-0.19(-0.33,-0.05)	-0.29(-0.42,-0.16)	126
Angola	918.57(780.94-1,080.59)	140	753.19(630.30-894.30)	164	-0.34(-0.37,-0.31)	-1.09(-1.10,-1.08)	-0.60(-0.63,-0.57)	-0.69(-0.71,-0.67)	195
Central African Republic	887.09(759.86-1,040.92)	148	790.77(672.25-930.20)	160	-0.38(-0.41,-0.35)	-0.45(-0.46,-0.44)	-0.35(-0.38,-0.33)	-0.40(-0.41,-0.39)	159
Congo	882.30(744.40-1,045.20)	151	809.28(675.03-980.02)	150	-0.29(-0.41,-0.17)	-0.61(-0.71,-0.50)	0.03(-0.07,0.13)	-0.32(-0.39,-0.25)	139
Democratic Republic of the Congo	781.18(660.34-920.00)	167	731.78(619.12-873.37)	174	-0.40(-0.43,-0.38)	-0.22(-0.29,-0.15)	-0.01(-0.06,0.04)	-0.22(-0.25,-0.20)	106
Equatorial Guinea	857.94(723.81-1,000.52)	154	730.89(605.79-888.61)	175	-0.67(-0.71,-0.63)	-1.07(-1.16,-0.98)	0.19(0.05,0.32)	-0.53(-0.58,-0.47)	182
Gabon	772.67(650.14-911.93)	173	692.53(573.54-838.81)	181	-0.66(-0.67,-0.65)	-0.63(-0.65,-0.62)	0.24(0.10,0.39)	-0.36(-0.41,-0.32)	149
Burundi	1,028.23(860.16-1,228.71)	112	865.28(717.09-1,031.29)	130	-0.66(-0.69,-0.63)	-0.57(-0.65,-0.48)	-0.55(-0.64,-0.46)	-0.60(-0.65,-0.56)	189
Comoros	922.59(771.38-1,113.21)	136	852.30(702.86-1,032.31)	134	-0.60(-0.63,-0.58)	-0.44(-0.46,-0.43)	0.27(0.23,0.31)	-0.26(-0.28,-0.25)	115

Djibouti	983.93(817.45-1,185.50)	119	857.79(713.64-1,045.24)	133	-0.66(-0.69,-0.64)	-0.75(-0.82,-0.68)	0.13(0.00,0.26)	-0.45(-0.50,-0.40)	167
Eritrea	961.29(807.59-1,146.91)	125	878.12(730.69-1,059.05)	126	-0.46(-0.48,-0.44)	-0.63(-0.68,-0.57)	0.21(0.11,0.30)	-0.32(-0.36,-0.28)	140
Ethiopia	795.49(664.08-959.13)	165	690.78(558.20-856.27)	182	-0.88(-1.10,-0.65)	-0.80(-0.86,-0.75)	0.40(0.24,0.55)	-0.46(-0.54,-0.37)	171
Kenya	726.86(598.88-888.08)	182	666.17(545.13-825.33)	187	-0.72(-0.84,-0.61)	-0.43(-0.55,-0.32)	0.42(0.26,0.59)	-0.26(-0.35,-0.18)	116
Madagascar	1,386.40(1,147.44-1,700.02)	21	1,055.40(882.71-1,253.13)	88	-0.45(-0.63,-0.26)	-1.70(-1.88,-1.51)	-0.65(-0.77,-0.53)	-0.99(-1.09,-0.89)	202
Malawi	837.40(689.78-1,003.93)	156	809.61(669.78-983.53)	149	-0.40(-0.46,-0.34)	-0.27(-0.29,-0.25)	0.34(0.29,0.40)	-0.12(-0.15,-0.09)	81
Mauritius	1,161.34(1,017.95-1,326.73)	73	1,003.96(871.70-1,161.94)	102	-0.38(-0.43,-0.34)	-0.73(-0.75,-0.71)	-0.36(-0.41,-0.31)	-0.51(-0.53,-0.49)	179
Mozambique	898.84(758.18-1,077.69)	145	874.03(728.05-1,054.17)	127	-0.20(-0.22,-0.18)	-0.32(-0.33,-0.31)	0.28(0.24,0.32)	-0.10(-0.11,-0.08)	76
Rwanda	1,442.80(1,197.17-1,755.75)	15	1,214.10(996.62-1,477.21)	38	-0.67(-0.75,-0.58)	-0.41(-0.53,-0.29)	-0.70(-0.96,-0.45)	-0.62(-0.71,-0.52)	191
Seychelles	943.38(829.11-1,083.08)	127	946.55(822.55-1,103.84)	110	-0.10(-0.16,-0.04)	-0.11(-0.13,-0.09)	0.30(0.26,0.35)	0.03(0.00,0.05)	31
Somalia	1,036.54(858.38-1,239.45)	110	892.70(751.98-1,067.25)	122	-0.64(-0.70,-0.57)	-0.87(-0.91,-0.82)	-0.07(-0.11,-0.04)	-0.51(-0.54,-0.48)	180
United Republic of Tanzania	1,112.88(918.40-1,355.88)	89	1,097.53(902.24-1,330.47)	72	-0.20(-0.28,-0.11)	0.47(0.35,0.60)	-0.31(-0.49,-0.14)	-0.05(-0.13,0.03)	57
Uganda	1,076.73(892.41-1,297.20)	101	985.60(810.76-1,209.17)	107	-0.37(-0.37,-0.36)	-0.55(-0.58,-0.51)	0.07(0.03,0.12)	-0.30(-0.32,-0.28)	132
Zambia	619.27(525.02-731.11)	195	626.91(514.86-757.73)	191	-0.89(-1.01,-0.77)	-0.02(-0.09,0.04)	1.20(1.04,1.37)	0.08(0.02,0.15)	21
Botswana	601.89(516.59-699.61)	199	645.33(528.72-794.89)	189	-0.18(-0.37,0.01)	0.22(-0.29,0.74)	0.47(0.32,0.62)	0.15(-0.03,0.33)	13
Lesotho	464.97(406.16-532.98)	204	460.01(403.45-526.40)	204	-0.40(-0.45,-0.36)	-0.04(-0.11,0.02)	0.40(0.33,0.47)	-0.02(-0.05,0.02)	49
Namibia	602.69(520.32-701.15)	198	561.83(475.75-661.65)	198	-0.30(-0.35,-0.24)	-0.57(-0.59,-0.54)	0.19(0.11,0.27)	-0.24(-0.27,-0.20)	109
South Africa	887.69(693.90-1,099.63)	146	793.85(618.47-998.94)	157	0.50(-0.09,1.08)	-4.75(-6.46,-3.01)	4.70(3.92,5.49)	0.00(-0.67,0.67)	40
Eswatini	887.64(756.10-1,047.97)	147	824.79(696.72-974.46)	142	0.34(0.23,0.45)	-0.71(-0.79,-0.63)	-0.46(-0.75,-0.17)	-0.31(-0.41,-0.21)	135
Zimbabwe	525.20(453.39-610.76)	203	532.07(456.98-619.42)	202	-0.42(-0.46,-0.37)	0.06(0.02,0.11)	0.61(0.56,0.67)	0.07(0.04,0.10)	24
Benin	719.90(611.96-851.80)	184	668.95(559.41-795.99)	186	-0.58(-0.75,-0.42)	-0.35(-0.38,-0.33)	0.23(0.16,0.30)	-0.24(-0.30,-0.19)	110
Burkina Faso	650.80(548.17-773.89)	191	651.69(543.73-791.92)	188	-0.28(-0.32,-0.23)	-0.05(-0.26,0.16)	0.37(0.28,0.45)	0.00(-0.08,0.07)	41
Cameroon	658.45(566.46-777.44)	189	626.01(527.17-748.64)	192	-0.83(-0.86,-0.79)	0.00(-0.07,0.07)	0.38(0.32,0.44)	-0.18(-0.21,-0.14)	97
Cabo Verde	682.30(574.20-809.84)	187	631.13(518.26-774.80)	190	-1.07(-1.11,-1.03)	-0.30(-0.42,-0.18)	0.64(0.57,0.72)	-0.26(-0.31,-0.21)	117
Chad	608.89(520.01-715.61)	196	593.41(502.33-706.73)	196	-0.60(-0.79,-0.41)	-0.03(-0.06,-0.01)	0.36(0.27,0.45)	-0.09(-0.15,-0.02)	69
C??te d'Ivoire	730.99(621.17-873.35)	180	711.91(589.91-857.88)	177	-0.07(-0.25,0.11)	-0.34(-0.42,-0.26)	0.18(0.01,0.34)	-0.08(-0.17,0.01)	64
Gambia	711.21(602.89-839.69)	186	669.64(558.53-796.26)	185	-0.72(-0.80,-0.64)	-0.21(-0.23,-0.18)	0.40(0.35,0.46)	-0.20(-0.24,-0.17)	100
Ghana	545.92(459.02-656.06)	201	558.09(463.19-674.01)	199	-0.40(-0.46,-0.35)	-0.08(-0.11,-0.05)	0.79(0.58,0.99)	0.09(0.02,0.15)	20
Guinea	779.17(660.59-937.16)	168	747.34(630.57-901.90)	167	-0.06(-0.13,0.01)	-0.57(-0.60,-0.53)	0.24(0.16,0.32)	-0.16(-0.20,-0.12)	89
Guinea-Bissau	773.77(657.70-905.28)	172	696.36(589.15-822.77)	180	-0.71(-0.78,-0.64)	-0.47(-0.51,-0.44)	0.14(0.07,0.20)	-0.37(-0.41,-0.34)	154
Liberia	682.10(571.76-823.89)	188	619.17(512.74-751.56)	193	-0.60(-0.91,-0.29)	-0.58(-0.64,-0.52)	0.28(0.12,0.44)	-0.32(-0.43,-0.21)	141
Mali	580.58(501.46-687.02)	200	587.70(499.91-698.13)	197	-0.44(-0.52,-0.37)	0.07(-0.04,0.17)	0.59(0.48,0.70)	0.07(0.01,0.13)	25
Mauritania	939.83(783.13-1,140.51)	130	878.56(728.92-1,070.03)	125	-0.35(-0.44,-0.27)	-0.48(-0.53,-0.44)	0.15(0.10,0.21)	-0.26(-0.30,-0.22)	118
Niger	761.42(644.64-903.01)	176	672.58(564.31-806.95)	183	-0.66(-0.71,-0.60)	-0.55(-0.57,-0.54)	0.01(-0.03,0.05)	-0.43(-0.45,-0.40)	162
Nigeria	921.87(771.50-1,108.82)	138	794.11(659.01-969.69)	156	-0.88(-1.36,-0.40)	-0.51(-0.58,-0.44)	-0.19(-0.65,0.27)	-0.53(-0.74,-0.32)	183
Sao Tome and Principe	918.97(778.02-1,085.88)	139	817.97(692.29-960.32)	145	0.17(0.06,0.28)	-1.03(-1.09,-0.96)	-0.29(-0.55,-0.02)	-0.43(-0.52,-0.33)	163
Senegal	625.72(536.52-739.90)	194	603.18(507.57-725.01)	194	-0.70(-0.74,-0.67)	-0.20(-0.24,-0.17)	0.62(0.50,0.75)	-0.12(-0.16,-0.08)	82
Sierra Leone	727.81(610.88-863.86)	181	671.90(562.66-807.42)	184	-0.71(-0.80,-0.63)	-0.35(-0.66,-0.04)	0.21(0.09,0.33)	-0.33(-0.44,-0.21)	143

Togo	813.94(684.06-970.26)	163	765.19(638.02-929.28)	162	-0.31(-0.40,-0.21)	-0.49(-0.57,-0.40)	0.22(0.15,0.30)	-0.22(-0.27,-0.16)	107
American Samoa	1,106.93(950.33-1,299.62)	93	1,094.47(911.22-1,289.21)	74	-1.11(-1.48,-0.74)	0.18(0.09,0.28)	1.09(0.60,1.59)	0.06(-0.14,0.26)	26
Bermuda	1,237.93(1,001.38-1,527.63)	49	1,285.15(1,024.18-1,584.46)	21	0.16(0.12,0.19)	0.16(0.12,0.19)	0.25(-0.23,0.72)	0.17(0.02,0.32)	9
Cook Islands	1,163.69(978.10-1,381.83)	71	1,130.14(944.65-1,335.11)	57	-1.03(-1.24,-0.82)	0.13(0.08,0.18)	0.73(0.53,0.92)	-0.05(-0.14,0.05)	58
Greenland	1,742.85(1,453.06-2,100.86)	3	1,407.24(1,195.30-1,689.35)	6	-0.15(-0.29,-0.02)	-1.53(-1.69,-1.37)	-0.54(-0.57,-0.51)	-0.76(-0.83,-0.69)	197
Guam	1,156.48(967.72-1,384.32)	75	1,087.24(906.93-1,298.14)	76	-0.54(-0.76,-0.32)	-0.42(-0.49,-0.35)	0.48(0.31,0.65)	-0.17(-0.27,-0.07)	93
Monaco	1,177.95(1,026.40-1,360.13)	65	1,162.57(1,004.83-1,337.45)	48	-0.16(-0.17,-0.14)	-0.04(-0.05,-0.03)	0.07(0.05,0.09)	-0.04(-0.05,-0.03)	53
Nauru	1,086.37(942.66-1,247.65)	97	1,073.19(910.41-1,272.27)	81	-0.51(-0.54,-0.47)	-0.51(-0.54,-0.47)	1.19(0.39,1.99)	0.02(-0.23,0.26)	36
Niue	1,113.31(948.64-1,317.45)	88	1,107.18(927.35-1,305.33)	66	-1.13(-1.24,-1.01)	0.09(0.07,0.12)	1.03(0.67,1.39)	0.00(-0.12,0.12)	42
Northern Mariana Islands	1,170.25(992.48-1,385.51)	68	1,114.30(936.14-1,334.65)	63	-0.93(-1.12,-0.74)	0.03(-0.06,0.11)	0.50(0.32,0.70)	-0.13(-0.22,-0.03)	84
Palau	1,159.98(994.35-1,341.86)	74	1,064.30(908.04-1,244.13)	85	-0.64(-0.70,-0.57)	-0.41(-0.42,-0.40)	0.21(0.05,0.37)	-0.29(-0.34,-0.23)	127
Puerto Rico	1,643.37(1,333.46-1,985.12)	4	1,546.00(1,254.67-1,904.49)	2	0.11(0.05,0.18)	-0.37(-0.46,-0.27)	-0.31(-0.55,-0.06)	-0.18(-0.27,-0.10)	98
Saint Kitts and Nevis	1,268.03(1,030.05-1,548.98)	40	1,267.06(1,017.93-1,573.40)	23	-0.13(-0.14,-0.12)	-0.13(-0.14,-0.12)	0.30(0.13,0.47)	0.00(-0.05,0.06)	43
San Marino	1,170.24(1,022.46-1,342.40)	69	1,159.87(1,005.06-1,335.66)	49	-0.14(-0.15,-0.13)	-0.03(-0.03,-0.02)	0.09(0.05,0.13)	-0.03(-0.04,-0.01)	51
Tokelau	1,096.98(917.07-1,309.23)	96	1,101.79(918.09-1,300.47)	69	-1.11(-1.34,-0.88)	0.26(0.17,0.35)	1.09(0.83,1.34)	0.08(-0.04,0.21)	22
Tuvalu	1,060.06(926.02-1,207.52)	104	1,093.51(910.76-1,289.41)	75	-0.54(-0.57,-0.50)	-0.54(-0.57,-0.50)	1.78(1.01,2.56)	0.17(-0.06,0.41)	10
United States Virgin Islands	1,163.25(954.81-1,439.40)	72	1,214.28(981.53-1,503.73)	37	-0.04(-0.07,0.00)	0.10(0.09,0.11)	0.42(0.31,0.54)	0.16(0.12,0.19)	11
South Sudan	942.87(786.96-1,129.46)	128	846.53(703.75-1,024.67)	135	-0.61(-0.65,-0.58)	-0.61(-0.65,-0.58)	0.25(0.03,0.47)	-0.34(-0.42,-0.26)	144
Sudan	922.02(775.42-1,088.25)	137	893.66(749.57-1,079.49)	121	-0.13(-0.15,-0.12)	-0.42(-0.46,-0.37)	0.31(0.23,0.39)	-0.10(-0.13,-0.07)	77
**Number of incident cases**									
China	10,224,943(8,846,743-12,043,793)	1	10,768,476(9,581,576-12,231,493)	1	-0.34(-0.96,0.29)	-0.60(-0.88,-0.33)	1.94(1.46,2.42)	0.28(-0.01,0.58)	123
Democratic People's Republic of Korea	237,127(202,626-280,311)	37	266,205(236,267-300,383)	47	1.44(1.30,1.57)	-0.05(-0.21,0.10)	-0.19(-0.26,-0.13)	0.35(0.28,0.42)	120
Taiwan (Province of China)	228,122(199,855-264,996)	39	236,048(210,713-267,834)	50	1.09(0.90,1.28)	-0.08(-0.14,-0.03)	-0.75(-1.19,-0.30)	0.07(-0.08,0.23)	136
Cambodia	107,275(91,535-125,713)	70	159,372(138,513-185,528)	67	1.05(1.02,1.08)	1.17(1.13,1.20)	1.89(1.62,2.17)	1.36(1.27,1.45)	60
Indonesia	2,227,474(1,945,397-2,590,600)	5	2,794,581(2,483,544-3,169,294)	4	0.47(0.28,0.66)	1.61(1.50,1.72)	0.35(0.27,0.43)	0.77(0.69,0.85)	93
Lao People's Democratic Republic	44,233(38,143-51,198)	114	65,392(57,798-74,848)	106	1.97(1.87,2.06)	1.00(0.96,1.03)	1.13(1.03,1.24)	1.35(1.30,1.40)	62
Malaysia	184,942(161,935-212,812)	48	288,908(253,065-337,399)	44	2.58(2.51,2.65)	1.45(1.40,1.49)	0.74(0.62,0.86)	1.54(1.49,1.60)	53
Maldives	3,048(2,555-3,688)	173	4,227(3,641-4,934)	170	0.63(0.43,0.82)	0.37(0.20,0.55)	2.52(2.40,2.64)	1.10(1.00,1.21)	70
Myanmar	390,722(347,251-443,706)	28	484,687(436,845-543,044)	27	0.68(0.65,0.71)	0.62(0.61,0.64)	0.95(0.93,0.98)	0.74(0.73,0.76)	96
Philippines	1,051,577(876,247-1,299,119)	8	1,490,866(1,273,671-1,778,183)	7	1.18(1.04,1.32)	1.22(1.16,1.29)	1.17(1.15,1.18)	1.19(1.14,1.25)	67
Sri Lanka	174,908(153,666-199,439)	49	230,219(205,001-258,970)	52	0.94(0.93,0.95)	1.06(1.04,1.08)	0.88(0.86,0.91)	0.95(0.94,0.96)	82
Thailand	612,679(537,700-705,086)	20	671,563(597,680-756,889)	22	0.59(0.55,0.62)	0.06(-0.13,0.24)	0.42(0.21,0.64)	0.29(0.18,0.40)	122
Timor-Leste	9,588(8,140-11,356)	148	14,549(12,546-16,989)	147	1.10(0.97,1.23)	1.36(1.18,1.54)	1.88(1.84,1.92)	1.42(1.34,1.50)	56
Viet Nam	693,553(594,992-818,645)	17	934,360(824,879-1,071,259)	11	1.78(1.51,2.06)	0.16(0.03,0.29)	1.13(1.03,1.23)	1.05(0.93,1.17)	74
Fiji	9,256(8,038-10,680)	153	9,532(8,382-10,954)	159	0.16(0.10,0.22)	-0.25(-0.30,-0.20)	0.46(0.36,0.55)	0.11(0.06,0.15)	133
Kiribati	1,111(963-1,279)	186	1,464(1,291-1,668)	181	1.05(0.97,1.13)	0.98(0.97,1.00)	0.82(0.75,0.89)	0.95(0.92,0.99)	83
Marshall Islands	565(479-667)	194	590(506-687)	194	0.07(0.04,0.09)	-0.21(-0.24,-0.19)	0.70(0.51,0.89)	0.16(0.10,0.22)	129
Micronesia (Federated States of)	1,259(1,070-1,465)	184	1,061(902-1,236)	185	-0.50(-0.59,-0.41)	-1.20(-1.27,-1.12)	-0.06(-0.64,0.52)	-0.61(-0.80,-0.43)	171

Papua New Guinea	58,693(50,328-67,901)	104	124,519(108,835-142,061)	76	3.17(3.15,3.19)	2.18(2.10,2.26)	2.55(2.52,2.58)	2.62(2.59,2.65)	17
Samoa	1,850(1,577-2,171)	177	2,201(1,862-2,610)	177	-0.17(-0.33,-0.01)	0.23(0.17,0.28)	1.91(1.72,2.10)	0.64(0.55,0.73)	102
Solomon Islands	4,165(3,503-4,913)	167	7,617(6,444-9,138)	162	2.21(1.81,2.61)	1.61(1.50,1.73)	2.73(2.44,3.01)	2.18(2.01,2.35)	33
Tonga	1,261(1,055-1,530)	183	1,212(1,016-1,445)	183	0.03(-0.22,0.29)	-0.22(-0.32,-0.12)	-0.12(-0.33,0.08)	-0.10(-0.21,0.02)	150
Vanuatu	1,784(1,517-2,092)	178	3,253(2,650-3,908)	175	1.65(1.59,1.70)	1.65(1.59,1.70)	3.41(2.31,4.52)	2.19(1.85,2.53)	32
Armenia	37,094(31,193-43,933)	123	26,631(22,641-31,228)	137	-2.16(-2.33,-1.98)	-1.41(-1.57,-1.25)	0.25(0.18,0.31)	-1.12(-1.19,-1.04)	187
Azerbaijan	84,192(70,827-100,338)	80	92,715(79,315-108,388)	92	-0.14(-0.26,-0.02)	-0.10(-0.15,-0.05)	1.19(0.99,1.39)	0.32(0.24,0.39)	121
Georgia	54,243(47,245-62,868)	107	31,673(27,157-37,009)	134	-2.86(-3.15,-2.56)	-2.18(-2.44,-1.91)	-0.32(-0.45,-0.20)	-1.80(-1.93,-1.66)	202
Kazakhstan	172,021(146,937-200,783)	50	183,399(156,916-214,733)	63	-2.67(-2.74,-2.60)	0.94(0.80,1.08)	2.45(2.24,2.66)	0.20(0.12,0.28)	127
Kyrgyzstan	60,997(51,401-73,081)	100	77,235(64,545-92,830)	99	-0.14(-0.29,0.01)	0.42(0.37,0.46)	2.17(2.01,2.33)	0.82(0.74,0.89)	90
Mongolia	28,673(24,009-34,347)	131	37,556(31,639-44,758)	126	-0.57(-0.88,-0.25)	0.38(0.01,0.75)	3.18(3.05,3.31)	0.99(0.83,1.16)	79
Tajikistan	75,346(62,525-90,691)	87	111,872(93,086-134,566)	83	0.71(0.59,0.83)	0.89(0.66,1.12)	2.53(2.47,2.58)	1.38(1.30,1.46)	59
Turkmenistan	51,232(42,947-61,754)	108	54,277(45,334-65,278)	114	-0.38(-0.50,-0.25)	-0.58(-0.79,-0.37)	1.61(1.53,1.69)	0.21(0.12,0.29)	126
Uzbekistan	314,586(262,562-377,382)	34	404,280(340,884-482,310)	31	0.82(0.72,0.92)	0.16(-0.14,0.46)	1.63(1.57,1.70)	0.85(0.75,0.96)	88
Albania	24,199(20,746-28,846)	134	18,106(15,841-20,865)	140	-0.65(-1.10,-0.19)	-1.55(-1.90,-1.20)	-0.33(-0.73,0.07)	-0.96(-1.31,-0.62)	184
Bosnia and Herzegovina	40,464(34,693-47,557)	117	26,889(23,427-30,889)	136	-1.48(-2.03,-0.92)	-1.28(-1.50,-1.06)	-1.65(-1.71,-1.58)	-1.45(-1.65,-1.26)	196
Bulgaria	70,647(61,340-81,464)	89	46,456(40,191-53,214)	120	-2.11(-2.44,-1.77)	-1.32(-1.38,-1.26)	-0.88(-1.08,-0.68)	-1.43(-1.55,-1.30)	194
Croatia	46,472(39,986-53,585)	112	32,239(28,369-36,909)	133	-1.36(-1.47,-1.25)	-1.62(-1.67,-1.57)	-0.73(-0.85,-0.62)	-1.27(-1.33,-1.21)	190
Czechia	70,399(60,186-81,869)	90	66,390(57,528-76,111)	103	-1.12(-1.41,-0.82)	-0.14(-0.23,-0.04)	0.88(0.61,1.15)	-0.17(-0.31,-0.03)	154
Hungary	85,029(73,683-97,354)	79	66,076(57,785-75,492)	105	-0.92(-1.23,-0.61)	-0.77(-1.10,-0.44)	-0.93(-1.10,-0.77)	-0.90(-1.06,-0.75)	182
North Macedonia	22,519(18,712-27,037)	136	19,699(17,296-22,262)	139	0.20(-0.07,0.46)	-1.49(-1.90,-1.09)	0.19(0.11,0.27)	-0.47(-0.69,-0.25)	165
Montenegro	4,731(3,967-5,610)	161	4,393(3,742-5,189)	169	-0.50(-0.52,-0.48)	-0.50(-0.52,-0.48)	0.40(0.07,0.74)	-0.22(-0.32,-0.12)	157
Poland	527,700(452,169-614,610)	21	348,098(299,130-407,340)	39	-1.26(-1.46,-1.07)	-2.10(-2.18,-2.03)	-0.97(-1.04,-0.89)	-1.48(-1.56,-1.40)	198
Romania	189,844(161,440-225,205)	47	134,582(114,731-158,271)	73	-1.11(-1.25,-0.98)	-1.38(-1.45,-1.31)	-1.08(-1.23,-0.93)	-1.19(-1.27,-1.11)	188
Serbia	68,486(59,970-78,753)	92	56,404(49,655-64,221)	112	-0.41(-0.88,0.06)	-1.07(-1.16,-0.98)	-0.56(-0.69,-0.43)	-0.71(-0.86,-0.55)	177
Slovakia	37,488(32,158-43,828)	120	33,271(28,866-38,379)	132	-0.73(-0.91,-0.56)	-0.51(-0.57,-0.45)	0.09(-0.06,0.25)	-0.40(-0.49,-0.32)	161
Slovenia	19,388(16,861-22,469)	140	16,575(14,362-19,181)	143	-0.91(-1.03,-0.80)	-0.75(-0.82,-0.68)	0.06(0.01,0.11)	-0.54(-0.59,-0.50)	169
Belarus	98,955(84,442-116,450)	74	66,134(56,585-78,252)	104	-1.95(-2.11,-1.79)	-2.31(-2.39,-2.22)	0.27(0.13,0.42)	-1.38(-1.46,-1.29)	192
Estonia	12,215(10,657-13,905)	146	8,127(7,095-9,283)	161	-2.67(-3.14,-2.20)	-2.06(-2.15,-1.97)	0.28(0.16,0.41)	-1.45(-1.61,-1.29)	197
Latvia	21,574(18,826-24,760)	138	11,593(9,895-13,593)	153	-3.04(-3.14,-2.93)	-2.14(-2.17,-2.12)	-1.02(-1.08,-0.95)	-2.11(-2.15,-2.07)	204
Lithuania	26,816(23,230-30,817)	132	16,561(14,245-19,120)	144	-1.91(-1.97,-1.85)	-2.15(-2.60,-1.70)	-0.96(-1.18,-0.74)	-1.69(-1.85,-1.52)	200
Republic of Moldova	36,457(30,769-43,838)	125	21,574(18,321-25,452)	138	-2.20(-2.31,-2.08)	-2.30(-2.36,-2.24)	-0.83(-0.93,-0.72)	-1.80(-1.86,-1.74)	203
Russian Federation	1,496,268(1,285,428-1,759,934)	7	977,895(837,720-1,145,889)	10	-2.25(-2.52,-1.98)	-2.26(-2.34,-2.18)	0.29(0.06,0.51)	-1.43(-1.56,-1.31)	195
Ukraine	470,705(391,683-562,514)	24	280,548(234,124-336,784)	46	-2.33(-2.57,-2.08)	-2.72(-2.87,-2.58)	0.03(-0.27,0.33)	-1.73(-1.87,-1.58)	201
Brunei Darussalam	3,142(2,660-3,740)	172	4,204(3,626-4,877)	171	1.80(1.73,1.87)	0.45(0.39,0.51)	0.89(0.85,0.94)	1.01(0.97,1.05)	76
Japan	1,788,610(1,572,865-2,047,499)	6	1,210,790(1,082,180-1,357,791)	9	-1.43(-1.56,-1.30)	-2.30(-2.44,-2.16)	-0.25(-0.34,-0.16)	-1.39(-1.47,-1.32)	193
Republic of Korea	435,437(380,428-501,616)	26	433,965(386,106-488,346)	29	0.02(-0.30,0.33)	-0.50(-0.57,-0.43)	0.60(0.52,0.68)	0.00(-0.10,0.10)	141
Singapore	31,228(27,052-35,898)	128	42,732(37,246-49,388)	123	1.71(1.44,1.98)	0.29(0.14,0.45)	1.38(0.99,1.77)	1.08(0.91,1.25)	72

Australia	191,915(163,411-221,183)	46	234,875(203,629-272,194)	51	1.33(1.15,1.52)	-0.45(-0.57,-0.32)	1.19(1.13,1.25)	0.71(0.63,0.79)	99
New Zealand	51,123(43,863-59,582)	109	51,547(44,341-60,189)	115	-0.15(-0.30,0.00)	0.18(0.11,0.25)	0.12(0.02,0.22)	0.01(-0.06,0.08)	140
Andorra	559(495-629)	195	775(691-867)	189	1.70(0.50,2.91)	2.00(0.92,3.09)	-0.52(-0.85,-0.19)	0.96(0.43,1.50)	81
Austria	87,434(78,771-96,855)	78	86,754(78,035-96,207)	96	-0.16(-0.23,-0.09)	-0.15(-0.17,-0.12)	0.33(0.26,0.40)	-0.03(-0.07,0.00)	143
Belgium	115,945(104,501-128,910)	67	110,601(99,106-122,955)	84	-1.21(-1.38,-1.03)	0.58(0.50,0.66)	0.19(0.10,0.29)	-0.20(-0.27,-0.12)	155
Cyprus	9,315(8,127-10,652)	152	13,843(12,240-15,661)	149	0.91(0.88,0.93)	1.55(1.47,1.63)	1.73(1.69,1.77)	1.39(1.36,1.42)	58
Denmark	61,057(55,127-67,920)	99	62,094(55,148-69,540)	109	0.45(0.32,0.58)	-0.10(-0.15,-0.05)	-0.14(-0.44,0.16)	0.06(-0.05,0.16)	138
Finland	55,908(50,390-61,902)	106	58,482(52,821-64,710)	111	0.41(0.36,0.46)	0.29(0.26,0.31)	-0.27(-0.30,-0.24)	0.14(0.12,0.17)	131
France	807,039(727,937-902,606)	14	789,638(708,298-882,777)	17	-0.50(-0.57,-0.44)	0.19(0.12,0.26)	0.02(-0.19,0.22)	-0.10(-0.17,-0.03)	151
Germany	892,278(789,638-984,465)	11	737,648(664,296-820,037)	21	-1.89(-2.02,-1.77)	-0.28(-0.39,-0.16)	0.13(-0.02,0.28)	-0.66(-0.74,-0.58)	174
Greece	104,934(93,291-118,174)	71	93,318(83,128-104,013)	91	-0.08(-0.10,-0.06)	-0.13(-0.17,-0.08)	-1.07(-1.18,-0.96)	-0.41(-0.45,-0.37)	164
Iceland	3,897(3,373-4,492)	168	4,417(3,862-5,068)	168	0.58(0.46,0.71)	0.19(0.15,0.24)	0.48(0.38,0.57)	0.43(0.37,0.49)	113
Ireland	49,589(43,633-56,501)	110	55,277(48,711-62,245)	113	-0.51(-0.54,-0.47)	1.39(1.30,1.48)	0.08(0.04,0.11)	0.38(0.33,0.42)	117
Israel	60,727(53,111-69,125)	101	104,211(91,109-118,432)	88	1.96(1.84,2.09)	1.73(1.69,1.78)	1.94(1.89,1.99)	1.87(1.83,1.91)	47
Italy	658,020(591,039-730,518)	18	570,773(512,850-630,811)	24	-0.66(-0.89,-0.43)	-0.27(-0.57,0.03)	-0.54(-0.68,-0.40)	-0.52(-0.66,-0.39)	168
Luxembourg	4,563(4,049-5,373)	165	6,454(5,779-7,209)	163	1.24(1.19,1.29)	0.63(0.58,0.68)	1.81(1.77,1.84)	1.18(1.15,1.21)	68
Malta	4,724(4,219-5,276)	162	4,616(4,131-5,154)	167	0.05(0.01,0.10)	-0.37(-0.38,-0.35)	0.10(0.03,0.17)	-0.09(-0.11,-0.06)	148
Netherlands	146,367(133,286-159,965)	55	156,751(142,463-171,304)	68	0.54(0.46,0.63)	-0.19(-0.26,-0.13)	0.32(0.28,0.36)	0.23(0.19,0.27)	124
Norway	63,409(55,955-71,566)	95	62,815(55,430-71,248)	108	-0.06(-0.17,0.06)	-0.21(-0.26,-0.16)	0.27(0.20,0.34)	-0.04(-0.09,0.01)	145
Portugal	135,534(119,758-154,432)	58	118,714(106,597-131,471)	77	-0.65(-0.94,-0.36)	0.29(0.12,0.45)	-0.90(-1.00,-0.81)	-0.50(-0.62,-0.38)	167
Spain	395,506(351,443-444,933)	27	442,054(395,323-492,744)	28	-0.40(-0.47,-0.33)	1.95(1.87,2.03)	-0.48(-0.56,-0.39)	0.38(0.34,0.42)	118
Sweden	123,870(109,286-138,790)	62	117,354(103,973-132,367)	78	-1.77(-1.98,-1.56)	-0.29(-0.69,0.11)	1.17(0.18,2.17)	-0.33(-0.68,0.02)	160
Switzerland	80,423(72,033-89,551)	83	90,008(80,411-100,265)	94	0.16(0.10,0.22)	-0.03(-0.10,0.03)	1.06(1.02,1.11)	0.37(0.33,0.40)	119
United Kingdom	898,204(793,326-1,016,858)	10	874,675(775,210-988,347)	14	-0.53(-0.81,-0.24)	0.11(0.07,0.16)	0.11(0.07,0.16)	-0.16(-0.26,-0.05)	152
Argentina	435,448(378,992-507,939)	25	545,391(468,386-640,857)	25	0.86(0.79,0.93)	0.88(0.86,0.89)	0.58(0.53,0.63)	0.78(0.75,0.81)	92
Chile	158,456(137,150-185,429)	52	192,401(167,855-221,623)	61	0.67(0.45,0.90)	-0.19(-0.33,-0.05)	1.48(1.31,1.65)	0.65(0.55,0.76)	101
Uruguay	39,071(34,077-45,134)	119	37,349(32,553-43,199)	127	0.01(-0.05,0.08)	-0.54(-0.60,-0.48)	0.13(0.07,0.18)	-0.16(-0.20,-0.12)	153
Canada	307,605(265,554-358,268)	35	364,788(319,565-418,535)	37	0.66(0.45,0.87)	0.96(0.88,1.04)	0.20(-0.18,0.57)	0.63(0.49,0.77)	103
United States of America	4,353,766(3,643,079-5,316,502)	3	5,545,294(4,814,147-6,415,919)	3	-1.32(-1.65,-0.99)	2.36(2.13,2.59)	1.24(1.04,1.44)	0.87(0.71,1.04)	85
Antigua and Barbuda	779(633-972)	189	869(714-1,046)	188	1.72(1.40,2.04)	-0.08(-0.17,0.01)	-0.42(-0.64,-0.20)	0.42(0.29,0.56)	114
Bahamas	3,280(2,668-4,079)	170	3,819(3,130-4,616)	173	1.88(1.69,2.07)	0.41(0.25,0.56)	-0.44(-0.59,-0.29)	0.57(0.47,0.66)	107
Barbados	3,036(2,546-3,651)	174	3,052(2,580-3,624)	176	-0.42(-0.54,-0.30)	0.89(0.84,0.94)	-0.46(-0.50,-0.42)	0.02(-0.03,0.06)	139
Belize	3,196(2,547-3,946)	171	5,215(4,223-6,376)	166	1.30(1.13,1.48)	2.43(2.14,2.71)	1.38(1.25,1.52)	1.68(1.56,1.79)	51
Cuba	140,047(115,056-169,727)	56	113,748(95,379-134,548)	80	-0.75(-0.82,-0.67)	-1.11(-1.42,-0.80)	-0.45(-0.79,-0.10)	-0.75(-0.90,-0.60)	179
Dominica	1,055(865-1,288)	187	773(645-927)	190	-0.56(-0.61,-0.52)	-1.14(-1.30,-0.98)	-1.41(-1.50,-1.31)	-1.04(-1.10,-0.98)	186
Dominican Republic	92,803(75,781-111,931)	77	112,067(92,417-136,646)	82	0.08(-0.08,0.25)	0.83(0.63,1.03)	1.26(1.01,1.50)	0.72(0.59,0.84)	98
Grenada	1,378(1,094-1,716)	182	1,226(1,017-1,495)	182	0.42(0.26,0.58)	-0.63(-0.68,-0.59)	-1.01(-1.12,-0.90)	-0.40(-0.46,-0.33)	162
Guyana	11,777(9,608-14,418)	147	9,732(7,997-11,804)	157	-0.08(-0.22,0.06)	-1.74(-1.83,-1.64)	-0.01(-0.22,0.20)	-0.63(-0.72,-0.54)	173

Haiti	129,197(104,024-156,403)	59	197,544(162,055-238,849)	58	1.69(1.54,1.85)	1.28(1.24,1.31)	1.55(1.44,1.67)	1.49(1.43,1.56)	54
Jamaica	37,289(30,063-46,266)	121	30,049(24,789-36,427)	135	0.01(-0.14,0.17)	-1.32(-1.42,-1.22)	-0.74(-0.87,-0.61)	-0.73(-0.81,-0.64)	178
Saint Lucia	2,224(1,801-2,730)	176	1,814(1,520-2,144)	179	0.27(0.15,0.39)	-1.26(-1.32,-1.21)	-1.04(-1.13,-0.96)	-0.69(-0.76,-0.62)	175
Saint Vincent and the Grenadines	1,616(1,303-2,009)	179	1,209(999-1,463)	184	-1.16(-1.17,-1.14)	-1.16(-1.17,-1.14)	-0.58(-0.83,-0.32)	-0.98(-1.06,-0.90)	185
Suriname	5,242(4,234-6,554)	160	6,434(5,260-7,861)	164	0.46(0.21,0.70)	1.05(0.96,1.14)	0.69(0.62,0.76)	0.77(0.67,0.87)	94
Trinidad and Tobago	14,089(11,526-17,260)	144	12,468(10,298-15,154)	151	-1.52(-1.57,-1.46)	-0.44(-0.48,-0.41)	0.92(0.66,1.18)	-0.40(-0.48,-0.31)	163
Bolivia (Plurinational State of)	113,846(90,084-140,050)	68	154,056(123,754-194,547)	69	1.21(1.08,1.34)	0.14(0.07,0.21)	2.00(1.87,2.14)	1.07(0.99,1.14)	73
Ecuador	158,024(124,598-196,949)	53	193,356(155,358-242,420)	59	0.25(-0.05,0.55)	0.31(0.20,0.42)	1.68(1.58,1.77)	0.70(0.58,0.82)	100
Peru	355,592(277,483-445,672)	32	364,093(292,650-459,246)	38	-0.09(-0.20,0.02)	-1.34(-1.58,-1.10)	1.96(1.86,2.07)	0.08(-0.04,0.21)	135
Colombia	373,988(299,569-465,273)	31	366,733(296,217-451,110)	35	0.41(0.10,0.72)	-0.18(-0.22,-0.14)	-0.18(-0.22,-0.14)	-0.07(-0.19,0.05)	147
Costa Rica	43,868(34,856-54,475)	115	45,446(36,970-55,803)	121	2.10(2.04,2.16)	-1.58(-1.75,-1.42)	-0.04(-0.11,0.03)	0.13(0.06,0.19)	132
El Salvador	95,831(74,709-120,583)	75	67,221(54,573-83,752)	102	-0.43(-0.56,-0.30)	-2.52(-2.62,-2.42)	-0.44(-0.53,-0.34)	-1.24(-1.31,-1.17)	189
Guatemala	126,959(99,587-156,863)	61	152,848(121,789-192,306)	71	1.05(0.83,1.26)	-0.28(-0.74,0.17)	1.17(1.11,1.23)	0.62(0.45,0.79)	104
Honduras	78,439(60,936-98,151)	85	101,412(80,961-127,037)	89	1.38(1.17,1.59)	0.21(0.16,0.26)	1.23(1.02,1.44)	0.89(0.79,0.99)	84
Mexico	878,069(699,839-1,118,961)	12	924,598(732,794-1,147,955)	12	-0.34(-0.56,-0.12)	0.14(-0.04,0.32)	1.00(0.94,1.05)	0.20(0.09,0.30)	128
Nicaragua	65,207(50,650-82,824)	93	61,612(48,953-77,296)	110	-0.20(-0.40,0.01)	-0.40(-0.56,-0.24)	0.04(-0.10,0.18)	-0.23(-0.33,-0.13)	158
Panama	30,468(24,602-37,336)	129	40,660(32,614-50,480)	124	1.08(0.98,1.19)	0.60(0.53,0.66)	1.44(1.33,1.54)	1.01(0.95,1.07)	77
Venezuela (Bolivarian Republic of)	233,313(184,955-292,810)	38	238,150(192,222-292,107)	49	0.80(0.49,1.10)	0.26(0.17,0.36)	-1.04(-1.30,-0.77)	-0.02(-0.16,0.13)	142
Brazil	2,470,862(1,941,393-3,138,864)	4	2,465,487(1,968,598-3,012,172)	5	-0.07(-0.22,0.09)	-0.60(-0.66,-0.53)	0.71(0.55,0.87)	-0.03(-0.11,0.05)	144
Paraguay	63,935(50,396-81,129)	94	92,193(73,706-115,264)	93	1.89(1.72,2.07)	1.00(0.98,1.02)	1.00(0.98,1.02)	1.28(1.22,1.33)	64
Algeria	223,097(185,764-271,230)	41	328,903(273,714-398,370)	41	0.90(0.68,1.11)	0.99(0.89,1.10)	2.34(2.21,2.48)	1.36(1.27,1.46)	61
Bahrain	4,675(3,876-5,656)	164	9,504(7,997-11,211)	160	1.92(1.83,2.01)	4.60(4.29,4.92)	0.75(0.64,0.86)	2.46(2.32,2.60)	23
Egypt	491,482(397,075-604,052)	23	752,686(614,049-921,930)	20	0.59(0.52,0.67)	1.60(1.38,1.83)	2.25(2.20,2.31)	1.48(1.40,1.55)	55
Iran (Islamic Republic of)	620,403(498,943-764,078)	19	633,257(533,798-754,359)	23	-0.04(-0.17,0.08)	-0.52(-0.56,-0.48)	0.90(0.85,0.94)	0.07(0.02,0.11)	137
Iraq	202,572(163,238-248,882)	45	365,155(297,870-452,588)	36	3.62(3.23,4.02)	1.12(0.41,1.84)	1.55(1.43,1.67)	2.09(1.82,2.35)	37
Jordan	40,110(32,122-50,668)	118	107,129(86,490-132,277)	85	1.77(1.68,1.86)	3.85(3.14,4.57)	4.79(4.65,4.93)	3.45(3.22,3.68)	5
Kuwait	16,856(13,784-20,406)	143	34,162(27,999-41,146)	130	-0.45(-0.78,-0.12)	3.87(3.67,4.08)	3.94(3.72,4.17)	2.50(2.34,2.65)	21
Lebanon	33,773(27,997-41,280)	126	47,120(39,274-56,338)	119	0.24(0.14,0.33)	0.52(0.24,0.80)	2.70(1.99,3.41)	1.10(0.86,1.35)	71
Libya	43,391(34,888-52,993)	116	50,222(42,502-58,902)	116	0.52(0.42,0.63)	0.84(0.79,0.89)	0.15(0.10,0.21)	0.51(0.46,0.55)	111
Morocco	203,812(170,676-242,738)	44	258,857(219,701-304,756)	48	1.15(1.10,1.20)	0.71(0.68,0.74)	0.69(0.63,0.76)	0.84(0.81,0.87)	89
Palestine	21,173(17,034-26,369)	139	44,510(35,838-55,049)	122	3.04(2.73,3.34)	2.93(2.84,3.02)	1.85(1.72,1.98)	2.63(2.52,2.73)	16
Oman	17,236(14,145-21,244)	141	36,809(30,489-44,762)	128	0.69(0.51,0.86)	2.01(1.15,2.87)	4.92(3.94,5.91)	2.58(2.16,3.00)	18
Qatar	3,566(2,915-4,409)	169	18,080(15,162-21,416)	141	2.35(2.16,2.54)	7.99(7.41,8.58)	6.98(6.73,7.24)	5.83(5.61,6.05)	1
Saudi Arabia	120,456(99,525-145,750)	64	217,842(184,391-256,805)	54	1.80(1.64,1.97)	2.26(1.51,3.01)	1.86(1.76,1.95)	1.96(1.72,2.21)	44
Syrian Arab Republic	127,029(104,919-154,802)	60	112,794(94,899-133,012)	81	0.48(0.09,0.88)	3.17(2.56,3.78)	-5.87(-7.10,-4.63)	-0.61(-1.09,-0.13)	172
Tunisia	76,469(63,837-92,650)	86	89,314(76,190-105,380)	95	0.81(0.75,0.87)	0.07(0.03,0.12)	0.83(0.69,0.97)	0.54(0.49,0.59)	110
Turkey	724,926(614,052-856,577)	16	806,353(706,387-924,162)	16	0.97(0.85,1.09)	-1.16(-1.49,-0.84)	1.64(1.50,1.78)	0.39(0.26,0.52)	115
United Arab Emirates	22,125(18,285-26,641)	137	82,042(68,683-97,957)	98	4.75(4.47,5.04)	9.65(7.52,11.82)	-0.38(-1.94,1.21)	4.52(3.61,5.45)	2

Yemen	160,748(130,979-197,888)	51	305,975(247,066-376,327)	43	2.23(2.10,2.36)	2.41(2.38,2.45)	2.08(1.91,2.26)	2.24(2.17,2.32)	29
Afghanistan	121,453(102,051-144,513)	63	402,588(329,942-493,979)	32	6.36(4.92,7.82)	3.33(3.21,3.45)	3.33(3.21,3.45)	4.21(3.74,4.69)	3
Bangladesh	746,946(659,941-855,852)	15	843,315(751,280-949,219)	15	0.99(0.86,1.11)	0.00(-0.04,0.05)	0.25(0.17,0.32)	0.39(0.34,0.45)	116
Bhutan	4,222(3,728-4,838)	166	3,919(3,510-4,420)	172	-0.19(-1.08,0.72)	-0.15(-0.29,-0.01)	-0.49(-0.86,-0.11)	-0.26(-0.58,0.05)	159
India	6,741,743(5,920,559-7,818,255)	2	9,168,248(8,096,198-10,402,187)	2	-0.49(-1.06,0.09)	3.03(2.19,3.87)	0.54(0.18,0.91)	1.01(0.67,1.35)	78
Nepal	119,647(106,766-135,747)	65	153,914(137,431-173,340)	70	1.61(1.54,1.67)	0.42(0.34,0.50)	0.66(0.59,0.73)	0.87(0.82,0.92)	86
Pakistan	852,534(747,917-981,452)	13	1,445,432(1,282,564-1,650,686)	8	2.26(2.15,2.37)	1.85(1.83,1.88)	1.34(1.22,1.47)	1.86(1.81,1.92)	48
Angola	118,833(96,611-144,576)	66	283,538(226,819-352,190)	45	3.45(3.42,3.47)	2.95(2.93,2.98)	2.72(2.64,2.80)	3.05(3.02,3.08)	11
Central African Republic	29,445(24,163-35,699)	130	48,896(40,145-59,619)	117	2.32(2.28,2.37)	1.83(1.78,1.88)	1.05(0.89,1.21)	1.76(1.70,1.81)	50
Congo	26,065(21,103-32,095)	133	47,146(37,958-58,574)	118	1.95(1.90,2.00)	1.92(1.77,2.07)	2.31(2.27,2.34)	2.08(2.03,2.13)	38
Democratic Republic of the Congo	386,056(308,297-476,662)	29	759,733(618,196-937,655)	19	2.24(2.22,2.27)	2.53(2.49,2.57)	2.33(2.29,2.37)	2.36(2.34,2.38)	27
Equatorial Guinea	4,695(3,763-5,762)	163	11,722(9,327-14,723)	152	3.87(3.80,3.94)	3.19(3.14,3.23)	2.65(2.58,2.71)	3.23(3.19,3.28)	9
Gabon	9,034(7,360-11,056)	154	12,815(10,394-15,761)	150	1.38(1.36,1.40)	0.88(0.86,0.89)	1.41(1.29,1.52)	1.21(1.17,1.25)	65
Burundi	74,721(59,613-93,054)	88	130,114(102,931-161,507)	75	0.54(0.32,0.76)	3.04(2.75,3.34)	2.47(1.74,3.20)	2.01(1.75,2.27)	40
Comoros	5,446(4,362-6,801)	159	6,391(5,183-7,841)	165	0.61(0.58,0.65)	0.42(0.40,0.44)	0.66(0.62,0.70)	0.55(0.53,0.57)	109
Djibouti	6,136(4,857-7,701)	158	11,220(9,150-14,025)	155	1.37(1.12,1.62)	2.24(1.97,2.52)	2.71(2.63,2.78)	2.15(2.03,2.27)	35
Eritrea	36,715(29,809-45,281)	124	67,275(53,973-83,632)	101	3.03(2.84,3.22)	2.07(1.95,2.19)	1.31(1.13,1.48)	2.14(2.01,2.26)	36
Ethiopia	526,390(418,961-667,332)	22	920,194(711,280-1,185,181)	13	1.87(1.72,2.03)	1.57(1.49,1.65)	2.69(2.47,2.92)	2.01(1.92,2.10)	41
Kenya	217,808(168,703-281,643)	43	379,630(297,974-482,528)	34	1.69(1.50,1.89)	2.03(1.91,2.15)	2.25(2.02,2.49)	1.98(1.86,2.10)	42
Madagascar	224,614(180,275-286,350)	40	333,457(269,547-409,338)	40	2.15(2.01,2.29)	0.59(0.43,0.75)	1.43(1.32,1.54)	1.33(1.24,1.41)	63
Malawi	103,714(82,102-129,527)	72	178,595(141,627-223,594)	64	0.70(0.37,1.03)	2.29(2.26,2.32)	2.61(2.48,2.74)	1.89(1.78,2.01)	45
Mauritius	12,311(10,694-14,197)	145	11,592(10,343-13,019)	154	0.52(0.47,0.56)	-0.50(-0.52,-0.48)	-0.58(-0.64,-0.52)	-0.21(-0.24,-0.19)	156
Mozambique	152,682(122,367-190,954)	54	327,611(259,686-415,048)	42	2.89(2.39,3.39)	2.29(2.22,2.36)	2.83(2.75,2.90)	2.65(2.49,2.81)	15
Rwanda	135,684(107,487-173,699)	57	171,083(135,937-213,654)	65	-0.42(-3.63,2.91)	1.03(0.81,1.26)	1.03(0.81,1.26)	0.58(-0.45,1.62)	106
Seychelles	706(614-817)	190	903(796-1,039)	187	0.59(0.53,0.64)	0.85(0.84,0.87)	1.17(1.12,1.22)	0.87(0.84,0.89)	87
Somalia	94,676(74,494-117,286)	76	228,009(184,092-286,055)	53	3.19(3.01,3.36)	2.77(2.69,2.84)	3.45(3.37,3.52)	3.11(3.04,3.18)	10
United Republic of Tanzania	374,332(294,714-473,508)	30	773,777(610,277-972,356)	18	2.52(2.41,2.63)	3.24(3.08,3.40)	1.96(1.75,2.18)	2.53(2.44,2.63)	19
Uganda	251,145(198,712-320,297)	36	517,065(401,424-658,818)	26	3.05(3.02,3.08)	2.33(2.29,2.37)	2.28(2.22,2.33)	2.53(2.50,2.55)	20
Zambia	63,122(50,993-78,068)	97	139,640(110,911-174,637)	72	1.36(1.13,1.58)	3.04(2.92,3.16)	4.18(3.94,4.42)	2.84(2.71,2.97)	14
Botswana	8,759(7,194-10,547)	155	14,953(12,045-18,710)	146	1.84(1.63,2.04)	1.64(1.08,2.20)	1.95(1.80,2.10)	1.77(1.58,1.97)	49
Lesotho	9,370(7,931-11,050)	151	9,643(8,337-11,146)	158	0.02(-0.07,0.12)	-0.40(-0.46,-0.34)	0.75(0.66,0.84)	0.10(0.05,0.15)	134
Namibia	9,452(7,857-11,394)	149	14,166(11,732-17,020)	148	2.21(2.05,2.37)	0.62(0.56,0.68)	1.48(1.39,1.57)	1.41(1.34,1.47)	57
South Africa	351,927(271,099-444,844)	33	419,275(326,299-526,850)	30	2.23(1.57,2.89)	-4.34(-6.19,-2.46)	5.61(4.80,6.44)	1.02(0.30,1.75)	75
Eswatini	8,243(6,649-10,296)	157	9,745(7,957-11,704)	156	2.25(1.93,2.56)	-0.40(-0.58,-0.23)	-0.13(-0.24,-0.02)	0.56(0.43,0.69)	108
Zimbabwe	62,986(51,666-76,848)	98	86,745(72,409-104,297)	97	0.48(0.42,0.53)	0.73(0.62,0.84)	2.24(2.14,2.33)	1.11(1.06,1.17)	69
Benin	44,689(35,762-55,807)	113	104,460(82,930-130,567)	87	2.61(2.47,2.75)	2.94(2.90,2.99)	3.48(3.38,3.57)	2.98(2.92,3.04)	12
Burkina Faso	83,011(66,381-102,800)	82	189,301(150,505-240,084)	62	2.17(2.12,2.21)	3.08(2.96,3.20)	3.49(3.42,3.56)	2.89(2.84,2.93)	13
Cameroon	83,858(69,162-103,392)	81	213,297(171,688-266,029)	56	2.71(2.64,2.78)	4.09(4.00,4.19)	3.16(3.09,3.22)	3.29(3.25,3.34)	8

Cabo Verde	2,952(2,363-3,659)	175	3,496(2,874-4,295)	174	1.11(1.02,1.19)	0.00(-0.09,0.08)	0.79(0.68,0.89)	0.59(0.53,0.65)	105
Chad	46,702(38,037-57,193)	111	130,256(103,658-164,144)	74	2.89(2.85,2.92)	3.86(3.79,3.93)	4.15(4.07,4.22)	3.61(3.57,3.64)	4
C??te d'Ivoire	110,224(88,941-138,525)	69	217,564(175,044-272,646)	55	3.14(2.86,3.43)	1.84(1.73,1.94)	2.18(1.95,2.40)	2.39(2.26,2.52)	26
Gambia	8,674(6,926-10,875)	156	17,391(13,987-21,447)	142	2.32(2.16,2.47)	2.46(2.42,2.50)	2.60(2.48,2.72)	2.43(2.36,2.50)	25
Ghana	102,040(81,310-127,777)	73	192,666(156,136-237,137)	60	1.52(1.45,1.58)	2.43(2.38,2.49)	2.76(2.65,2.87)	2.24(2.19,2.29)	30
Guinea	60,388(48,997-76,179)	102	115,879(93,303-147,428)	79	2.86(2.17,3.55)	1.24(1.10,1.38)	2.66(2.56,2.76)	2.20(1.98,2.43)	31
Guinea-Bissau	9,412(7,658-11,574)	150	15,303(12,380-18,721)	145	1.48(1.38,1.58)	1.53(1.47,1.59)	2.11(2.05,2.17)	1.68(1.63,1.72)	52
Liberia	17,224(13,714-21,590)	142	34,054(27,167-42,581)	131	2.83(0.96,4.74)	2.46(2.17,2.75)	1.60(1.08,2.11)	2.31(1.69,2.93)	28
Mali	63,142(52,102-77,873)	96	164,334(133,100-205,732)	66	2.07(1.58,2.56)	3.71(3.63,3.80)	4.30(3.98,4.61)	3.38(3.19,3.57)	7
Mauritania	23,163(18,337-29,462)	135	39,778(31,795-50,355)	125	1.96(1.91,2.01)	2.03(2.02,2.05)	1.67(1.64,1.70)	1.88(1.86,1.90)	46
Niger	80,227(64,096-100,290)	84	211,162(167,642-267,839)	57	2.98(2.96,3.01)	3.54(3.50,3.58)	3.77(3.72,3.81)	3.41(3.39,3.43)	6
Nigeria	994,196(792,681-1,255,648)	9	2,048,973(1,626,075-2,614,328)	6	2.39(2.11,2.68)	3.16(2.93,3.39)	2.15(2.06,2.23)	2.50(2.37,2.62)	22
Sao Tome and Principe	1,389(1,120-1,717)	181	1,758(1,443-2,115)	180	1.44(1.11,1.77)	0.35(0.30,0.40)	0.63(0.57,0.69)	0.79(0.68,0.89)	91
Senegal	59,774(48,905-73,938)	103	105,257(85,156-130,676)	86	1.51(1.42,1.61)	2.09(2.06,2.12)	2.40(2.32,2.49)	1.98(1.94,2.03)	43
Sierra Leone	32,821(26,638-40,889)	127	64,952(52,451-80,844)	107	1.05(0.81,1.29)	3.36(2.98,3.74)	2.69(2.46,2.93)	2.44(2.26,2.63)	24
Togo	37,138(29,431-46,929)	122	68,757(54,977-85,885)	100	2.30(2.19,2.41)	1.93(1.83,2.04)	2.33(2.26,2.40)	2.16(2.11,2.22)	34
American Samoa	579(483-700)	192	602(503-716)	192	0.84(0.61,1.08)	-0.28(-0.52,-0.05)	0.12(-0.92,1.16)	0.23(-0.11,0.57)	125
Bermuda	613(509-737)	191	528(442-620)	195	0.11(0.06,0.16)	-0.31(-0.41,-0.21)	-1.29(-1.48,-1.10)	-0.48(-0.55,-0.41)	166
Cook Islands	232(193-281)	199	193(161-226)	199	-1.41(-1.62,-1.20)	-0.24(-0.37,-0.12)	-0.07(-0.32,0.18)	-0.58(-0.71,-0.46)	170
Greenland	922(765-1,110)	188	729(632-853)	191	0.25(-0.42,0.91)	-1.87(-2.04,-1.69)	-0.78(-0.86,-0.69)	-0.87(-1.09,-0.65)	181
Guam	1,597(1,329-1,930)	180	1,824(1,530-2,168)	178	1.34(1.19,1.50)	-0.53(-0.68,-0.38)	0.92(0.69,1.16)	0.51(0.40,0.63)	112
Monaco	289(261-321)	197	359(322-400)	197	0.86(0.81,0.90)	0.70(0.65,0.76)	0.72(0.68,0.76)	0.76(0.73,0.78)	95
Nauru	123(104-148)	201	119(98-143)	202	-0.44(-0.59,-0.29)	-0.77(-0.88,-0.66)	1.19(0.27,2.11)	-0.05(-0.34,0.24)	146
Niue	27(23-33)	203	18(15-21)	203	-3.10(-3.26,-2.95)	-2.14(-2.32,-1.96)	1.56(0.93,2.20)	-1.28(-1.51,-1.04)	191
Northern Mariana Islands	511(431-607)	196	417(353-488)	196	3.35(3.01,3.68)	-3.00(-3.13,-2.87)	-2.82(-3.07,-2.57)	-0.69(-0.86,-0.52)	176
Palau	178(151-207)	200	171(149-196)	200	1.63(1.49,1.77)	-1.34(-1.70,-0.98)	-0.49(-0.66,-0.31)	-0.09(-0.22,0.05)	149
Puerto Rico	57,498(46,711-69,637)	105	36,419(30,697-43,275)	129	0.12(0.03,0.21)	-1.81(-1.93,-1.68)	-3.13(-3.37,-2.89)	-1.60(-1.69,-1.51)	199
Saint Kitts and Nevis	577(462-713)	193	596(490-727)	193	0.04(-0.17,0.25)	0.30(0.23,0.38)	0.16(-0.07,0.38)	0.16(0.04,0.27)	130
San Marino	244(218-276)	198	324(288-364)	198	1.36(1.34,1.37)	1.12(1.06,1.18)	0.52(0.49,0.55)	0.99(0.97,1.02)	80
Tokelau	21(17-26)	204	16(14-20)	204	-2.29(-3.14,-1.42)	-2.80(-3.60,-2.00)	1.91(1.45,2.37)	-0.95(-1.39,-0.52)	183
Tuvalu	104(90-121)	202	127(106-150)	201	-0.46(-0.58,-0.35)	0.35(0.27,0.44)	2.53(1.81,3.26)	0.74(0.52,0.97)	97
United States Virgin Islands	1,258(1,028-1,560)	185	991(824-1,191)	186	-0.34(-0.38,-0.29)	-1.16(-1.18,-1.13)	-0.87(-1.04,-0.70)	-0.81(-0.87,-0.76)	180
South Sudan	68,738(55,252-86,177)	91	97,351(77,174-122,037)	90	1.54(1.35,1.74)	1.97(1.92,2.01)	-0.07(-0.58,0.44)	1.20(1.03,1.37)	66
Sudan	219,167(174,376-272,157)	42	400,310(325,216-499,438)	33	2.92(2.79,3.05)	1.51(1.38,1.65)	1.95(1.76,2.14)	2.08(1.98,2.17)	39

sTable 4. The incident cases and age-standardized rate of incidence attributable to six immune mediated inflammatory diseases according to SDI regions and its temporal trends from 1990 to 2019.

	1990		2019		1990-1999	2000-2009	2010-2019	1990-2019	
	**N(95%CI)**	**Rank**	**N(95%CI)**	**Rank**	**AAPC(95%CI)**	**AAPC(95%CI)**	**AAPC(95%CI)**	**AAPC(95%CI)**	**Rank**
**Age standardized rate**									
Asthma									
High-middle SDI	559.79(446.64-704.44)	3	474.27(358.92-614.35)	4	-1.15(-1.58,-0.72)	-1.29(-1.58,-1.01)	1.33(0.74,1.92)	-0.44(-0.71,-0.16)	5
High SDI	933.66(749.54-1169.89)	1	897.36(711.33-1111.67)	1	-1.87(-2.04,-1.70)	0.95(0.67,1.24)	0.38(0.21,0.55)	-0.10(-0.23,0.04)	1
Low-middle SDI	475.36(395.35-574.04)	5	415.97(340.39-514.53)	5	-1.61(-1.73,-1.48)	0.15(-0.19,0.49)	0.35(-0.41,1.11)	-0.35(-0.62,-0.08)	4
Low SDI	594.50(491.39-718.57)	2	544.09(441.51-666.93)	2	-0.90(-1.02,-0.78)	-0.03(-0.16,0.10)	0.22(-0.21,0.65)	-0.26(-0.40,-0.11)	2
Middle SDI	523.33(417.01-662.57)	4	483.85(375.43-623.84)	3	-1.19(-1.28,-1.10)	-0.57(-0.68,-0.46)	0.95(0.05,1.87)	-0.29(-0.58,0.00)	3
Inflammatory bowel disease									
High-middle SDI	6.12(5.36-6.99)	2	5.58(4.98-6.29)	2	-0.82(-1.02,-0.61)	-0.21(-0.27,-0.15)	0.10(0.05,0.15)	-0.33(-0.41,-0.25)	5
High SDI	18.96(16.73-21.58)	1	17.61(16.03-19.41)	1	-1.50(-1.61,-1.38)	0.40(0.37,0.43)	0.20(0.16,0.23)	-0.25(-0.29,-0.22)	4
Low-middle SDI	2.00(1.70-2.40)	3	2.22(1.89-2.66)	4	0.96(0.42,1.50)	0.41(0.31,0.52)	-0.35(-0.59,-0.11)	0.32(0.13,0.51)	2
Low SDI	1.46(1.23-1.77)	5	1.59(1.35-1.91)	5	0.34(0.30,0.38)	0.43(0.37,0.48)	0.14(-0.02,0.30)	0.30(0.23,0.37)	3
Middle SDI	1.83(1.58-2.15)	4	2.42(2.06-2.87)	3	1.34(1.14,1.53)	0.51(0.33,0.69)	0.88(0.82,0.93)	0.96(0.87,1.05)	1
Multiple sclerosis									
High-middle SDI	0.88(0.77-0.99)	2	0.81(0.71-0.90)	2	-0.63(-0.71,-0.55)	-0.07(-0.09,-0.05)	-0.19(-0.25,-0.13)	-0.31(-0.34,-0.27)	5
High SDI	2.29(2.02-2.56)	1	2.53(2.29-2.76)	1	0.17(0.13,0.21)	0.30(0.28,0.32)	0.54(0.52,0.56)	0.35(0.33,0.37)	3
Low-middle SDI	0.37(0.31-0.44)	3	0.42(0.34-0.49)	4	0.16(0.14,0.17)	0.45(0.44,0.47)	0.58(0.54,0.62)	0.41(0.39,0.42)	2
Low SDI	0.37(0.30-0.44)	5	0.40(0.33-0.48)	5	0.13(0.05,0.20)	0.33(0.25,0.40)	0.62(0.60,0.65)	0.35(0.31,0.39)	4
Middle SDI	0.37(0.31-0.43)	4	0.44(0.37-0.51)	3	0.26(0.21,0.30)	0.87(0.85,0.88)	0.67(0.63,0.71)	0.62(0.60,0.64)	1
Rheumatoid arthritis									
High-middle SDI	10.17(9.26-11.23)	5	11.24(10.24-12.38)	5	0.40(0.37,0.42)	0.60(0.59,0.61)	-0.02(-0.09,0.05)	0.34(0.32,0.37)	1
High SDI	15.95(14.73-17.25)	1	17.13(15.77-18.61)	1	0.34(0.20,0.47)	0.54(0.47,0.62)	-0.28(-0.42,-0.15)	0.22(0.15,0.30)	5
Low-middle SDI	13.89(12.63-15.29)	2	15.31(13.90-16.84)	2	0.28(0.27,0.29)	0.38(0.37,0.39)	0.30(0.22,0.39)	0.33(0.30,0.36)	3
Low SDI	12.01(10.85-13.21)	3	13.27(12.06-14.56)	3	0.12(-0.01,0.24)	0.50(0.49,0.51)	0.37(0.33,0.40)	0.34(0.30,0.38)	2
Middle SDI	10.65(9.63-11.76)	4	11.48(10.41-12.69)	4	0.38(0.37,0.39)	0.38(0.37,0.39)	0.01(-0.16,0.17)	0.26(0.21,0.31)	4
Psoriasis									
High-middle SDI	84.21(81.14-87.19)	2	69.48(67.10-71.89)	2	-0.74(-0.76,-0.73)	-0.57(-0.60,-0.54)	-0.68(-0.69,-0.67)	-0.66(-0.67,-0.64)	4
High SDI	125.33(121.17-129.37)	1	112.58(108.89-116.07)	1	-0.31(-0.34,-0.27)	-0.57(-0.59,-0.54)	-0.26(-0.29,-0.23)	-0.38(-0.40,-0.36)	1
Low-middle SDI	52.75(50.95-54.63)	4	45.45(43.80-47.02)	3	-0.33(-0.34,-0.32)	-0.51(-0.54,-0.48)	-0.72(-0.74,-0.71)	-0.52(-0.53,-0.51)	3
Low SDI	45.21(43.57-46.85)	5	39.82(38.40-41.29)	5	-0.19(-0.23,-0.16)	-0.43(-0.44,-0.42)	-0.67(-0.73,-0.61)	-0.44(-0.46,-0.42)	2
Middle SDI	53.26(51.39-55.18)	3	43.45(41.94-44.94)	4	-0.63(-0.64,-0.62)	-0.68(-0.71,-0.66)	-0.79(-0.79,-0.78)	-0.70(-0.71,-0.69)	5
Atopic dermatitis									
High-middle SDI	352.30(335.52-369.96)	3	370.26(352.74-388.58)	2	0.19(0.17,0.21)	0.30(0.29,0.32)	-0.01(-0.02,0.01)	0.17(0.16,0.18)	1
High SDI	409.13(385.75-432.82)	1	394.45(371.60-417.31)	1	0.14(0.06,0.22)	-0.52(-0.57,-0.48)	-0.05(-0.09,0.00)	-0.14(-0.18,-0.10)	4

Low-middle SDI	299.54(283.79-316.48)	4	287.48(272.29-303.44)	4	-0.03(-0.04,-0.02)	-0.13(-0.13,-0.12)	-0.27(-0.29,-0.25)	-0.14(-0.15,-0.14)	5
Low SDI	205.73(194.46-218.17)	5	199.38(188.75-211.08)	5	-0.06(-0.09,-0.04)	-0.08(-0.08,-0.07)	-0.18(-0.19,-0.17)	-0.11(-0.12,-0.10)	2
Middle SDI	375.57(359.57-390.98)	2	361.82(345.76-377.46)	3	-0.11(-0.12,-0.11)	-0.11(-0.11,-0.11)	-0.17(-0.18,-0.16)	-0.13(-0.13,-0.13)	3
**Number of incident cases**									
Asthma									
High-middle SDI	6,242,717(5,020,769-7,876,345)	3	5,410,537(4,349,433-6,726,613)	5	-1.08(-1.51,-0.66)	-1.46(-1.66,-1.26)	1.52(1.07,1.98)	-0.40(-0.63,-0.17)	5
High SDI	6,920,037(5,729,678-8,410,662)	2	7,056,076(5,900,059-8,406,846)	3	-1.88(-2.04,-1.72)	1.10(0.86,1.34)	0.80(0.65,0.95)	0.09(-0.03,0.20)	4
Low-middle SDI	5,682,545(4,523,305-7,225,234)	4	7,096,394(5,718,776-8,795,881)	2	-0.28(-0.42,-0.14)	1.57(1.22,1.92)	1.27(0.55,2.00)	0.89(0.62,1.15)	2
Low SDI	3,763,030(2,966,775-4,825,345)	5	7,016,216(5,410,570-9,113,992)	4	1.77(1.63,1.90)	2.77(2.58,2.96)	2.17(1.71,2.63)	2.24(2.08,2.40)	1
Middle SDI	9,529,902(7,357,881-12,466,457)	1	10,369,656(8,174,206-13,107,057)	1	-0.41(-0.83,0.02)	-0.17(-0.38,0.03)	1.84(1.42,2.25)	0.38(0.16,0.60)	3
Inflammatory bowel disease									
High-middle SDI	71,474(62,545-81,510)	2	94,592(84,133-107,200)	2	0.79(0.65,0.92)	1.17(1.06,1.28)	0.90(0.81,0.99)	0.95(0.89,1.02)	4
High SDI	171,841(151,878-195,746)	1	196,451(179,556-217,510)	1	-0.74(-0.88,-0.61)	1.38(1.33,1.42)	0.89(0.83,0.95)	0.47(0.42,0.52)	5
Low-middle SDI	17,434(14,532-20,935)	3	36,903(31,053-44,312)	4	3.31(2.50,4.14)	2.69(2.58,2.81)	1.66(1.38,1.94)	2.56(2.29,2.84)	3
Low SDI	5,279(4,395-6,379)	4	12,776(10,613-15,507)	5	2.87(2.73,3.02)	3.28(3.20,3.36)	3.00(2.84,3.15)	3.07(2.99,3.15)	1
Middle SDI	27,459(23,259-32,310)	5	63,681(54,080-75,590)	3	3.99(3.55,4.44)	2.57(2.50,2.63)	2.33(2.17,2.50)	2.94(2.79,3.09)	2
Multiple sclerosis									
High-middle SDI	10,581(9,261-11,867)	2	12,330(10,846-13,773)	2	0.71(0.65,0.77)	0.84(0.76,0.93)	-0.08(-0.18,0.02)	0.50(0.45,0.55)	5
High SDI	20,101(17,824-22,508)	1	24,240(21,900-26,507)	1	0.52(0.37,0.68)	0.69(0.62,0.75)	0.80(0.77,0.82)	0.67(0.62,0.73)	4
Low-middle SDI	3,647(2,952-4,399)	4	7,448(6,088-8,850)	4	2.48(2.47,2.48)	2.54(2.51,2.58)	2.44(2.41,2.47)	2.49(2.47,2.50)	2
Low SDI	1,542(1,237-1,874)	5	3,951(3,192-4,745)	5	3.03(2.93,3.12)	3.31(3.24,3.38)	3.60(3.57,3.63)	3.30(3.26,3.34)	1
Middle SDI	5,965(4,920-7,066)	3	11,344(9,469-13,171)	3	2.43(2.33,2.54)	2.54(2.49,2.59)	1.72(1.65,1.79)	2.26(2.21,2.31)	3
Rheumatoid arthritis									
High-middle SDI	118,141(107,393-130,451)	3	199,084(179,889-219,047)	4	2.05(1.87,2.22)	2.14(2.07,2.22)	1.09(0.94,1.25)	1.79(1.70,1.87)	4
High SDI	149,344(138,368-161,540)	2	232,587(214,481-252,315)	3	1.66(1.52,1.79)	1.95(1.88,2.02)	0.87(0.71,1.02)	1.52(1.44,1.60)	5
Low-middle SDI	109,206(99,111-119,978)	4	242,444(219,999-266,778)	2	2.67(2.65,2.69)	2.86(2.84,2.89)	2.75(2.69,2.81)	2.77(2.74,2.79)	2
Low SDI	38,403(34,587-42,429)	5	94,503(85,249-104,582)	5	2.54(2.47,2.61)	3.33(3.29,3.38)	3.56(3.53,3.58)	3.15(3.12,3.17)	1
Middle SDI	152,154(137,557-168,806)	1	305,363(275,479-337,878)	1	2.77(2.76,2.78)	2.64(2.63,2.65)	1.83(1.74,1.92)	2.43(2.40,2.46)	3
Psoriasis									
High-middle SDI	976,580(940,735-1,011,088)	2	1,107,305(1,066,590-1,146,738)	2	0.50(0.49,0.52)	0.56(0.54,0.58)	0.22(0.20,0.24)	0.44(0.43,0.45)	4
High SDI	1,074,706(1,038,316-1,110,451)	1	1,222,661(1,181,243-1,262,575)	1	0.50(0.47,0.53)	0.40(0.38,0.42)	0.41(0.37,0.45)	0.43(0.42,0.45)	5
Low-middle SDI	542,259(524,003-562,547)	4	785,657(758,178-813,890)	4	1.62(1.59,1.64)	1.30(1.28,1.32)	0.92(0.90,0.95)	1.28(1.27,1.30)	2
Low SDI	210,360(202,895-218,246)	5	400,643(386,621-416,424)	5	2.42(2.26,2.58)	2.35(2.31,2.39)	1.96(1.93,1.99)	2.24(2.18,2.29)	1
Middle SDI	847,467(818,351-878,420)	3	1,103,743(1,063,920-1,142,626)	3	1.25(1.24,1.26)	0.92(0.88,0.97)	0.55(0.52,0.58)	0.91(0.89,0.93)	3
Atopic dermatitis									
High-middle SDI	3,970,974(3,778,337-4,166,089)	3	4,752,301(4,543,971-4,954,992)	3	0.30(0.27,0.33)	0.84(0.82,0.86)	0.69(0.66,0.72)	0.62(0.60,0.64)	4
High SDI	2,972,029(2,818,698-3,125,298)	4	3,356,190(3,198,388-3,509,968)	4	0.54(0.50,0.59)	0.13(0.10,0.16)	0.51(0.49,0.52)	0.41(0.39,0.43)	5
Low-middle SDI	4,075,278(3,843,072-4,337,720)	2	5,138,236(4,862,771-5,429,596)	2	1.24(1.20,1.28)	0.83(0.81,0.84)	0.34(0.32,0.35)	0.80(0.79,0.82)	2

Low SDI	1,503,649(1,413,742-1,605,800)	5	2,874,078(2,707,609-3,064,170)	5	2.53(2.52,2.54)	2.50(2.47,2.53)	1.73(1.65,1.82)	2.26(2.23,2.30)	1
Middle SDI	6,654,491(6,353,883-6,958,746)	1	8,310,047(7,938,439-8,656,340)	1	0.87(0.85,0.89)	0.75(0.70,0.80)	0.69(0.68,0.70)	0.77(0.75,0.78)	3

sTable 5. The incident cases and age-standardized rate of incidence attributable to six immune mediated inflammatory diseases according to GBD regions and its temporal trends from 1990 to 2019.

	1990		2019		1990-1999	2000-2009	2010-2019	1990-2019	
	N(95%CI)	Rank	N(95%CI)	Rank	AAPC(95%CI)	AAPC(95%CI)	AAPC(95%CI)	AAPC(95%CI)	Rank
**Age standardized rate**									
Asthma									
East Asia	400.46(301.23-534.05)	20	361.24(264.84-499.83)	20	-2.55(-2.89,-2.21)	-1.76(-2.20,-1.32)	2.81(-1.79,7.64)	-0.54(-2.00,0.93)	13
Southeast Asia	576.89(474.49-709.35)	17	553.39(445.75-698.10)	13	-0.54(-0.60,-0.48)	-0.02(-0.16,0.12)	0.13(0.00,0.27)	-0.17(-0.23,-0.10)	5
Oceania	781.11(662.50-910.50)	7	665.36(567.70-777.32)	6	-0.22(-0.26,-0.17)	-1.04(-1.13,-0.96)	-0.29(-0.49,-0.09)	-0.54(-0.61,-0.47)	14
Central Asia	406.44(328.07-503.31)	19	379.29(293.17-489.20)	19	-0.34(-0.41,-0.28)	-0.89(-1.03,-0.74)	0.60(0.48,0.72)	-0.22(-0.28,-0.15)	6
Central Europe	714.15(592.85-873.90)	11	616.99(483.35-789.96)	8	-0.45(-0.58,-0.33)	-0.85(-0.91,-0.78)	-0.12(-0.30,0.05)	-0.50(-0.58,-0.43)	12
Eastern Europe	655.11(519.74-826.37)	12	461.89(338.65-613.63)	18	-1.14(-1.32,-0.95)	-2.46(-2.64,-2.28)	0.31(-0.07,0.68)	-1.17(-1.33,-1.01)	20
High-income Asia Pacific	852.87(707.47-1042.26)	5	554.49(421.06-724.34)	12	-2.16(-2.37,-1.95)	-2.95(-3.13,-2.77)	0.82(0.64,1.01)	-1.56(-1.68,-1.45)	21
Australasia	784.22(622.41-957.42)	6	615.20(473.25-790.74)	9	0.35(-0.06,0.77)	-3.07(-3.34,-2.81)	0.01(-0.22,0.24)	-0.87(-1.05,-0.68)	19
Western Europe	627.59(526.75-750.60)	13	512.21(397.33-640.62)	16	-1.51(-2.13,-0.89)	-0.49(-0.56,-0.42)	-0.18(-0.69,0.33)	-0.71(-0.97,-0.46)	17
Southern Latin America	729.20(612.38-887.35)	9	742.26(592.41-935.55)	5	0.23(0.02,0.43)	0.02(0.00,0.04)	0.02(0.00,0.04)	0.07(0.00,0.13)	2
High-income North America	1351.69(1038.96-1769.35)	1	1474.11(1188.89-1810.73)	1	-2.63(-3.02,-2.24)	2.55(2.17,2.93)	0.73(0.50,0.97)	0.37(0.16,0.58)	1
Caribbean	1008.24(804.93-1253.61)	3	938.42(741.65-1178.96)	2	-0.39(-0.41,-0.37)	-0.39(-0.41,-0.37)	0.17(0.02,0.31)	-0.22(-0.26,-0.17)	7
Andean Latin America	928.50(698.16-1197.35)	4	760.27(565.28-1022.67)	4	-0.90(-0.97,-0.83)	-1.56(-1.60,-1.51)	0.53(0.44,0.62)	-0.70(-0.74,-0.66)	16
Central Latin America	717.59(561.67-909.60)	10	583.29(427.80-775.69)	11	-1.61(-1.94,-1.27)	-0.98(-1.10,-0.86)	0.38(0.32,0.44)	-0.75(-0.86,-0.63)	18
Tropical Latin America	1045.37(769.77-1384.46)	2	916.11(645.54-1218.08)	3	-0.65(-0.91,-0.39)	-1.36(-1.48,-1.25)	0.83(0.53,1.13)	-0.47(-0.61,-0.33)	11
North Africa and Middle East	613.97(502.26-748.23)	14	589.78(473.10-728.85)	10	-0.32(-0.36,-0.28)	-0.53(-0.56,-0.51)	0.55(0.43,0.68)	-0.13(-0.17,-0.09)	4
South Asia	388.17(325.47-459.44)	21	329.58(274.87-394.58)	21	-3.32(-4.15,-2.49)	1.84(0.53,3.16)	-0.42(-1.00,0.16)	-0.66(-1.18,-0.13)	15
Central Sub-Saharan Africa	585.13(480.34-707.87)	15	518.71(420.69-641.53)	14	-0.46(-0.47,-0.45)	-0.66(-0.74,-0.58)	-0.08(-0.13,-0.03)	-0.41(-0.44,-0.38)	8
Eastern Sub-Saharan Africa	744.14(605.15-919.93)	8	651.85(518.29-817.23)	7	-0.73(-0.82,-0.64)	-0.64(-0.66,-0.61)	0.07(-0.01,0.15)	-0.45(-0.49,-0.41)	10
Southern Sub-Saharan Africa	563.12(429.98-713.65)	18	496.95(376.47-641.51)	17	0.55(-0.05,1.15)	-5.19(-6.98,-3.38)	5.06(4.22,5.90)	-0.03(-0.73,0.67)	3
Western Sub-Saharan Africa	581.54(469.32-726.04)	16	514.29(408.95-658.46)	15	-0.83(-1.26,-0.40)	-0.52(-0.60,-0.44)	0.04(-0.23,0.31)	-0.44(-0.61,-0.28)	9
Inflammatory bowel disease									
East Asia	1.45(1.22-1.71)	15	2.95(2.55-3.43)	10	3.23(2.93,3.53)	1.80(1.48,2.12)	2.14(2.08,2.21)	2.48(2.33,2.63)	1
Southeast Asia	0.46(0.38-0.56)	20	0.70(0.59-0.84)	20	2.55(2.42,2.67)	0.57(0.49,0.66)	1.02(0.98,1.06)	1.46(1.41,1.51)	3
Oceania	0.44(0.36-0.52)	21	0.56(0.47-0.67)	21	0.39(0.32,0.47)	0.91(0.80,1.01)	1.14(1.06,1.22)	0.83(0.77,0.88)	5
Central Asia	5.99(5.13-7.07)	8	6.90(5.87-8.13)	7	0.35(0.20,0.49)	0.37(0.05,0.69)	0.72(0.60,0.83)	0.50(0.37,0.62)	11
Central Europe	15.78(13.61-18.19)	2	11.59(10.51-12.81)	5	-2.51(-2.67,-2.35)	-0.56(-0.71,-0.41)	0.31(0.28,0.34)	-1.11(-1.19,-1.04)	20
Eastern Europe	7.10(6.12-8.29)	6	7.34(6.34-8.55)	6	0.70(0.62,0.79)	-0.04(-0.19,0.11)	-0.17(-0.36,0.02)	0.16(0.07,0.25)	17
High-income Asia Pacific	9.25(7.88-10.91)	5	14.93(13.14-16.93)	4	4.20(3.56,4.84)	0.47(0.32,0.63)	0.68(0.58,0.77)	1.68(1.47,1.89)	2
Australasia	13.56(11.64-15.80)	4	20.03(17.79-22.57)	2	3.35(3.14,3.57)	0.47(0.44,0.49)	0.39(0.38,0.41)	1.35(1.28,1.42)	4
Western Europe	13.98(12.67-15.48)	3	16.94(15.33-18.73)	3	1.04(0.95,1.13)	0.58(0.55,0.62)	0.41(0.33,0.49)	0.67(0.62,0.71)	9
Southern Latin America	1.82(1.54-2.13)	13	1.92(1.63-2.26)	14	0.95(0.88,1.03)	0.20(0.18,0.23)	-0.57(-0.68,-0.45)	0.19(0.15,0.24)	16

High-income North America	34.88(30.59-40.02)	1	24.51(22.65-26.77)	1	-4.49(-4.63,-4.35)	0.60(0.31,0.89)	-0.03(-0.11,0.04)	-1.24(-1.37,-1.11)	21
Caribbean	2.11(1.83-2.49)	12	2.63(2.24-3.14)	12	1.43(1.34,1.52)	-0.79(-0.84,-0.74)	1.69(1.64,1.74)	0.76(0.72,0.80)	7
Andean Latin America	1.63(1.41-1.91)	14	1.82(1.57-2.12)	15	1.66(1.51,1.82)	-2.09(-2.16,-2.01)	1.61(1.53,1.69)	0.37(0.31,0.44)	13
Central Latin America	3.16(2.74-3.66)	9	2.64(2.29-3.06)	11	-1.16(-1.34,-0.97)	-1.63(-1.74,-1.52)	0.83(0.75,0.91)	-0.60(-0.67,-0.52)	19
Tropical Latin America	6.09(5.40-6.93)	7	5.25(4.60-6.03)	8	-0.41(-0.56,-0.26)	-0.73(-1.44,-0.01)	-0.41(-0.50,-0.31)	-0.51(-0.74,-0.27)	18
North Africa and Middle East	2.95(2.59-3.38)	10	3.74(3.19-4.45)	9	1.46(1.42,1.49)	-0.14(-0.22,-0.06)	1.12(1.07,1.17)	0.83(0.79,0.86)	6
South Asia	2.15(1.79-2.64)	11	2.32(1.93-2.84)	13	0.79(0.76,0.83)	0.79(0.76,0.83)	-0.82(-1.41,-0.23)	0.29(0.10,0.47)	14
Central Sub-Saharan Africa	1.04(0.88-1.26)	17	1.26(1.06-1.52)	17	0.68(0.65,0.72)	0.32(0.23,0.41)	1.16(1.00,1.32)	0.70(0.63,0.76)	8
Eastern Sub-Saharan Africa	0.89(0.76-1.07)	19	1.04(0.88-1.25)	19	0.19(0.18,0.20)	0.67(0.63,0.70)	0.71(0.65,0.78)	0.54(0.51,0.56)	10
Southern Sub-Saharan Africa	1.30(1.10-1.56)	16	1.37(1.17-1.65)	16	0.39(0.28,0.49)	-0.31(-0.49,-0.12)	0.57(0.50,0.64)	0.24(0.16,0.31)	15
Western Sub-Saharan Africa	1.01(0.86-1.23)	18	1.17(1.00-1.41)	18	0.26(0.21,0.32)	-0.22(-0.24,-0.20)	1.56(1.46,1.65)	0.48(0.45,0.52)	12
Multiple sclerosis									
East Asia	0.18(0.15-0.22)	13	0.18(0.14-0.21)	20	-0.50(-0.54,-0.47)	-0.40(-0.44,-0.36)	0.49(0.39,0.59)	-0.16(-0.20,-0.13)	17
Southeast Asia	0.18(0.15-0.22)	11	0.18(0.15-0.21)	19	-0.13(-0.15,-0.12)	-0.06(-0.08,-0.05)	0.01(0.00,0.01)	-0.06(-0.07,-0.05)	16
Oceania	0.14(0.12-0.17)	17	0.14(0.11-0.17)	21	-0.26(-0.30,-0.21)	-0.13(-0.18,-0.08)	-0.10(-0.12,-0.08)	-0.16(-0.18,-0.14)	18
Central Asia	1.53(1.36-1.72)	18	1.39(1.21-1.58)	6	-0.20(-0.21,-0.18)	-0.33(-0.34,-0.32)	-0.49(-0.55,-0.43)	-0.34(-0.36,-0.32)	20
Central Europe	1.78(1.58-1.98)	12	1.70(1.52-1.88)	4	-0.05(-0.12,0.03)	0.06(-0.04,0.16)	-0.62(-0.69,-0.54)	-0.18(-0.24,-0.13)	19
Eastern Europe	1.26(1.07-1.44)	6	1.05(0.89-1.22)	7	-0.72(-0.79,-0.66)	-0.22(-0.26,-0.18)	-0.95(-1.16,-0.73)	-0.60(-0.68,-0.53)	21
High-income Asia Pacific	0.35(0.28-0.42)	9	0.36(0.29-0.44)	15	0.22(0.10,0.34)	-0.05(-0.07,-0.03)	0.39(0.32,0.45)	0.17(0.12,0.21)	9
Australasia	1.54(1.38-1.71)	15	2.09(1.82-2.35)	3	3.06(2.84,3.29)	0.38(0.32,0.44)	-0.58(-0.78,-0.38)	1.05(0.95,1.16)	1
Western Europe	2.59(2.30-2.90)	16	3.18(2.81-3.55)	2	0.84(0.78,0.89)	0.61(0.55,0.67)	0.64(0.53,0.75)	0.69(0.65,0.74)	3
Southern Latin America	0.91(0.76-1.06)	10	0.94(0.79-1.10)	8	0.17(0.16,0.18)	0.10(0.09,0.12)	0.06(0.04,0.07)	0.11(0.11,0.12)	10
High-income North America	3.48(3.07-3.90)	1	3.57(3.29-3.84)	1	-0.36(-0.62,-0.09)	0.29(0.28,0.30)	0.29(0.28,0.30)	0.09(0.01,0.17)	12
Caribbean	0.43(0.35-0.51)	8	0.47(0.38-0.55)	10	0.52(0.49,0.55)	0.24(0.23,0.25)	0.13(0.10,0.15)	0.29(0.28,0.31)	7
Andean Latin America	0.27(0.22-0.32)	2	0.32(0.26-0.38)	16	1.05(1.01,1.09)	0.65(0.59,0.72)	0.28(0.26,0.30)	0.66(0.63,0.68)	4
Central Latin America	0.32(0.26-0.38)	4	0.40(0.33-0.47)	13	1.44(1.40,1.49)	0.72(0.69,0.75)	0.12(0.10,0.15)	0.76(0.74,0.78)	2
Tropical Latin America	0.75(0.63-0.88)	14	0.79(0.66-0.93)	9	0.04(-0.04,0.13)	0.04(-0.04,0.13)	0.04(-0.48,0.57)	0.10(-0.09,0.29)	11
North Africa and Middle East	1.32(1.14-1.49)	7	1.39(1.20-1.58)	5	-0.16(-0.22,-0.10)	0.22(0.19,0.25)	0.47(0.44,0.50)	0.20(0.17,0.22)	8
South Asia	0.37(0.30-0.44)	3	0.40(0.33-0.48)	12	0.05(-0.02,0.11)	0.35(0.32,0.38)	0.53(0.50,0.55)	0.31(0.28,0.34)	6
Central Sub-Saharan Africa	0.22(0.17-0.26)	5	0.22(0.18-0.27)	18	-0.21(-0.25,-0.17)	0.12(0.11,0.13)	0.35(0.34,0.37)	0.09(0.08,0.11)	13
Eastern Sub-Saharan Africa	0.26(0.20-0.31)	21	0.26(0.20-0.31)	17	-0.46(-0.49,-0.43)	0.02(-0.05,0.09)	0.49(0.44,0.54)	0.01(-0.02,0.04)	15
Southern Sub-Saharan Africa	0.37(0.30-0.45)	20	0.38(0.31-0.46)	14	-0.46(-0.48,-0.44)	0.09(0.00,0.18)	0.53(0.46,0.60)	0.06(0.02,0.09)	14
Western Sub-Saharan Africa	0.37(0.30-0.43)	19	0.41(0.34-0.48)	11	0.31(0.29,0.33)	0.41(0.40,0.42)	0.51(0.48,0.55)	0.41(0.39,0.42)	5
Rheumatoid arthritis									
East Asia	11.41(10.31-12.65)	10	11.68(10.56-12.89)	13	0.26(0.25,0.27)	0.38(0.37,0.39)	-0.52(-0.60,-0.43)	0.07(0.05,0.10)	19
Southeast Asia	5.05(4.47-5.68)	18	5.54(4.92-6.24)	19	0.27(0.19,0.35)	0.61(0.57,0.65)	-0.02(-0.19,0.15)	0.31(0.25,0.37)	10
Oceania	3.69(3.20-4.26)	21	3.92(3.40-4.51)	21	0.37(0.32,0.41)	-0.09(-0.16,-0.02)	0.40(0.32,0.48)	0.22(0.18,0.26)	14
Central Asia	13.57(12.47-14.89)	9	17.33(15.91-18.97)	5	0.18(0.09,0.26)	1.14(1.04,1.25)	1.23(1.17,1.29)	0.88(0.83,0.94)	3

Central Europe	10.82(9.73-11.99)	13	11.44(10.20-12.72)	14	0.45(0.13,0.77)	0.45(0.35,0.54)	-0.57(-0.78,-0.35)	0.15(0.01,0.29)	17
Eastern Europe	7.19(6.45-8.00)	17	7.61(6.83-8.47)	17	0.13(0.11,0.15)	0.32(0.30,0.34)	0.13(0.12,0.14)	0.20(0.19,0.21)	15
High-income Asia Pacific	14.50(13.05-15.99)	8	14.19(12.75-15.65)	11	0.16(0.11,0.20)	0.16(0.11,0.20)	-0.45(-1.26,0.36)	-0.03(-0.29,0.22)	20
Australasia	19.12(17.25-21.24)	2	20.89(18.75-23.34)	3	0.81(0.78,0.84)	0.62(0.58,0.65)	-0.56(-0.82,-0.30)	0.31(0.22,0.40)	11
Western Europe	14.94(13.64-16.35)	7	15.76(14.35-17.32)	8	0.42(0.40,0.44)	0.42(0.40,0.44)	-0.32(-0.68,0.05)	0.19(0.08,0.31)	16
Southern Latin America	10.93(9.78-12.24)	12	14.51(13.16-16.09)	10	1.50(1.48,1.53)	1.00(0.98,1.02)	0.37(0.23,0.52)	0.97(0.93,1.02)	2
High-income North America	18.12(16.98-19.40)	3	21.46(20.02-23.09)	1	0.59(0.54,0.63)	0.94(0.89,0.98)	0.18(0.15,0.21)	0.59(0.57,0.62)	7
Caribbean	8.84(7.78-10.03)	16	10.77(9.55-12.17)	15	0.85(0.83,0.86)	0.79(0.74,0.85)	0.41(0.40,0.42)	0.69(0.67,0.70)	5
Andean Latin America	10.01(8.93-11.31)	14	14.74(13.16-16.62)	9	1.67(1.58,1.76)	1.28(1.18,1.39)	1.06(1.04,1.09)	1.36(1.31,1.40)	1
Central Latin America	20.35(18.54-22.23)	1	21.12(19.29-23.07)	2	0.20(0.15,0.25)	-0.07(-0.18,0.04)	0.16(-0.07,0.39)	0.09(0.00,0.17)	18
Tropical Latin America	11.25(10.07-12.59)	11	12.00(10.74-13.41)	12	0.11(0.03,0.19)	0.15(0.11,0.19)	0.46(0.41,0.51)	0.24(0.20,0.28)	13
North Africa and Middle East	4.68(4.15-5.30)	19	5.86(5.18-6.63)	18	0.55(0.51,0.60)	0.92(0.89,0.94)	0.87(0.82,0.92)	0.79(0.76,0.82)	4
South Asia	16.66(15.08-18.36)	5	18.09(16.35-19.94)	4	0.25(0.25,0.26)	0.25(0.25,0.26)	0.32(0.26,0.38)	0.28(0.26,0.30)	12
Central Sub-Saharan Africa	9.18(8.12-10.32)	15	10.57(9.39-11.92)	16	0.02(-0.06,0.10)	0.75(0.72,0.78)	0.64(0.34,0.94)	0.49(0.39,0.59)	8
Eastern Sub-Saharan Africa	15.05(13.60-16.61)	6	17.02(15.40-18.77)	7	0.16(0.07,0.25)	0.71(0.67,0.76)	0.38(0.32,0.43)	0.43(0.39,0.47)	9
Southern Sub-Saharan Africa	17.51(15.69-19.48)	4	17.16(15.35-19.11)	6	-0.21(-0.29,-0.14)	0.23(0.17,0.29)	-0.27(-0.41,-0.13)	-0.08(-0.13,-0.02)	21
Western Sub-Saharan Africa	3.90(3.45-4.40)	20	4.63(4.09-5.23)	20	0.46(0.34,0.59)	0.53(0.40,0.65)	0.92(0.75,1.10)	0.62(0.53,0.71)	6
Psoriasis									
East Asia	68.35(65.91-70.81)	10	54.11(52.23-55.94)	12	-0.79(-0.81,-0.76)	-0.77(-0.83,-0.71)	-0.81(-0.84,-0.78)	-0.80(-0.82,-0.77)	19
Southeast Asia	23.05(22.17-23.88)	21	20.07(19.29-20.78)	21	-0.56(-0.59,-0.52)	-0.42(-0.43,-0.41)	-0.46(-0.56,-0.36)	-0.48(-0.51,-0.44)	14
Oceania	43.09(41.40-44.85)	15	39.17(37.63-40.77)	16	-0.38(-0.40,-0.35)	-0.21(-0.22,-0.20)	-0.42(-0.47,-0.37)	-0.33(-0.34,-0.31)	8
Central Asia	74.11(71.17-77.12)	7	62.24(59.72-64.69)	7	-0.47(-0.51,-0.43)	-0.78(-0.82,-0.75)	-0.48(-0.50,-0.45)	-0.59(-0.61,-0.57)	17
Central Europe	71.31(68.82-73.84)	9	60.27(58.48-62.17)	8	-0.61(-0.65,-0.57)	-0.71(-0.79,-0.64)	-0.32(-0.36,-0.29)	-0.56(-0.59,-0.53)	16
Eastern Europe	67.11(64.77-69.57)	11	59.54(57.57-61.52)	9	-0.53(-0.55,-0.51)	-0.39(-0.44,-0.34)	-0.23(-0.25,-0.21)	-0.41(-0.43,-0.39)	10
High-income Asia Pacific	41.36(39.94-42.86)	16	39.97(38.55-41.42)	15	-0.21(-0.22,-0.20)	-0.09(-0.10,-0.09)	-0.07(-0.08,-0.05)	-0.11(-0.12,-0.11)	1
Australasia	157.82(151.86-163.78)	2	145.38(139.62-151.43)	2	-0.03(-0.07,0.01)	-0.20(-0.22,-0.18)	-0.63(-0.76,-0.49)	-0.28(-0.33,-0.23)	6
Western Europe	216.88(209.34-224.16)	1	204.51(197.64-211.36)	1	-0.18(-0.18,-0.18)	-0.18(-0.18,-0.18)	-0.25(-0.28,-0.22)	-0.20(-0.21,-0.19)	3
Southern Latin America	110.10(105.50-114.69)	3	102.07(97.89-106.31)	3	-0.34(-0.37,-0.31)	-0.17(-0.19,-0.16)	-0.28(-0.30,-0.26)	-0.27(-0.28,-0.25)	5
High-income North America	105.04(101.70-108.14)	4	92.70(89.72-95.53)	4	-0.06(-0.10,-0.01)	-1.00(-1.09,-0.91)	-0.18(-0.22,-0.13)	-0.45(-0.49,-0.41)	12
Caribbean	61.27(58.86-63.74)	13	56.92(54.72-59.25)	10	-0.16(-0.19,-0.13)	-0.38(-0.39,-0.36)	-0.23(-0.23,-0.22)	-0.25(-0.26,-0.24)	4
Andean Latin America	94.80(90.91-98.80)	5	82.37(78.80-85.77)	6	-0.44(-0.50,-0.38)	-0.42(-0.44,-0.40)	-0.62(-0.65,-0.58)	-0.49(-0.52,-0.46)	15
Central Latin America	23.59(22.71-24.45)	20	20.71(19.93-21.49)	20	-0.59(-0.65,-0.52)	-0.34(-0.42,-0.25)	-0.39(-0.41,-0.38)	-0.45(-0.48,-0.41)	13
Tropical Latin America	92.37(89.15-95.39)	6	87.14(84.12-90.05)	5	-0.24(-0.26,-0.23)	-0.14(-0.16,-0.13)	-0.20(-0.22,-0.19)	-0.19(-0.20,-0.18)	2
North Africa and Middle East	72.15(69.36-74.86)	8	55.50(53.46-57.52)	11	-0.88(-0.89,-0.87)	-0.99(-1.01,-0.97)	-0.82(-0.83,-0.82)	-0.90(-0.91,-0.89)	21
South Asia	48.38(46.75-50.10)	14	44.41(42.86-45.97)	14	-0.03(-0.04,-0.02)	-0.35(-0.36,-0.34)	-0.54(-0.56,-0.51)	-0.30(-0.31,-0.29)	7
Central Sub-Saharan Africa	61.81(59.24-64.51)	12	51.61(49.40-53.80)	13	-0.27(-0.34,-0.21)	-0.39(-0.49,-0.30)	-1.26(-1.32,-1.21)	-0.64(-0.68,-0.59)	18
Eastern Sub-Saharan Africa	27.85(26.79-28.93)	19	25.13(24.21-26.09)	19	-0.33(-0.38,-0.27)	-0.23(-0.28,-0.18)	-0.49(-0.51,-0.48)	-0.36(-0.39,-0.34)	9
Southern Sub-Saharan Africa	36.78(35.50-38.17)	18	32.87(31.69-34.06)	17	-0.56(-0.71,-0.41)	-0.20(-0.38,-0.03)	-0.44(-0.47,-0.40)	-0.41(-0.49,-0.34)	11

Western Sub-Saharan Africa	40.67(39.20-42.12)	17	32.46(31.29-33.64)	18	-0.72(-0.79,-0.66)	-0.76(-0.98,-0.55)	-0.89(-1.10,-0.67)	-0.80(-0.90,-0.70)	20
Atopic dermatitis									
East Asia	412.26(395.49-427.83)	6	413.82(396.40-429.81)	6	-0.09(-0.11,-0.07)	0.05(0.04,0.06)	0.07(0.04,0.11)	0.01(0.00,0.03)	4
Southeast Asia	532.36(505.38-558.15)	3	530.26(503.33-555.12)	3	-0.02(-0.02,-0.02)	0.00(-0.01,0.00)	-0.02(-0.02,-0.02)	-0.01(-0.01,-0.01)	13
Oceania	484.15(450.84-516.78)	4	484.95(451.62-517.53)	4	0.02(0.02,0.03)	-0.01(-0.01,-0.01)	0.00(0.00,0.00)	0.01(0.00,0.01)	5
Central Asia	614.63(543.66-695.31)	1	610.57(539.97-691.42)	1	-0.02(-0.03,-0.02)	-0.03(-0.03,-0.03)	-0.02(-0.02,-0.01)	-0.02(-0.02,-0.02)	17
Central Europe	198.01(185.19-211.75)	17	193.63(182.21-206.07)	17	0.06(0.05,0.08)	-0.26(-0.28,-0.25)	0.01(0.00,0.02)	-0.07(-0.08,-0.07)	19
Eastern Europe	260.89(243.49-279.32)	13	266.04(248.59-283.86)	13	0.12(0.05,0.20)	-0.01(-0.11,0.09)	0.05(0.00,0.09)	0.07(0.02,0.11)	1
High-income Asia Pacific	547.85(513.24-583.03)	2	545.03(509.47-579.47)	2	-0.02(-0.04,0.00)	0.08(0.06,0.11)	-0.16(-0.22,-0.11)	-0.03(-0.05,-0.01)	18
Australasia	361.75(334.45-391.81)	8	360.50(333.61-388.98)	8	0.04(0.03,0.05)	0.01(0.01,0.02)	-0.09(-0.10,-0.08)	-0.01(-0.02,-0.01)	14
Western Europe	477.21(446.25-510.45)	5	476.69(445.44-510.57)	5	-0.04(-0.08,0.01)	-0.04(-0.05,-0.04)	0.07(0.02,0.12)	0.00(-0.03,0.02)	7
Southern Latin America	402.46(371.52-436.12)	7	401.80(370.92-435.35)	7	-0.01(-0.01,-0.01)	0.00(0.00,0.00)	-0.01(-0.01,0.00)	-0.01(-0.01,-0.01)	15
High-income North America	312.93(296.63-329.93)	10	295.03(279.20-311.24)	10	0.88(0.72,1.04)	-1.61(-1.69,-1.53)	0.04(-0.04,0.12)	-0.21(-0.28,-0.14)	21
Caribbean	274.15(251.70-296.05)	12	273.97(251.52-295.86)	12	0.00(0.00,0.00)	0.00(0.00,0.00)	0.00(0.00,0.00)	0.00(0.00,0.00)	8
Andean Latin America	258.61(242.89-274.25)	14	259.37(244.62-274.99)	14	-0.13(-0.17,-0.09)	0.02(0.01,0.02)	0.19(0.15,0.22)	0.02(0.01,0.04)	3
Central Latin America	217.24(204.23-231.73)	15	217.08(203.95-231.55)	15	-0.03(-0.03,-0.02)	0.00(0.00,0.00)	0.02(0.02,0.02)	0.00(0.00,0.00)	9
Tropical Latin America	320.61(303.39-339.02)	9	320.05(303.52-337.54)	9	-0.04(-0.06,-0.03)	0.00(0.00,0.01)	0.02(0.02,0.03)	-0.01(-0.01,0.00)	16
North Africa and Middle East	202.35(189.14-216.48)	16	195.14(182.57-208.75)	16	-0.07(-0.07,-0.06)	-0.19(-0.19,-0.18)	-0.12(-0.12,-0.11)	-0.13(-0.13,-0.12)	20
South Asia	277.40(260.24-296.27)	11	277.76(260.54-296.42)	11	0.12(0.11,0.12)	0.00(0.00,0.00)	-0.11(-0.12,-0.10)	0.00(0.00,0.01)	10
Central Sub-Saharan Africa	159.48(146.77-173.94)	20	159.46(146.75-173.97)	20	0.00(0.00,0.00)	0.00(0.00,0.00)	0.00(0.00,0.00)	0.00(0.00,0.00)	11
Eastern Sub-Saharan Africa	155.13(146.67-164.49)	21	157.63(149.10-167.07)	21	0.04(0.02,0.06)	0.06(0.05,0.07)	0.08(0.06,0.10)	0.06(0.05,0.07)	2
Southern Sub-Saharan Africa	160.66(151.27-171.63)	19	160.50(151.12-171.49)	19	0.00(0.00,0.00)	-0.01(-0.01,-0.01)	-0.01(-0.01,-0.01)	0.00(0.00,0.00)	12
Western Sub-Saharan Africa	161.57(153.29-170.37)	18	162.15(153.96-170.97)	18	-0.04(-0.04,-0.03)	0.02(0.02,0.03)	0.05(0.04,0.06)	0.01(0.01,0.02)	6
**Number of incident cases**									
Asthma									
East Asia	4,718,775(3,544,699-6,361,232)	1	3,979,880(3,082,882-5,179,775)	3	-1.66(-3.02,-0.27)	-2.79(-3.60,-1.98)	4.13(2.73,5.55)	-0.28(-1.05,0.48)	16
Southeast Asia	2,894,847(2,306,202-3,676,835)	4	3,450,998(2,810,277-4,271,027)	5	0.40(0.34,0.47)	0.80(0.67,0.93)	0.61(0.47,0.74)	0.58(0.52,0.65)	11
Oceania	53,282(43,278-65,083)	21	91,492(76,089-110,025)	21	2.50(2.42,2.57)	1.24(1.16,1.32)	2.01(1.89,2.13)	1.90(1.84,1.95)	4
Central Asia	299,599(232,086-389,478)	19	353,674(272,735-459,839)	19	-0.04(-0.16,0.07)	-0.31(-0.42,-0.20)	2.25(2.08,2.41)	0.59(0.50,0.67)	10
Central Europe	860,511(723,121-1,034,183)	13	586,970(486,653-704,768)	14	-1.20(-1.32,-1.08)	-1.90(-1.95,-1.84)	-0.79(-0.94,-0.65)	-1.33(-1.40,-1.27)	19
Eastern Europe	1,433,382(1,159,698-1,770,626)	10	755,273(586,070-966,281)	13	-2.67(-2.90,-2.44)	-3.76(-3.90,-3.62)	0.13(-0.21,0.48)	-2.18(-2.33,-2.02)	21
High-income Asia Pacific	1,371,250(1,152,445-1,646,704)	11	795,949(663,981-951,354)	12	-2.01(-2.20,-1.82)	-3.53(-3.73,-3.32)	0.07(-0.09,0.22)	-1.96(-2.07,-1.84)	20
Australasia	137,909(110,796-166,316)	20	139,912(111,577-175,051)	20	0.88(0.54,1.21)	-1.83(-2.05,-1.61)	0.97(0.82,1.12)	0.02(-0.13,0.17)	14
Western Europe	2,251,292(1,922,884-2,628,385)	6	1,785,531(1,472,143-2,130,570)	8	-1.90(-2.61,-1.19)	-0.49(-0.57,-0.41)	-0.10(-0.64,0.44)	-0.81(-1.09,-0.53)	18
Southern Latin America	368,390(307,660-451,220)	17	453,729(370,284-559,750)	16	0.95(0.81,1.10)	0.54(0.46,0.63)	0.77(0.70,0.84)	0.74(0.68,0.79)	9
High-income North America	3,368,771(2,678,501-4,315,052)	3	4,366,448(3,653,279-5,207,636)	2	-2.06(-2.47,-1.64)	3.17(2.83,3.51)	1.35(1.08,1.62)	0.97(0.75,1.20)	8
Caribbean	396,154(314,621-493,778)	16	403,255(321,534-502,347)	17	0.02(-0.01,0.06)	-0.16(-0.20,-0.13)	0.30(0.02,0.58)	0.04(-0.05,0.13)	13
Andean Latin America	472,850(350,865-612,633)	14	484,221(360,725-652,231)	15	-0.14(-0.24,-0.04)	-1.20(-1.29,-1.10)	1.83(1.74,1.93)	0.07(0.01,0.13)	12

Central Latin America	1,435,630(1,089,179-1,851,815)	9	1,372,619(1,015,874-1,823,855)	10	-0.32(-0.58,-0.06)	-0.47(-0.57,-0.37)	0.41(0.38,0.45)	-0.16(-0.25,-0.07)	15
Tropical Latin America	1,841,692(1,338,521-2,484,003)	8	1,686,457(1,214,675-2,210,165)	9	-0.38(-0.61,-0.14)	-1.20(-1.26,-1.15)	0.78(0.51,1.04)	-0.34(-0.45,-0.22)	17
North Africa and Middle East	2,397,788(1,873,586-3,050,027)	5	3,488,139(2,767,724-4,354,302)	4	1.29(1.21,1.38)	0.86(0.82,0.90)	1.85(1.67,2.04)	1.30(1.24,1.37)	5
South Asia	3,901,616(3,185,149-4,919,914)	2	5,437,961(4,525,432-6,587,224)	1	-2.17(-3.19,-1.14)	4.56(3.01,6.14)	0.88(0.23,1.53)	1.05(0.43,1.68)	7
Central Sub-Saharan Africa	415,201(318,263-530,212)	15	818,097(629,087-1,053,677)	11	2.37(2.34,2.39)	2.36(2.33,2.39)	2.41(2.37,2.45)	2.37(2.35,2.39)	2
Eastern Sub-Saharan Africa	1,866,566(1,437,024-2,426,543)	7	3,319,135(2,507,204-4,344,481)	6	1.81(1.64,1.98)	1.97(1.91,2.02)	2.30(2.14,2.46)	2.02(1.93,2.10)	3
Southern Sub-Saharan Africa	323,886(237,510-424,938)	18	385,370(289,150-504,564)	18	2.17(1.45,2.89)	-5.03(-7.09,-2.92)	6.61(5.67,7.57)	1.06(0.24,1.88)	6
Western Sub-Saharan Africa	1,353,823(1,025,497-1,781,921)	12	2,824,158(2,102,924-3,781,716)	7	2.23(1.98,2.48)	2.97(2.67,3.27)	2.48(2.42,2.55)	2.51(2.38,2.64)	1
Inflammatory bowel disease									
East Asia	17,567(14,627-20,802)	7	52,319(44,680-61,455)	3	5.59(5.22,5.97)	3.24(3.13,3.35)	2.74(2.61,2.87)	3.81(3.68,3.94)	2
Southeast Asia	1,882(1,527-2,270)	13	4,980(4,182-5,972)	13	5.26(5.04,5.48)	2.65(2.47,2.83)	2.49(2.46,2.52)	3.42(3.31,3.53)	7
Oceania	22(18-27)	21	63(52-77)	21	3.10(3.07,3.13)	3.79(3.73,3.85)	3.89(3.81,3.98)	3.61(3.57,3.65)	4
Central Asia	3,514(2,995-4,146)	11	6,461(5,487-7,637)	11	1.58(1.43,1.73)	2.19(2.11,2.27)	2.51(2.00,3.03)	2.10(1.93,2.27)	13
Central Europe	20,997(18,082-24,170)	3	14,809(13,443-16,391)	8	-2.39(-2.66,-2.13)	-0.71(-1.09,-0.34)	-0.06(-0.10,-0.02)	-1.25(-1.40,-1.09)	21
Eastern Europe	18,150(15,600-21,209)	5	19,500(16,838-22,671)	7	0.97(0.89,1.05)	0.00(-0.14,0.14)	-0.12(-0.30,0.05)	0.28(0.20,0.36)	19
High-income Asia Pacific	18,105(15,391-21,422)	6	27,084(23,870-30,621)	5	4.04(3.25,4.84)	0.33(0.24,0.42)	0.03(-0.29,0.36)	1.37(1.11,1.64)	17
Australasia	2,979(2,556-3,481)	12	6,446(5,755-7,212)	12	4.69(4.39,4.99)	1.83(1.82,1.84)	1.83(1.82,1.84)	2.71(2.62,2.80)	9
Western Europe	59,081(53,733-65,529)	2	78,645(71,611-86,709)	2	1.36(1.20,1.51)	1.07(1.01,1.12)	0.59(0.47,0.72)	1.00(0.93,1.07)	18
Southern Latin America	879(742-1,030)	16	1,382(1,177-1,620)	16	2.35(2.27,2.44)	1.61(1.58,1.64)	0.76(0.63,0.89)	1.57(1.52,1.62)	16
High-income North America	107,616(94,147-123,884)	1	99,611(91,945-109,000)	1	-3.74(-4.10,-3.38)	1.77(1.68,1.85)	0.89(0.82,0.96)	-0.27(-0.39,-0.15)	20
Caribbean	677(580-796)	17	1,312(1,121-1,571)	17	3.35(3.23,3.48)	0.82(0.77,0.88)	2.83(2.76,2.90)	2.33(2.28,2.38)	12
Andean Latin America	472(402-550)	19	1,128(975-1,314)	19	4.46(4.25,4.67)	0.55(0.45,0.65)	4.25(4.15,4.36)	3.06(2.98,3.15)	8
Central Latin America	3,968(3,402-4,618)	10	6,754(5,834-7,826)	10	1.80(1.52,2.09)	0.92(0.75,1.09)	2.82(2.69,2.94)	1.89(1.78,2.01)	14
Tropical Latin America	7,826(6,859-8,976)	9	12,928(11,334-14,874)	9	2.00(1.86,2.14)	1.78(1.21,2.36)	1.43(1.28,1.59)	1.74(1.55,1.93)	15
North Africa and Middle East	8,107(7,040-9,204)	8	22,722(19,277-26,952)	6	4.62(4.52,4.72)	2.63(2.54,2.72)	3.59(3.56,3.62)	3.64(3.59,3.68)	3
South Asia	18,439(15,169-22,582)	4	39,705(32,943-48,454)	4	3.08(2.92,3.25)	3.35(3.31,3.39)	1.44(0.99,1.88)	2.67(2.52,2.82)	10
Central Sub-Saharan Africa	381(318-469)	20	1,150(955-1,400)	18	3.67(3.63,3.71)	3.52(3.40,3.64)	4.60(4.38,4.82)	3.90(3.82,3.99)	1
Eastern Sub-Saharan Africa	1,092(908-1,315)	15	2,938(2,449-3,538)	15	2.85(2.71,2.98)	3.57(3.40,3.75)	3.90(3.84,3.97)	3.44(3.37,3.51)	6
Southern Sub-Saharan Africa	504(423-608)	18	989(837-1,188)	20	3.04(2.96,3.11)	1.40(1.21,1.58)	2.66(2.57,2.75)	2.38(2.30,2.45)	11
Western Sub-Saharan Africa	1,314(1,102-1,599)	14	3,628(3,028-4,401)	14	3.27(3.20,3.34)	2.87(2.85,2.90)	4.68(4.56,4.80)	3.55(3.51,3.60)	5
Multiple sclerosis									
East Asia	2,100(1,696-2,542)	7	3,120(2,555-3,722)	5	1.40(1.36,1.44)	1.21(1.17,1.25)	1.48(1.30,1.67)	1.36(1.30,1.42)	15
Southeast Asia	725(571-891)	10	1,251(1,010-1,504)	11	2.25(2.23,2.27)	1.88(1.86,1.90)	1.60(1.57,1.62)	1.90(1.89,1.91)	12
Oceania	7(6-9)	21	16(12-19)	21	2.50(2.45,2.54)	2.76(2.71,2.80)	2.57(2.54,2.60)	2.62(2.59,2.64)	7
Central Asia	887(779-999)	9	1,352(1,176-1,537)	10	1.22(1.15,1.30)	2.23(2.13,2.33)	0.79(0.73,0.85)	1.46(1.41,1.51)	14
Central Europe	2,247(1,986-2,482)	6	1,841(1,648-2,036)	8	0.08(-0.04,0.21)	-0.48(-0.55,-0.41)	-1.77(-1.85,-1.70)	-0.71(-0.77,-0.65)	20
Eastern Europe	2,920(2,484-3,330)	5	2,152(1,832-2,468)	6	-0.72(-0.83,-0.61)	-0.42(-0.54,-0.31)	-2.16(-2.30,-2.03)	-1.07(-1.18,-0.97)	21
High-income Asia Pacific	655(528-787)	11	727(597-872)	14	0.70(0.62,0.78)	0.06(0.04,0.08)	0.33(0.27,0.39)	0.37(0.34,0.41)	19

Australasia	336(301-374)	16	593(520-664)	16	3.99(3.41,4.57)	1.40(1.30,1.50)	0.66(0.30,1.01)	1.96(1.75,2.18)	10
Western Europe	10,254(9,116-11,454)	2	12,497(11,106-13,903)	1	1.06(1.00,1.12)	0.69(0.67,0.71)	0.30(0.21,0.39)	0.68(0.64,0.71)	17
Southern Latin America	445(370-519)	14	657(550-760)	15	1.45(1.44,1.47)	1.42(1.41,1.42)	1.16(1.12,1.21)	1.35(1.33,1.36)	16
High-income North America	10,608(9,353-11,936)	1	12,058(11,089-12,990)	2	-0.19(-0.27,-0.10)	0.70(0.64,0.77)	0.76(0.73,0.80)	0.45(0.41,0.49)	18
Caribbean	148(119-176)	18	230(189-271)	19	2.34(2.22,2.45)	1.33(1.29,1.37)	1.00(0.99,1.02)	1.54(1.50,1.58)	13
Andean Latin America	86(68-104)	20	206(165-244)	20	3.77(3.68,3.87)	2.96(2.91,3.01)	2.59(2.57,2.61)	3.09(3.06,3.13)	3
Central Latin America	454(361-548)	13	1,042(856-1,222)	12	4.28(4.20,4.37)	2.80(2.75,2.85)	1.62(1.56,1.68)	2.89(2.85,2.94)	5
Tropical Latin America	1,041(858-1,223)	8	1,968(1,637-2,320)	7	2.73(2.36,3.10)	2.32(2.23,2.40)	1.47(0.95,1.99)	2.18(1.98,2.38)	9
North Africa and Middle East	4,189(3,598-4,785)	3	9,218(7,879-10,525)	3	2.90(2.79,3.01)	3.03(2.99,3.07)	2.28(2.21,2.34)	2.78(2.73,2.82)	6
South Asia	3,560(2,839-4,324)	4	7,447(5,987-8,965)	4	2.46(2.42,2.50)	2.70(2.69,2.71)	2.58(2.48,2.68)	2.59(2.55,2.62)	8
Central Sub-Saharan Africa	93(73-116)	19	243(188-302)	18	2.93(2.89,2.96)	3.44(3.41,3.47)	3.74(3.70,3.79)	3.37(3.35,3.40)	2
Eastern Sub-Saharan Africa	370(289-459)	15	876(681-1,087)	13	2.49(2.39,2.58)	2.96(2.94,2.99)	3.61(3.55,3.67)	3.02(2.98,3.06)	4
Southern Sub-Saharan Africa	174(138-213)	17	302(240-365)	17	2.04(1.99,2.09)	1.67(1.63,1.70)	2.06(1.95,2.16)	1.91(1.87,1.95)	11
Western Sub-Saharan Africa	554(446-669)	12	1,549(1,264-1,846)	9	3.59(3.56,3.62)	3.64(3.63,3.65)	3.61(3.57,3.65)	3.61(3.59,3.63)	1
Rheumatoid arthritis									
East Asia	129,319(116,794-143,092)	1	229,076(204,356-254,751)	2	2.46(2.45,2.47)	2.46(2.45,2.47)	0.94(0.74,1.15)	1.99(1.92,2.05)	16
Southeast Asia	20,115(17,807-22,784)	7	40,269(35,541-45,594)	7	2.66(2.58,2.75)	2.76(2.72,2.79)	1.77(1.66,1.87)	2.40(2.35,2.45)	11
Oceania	218(186-259)	21	499(428-583)	21	3.09(3.04,3.15)	2.67(2.66,2.69)	2.89(2.85,2.92)	2.89(2.87,2.92)	7
Central Asia	7,950(7,203-8,839)	13	16,172(14,695-17,910)	13	1.55(1.49,1.60)	2.80(2.76,2.85)	3.07(3.03,3.11)	2.50(2.47,2.53)	9
Central Europe	14,738(13,204-16,366)	11	16,228(14,513-18,044)	12	0.90(0.57,1.23)	0.78(0.64,0.92)	-0.93(-1.21,-0.65)	0.28(0.13,0.44)	20
Eastern Europe	17,426(15,654-19,402)	8	17,769(15,896-19,888)	11	0.44(0.41,0.47)	-0.01(-0.02,0.01)	-0.27(-0.28,-0.25)	0.07(0.06,0.08)	21
High-income Asia Pacific	29,396(26,395-32,483)	5	37,155(33,402-41,233)	8	1.46(1.31,1.61)	1.23(1.10,1.36)	-0.52(-0.83,-0.22)	0.76(0.63,0.89)	19
Australasia	4,247(3,843-4,703)	17	7,968(7,149-8,851)	19	2.51(2.32,2.70)	2.87(2.76,2.97)	1.01(0.75,1.26)	2.15(2.03,2.28)	12
Western Europe	69,385(63,395-75,650)	3	92,644(84,751-101,325)	4	1.16(1.02,1.31)	1.40(1.31,1.49)	0.29(0.12,0.47)	0.98(0.89,1.07)	18
Southern Latin America	5,214(4,663-5,861)	15	10,667(9,724-11,791)	16	2.98(2.96,3.00)	2.60(2.58,2.62)	1.84(1.71,1.97)	2.49(2.44,2.53)	10
High-income North America	56,815(53,011-61,123)	4	104,527(96,439-113,263)	3	2.06(2.01,2.11)	2.57(2.54,2.59)	1.73(1.67,1.80)	2.14(2.10,2.17)	13
Caribbean	2,922(2,560-3,361)	20	5,323(4,720-6,014)	20	2.62(2.60,2.65)	2.21(2.17,2.26)	1.43(1.42,1.45)	2.09(2.07,2.10)	14
Andean Latin America	3,038(2,651-3,510)	19	9,218(8,173-10,464)	18	4.40(4.25,4.54)	3.83(3.78,3.88)	3.45(3.25,3.66)	3.91(3.82,4.00)	1
Central Latin America	24,316(22,123-26,694)	6	53,797(49,153-58,879)	5	3.39(3.21,3.57)	2.68(2.60,2.76)	2.18(2.00,2.36)	2.75(2.66,2.85)	8
Tropical Latin America	16,894(15,152-18,948)	9	28,860(25,680-32,360)	10	2.42(2.37,2.47)	1.76(1.65,1.87)	1.49(1.46,1.52)	1.89(1.85,1.93)	17
North Africa and Middle East	13,509(11,860-15,455)	12	36,548(32,078-41,603)	9	3.51(3.46,3.55)	3.76(3.69,3.82)	3.23(3.21,3.26)	3.50(3.48,3.53)	4
South Asia	121,147(109,682-133,792)	2	290,966(262,698-320,812)	1	2.89(2.88,2.91)	3.12(3.08,3.15)	3.17(3.12,3.21)	3.07(3.05,3.09)	6
Central Sub-Saharan Africa	3,221(2,856-3,681)	18	9,347(8,255-10,702)	17	2.99(2.74,3.24)	4.15(4.07,4.22)	3.97(3.80,4.14)	3.71(3.60,3.81)	2
Eastern Sub-Saharan Africa	15,629(14,046-17,361)	10	41,085(36,922-45,824)	6	2.62(2.46,2.77)	3.60(3.50,3.69)	3.85(3.78,3.92)	3.36(3.30,3.43)	5
Southern Sub-Saharan Africa	7,289(6,576-8,103)	14	13,132(11,766-14,691)	15	2.53(2.40,2.65)	2.02(1.97,2.06)	1.48(1.31,1.66)	2.01(1.94,2.08)	15
Western Sub-Saharan Africa	4,675(4,135-5,320)	16	13,142(11,577-14,996)	14	3.35(3.20,3.49)	3.43(3.28,3.59)	4.28(4.08,4.48)	3.67(3.56,3.77)	3
Psoriasis									
East Asia	813,617(784,061-843,006)	2	925,967(891,325-958,216)	2	0.87(0.83,0.91)	0.47(0.46,0.48)	0.01(-0.05,0.06)	0.45(0.43,0.47)	17

Southeast Asia	94,621(91,120-98,283)	8	139,036(133,404-144,184)	8	1.54(1.53,1.56)	1.39(1.37,1.41)	1.07(0.93,1.21)	1.34(1.29,1.38)	10
Oceania	2,382(2,286-2,479)	21	4,694(4,512-4,889)	21	2.27(2.24,2.31)	2.57(2.54,2.60)	2.21(2.18,2.24)	2.36(2.34,2.39)	3
Central Asia	48,180(46,156-50,094)	13	58,976(56,469-61,360)	15	0.54(0.51,0.56)	0.41(0.37,0.46)	1.22(1.19,1.25)	0.70(0.68,0.72)	15
Central Europe	92,543(89,254-95,815)	9	76,996(74,396-79,469)	12	-0.52(-0.54,-0.50)	-0.82(-0.89,-0.76)	-0.48(-0.59,-0.36)	-0.62(-0.67,-0.58)	21
Eastern Europe	161,524(155,673-167,380)	6	139,976(134,745-144,910)	7	-0.46(-0.58,-0.34)	-0.84(-0.92,-0.77)	-0.04(-0.10,0.01)	-0.48(-0.53,-0.43)	20
High-income Asia Pacific	76,336(73,621-79,135)	10	83,991(80,923-86,862)	11	0.52(0.51,0.54)	0.33(0.31,0.36)	0.14(0.09,0.20)	0.34(0.32,0.36)	18
Australasia	32,497(31,226-33,743)	17	43,553(41,826-45,339)	18	1.21(1.17,1.25)	1.16(1.14,1.18)	0.68(0.64,0.72)	1.02(1.00,1.05)	12
Western Europe	873,918(842,795-904,003)	1	946,916(913,064-979,530)	1	0.30(0.26,0.33)	0.43(0.39,0.46)	0.05(0.00,0.11)	0.27(0.24,0.29)	19
Southern Latin America	54,019(51,757-56,252)	12	70,211(67,264-73,298)	13	0.82(0.79,0.84)	1.02(1.01,1.02)	0.90(0.89,0.92)	0.91(0.90,0.92)	14
High-income North America	302,143(292,313-311,286)	4	359,271(347,584-370,502)	4	0.82(0.72,0.93)	0.31(0.19,0.44)	0.70(0.67,0.72)	0.61(0.55,0.66)	16
Caribbean	20,607(19,829-21,437)	19	27,467(26,394-28,614)	19	1.43(1.39,1.47)	0.81(0.80,0.82)	0.75(0.74,0.76)	1.00(0.98,1.02)	13
Andean Latin America	33,750(32,270-35,227)	16	52,103(49,796-54,275)	17	1.69(1.65,1.73)	1.39(1.38,1.41)	1.46(1.34,1.58)	1.51(1.47,1.55)	6
Central Latin America	34,843(33,618-36,214)	15	52,370(50,378-54,362)	16	1.77(1.71,1.83)	1.46(1.44,1.48)	1.03(0.97,1.09)	1.42(1.39,1.45)	8
Tropical Latin America	134,553(129,923-139,258)	7	202,572(195,312-209,349)	6	1.62(1.62,1.63)	1.51(1.49,1.53)	1.13(1.08,1.17)	1.42(1.41,1.44)	7
North Africa and Middle East	224,312(215,902-233,060)	5	336,787(324,072-349,591)	5	1.57(1.53,1.61)	1.39(1.36,1.42)	1.27(1.24,1.31)	1.41(1.39,1.43)	9
South Asia	491,106(474,325-509,187)	3	792,848(765,638-821,309)	3	2.06(2.04,2.07)	1.72(1.70,1.74)	1.18(1.14,1.22)	1.66(1.64,1.68)	5
Central Sub-Saharan Africa	30,906(29,579-32,280)	18	61,984(59,320-64,656)	14	2.67(2.63,2.72)	2.77(2.71,2.83)	1.76(1.72,1.81)	2.42(2.38,2.45)	2
Eastern Sub-Saharan Africa	44,727(43,000-46,689)	14	89,695(86,383-93,561)	10	2.42(2.29,2.55)	2.57(2.49,2.65)	2.32(2.29,2.35)	2.42(2.36,2.49)	1
Southern Sub-Saharan Africa	17,597(16,984-18,313)	20	25,357(24,396-26,331)	20	1.70(1.62,1.79)	1.01(0.92,1.09)	1.11(1.06,1.17)	1.25(1.21,1.30)	11
Western Sub-Saharan Africa	69,053(66,537-71,782)	11	131,825(127,072-137,224)	9	2.31(2.25,2.36)	2.36(2.26,2.47)	2.07(1.91,2.23)	2.24(2.17,2.30)	4
Atopic dermatitis									
East Asia	5,008,814(4,791,100-5,211,250)	1	6,080,367(5,807,761-6,330,811)	1	0.63(0.61,0.66)	0.62(0.62,0.63)	0.77(0.76,0.78)	0.67(0.66,0.68)	11
Southeast Asia	2,508,163(2,378,893-2,643,072)	3	3,524,067(3,336,135-3,697,332)	3	1.44(1.42,1.47)	1.23(1.22,1.24)	0.88(0.84,0.91)	1.18(1.16,1.19)	7
Oceania	32,286(29,914-34,610)	21	65,854(61,124-70,491)	21	2.59(2.58,2.61)	2.42(2.40,2.45)	2.46(2.43,2.49)	2.50(2.48,2.51)	3
Central Asia	518,253(452,966-593,851)	10	583,002(513,906-662,029)	11	-0.64(-0.88,-0.40)	0.31(0.25,0.38)	1.53(1.30,1.77)	0.40(0.29,0.52)	17
Central Europe	216,330(203,707-229,301)	14	172,334(164,449-180,290)	16	-1.37(-1.43,-1.32)	-0.65(-0.78,-0.52)	-0.43(-0.50,-0.35)	-0.81(-0.87,-0.75)	21
Eastern Europe	529,587(497,103-563,367)	9	447,762(424,028-471,302)	13	-2.22(-2.40,-2.03)	-0.08(-0.18,0.01)	0.38(0.12,0.64)	-0.60(-0.72,-0.49)	20
High-income Asia Pacific	762,674(719,624-803,368)	7	746,786(710,243-780,211)	9	0.09(0.05,0.12)	-0.21(-0.31,-0.11)	-0.06(-0.13,0.01)	-0.05(-0.09,-0.01)	19
Australasia	65,070(60,552-69,978)	20	87,950(82,270-93,692)	20	0.92(0.85,1.00)	1.14(1.10,1.17)	1.06(1.01,1.10)	1.05(1.02,1.08)	8
Western Europe	1,499,861(1,416,522-1,587,693)	4	1,649,703(1,557,015-1,741,430)	4	0.03(-0.02,0.08)	0.54(0.48,0.61)	0.30(0.25,0.34)	0.31(0.27,0.34)	18
Southern Latin America	204,051(188,088-221,484)	15	238,534(222,335-256,745)	15	0.51(0.37,0.64)	0.39(0.37,0.41)	0.70(0.58,0.83)	0.54(0.48,0.60)	15
High-income North America	816,447(778,446-853,366)	6	968,991(928,999-1,008,590)	6	1.49(1.35,1.64)	-0.44(-0.50,-0.37)	0.61(0.55,0.68)	0.57(0.51,0.64)	13
Caribbean	103,916(95,171-112,845)	18	119,850(110,716-128,900)	19	0.70(0.58,0.81)	0.32(0.23,0.40)	0.50(0.47,0.53)	0.51(0.46,0.55)	16
Andean Latin America	117,267(109,117-125,225)	17	164,628(155,251-174,694)	17	1.09(1.06,1.13)	0.81(0.77,0.85)	1.75(1.63,1.87)	1.20(1.16,1.24)	6
Central Latin America	426,931(398,511-458,889)	12	512,097(482,639-544,328)	12	1.26(1.20,1.31)	0.46(0.43,0.48)	0.17(0.13,0.21)	0.63(0.60,0.65)	12
Tropical Latin America	532,791(502,570-566,391)	8	624,895(595,717-655,131)	10	0.55(0.51,0.59)	0.64(0.62,0.66)	0.45(0.40,0.49)	0.56(0.53,0.58)	14
North Africa and Middle East	848,965(790,033-911,474)	5	1,215,388(1,135,711-1,299,728)	5	1.51(1.45,1.57)	1.27(1.25,1.30)	0.96(0.92,1.00)	1.25(1.22,1.28)	5
South Asia	3,929,224(3,672,984-4,212,721)	2	5,045,903(4,737,984-5,382,675)	2	1.39(1.35,1.43)	1.02(1.00,1.05)	0.18(0.15,0.20)	0.87(0.85,0.89)	9

Central Sub-Saharan Africa	124,324(112,638-137,510)	16	273,029(248,123-301,235)	14	2.86(2.85,2.87)	3.05(3.04,3.07)	2.28(2.23,2.33)	2.75(2.73,2.77)	2
Eastern Sub-Saharan Africa	409,253(383,233-437,261)	13	831,110(779,815-887,034)	8	2.68(2.61,2.75)	2.64(2.60,2.68)	2.08(2.04,2.12)	2.48(2.45,2.51)	4
Southern Sub-Saharan Africa	101,286(94,704-109,010)	19	129,378(121,579-138,491)	18	1.29(1.25,1.33)	0.57(0.53,0.60)	0.69(0.61,0.76)	0.84(0.81,0.87)	10
Western Sub-Saharan Africa	431,666(405,303-459,428)	11	964,390(910,096-1,023,571)	7	2.85(2.82,2.89)	3.18(3.15,3.20)	2.35(2.26,2.45)	2.82(2.79,2.85)	1

sTable 6. The incident cases and age-standardized rate of incidence attributable to six immune mediated inflammatory diseases according to countries and territories and its temporal trends from 1990 to 2019.

	1990		2019		1990-1999	2000-2009	2010-2019	1990-2019	
	**N(95%CI)**	**Rank**	**N(95%CI)**	**Rank**	**AAPC(95%CI)**	**AAPC(95%CI)**	**AAPC(95%CI)**	**AAPC(95%CI)**	**Rank**
**Age standardized rate**									
Asthma									
China	394.58(295.36-529.45)	186	355.33(259.68-492.64)	191	-2.72(-3.07,-2.38)	-1.86(-2.31,-1.40)	3.08(-1.62,8.00)	-0.55(-2.03,0.95)	144
Democratic People's Republic of Korea	542.62(426.50-688.82)	141	534.72(427.35-669.82)	119	0.66(0.48,0.85)	-0.45(-0.60,-0.29)	-0.47(-0.59,-0.35)	-0.11(-0.20,-0.02)	70
Taiwan (Province of China)	581.13(467.25-733.13)	118	543.56(414.25-731.18)	110	1.37(0.50,2.26)	-0.21(-0.35,-0.06)	-1.87(-3.44,-0.27)	-0.24(-0.81,0.34)	89
Cambodia	375.03(302.63-460.88)	190	414.91(329.42-525.97)	177	-0.71(-0.87,-0.56)	0.52(0.30,0.73)	1.46(1.22,1.70)	0.42(0.30,0.55)	7
Indonesia	583.45(477.68-717.07)	117	544.94(437.00-681.94)	107	-1.30(-1.49,-1.11)	0.75(0.49,1.00)	-0.34(-0.98,0.31)	-0.29(-0.52,-0.06)	98
Lao People's Democratic Republic	417.34(345.44-504.36)	181	368.90(303.60-453.78)	186	-0.69(-0.79,-0.60)	-0.63(-0.67,-0.59)	0.09(-0.01,0.19)	-0.44(-0.49,-0.39)	129
Malaysia	480.71(402.95-575.60)	161	433.51(339.20-569.29)	171	-0.68(-0.81,-0.55)	-0.03(-0.55,0.50)	-0.37(-1.08,0.34)	-0.41(-0.70,-0.11)	121
Maldives	595.28(484.98-737.89)	111	421.60(319.95-549.79)	174	-1.45(-1.53,-1.37)	-1.93(-1.97,-1.88)	0.06(-0.06,0.18)	-1.16(-1.21,-1.11)	195
Myanmar	384.93(329.60-452.56)	189	358.26(303.33-431.55)	189	-0.69(-0.75,-0.63)	-0.43(-0.46,-0.40)	0.46(0.38,0.53)	-0.24(-0.28,-0.21)	90
Philippines	904.92(720.28-1162.25)	28	742.69(590.99-952.72)	37	-1.13(-1.20,-1.06)	-0.51(-0.52,-0.49)	-0.35(-0.43,-0.26)	-0.68(-0.72,-0.65)	168
Sri Lanka	541.33(459.02-635.65)	142	563.80(474.79-681.31)	96	0.35(0.32,0.38)	-0.02(-0.08,0.04)	0.10(0.03,0.17)	0.13(0.10,0.16)	26
Thailand	561.66(470.99-678.15)	126	535.35(419.88-686.45)	116	-0.07(-0.18,0.03)	-1.20(-1.67,-0.73)	1.17(0.32,2.02)	-0.16(-0.49,0.17)	78
Timor-Leste	526.94(431.19-643.72)	146	483.86(395.99-593.85)	143	-0.61(-0.71,-0.50)	-0.53(-0.59,-0.48)	0.35(0.24,0.45)	-0.29(-0.34,-0.23)	99
Viet Nam	422.53(341.27-528.99)	176	485.57(391.94-612.69)	141	1.13(0.77,1.49)	-0.50(-0.86,-0.14)	0.90(0.65,1.16)	0.49(0.28,0.69)	4
Fiji	710.93(606.52-830.20)	65	540.26(451.09-655.47)	112	-0.64(-0.76,-0.52)	-1.82(-1.91,-1.72)	-0.24(-0.48,0.01)	-0.93(-1.03,-0.83)	183
Kiribati	886.31(756.86-1029.76)	32	696.82(599.47-812.23)	43	-0.53(-0.56,-0.50)	-1.12(-1.14,-1.10)	-0.77(-0.81,-0.73)	-0.82(-0.84,-0.80)	181
Marshall Islands	598.88(507.55-704.02)	109	511.09(407.53-635.32)	130	-1.17(-1.24,-1.10)	-1.17(-1.24,-1.10)	1.36(0.84,1.88)	-0.39(-0.56,-0.22)	116
Micronesia (Federated States of)	592.65(493.82-699.58)	113	528.02(411.24-662.60)	122	-1.11(-1.17,-1.05)	-1.11(-1.17,-1.05)	1.37(0.04,2.72)	-0.35(-0.76,0.07)	105
Papua New Guinea	847.78(717.77-982.73)	42	704.16(603.93-818.13)	41	-0.06(-0.11,-0.02)	-1.14(-1.25,-1.04)	-0.73(-0.78,-0.68)	-0.66(-0.70,-0.62)	163
Samoa	555.93(455.26-675.20)	132	498.73(391.04-635.58)	137	-1.06(-1.12,-1.00)	-0.77(-0.78,-0.76)	0.85(0.68,1.01)	-0.36(-0.42,-0.31)	107
Solomon Islands	593.79(488.63-708.38)	112	567.36(455.13-713.71)	95	-0.88(-1.66,-0.10)	-0.95(-1.15,-0.74)	1.88(1.39,2.38)	-0.06(-0.36,0.24)	60
Tonga	683.33(555.51-857.68)	74	599.07(467.86-762.30)	77	-0.49(-0.96,-0.02)	-1.38(-1.55,-1.20)	0.74(0.39,1.10)	-0.39(-0.60,-0.18)	117
Vanuatu	549.66(454.61-656.06)	137	524.55(380.17-684.89)	125	-1.42(-1.51,-1.32)	-1.42(-1.51,-1.32)	3.12(0.91,5.37)	-0.03(-0.70,0.65)	56
Armenia	333.69(253.18-432.43)	197	352.22(257.28-462.20)	192	-0.47(-0.63,-0.32)	0.19(0.16,0.22)	1.01(0.83,1.18)	0.23(0.16,0.31)	16
Azerbaijan	351.95(275.37-451.46)	195	343.45(261.91-445.74)	195	-0.65(-0.75,-0.54)	-0.33(-0.46,-0.19)	1.03(0.65,1.40)	0.00(-0.15,0.16)	50
Georgia	343.46(279.10-431.30)	196	342.53(257.82-452.18)	196	-0.42(-0.96,0.13)	0.68(0.15,1.20)	0.06(-0.29,0.42)	0.06(-0.21,0.34)	37
Kazakhstan	279.75(219.89-350.81)	201	285.86(219.41-366.73)	199	-1.25(-1.45,-1.05)	0.05(-0.03,0.14)	1.59(1.36,1.83)	0.13(0.01,0.24)	27
Kyrgyzstan	476.68(388.55-594.20)	165	394.33(299.15-513.70)	180	-1.20(-1.41,-0.98)	-0.86(-0.96,-0.76)	0.16(-0.20,0.51)	-0.66(-0.80,-0.52)	164
Mongolia	408.26(332.10-498.65)	182	361.35(279.74-462.22)	188	-0.73(-0.86,-0.61)	-0.70(-0.72,-0.68)	0.25(0.18,0.33)	-0.42(-0.46,-0.37)	124
Tajikistan	407.07(325.40-503.26)	184	385.31(297.29-491.32)	183	-0.72(-0.90,-0.53)	-0.44(-0.50,-0.37)	0.67(0.49,0.85)	-0.17(-0.27,-0.08)	80
Turkmenistan	432.38(352.21-540.56)	174	344.47(254.91-456.33)	194	-1.62(-1.72,-1.52)	-1.55(-1.81,-1.30)	1.01(0.80,1.23)	-0.74(-0.85,-0.62)	174
Uzbekistan	560.81(454.97-685.96)	128	469.12(372.19-595.28)	151	-0.29(-0.39,-0.20)	-1.53(-1.63,-1.43)	0.09(-0.06,0.24)	-0.64(-0.71,-0.56)	159

Albania	418.16(350.48-518.12)	180	453.34(361.82-573.26)	160	-0.65(-0.82,-0.48)	1.13(0.83,1.43)	0.44(-0.37,1.26)	0.32(0.04,0.60)	10
Bosnia and Herzegovina	638.77(518.91-795.32)	96	636.76(506.83-806.55)	63	-0.08(-0.19,0.04)	-0.04(-0.26,0.19)	0.05(0.02,0.08)	-0.01(-0.09,0.07)	53
Bulgaria	580.52(475.61-716.11)	119	535.93(418.26-677.37)	114	-0.81(-1.18,-0.43)	-0.15(-0.27,-0.03)	0.24(0.13,0.34)	-0.27(-0.42,-0.11)	93
Croatia	694.89(562.89-854.67)	70	585.73(465.67-736.93)	85	-0.88(-1.11,-0.65)	-0.83(-0.92,-0.74)	0.15(-0.12,0.41)	-0.58(-0.70,-0.45)	147
Czechia	471.45(370.23-598.68)	166	472.29(368.37-605.33)	148	-0.40(-0.64,-0.16)	-0.06(-0.15,0.04)	0.71(0.50,0.93)	0.05(-0.08,0.17)	38
Hungary	559.96(450.26-694.95)	129	501.38(392.64-635.74)	135	-1.01(-1.48,-0.55)	0.13(-0.40,0.67)	-0.22(-0.51,0.07)	-0.41(-0.66,-0.16)	122
North Macedonia	848.06(674.93-1056.58)	41	709.20(584.54-868.07)	40	-0.14(-0.47,0.18)	-1.66(-2.03,-1.29)	0.00(-0.08,0.08)	-0.62(-0.79,-0.45)	157
Montenegro	495.01(385.18-630.44)	157	537.87(421.17-693.12)	113	-0.21(-0.53,0.10)	0.19(0.12,0.27)	1.09(0.81,1.37)	0.34(0.21,0.48)	9
Poland	1065.27(883.56-1281.61)	8	780.71(606.61-1005.19)	31	-0.85(-1.10,-0.59)	-1.75(-1.86,-1.63)	-0.65(-0.77,-0.52)	-1.12(-1.24,-1.01)	192
Romania	597.20(481.50-757.78)	110	601.56(468.94-768.93)	76	0.28(0.15,0.42)	-0.12(-0.25,0.02)	-0.03(-0.15,0.09)	0.02(-0.06,0.11)	43
Serbia	483.07(391.95-598.76)	159	451.53(356.39-571.51)	162	-0.13(-0.18,-0.08)	-0.46(-0.47,-0.44)	-0.04(-0.10,0.01)	-0.23(-0.25,-0.20)	86
Slovakia	455.71(361.64-574.77)	169	455.76(355.33-579.07)	158	-0.36(-0.45,-0.27)	0.11(-0.08,0.30)	0.22(-0.21,0.66)	-0.01(-0.16,0.15)	54
Slovenia	748.63(617.57-913.99)	56	645.40(509.37-818.18)	61	-0.57(-0.69,-0.44)	-0.73(-0.76,-0.70)	-0.13(-0.26,0.01)	-0.49(-0.55,-0.43)	134
Belarus	685.00(551.35-845.20)	73	534.85(406.63-698.11)	118	-1.08(-1.28,-0.88)	-1.79(-1.94,-1.64)	0.61(0.32,0.89)	-0.81(-0.95,-0.68)	180
Estonia	431.52(346.92-525.58)	175	363.10(270.06-472.99)	187	-0.37(-0.50,-0.24)	-2.86(-3.26,-2.46)	2.15(1.31,3.00)	-0.54(-0.84,-0.23)	142
Latvia	610.39(512.40-742.95)	107	508.56(382.27-659.87)	133	-1.76(-1.81,-1.71)	0.09(-0.03,0.22)	0.01(-0.11,0.13)	-0.59(-0.65,-0.54)	152
Lithuania	469.52(379.23-579.78)	167	448.71(333.41-581.33)	165	-1.44(-1.54,-1.33)	1.00(0.73,1.28)	0.34(0.10,0.59)	-0.10(-0.22,0.03)	68
Republic of Moldova	530.18(421.20-676.84)	144	453.48(339.57-600.80)	159	-0.71(-0.76,-0.66)	-1.03(-1.12,-0.94)	0.22(0.12,0.33)	-0.52(-0.57,-0.46)	139
Russian Federation	657.99(527.09-833.14)	83	434.05(319.40-575.98)	170	-1.22(-1.41,-1.02)	-2.86(-3.04,-2.68)	0.12(-0.27,0.51)	-1.40(-1.57,-1.23)	202
Ukraine	682.78(515.12-875.35)	75	552.68(398.88-743.62)	102	-0.94(-1.17,-0.72)	-1.67(-1.86,-1.47)	0.81(0.45,1.16)	-0.67(-0.84,-0.50)	167
Brunei Darussalam	669.84(547.83-827.37)	79	622.60(504.15-773.55)	69	0.37(0.13,0.60)	-0.76(-0.83,-0.70)	-0.30(-0.46,-0.14)	-0.27(-0.36,-0.18)	94
Japan	963.11(785.39-1201.19)	19	584.11(439.70-775.33)	86	-2.32(-2.48,-2.16)	-4.23(-4.30,-4.15)	1.46(1.05,1.87)	-1.73(-1.87,-1.59)	204
Republic of Korea	586.87(486.52-715.45)	114	510.52(400.45-652.89)	131	-0.89(-1.10,-0.68)	-0.01(-0.22,0.21)	-0.42(-0.48,-0.35)	-0.51(-0.62,-0.41)	137
Singapore	693.90(575.23-830.60)	71	500.44(386.25-653.54)	136	-1.82(-2.01,-1.63)	-1.16(-1.29,-1.04)	-0.21(-0.30,-0.11)	-1.12(-1.21,-1.03)	193
Australia	748.63(585.44-918.13)	57	598.70(458.18-769.55)	78	0.69(0.51,0.88)	-3.35(-3.63,-3.07)	0.17(-0.26,0.61)	-0.79(-0.98,-0.60)	178
New Zealand	967.25(754.33-1222.76)	16	702.45(520.34-936.85)	42	-1.67(-1.87,-1.47)	-1.22(-1.38,-1.07)	-0.31(-0.39,-0.23)	-1.12(-1.22,-1.03)	194
Andorra	525.67(417.32-655.61)	147	484.22(374.98-612.01)	142	-0.36(-0.40,-0.31)	-0.57(-0.59,-0.55)	0.11(0.05,0.17)	-0.28(-0.31,-0.25)	95
Austria	525.10(439.60-633.31)	148	449.81(351.51-562.99)	163	-0.94(-1.17,-0.70)	-0.18(-0.34,-0.02)	-0.54(-0.72,-0.35)	-0.59(-0.70,-0.47)	153
Belgium	547.28(461.73-657.80)	140	417.30(322.27-519.50)	176	-2.76(-3.33,-2.20)	0.90(0.51,1.29)	-0.90(-1.22,-0.58)	-1.08(-1.33,-0.82)	190
Cyprus	568.60(456.94-703.71)	122	546.48(432.11-683.41)	106	-0.80(-0.84,-0.76)	0.01(0.00,0.02)	0.33(0.28,0.39)	-0.13(-0.15,-0.10)	74
Denmark	549.94(450.69-675.35)	136	467.94(359.21-597.18)	152	-0.77(-0.93,-0.62)	-0.30(-0.79,0.20)	-0.94(-1.86,-0.02)	-0.64(-0.99,-0.29)	160
Finland	487.44(396.71-601.07)	158	456.87(361.73-567.13)	157	0.08(0.02,0.14)	-0.09(-0.21,0.03)	-0.58(-0.69,-0.46)	-0.22(-0.27,-0.16)	85
France	646.62(544.89-786.56)	91	522.36(410.41-651.31)	127	-1.38(-1.83,-0.92)	-0.88(-0.94,-0.82)	0.05(-0.37,0.47)	-0.75(-0.94,-0.55)	175
Germany	558.74(465.31-660.93)	131	390.99(300.53-495.42)	182	-3.79(-4.38,-3.20)	0.81(0.24,1.37)	-0.92(-1.09,-0.74)	-1.36(-1.64,-1.08)	201
Greece	464.12(378.44-575.60)	168	451.83(352.34-563.85)	161	-0.66(-0.88,-0.44)	0.72(0.61,0.82)	-0.42(-0.52,-0.33)	-0.11(-0.19,-0.02)	71
Iceland	857.85(676.21-1065.53)	39	695.50(545.90-881.69)	44	-0.56(-1.03,-0.08)	-1.46(-1.70,-1.22)	-0.18(-0.53,0.16)	-0.72(-0.94,-0.49)	173
Ireland	693.67(571.77-838.77)	72	535.46(420.28-664.27)	115	-1.07(-1.24,-0.91)	-0.64(-0.90,-0.39)	-1.15(-1.87,-0.43)	-0.95(-1.20,-0.70)	185
Israel	519.52(420.37-637.78)	152	439.70(339.13-548.97)	167	-1.14(-1.25,-1.04)	-0.52(-0.56,-0.48)	0.08(0.01,0.16)	-0.58(-0.63,-0.53)	148

Italy	522.30(430.38-634.44)	150	447.73(330.69-574.70)	166	-1.02(-1.63,-0.41)	-0.37(-1.66,0.95)	-0.47(-1.01,0.07)	-0.68(-1.17,-0.18)	169
Luxembourg	638.97(508.83-819.85)	95	526.32(409.87-657.79)	123	-0.27(-0.45,-0.09)	-1.13(-1.18,-1.09)	-0.59(-0.77,-0.40)	-0.70(-0.78,-0.61)	171
Malta	630.61(528.06-753.98)	100	543.14(427.58-675.99)	111	-0.78(-0.92,-0.64)	-0.42(-0.46,-0.39)	-0.28(-0.51,-0.05)	-0.53(-0.62,-0.44)	141
Netherlands	363.72(303.17-436.48)	193	332.70(270.47-401.54)	197	0.13(-0.24,0.50)	-1.67(-1.79,-1.55)	1.03(0.91,1.15)	-0.28(-0.41,-0.15)	96
Norway	901.39(737.96-1101.35)	29	650.21(498.29-826.89)	59	-1.07(-1.19,-0.94)	-1.40(-1.57,-1.23)	-0.88(-1.45,-0.31)	-1.16(-1.40,-0.93)	196
Portugal	724.55(600.98-891.19)	62	636.31(503.81-787.32)	64	-1.35(-1.85,-0.85)	1.39(1.09,1.68)	-1.18(-1.40,-0.95)	-0.55(-0.76,-0.34)	145
Spain	447.14(361.68-556.63)	171	463.66(364.70-581.08)	155	-0.93(-1.03,-0.84)	1.58(1.46,1.71)	-0.38(-0.76,0.00)	0.11(-0.02,0.24)	29
Sweden	839.68(688.11-1029.83)	45	611.44(467.07-789.49)	73	-3.04(-3.16,-2.91)	-1.64(-1.96,-1.31)	1.38(0.57,2.19)	-1.16(-1.44,-0.88)	197
Switzerland	620.67(503.78-761.07)	103	525.15(411.55-668.59)	124	-0.70(-0.73,-0.67)	-1.05(-1.08,-1.02)	0.04(-0.03,0.11)	-0.58(-0.61,-0.55)	149
United Kingdom	989.29(792.45-1209.39)	14	757.03(585.46-955.27)	34	-1.05(-2.08,0.00)	-1.03(-1.16,-0.89)	-1.03(-1.16,-0.89)	-1.03(-1.37,-0.70)	189
Argentina	760.86(633.95-926.74)	54	781.45(620.88-986.36)	30	0.26(0.17,0.34)	0.31(0.27,0.36)	-0.26(-0.31,-0.20)	0.08(0.04,0.12)	33
Chile	653.41(540.02-801.02)	86	645.84(514.69-820.60)	60	0.14(0.03,0.25)	-0.75(-0.86,-0.63)	0.73(0.53,0.92)	0.01(-0.08,0.10)	48
Uruguay	754.13(620.16-924.96)	55	691.25(549.72-873.41)	45	-0.59(-0.70,-0.47)	-0.41(-0.83,0.00)	0.09(-0.11,0.28)	-0.33(-0.50,-0.17)	104
Canada	845.76(671.64-1070.35)	43	783.77(611.28-994.67)	29	0.32(-0.16,0.81)	0.54(0.37,0.72)	-1.15(-1.72,-0.57)	-0.17(-0.44,0.09)	81
United States of America	1404.61(1077.50-1842.92)	1	1547.24(1251.20-1899.94)	1	-2.87(-3.29,-2.45)	2.62(2.33,2.91)	1.12(0.87,1.37)	0.40(0.20,0.61)	8
Antigua and Barbuda	875.33(675.98-1143.74)	37	918.52(698.96-1177.53)	17	-0.06(-0.36,0.24)	0.02(-0.08,0.12)	0.69(0.47,0.92)	0.22(0.09,0.35)	18
Bahamas	882.20(685.43-1145.21)	33	918.06(697.41-1185.11)	18	0.03(-0.24,0.31)	-0.08(-0.17,0.00)	0.60(0.41,0.80)	0.19(0.07,0.31)	20
Barbados	951.02(764.25-1192.76)	21	1028.32(795.41-1318.23)	7	0.09(0.02,0.16)	0.76(0.63,0.88)	-0.17(-0.50,0.17)	0.25(0.13,0.36)	12
Belize	948.57(740.74-1194.64)	22	906.64(693.53-1157.77)	20	-0.07(-0.47,0.32)	-0.41(-0.47,-0.34)	0.23(-0.13,0.59)	-0.11(-0.28,0.07)	72
Cuba	1045.33(814.05-1325.04)	10	1047.77(802.46-1343.96)	6	0.03(-0.04,0.10)	-0.19(-0.34,-0.05)	0.29(0.16,0.41)	0.04(-0.03,0.11)	39
Dominica	964.32(762.74-1212.97)	18	1012.45(789.50-1281.00)	9	0.65(0.52,0.79)	0.01(-0.05,0.06)	-0.10(-0.22,0.02)	0.19(0.12,0.25)	21
Dominican Republic	706.06(557.48-873.94)	67	687.85(533.14-892.07)	46	-1.61(-1.86,-1.36)	0.93(0.70,1.16)	0.71(0.14,1.28)	0.00(-0.22,0.23)	51
Grenada	1023.66(788.30-1307.80)	12	1059.01(824.14-1363.18)	5	0.08(0.03,0.13)	-0.06(-0.08,-0.04)	0.36(0.31,0.41)	0.12(0.09,0.14)	28
Guyana	964.67(769.58-1199.63)	17	945.61(743.84-1197.21)	11	0.23(0.07,0.39)	-0.58(-0.64,-0.51)	0.25(0.13,0.37)	-0.05(-0.13,0.02)	59
Haiti	1182.75(943.01-1447.88)	7	1061.05(847.11-1313.37)	3	-0.34(-0.41,-0.27)	-0.75(-0.79,-0.71)	0.06(-0.02,0.13)	-0.36(-0.41,-0.32)	108
Jamaica	1038.21(814.51-1324.25)	11	925.34(713.71-1194.11)	14	-0.22(-0.38,-0.07)	-0.89(-0.92,-0.85)	0.05(-0.44,0.53)	-0.39(-0.55,-0.23)	118
Saint Lucia	1014.96(803.87-1272.23)	13	999.01(777.10-1265.24)	10	0.35(0.30,0.39)	-0.55(-0.57,-0.52)	0.13(-0.08,0.33)	-0.06(-0.13,0.00)	61
Saint Vincent and the Grenadines	907.49(711.72-1161.34)	27	920.12(710.22-1172.12)	16	-0.23(-0.40,-0.06)	-0.10(-0.21,0.02)	0.65(0.45,0.85)	0.09(-0.01,0.20)	32
Suriname	878.96(684.54-1138.77)	34	885.31(678.41-1145.34)	22	-0.19(-0.20,-0.17)	-0.19(-0.20,-0.17)	0.54(0.32,0.76)	0.04(-0.03,0.11)	40
Trinidad and Tobago	723.46(567.58-925.00)	63	801.89(604.25-1052.94)	28	-0.15(-0.28,-0.03)	-0.15(-0.28,-0.03)	2.24(1.46,3.02)	0.58(0.32,0.84)	3
Bolivia (Plurinational State of)	915.01(693.30-1157.15)	26	768.28(577.27-1028.77)	33	-0.80(-0.99,-0.61)	-1.04(-1.12,-0.96)	0.27(0.08,0.45)	-0.59(-0.68,-0.49)	154
Ecuador	877.93(656.25-1142.75)	35	728.08(535.41-985.52)	38	-1.53(-2.05,-1.01)	-0.99(-1.57,-0.42)	0.87(0.74,1.00)	-0.66(-0.93,-0.38)	165
Peru	956.31(715.69-1242.62)	20	774.98(573.45-1051.45)	32	-0.66(-0.81,-0.52)	-2.08(-2.29,-1.86)	0.68(0.53,0.83)	-0.79(-0.90,-0.68)	179
Colombia	761.12(595.29-965.90)	53	613.08(452.56-809.54)	71	-1.72(-2.61,-0.81)	-0.69(-1.02,-0.36)	0.38(0.26,0.51)	-0.69(-1.00,-0.37)	170
Costa Rica	969.86(757.97-1219.63)	15	847.63(642.09-1101.32)	26	0.79(0.63,0.95)	-1.77(-2.38,-1.15)	-0.08(-0.34,0.17)	-0.42(-0.65,-0.19)	125
El Salvador	1212.32(933.75-1536.01)	5	844.10(652.44-1093.89)	27	-1.34(-1.48,-1.21)	-1.90(-2.00,-1.80)	-0.34(-0.47,-0.22)	-1.27(-1.34,-1.19)	200
Guatemala	855.42(659.56-1065.97)	40	530.28(397.11-705.03)	120	-2.05(-2.34,-1.75)	-2.60(-3.25,-1.94)	-0.38(-0.48,-0.28)	-1.71(-1.95,-1.46)	203
Honduras	890.59(683.83-1123.42)	31	662.58(505.46-863.27)	54	-0.72(-0.95,-0.49)	-1.73(-1.79,-1.66)	-0.43(-0.69,-0.17)	-1.01(-1.13,-0.90)	187

Mexico	606.68(466.31-796.02)	108	523.83(370.22-712.36)	126	-2.02(-2.32,-1.72)	-0.51(-0.78,-0.25)	1.27(1.19,1.36)	-0.52(-0.67,-0.37)	140
Nicaragua	915.63(708.01-1175.31)	25	653.10(490.42-859.18)	57	-1.50(-2.19,-0.81)	-1.28(-1.60,-0.96)	-0.73(-0.82,-0.64)	-1.20(-1.45,-0.94)	199
Panama	860.31(674.27-1078.62)	38	748.52(566.80-977.71)	36	-0.28(-0.47,-0.09)	-0.80(-0.89,-0.71)	-0.28(-0.50,-0.06)	-0.48(-0.59,-0.37)	133
Venezuela (Bolivarian Republic of)	770.56(592.13-991.30)	51	668.58(500.46-871.93)	53	-0.53(-0.66,-0.40)	-0.84(-0.89,-0.78)	0.06(-0.05,0.17)	-0.50(-0.56,-0.43)	135
Brazil	1053.29(774.10-1398.37)	9	916.35(643.52-1221.96)	19	-0.67(-0.94,-0.40)	-1.42(-1.54,-1.30)	0.85(0.53,1.16)	-0.50(-0.65,-0.35)	136
Paraguay	810.59(610.76-1066.71)	49	924.14(682.94-1226.46)	15	0.41(0.38,0.43)	0.41(0.38,0.43)	0.66(0.51,0.82)	0.49(0.43,0.54)	5
Algeria	506.60(414.46-621.58)	155	534.87(419.08-677.12)	117	-0.43(-0.55,-0.31)	0.08(-0.26,0.42)	1.14(0.94,1.35)	0.22(0.09,0.36)	19
Bahrain	656.32(531.21-810.75)	85	586.34(459.84-738.65)	84	-0.15(-0.25,-0.06)	-0.91(-0.94,-0.88)	-0.01(-0.09,0.07)	-0.36(-0.41,-0.32)	109
Egypt	584.67(471.33-723.49)	116	554.85(444.07-692.03)	100	-0.65(-0.71,-0.58)	-0.48(-0.51,-0.45)	0.71(0.63,0.79)	-0.16(-0.20,-0.12)	79
Iran (Islamic Republic of)	646.00(516.21-800.13)	92	544.09(418.92-689.93)	109	-0.15(-0.23,-0.07)	-1.32(-1.38,-1.25)	-0.21(-0.28,-0.14)	-0.61(-0.66,-0.57)	156
Iraq	696.37(567.01-848.57)	69	555.57(436.74-709.95)	99	-0.88(-0.90,-0.86)	-0.97(-0.98,-0.96)	-0.45(-0.48,-0.42)	-0.78(-0.80,-0.77)	177
Jordan	631.38(501.24-795.96)	99	629.02(485.06-805.67)	67	0.01(-0.11,0.12)	-0.31(-0.35,-0.28)	0.35(0.24,0.47)	0.02(-0.04,0.08)	44
Kuwait	659.01(521.58-822.27)	82	641.21(484.83-830.32)	62	-0.66(-0.70,-0.62)	0.18(0.08,0.28)	0.16(-0.04,0.36)	-0.09(-0.17,-0.02)	67
Lebanon	661.01(538.02-821.45)	81	671.71(533.78-842.60)	51	-0.08(-0.14,-0.02)	-0.12(-0.16,-0.08)	0.46(0.38,0.55)	0.07(0.04,0.11)	34
Libya	622.76(501.50-758.50)	102	589.80(467.82-727.84)	83	-0.53(-0.69,-0.37)	-0.21(-0.27,-0.15)	0.29(0.13,0.45)	-0.18(-0.26,-0.09)	82
Morocco	451.35(365.75-551.69)	170	487.80(390.15-613.38)	139	0.02(-0.05,0.09)	-0.04(-0.08,0.00)	0.91(0.82,1.00)	0.28(0.23,0.32)	11
Palestine	558.87(448.40-693.06)	130	547.77(427.08-693.22)	104	-0.96(-1.09,-0.82)	-0.03(-0.08,0.03)	0.91(0.75,1.07)	-0.03(-0.10,0.04)	57
Oman	480.51(378.41-606.50)	162	616.57(477.55-787.33)	70	-0.35(-0.50,-0.21)	1.72(1.52,1.92)	1.47(0.87,2.08)	0.93(0.73,1.14)	1
Qatar	551.73(425.25-709.91)	134	554.69(429.44-710.98)	101	0.21(0.14,0.27)	-0.66(-0.74,-0.57)	0.60(0.27,0.92)	0.02(-0.08,0.13)	45
Saudi Arabia	373.60(298.39-461.44)	191	461.34(354.28-587.67)	156	0.02(-0.13,0.17)	1.44(1.02,1.87)	0.89(0.42,1.37)	0.74(0.54,0.95)	2
Syrian Arab Republic	540.62(443.66-655.86)	143	544.30(439.39-668.84)	108	-1.14(-1.81,-0.48)	1.28(0.87,1.69)	-0.21(-0.68,0.26)	0.00(-0.32,0.32)	52
Tunisia	562.55(462.36-695.76)	125	578.04(459.74-725.11)	90	0.14(0.02,0.27)	-0.31(-0.38,-0.24)	0.49(0.39,0.59)	0.10(0.03,0.16)	30
Turkey	741.21(608.55-890.91)	59	718.96(590.99-869.31)	39	0.02(-0.14,0.18)	-1.65(-2.15,-1.15)	1.88(1.64,2.12)	-0.03(-0.23,0.18)	58
United Arab Emirates	941.45(767.48-1133.68)	23	905.80(736.37-1107.77)	21	0.15(-0.26,0.56)	1.61(-0.90,4.19)	-2.87(-3.85,-1.88)	-0.37(-1.26,0.53)	111
Yemen	681.80(568.54-817.92)	77	608.20(485.94-750.18)	75	-0.57(-0.64,-0.49)	-0.67(-0.72,-0.61)	0.16(0.05,0.26)	-0.37(-0.42,-0.32)	112
Afghanistan	652.33(543.81-774.52)	87	631.24(521.18-764.55)	66	-0.53(-0.63,-0.43)	-0.15(-0.17,-0.13)	0.38(0.31,0.45)	-0.10(-0.14,-0.07)	69
Bangladesh	266.77(231.74-305.57)	202	207.54(178.04-243.32)	203	-0.24(-0.49,0.01)	-1.97(-2.13,-1.80)	-0.44(-0.62,-0.26)	-0.94(-1.07,-0.81)	184
Bhutan	291.80(253.79-335.73)	200	215.15(185.60-252.43)	202	-0.62(-0.71,-0.53)	-1.75(-1.84,-1.66)	-0.76(-0.94,-0.59)	-1.09(-1.16,-1.02)	191
India	419.33(349.81-500.80)	179	356.64(294.29-428.24)	190	-4.00(-4.88,-3.11)	2.75(1.14,4.38)	-0.55(-1.28,0.18)	-0.65(-1.27,-0.01)	162
Nepal	204.91(179.58-233.76)	204	175.47(150.18-204.47)	204	-0.36(-0.51,-0.21)	-1.88(-2.22,-1.54)	1.12(0.64,1.60)	-0.46(-0.66,-0.25)	130
Pakistan	309.23(259.17-367.14)	199	252.16(211.53-304.07)	200	-0.04(-0.70,0.62)	-1.44(-1.85,-1.03)	-0.41(-0.72,-0.10)	-0.56(-0.84,-0.28)	146
Angola	682.64(561.74-825.83)	76	528.88(422.49-651.46)	121	-0.40(-0.43,-0.37)	-1.46(-1.47,-1.44)	-0.74(-0.78,-0.70)	-0.88(-0.91,-0.86)	182
Central African Republic	650.85(540.38-785.92)	88	562.22(460.23-683.20)	97	-0.47(-0.51,-0.42)	-0.60(-0.61,-0.59)	-0.44(-0.48,-0.41)	-0.51(-0.53,-0.49)	138
Congo	656.84(535.27-801.11)	84	593.11(475.02-745.68)	81	-0.34(-0.49,-0.18)	-0.78(-0.92,-0.64)	0.11(-0.02,0.24)	-0.38(-0.47,-0.28)	113
Democratic Republic of the Congo	550.35(446.13-670.50)	135	508.57(412.31-631.68)	132	-0.56(-0.59,-0.52)	-0.31(-0.39,-0.24)	0.09(0.03,0.15)	-0.28(-0.31,-0.25)	97
Equatorial Guinea	623.00(505.59-746.76)	101	517.61(408.71-656.97)	128	-0.78(-0.85,-0.72)	-1.30(-1.42,-1.19)	0.41(0.11,0.72)	-0.60(-0.70,-0.49)	155
Gabon	548.78(442.70-669.70)	138	478.15(375.30-606.14)	146	-0.88(-0.90,-0.86)	-0.85(-0.87,-0.83)	0.42(0.18,0.65)	-0.46(-0.54,-0.39)	131
Burundi	833.14(677.28-1020.36)	46	669.14(533.39-821.81)	52	-0.83(-0.87,-0.78)	-0.74(-0.85,-0.63)	-0.69(-0.81,-0.57)	-0.77(-0.82,-0.71)	176

Comoros	726.91(588.06-904.17)	61	655.60(519.00-822.06)	56	-0.77(-0.80,-0.74)	-0.60(-0.62,-0.59)	0.35(0.30,0.40)	-0.35(-0.37,-0.33)	106
Djibouti	787.84(633.81-975.95)	50	661.22(529.76-835.01)	55	-0.83(-0.86,-0.80)	-0.97(-1.05,-0.89)	0.16(0.00,0.32)	-0.58(-0.64,-0.52)	150
Eritrea	763.70(622.29-936.04)	52	680.07(545.48-847.25)	48	-0.57(-0.60,-0.54)	-0.82(-0.89,-0.75)	0.25(0.12,0.39)	-0.41(-0.46,-0.36)	123
Ethiopia	611.11(489.95-764.23)	106	505.60(383.13-660.03)	134	-1.12(-1.38,-0.85)	-1.09(-1.15,-1.02)	0.47(0.28,0.66)	-0.62(-0.72,-0.51)	158
Kenya	548.20(429.89-699.47)	139	483.58(373.46-631.84)	144	-0.94(-1.14,-0.73)	-0.73(-0.84,-0.62)	0.60(0.36,0.83)	-0.39(-0.51,-0.27)	119
Madagascar	1190.13(963.54-1490.45)	6	859.32(699.05-1043.76)	25	-0.53(-0.74,-0.31)	-2.02(-2.24,-1.81)	-0.79(-0.93,-0.64)	-1.18(-1.30,-1.06)	198
Malawi	638.55(503.42-791.42)	97	610.65(483.48-770.87)	74	-0.51(-0.60,-0.43)	-0.38(-0.41,-0.35)	0.46(0.39,0.53)	-0.15(-0.19,-0.11)	77
Mauritius	620.04(510.55-749.52)	104	464.66(366.22-586.49)	154	-0.73(-0.81,-0.65)	-1.45(-1.49,-1.41)	-0.70(-0.83,-0.58)	-1.00(-1.05,-0.95)	186
Mozambique	698.04(569.80-863.33)	68	673.40(540.14-839.70)	50	-0.25(-0.27,-0.23)	-0.43(-0.44,-0.42)	0.38(0.34,0.43)	-0.12(-0.14,-0.11)	73
Rwanda	1290.82(1056.01-1592.12)	4	1060.12(853.89-1311.28)	4	-0.72(-0.81,-0.63)	-0.48(-0.61,-0.35)	-0.83(-1.10,-0.55)	-0.70(-0.80,-0.60)	172
Seychelles	407.54(326.86-511.56)	183	423.40(331.97-546.15)	173	-0.22(-0.34,-0.09)	-0.03(-0.07,0.02)	0.78(0.65,0.90)	0.17(0.10,0.23)	23
Somalia	832.01(666.99-1020.99)	47	686.78(559.37-847.08)	47	-0.80(-0.89,-0.72)	-1.13(-1.18,-1.08)	-0.10(-0.14,-0.06)	-0.66(-0.69,-0.62)	166
United Republic of Tanzania	875.62(696.64-1101.24)	36	862.07(681.57-1078.99)	24	-0.25(-0.35,-0.15)	0.60(0.45,0.75)	-0.38(-0.59,-0.16)	-0.06(-0.15,0.04)	62
Uganda	840.67(673.36-1042.16)	44	750.58(592.98-954.96)	35	-0.48(-0.49,-0.47)	-0.71(-0.76,-0.67)	0.15(0.08,0.22)	-0.38(-0.41,-0.35)	114
Zambia	421.55(339.74-519.82)	177	427.73(328.36-544.79)	172	-1.36(-1.49,-1.22)	0.02(-0.05,0.09)	1.67(1.01,2.34)	0.10(-0.11,0.31)	31
Botswana	389.48(320.00-469.11)	188	435.99(335.61-567.31)	168	-0.23(-0.49,0.02)	0.37(-0.40,1.15)	0.69(0.47,0.91)	0.25(-0.02,0.52)	13
Lesotho	247.35(204.63-297.19)	203	247.41(206.92-295.77)	201	-0.61(-0.75,-0.47)	-0.05(-0.12,0.02)	0.80(0.66,0.93)	0.04(-0.04,0.11)	41
Namibia	390.33(323.95-470.67)	187	350.99(281.11-432.65)	193	-0.42(-0.50,-0.34)	-0.88(-0.92,-0.84)	0.31(0.19,0.43)	-0.36(-0.41,-0.30)	110
South Africa	668.65(487.87-866.72)	80	579.84(417.36-771.38)	89	0.73(-0.08,1.55)	-6.75(-9.20,-4.23)	7.06(5.87,8.27)	0.07(-0.92,1.06)	35
Eswatini	671.21(555.84-813.15)	78	612.82(500.88-744.23)	72	0.49(0.35,0.63)	-0.90(-1.00,-0.80)	-0.62(-0.99,-0.25)	-0.38(-0.51,-0.25)	115
Zimbabwe	318.54(262.03-386.57)	198	327.66(267.93-397.58)	198	-0.66(-0.75,-0.57)	0.05(-0.02,0.12)	1.04(0.95,1.14)	0.14(0.09,0.18)	25
Benin	515.83(419.33-635.49)	153	469.79(371.60-584.66)	150	-0.76(-0.96,-0.56)	-0.48(-0.50,-0.45)	0.37(0.28,0.45)	-0.30(-0.37,-0.24)	100
Burkina Faso	444.04(353.04-554.78)	172	449.76(353.22-577.78)	164	-0.34(-0.39,-0.28)	-0.04(-0.30,0.22)	0.57(0.46,0.67)	0.04(-0.06,0.13)	42
Cameroon	441.21(361.67-546.31)	173	412.72(325.55-523.00)	178	-1.21(-1.26,-1.16)	0.01(-0.09,0.12)	0.62(0.53,0.72)	-0.23(-0.28,-0.18)	87
Cabo Verde	479.70(383.02-595.11)	163	435.21(333.47-566.85)	169	-1.42(-1.45,-1.39)	-0.43(-0.46,-0.40)	1.00(0.90,1.11)	-0.30(-0.34,-0.26)	101
Chad	400.98(323.68-495.27)	185	391.06(311.45-492.20)	181	-0.85(-1.07,-0.62)	0.00(-0.03,0.03)	0.60(0.50,0.70)	-0.08(-0.16,0.00)	66
C??te d'Ivoire	529.01(430.54-659.13)	145	514.61(403.90-648.43)	129	0.02(-0.22,0.26)	-0.52(-0.63,-0.41)	0.28(0.04,0.51)	-0.07(-0.20,0.06)	65
Gambia	506.64(409.79-622.85)	154	470.42(370.74-584.81)	149	-0.96(-1.09,-0.84)	-0.27(-0.30,-0.23)	0.61(0.52,0.70)	-0.25(-0.30,-0.19)	91
Ghana	363.35(286.51-462.48)	194	380.29(295.37-485.33)	185	-0.33(-0.40,-0.27)	-0.05(-0.08,-0.02)	1.00(0.89,1.10)	0.19(0.15,0.23)	22
Guinea	574.00(466.95-719.75)	121	546.83(441.45-689.13)	105	-0.04(-0.14,0.06)	-0.74(-0.79,-0.69)	0.35(0.23,0.47)	-0.18(-0.24,-0.13)	83
Guinea-Bissau	568.27(463.73-687.48)	123	496.25(400.43-610.59)	138	-0.89(-0.98,-0.79)	-0.67(-0.71,-0.63)	0.22(0.13,0.31)	-0.47(-0.52,-0.43)	132
Liberia	479.29(380.36-608.83)	164	420.78(325.63-541.06)	175	-0.84(-1.27,-0.41)	-0.81(-0.89,-0.72)	0.46(0.23,0.69)	-0.43(-0.58,-0.27)	127
Mali	370.62(305.49-462.29)	192	383.30(308.95-479.04)	184	-0.63(-0.75,-0.51)	0.17(0.00,0.34)	0.96(0.78,1.14)	0.16(0.07,0.26)	24
Mauritania	736.41(591.18-924.79)	60	679.73(541.58-858.93)	49	-0.40(-0.50,-0.31)	-0.64(-0.71,-0.57)	0.21(0.13,0.28)	-0.31(-0.36,-0.26)	102
Niger	552.89(447.71-682.14)	133	467.77(370.98-589.81)	153	-0.87(-0.95,-0.79)	-0.78(-0.81,-0.76)	0.04(-0.02,0.10)	-0.58(-0.61,-0.54)	151
Nigeria	709.77(570.17-885.26)	66	591.93(467.20-756.46)	82	-1.12(-1.73,-0.50)	-0.62(-0.71,-0.52)	-0.20(-0.80,0.41)	-0.64(-0.92,-0.37)	161
Sao Tome and Principe	719.37(589.67-874.30)	64	623.18(508.70-753.49)	68	0.27(0.14,0.40)	-1.39(-1.50,-1.28)	-0.37(-0.71,-0.03)	-0.54(-0.66,-0.42)	143
Senegal	421.09(343.40-522.90)	178	403.64(319.38-513.24)	179	-0.99(-1.03,-0.94)	-0.29(-0.36,-0.23)	1.01(0.86,1.16)	-0.13(-0.18,-0.07)	75

Sierra Leone	523.80(418.29-647.57)	149	472.48(374.62-595.83)	147	-0.95(-1.07,-0.84)	-0.47(-0.89,-0.06)	0.33(0.17,0.50)	-0.42(-0.57,-0.27)	126
Togo	611.68(493.18-755.81)	105	567.43(451.61-719.46)	94	-0.37(-0.50,-0.24)	-0.63(-0.74,-0.52)	0.32(0.22,0.43)	-0.26(-0.33,-0.18)	92
American Samoa	579.93(458.92-737.80)	120	568.90(421.19-728.73)	93	-2.27(-3.04,-1.50)	0.35(0.01,0.69)	2.13(1.21,3.07)	0.07(-0.36,0.50)	36
Bermuda	894.72(684.57-1158.18)	30	944.01(709.69-1216.77)	12	0.22(0.18,0.27)	0.22(0.18,0.27)	0.35(-0.30,1.00)	0.24(0.03,0.45)	14
Cook Islands	639.21(489.11-822.40)	94	598.48(449.36-767.93)	79	-1.94(-2.41,-1.46)	0.08(-0.07,0.24)	1.37(1.02,1.72)	-0.14(-0.34,0.07)	76
Greenland	1352.09(1083.75-1687.09)	2	1017.29(827.76-1276.14)	8	-0.22(-0.40,-0.03)	-2.01(-2.23,-1.79)	-0.72(-0.76,-0.67)	-1.01(-1.11,-0.91)	188
Guam	641.12(487.08-834.26)	93	570.24(425.28-746.41)	92	-1.11(-1.52,-0.70)	-0.78(-0.91,-0.64)	0.99(0.66,1.33)	-0.32(-0.51,-0.13)	103
Monaco	497.35(389.21-632.62)	156	486.73(373.13-614.41)	140	-0.29(-0.33,-0.26)	-0.07(-0.12,-0.01)	0.17(0.12,0.22)	-0.06(-0.09,-0.03)	63
Nauru	561.54(453.48-688.41)	127	548.55(421.32-712.77)	103	-1.06(-1.14,-0.98)	-1.06(-1.14,-0.98)	2.46(0.68,4.27)	0.02(-0.52,0.57)	46
Niue	586.61(457.59-755.67)	115	583.21(438.72-746.59)	87	-2.22(-2.40,-2.04)	0.23(0.19,0.28)	2.02(1.43,2.60)	0.01(-0.18,0.20)	49
Northern Mariana Islands	648.41(505.83-829.56)	89	594.53(451.47-780.45)	80	-1.84(-2.16,-1.52)	0.22(0.04,0.40)	1.06(0.67,1.45)	-0.18(-0.37,0.01)	84
Palau	637.71(507.42-784.60)	98	557.94(435.94-704.38)	98	-0.97(-1.39,-0.56)	-0.91(-1.05,-0.77)	0.73(0.45,1.02)	-0.40(-0.57,-0.22)	120
Puerto Rico	1302.91(1019.27-1618.45)	3	1207.00(942.25-1538.98)	2	0.15(0.06,0.23)	-0.46(-0.57,-0.34)	-0.39(-0.70,-0.07)	-0.23(-0.34,-0.12)	88
Saint Kitts and Nevis	924.44(712.93-1179.12)	24	927.17(704.46-1207.11)	13	-0.17(-0.18,-0.15)	-0.17(-0.18,-0.15)	0.43(0.20,0.66)	0.02(-0.05,0.09)	47
San Marino	482.99(379.61-607.39)	160	479.17(369.03-607.09)	145	-0.25(-0.27,-0.23)	-0.03(-0.04,-0.02)	0.23(0.13,0.33)	-0.02(-0.05,0.02)	55
Tokelau	565.33(421.44-742.41)	124	580.07(431.65-744.16)	88	-2.34(-2.85,-1.82)	0.85(0.70,1.01)	2.18(1.71,2.64)	0.23(-0.01,0.46)	17
Tuvalu	520.11(422.40-631.83)	151	573.64(425.97-734.65)	91	-1.00(-1.07,-0.93)	-1.00(-1.07,-0.93)	3.80(2.24,5.38)	0.46(-0.01,0.94)	6
United States Virgin Islands	824.35(642.07-1074.49)	48	879.43(672.99-1142.58)	23	-0.03(-0.07,0.01)	0.16(0.15,0.18)	0.59(0.44,0.75)	0.24(0.19,0.29)	15
South Sudan	747.36(604.04-920.61)	58	650.92(520.64-815.58)	58	-0.79(-0.84,-0.75)	-0.79(-0.84,-0.75)	0.33(0.03,0.62)	-0.43(-0.54,-0.33)	128
Sudan	647.33(518.05-795.29)	90	635.68(508.20-803.78)	65	-0.09(-0.12,-0.07)	-0.50(-0.56,-0.44)	0.51(0.40,0.63)	-0.06(-0.10,-0.01)	64
Inflammatory bowel disease									
China	1.47(1.24-1.74)	123	3.01(2.59-3.50)	81	3.26(2.95,3.58)	1.77(1.43,2.11)	2.15(2.08,2.22)	2.48(2.33,2.64)	4
Democratic People's Republic of Korea	0.96(0.80-1.15)	153	1.29(1.08-1.53)	138	1.47(0.71,2.24)	0.78(0.74,0.82)	0.78(0.74,0.82)	0.99(0.76,1.23)	53
Taiwan (Province of China)	0.71(0.62-0.81)	173	1.72(1.54-1.93)	125	0.81(0.22,1.42)	6.92(5.39,8.47)	2.34(2.04,2.64)	3.20(2.68,3.73)	1
Cambodia	0.37(0.30-0.45)	201	0.49(0.40-0.59)	203	0.77(0.69,0.86)	1.33(1.28,1.39)	0.61(0.49,0.73)	0.92(0.86,0.97)	61
Indonesia	0.43(0.36-0.52)	197	0.55(0.46-0.66)	198	1.58(1.35,1.82)	0.45(0.33,0.56)	0.68(0.64,0.73)	0.87(0.79,0.96)	70
Lao People's Democratic Republic	0.37(0.30-0.46)	200	0.51(0.42-0.63)	202	0.82(0.79,0.84)	0.88(0.79,0.98)	1.51(1.38,1.63)	1.06(1.00,1.11)	40
Malaysia	0.46(0.39-0.52)	193	0.76(0.67-0.85)	177	3.03(2.97,3.09)	1.05(0.99,1.11)	1.39(1.36,1.42)	1.77(1.74,1.80)	13
Maldives	0.34(0.28-0.41)	203	0.51(0.43-0.61)	201	2.16(2.08,2.25)	0.77(0.70,0.84)	1.34(1.13,1.55)	1.38(1.30,1.45)	22
Myanmar	0.40(0.32-0.49)	199	0.55(0.46-0.68)	197	0.65(0.62,0.68)	1.25(1.23,1.28)	1.54(1.48,1.60)	1.15(1.13,1.18)	34
Philippines	0.48(0.39-0.58)	188	0.63(0.52-0.76)	190	1.37(1.17,1.57)	-0.05(-0.16,0.05)	1.64(1.54,1.74)	0.97(0.89,1.05)	54
Sri Lanka	0.67(0.56-0.81)	174	1.15(1.02-1.30)	152	3.86(3.63,4.09)	0.83(0.78,0.89)	1.36(1.30,1.43)	1.93(1.85,2.00)	10
Thailand	0.33(0.26-0.41)	204	0.46(0.38-0.56)	204	1.81(1.63,1.99)	0.52(0.48,0.57)	1.32(1.28,1.36)	1.20(1.14,1.25)	30
Timor-Leste	0.36(0.29-0.45)	202	0.51(0.42-0.62)	200	0.33(0.28,0.38)	2.20(2.11,2.30)	1.18(1.07,1.28)	1.23(1.18,1.29)	28
Viet Nam	0.66(0.53-0.82)	175	1.37(1.11-1.70)	133	5.86(5.48,6.25)	1.10(0.78,1.41)	0.82(0.77,0.88)	2.49(2.30,2.68)	3
Fiji	0.54(0.44-0.64)	180	0.72(0.61-0.86)	182	0.58(0.56,0.60)	1.12(1.03,1.22)	1.20(1.05,1.35)	1.00(0.94,1.06)	50
Kiribati	0.48(0.40-0.58)	187	0.58(0.48-0.70)	195	0.20(0.16,0.23)	0.61(0.45,0.76)	0.92(0.72,1.12)	0.59(0.50,0.68)	110
Marshall Islands	0.46(0.38-0.55)	190	0.63(0.53-0.76)	189	0.52(0.49,0.55)	1.30(1.22,1.37)	1.30(1.23,1.37)	1.06(1.02,1.10)	41

Micronesia (Federated States of)	0.49(0.41-0.59)	183	0.67(0.56-0.81)	184	0.57(0.51,0.62)	1.37(1.27,1.47)	1.07(0.97,1.18)	1.02(0.97,1.08)	45
Papua New Guinea	0.40(0.33-0.48)	198	0.52(0.44-0.64)	199	0.40(0.33,0.47)	0.95(0.84,1.06)	1.27(1.18,1.35)	0.88(0.83,0.94)	67
Samoa	0.46(0.38-0.55)	192	0.60(0.51-0.73)	192	-0.60(-0.74,-0.45)	1.23(1.05,1.40)	1.96(1.50,2.42)	0.90(0.71,1.09)	63
Solomon Islands	0.43(0.36-0.52)	196	0.56(0.46-0.68)	196	0.60(0.57,0.64)	0.86(0.76,0.95)	0.98(0.94,1.02)	0.86(0.82,0.90)	72
Tonga	0.45(0.38-0.53)	195	0.66(0.55-0.78)	185	0.85(0.74,0.97)	1.46(1.33,1.59)	1.48(1.40,1.55)	1.32(1.25,1.39)	26
Vanuatu	0.45(0.37-0.55)	194	0.59(0.49-0.71)	194	0.45(0.41,0.49)	1.14(0.98,1.29)	1.10(1.01,1.19)	0.94(0.86,1.01)	57
Armenia	5.48(4.60-6.46)	53	6.64(5.62-7.95)	53	0.73(0.67,0.79)	0.92(0.90,0.95)	0.27(0.14,0.40)	0.66(0.62,0.71)	97
Azerbaijan	6.74(5.74-7.97)	41	7.01(5.92-8.40)	49	-0.02(-0.09,0.05)	0.34(0.30,0.38)	0.08(-0.06,0.23)	0.14(0.09,0.20)	168
Georgia	6.48(5.54-7.62)	42	6.32(5.38-7.44)	56	-0.19(-0.29,-0.10)	0.03(-0.20,0.26)	-0.16(-0.46,0.13)	-0.13(-0.25,0.00)	188
Kazakhstan	6.01(5.11-7.11)	47	7.20(6.14-8.56)	46	0.89(0.77,1.00)	-0.48(-0.59,-0.37)	1.48(1.01,1.96)	0.60(0.44,0.75)	108
Kyrgyzstan	6.03(5.14-7.11)	46	6.39(5.41-7.54)	55	0.47(0.38,0.55)	0.06(0.02,0.11)	-0.14(-0.20,-0.07)	0.13(0.08,0.18)	171
Mongolia	6.23(5.27-7.49)	44	7.35(6.20-8.69)	43	0.50(0.43,0.58)	0.73(0.37,1.09)	0.34(0.21,0.46)	0.52(0.40,0.65)	119
Tajikistan	5.83(4.91-6.93)	49	6.23(5.32-7.30)	57	-0.22(-0.69,0.26)	0.60(-0.22,1.44)	0.22(0.12,0.32)	0.16(-0.15,0.47)	165
Turkmenistan	6.75(5.73-7.99)	40	7.68(6.53-9.05)	42	-0.17(-0.21,-0.12)	1.18(0.97,1.39)	0.46(0.36,0.55)	0.45(0.38,0.52)	127
Uzbekistan	5.44(4.62-6.45)	54	6.90(5.90-8.20)	51	0.42(0.10,0.74)	1.06(0.63,1.49)	1.02(0.83,1.21)	0.87(0.69,1.06)	71
Albania	7.94(6.78-9.31)	35	9.48(8.09-11.13)	30	0.46(0.37,0.55)	1.37(1.32,1.42)	0.10(0.07,0.14)	0.60(0.57,0.64)	109
Bosnia and Herzegovina	7.18(6.30-8.17)	39	9.26(8.25-10.53)	33	0.96(0.81,1.11)	1.17(1.00,1.34)	0.38(0.33,0.43)	0.89(0.81,0.97)	65
Bulgaria	7.80(6.60-9.14)	37	10.34(8.83-12.16)	26	2.98(2.94,3.02)	0.09(0.00,0.18)	0.05(0.03,0.07)	0.97(0.92,1.02)	55
Croatia	10.11(9.48-10.76)	24	13.09(12.06-14.21)	22	0.83(0.59,1.07)	0.81(0.76,0.87)	1.01(0.76,1.27)	0.90(0.79,1.02)	64
Czechia	4.86(4.13-5.83)	57	9.26(8.60-9.97)	34	2.50(2.01,2.99)	1.14(1.06,1.23)	3.22(2.39,4.07)	2.21(1.90,2.51)	5
Hungary	11.99(11.06-12.81)	16	21.55(20.45-22.74)	10	3.26(2.87,3.66)	2.31(2.14,2.49)	0.35(0.18,0.52)	2.04(1.88,2.19)	7
North Macedonia	9.30(7.93-10.90)	28	9.96(8.49-11.66)	27	0.17(0.14,0.20)	0.70(0.68,0.72)	-0.28(-0.34,-0.21)	0.23(0.21,0.26)	156
Montenegro	9.07(7.74-10.58)	29	9.46(8.02-11.15)	31	-0.68(-0.82,-0.55)	0.80(0.70,0.89)	0.41(0.37,0.46)	0.17(0.11,0.22)	163
Poland	33.98(28.90-39.68)	3	13.28(11.82-14.95)	21	-5.42(-5.66,-5.18)	-2.76(-2.99,-2.52)	-0.54(-0.58,-0.50)	-3.26(-3.38,-3.14)	204
Romania	5.24(4.44-6.18)	55	5.63(5.11-6.27)	61	-1.96(-2.14,-1.77)	0.95(0.90,1.00)	1.61(1.41,1.82)	0.24(0.15,0.33)	152
Serbia	8.89(7.60-10.37)	30	12.68(10.96-14.68)	23	3.25(2.84,3.66)	0.28(0.24,0.33)	0.19(0.15,0.22)	1.19(1.06,1.32)	31
Slovakia	10.35(9.18-11.58)	23	9.70(8.69-10.81)	28	0.09(-0.09,0.27)	-0.22(-0.29,-0.14)	-0.58(-0.88,-0.28)	-0.24(-0.36,-0.12)	192
Slovenia	10.77(9.39-12.53)	21	12.39(10.96-14.10)	24	1.03(0.99,1.06)	0.46(0.44,0.48)	-0.03(-0.05,-0.01)	0.49(0.47,0.50)	125
Belarus	4.51(3.86-5.30)	58	5.71(4.86-6.75)	60	1.10(0.97,1.22)	0.34(0.26,0.42)	1.04(0.70,1.38)	0.79(0.67,0.91)	80
Estonia	3.68(3.26-4.16)	65	6.82(6.04-7.64)	52	3.31(3.13,3.50)	1.17(1.12,1.22)	2.14(1.69,2.59)	2.13(1.98,2.28)	6
Latvia	5.56(4.79-6.41)	51	7.15(6.25-8.09)	47	2.64(2.45,2.83)	0.09(-0.03,0.21)	-0.03(-0.17,0.11)	0.85(0.75,0.94)	73
Lithuania	5.92(5.20-6.73)	48	8.26(7.85-8.65)	38	1.54(1.46,1.62)	0.99(0.98,1.01)	0.90(0.84,0.97)	1.18(1.14,1.21)	33
Republic of Moldova	4.41(3.71-5.26)	59	4.51(4.16-4.86)	65	-1.62(-1.71,-1.54)	0.94(0.93,0.95)	0.94(0.93,0.95)	0.09(0.06,0.12)	174
Russian Federation	7.96(6.86-9.34)	33	7.86(6.75-9.25)	41	0.54(0.34,0.74)	-0.30(-0.49,-0.11)	-0.50(-0.55,-0.45)	-0.06(-0.16,0.03)	185
Ukraine	5.64(4.78-6.68)	50	6.22(5.33-7.29)	58	0.67(0.60,0.74)	0.28(0.23,0.33)	0.12(-0.03,0.27)	0.35(0.30,0.41)	139
Brunei Darussalam	4.95(4.11-5.97)	56	5.09(4.17-6.09)	64	-0.03(-0.05,-0.01)	0.09(0.02,0.15)	0.22(0.20,0.24)	0.08(0.06,0.11)	175
Japan	11.22(9.49-13.32)	18	19.65(16.87-22.71)	12	4.87(4.12,5.63)	0.54(0.27,0.82)	0.93(0.81,1.05)	1.98(1.72,2.25)	8
Republic of Korea	3.37(2.96-3.78)	70	7.33(7.03-7.63)	44	6.21(5.62,6.82)	1.16(1.14,1.18)	1.16(1.14,1.18)	2.73(2.55,2.91)	2

Singapore	3.47(2.87-4.13)	68	3.21(2.68-3.90)	76	-1.09(-1.47,-0.72)	0.40(0.20,0.60)	0.01(-0.23,0.24)	-0.30(-0.48,-0.13)	193
Australia	12.09(10.32-14.31)	15	19.62(17.36-22.17)	13	4.41(4.09,4.74)	0.50(0.49,0.52)	0.50(0.49,0.52)	1.70(1.60,1.80)	16
New Zealand	21.00(18.17-24.03)	7	22.45(19.83-25.48)	8	0.26(0.21,0.31)	0.24(0.23,0.26)	0.17(0.10,0.23)	0.23(0.20,0.26)	157
Andorra	8.55(7.26-10.05)	31	9.18(7.75-10.83)	35	1.06(0.94,1.18)	-0.23(-0.26,-0.19)	-0.13(-0.22,-0.05)	0.26(0.21,0.31)	149
Austria	19.15(17.10-21.06)	10	20.81(18.80-23.22)	11	0.92(0.57,1.28)	1.10(0.35,1.86)	-0.88(-1.11,-0.65)	0.34(0.07,0.61)	140
Belgium	7.69(7.28-8.13)	38	13.40(11.65-15.72)	20	1.06(1.01,1.10)	0.48(0.46,0.50)	4.67(4.46,4.89)	1.94(1.88,2.01)	9
Cyprus	3.97(3.44-4.65)	63	6.07(5.48-6.74)	59	3.45(3.30,3.60)	0.78(0.63,0.94)	0.12(0.01,0.22)	1.41(1.32,1.49)	20
Denmark	24.36(22.66-26.11)	5	21.82(20.43-23.28)	9	-1.16(-1.31,-1.01)	0.35(0.30,0.40)	-0.36(-0.64,-0.08)	-0.34(-0.44,-0.24)	195
Finland	14.91(14.34-15.46)	13	24.74(22.08-27.29)	4	0.65(0.59,0.71)	1.43(1.25,1.60)	3.32(3.05,3.60)	1.76(1.65,1.87)	14
France	11.23(10.86-11.58)	17	13.45(12.64-14.08)	19	0.53(0.51,0.55)	0.53(0.51,0.55)	0.82(0.37,1.26)	0.62(0.48,0.76)	106
Germany	10.77(9.41-12.38)	22	18.37(16.07-20.92)	14	4.17(3.75,4.60)	0.57(0.46,0.69)	1.01(0.63,1.39)	1.84(1.65,2.04)	11
Greece	7.88(6.77-9.03)	36	7.95(7.36-8.56)	40	-0.09(-0.19,0.01)	-0.30(-0.59,-0.01)	0.57(0.48,0.66)	0.00(-0.11,0.10)	179
Iceland	11.18(9.58-13.00)	19	17.01(14.81-19.23)	16	4.12(3.87,4.38)	0.37(0.33,0.41)	0.02(-0.01,0.06)	1.41(1.33,1.49)	21
Ireland	9.71(8.23-11.39)	26	8.17(7.14-9.44)	39	-0.57(-0.87,-0.28)	-1.89(-2.19,-1.60)	0.52(0.45,0.59)	-0.59(-0.73,-0.45)	199
Israel	6.25(5.41-7.22)	43	7.07(6.48-7.84)	48	1.64(1.41,1.86)	-1.05(-1.19,-0.90)	0.45(0.38,0.52)	0.44(0.35,0.53)	128
Italy	18.93(16.56-21.54)	11	18.16(16.07-20.44)	15	-0.95(-1.19,-0.71)	1.19(0.94,1.45)	-0.72(-0.90,-0.55)	-0.06(-0.20,0.07)	186
Luxembourg	18.82(16.34-21.72)	12	15.55(13.60-17.92)	17	-1.38(-1.61,-1.15)	-0.17(-0.22,-0.12)	-0.33(-0.60,-0.05)	-0.63(-0.75,-0.52)	200
Malta	7.94(6.81-9.40)	34	8.92(7.75-10.47)	37	1.09(0.84,1.35)	0.08(0.04,0.12)	0.08(0.04,0.12)	0.42(0.33,0.50)	132
Netherlands	13.19(12.55-13.87)	14	7.21(6.24-8.42)	45	0.62(0.59,0.65)	-0.52(-0.63,-0.41)	-6.52(-7.07,-5.98)	-2.12(-2.30,-1.93)	203
Norway	28.71(24.47-33.64)	4	36.64(31.66-42.06)	2	1.22(1.05,1.40)	1.27(1.13,1.41)	-0.08(-0.19,0.03)	0.83(0.74,0.91)	77
Portugal	5.49(4.82-6.34)	52	9.37(8.55-10.26)	32	4.36(4.09,4.64)	0.79(0.75,0.84)	0.38(0.20,0.57)	1.83(1.72,1.93)	12
Spain	10.01(9.64-10.37)	25	14.01(13.46-14.57)	18	1.83(1.72,1.94)	0.72(0.68,0.76)	1.03(0.88,1.19)	1.14(1.08,1.21)	35
Sweden	23.51(20.53-26.98)	6	27.26(23.97-31.28)	3	0.60(0.54,0.66)	0.80(0.54,1.05)	0.16(-0.24,0.56)	0.54(0.38,0.71)	114
Switzerland	10.95(9.68-12.50)	20	11.45(10.20-12.96)	25	1.12(1.01,1.23)	-0.19(-0.25,-0.13)	-0.37(-0.44,-0.30)	0.16(0.11,0.21)	166
United Kingdom	19.93(17.45-22.89)	8	23.11(20.33-26.28)	7	0.94(0.80,1.07)	0.55(0.51,0.59)	0.01(-0.04,0.06)	0.50(0.46,0.55)	122
Argentina	1.63(1.36-1.94)	121	1.73(1.44-2.03)	124	0.39(0.30,0.47)	-0.05(-0.08,-0.01)	0.25(0.23,0.28)	0.19(0.16,0.22)	159
Chile	2.37(2.03-2.73)	85	2.44(2.10-2.81)	104	1.61(1.40,1.81)	0.68(0.63,0.73)	-1.87(-2.13,-1.61)	0.13(0.01,0.24)	172
Uruguay	1.30(1.09-1.56)	127	1.76(1.47-2.09)	123	1.79(1.61,1.98)	0.62(0.57,0.66)	0.79(0.36,1.21)	1.03(0.89,1.18)	44
Canada	37.90(36.81-38.96)	1	36.97(35.65-38.27)	1	0.26(0.16,0.35)	0.89(0.86,0.93)	-1.54(-1.77,-1.30)	-0.06(-0.15,0.02)	187
United States of America	34.58(29.79-40.26)	2	23.19(21.18-25.62)	6	-5.26(-5.84,-4.68)	0.60(0.46,0.74)	0.17(0.07,0.27)	-1.40(-1.60,-1.20)	202
Antigua and Barbuda	2.05(1.74-2.46)	107	2.60(2.21-3.13)	98	1.55(1.21,1.89)	-0.56(-0.64,-0.48)	1.76(1.67,1.86)	0.81(0.70,0.92)	78
Bahamas	2.21(1.88-2.62)	100	3.25(2.71-3.92)	74	1.64(1.51,1.76)	0.22(-0.12,0.56)	2.30(2.14,2.45)	1.38(1.24,1.52)	23
Barbados	4.26(3.60-5.10)	61	5.13(4.39-6.10)	63	1.64(1.53,1.74)	-1.00(-1.28,-0.71)	1.45(1.32,1.58)	0.68(0.56,0.80)	95
Belize	2.09(1.77-2.52)	106	2.70(2.26-3.27)	94	1.69(1.53,1.85)	-0.30(-0.36,-0.24)	1.55(1.48,1.62)	0.89(0.83,0.94)	66
Cuba	2.11(1.79-2.55)	104	2.53(2.12-3.02)	101	1.29(1.12,1.47)	-1.06(-1.15,-0.97)	1.76(1.67,1.85)	0.64(0.57,0.72)	100
Dominica	2.05(1.73-2.44)	108	3.02(2.54-3.62)	80	3.03(2.93,3.12)	0.33(0.13,0.54)	0.94(0.84,1.04)	1.36(1.27,1.46)	24
Dominican Republic	2.01(1.68-2.40)	109	2.68(2.25-3.25)	95	1.65(1.40,1.89)	-0.08(-0.15,-0.01)	1.67(1.58,1.76)	1.00(0.91,1.08)	51
Grenada	2.24(1.86-2.68)	96	2.98(2.48-3.61)	82	1.96(1.88,2.04)	-0.35(-0.56,-0.13)	1.56(1.47,1.66)	1.02(0.94,1.10)	46

Guyana	2.28(1.91-2.74)	91	2.87(2.41-3.44)	88	1.61(1.45,1.78)	-0.97(-1.06,-0.89)	1.81(1.73,1.90)	0.79(0.72,0.86)	81
Haiti	1.95(1.63-2.34)	111	2.42(2.03-2.97)	105	1.56(1.41,1.71)	-0.81(-0.88,-0.74)	1.59(1.51,1.66)	0.76(0.70,0.82)	86
Jamaica	1.90(1.60-2.26)	113	2.50(2.11-2.99)	102	1.22(0.84,1.60)	-0.31(-0.53,-0.10)	2.30(2.06,2.54)	1.02(0.84,1.19)	47
Saint Lucia	2.25(1.88-2.70)	94	2.64(2.22-3.19)	97	1.12(0.83,1.41)	-0.99(-1.15,-0.84)	1.67(1.51,1.84)	0.56(0.44,0.69)	112
Saint Vincent and the Grenadines	2.11(1.78-2.54)	105	2.80(2.35-3.35)	89	2.60(2.49,2.72)	-0.96(-1.02,-0.90)	1.34(1.28,1.40)	0.95(0.90,1.00)	56
Suriname	2.22(1.86-2.66)	99	2.76(2.31-3.34)	91	1.31(1.21,1.42)	-0.65(-0.71,-0.60)	1.68(1.62,1.74)	0.76(0.72,0.80)	87
Trinidad and Tobago	2.51(2.10-3.01)	80	3.19(2.68-3.86)	77	2.28(2.23,2.34)	-0.92(-1.02,-0.82)	1.47(1.42,1.52)	0.80(0.75,0.84)	79
Bolivia (Plurinational State of)	1.71(1.48-2.04)	116	1.85(1.58-2.23)	121	1.17(1.07,1.27)	-1.70(-1.86,-1.54)	1.33(1.04,1.62)	0.29(0.16,0.41)	144
Ecuador	1.55(1.37-1.77)	122	1.89(1.67-2.13)	119	3.12(2.94,3.31)	-2.58(-2.77,-2.38)	1.38(1.17,1.58)	0.59(0.48,0.69)	111
Peru	1.65(1.39-1.96)	119	1.78(1.50-2.11)	122	1.05(0.80,1.30)	-1.91(-2.03,-1.78)	1.78(1.65,1.91)	0.30(0.20,0.41)	142
Colombia	2.21(1.88-2.59)	101	2.20(1.86-2.59)	113	-0.21(-0.51,0.09)	-1.42(-1.63,-1.21)	1.49(1.34,1.64)	0.02(-0.11,0.15)	178
Costa Rica	2.55(2.16-2.99)	78	2.32(1.99-2.73)	110	-0.31(-0.44,-0.19)	-1.53(-1.62,-1.45)	0.74(0.67,0.80)	-0.31(-0.37,-0.25)	194
El Salvador	2.53(2.15-2.99)	79	2.41(2.06-2.81)	106	0.26(0.07,0.46)	-1.23(-1.31,-1.14)	0.44(0.34,0.54)	-0.14(-0.22,-0.06)	189
Guatemala	2.33(1.97-2.76)	89	2.33(1.99-2.74)	109	0.27(0.12,0.42)	-1.37(-1.46,-1.28)	0.89(0.82,0.95)	0.00(-0.07,0.06)	180
Honduras	2.29(1.95-2.69)	90	2.16(1.83-2.55)	117	0.12(-0.06,0.29)	-1.73(-1.84,-1.62)	0.77(0.69,0.85)	-0.19(-0.27,-0.12)	190
Mexico	3.99(3.46-4.63)	62	3.06(2.67-3.52)	79	-1.83(-2.06,-1.59)	-1.58(-1.74,-1.42)	0.69(0.57,0.80)	-0.86(-0.97,-0.76)	201
Nicaragua	2.38(2.02-2.84)	83	2.06(1.77-2.43)	118	-0.55(-0.93,-0.18)	-0.87(-1.21,-0.53)	-0.41(-0.51,-0.32)	-0.55(-0.72,-0.38)	198
Panama	2.45(2.13-2.82)	81	2.20(1.88-2.56)	114	-0.40(-0.91,0.12)	-2.16(-2.49,-1.82)	1.12(0.86,1.38)	-0.40(-0.62,-0.18)	196
Venezuela (Bolivarian Republic of)	2.27(1.98-2.57)	93	2.18(1.85-2.55)	115	0.27(0.13,0.41)	-1.96(-2.24,-1.68)	1.05(0.58,1.52)	-0.19(-0.37,-0.01)	191
Brazil	6.15(5.46-7.00)	45	5.30(4.65-6.09)	62	-0.46(-0.62,-0.30)	-0.70(-1.44,0.05)	-0.38(-0.48,-0.28)	-0.51(-0.75,-0.26)	197
Paraguay	3.48(3.05-4.01)	67	3.38(2.90-3.94)	73	3.08(2.85,3.30)	-3.04(-3.79,-2.28)	-0.39(-0.64,-0.14)	-0.05(-0.31,0.22)	184
Algeria	2.87(2.55-3.23)	73	3.87(3.23-4.74)	68	1.77(1.74,1.80)	0.65(0.50,0.81)	0.59(0.55,0.64)	1.02(0.97,1.07)	48
Bahrain	2.64(2.30-3.00)	76	3.21(2.68-3.88)	75	0.98(0.71,1.25)	0.70(-0.28,1.70)	0.40(0.15,0.65)	0.63(0.30,0.96)	104
Egypt	3.48(3.05-3.91)	66	4.23(3.51-5.16)	66	1.48(1.40,1.55)	-0.79(-0.98,-0.60)	1.16(0.95,1.36)	0.64(0.55,0.74)	101
Iran (Islamic Republic of)	3.30(2.74-4.05)	71	3.43(2.86-4.23)	72	0.86(0.59,1.12)	-0.32(-0.37,-0.28)	-0.32(-0.37,-0.28)	0.14(0.04,0.23)	169
Iraq	1.82(1.55-2.12)	114	2.54(2.12-3.08)	100	0.38(0.19,0.56)	1.78(1.58,1.99)	1.07(0.92,1.21)	1.13(1.02,1.24)	36
Jordan	4.40(3.86-5.00)	60	6.91(5.78-8.15)	50	2.89(2.48,3.30)	0.96(0.91,1.02)	0.96(0.91,1.02)	1.56(1.43,1.69)	18
Kuwait	3.43(3.19-3.71)	69	3.72(3.09-4.54)	69	0.66(0.36,0.96)	0.58(-0.52,1.69)	-0.52(-1.34,0.30)	0.32(-0.13,0.78)	141
Lebanon	2.34(1.93-2.90)	87	3.56(2.97-4.37)	70	1.93(1.77,2.09)	1.75(1.63,1.86)	0.74(0.68,0.79)	1.43(1.36,1.50)	19
Libya	1.64(1.37-1.99)	120	2.41(2.01-2.96)	107	2.83(2.62,3.03)	-0.84(-1.25,-0.42)	2.22(1.37,3.08)	1.30(0.99,1.61)	27
Morocco	1.43(1.21-1.69)	124	1.61(1.36-1.91)	126	0.37(0.31,0.43)	0.07(0.03,0.12)	0.75(0.73,0.77)	0.40(0.38,0.43)	134
Palestine	2.00(1.67-2.43)	110	2.94(2.47-3.50)	83	3.08(2.96,3.19)	-0.13(-0.38,0.11)	0.95(0.79,1.12)	1.36(1.25,1.46)	25
Oman	2.43(2.02-2.90)	82	2.73(2.27-3.27)	93	1.98(1.61,2.36)	-0.74(-1.15,-0.32)	-0.08(-0.16,0.01)	0.39(0.20,0.57)	136
Qatar	2.85(2.38-3.43)	74	3.55(3.00-4.28)	71	0.81(0.74,0.88)	0.95(0.92,0.98)	0.50(0.41,0.58)	0.78(0.74,0.82)	83
Saudi Arabia	2.64(2.24-3.13)	77	2.66(2.24-3.18)	96	1.05(0.90,1.20)	-1.31(-1.48,-1.14)	0.26(-0.21,0.74)	-0.01(-0.18,0.15)	181
Syrian Arab Republic	2.70(2.24-3.26)	75	3.16(2.63-3.83)	78	0.45(0.35,0.55)	-0.82(-0.94,-0.70)	2.17(1.77,2.57)	0.53(0.40,0.67)	117
Tunisia	2.36(1.97-2.86)	86	2.73(2.25-3.32)	92	0.52(0.44,0.60)	0.81(0.14,1.48)	0.25(0.09,0.41)	0.52(0.30,0.73)	120
Turkey	3.87(3.46-4.29)	64	6.44(5.55-7.42)	54	2.88(2.65,3.10)	0.04(-0.26,0.34)	2.65(2.33,2.97)	1.73(1.56,1.91)	15

United Arab Emirates	3.21(2.70-3.87)	72	4.12(3.40-5.01)	67	1.99(1.92,2.06)	0.59(0.50,0.69)	0.05(-0.13,0.22)	0.85(0.78,0.92)	74
Yemen	2.12(1.75-2.59)	103	2.54(2.10-3.09)	99	0.69(0.66,0.71)	0.91(0.84,0.99)	0.24(0.10,0.38)	0.64(0.58,0.69)	102
Afghanistan	2.27(1.87-2.72)	92	2.45(2.03-2.96)	103	-0.35(-0.38,-0.32)	0.31(0.13,0.49)	0.73(0.65,0.80)	0.24(0.17,0.30)	153
Bangladesh	1.76(1.45-2.17)	115	2.17(1.78-2.65)	116	1.39(1.29,1.49)	0.33(0.11,0.56)	0.30(0.15,0.45)	0.71(0.61,0.81)	93
Bhutan	1.68(1.38-2.11)	117	1.86(1.55-2.29)	120	1.29(1.13,1.45)	-1.03(-1.58,-0.47)	1.28(1.07,1.49)	0.43(0.22,0.63)	129
India	2.23(1.85-2.73)	98	2.34(1.95-2.86)	108	0.81(0.77,0.85)	0.81(0.77,0.85)	-1.12(-1.79,-0.45)	0.21(0.00,0.42)	158
Nepal	1.66(1.37-2.07)	118	2.24(1.85-2.76)	112	1.32(1.23,1.41)	0.83(0.80,0.85)	0.97(0.89,1.05)	1.05(1.01,1.10)	42
Pakistan	1.91(1.59-2.34)	112	2.30(1.91-2.86)	111	0.78(0.69,0.87)	0.38(0.29,0.47)	0.71(0.68,0.74)	0.66(0.62,0.71)	98
Angola	1.09(0.91-1.34)	137	1.32(1.12-1.59)	136	0.68(0.65,0.70)	0.61(0.56,0.65)	0.69(0.65,0.73)	0.67(0.65,0.70)	96
Central African Republic	1.10(0.92-1.37)	136	1.13(0.94-1.35)	155	-0.04(-0.13,0.05)	0.12(0.10,0.15)	0.14(0.05,0.22)	0.08(0.04,0.12)	176
Congo	1.10(0.92-1.33)	135	1.41(1.18-1.68)	131	1.80(1.44,2.15)	-0.12(-0.54,0.31)	0.93(0.84,1.01)	0.93(0.74,1.11)	59
Democratic Republic of the Congo	1.01(0.85-1.23)	145	1.22(1.03-1.49)	142	0.70(0.65,0.75)	0.14(-0.02,0.30)	1.40(1.12,1.68)	0.70(0.59,0.81)	94
Equatorial Guinea	0.99(0.83-1.21)	149	1.60(1.36-1.92)	127	0.78(0.64,0.92)	2.84(2.49,3.20)	1.24(0.99,1.50)	1.61(1.45,1.76)	17
Gabon	1.21(1.02-1.47)	131	1.56(1.30-1.88)	128	1.06(1.02,1.09)	0.61(0.58,0.64)	0.95(0.89,1.01)	0.88(0.85,0.90)	68
Burundi	0.93(0.78-1.11)	160	0.97(0.81-1.18)	173	0.04(0.01,0.06)	0.62(0.59,0.65)	-0.25(-0.33,-0.17)	0.15(0.12,0.18)	167
Comoros	0.97(0.82-1.16)	151	1.13(0.94-1.37)	153	0.78(0.76,0.81)	0.01(-0.05,0.07)	0.87(0.74,0.99)	0.52(0.47,0.57)	121
Djibouti	0.96(0.81-1.15)	157	1.01(0.85-1.22)	168	0.94(0.83,1.06)	-0.07(-0.12,-0.02)	-0.25(-0.37,-0.13)	0.17(0.11,0.23)	164
Eritrea	0.90(0.77-1.09)	164	1.09(0.90-1.31)	160	0.99(0.95,1.03)	0.51(0.45,0.57)	0.42(0.39,0.46)	0.65(0.62,0.67)	99
Ethiopia	0.83(0.70-1.00)	171	0.99(0.84-1.20)	170	0.21(0.11,0.32)	0.82(0.68,0.96)	0.86(0.81,0.91)	0.62(0.56,0.68)	107
Kenya	0.87(0.74-1.05)	168	1.16(0.99-1.39)	150	0.12(0.07,0.18)	0.65(0.42,0.88)	1.97(1.62,2.32)	0.91(0.77,1.06)	62
Madagascar	0.99(0.84-1.21)	148	1.06(0.90-1.29)	162	0.19(0.12,0.25)	0.48(0.32,0.64)	0.08(0.04,0.12)	0.24(0.19,0.30)	154
Malawi	0.95(0.80-1.17)	158	1.10(0.92-1.34)	159	0.12(0.04,0.20)	0.63(0.48,0.78)	0.74(0.69,0.80)	0.50(0.44,0.55)	123
Mauritius	0.46(0.38-0.55)	191	0.59(0.50-0.71)	193	0.89(0.80,0.97)	0.67(0.65,0.69)	1.09(1.05,1.13)	0.88(0.85,0.91)	69
Mozambique	0.83(0.70-1.02)	170	1.00(0.85-1.19)	169	0.55(0.45,0.65)	0.83(0.81,0.86)	0.49(0.40,0.59)	0.64(0.59,0.69)	103
Rwanda	0.96(0.81-1.16)	154	1.04(0.87-1.25)	166	0.09(-0.02,0.19)	1.10(1.00,1.19)	-0.29(-0.49,-0.08)	0.29(0.20,0.37)	145
Seychelles	0.49(0.40-0.59)	185	0.61(0.51-0.73)	191	1.07(1.01,1.13)	0.24(0.17,0.31)	1.08(1.01,1.15)	0.79(0.75,0.82)	82
Somalia	0.77(0.65-0.94)	172	0.76(0.63-0.94)	176	-0.08(-0.18,0.03)	0.23(0.02,0.43)	-0.26(-0.31,-0.20)	-0.04(-0.12,0.03)	183
United Republic of Tanzania	0.91(0.76-1.11)	161	1.05(0.88-1.28)	165	-0.17(-0.20,-0.14)	0.53(0.34,0.71)	1.00(0.91,1.08)	0.47(0.41,0.54)	126
Uganda	0.90(0.76-1.10)	167	1.07(0.90-1.30)	161	0.30(0.26,0.35)	0.69(0.51,0.87)	0.84(0.74,0.94)	0.63(0.55,0.71)	105
Zambia	1.07(0.91-1.29)	139	1.11(0.95-1.35)	158	0.16(0.00,0.31)	0.86(0.68,1.04)	-0.84(-0.96,-0.73)	0.11(0.02,0.21)	173
Botswana	1.32(1.11-1.63)	125	1.47(1.22-1.79)	130	-0.28(-0.55,0.00)	0.81(0.65,0.97)	0.57(0.36,0.78)	0.41(0.27,0.55)	133
Lesotho	1.18(0.99-1.43)	132	1.36(1.14-1.65)	135	0.49(0.44,0.55)	0.40(0.20,0.59)	0.64(0.60,0.69)	0.50(0.44,0.57)	124
Namibia	1.28(1.08-1.55)	128	1.37(1.16-1.68)	134	0.65(0.55,0.74)	0.31(0.26,0.35)	-0.24(-0.33,-0.16)	0.26(0.21,0.30)	150
South Africa	1.32(1.12-1.59)	126	1.37(1.17-1.64)	132	0.27(0.03,0.51)	-0.36(-0.58,-0.13)	0.55(0.48,0.61)	0.19(0.07,0.30)	160
Eswatini	1.28(1.07-1.55)	129	1.49(1.26-1.81)	129	0.66(0.60,0.71)	0.29(0.22,0.36)	0.72(0.69,0.74)	0.54(0.50,0.57)	115
Zimbabwe	1.23(1.04-1.49)	130	1.30(1.09-1.59)	137	0.85(0.60,1.11)	-0.98(-1.10,-0.87)	0.77(0.67,0.87)	0.19(0.10,0.28)	161
Benin	1.05(0.88-1.27)	140	1.16(0.98-1.39)	149	-0.11(-0.22,-0.01)	0.09(0.04,0.13)	1.18(0.86,1.51)	0.36(0.26,0.47)	138
Burkina Faso	0.97(0.82-1.17)	152	1.05(0.89-1.27)	164	-0.08(-0.20,0.05)	0.02(-0.04,0.07)	0.87(0.73,1.01)	0.27(0.21,0.34)	148

Cameroon	1.16(0.97-1.42)	133	1.28(1.08-1.56)	139	0.52(0.29,0.75)	-0.25(-0.43,-0.07)	0.59(0.26,0.92)	0.28(0.12,0.44)	146
Cabo Verde	0.96(0.81-1.16)	155	1.19(1.00-1.44)	146	0.59(0.55,0.62)	0.30(0.25,0.35)	1.43(1.23,1.64)	0.74(0.68,0.81)	90
Chad	0.90(0.75-1.09)	166	0.99(0.83-1.20)	172	0.00(-0.11,0.11)	-0.39(-0.42,-0.35)	1.46(1.29,1.64)	0.30(0.24,0.37)	143
C??te d'Ivoire	1.07(0.90-1.32)	138	1.26(1.05-1.55)	140	0.96(0.81,1.12)	-0.35(-0.43,-0.28)	1.18(0.65,1.71)	0.53(0.36,0.70)	118
Gambia	0.91(0.76-1.09)	163	1.22(1.03-1.49)	143	0.41(0.31,0.51)	1.31(1.24,1.38)	1.26(0.87,1.65)	1.01(0.89,1.14)	49
Ghana	1.00(0.84-1.20)	147	1.20(1.01-1.46)	145	0.79(0.56,1.01)	0.01(-0.18,0.20)	0.90(0.63,1.18)	0.56(0.41,0.71)	113
Guinea	0.97(0.81-1.19)	150	1.06(0.89-1.27)	163	0.20(0.17,0.24)	-0.41(-0.49,-0.34)	1.18(1.03,1.34)	0.28(0.23,0.34)	147
Guinea-Bissau	1.00(0.84-1.23)	146	1.13(0.94-1.38)	154	0.22(0.17,0.27)	-0.14(-0.20,-0.09)	1.21(0.98,1.43)	0.40(0.32,0.47)	135
Liberia	1.05(0.88-1.27)	141	1.18(1.00-1.42)	148	-0.28(-0.36,-0.19)	-0.04(-0.19,0.10)	1.49(1.34,1.63)	0.43(0.36,0.50)	130
Mali	0.91(0.77-1.12)	162	1.02(0.87-1.24)	167	0.70(0.65,0.74)	-0.75(-0.80,-0.70)	1.34(1.22,1.45)	0.39(0.35,0.44)	137
Mauritania	1.11(0.93-1.35)	134	1.12(0.94-1.36)	157	-1.49(-1.65,-1.34)	0.87(0.65,1.09)	0.74(0.39,1.08)	-0.02(-0.16,0.12)	182
Niger	0.86(0.72-1.03)	169	0.90(0.76-1.09)	174	0.14(0.08,0.21)	-0.73(-0.82,-0.65)	1.11(0.90,1.33)	0.14(0.06,0.21)	170
Nigeria	1.02(0.87-1.23)	144	1.20(1.02-1.45)	144	0.05(-0.02,0.13)	-0.28(-0.59,0.03)	2.08(1.66,2.50)	0.54(0.35,0.72)	116
Sao Tome and Principe	0.90(0.76-1.09)	165	1.15(0.96-1.41)	151	0.52(0.42,0.62)	-0.40(-0.53,-0.28)	2.61(2.32,2.90)	0.84(0.74,0.94)	75
Senegal	1.04(0.87-1.25)	142	1.18(1.00-1.47)	147	0.03(0.00,0.07)	-0.21(-0.36,-0.05)	1.66(1.45,1.87)	0.43(0.34,0.53)	131
Sierra Leone	1.03(0.87-1.27)	143	1.12(0.95-1.37)	156	-0.22(-0.26,-0.18)	-0.53(-0.67,-0.38)	1.74(1.53,1.94)	0.26(0.17,0.34)	151
Togo	0.96(0.81-1.17)	156	1.23(1.04-1.48)	141	1.64(1.39,1.89)	-0.26(-0.37,-0.14)	1.39(0.86,1.93)	0.84(0.65,1.03)	76
American Samoa	0.49(0.41-0.58)	184	0.64(0.54-0.77)	187	0.53(0.49,0.57)	1.15(1.10,1.20)	0.99(0.85,1.13)	0.93(0.87,0.99)	60
Bermuda	2.34(1.97-2.86)	88	2.91(2.46-3.47)	87	1.52(1.27,1.76)	-0.79(-0.94,-0.64)	1.38(1.28,1.49)	0.77(0.67,0.88)	84
Cook Islands	0.54(0.45-0.64)	179	0.74(0.63-0.90)	180	0.54(0.51,0.57)	1.35(1.23,1.46)	1.34(1.27,1.41)	1.12(1.07,1.17)	37
Greenland	19.43(16.54-23.03)	9	24.18(20.79-27.92)	5	1.41(1.22,1.61)	0.87(0.82,0.92)	0.03(-0.16,0.22)	0.75(0.66,0.84)	89
Guam	0.55(0.46-0.66)	178	0.75(0.64-0.90)	178	0.39(0.27,0.51)	1.40(1.31,1.48)	1.25(1.15,1.35)	1.04(0.98,1.11)	43
Monaco	9.42(8.02-11.14)	27	9.53(8.11-11.17)	29	0.38(0.25,0.52)	-0.08(-0.14,-0.02)	-0.16(-0.18,-0.15)	0.04(-0.01,0.09)	177
Nauru	0.47(0.39-0.57)	189	0.64(0.54-0.77)	188	1.80(0.82,2.80)	0.98(0.80,1.17)	0.22(-0.25,0.68)	1.00(0.65,1.35)	52
Niue	0.51(0.43-0.62)	182	0.73(0.61-0.87)	181	0.64(0.59,0.70)	1.75(1.69,1.81)	1.15(1.11,1.19)	1.23(1.19,1.26)	29
Northern Mariana Islands	0.60(0.50-0.71)	176	0.74(0.63-0.89)	179	0.21(0.20,0.23)	1.01(0.95,1.06)	1.00(0.90,1.10)	0.76(0.73,0.80)	88
Palau	0.56(0.47-0.66)	177	0.76(0.64-0.91)	175	0.66(0.63,0.70)	1.23(1.07,1.38)	1.30(1.06,1.53)	1.09(0.99,1.19)	38
Puerto Rico	2.37(2.18-2.58)	84	2.93(2.62-3.25)	85	1.49(1.18,1.80)	-0.89(-1.08,-0.70)	1.37(1.21,1.53)	0.72(0.59,0.86)	92
Saint Kitts and Nevis	2.15(1.82-2.57)	102	2.93(2.48-3.54)	84	2.69(2.40,2.97)	-0.82(-0.91,-0.72)	1.81(1.69,1.92)	1.07(0.97,1.17)	39
San Marino	8.46(7.22-9.99)	32	9.06(7.71-10.59)	36	0.60(0.54,0.66)	-0.15(-0.18,-0.11)	0.21(0.19,0.24)	0.24(0.22,0.27)	155
Tokelau	0.51(0.43-0.61)	181	0.64(0.54-0.78)	186	0.24(0.09,0.39)	-0.16(-0.56,0.24)	2.28(2.14,2.42)	0.77(0.62,0.91)	85
Tuvalu	0.48(0.40-0.58)	186	0.69(0.57-0.83)	183	1.06(1.00,1.12)	1.35(1.23,1.47)	1.11(1.05,1.17)	1.19(1.14,1.25)	32
United States Virgin Islands	2.24(1.89-2.65)	95	2.91(2.47-3.50)	86	2.21(2.09,2.33)	-0.39(-0.53,-0.24)	1.01(0.68,1.34)	0.94(0.82,1.06)	58
South Sudan	0.94(0.80-1.14)	159	0.99(0.84-1.20)	171	0.05(-0.04,0.14)	0.45(0.25,0.64)	0.04(-0.01,0.08)	0.18(0.11,0.24)	162
Sudan	2.23(1.86-2.68)	97	2.77(2.32-3.33)	90	0.71(0.68,0.74)	0.53(0.50,0.56)	0.98(0.94,1.03)	0.73(0.71,0.76)	91
Multiple sclerosis									
China	0.18(0.15-0.22)	183	0.17(0.14-0.21)	185	-0.50(-0.51,-0.48)	-0.50(-0.51,-0.48)	0.48(0.38,0.58)	-0.19(-0.23,-0.16)	190
Democratic People's Republic of Korea	0.30(0.24-0.36)	136	0.29(0.24-0.35)	143	-0.37(-0.41,-0.33)	-0.23(-0.28,-0.17)	0.24(0.22,0.26)	-0.12(-0.15,-0.10)	180

Taiwan (Province of China)	0.17(0.15-0.19)	185	0.27(0.23-0.31)	151	-0.61(-0.67,-0.55)	3.62(3.49,3.76)	2.17(1.97,2.36)	1.55(1.47,1.63)	1
Cambodia	0.19(0.15-0.22)	179	0.18(0.15-0.22)	182	-0.18(-0.23,-0.13)	-0.14(-0.15,-0.13)	-0.07(-0.10,-0.03)	-0.13(-0.15,-0.11)	183
Indonesia	0.16(0.13-0.19)	188	0.16(0.13-0.19)	187	-0.10(-0.11,-0.10)	-0.01(-0.02,0.00)	0.05(0.03,0.08)	-0.02(-0.03,-0.01)	152
Lao People's Democratic Republic	0.23(0.19-0.27)	160	0.22(0.18-0.26)	164	-0.25(-0.28,-0.22)	-0.20(-0.29,-0.12)	0.15(0.10,0.21)	-0.10(-0.14,-0.07)	175
Malaysia	0.13(0.10-0.15)	202	0.12(0.10-0.15)	202	0.06(0.05,0.08)	-0.04(-0.06,-0.02)	-0.20(-0.34,-0.06)	-0.06(-0.10,-0.01)	163
Maldives	0.12(0.10-0.15)	203	0.11(0.09-0.14)	203	-0.18(-0.20,-0.16)	-0.29(-0.29,-0.28)	-0.50(-0.53,-0.47)	-0.32(-0.34,-0.31)	195
Myanmar	0.26(0.21-0.31)	148	0.25(0.21-0.30)	156	-0.11(-0.18,-0.03)	-0.15(-0.16,-0.13)	-0.15(-0.16,-0.13)	-0.13(-0.15,-0.10)	184
Philippines	0.19(0.16-0.23)	176	0.19(0.16-0.23)	176	-0.12(-0.16,-0.08)	0.02(-0.01,0.06)	0.27(0.25,0.28)	0.06(0.04,0.08)	128
Sri Lanka	0.16(0.13-0.19)	187	0.16(0.13-0.19)	189	0.28(0.27,0.29)	-0.04(-0.05,-0.03)	-0.53(-0.64,-0.43)	-0.10(-0.13,-0.06)	176
Thailand	0.19(0.15-0.22)	180	0.18(0.15-0.22)	183	-0.19(-0.22,-0.17)	-0.15(-0.16,-0.14)	-0.05(-0.06,-0.04)	-0.13(-0.14,-0.12)	185
Timor-Leste	0.15(0.12-0.18)	192	0.15(0.12-0.19)	191	-0.12(-0.18,-0.06)	-0.09(-0.11,-0.06)	0.20(0.17,0.23)	0.00(-0.03,0.02)	148
Viet Nam	0.20(0.16-0.24)	168	0.20(0.16-0.24)	172	-0.14(-0.18,-0.10)	-0.04(-0.09,0.01)	0.02(0.01,0.03)	-0.05(-0.07,-0.03)	159
Fiji	0.19(0.15-0.22)	182	0.18(0.15-0.22)	181	-0.27(-0.32,-0.22)	-0.05(-0.08,-0.01)	0.19(0.16,0.22)	-0.04(-0.07,-0.02)	156
Kiribati	0.15(0.12-0.17)	195	0.14(0.12-0.17)	196	0.53(0.51,0.54)	-0.03(-0.05,-0.01)	-0.90(-1.03,-0.77)	-0.13(-0.18,-0.09)	186
Marshall Islands	0.15(0.13-0.18)	191	0.15(0.12-0.18)	193	0.23(0.20,0.25)	-0.03(-0.07,0.01)	-0.42(-0.46,-0.37)	-0.07(-0.09,-0.04)	168
Micronesia (Federated States of)	0.15(0.12-0.18)	194	0.14(0.11-0.17)	195	0.10(0.09,0.10)	-0.08(-0.09,-0.07)	-0.38(-0.42,-0.34)	-0.12(-0.13,-0.11)	181
Papua New Guinea	0.13(0.10-0.16)	200	0.13(0.10-0.15)	200	-0.21(-0.26,-0.16)	-0.03(-0.10,0.04)	0.05(0.02,0.07)	-0.06(-0.09,-0.04)	164
Samoa	0.17(0.14-0.20)	186	0.16(0.13-0.20)	186	-0.14(-0.18,-0.11)	-0.09(-0.13,-0.05)	-0.07(-0.07,-0.06)	-0.09(-0.11,-0.08)	173
Solomon Islands	0.16(0.13-0.19)	190	0.16(0.13-0.19)	190	0.10(0.09,0.10)	-0.06(-0.07,-0.06)	-0.35(-0.38,-0.31)	-0.10(-0.11,-0.09)	177
Tonga	0.20(0.16-0.24)	171	0.20(0.16-0.24)	173	-0.33(-0.36,-0.29)	-0.05(-0.08,-0.03)	0.21(0.19,0.23)	-0.06(-0.07,-0.04)	165
Vanuatu	0.19(0.15-0.23)	178	0.19(0.15-0.22)	180	-0.20(-0.21,-0.19)	-0.05(-0.05,-0.04)	0.10(0.09,0.10)	-0.05(-0.05,-0.05)	160
Armenia	1.11(0.97-1.28)	60	1.41(1.23-1.60)	50	1.01(0.94,1.07)	0.83(0.75,0.92)	0.64(0.58,0.70)	0.83(0.79,0.87)	16
Azerbaijan	0.84(0.70-0.98)	74	0.91(0.75-1.07)	74	-0.38(-0.44,-0.32)	0.30(0.24,0.37)	1.07(0.57,1.57)	0.32(0.16,0.48)	67
Georgia	0.89(0.74-1.05)	71	1.11(0.94-1.28)	63	0.60(0.11,1.10)	0.66(0.56,0.76)	1.25(1.05,1.46)	0.83(0.65,1.00)	17
Kazakhstan	2.75(2.46-3.06)	14	2.77(2.48-3.10)	18	0.71(0.68,0.74)	0.30(0.26,0.34)	-0.89(-1.24,-0.53)	0.04(-0.07,0.16)	138
Kyrgyzstan	0.85(0.72-1.01)	73	0.87(0.71-1.03)	77	-0.79(-0.84,-0.75)	0.06(0.01,0.11)	1.06(0.67,1.45)	0.10(-0.02,0.22)	109
Mongolia	1.02(0.84-1.23)	63	1.03(0.85-1.22)	66	-1.21(-1.28,-1.14)	0.08(0.00,0.15)	1.45(0.88,2.01)	0.09(-0.09,0.27)	115
Tajikistan	0.78(0.65-0.93)	76	0.83(0.69-0.98)	79	-0.55(-0.61,-0.48)	0.21(0.14,0.28)	1.13(0.61,1.65)	0.25(0.09,0.42)	79
Turkmenistan	1.56(1.40-1.74)	34	1.60(1.43-1.78)	38	0.15(0.14,0.16)	0.09(0.07,0.12)	-0.02(-0.11,0.06)	0.07(0.04,0.10)	123
Uzbekistan	1.27(1.10-1.44)	44	1.02(0.86-1.19)	68	-0.78(-0.84,-0.72)	-0.53(-0.55,-0.51)	-0.92(-1.01,-0.82)	-0.76(-0.79,-0.72)	202
Albania	4.07(3.73-4.40)	4	3.05(2.80-3.29)	13	0.14(0.03,0.26)	-0.44(-0.57,-0.31)	-2.48(-3.69,-1.25)	-0.92(-1.31,-0.52)	203
Bosnia and Herzegovina	1.14(0.99-1.30)	57	1.14(1.01-1.26)	62	0.14(0.10,0.18)	-0.04(-0.05,-0.02)	-0.11(-0.13,-0.09)	-0.01(-0.03,0.01)	150
Bulgaria	2.09(1.91-2.31)	22	1.93(1.74-2.12)	28	0.40(0.32,0.47)	0.04(-0.03,0.12)	-1.56(-2.20,-0.92)	-0.35(-0.55,-0.14)	196
Croatia	0.97(0.84-1.09)	67	1.18(1.04-1.32)	58	1.21(1.14,1.28)	0.75(0.70,0.79)	0.12(0.09,0.15)	0.70(0.66,0.73)	27
Czechia	1.56(1.39-1.72)	35	1.38(1.21-1.54)	51	-0.18(-0.26,-0.11)	-0.47(-0.55,-0.38)	-0.60(-0.66,-0.54)	-0.42(-0.47,-0.38)	198
Hungary	1.93(1.80-2.08)	26	1.45(1.27-1.63)	48	0.08(0.02,0.14)	-0.09(-0.21,0.03)	-3.03(-3.63,-2.44)	-0.96(-1.16,-0.76)	204
North Macedonia	1.31(1.14-1.47)	43	1.58(1.39-1.76)	39	1.09(1.06,1.13)	0.67(0.63,0.71)	0.10(0.03,0.17)	0.62(0.60,0.65)	34
Montenegro	1.95(1.77-2.14)	24	2.03(1.85-2.23)	26	0.56(0.54,0.59)	0.33(0.31,0.36)	-0.50(-0.73,-0.27)	0.14(0.06,0.21)	100

Poland	2.51(2.17-2.84)	16	2.30(2.05-2.55)	22	-0.56(-0.65,-0.47)	-0.16(-0.21,-0.11)	-0.26(-0.28,-0.25)	-0.31(-0.35,-0.28)	194
Romania	0.85(0.73-0.98)	72	0.77(0.64-0.89)	84	-0.43(-0.45,-0.41)	-0.26(-0.33,-0.20)	-0.41(-0.52,-0.31)	-0.36(-0.40,-0.32)	197
Serbia	1.57(1.42-1.73)	33	1.91(1.73-2.08)	29	1.44(1.30,1.57)	0.39(0.33,0.44)	0.29(0.27,0.31)	0.68(0.63,0.73)	28
Slovakia	1.05(0.90-1.21)	61	1.16(1.00-1.33)	61	-0.01(-0.02,0.01)	0.36(0.25,0.47)	0.68(0.59,0.78)	0.35(0.30,0.39)	63
Slovenia	1.62(1.45-1.79)	32	1.69(1.52-1.86)	35	0.49(0.48,0.51)	0.18(0.14,0.21)	-0.23(-0.32,-0.14)	0.15(0.12,0.18)	97
Belarus	0.93(0.77-1.08)	70	0.90(0.75-1.06)	75	-0.27(-0.33,-0.20)	-0.01(-0.06,0.05)	0.08(-0.08,0.25)	-0.06(-0.12,0.00)	166
Estonia	1.26(1.09-1.42)	48	1.10(0.95-1.25)	64	-0.52(-0.56,-0.48)	-0.15(-0.19,-0.11)	-0.71(-0.87,-0.55)	-0.45(-0.50,-0.40)	199
Latvia	1.35(1.19-1.52)	41	1.26(1.10-1.43)	54	-0.32(-0.38,-0.27)	-0.09(-0.12,-0.06)	-0.30(-0.45,-0.16)	-0.23(-0.28,-0.18)	191
Lithuania	1.26(1.11-1.43)	45	1.18(1.02-1.34)	59	-0.20(-0.22,-0.18)	-0.05(-0.06,-0.04)	-0.50(-0.53,-0.46)	-0.23(-0.25,-0.22)	192
Republic of Moldova	0.66(0.53-0.79)	84	0.68(0.58-0.79)	87	-0.20(-0.49,0.10)	0.04(-0.18,0.27)	0.47(0.41,0.54)	0.13(0.01,0.26)	104
Russian Federation	1.13(0.96-1.30)	59	0.94(0.79-1.09)	73	-0.83(-0.89,-0.78)	-0.23(-0.28,-0.17)	-0.85(-1.05,-0.65)	-0.63(-0.70,-0.56)	201
Ukraine	1.76(1.51-2.01)	28	1.47(1.26-1.69)	45	-0.46(-0.56,-0.37)	-0.11(-0.15,-0.06)	-1.35(-1.66,-1.03)	-0.60(-0.71,-0.50)	200
Brunei Darussalam	0.15(0.12-0.18)	193	0.15(0.12-0.19)	192	0.88(0.86,0.90)	0.09(0.07,0.11)	-0.70(-0.88,-0.52)	0.09(0.04,0.15)	116
Japan	0.34(0.27-0.41)	125	0.37(0.30-0.45)	122	0.46(0.34,0.58)	-0.04(-0.07,0.00)	0.56(0.48,0.64)	0.34(0.29,0.39)	64
Republic of Korea	0.38(0.30-0.46)	109	0.37(0.30-0.45)	121	-0.34(-0.37,-0.31)	-0.04(-0.07,-0.01)	0.17(0.15,0.19)	-0.07(-0.09,-0.05)	169
Singapore	0.13(0.10-0.15)	201	0.13(0.10-0.15)	201	0.73(0.70,0.76)	0.05(0.01,0.08)	-0.88(-1.17,-0.60)	-0.04(-0.13,0.05)	157
Australia	1.55(1.39-1.71)	36	2.20(1.91-2.48)	24	3.63(3.41,3.86)	0.36(0.30,0.42)	-0.69(-0.89,-0.48)	1.21(1.11,1.32)	5
New Zealand	1.51(1.28-1.72)	37	1.41(1.20-1.61)	49	-0.28(-0.29,-0.28)	-0.10(-0.10,-0.09)	-0.34(-0.40,-0.27)	-0.23(-0.25,-0.21)	193
Andorra	1.94(1.71-2.22)	25	2.20(1.93-2.51)	23	0.87(0.85,0.90)	0.33(0.31,0.36)	0.12(0.07,0.16)	0.45(0.43,0.47)	49
Austria	2.18(1.90-2.49)	21	2.83(2.50-3.22)	16	1.40(1.36,1.44)	0.60(0.57,0.62)	0.74(0.55,0.94)	0.92(0.85,0.98)	8
Belgium	2.46(2.20-2.71)	17	2.70(2.37-3.12)	19	0.36(0.34,0.38)	0.43(0.38,0.48)	0.14(0.10,0.18)	0.31(0.29,0.34)	68
Cyprus	1.20(1.06-1.33)	52	1.53(1.30-1.78)	40	0.50(0.46,0.53)	1.56(1.50,1.63)	0.33(0.28,0.37)	0.85(0.82,0.88)	12
Denmark	4.02(3.73-4.35)	5	4.30(3.87-4.79)	6	0.26(0.21,0.30)	0.58(0.54,0.62)	-0.25(-0.41,-0.09)	0.22(0.17,0.27)	83
Finland	2.53(2.31-2.75)	15	2.90(2.66-3.15)	15	0.51(0.44,0.58)	-0.25(-0.39,-0.10)	1.07(0.75,1.38)	0.40(0.28,0.51)	55
France	2.34(2.03-2.71)	18	2.99(2.68-3.32)	14	1.51(1.42,1.59)	0.58(0.56,0.60)	0.24(0.20,0.29)	0.84(0.80,0.87)	13
Germany	2.75(2.45-3.12)	13	3.15(2.79-3.60)	12	0.79(0.78,0.81)	0.43(0.41,0.46)	0.17(0.13,0.22)	0.47(0.45,0.48)	44
Greece	1.03(0.89-1.16)	62	1.32(1.13-1.52)	53	0.75(0.74,0.77)	0.58(0.56,0.60)	1.35(1.29,1.41)	0.88(0.86,0.90)	11
Iceland	3.76(3.57-3.97)	6	3.78(3.29-4.37)	7	0.12(0.10,0.13)	0.29(0.27,0.31)	-0.45(-0.79,-0.10)	0.01(-0.09,0.12)	145
Ireland	4.09(3.63-4.56)	3	4.34(3.74-5.00)	5	0.70(0.63,0.77)	0.82(0.78,0.85)	-0.92(-1.10,-0.75)	0.22(0.16,0.28)	84
Israel	1.14(0.94-1.33)	58	1.24(1.03-1.45)	56	-0.07(-0.12,-0.03)	0.44(0.42,0.47)	0.52(0.44,0.61)	0.31(0.28,0.34)	69
Italy	2.22(1.90-2.55)	20	2.81(2.39-3.27)	17	0.38(0.36,0.39)	1.22(1.18,1.26)	0.70(0.61,0.79)	0.80(0.77,0.84)	21
Luxembourg	2.97(2.66-3.32)	12	3.16(2.78-3.61)	11	0.49(0.47,0.51)	0.24(0.22,0.26)	-0.14(-0.30,0.02)	0.21(0.16,0.26)	86
Malta	0.77(0.67-0.87)	78	0.97(0.81-1.12)	71	0.27(0.25,0.30)	0.27(0.25,0.30)	1.98(1.67,2.29)	0.80(0.70,0.90)	22
Netherlands	3.08(2.76-3.48)	11	3.25(2.84-3.71)	10	0.16(0.10,0.21)	0.49(0.41,0.57)	-0.21(-0.29,-0.12)	0.17(0.12,0.21)	93
Norway	3.50(3.03-3.98)	7	5.05(4.33-5.84)	2	1.47(1.28,1.66)	0.88(0.49,1.27)	1.52(1.40,1.64)	1.30(1.16,1.45)	4
Portugal	1.20(1.00-1.38)	53	1.16(1.06-1.26)	60	-0.16(-0.17,-0.15)	-0.05(-0.05,-0.04)	-0.12(-0.17,-0.07)	-0.11(-0.12,-0.09)	179
Spain	1.87(1.67-2.07)	27	2.63(2.41-2.83)	20	1.98(1.20,2.76)	0.84(0.75,0.92)	0.84(0.75,0.92)	1.19(0.94,1.44)	7
Sweden	4.52(3.93-5.11)	1	5.32(4.68-6.01)	1	0.73(0.65,0.82)	0.67(0.59,0.75)	0.31(0.26,0.37)	0.58(0.54,0.63)	36

Switzerland	3.23(2.90-3.61)	10	3.38(3.01-3.80)	9	0.33(0.30,0.36)	0.18(0.12,0.25)	-0.11(-0.16,-0.06)	0.15(0.12,0.18)	98
United Kingdom	3.41(3.02-3.80)	9	4.40(3.89-4.95)	4	1.14(0.99,1.29)	0.77(0.71,0.83)	0.80(0.64,0.95)	0.90(0.82,0.98)	10
Argentina	0.97(0.81-1.12)	66	0.99(0.83-1.15)	69	0.40(0.38,0.41)	-0.01(-0.02,0.00)	-0.19(-0.28,-0.10)	0.07(0.05,0.10)	124
Chile	0.78(0.63-0.93)	77	0.82(0.67-0.98)	80	-0.32(-0.34,-0.30)	0.11(0.09,0.13)	0.82(0.64,1.00)	0.20(0.14,0.26)	88
Uruguay	0.93(0.79-1.08)	69	0.96(0.81-1.11)	72	0.30(0.28,0.32)	0.11(0.05,0.18)	-0.15(-0.28,-0.03)	0.09(0.04,0.14)	117
Canada	4.12(4.02-4.22)	2	4.76(4.63-4.89)	3	0.37(0.28,0.46)	0.19(0.16,0.22)	0.98(0.88,1.08)	0.49(0.44,0.54)	42
United States of America	3.41(2.96-3.87)	8	3.44(3.14-3.73)	8	-0.45(-0.54,-0.35)	0.29(0.27,0.31)	0.19(0.14,0.25)	0.04(0.01,0.08)	139
Antigua and Barbuda	0.60(0.51-0.68)	88	0.76(0.65-0.86)	85	2.02(1.98,2.06)	0.80(0.74,0.85)	-0.33(-0.36,-0.30)	0.81(0.79,0.84)	19
Bahamas	0.51(0.42-0.60)	90	0.57(0.47-0.67)	90	0.57(0.56,0.59)	0.39(0.38,0.41)	0.24(0.20,0.29)	0.40(0.38,0.42)	56
Barbados	0.56(0.47-0.65)	89	0.72(0.61-0.82)	86	1.93(1.85,2.01)	0.81(0.75,0.87)	-0.31(-0.35,-0.26)	0.81(0.78,0.85)	20
Belize	0.34(0.28-0.41)	123	0.38(0.30-0.45)	117	0.08(0.06,0.10)	0.37(0.35,0.39)	0.58(0.45,0.71)	0.34(0.30,0.38)	65
Cuba	0.50(0.42-0.59)	91	0.57(0.48-0.66)	91	0.80(0.78,0.83)	0.49(0.38,0.60)	-0.06(-0.09,-0.04)	0.41(0.37,0.44)	52
Dominica	0.33(0.27-0.40)	126	0.36(0.29-0.43)	123	0.14(0.13,0.16)	0.33(0.29,0.38)	0.35(0.26,0.44)	0.28(0.25,0.31)	73
Dominican Republic	0.36(0.29-0.43)	117	0.39(0.31-0.47)	111	-0.08(-0.10,-0.06)	0.25(0.23,0.27)	0.69(0.53,0.86)	0.28(0.23,0.33)	74
Grenada	0.46(0.38-0.54)	93	0.53(0.44-0.61)	93	1.41(1.31,1.51)	0.32(0.23,0.40)	-0.47(-0.56,-0.37)	0.41(0.35,0.48)	53
Guyana	0.25(0.20-0.30)	150	0.27(0.22-0.33)	152	0.30(0.27,0.34)	0.34(0.33,0.35)	0.23(0.21,0.25)	0.29(0.28,0.31)	72
Haiti	0.45(0.37-0.53)	95	0.50(0.41-0.59)	96	0.41(0.36,0.45)	0.34(0.33,0.35)	0.35(0.31,0.39)	0.36(0.34,0.38)	62
Jamaica	0.35(0.28-0.42)	119	0.38(0.31-0.46)	115	0.08(0.05,0.11)	0.30(0.27,0.32)	0.48(0.31,0.66)	0.28(0.22,0.34)	75
Saint Lucia	0.34(0.28-0.41)	122	0.37(0.30-0.45)	119	0.43(0.39,0.48)	0.36(0.32,0.40)	0.08(0.07,0.09)	0.28(0.26,0.30)	76
Saint Vincent and the Grenadines	0.33(0.27-0.40)	127	0.37(0.30-0.44)	120	0.45(0.43,0.47)	0.46(0.43,0.50)	0.27(0.23,0.32)	0.39(0.37,0.41)	57
Suriname	0.24(0.19-0.29)	153	0.27(0.21-0.32)	153	0.60(0.58,0.62)	0.37(0.37,0.38)	0.15(0.13,0.16)	0.37(0.37,0.38)	61
Trinidad and Tobago	0.29(0.23-0.35)	141	0.33(0.27-0.39)	138	0.57(0.53,0.61)	0.41(0.40,0.43)	0.21(0.17,0.26)	0.39(0.37,0.41)	58
Bolivia (Plurinational State of)	0.36(0.29-0.43)	115	0.41(0.33-0.48)	108	0.59(0.56,0.62)	0.47(0.47,0.48)	0.23(0.19,0.26)	0.42(0.40,0.44)	51
Ecuador	0.21(0.17-0.25)	164	0.26(0.21-0.30)	154	1.28(1.24,1.32)	0.56(0.54,0.57)	0.19(0.13,0.25)	0.67(0.65,0.69)	29
Peru	0.27(0.22-0.32)	146	0.33(0.26-0.39)	136	1.12(1.08,1.16)	0.72(0.66,0.78)	0.36(0.34,0.38)	0.73(0.70,0.75)	24
Colombia	0.19(0.16-0.23)	173	0.22(0.18-0.27)	163	1.24(1.11,1.37)	0.46(0.38,0.54)	-0.32(-0.43,-0.21)	0.46(0.39,0.53)	46
Costa Rica	0.29(0.24-0.34)	140	0.34(0.28-0.40)	129	1.37(1.35,1.39)	0.72(0.70,0.75)	-0.59(-0.78,-0.39)	0.51(0.45,0.57)	41
El Salvador	0.24(0.20-0.29)	152	0.28(0.22-0.33)	149	0.63(0.61,0.66)	0.43(0.42,0.44)	0.34(0.31,0.37)	0.46(0.45,0.48)	47
Guatemala	0.27(0.22-0.33)	145	0.33(0.27-0.39)	135	0.92(0.88,0.96)	0.65(0.61,0.69)	0.46(0.44,0.48)	0.67(0.65,0.69)	30
Honduras	0.26(0.21-0.31)	149	0.29(0.23-0.34)	146	0.40(0.37,0.43)	0.37(0.36,0.39)	0.36(0.34,0.38)	0.38(0.36,0.39)	60
Mexico	0.41(0.33-0.48)	101	0.53(0.44-0.62)	92	1.59(1.56,1.63)	0.88(0.84,0.91)	0.29(0.27,0.31)	0.91(0.89,0.93)	9
Nicaragua	0.24(0.19-0.28)	155	0.27(0.22-0.32)	150	0.75(0.72,0.77)	0.40(0.38,0.42)	0.15(0.14,0.17)	0.43(0.42,0.45)	50
Panama	0.22(0.18-0.26)	161	0.24(0.20-0.28)	159	0.76(0.66,0.87)	0.32(0.27,0.38)	-0.38(-0.46,-0.30)	0.24(0.19,0.29)	81
Venezuela (Bolivarian Republic of)	0.24(0.20-0.29)	151	0.28(0.23-0.33)	147	1.42(1.28,1.55)	0.52(0.42,0.63)	-0.49(-0.57,-0.40)	0.49(0.42,0.55)	43
Brazil	0.75(0.63-0.88)	79	0.80(0.67-0.94)	82	0.05(-0.04,0.14)	0.05(-0.04,0.14)	0.03(-0.51,0.57)	0.10(-0.10,0.30)	110
Paraguay	0.60(0.48-0.72)	87	0.63(0.50-0.75)	89	-0.46(-0.48,-0.44)	0.08(0.06,0.10)	0.88(0.71,1.06)	0.16(0.11,0.22)	95
Algeria	1.24(1.02-1.45)	50	1.50(1.26-1.77)	42	0.81(0.76,0.86)	0.61(0.60,0.62)	0.57(0.56,0.58)	0.66(0.64,0.68)	31
Bahrain	0.94(0.76-1.12)	68	1.20(0.98-1.45)	57	0.88(0.76,1.01)	0.45(0.40,0.50)	1.20(1.14,1.27)	0.84(0.79,0.90)	14

Egypt	0.69(0.56-0.82)	83	0.81(0.66-0.97)	81	0.40(0.23,0.57)	0.34(0.28,0.41)	0.93(0.88,0.97)	0.56(0.50,0.62)	37
Iran (Islamic Republic of)	2.07(1.78-2.38)	23	1.97(1.71-2.23)	27	-1.55(-2.07,-1.02)	0.44(0.41,0.47)	0.44(0.41,0.47)	-0.18(-0.35,-0.01)	189
Iraq	1.18(0.94-1.41)	56	1.34(1.08-1.63)	52	-0.20(-0.27,-0.13)	0.46(0.43,0.49)	1.10(1.04,1.17)	0.46(0.42,0.49)	48
Jordan	1.65(1.44-1.86)	31	1.61(1.31-1.92)	37	0.41(0.39,0.43)	-0.82(-0.89,-0.74)	0.38(0.31,0.45)	-0.07(-0.11,-0.04)	170
Kuwait	1.19(0.99-1.41)	54	1.78(1.49-2.10)	32	2.23(2.11,2.36)	1.08(1.05,1.12)	0.78(0.69,0.86)	1.42(1.36,1.47)	3
Lebanon	1.45(1.21-1.71)	39	1.84(1.52-2.16)	30	1.05(1.01,1.09)	0.97(0.78,1.16)	0.48(0.43,0.52)	0.83(0.76,0.89)	18
Libya	1.18(0.97-1.39)	55	1.48(1.26-1.73)	43	1.38(1.34,1.42)	0.77(0.69,0.84)	0.21(0.17,0.25)	0.79(0.75,0.82)	23
Morocco	1.23(1.00-1.46)	51	1.47(1.23-1.75)	44	0.58(0.53,0.62)	0.54(0.43,0.65)	0.87(0.78,0.96)	0.66(0.61,0.71)	32
Palestine	1.26(1.03-1.49)	47	1.46(1.20-1.75)	46	0.45(0.38,0.53)	0.43(0.41,0.45)	0.69(0.67,0.70)	0.52(0.50,0.55)	40
Oman	0.97(0.81-1.14)	65	1.24(1.05-1.45)	55	1.88(1.79,1.96)	0.60(0.49,0.71)	0.11(0.01,0.21)	0.84(0.77,0.90)	15
Qatar	1.26(1.01-1.52)	49	1.81(1.59-2.04)	31	4.21(4.00,4.42)	-0.36(-0.45,-0.27)	0.07(-0.03,0.16)	1.20(1.11,1.28)	6
Saudi Arabia	0.83(0.67-1.00)	75	1.03(0.82-1.24)	67	0.60(0.53,0.67)	0.59(0.53,0.64)	0.95(0.93,0.97)	0.72(0.69,0.75)	26
Syrian Arab Republic	1.26(1.02-1.53)	46	1.52(1.23-1.84)	41	0.16(0.01,0.30)	0.55(0.51,0.58)	1.19(0.99,1.39)	0.64(0.56,0.73)	33
Tunisia	1.41(1.17-1.68)	40	1.74(1.45-2.06)	33	0.80(0.75,0.85)	0.64(0.60,0.67)	0.73(0.71,0.74)	0.73(0.71,0.75)	25
Turkey	1.73(1.64-1.81)	30	1.69(1.61-1.78)	34	-0.06(-0.09,-0.03)	-0.09(-0.09,-0.08)	-0.06(-0.11,-0.01)	-0.07(-0.09,-0.05)	171
United Arab Emirates	1.01(0.86-1.17)	64	1.06(0.92-1.20)	65	-0.08(-0.25,0.09)	0.14(-0.16,0.43)	0.39(0.27,0.50)	0.18(0.06,0.29)	91
Yemen	0.75(0.60-0.90)	80	0.88(0.72-1.05)	76	0.50(0.45,0.56)	0.62(0.56,0.68)	0.50(0.47,0.54)	0.55(0.52,0.58)	38
Afghanistan	1.46(1.22-1.70)	38	1.64(1.40-1.90)	36	-0.03(-0.09,0.04)	0.46(0.44,0.48)	0.78(0.76,0.80)	0.41(0.39,0.44)	54
Bangladesh	0.37(0.30-0.44)	113	0.37(0.30-0.45)	118	-0.18(-0.20,-0.16)	0.14(0.10,0.19)	0.24(0.23,0.25)	0.06(0.05,0.08)	129
Bhutan	0.40(0.32-0.48)	106	0.41(0.33-0.49)	106	-0.21(-0.23,-0.19)	0.10(0.08,0.12)	0.51(0.36,0.65)	0.13(0.08,0.17)	105
India	0.36(0.29-0.43)	114	0.40(0.32-0.47)	110	0.11(0.05,0.16)	0.38(0.34,0.41)	0.53(0.50,0.56)	0.34(0.32,0.37)	66
Nepal	0.41(0.34-0.49)	100	0.44(0.36-0.52)	100	-0.22(-0.28,-0.16)	0.23(0.17,0.29)	0.68(0.62,0.74)	0.23(0.19,0.27)	82
Pakistan	0.45(0.36-0.53)	96	0.47(0.39-0.56)	98	-0.20(-0.28,-0.12)	0.25(0.21,0.28)	0.56(0.42,0.70)	0.21(0.15,0.26)	87
Angola	0.28(0.23-0.34)	143	0.29(0.23-0.35)	144	-0.26(-0.28,-0.25)	0.12(0.08,0.15)	0.41(0.40,0.42)	0.09(0.08,0.10)	118
Central African Republic	0.24(0.19-0.29)	154	0.25(0.20-0.30)	157	-0.22(-0.23,-0.21)	0.10(0.08,0.12)	0.44(0.43,0.45)	0.11(0.10,0.11)	108
Congo	0.20(0.16-0.24)	170	0.20(0.16-0.25)	170	0.00(-0.03,0.03)	0.13(0.10,0.17)	-0.03(-0.05,0.00)	0.04(0.02,0.06)	140
Democratic Republic of the Congo	0.20(0.16-0.24)	167	0.20(0.16-0.25)	168	-0.25(-0.29,-0.22)	0.08(0.07,0.09)	0.33(0.30,0.37)	0.06(0.04,0.07)	130
Equatorial Guinea	0.20(0.16-0.24)	169	0.19(0.15-0.23)	175	-0.24(-0.29,-0.19)	-0.10(-0.12,-0.09)	-0.06(-0.07,-0.05)	-0.13(-0.15,-0.11)	187
Gabon	0.19(0.15-0.23)	175	0.20(0.16-0.24)	171	0.14(0.12,0.17)	0.27(0.26,0.29)	0.05(0.03,0.07)	0.16(0.15,0.18)	96
Burundi	0.21(0.17-0.26)	162	0.21(0.17-0.26)	167	-0.43(-0.47,-0.39)	-0.11(-0.12,-0.09)	0.41(0.37,0.45)	-0.05(-0.07,-0.03)	161
Comoros	0.29(0.23-0.35)	142	0.29(0.23-0.35)	145	-0.26(-0.29,-0.23)	0.00(-0.04,0.04)	0.32(0.27,0.37)	0.02(0.00,0.04)	144
Djibouti	0.28(0.22-0.33)	144	0.28(0.22-0.34)	148	-0.29(-0.33,-0.25)	0.15(0.13,0.18)	0.34(0.32,0.35)	0.06(0.04,0.08)	131
Eritrea	0.33(0.27-0.40)	128	0.33(0.27-0.40)	131	-0.35(-0.43,-0.27)	-0.01(-0.04,0.03)	0.48(0.41,0.55)	0.04(0.00,0.08)	141
Ethiopia	0.26(0.21-0.32)	147	0.25(0.20-0.31)	155	-0.81(-0.84,-0.77)	-0.07(-0.12,-0.02)	0.56(0.51,0.61)	-0.10(-0.13,-0.07)	178
Kenya	0.21(0.17-0.25)	165	0.21(0.17-0.26)	165	-0.17(-0.17,-0.16)	0.15(0.14,0.17)	0.38(0.36,0.39)	0.12(0.11,0.12)	106
Madagascar	0.35(0.28-0.43)	118	0.36(0.29-0.44)	124	-0.44(-0.45,-0.42)	-0.03(-0.05,-0.01)	0.71(0.56,0.85)	0.08(0.03,0.12)	121
Malawi	0.29(0.24-0.36)	138	0.30(0.24-0.36)	142	-0.44(-0.48,-0.41)	0.10(0.00,0.20)	0.58(0.52,0.65)	0.07(0.03,0.11)	125
Mauritius	0.21(0.17-0.26)	163	0.21(0.17-0.26)	166	-0.30(-0.36,-0.24)	-0.02(-0.04,0.01)	0.32(0.28,0.35)	0.00(-0.02,0.03)	149

Mozambique	0.35(0.28-0.42)	121	0.35(0.29-0.42)	126	-0.37(-0.39,-0.34)	0.06(0.03,0.09)	0.58(0.35,0.82)	0.09(0.01,0.16)	119
Rwanda	0.21(0.16-0.25)	166	0.20(0.16-0.25)	169	-0.13(-0.24,-0.02)	-0.09(-0.10,-0.07)	0.02(0.01,0.03)	-0.07(-0.10,-0.03)	172
Seychelles	0.19(0.15-0.22)	181	0.19(0.16-0.22)	179	0.77(0.74,0.80)	0.05(0.02,0.08)	-0.81(-1.04,-0.57)	0.01(-0.06,0.09)	146
Somalia	0.23(0.18-0.28)	156	0.24(0.19-0.29)	161	-0.34(-0.36,-0.32)	0.05(-0.01,0.12)	0.43(0.31,0.54)	0.05(0.01,0.09)	136
United Republic of Tanzania	0.23(0.18-0.28)	158	0.23(0.19-0.29)	162	-0.29(-0.31,-0.27)	0.08(0.02,0.13)	0.41(0.38,0.45)	0.06(0.04,0.09)	132
Uganda	0.19(0.15-0.23)	177	0.19(0.15-0.24)	174	-0.23(-0.25,-0.22)	0.07(0.03,0.12)	0.46(0.38,0.55)	0.10(0.07,0.13)	111
Zambia	0.31(0.25-0.38)	134	0.31(0.25-0.37)	140	-0.33(-0.36,-0.30)	0.01(-0.03,0.04)	0.22(0.17,0.27)	-0.03(-0.06,-0.01)	154
Botswana	0.32(0.26-0.39)	132	0.33(0.26-0.40)	134	-0.29(-0.30,-0.28)	0.05(0.00,0.10)	0.48(0.47,0.49)	0.08(0.06,0.10)	122
Lesotho	0.40(0.33-0.49)	104	0.42(0.34-0.51)	102	-0.40(-0.41,-0.39)	0.08(0.06,0.09)	0.74(0.63,0.86)	0.14(0.10,0.18)	101
Namibia	0.33(0.26-0.39)	130	0.33(0.26-0.40)	137	-0.35(-0.36,-0.33)	-0.02(-0.02,-0.01)	0.40(0.39,0.42)	0.01(0.00,0.02)	147
South Africa	0.39(0.32-0.47)	107	0.40(0.32-0.48)	109	-0.55(-0.58,-0.53)	0.07(-0.04,0.17)	0.58(0.50,0.66)	0.03(-0.01,0.07)	143
Eswatini	0.38(0.30-0.46)	111	0.39(0.31-0.47)	112	-0.38(-0.39,-0.37)	0.05(0.04,0.06)	0.63(0.53,0.73)	0.10(0.07,0.13)	112
Zimbabwe	0.29(0.23-0.35)	139	0.31(0.25-0.38)	139	-0.07(-0.08,-0.07)	0.18(0.17,0.19)	0.50(0.45,0.55)	0.20(0.19,0.22)	89
Benin	0.35(0.28-0.42)	120	0.36(0.29-0.42)	125	-0.03(-0.04,-0.01)	0.19(0.17,0.20)	0.04(0.00,0.09)	0.07(0.05,0.08)	126
Burkina Faso	0.38(0.31-0.45)	110	0.38(0.31-0.46)	114	-0.26(-0.29,-0.22)	0.13(0.12,0.14)	0.23(0.19,0.26)	0.04(0.02,0.05)	142
Cameroon	0.32(0.26-0.39)	131	0.33(0.27-0.40)	132	0.21(0.19,0.22)	0.18(0.16,0.19)	-0.09(-0.23,0.04)	0.10(0.05,0.14)	113
Cabo Verde	0.38(0.31-0.46)	108	0.39(0.32-0.47)	113	-0.26(-0.30,-0.22)	0.14(0.04,0.23)	0.31(0.28,0.34)	0.06(0.03,0.10)	133
Chad	0.41(0.33-0.49)	102	0.42(0.34-0.50)	103	-0.26(-0.28,-0.24)	0.16(0.12,0.20)	0.39(0.38,0.40)	0.10(0.08,0.11)	114
C??te d'Ivoire	0.33(0.27-0.39)	129	0.34(0.28-0.41)	128	0.20(0.19,0.21)	0.27(0.25,0.28)	-0.08(-0.13,-0.04)	0.14(0.12,0.15)	102
Gambia	0.38(0.31-0.45)	112	0.41(0.33-0.48)	107	0.14(0.12,0.16)	0.37(0.36,0.39)	0.31(0.26,0.35)	0.28(0.27,0.30)	77
Ghana	0.65(0.56-0.74)	85	0.99(0.89-1.09)	70	2.62(2.52,2.73)	1.09(1.04,1.13)	0.67(0.59,0.75)	1.44(1.39,1.49)	2
Guinea	0.36(0.29-0.43)	116	0.38(0.31-0.45)	116	0.00(-0.05,0.05)	0.32(0.30,0.35)	0.22(0.20,0.25)	0.18(0.16,0.20)	92
Guinea-Bissau	0.41(0.33-0.48)	103	0.41(0.34-0.49)	105	-0.05(-0.05,-0.04)	0.19(0.18,0.20)	0.08(0.06,0.09)	0.06(0.06,0.07)	134
Liberia	0.30(0.24-0.36)	137	0.31(0.25-0.36)	141	-0.15(-0.20,-0.09)	0.11(0.09,0.13)	0.24(0.22,0.26)	0.07(0.05,0.09)	127
Mali	0.43(0.35-0.51)	98	0.44(0.36-0.52)	99	-0.32(-0.34,-0.31)	0.16(0.13,0.19)	0.34(0.31,0.37)	0.05(0.04,0.07)	137
Mauritania	0.49(0.40-0.58)	92	0.50(0.42-0.59)	95	-0.08(-0.10,-0.06)	0.16(0.15,0.18)	0.20(0.15,0.26)	0.09(0.07,0.11)	120
Niger	0.43(0.35-0.51)	99	0.43(0.35-0.51)	101	-0.38(-0.40,-0.35)	0.15(0.11,0.19)	0.38(0.33,0.43)	0.06(0.03,0.08)	135
Nigeria	0.31(0.25-0.38)	135	0.34(0.27-0.40)	130	-0.14(-0.19,-0.09)	0.34(0.31,0.37)	0.62(0.56,0.68)	0.28(0.25,0.31)	78
Sao Tome and Principe	0.23(0.19-0.28)	157	0.24(0.19-0.29)	158	0.11(0.09,0.13)	0.29(0.28,0.30)	0.05(0.01,0.10)	0.15(0.14,0.17)	99
Senegal	0.40(0.33-0.47)	105	0.41(0.34-0.49)	104	-0.01(-0.05,0.02)	0.20(0.19,0.22)	0.24(0.23,0.25)	0.14(0.13,0.16)	103
Sierra Leone	0.32(0.25-0.38)	133	0.33(0.27-0.40)	133	0.09(0.05,0.12)	0.14(0.11,0.18)	0.30(0.25,0.36)	0.17(0.15,0.20)	94
Togo	0.34(0.28-0.40)	124	0.35(0.29-0.42)	127	0.04(0.03,0.05)	0.26(0.25,0.28)	0.04(0.00,0.09)	0.12(0.11,0.13)	107
American Samoa	0.16(0.13-0.19)	189	0.16(0.13-0.19)	188	-0.17(-0.20,-0.14)	0.00(-0.01,0.01)	0.13(0.11,0.16)	-0.01(-0.03,0.00)	151
Bermuda	0.62(0.51-0.72)	86	0.66(0.55-0.78)	88	0.36(0.28,0.44)	0.31(0.24,0.38)	0.11(0.08,0.13)	0.25(0.22,0.29)	80
Cook Islands	0.19(0.16-0.23)	174	0.19(0.15-0.23)	177	-0.17(-0.18,-0.16)	-0.02(-0.02,-0.01)	0.13(0.11,0.15)	-0.02(-0.02,-0.01)	153
Greenland	2.34(1.92-2.80)	19	2.55(2.08-3.07)	21	-0.61(-0.63,-0.59)	0.16(0.14,0.18)	1.40(1.21,1.59)	0.31(0.25,0.37)	70
Guam	0.15(0.12-0.18)	196	0.14(0.11-0.18)	194	-0.16(-0.19,-0.14)	-0.03(-0.04,-0.02)	0.10(0.07,0.13)	-0.03(-0.05,-0.02)	155
Monaco	1.74(1.49-2.00)	29	2.07(1.79-2.38)	25	0.47(0.45,0.48)	0.70(0.59,0.81)	0.62(0.54,0.71)	0.60(0.55,0.64)	35

Nauru	0.11(0.09-0.13)	204	0.11(0.09-0.13)	204	0.28(0.28,0.29)	0.02(0.01,0.03)	-0.44(-0.50,-0.38)	-0.04(-0.06,-0.03)	158
Niue	0.19(0.16-0.23)	172	0.19(0.15-0.23)	178	-0.18(-0.22,-0.15)	-0.09(-0.12,-0.06)	0.10(0.09,0.11)	-0.05(-0.07,-0.04)	162
Northern Mariana Islands	0.17(0.14-0.21)	184	0.17(0.14-0.21)	184	0.11(0.00,0.21)	-0.16(-0.18,-0.15)	-0.16(-0.18,-0.15)	-0.06(-0.10,-0.02)	167
Palau	0.14(0.11-0.16)	198	0.13(0.11-0.16)	198	0.03(0.00,0.07)	0.04(-0.05,0.14)	-0.36(-0.42,-0.30)	-0.09(-0.13,-0.05)	174
Puerto Rico	0.45(0.37-0.53)	94	0.52(0.43-0.61)	94	1.07(0.99,1.14)	0.43(0.41,0.46)	-0.06(-0.08,-0.03)	0.47(0.44,0.50)	45
Saint Kitts and Nevis	0.73(0.62-0.84)	82	0.79(0.67-0.90)	83	1.21(1.19,1.23)	0.32(0.30,0.34)	-0.84(-1.02,-0.65)	0.22(0.16,0.28)	85
San Marino	1.35(1.09-1.60)	42	1.46(1.19-1.74)	47	-0.31(-0.34,-0.28)	0.21(0.18,0.24)	1.01(0.77,1.25)	0.30(0.22,0.37)	71
Tokelau	0.14(0.11-0.17)	197	0.13(0.11-0.16)	197	-0.16(-0.22,-0.09)	-0.19(-0.27,-0.10)	-0.03(-0.05,-0.01)	-0.12(-0.15,-0.08)	182
Tuvalu	0.13(0.11-0.16)	199	0.13(0.10-0.15)	199	-0.22(-0.24,-0.20)	-0.09(-0.09,-0.08)	-0.13(-0.18,-0.07)	-0.14(-0.16,-0.12)	188
United States Virgin Islands	0.44(0.36-0.51)	97	0.49(0.40-0.57)	97	0.81(0.75,0.86)	0.43(0.38,0.48)	-0.06(-0.07,-0.04)	0.39(0.36,0.41)	59
South Sudan	0.23(0.18-0.28)	159	0.24(0.19-0.29)	160	-0.19(-0.22,-0.16)	0.13(0.10,0.16)	0.64(0.41,0.87)	0.19(0.11,0.26)	90
Sudan	0.74(0.60-0.89)	81	0.87(0.71-1.03)	78	0.53(0.48,0.58)	0.45(0.42,0.49)	0.59(0.57,0.60)	0.53(0.51,0.55)	39
Rheumatoid arthritis									
China	11.45(10.31-12.71)	64	11.74(10.59-12.96)	89	0.28(0.27,0.29)	0.39(0.38,0.40)	-0.54(-0.62,-0.45)	0.08(0.05,0.11)	188
Democratic People's Republic of Korea	10.92(9.71-12.23)	69	9.86(8.72-11.08)	107	-0.76(-0.91,-0.61)	-0.25(-0.38,-0.12)	-0.07(-0.18,0.04)	-0.37(-0.45,-0.29)	203
Taiwan (Province of China)	10.13(9.91-10.43)	83	10.44(10.23-10.70)	104	0.18(0.18,0.18)	0.18(0.18,0.18)	-0.06(-0.11,-0.01)	0.11(0.09,0.12)	187
Cambodia	4.37(3.84-5.03)	148	5.19(4.58-5.93)	148	0.23(0.10,0.36)	0.89(0.75,1.03)	0.65(0.62,0.68)	0.59(0.52,0.66)	103
Indonesia	4.02(3.59-4.52)	175	4.07(3.63-4.55)	200	-0.07(-0.09,-0.05)	0.04(0.02,0.05)	0.18(0.13,0.23)	0.04(0.03,0.06)	191
Lao People's Democratic Republic	4.24(3.69-4.87)	154	5.02(4.40-5.76)	155	0.18(0.12,0.24)	0.71(0.68,0.74)	0.85(0.83,0.86)	0.58(0.56,0.61)	106
Malaysia	4.06(3.49-4.70)	169	4.77(4.07-5.57)	173	0.36(0.31,0.41)	0.67(0.64,0.69)	0.68(0.62,0.74)	0.58(0.55,0.61)	107
Maldives	4.74(4.16-5.49)	140	5.86(5.10-6.77)	138	1.04(0.76,1.33)	1.60(1.51,1.69)	-0.71(-0.93,-0.48)	0.71(0.59,0.83)	82
Myanmar	5.02(4.43-5.70)	138	6.21(5.52-7.05)	135	0.26(0.22,0.29)	0.93(0.90,0.95)	0.99(0.96,1.02)	0.74(0.72,0.76)	75
Philippines	5.81(5.18-6.47)	131	5.57(4.98-6.22)	141	-0.06(-0.10,-0.02)	-0.06(-0.10,-0.02)	-0.15(-0.49,0.19)	-0.09(-0.20,0.02)	196
Sri Lanka	4.04(3.46-4.72)	173	4.76(4.09-5.53)	176	0.13(0.11,0.15)	0.91(0.88,0.94)	0.65(0.60,0.70)	0.56(0.54,0.58)	118
Thailand	8.50(7.46-9.67)	107	9.60(8.37-10.98)	109	0.63(0.37,0.89)	1.18(1.13,1.24)	-0.73(-1.48,0.02)	0.41(0.16,0.66)	144
Timor-Leste	4.08(3.55-4.68)	165	4.83(4.22-5.52)	170	0.36(0.31,0.41)	0.81(0.79,0.82)	0.57(0.54,0.61)	0.58(0.56,0.61)	108
Viet Nam	4.51(3.93-5.17)	145	5.63(4.88-6.48)	140	0.51(0.48,0.54)	0.90(0.88,0.92)	0.88(0.83,0.94)	0.77(0.75,0.79)	66
Fiji	3.71(3.13-4.37)	194	4.04(3.41-4.79)	201	0.01(-0.21,0.23)	0.12(0.07,0.17)	0.90(0.77,1.02)	0.33(0.24,0.41)	160
Kiribati	3.27(2.78-3.82)	202	3.47(2.96-4.10)	204	0.16(0.04,0.27)	0.06(0.01,0.10)	0.52(0.41,0.64)	0.24(0.18,0.30)	175
Marshall Islands	4.25(3.71-4.89)	153	4.63(4.04-5.34)	181	0.03(-0.11,0.16)	0.36(0.34,0.39)	0.48(0.40,0.55)	0.29(0.24,0.34)	168
Micronesia (Federated States of)	4.11(3.57-4.76)	164	4.77(4.14-5.49)	175	0.67(0.63,0.71)	0.45(0.27,0.64)	0.39(0.33,0.45)	0.49(0.43,0.56)	129
Papua New Guinea	3.51(3.04-4.05)	197	3.76(3.26-4.34)	202	0.32(0.29,0.34)	-0.04(-0.08,0.00)	0.43(0.39,0.47)	0.24(0.21,0.26)	176
Samoa	4.36(3.79-5.05)	149	4.74(4.10-5.49)	178	0.61(0.49,0.74)	-0.11(-0.15,-0.07)	0.42(0.30,0.53)	0.31(0.24,0.37)	163
Solomon Islands	4.39(3.87-4.99)	147	4.94(4.34-5.67)	161	0.74(0.68,0.80)	-0.06(-0.14,0.02)	0.52(0.47,0.57)	0.42(0.38,0.45)	142
Tonga	4.04(3.46-4.70)	171	4.41(3.79-5.14)	192	0.45(0.31,0.60)	-0.01(-0.06,0.04)	0.50(0.39,0.62)	0.31(0.24,0.38)	164
Vanuatu	4.03(3.50-4.64)	174	4.30(3.74-4.97)	195	0.33(0.24,0.43)	-0.12(-0.21,-0.03)	0.40(0.38,0.43)	0.22(0.18,0.27)	177
Armenia	6.11(5.35-7.01)	127	8.22(7.23-9.43)	118	0.74(0.56,0.92)	1.16(1.10,1.21)	1.27(0.97,1.57)	1.06(0.95,1.18)	20
Azerbaijan	5.57(4.77-6.50)	134	6.91(5.94-8.03)	133	-0.30(-0.56,-0.04)	0.92(0.81,1.03)	1.73(1.49,1.97)	0.79(0.66,0.92)	60

Georgia	7.10(6.02-8.26)	116	7.73(6.58-8.94)	124	-0.27(-0.54,0.00)	0.14(0.06,0.22)	1.15(0.97,1.34)	0.34(0.23,0.45)	159
Kazakhstan	19.25(17.65-21.07)	13	25.50(23.45-28.01)	3	-0.22(-0.29,-0.15)	1.30(1.28,1.33)	1.80(1.69,1.91)	0.99(0.94,1.03)	26
Kyrgyzstan	14.74(13.20-16.46)	33	19.04(17.13-21.25)	28	0.30(0.18,0.42)	1.02(0.96,1.08)	1.35(1.30,1.40)	0.90(0.85,0.95)	40
Mongolia	7.29(6.35-8.33)	114	9.49(8.26-10.82)	110	-0.04(-0.13,0.06)	1.90(1.84,1.96)	0.97(0.93,1.01)	0.94(0.90,0.98)	34
Tajikistan	9.66(8.53-10.96)	93	11.44(10.07-13.08)	97	0.45(0.43,0.47)	0.60(0.59,0.62)	0.70(0.66,0.73)	0.58(0.57,0.59)	109
Turkmenistan	5.50(4.76-6.35)	135	6.91(6.03-7.90)	132	0.24(0.03,0.44)	0.68(0.52,0.84)	1.44(1.38,1.50)	0.82(0.73,0.91)	52
Uzbekistan	17.41(15.70-19.16)	20	20.49(18.48-22.59)	23	0.08(0.01,0.15)	0.87(0.77,0.96)	0.68(0.63,0.74)	0.58(0.53,0.62)	110
Albania	6.17(5.35-7.06)	123	8.69(7.57-9.93)	115	1.02(0.94,1.09)	1.32(1.22,1.42)	1.25(0.99,1.52)	1.20(1.10,1.29)	13
Bosnia and Herzegovina	10.04(8.94-11.11)	85	13.12(11.69-14.64)	71	0.77(0.48,1.06)	1.08(0.88,1.28)	0.77(0.68,0.86)	0.95(0.82,1.07)	32
Bulgaria	5.96(5.14-6.90)	129	6.74(5.84-7.81)	134	-0.18(-0.32,-0.04)	0.43(0.38,0.49)	1.15(1.05,1.25)	0.46(0.39,0.52)	135
Croatia	10.17(9.04-11.52)	80	12.97(11.49-14.74)	73	0.98(0.97,1.00)	0.77(0.76,0.79)	0.79(0.69,0.89)	0.85(0.81,0.88)	43
Czechia	7.74(6.71-8.75)	112	9.48(8.25-10.78)	111	0.71(0.37,1.05)	0.53(0.48,0.58)	0.98(0.86,1.11)	0.72(0.61,0.84)	79
Hungary	10.24(9.31-11.29)	77	11.62(10.53-12.85)	94	0.96(0.90,1.01)	0.58(0.53,0.63)	-0.24(-0.60,0.12)	0.45(0.33,0.57)	139
North Macedonia	5.61(4.84-6.53)	133	7.03(6.06-8.13)	131	0.50(0.38,0.62)	0.90(0.86,0.95)	1.00(0.76,1.23)	0.81(0.72,0.89)	56
Montenegro	6.92(5.97-7.97)	118	8.18(7.11-9.39)	119	-0.14(-0.27,-0.02)	0.85(0.81,0.89)	1.11(1.00,1.23)	0.60(0.54,0.67)	100
Poland	19.26(17.25-21.35)	12	16.02(14.29-17.76)	46	-0.33(-0.41,-0.26)	0.12(0.11,0.14)	-1.90(-2.11,-1.69)	-0.65(-0.72,-0.58)	204
Romania	5.86(5.01-6.79)	130	7.43(6.42-8.57)	127	0.64(0.47,0.81)	0.69(0.64,0.74)	1.20(1.09,1.32)	0.85(0.78,0.92)	44
Serbia	6.45(5.94-7.05)	120	9.05(7.94-10.27)	114	0.57(0.52,0.62)	0.57(0.52,0.62)	2.81(1.71,3.92)	1.26(0.92,1.60)	7
Slovakia	6.01(5.20-6.90)	128	7.36(6.36-8.42)	128	0.51(0.47,0.54)	0.62(0.57,0.66)	1.07(0.75,1.39)	0.72(0.62,0.83)	80
Slovenia	12.24(10.85-13.85)	59	15.60(13.92-17.70)	47	0.72(0.70,0.74)	1.18(1.14,1.21)	0.62(0.56,0.68)	0.84(0.81,0.86)	46
Belarus	4.79(4.12-5.60)	139	6.17(5.33-7.14)	137	0.05(-0.10,0.21)	1.02(0.93,1.10)	1.62(1.55,1.70)	0.91(0.84,0.98)	39
Estonia	13.85(12.54-15.11)	44	16.91(15.45-18.42)	40	0.35(0.18,0.53)	1.58(1.51,1.65)	-0.11(-0.45,0.24)	0.67(0.55,0.80)	89
Latvia	12.16(10.92-13.54)	60	14.42(12.92-16.06)	59	0.15(-0.02,0.32)	1.02(0.97,1.07)	0.46(0.34,0.58)	0.60(0.53,0.68)	101
Lithuania	13.27(12.07-14.66)	49	14.95(13.43-16.48)	54	0.48(0.02,0.93)	0.57(0.48,0.65)	0.16(-0.07,0.39)	0.41(0.25,0.58)	145
Republic of Moldova	5.11(4.45-5.92)	137	6.18(5.38-7.10)	136	-0.06(-0.20,0.08)	0.72(0.68,0.76)	1.41(1.31,1.50)	0.69(0.63,0.74)	87
Russian Federation	7.78(6.98-8.68)	111	8.11(7.29-9.05)	120	0.09(0.05,0.13)	0.28(0.26,0.29)	0.04(0.01,0.06)	0.14(0.12,0.16)	185
Ukraine	5.16(4.59-5.78)	136	5.42(4.82-6.12)	143	0.29(0.15,0.43)	0.06(0.00,0.12)	0.26(-0.15,0.68)	0.19(0.05,0.33)	181
Brunei Darussalam	9.09(8.09-10.29)	101	10.74(9.56-12.15)	103	1.00(0.97,1.03)	0.67(0.64,0.71)	0.08(-0.19,0.35)	0.59(0.50,0.68)	104
Japan	15.32(13.75-16.98)	28	14.81(13.23-16.41)	56	-0.21(-0.55,0.13)	0.21(0.06,0.35)	-0.63(-1.69,0.43)	-0.19(-0.54,0.17)	200
Republic of Korea	10.46(9.54-11.50)	74	13.43(12.25-14.69)	68	1.31(1.29,1.34)	1.12(1.09,1.15)	0.07(-0.17,0.31)	0.84(0.77,0.92)	47
Singapore	6.81(5.86-7.83)	119	8.37(7.14-9.67)	117	0.46(0.40,0.52)	0.93(0.89,0.97)	0.79(0.71,0.87)	0.74(0.70,0.78)	76
Australia	18.30(16.41-20.50)	16	20.56(18.26-23.09)	21	0.97(0.95,1.00)	0.73(0.70,0.76)	-0.58(-0.80,-0.36)	0.40(0.32,0.47)	147
New Zealand	23.30(20.74-26.06)	5	22.59(20.14-25.12)	7	-0.01(-0.16,0.15)	0.07(0.01,0.12)	-0.51(-0.62,-0.39)	-0.13(-0.20,-0.06)	198
Andorra	14.59(12.88-16.72)	35	16.32(14.42-18.62)	43	0.96(0.77,1.15)	0.33(0.27,0.38)	-0.29(-0.42,-0.16)	0.37(0.29,0.45)	154
Austria	12.31(11.06-13.84)	56	13.00(11.60-14.66)	72	0.68(0.55,0.81)	0.43(0.33,0.53)	-0.71(-0.95,-0.48)	0.16(0.06,0.26)	184
Belgium	12.95(11.63-14.54)	52	14.71(13.15-16.64)	57	0.63(0.53,0.74)	0.59(0.54,0.63)	-0.01(-0.10,0.09)	0.42(0.37,0.48)	143
Cyprus	17.02(15.24-19.05)	22	21.63(19.31-24.50)	10	0.87(0.79,0.96)	1.66(1.54,1.77)	-0.12(-0.31,0.07)	0.80(0.72,0.88)	57
Denmark	17.82(16.37-19.58)	18	20.66(18.87-22.97)	19	0.48(0.46,0.50)	0.72(0.71,0.73)	0.33(0.30,0.36)	0.51(0.50,0.53)	125

Finland	24.97(23.04-27.10)	4	27.89(25.50-30.76)	2	0.86(0.83,0.90)	0.86(0.83,0.90)	-0.67(-1.28,-0.04)	0.39(0.19,0.58)	150
France	11.70(10.51-12.99)	61	13.70(12.15-15.43)	66	0.70(0.63,0.78)	0.81(0.75,0.87)	0.02(-0.08,0.12)	0.53(0.48,0.58)	123
Germany	11.10(9.90-12.46)	67	12.18(10.72-13.90)	84	0.80(0.75,0.86)	0.50(0.45,0.55)	-0.47(-0.60,-0.34)	0.30(0.24,0.35)	166
Greece	10.38(9.24-11.64)	75	11.50(10.24-12.91)	96	0.30(0.22,0.39)	0.38(0.34,0.42)	0.47(0.38,0.57)	0.38(0.34,0.43)	153
Iceland	12.30(10.94-13.92)	57	13.33(11.76-15.17)	69	0.67(0.56,0.78)	0.44(0.38,0.50)	-0.38(-0.47,-0.29)	0.26(0.21,0.32)	171
Ireland	25.42(23.17-27.84)	3	30.03(26.97-33.31)	1	1.09(1.06,1.13)	1.09(1.06,1.13)	-0.49(-1.17,0.19)	0.60(0.38,0.81)	102
Israel	9.56(8.36-10.94)	97	11.43(9.99-13.08)	98	0.62(0.54,0.70)	0.72(0.67,0.77)	0.58(0.49,0.67)	0.64(0.59,0.69)	94
Italy	13.96(12.52-15.45)	43	13.81(12.40-15.31)	65	-0.03(-0.05,-0.02)	-0.07(-0.07,-0.06)	-0.01(-0.02,0.00)	-0.03(-0.04,-0.03)	193
Luxembourg	10.88(9.64-12.31)	70	12.33(10.93-13.91)	80	0.89(0.85,0.92)	0.69(0.65,0.74)	-0.27(-0.62,0.07)	0.45(0.33,0.56)	140
Malta	10.36(9.26-11.74)	76	11.62(10.31-13.15)	95	0.77(0.73,0.81)	0.55(0.51,0.59)	-0.12(-0.41,0.17)	0.41(0.32,0.51)	146
Netherlands	21.03(18.95-23.46)	7	20.67(18.57-23.21)	18	0.67(0.59,0.75)	0.67(0.59,0.75)	-1.75(-2.22,-1.28)	-0.09(-0.25,0.07)	197
Norway	22.14(19.80-24.66)	6	21.74(19.41-24.23)	9	0.02(-0.03,0.06)	0.02(-0.03,0.06)	-0.14(-1.02,0.74)	-0.03(-0.31,0.24)	194
Portugal	9.81(8.82-10.91)	89	12.45(11.15-13.83)	77	1.26(1.22,1.30)	0.98(0.93,1.03)	0.10(-0.03,0.22)	0.79(0.74,0.84)	61
Spain	11.23(10.36-12.22)	66	12.88(11.94-13.96)	74	0.87(0.85,0.89)	0.39(0.36,0.42)	0.12(0.08,0.16)	0.47(0.45,0.49)	131
Sweden	20.61(19.07-22.32)	8	21.39(19.69-23.28)	11	0.08(0.03,0.13)	0.13(0.12,0.15)	0.15(0.07,0.23)	0.12(0.09,0.15)	186
Switzerland	16.42(14.62-18.41)	24	17.69(15.72-20.01)	36	0.58(0.52,0.63)	0.37(0.31,0.42)	-0.24(-0.38,-0.09)	0.25(0.18,0.31)	174
United Kingdom	25.50(23.13-28.01)	2	23.59(21.45-25.87)	6	0.02(0.00,0.03)	0.02(0.00,0.03)	-0.83(-1.10,-0.56)	-0.25(-0.33,-0.16)	201
Argentina	9.74(8.65-11.07)	90	11.63(10.40-13.06)	93	1.32(1.19,1.44)	0.54(0.46,0.62)	-0.24(-0.43,-0.05)	0.56(0.48,0.64)	119
Chile	14.58(13.10-16.23)	36	20.96(18.91-23.47)	15	1.65(1.63,1.67)	1.23(1.18,1.29)	0.88(0.85,0.91)	1.26(1.24,1.28)	8
Uruguay	10.15(9.00-11.51)	82	12.72(11.26-14.47)	76	0.97(0.89,1.06)	0.66(0.64,0.69)	0.69(0.60,0.78)	0.79(0.75,0.83)	62
Canada	17.50(16.81-18.17)	19	22.58(21.72-23.39)	8	1.61(1.56,1.65)	0.62(0.59,0.66)	0.43(0.40,0.46)	0.87(0.85,0.90)	41
United States of America	18.19(16.97-19.58)	17	21.32(19.78-23.12)	12	0.48(0.43,0.52)	0.96(0.92,1.00)	0.14(0.11,0.18)	0.56(0.54,0.59)	120
Antigua and Barbuda	9.70(8.59-11.01)	92	12.04(10.69-13.63)	85	0.94(0.93,0.96)	0.87(0.83,0.92)	0.50(0.46,0.55)	0.76(0.74,0.78)	72
Bahamas	9.63(8.49-11.06)	95	11.31(9.95-12.93)	100	0.95(0.88,1.03)	0.57(0.51,0.63)	0.09(0.06,0.12)	0.55(0.51,0.59)	122
Barbados	12.38(11.14-13.89)	54	15.05(13.39-17.04)	52	1.05(1.01,1.10)	0.77(0.74,0.80)	0.15(0.11,0.20)	0.66(0.64,0.69)	90
Belize	9.31(8.29-10.49)	100	11.72(10.31-13.34)	90	0.86(0.67,1.05)	1.10(0.91,1.28)	0.42(0.37,0.48)	0.77(0.68,0.86)	67
Cuba	9.65(8.39-11.06)	94	12.28(10.78-13.98)	82	0.97(0.93,1.00)	0.91(0.88,0.94)	0.62(0.59,0.65)	0.83(0.81,0.85)	49
Dominica	10.02(8.89-11.28)	86	11.13(9.94-12.60)	101	0.73(0.69,0.76)	0.35(0.27,0.44)	-0.01(-0.03,0.00)	0.36(0.33,0.39)	156
Dominican Republic	6.32(5.43-7.30)	122	8.01(6.87-9.41)	123	1.06(1.03,1.09)	0.94(0.90,0.98)	0.45(0.36,0.55)	0.83(0.79,0.86)	50
Grenada	6.43(5.56-7.47)	121	8.09(7.01-9.33)	121	1.11(1.07,1.15)	0.63(0.56,0.70)	0.74(0.65,0.83)	0.82(0.78,0.86)	53
Guyana	6.12(5.29-7.08)	126	7.71(6.71-8.87)	125	0.84(0.83,0.85)	0.44(0.43,0.44)	1.16(1.10,1.23)	0.80(0.78,0.82)	58
Haiti	8.88(7.93-9.97)	104	10.95(9.70-12.32)	102	0.90(0.86,0.93)	0.72(0.58,0.87)	0.54(0.32,0.77)	0.72(0.62,0.81)	81
Jamaica	7.39(6.40-8.47)	113	9.12(8.00-10.53)	113	0.84(0.79,0.89)	0.92(0.81,1.02)	0.53(0.44,0.61)	0.74(0.70,0.79)	77
Saint Lucia	10.22(9.16-11.34)	79	12.73(11.39-14.31)	75	1.29(1.27,1.31)	0.73(0.71,0.74)	0.26(0.21,0.32)	0.76(0.74,0.78)	73
Saint Vincent and the Grenadines	7.01(6.13-8.03)	117	8.41(7.35-9.70)	116	0.53(0.47,0.59)	0.79(0.75,0.83)	0.62(0.54,0.70)	0.64(0.60,0.68)	95
Suriname	6.17(5.30-7.18)	125	7.68(6.65-8.95)	126	0.36(0.25,0.47)	0.81(0.76,0.86)	1.20(1.06,1.34)	0.79(0.73,0.85)	63
Trinidad and Tobago	12.24(11.02-13.67)	58	14.68(13.18-16.48)	58	0.56(0.51,0.62)	1.05(1.00,1.10)	0.19(0.10,0.28)	0.62(0.57,0.66)	96
Bolivia (Plurinational State of)	10.24(9.18-11.54)	78	14.38(12.91-16.22)	60	1.24(1.17,1.31)	1.39(1.36,1.41)	0.97(0.88,1.07)	1.19(1.15,1.23)	16

Ecuador	12.34(11.13-13.78)	55	18.00(16.19-20.23)	30	2.13(2.07,2.19)	0.98(0.86,1.10)	0.86(0.81,0.91)	1.31(1.26,1.36)	5
Peru	8.89(7.86-10.20)	103	13.29(11.71-15.23)	70	1.39(1.25,1.54)	1.52(1.47,1.57)	1.30(1.27,1.34)	1.43(1.37,1.49)	1
Colombia	11.08(9.85-12.41)	68	14.04(12.55-15.76)	63	1.36(1.15,1.57)	1.04(0.94,1.13)	-0.15(-0.42,0.11)	0.77(0.65,0.88)	68
Costa Rica	16.18(14.44-18.13)	26	21.07(18.86-23.48)	13	0.90(0.81,0.99)	0.96(0.91,1.00)	0.94(0.86,1.03)	0.93(0.89,0.98)	35
El Salvador	8.73(7.62-9.97)	105	11.89(10.42-13.66)	87	1.48(1.34,1.62)	1.03(0.89,1.16)	0.68(0.64,0.72)	1.09(1.02,1.16)	18
Guatemala	10.11(9.01-11.46)	84	14.95(13.31-16.83)	55	2.04(1.99,2.09)	1.20(1.14,1.27)	0.96(0.85,1.06)	1.39(1.35,1.43)	2
Honduras	18.82(17.03-20.88)	14	25.06(22.49-27.68)	5	1.26(1.17,1.34)	1.01(0.99,1.03)	0.64(0.57,0.71)	0.97(0.94,1.01)	28
Mexico	27.40(24.69-30.19)	1	25.43(22.94-28.02)	4	-0.21(-0.25,-0.16)	-0.65(-1.02,-0.29)	-0.05(-0.15,0.04)	-0.29(-0.41,-0.17)	202
Nicaragua	11.50(10.18-12.94)	63	15.36(13.61-17.28)	48	0.71(0.61,0.80)	1.40(1.34,1.46)	0.79(0.71,0.88)	1.00(0.95,1.05)	24
Panama	11.61(10.37-13.04)	62	14.33(12.74-16.24)	61	0.94(0.65,1.22)	0.45(0.36,0.55)	0.86(0.81,0.91)	0.74(0.64,0.83)	78
Venezuela (Bolivarian Republic of)	17.14(15.96-18.37)	21	21.02(19.64-22.31)	14	1.02(0.94,1.10)	0.49(0.42,0.57)	0.56(0.45,0.66)	0.69(0.63,0.74)	88
Brazil	11.29(10.11-12.63)	65	11.98(10.71-13.39)	86	0.09(0.01,0.17)	0.14(0.10,0.18)	0.43(0.38,0.49)	0.22(0.18,0.26)	178
Paraguay	9.58(8.39-10.94)	96	13.50(11.78-15.29)	67	1.51(1.37,1.65)	0.71(0.64,0.77)	1.50(1.37,1.62)	1.20(1.13,1.28)	14
Algeria	3.99(3.44-4.61)	179	5.24(4.55-6.05)	147	0.34(0.31,0.38)	0.96(0.95,0.97)	1.57(1.43,1.70)	0.96(0.91,1.00)	30
Bahrain	5.73(5.05-6.54)	132	8.06(7.08-9.21)	122	1.27(0.84,1.71)	1.01(0.95,1.06)	1.36(1.18,1.54)	1.20(1.05,1.35)	15
Egypt	3.75(3.20-4.41)	190	4.91(4.20-5.70)	162	0.87(0.70,1.05)	0.59(0.54,0.64)	1.41(1.30,1.52)	0.96(0.88,1.03)	31
Iran (Islamic Republic of)	4.68(4.18-5.24)	141	5.04(4.47-5.64)	153	0.16(0.12,0.21)	0.00(-0.03,0.03)	0.65(0.43,0.88)	0.26(0.19,0.33)	172
Iraq	4.01(3.46-4.68)	176	5.03(4.34-5.80)	154	-0.26(-0.43,-0.10)	1.23(1.19,1.27)	1.45(1.32,1.57)	0.80(0.73,0.87)	59
Jordan	3.76(3.22-4.38)	188	5.04(4.33-5.90)	152	0.53(0.41,0.65)	1.28(1.26,1.30)	1.28(1.26,1.30)	1.05(1.00,1.09)	21
Kuwait	4.29(3.65-5.05)	152	5.70(4.87-6.66)	139	0.07(-0.11,0.26)	0.99(0.84,1.14)	1.85(1.80,1.91)	1.00(0.91,1.08)	25
Lebanon	3.90(3.34-4.58)	186	5.41(4.58-6.31)	144	0.66(0.54,0.78)	1.37(1.31,1.43)	1.43(1.15,1.70)	1.17(1.07,1.26)	17
Libya	3.71(3.15-4.38)	193	4.65(3.92-5.47)	180	0.55(0.48,0.62)	1.18(1.06,1.29)	0.71(0.43,1.00)	0.82(0.72,0.92)	54
Morocco	3.45(2.96-4.00)	199	4.51(3.88-5.28)	189	0.45(0.40,0.51)	0.91(0.89,0.92)	1.45(1.28,1.62)	0.93(0.88,0.99)	36
Palestine	4.06(3.51-4.70)	168	4.97(4.30-5.72)	160	0.41(0.36,0.46)	0.49(0.46,0.51)	1.22(1.18,1.27)	0.70(0.68,0.73)	85
Oman	3.41(2.93-3.97)	200	4.67(4.00-5.43)	179	1.49(1.22,1.77)	1.00(0.89,1.11)	0.89(0.69,1.09)	1.09(0.97,1.22)	19
Qatar	3.98(3.41-4.62)	181	5.30(4.54-6.18)	145	1.04(0.93,1.16)	0.75(0.66,0.84)	1.29(1.19,1.40)	1.02(0.95,1.08)	22
Saudi Arabia	3.50(2.95-4.13)	198	4.97(4.21-5.84)	159	0.90(0.79,1.02)	1.11(1.09,1.14)	1.71(1.40,2.02)	1.23(1.13,1.33)	9
Syrian Arab Republic	3.76(3.22-4.39)	189	5.00(4.25-5.87)	157	0.73(0.51,0.96)	0.83(0.72,0.95)	1.46(1.34,1.58)	1.02(0.93,1.12)	23
Tunisia	3.77(3.21-4.38)	187	5.01(4.26-5.83)	156	0.51(0.44,0.58)	1.05(1.04,1.07)	1.41(1.18,1.63)	0.99(0.92,1.07)	27
Turkey	7.93(7.14-8.81)	110	11.35(10.27-12.51)	99	1.02(0.92,1.13)	1.88(1.80,1.96)	0.69(0.57,0.82)	1.23(1.17,1.30)	10
United Arab Emirates	3.73(3.20-4.33)	191	4.80(4.13-5.57)	172	0.38(0.24,0.52)	0.31(0.10,0.51)	2.05(1.93,2.18)	0.86(0.76,0.96)	42
Yemen	3.15(2.71-3.68)	204	3.75(3.23-4.37)	203	0.21(0.16,0.26)	0.84(0.82,0.86)	0.72(0.64,0.80)	0.61(0.57,0.64)	98
Afghanistan	3.93(3.43-4.51)	185	4.40(3.85-5.07)	193	-0.46(-0.52,-0.40)	0.69(0.67,0.72)	0.89(0.88,0.90)	0.39(0.37,0.41)	151
Bangladesh	14.66(13.17-16.34)	34	20.95(18.66-23.49)	16	0.60(0.51,0.70)	1.60(1.54,1.67)	1.38(1.29,1.48)	1.22(1.16,1.27)	11
Bhutan	13.96(12.60-15.53)	42	20.60(18.47-22.93)	20	0.91(0.79,1.02)	1.80(1.76,1.84)	1.21(1.11,1.30)	1.34(1.29,1.39)	3
India	16.48(14.87-18.21)	23	17.40(15.70-19.20)	38	0.26(0.22,0.29)	0.14(0.12,0.16)	0.14(0.06,0.21)	0.18(0.15,0.20)	183
Nepal	14.23(12.86-15.83)	41	20.53(18.53-22.75)	22	1.15(1.07,1.23)	1.28(1.17,1.39)	1.38(1.33,1.43)	1.28(1.23,1.33)	6
Pakistan	20.10(18.25-22.19)	10	20.36(18.41-22.55)	25	-0.26(-0.39,-0.13)	0.09(0.05,0.14)	0.34(0.24,0.45)	0.06(0.00,0.12)	189

Angola	9.35(8.34-10.49)	99	11.68(10.37-13.19)	91	0.22(0.14,0.29)	1.24(1.21,1.27)	0.76(0.46,1.06)	0.77(0.68,0.87)	69
Central African Republic	9.70(8.61-10.91)	91	9.85(8.79-11.00)	108	-0.14(-0.23,-0.05)	0.38(0.35,0.40)	-0.12(-0.41,0.17)	0.06(-0.03,0.16)	190
Congo	9.89(8.83-11.10)	87	11.66(10.34-13.09)	92	-0.17(-0.29,-0.06)	1.48(1.23,1.74)	0.47(0.28,0.66)	0.58(0.47,0.69)	111
Democratic Republic of the Congo	9.03(7.95-10.19)	102	10.09(8.93-11.47)	106	-0.04(-0.18,0.09)	0.53(0.50,0.56)	0.53(0.50,0.56)	0.35(0.30,0.40)	157
Equatorial Guinea	8.31(7.41-9.41)	108	12.33(10.92-13.97)	79	1.09(0.88,1.31)	2.41(2.26,2.57)	0.30(0.08,0.52)	1.34(1.21,1.47)	4
Gabon	9.87(8.78-11.04)	88	12.29(10.93-13.92)	81	0.34(0.27,0.40)	1.13(1.09,1.18)	0.86(0.53,1.20)	0.77(0.67,0.88)	70
Burundi	13.16(11.87-14.63)	50	15.27(13.74-16.95)	49	0.36(0.23,0.48)	1.20(1.10,1.30)	-0.11(-0.27,0.05)	0.51(0.42,0.59)	126
Comoros	14.50(13.05-16.16)	37	17.79(15.86-19.78)	33	0.29(0.18,0.39)	1.35(1.25,1.45)	0.35(0.27,0.43)	0.70(0.64,0.76)	86
Djibouti	15.74(14.10-17.57)	27	19.11(17.11-21.32)	27	-0.36(-0.58,-0.13)	1.36(1.30,1.42)	0.83(0.72,0.93)	0.66(0.58,0.74)	91
Eritrea	13.76(12.50-15.29)	45	17.44(15.51-19.50)	37	0.75(0.63,0.86)	0.80(0.55,1.05)	0.89(0.79,0.98)	0.83(0.73,0.92)	51
Ethiopia	14.28(12.81-15.82)	40	15.19(13.61-16.84)	50	0.00(-0.11,0.11)	0.41(0.36,0.45)	0.12(0.02,0.22)	0.20(0.14,0.25)	180
Kenya	18.34(16.47-20.32)	15	17.93(16.06-19.92)	31	-0.16(-0.19,-0.14)	-0.03(-0.04,-0.03)	-0.06(-0.13,0.02)	-0.08(-0.11,-0.05)	195
Madagascar	13.43(12.07-14.91)	48	15.15(13.60-16.81)	51	0.36(0.27,0.46)	0.60(0.51,0.70)	0.21(0.12,0.31)	0.40(0.34,0.46)	148
Malawi	15.13(13.63-16.90)	31	17.74(16.04-19.62)	35	0.16(0.10,0.23)	0.84(0.81,0.86)	0.65(0.53,0.76)	0.56(0.52,0.61)	121
Mauritius	6.17(5.35-7.24)	124	7.33(6.39-8.54)	129	0.93(0.85,1.01)	0.24(0.18,0.30)	0.65(0.55,0.75)	0.58(0.53,0.63)	112
Mozambique	14.39(13.03-16.00)	39	17.74(15.91-19.78)	34	0.67(0.59,0.75)	0.89(0.84,0.94)	0.52(0.44,0.59)	0.71(0.66,0.75)	83
Rwanda	14.42(12.95-16.03)	38	18.91(16.83-21.03)	29	-0.12(-0.51,0.28)	1.91(1.81,2.01)	0.80(0.64,0.95)	0.93(0.79,1.07)	37
Seychelles	3.99(3.41-4.66)	178	4.53(3.87-5.28)	188	0.26(0.16,0.35)	0.38(0.33,0.43)	0.76(0.66,0.85)	0.46(0.41,0.51)	136
Somalia	19.31(17.23-21.50)	11	20.45(18.17-22.99)	24	-0.22(-0.34,-0.09)	0.54(0.50,0.57)	0.19(0.06,0.32)	0.19(0.14,0.25)	182
United Republic of Tanzania	15.17(13.49-16.93)	30	17.86(15.93-19.93)	32	0.28(0.21,0.34)	0.91(0.86,0.96)	0.52(0.40,0.64)	0.57(0.52,0.62)	116
Uganda	13.55(12.14-15.04)	47	16.15(14.48-17.99)	45	-0.14(-0.34,0.07)	1.20(1.00,1.40)	0.68(0.62,0.74)	0.58(0.48,0.68)	113
Zambia	16.30(14.72-18.07)	25	20.81(18.90-22.98)	17	0.00(-0.23,0.22)	1.24(1.19,1.28)	1.24(1.19,1.28)	0.85(0.77,0.93)	45
Botswana	12.96(11.59-14.58)	51	16.39(14.45-18.36)	42	0.93(0.78,1.08)	0.54(0.35,0.72)	0.75(0.53,0.96)	0.75(0.63,0.86)	74
Lesotho	12.93(11.60-14.42)	53	14.10(12.58-15.75)	62	0.02(-0.17,0.21)	0.12(0.06,0.18)	0.78(0.72,0.85)	0.30(0.22,0.37)	167
Namibia	13.62(12.17-15.33)	46	17.16(15.28-19.13)	39	0.31(0.14,0.48)	1.00(0.93,1.07)	0.91(0.75,1.07)	0.77(0.69,0.86)	71
South Africa	20.24(18.16-22.56)	9	19.29(17.25-21.47)	26	-0.34(-0.39,-0.29)	0.22(0.19,0.25)	-0.36(-0.59,-0.13)	-0.15(-0.23,-0.08)	199
Eswatini	14.89(13.33-16.68)	32	16.51(14.82-18.31)	41	0.09(-0.05,0.23)	-0.04(-0.11,0.02)	0.90(0.72,1.07)	0.32(0.24,0.40)	162
Zimbabwe	7.21(6.34-8.28)	115	7.18(6.32-8.20)	130	-0.66(-0.75,-0.58)	-0.64(-0.71,-0.57)	1.42(1.36,1.49)	-0.01(-0.06,0.04)	192
Benin	4.15(3.63-4.74)	159	4.83(4.24-5.53)	171	0.38(0.34,0.41)	0.67(0.63,0.71)	0.49(0.44,0.55)	0.52(0.49,0.54)	124
Burkina Faso	3.97(3.46-4.54)	183	4.59(4.02-5.24)	182	0.57(0.52,0.61)	0.65(0.60,0.70)	0.28(0.22,0.35)	0.51(0.47,0.54)	127
Cameroon	4.50(3.90-5.18)	146	5.29(4.64-6.07)	146	0.02(-0.05,0.10)	0.72(0.61,0.83)	0.96(0.88,1.05)	0.58(0.52,0.63)	114
Cabo Verde	3.36(2.90-3.90)	201	4.19(3.63-4.84)	198	0.41(0.36,0.46)	0.87(0.85,0.89)	1.04(0.94,1.13)	0.78(0.75,0.81)	64
Chad	3.98(3.48-4.55)	180	4.50(3.94-5.10)	190	0.05(-0.03,0.12)	0.55(0.53,0.58)	0.74(0.70,0.79)	0.43(0.40,0.46)	141
C??te d'Ivoire	4.32(3.76-4.95)	151	4.91(4.27-5.60)	163	0.15(0.09,0.20)	0.62(0.58,0.67)	0.57(0.45,0.69)	0.46(0.41,0.50)	137
Gambia	4.15(3.61-4.74)	160	4.75(4.12-5.46)	177	0.32(0.30,0.34)	0.49(0.40,0.58)	0.62(0.59,0.66)	0.46(0.43,0.49)	138
Ghana	4.05(3.50-4.72)	170	5.10(4.38-5.94)	149	0.77(0.73,0.81)	0.54(0.50,0.59)	1.20(1.06,1.34)	0.82(0.76,0.87)	55
Guinea	4.15(3.63-4.72)	161	4.77(4.17-5.51)	174	0.28(0.26,0.30)	0.64(0.58,0.69)	0.59(0.55,0.62)	0.49(0.46,0.51)	130
Guinea-Bissau	4.07(3.55-4.67)	167	4.56(4.00-5.18)	187	0.06(0.05,0.07)	0.60(0.58,0.61)	0.55(0.52,0.58)	0.40(0.39,0.41)	149

Liberia	4.12(3.61-4.71)	163	4.87(4.24-5.58)	166	0.12(0.07,0.17)	1.11(1.05,1.18)	0.45(0.36,0.54)	0.58(0.54,0.62)	115
Mali	4.04(3.52-4.61)	172	4.88(4.26-5.60)	164	0.76(0.72,0.80)	0.79(0.68,0.90)	0.48(0.42,0.55)	0.66(0.62,0.70)	92
Mauritania	4.32(3.76-4.94)	150	5.50(4.78-6.30)	142	0.81(0.80,0.83)	0.77(0.76,0.79)	0.93(0.92,0.95)	0.84(0.83,0.85)	48
Niger	3.95(3.45-4.50)	184	4.57(4.00-5.22)	186	0.35(0.31,0.39)	0.80(0.76,0.84)	0.34(0.27,0.41)	0.50(0.47,0.53)	128
Nigeria	3.66(3.27-4.09)	195	4.35(3.88-4.86)	194	0.52(0.50,0.53)	0.43(0.41,0.44)	0.98(0.82,1.14)	0.61(0.55,0.66)	99
Sao Tome and Principe	3.97(3.43-4.55)	182	4.88(4.21-5.63)	165	0.44(0.34,0.53)	0.71(0.67,0.75)	0.99(0.89,1.09)	0.71(0.66,0.76)	84
Senegal	4.24(3.70-4.88)	156	4.84(4.26-5.57)	169	0.31(0.27,0.35)	0.50(0.46,0.54)	0.60(0.54,0.65)	0.47(0.44,0.49)	132
Sierra Leone	4.01(3.50-4.56)	177	4.58(3.95-5.24)	184	0.07(0.03,0.11)	0.43(0.36,0.50)	0.91(0.87,0.96)	0.47(0.43,0.50)	133
Togo	4.13(3.60-4.74)	162	4.57(3.95-5.23)	185	-0.12(-0.19,-0.05)	0.43(0.35,0.51)	0.79(0.70,0.88)	0.37(0.32,0.42)	155
American Samoa	4.55(3.91-5.30)	143	4.87(4.18-5.64)	167	0.71(0.65,0.77)	-0.07(-0.29,0.15)	0.19(0.11,0.27)	0.26(0.18,0.34)	173
Bermuda	9.44(8.30-10.77)	98	12.27(10.66-14.14)	83	1.31(1.23,1.39)	1.04(0.98,1.09)	0.42(0.37,0.46)	0.93(0.89,0.97)	38
Cook Islands	4.24(3.62-4.96)	155	5.05(4.31-5.92)	151	0.60(0.49,0.72)	0.45(0.42,0.49)	0.80(0.73,0.88)	0.62(0.57,0.67)	97
Greenland	10.57(9.49-11.83)	72	15.04(13.63-16.78)	53	1.58(1.52,1.63)	0.92(0.89,0.95)	1.19(0.97,1.42)	1.21(1.14,1.28)	12
Guam	4.53(3.84-5.36)	144	4.85(4.11-5.71)	168	0.52(0.41,0.62)	-0.11(-0.14,-0.09)	0.35(0.27,0.43)	0.27(0.23,0.32)	170
Monaco	8.54(7.40-9.80)	106	9.44(8.13-10.90)	112	0.04(-0.11,0.18)	0.38(0.30,0.46)	0.75(0.60,0.90)	0.39(0.31,0.47)	152
Nauru	4.07(3.48-4.75)	166	4.49(3.87-5.20)	191	-0.18(-0.28,-0.09)	-0.18(-0.29,-0.07)	1.53(1.44,1.61)	0.35(0.28,0.41)	158
Niue	4.23(3.63-4.93)	157	4.99(4.29-5.77)	158	0.61(0.43,0.79)	0.50(0.47,0.54)	0.67(0.57,0.77)	0.59(0.52,0.65)	105
Northern Mariana Islands	4.61(3.96-5.37)	142	5.06(4.35-5.89)	150	1.32(1.22,1.41)	-0.32(-0.45,-0.18)	-0.03(-0.25,0.18)	0.31(0.22,0.40)	165
Palau	4.22(3.63-4.93)	158	4.58(3.90-5.33)	183	0.43(0.26,0.61)	0.23(0.19,0.27)	0.23(-0.04,0.50)	0.29(0.19,0.39)	169
Puerto Rico	10.69(9.35-12.50)	71	14.03(12.26-16.29)	64	1.04(0.99,1.09)	1.20(1.17,1.22)	0.59(0.50,0.69)	0.95(0.92,0.99)	33
Saint Kitts and Nevis	10.16(8.95-11.62)	81	12.38(10.89-14.18)	78	1.13(1.07,1.20)	0.65(0.56,0.73)	0.20(0.11,0.29)	0.66(0.61,0.71)	93
San Marino	10.52(8.82-12.40)	73	11.87(9.95-13.98)	88	0.03(-0.22,0.29)	0.52(0.43,0.62)	0.89(0.61,1.17)	0.47(0.33,0.61)	134
Tokelau	3.65(3.14-4.25)	196	4.29(3.68-4.98)	196	0.44(0.18,0.70)	0.39(0.31,0.47)	0.88(0.70,1.06)	0.57(0.46,0.69)	117
Tuvalu	3.71(3.20-4.34)	192	4.07(3.49-4.76)	199	0.50(0.47,0.53)	0.01(-0.01,0.03)	0.50(0.45,0.55)	0.33(0.31,0.35)	161
United States Virgin Islands	8.29(7.21-9.63)	109	10.37(8.94-12.09)	105	1.10(1.01,1.19)	0.93(0.77,1.08)	0.30(0.25,0.35)	0.78(0.71,0.84)	65
South Sudan	15.20(13.55-16.98)	29	16.20(14.50-18.15)	44	-0.10(-0.30,0.10)	0.56(0.51,0.60)	0.10(0.08,0.13)	0.21(0.14,0.27)	179
Sudan	3.20(2.76-3.72)	203	4.20(3.63-4.84)	197	0.36(0.28,0.43)	0.84(0.80,0.87)	1.75(1.69,1.82)	0.97(0.93,1.01)	29
Psoriasis									
China	68.25(65.84-70.70)	76	53.96(52.08-55.79)	84	-0.79(-0.81,-0.77)	-0.78(-0.84,-0.71)	-0.81(-0.84,-0.78)	-0.80(-0.82,-0.77)	182
Democratic People's Republic of Korea	78.63(75.46-81.89)	42	65.62(63.03-68.17)	42	-0.52(-0.55,-0.49)	-0.65(-0.66,-0.64)	-0.69(-0.71,-0.67)	-0.62(-0.63,-0.61)	141
Taiwan (Province of China)	63.63(61.06-66.31)	91	50.17(48.18-52.02)	99	-0.92(-0.96,-0.87)	-0.74(-0.77,-0.71)	-0.84(-0.91,-0.77)	-0.82(-0.85,-0.79)	185
Cambodia	31.31(30.05-32.54)	173	26.61(25.56-27.67)	172	-0.53(-0.59,-0.47)	-0.66(-0.70,-0.62)	-0.53(-0.56,-0.50)	-0.57(-0.59,-0.54)	125
Indonesia	14.32(13.76-14.84)	204	12.92(12.42-13.38)	204	-0.47(-0.66,-0.29)	-0.43(-0.85,-0.01)	-0.19(-0.28,-0.10)	-0.35(-0.50,-0.20)	53
Lao People's Democratic Republic	32.21(30.88-33.49)	169	27.20(26.15-28.17)	170	-0.49(-0.53,-0.46)	-0.63(-0.64,-0.62)	-0.64(-0.69,-0.59)	-0.58(-0.60,-0.56)	130
Malaysia	23.39(22.46-24.25)	198	19.73(18.93-20.50)	200	-0.82(-0.85,-0.80)	-0.56(-0.66,-0.45)	-0.40(-0.43,-0.37)	-0.59(-0.63,-0.56)	134
Maldives	28.36(27.30-29.48)	179	22.91(21.97-23.84)	189	-1.11(-1.12,-1.09)	-0.67(-0.68,-0.65)	-0.45(-0.49,-0.40)	-0.73(-0.74,-0.72)	172
Myanmar	31.75(30.57-33.00)	170	26.46(25.45-27.42)	174	-0.43(-0.45,-0.42)	-0.86(-0.88,-0.84)	-0.60(-0.65,-0.56)	-0.63(-0.64,-0.61)	145
Philippines	26.62(25.68-27.54)	190	24.32(23.45-25.18)	182	-0.42(-0.47,-0.37)	0.14(0.10,0.18)	-0.72(-0.84,-0.59)	-0.30(-0.34,-0.26)	43

Sri Lanka	42.54(40.75-44.43)	127	34.90(33.40-36.41)	132	-0.70(-0.73,-0.67)	-0.52(-0.54,-0.51)	-0.81(-0.86,-0.75)	-0.68(-0.70,-0.66)	164
Thailand	25.66(24.59-26.69)	193	22.29(21.38-23.16)	192	-0.66(-0.68,-0.64)	-0.39(-0.40,-0.38)	-0.40(-0.48,-0.32)	-0.48(-0.50,-0.45)	101
Timor-Leste	30.72(29.52-31.95)	174	25.48(24.46-26.45)	179	-0.07(-0.12,-0.01)	-1.47(-1.56,-1.38)	-0.40(-0.56,-0.24)	-0.63(-0.69,-0.56)	146
Viet Nam	28.97(27.81-30.15)	177	24.01(23.05-24.90)	185	-0.77(-0.81,-0.72)	-0.65(-0.66,-0.64)	-0.55(-0.57,-0.53)	-0.65(-0.67,-0.64)	153
Fiji	39.16(37.58-40.89)	138	34.22(32.82-35.65)	137	-0.60(-0.61,-0.59)	-0.34(-0.35,-0.33)	-0.46(-0.51,-0.41)	-0.46(-0.48,-0.45)	95
Kiribati	39.24(37.71-40.75)	137	37.03(35.47-38.47)	126	-0.34(-0.40,-0.29)	0.03(0.00,0.06)	-0.34(-0.38,-0.29)	-0.20(-0.23,-0.18)	17
Marshall Islands	41.86(40.13-43.63)	128	35.74(34.30-37.22)	129	-0.64(-0.66,-0.61)	-0.46(-0.47,-0.45)	-0.53(-0.58,-0.49)	-0.54(-0.56,-0.52)	117
Micronesia (Federated States of)	39.48(37.85-41.09)	134	34.52(33.10-35.88)	136	-0.58(-0.61,-0.54)	-0.43(-0.44,-0.42)	-0.39(-0.43,-0.35)	-0.46(-0.48,-0.44)	96
Papua New Guinea	44.77(42.94-46.62)	121	40.26(38.65-41.89)	120	-0.37(-0.40,-0.34)	-0.23(-0.24,-0.22)	-0.49(-0.54,-0.45)	-0.37(-0.38,-0.35)	58
Samoa	38.21(36.69-39.82)	142	36.22(34.78-37.72)	127	-0.07(-0.08,-0.06)	-0.07(-0.08,-0.06)	-0.43(-0.47,-0.38)	-0.18(-0.20,-0.17)	13
Solomon Islands	45.75(43.89-47.63)	119	40.32(38.73-42.01)	119	-0.53(-0.57,-0.50)	-0.22(-0.24,-0.20)	-0.54(-0.62,-0.46)	-0.43(-0.46,-0.40)	82
Tonga	40.31(38.65-41.96)	132	35.65(34.12-37.07)	130	-0.54(-0.57,-0.51)	-0.21(-0.23,-0.20)	-0.54(-0.61,-0.47)	-0.42(-0.44,-0.39)	75
Vanuatu	44.26(42.40-46.12)	123	39.22(37.62-40.79)	122	-0.47(-0.48,-0.45)	-0.37(-0.38,-0.36)	-0.41(-0.43,-0.38)	-0.41(-0.42,-0.40)	72
Armenia	72.91(69.75-76.01)	56	59.08(56.49-61.61)	59	-0.63(-0.64,-0.61)	-1.09(-1.13,-1.06)	-0.36(-0.42,-0.30)	-0.73(-0.75,-0.70)	173
Azerbaijan	70.13(67.19-73.03)	68	59.72(57.17-62.30)	56	-0.15(-0.18,-0.12)	-0.98(-1.14,-0.81)	-0.36(-0.40,-0.31)	-0.53(-0.59,-0.47)	112
Georgia	67.69(64.70-70.62)	79	59.71(56.98-62.39)	57	-0.10(-0.17,-0.03)	-0.62(-0.66,-0.58)	-0.56(-0.60,-0.52)	-0.42(-0.45,-0.39)	76
Kazakhstan	73.10(70.03-76.27)	53	60.82(58.18-63.48)	51	-0.61(-0.66,-0.56)	-0.79(-0.84,-0.75)	-0.41(-0.44,-0.37)	-0.62(-0.65,-0.59)	142
Kyrgyzstan	75.07(71.80-78.34)	49	65.51(62.69-68.44)	43	-0.41(-0.49,-0.34)	-0.58(-0.69,-0.47)	-0.38(-0.42,-0.34)	-0.45(-0.49,-0.40)	91
Mongolia	79.06(75.73-82.49)	41	64.79(62.10-67.41)	45	-0.70(-0.74,-0.66)	-0.83(-0.87,-0.79)	-0.45(-0.47,-0.42)	-0.67(-0.70,-0.65)	159
Tajikistan	77.86(74.53-81.29)	44	66.70(64.02-69.41)	40	-0.25(-0.30,-0.20)	-0.81(-0.84,-0.77)	-0.59(-0.61,-0.57)	-0.53(-0.55,-0.51)	113
Turkmenistan	72.63(69.56-75.68)	57	58.52(56.03-61.07)	63	-0.68(-0.72,-0.63)	-0.83(-0.92,-0.75)	-0.73(-0.85,-0.61)	-0.76(-0.82,-0.70)	176
Uzbekistan	77.46(74.17-80.71)	45	62.94(60.23-65.55)	46	-0.66(-0.69,-0.64)	-0.84(-0.91,-0.76)	-0.59(-0.62,-0.56)	-0.71(-0.74,-0.69)	170
Albania	74.94(72.10-77.72)	50	60.91(58.67-63.18)	48	-0.52(-0.57,-0.48)	-1.11(-1.16,-1.06)	-0.38(-0.42,-0.35)	-0.70(-0.73,-0.68)	169
Bosnia and Herzegovina	76.32(73.57-79.17)	47	60.12(57.89-62.42)	55	-0.74(-0.86,-0.62)	-1.17(-1.22,-1.13)	-0.46(-0.56,-0.36)	-0.82(-0.87,-0.76)	186
Bulgaria	70.45(67.78-73.06)	67	58.11(55.82-60.29)	65	-0.67(-0.75,-0.58)	-0.91(-0.97,-0.85)	-0.33(-0.38,-0.29)	-0.65(-0.69,-0.61)	154
Croatia	69.00(66.50-71.73)	73	57.50(55.34-59.50)	70	-0.52(-0.57,-0.47)	-0.92(-0.98,-0.86)	-0.32(-0.36,-0.28)	-0.61(-0.65,-0.58)	139
Czechia	69.88(67.24-72.49)	69	57.23(55.07-59.25)	72	-0.89(-0.96,-0.81)	-0.84(-0.92,-0.77)	-0.23(-0.27,-0.18)	-0.67(-0.71,-0.63)	160
Hungary	70.71(67.93-73.24)	65	58.06(55.95-60.30)	66	-0.77(-0.83,-0.70)	-0.90(-0.97,-0.82)	-0.24(-0.29,-0.19)	-0.66(-0.69,-0.62)	157
North Macedonia	71.00(68.39-73.55)	64	58.56(56.37-60.71)	62	-0.54(-0.57,-0.51)	-1.00(-1.10,-0.90)	-0.37(-0.41,-0.33)	-0.66(-0.69,-0.62)	158
Montenegro	67.25(64.71-69.65)	80	56.85(54.75-58.93)	75	-0.25(-0.29,-0.20)	-1.03(-1.15,-0.92)	-0.39(-0.44,-0.34)	-0.58(-0.62,-0.53)	131
Poland	73.46(70.74-76.22)	52	65.78(64.06-67.44)	41	-0.49(-0.52,-0.47)	-0.22(-0.28,-0.16)	-0.36(-0.38,-0.33)	-0.38(-0.40,-0.35)	62
Romania	68.55(66.08-71.07)	75	55.82(53.80-57.82)	79	-0.60(-0.71,-0.49)	-1.03(-1.12,-0.95)	-0.35(-0.42,-0.29)	-0.68(-0.74,-0.63)	165
Serbia	71.32(68.70-73.97)	61	58.33(56.20-60.41)	64	-0.55(-0.57,-0.53)	-0.98(-1.03,-0.92)	-0.45(-0.60,-0.30)	-0.68(-0.73,-0.63)	166
Slovakia	71.41(68.82-73.99)	60	57.51(55.38-59.67)	69	-0.88(-0.93,-0.83)	-0.93(-1.01,-0.85)	-0.29(-0.34,-0.24)	-0.72(-0.76,-0.68)	171
Slovenia	67.17(64.63-69.60)	81	55.86(53.76-57.87)	78	-0.65(-0.66,-0.63)	-0.88(-0.93,-0.82)	-0.27(-0.37,-0.17)	-0.62(-0.66,-0.58)	143
Belarus	73.03(70.29-75.73)	54	60.86(58.59-63.25)	49	-0.53(-0.60,-0.47)	-0.85(-0.90,-0.81)	-0.45(-0.49,-0.42)	-0.63(-0.66,-0.60)	147
Estonia	70.59(67.86-73.28)	66	58.83(56.64-60.87)	60	-0.69(-0.74,-0.64)	-0.79(-0.84,-0.74)	-0.35(-0.38,-0.32)	-0.63(-0.65,-0.60)	148
Latvia	69.73(67.18-72.28)	70	58.73(56.55-60.91)	61	-0.61(-0.67,-0.55)	-0.79(-0.84,-0.73)	-0.28(-0.32,-0.24)	-0.57(-0.60,-0.54)	126

Lithuania	69.53(66.79-72.14)	71	57.60(55.41-59.75)	68	-0.61(-0.66,-0.57)	-0.86(-0.89,-0.82)	-0.40(-0.42,-0.38)	-0.64(-0.66,-0.62)	151
Republic of Moldova	71.23(68.58-73.88)	62	61.25(58.87-63.51)	47	-0.31(-0.38,-0.23)	-0.73(-0.78,-0.67)	-0.49(-0.53,-0.45)	-0.52(-0.55,-0.48)	109
Russian Federation	65.91(63.50-68.38)	84	59.21(57.28-61.16)	58	-0.63(-0.67,-0.59)	-0.24(-0.35,-0.13)	-0.20(-0.25,-0.15)	-0.38(-0.42,-0.34)	63
Ukraine	68.60(66.11-71.12)	74	60.36(58.10-62.65)	53	-0.36(-0.42,-0.31)	-0.64(-0.69,-0.59)	-0.25(-0.28,-0.21)	-0.44(-0.46,-0.41)	87
Brunei Darussalam	37.38(36.03-38.81)	148	34.88(33.55-36.29)	133	-0.30(-0.31,-0.30)	-0.30(-0.31,-0.30)	-0.14(-0.18,-0.10)	-0.24(-0.25,-0.22)	26
Japan	40.49(39.10-41.93)	131	40.38(39.01-41.81)	118	-0.06(-0.10,-0.02)	0.04(0.03,0.05)	-0.01(-0.06,0.04)	-0.01(-0.03,0.01)	1
Republic of Korea	44.19(42.50-45.92)	124	39.90(38.31-41.50)	121	-0.61(-0.63,-0.59)	-0.28(-0.31,-0.25)	-0.15(-0.19,-0.11)	-0.34(-0.36,-0.32)	51
Singapore	35.99(34.58-37.41)	153	33.04(31.78-34.29)	142	-0.42(-0.46,-0.39)	-0.31(-0.32,-0.30)	-0.13(-0.18,-0.08)	-0.29(-0.31,-0.27)	40
Australia	155.05(149.08-161.14)	26	142.73(136.77-148.79)	26	0.03(0.00,0.06)	-0.26(-0.33,-0.19)	-0.68(-0.73,-0.63)	-0.29(-0.32,-0.26)	41
New Zealand	171.59(165.66-177.59)	25	159.87(154.62-165.44)	25	-0.25(-0.29,-0.22)	-0.18(-0.20,-0.16)	-0.32(-0.35,-0.30)	-0.25(-0.27,-0.24)	32
Andorra	212.89(205.45-220.17)	19	205.10(197.96-212.51)	16	-0.14(-0.15,-0.12)	-0.14(-0.15,-0.13)	-0.11(-0.12,-0.10)	-0.13(-0.14,-0.12)	5
Austria	229.67(221.20-237.70)	10	216.20(208.51-223.83)	8	-0.28(-0.30,-0.26)	-0.18(-0.20,-0.16)	-0.16(-0.17,-0.16)	-0.21(-0.22,-0.20)	20
Belgium	234.08(225.61-242.24)	6	218.93(211.04-227.05)	5	-0.27(-0.27,-0.26)	-0.21(-0.21,-0.20)	-0.22(-0.23,-0.20)	-0.23(-0.24,-0.23)	25
Cyprus	229.08(220.91-236.95)	11	204.89(197.27-212.13)	17	-0.58(-0.58,-0.57)	-0.43(-0.44,-0.42)	-0.15(-0.16,-0.15)	-0.38(-0.39,-0.38)	64
Denmark	230.39(222.16-238.47)	9	218.22(210.57-225.87)	7	-0.24(-0.26,-0.23)	-0.16(-0.20,-0.13)	-0.14(-0.14,-0.14)	-0.19(-0.20,-0.17)	14
Finland	247.68(239.38-256.34)	2	232.84(224.40-240.97)	2	-0.23(-0.23,-0.22)	-0.23(-0.23,-0.22)	-0.19(-0.19,-0.18)	-0.21(-0.22,-0.21)	21
France	269.37(259.26-279.81)	1	251.69(242.53-261.04)	1	-0.11(-0.14,-0.07)	-0.23(-0.25,-0.20)	-0.40(-0.42,-0.37)	-0.24(-0.26,-0.22)	27
Germany	188.69(181.93-195.68)	21	182.18(176.00-188.52)	21	-0.20(-0.21,-0.19)	-0.20(-0.21,-0.19)	-0.03(-0.27,0.21)	-0.15(-0.22,-0.07)	8
Greece	231.79(223.71-240.33)	7	215.64(208.24-223.06)	9	-0.38(-0.38,-0.37)	-0.27(-0.27,-0.26)	-0.11(-0.12,-0.09)	-0.25(-0.26,-0.24)	33
Iceland	246.90(238.40-255.18)	3	230.03(221.98-238.28)	3	-0.25(-0.25,-0.24)	-0.27(-0.28,-0.27)	-0.21(-0.21,-0.20)	-0.24(-0.25,-0.24)	28
Ireland	239.47(231.16-247.78)	5	218.86(211.50-226.86)	6	-0.37(-0.38,-0.36)	-0.30(-0.31,-0.30)	-0.26(-0.27,-0.25)	-0.31(-0.32,-0.31)	44
Israel	218.52(211.23-225.94)	15	206.30(199.54-214.06)	15	-0.24(-0.26,-0.23)	-0.17(-0.18,-0.16)	-0.19(-0.20,-0.18)	-0.20(-0.21,-0.19)	18
Italy	184.41(178.27-190.41)	22	172.83(166.99-178.66)	23	-0.03(-0.15,0.08)	-0.12(-0.17,-0.07)	-0.62(-0.71,-0.53)	-0.24(-0.29,-0.18)	29
Luxembourg	221.32(213.87-228.95)	14	212.07(204.68-219.30)	11	-0.15(-0.18,-0.12)	-0.16(-0.16,-0.15)	-0.13(-0.16,-0.11)	-0.15(-0.16,-0.14)	9
Malta	231.18(223.17-239.56)	8	211.56(203.96-218.96)	12	-0.38(-0.39,-0.36)	-0.26(-0.27,-0.26)	-0.27(-0.28,-0.26)	-0.31(-0.31,-0.30)	45
Netherlands	227.96(220.01-235.90)	12	215.61(207.84-223.43)	10	-0.24(-0.24,-0.23)	-0.18(-0.19,-0.16)	-0.16(-0.17,-0.16)	-0.19(-0.20,-0.19)	15
Norway	173.44(167.91-179.11)	24	165.30(160.02-170.69)	24	-0.20(-0.21,-0.19)	-0.20(-0.21,-0.19)	-0.12(-0.23,-0.01)	-0.17(-0.21,-0.14)	11
Portugal	244.58(235.64-253.11)	4	223.65(215.51-231.76)	4	-0.38(-0.39,-0.38)	-0.28(-0.29,-0.28)	-0.26(-0.26,-0.26)	-0.31(-0.31,-0.30)	46
Spain	224.61(215.33-233.61)	13	208.98(200.67-217.33)	13	-0.33(-0.34,-0.32)	-0.21(-0.21,-0.20)	-0.21(-0.23,-0.19)	-0.25(-0.25,-0.24)	34
Sweden	179.42(173.08-185.66)	23	175.79(169.54-181.99)	22	-0.17(-0.18,-0.15)	-0.04(-0.07,-0.02)	-0.03(-0.10,0.05)	-0.08(-0.11,-0.05)	3
Switzerland	211.82(204.19-218.85)	20	203.90(196.94-211.48)	19	-0.14(-0.17,-0.11)	-0.13(-0.13,-0.13)	-0.13(-0.13,-0.13)	-0.13(-0.14,-0.12)	6
United Kingdom	218.28(211.54-225.22)	16	201.59(195.40-207.84)	20	-0.32(-0.34,-0.30)	-0.25(-0.26,-0.25)	-0.24(-0.27,-0.22)	-0.27(-0.28,-0.26)	39
Argentina	110.06(105.41-114.87)	29	102.87(98.53-107.31)	28	-0.33(-0.34,-0.32)	-0.15(-0.16,-0.14)	-0.25(-0.29,-0.20)	-0.24(-0.25,-0.22)	30
Chile	110.63(105.77-115.35)	28	100.16(95.92-104.24)	30	-0.42(-0.45,-0.38)	-0.30(-0.32,-0.28)	-0.33(-0.35,-0.31)	-0.35(-0.36,-0.33)	54
Uruguay	108.41(103.89-113.18)	30	101.79(97.46-106.31)	29	-0.22(-0.26,-0.19)	-0.13(-0.14,-0.11)	-0.30(-0.31,-0.28)	-0.22(-0.24,-0.21)	23
Canada	96.34(93.05-99.37)	33	88.50(85.65-91.68)	34	-0.34(-0.35,-0.33)	-0.34(-0.34,-0.34)	-0.20(-0.21,-0.19)	-0.29(-0.30,-0.29)	42
United States of America	105.99(102.67-109.14)	32	93.16(90.26-96.00)	31	-0.03(-0.08,0.02)	-1.06(-1.16,-0.97)	-0.17(-0.22,-0.12)	-0.47(-0.51,-0.42)	99
Antigua and Barbuda	55.61(53.29-57.95)	107	50.27(48.20-52.37)	97	-0.40(-0.41,-0.40)	-0.40(-0.41,-0.40)	-0.25(-0.27,-0.23)	-0.35(-0.36,-0.34)	55

Bahamas	53.85(51.73-56.09)	109	50.00(47.92-52.02)	100	-0.28(-0.39,-0.16)	-0.34(-0.37,-0.30)	-0.11(-0.16,-0.07)	-0.25(-0.29,-0.21)	35
Barbados	52.64(50.52-54.79)	114	49.07(47.09-51.16)	103	-0.25(-0.29,-0.22)	-0.32(-0.35,-0.30)	-0.16(-0.17,-0.14)	-0.24(-0.25,-0.22)	31
Belize	65.86(63.19-68.43)	85	56.46(54.18-58.82)	77	-0.77(-0.80,-0.75)	-0.54(-0.56,-0.52)	-0.25(-0.27,-0.23)	-0.52(-0.54,-0.51)	110
Cuba	58.32(56.05-60.82)	99	54.66(52.41-56.96)	81	-0.03(-0.04,-0.02)	-0.28(-0.30,-0.26)	-0.39(-0.40,-0.38)	-0.22(-0.23,-0.21)	24
Dominica	56.88(54.74-59.23)	102	50.52(48.55-52.56)	96	-0.54(-0.55,-0.53)	-0.47(-0.49,-0.46)	-0.20(-0.22,-0.18)	-0.41(-0.42,-0.40)	73
Dominican Republic	65.10(62.30-67.89)	88	57.03(54.64-59.37)	73	-0.11(-0.13,-0.08)	-0.96(-1.00,-0.93)	-0.38(-0.44,-0.33)	-0.46(-0.49,-0.44)	97
Grenada	61.08(58.56-63.71)	93	52.27(50.08-54.46)	90	-1.00(-1.02,-0.98)	-0.33(-0.35,-0.31)	-0.31(-0.35,-0.27)	-0.53(-0.54,-0.51)	114
Guyana	58.22(55.81-60.54)	100	52.04(49.88-54.12)	91	-0.45(-0.48,-0.42)	-0.26(-0.28,-0.25)	-0.42(-0.44,-0.40)	-0.38(-0.40,-0.37)	65
Haiti	71.89(68.95-74.80)	58	65.21(62.59-67.80)	44	-0.39(-0.42,-0.36)	-0.34(-0.35,-0.32)	-0.26(-0.27,-0.24)	-0.33(-0.34,-0.32)	49
Jamaica	59.72(57.19-62.16)	96	52.88(50.77-55.16)	86	-0.49(-0.50,-0.49)	-0.45(-0.47,-0.44)	-0.28(-0.30,-0.25)	-0.41(-0.42,-0.40)	74
Saint Lucia	59.44(57.05-61.96)	97	52.44(50.23-54.64)	88	-0.70(-0.71,-0.70)	-0.33(-0.33,-0.32)	-0.24(-0.25,-0.22)	-0.43(-0.43,-0.42)	83
Saint Vincent and the Grenadines	61.08(58.53-63.50)	92	53.88(51.51-56.14)	85	-0.58(-0.59,-0.57)	-0.39(-0.40,-0.38)	-0.32(-0.35,-0.30)	-0.43(-0.44,-0.42)	84
Suriname	56.43(54.17-58.73)	104	50.84(48.83-52.86)	95	-0.27(-0.29,-0.26)	-0.50(-0.51,-0.48)	-0.31(-0.31,-0.30)	-0.36(-0.36,-0.35)	56
Trinidad and Tobago	53.06(50.95-55.34)	112	47.63(45.67-49.64)	104	-0.42(-0.44,-0.40)	-0.51(-0.54,-0.48)	-0.17(-0.18,-0.15)	-0.37(-0.38,-0.35)	59
Bolivia (Plurinational State of)	107.26(102.83-111.79)	31	92.16(88.06-96.08)	32	-0.71(-0.72,-0.69)	-0.44(-0.49,-0.39)	-0.48(-0.52,-0.43)	-0.53(-0.55,-0.51)	115
Ecuador	89.02(85.11-93.12)	38	76.72(73.47-80.21)	37	-0.43(-0.54,-0.32)	-0.55(-0.56,-0.53)	-0.55(-0.56,-0.53)	-0.51(-0.55,-0.48)	108
Peru	93.85(89.77-98.01)	36	81.88(78.21-85.25)	36	-0.35(-0.50,-0.20)	-0.40(-0.43,-0.37)	-0.71(-0.77,-0.64)	-0.48(-0.53,-0.43)	102
Colombia	22.02(21.17-22.83)	200	19.17(18.44-19.94)	201	-0.47(-0.56,-0.37)	-0.39(-0.50,-0.28)	-0.55(-0.58,-0.53)	-0.48(-0.53,-0.43)	103
Costa Rica	21.66(20.82-22.47)	202	19.02(18.25-19.74)	202	-0.61(-0.69,-0.53)	-0.37(-0.38,-0.36)	-0.37(-0.38,-0.36)	-0.44(-0.47,-0.42)	88
El Salvador	24.96(23.97-25.88)	194	21.31(20.46-22.19)	196	-0.56(-0.60,-0.52)	-0.63(-0.66,-0.59)	-0.40(-0.42,-0.38)	-0.54(-0.56,-0.52)	118
Guatemala	27.23(26.19-28.31)	183	22.59(21.68-23.47)	191	-0.82(-0.84,-0.79)	-0.64(-0.65,-0.62)	-0.44(-0.47,-0.42)	-0.63(-0.65,-0.62)	149
Honduras	26.78(25.73-27.91)	186	23.18(22.34-24.14)	188	-0.66(-0.71,-0.61)	-0.46(-0.50,-0.42)	-0.32(-0.33,-0.30)	-0.49(-0.51,-0.46)	105
Mexico	24.06(23.18-24.93)	197	21.09(20.32-21.89)	197	-0.61(-0.63,-0.60)	-0.34(-0.34,-0.33)	-0.40(-0.41,-0.38)	-0.46(-0.46,-0.45)	98
Nicaragua	26.02(25.02-27.03)	192	22.20(21.36-23.11)	193	-0.74(-0.81,-0.66)	-0.45(-0.46,-0.44)	-0.45(-0.46,-0.44)	-0.54(-0.56,-0.52)	119
Panama	21.19(20.42-22.01)	203	18.78(18.06-19.47)	203	-0.43(-0.58,-0.29)	-0.36(-0.38,-0.34)	-0.47(-0.56,-0.37)	-0.42(-0.47,-0.36)	77
Venezuela (Bolivarian Republic of)	21.87(21.02-22.67)	201	19.93(19.15-20.69)	199	-0.50(-0.58,-0.43)	-0.24(-0.37,-0.11)	-0.15(-0.22,-0.08)	-0.31(-0.37,-0.25)	47
Brazil	92.30(89.11-95.31)	37	87.04(84.04-89.94)	35	-0.24(-0.26,-0.23)	-0.15(-0.17,-0.12)	-0.20(-0.22,-0.18)	-0.19(-0.21,-0.18)	16
Paraguay	94.78(90.72-98.87)	35	90.11(86.20-94.15)	33	-0.18(-0.21,-0.14)	-0.07(-0.08,-0.05)	-0.26(-0.28,-0.24)	-0.17(-0.19,-0.15)	12
Algeria	67.92(65.07-70.65)	77	54.17(52.08-56.37)	83	-0.92(-0.93,-0.91)	-0.80(-0.84,-0.77)	-0.63(-0.67,-0.59)	-0.78(-0.80,-0.76)	179
Bahrain	60.78(58.28-63.34)	95	49.92(48.02-51.90)	101	-0.66(-0.68,-0.65)	-0.83(-0.86,-0.80)	-0.56(-0.60,-0.52)	-0.68(-0.69,-0.66)	167
Egypt	43.36(41.50-45.06)	126	34.52(33.29-35.81)	135	-1.09(-1.15,-1.03)	-0.52(-0.55,-0.49)	-0.78(-0.84,-0.72)	-0.78(-0.81,-0.74)	180
Iran (Islamic Republic of)	71.61(69.16-73.98)	59	54.44(52.53-56.26)	82	-0.91(-0.95,-0.86)	-1.23(-1.27,-1.18)	-0.68(-0.69,-0.67)	-0.93(-0.96,-0.91)	196
Iraq	79.30(75.91-82.68)	40	60.85(58.49-63.28)	50	-0.60(-0.68,-0.53)	-0.92(-0.96,-0.87)	-1.26(-1.31,-1.20)	-0.93(-0.96,-0.89)	197
Jordan	64.85(62.04-67.48)	89	51.73(49.67-53.72)	92	-0.76(-0.78,-0.75)	-0.85(-0.86,-0.84)	-0.69(-0.71,-0.68)	-0.77(-0.78,-0.77)	178
Kuwait	56.06(53.74-58.58)	106	46.60(44.77-48.35)	109	-0.53(-0.71,-0.35)	-0.79(-0.82,-0.76)	-0.59(-0.65,-0.54)	-0.65(-0.71,-0.59)	155
Lebanon	67.89(65.16-70.67)	78	52.71(50.65-54.76)	87	-0.72(-0.73,-0.71)	-1.04(-1.04,-1.03)	-0.84(-0.85,-0.83)	-0.87(-0.88,-0.86)	191
Libya	64.75(62.12-67.53)	90	50.26(48.33-52.36)	98	-1.39(-1.47,-1.31)	-0.96(-0.98,-0.93)	-0.21(-0.24,-0.18)	-0.86(-0.89,-0.83)	190
Morocco	75.21(72.16-78.34)	48	60.20(57.80-62.55)	54	-0.67(-0.68,-0.65)	-0.80(-0.82,-0.77)	-0.82(-0.84,-0.81)	-0.76(-0.78,-0.75)	177

Palestine	76.80(73.54-79.98)	46	56.99(54.75-59.21)	74	-1.18(-1.22,-1.13)	-0.95(-1.09,-0.81)	-0.97(-1.06,-0.88)	-1.04(-1.10,-0.98)	201
Oman	66.98(64.30-69.74)	82	46.94(45.08-48.79)	107	-1.77(-1.78,-1.75)	-1.17(-1.20,-1.14)	-0.72(-0.74,-0.70)	-1.22(-1.23,-1.20)	202
Qatar	58.71(56.28-61.09)	98	45.78(43.96-47.63)	110	-1.01(-1.02,-1.00)	-0.86(-0.87,-0.86)	-0.68(-0.69,-0.67)	-0.85(-0.86,-0.85)	189
Saudi Arabia	71.06(67.99-74.32)	63	47.26(45.31-49.08)	106	-1.98(-2.02,-1.93)	-1.29(-1.31,-1.27)	-0.90(-0.93,-0.88)	-1.39(-1.41,-1.37)	203
Syrian Arab Republic	73.00(70.12-75.86)	55	57.27(55.01-59.55)	71	-1.05(-1.06,-1.03)	-0.96(-0.98,-0.95)	-0.45(-0.46,-0.43)	-0.83(-0.84,-0.82)	187
Tunisia	69.27(66.29-72.12)	72	54.81(52.71-57.01)	80	-0.97(-1.00,-0.94)	-0.83(-0.87,-0.79)	-0.59(-0.61,-0.58)	-0.80(-0.82,-0.78)	183
Turkey	95.39(91.46-99.34)	34	74.72(71.90-77.54)	38	-0.92(-0.92,-0.91)	-0.82(-0.82,-0.81)	-0.78(-0.83,-0.73)	-0.84(-0.85,-0.82)	188
United Arab Emirates	57.08(54.76-59.46)	101	43.97(42.21-45.67)	112	-1.26(-1.28,-1.24)	-1.06(-1.08,-1.05)	-0.32(-0.37,-0.26)	-0.89(-0.91,-0.87)	193
Yemen	78.31(75.33-81.37)	43	60.54(58.09-62.97)	52	-0.86(-0.88,-0.83)	-1.26(-1.27,-1.24)	-0.52(-0.56,-0.47)	-0.89(-0.91,-0.87)	194
Afghanistan	85.84(82.40-89.32)	39	70.94(68.26-73.78)	39	-0.15(-0.18,-0.13)	-0.90(-0.91,-0.88)	-0.91(-0.95,-0.86)	-0.65(-0.67,-0.63)	156
Bangladesh	46.83(45.16-48.62)	118	39.21(37.70-40.66)	123	-0.71(-0.73,-0.69)	-0.41(-0.42,-0.40)	-0.73(-0.73,-0.72)	-0.61(-0.61,-0.60)	140
Bhutan	48.73(46.96-50.52)	115	41.07(39.60-42.60)	115	-0.54(-0.55,-0.52)	-0.63(-0.64,-0.62)	-0.58(-0.59,-0.57)	-0.59(-0.59,-0.58)	135
India	48.61(46.95-50.32)	116	45.72(44.12-47.31)	111	0.13(0.12,0.14)	-0.30(-0.31,-0.29)	-0.50(-0.53,-0.46)	-0.21(-0.22,-0.20)	22
Nepal	45.35(43.74-46.96)	120	34.99(33.78-36.33)	131	-0.78(-0.82,-0.74)	-1.08(-1.09,-1.07)	-0.76(-0.88,-0.64)	-0.89(-0.93,-0.85)	195
Pakistan	48.53(46.87-50.26)	117	41.09(39.61-42.57)	114	-0.58(-0.60,-0.56)	-0.61(-0.63,-0.60)	-0.51(-0.52,-0.50)	-0.58(-0.58,-0.57)	132
Angola	65.95(63.15-68.87)	83	51.33(49.14-53.50)	93	-0.72(-0.83,-0.61)	-0.72(-0.77,-0.67)	-1.20(-1.22,-1.17)	-0.88(-0.91,-0.84)	192
Central African Republic	65.67(62.93-68.40)	87	57.71(55.20-60.21)	67	-0.43(-0.45,-0.41)	-0.44(-0.48,-0.40)	-0.46(-0.48,-0.44)	-0.45(-0.47,-0.43)	92
Congo	54.58(52.27-57.21)	108	43.44(41.58-45.37)	113	-0.68(-0.71,-0.65)	-0.74(-0.76,-0.72)	-0.91(-0.97,-0.85)	-0.78(-0.80,-0.76)	181
Democratic Republic of the Congo	61.08(58.45-63.86)	94	52.29(49.99-54.59)	89	-0.11(-0.20,-0.02)	-0.23(-0.33,-0.14)	-1.35(-1.41,-1.29)	-0.55(-0.61,-0.50)	120
Equatorial Guinea	65.80(62.91-68.68)	86	40.70(38.90-42.61)	116	-1.56(-1.61,-1.51)	-2.17(-2.19,-2.15)	-1.20(-1.28,-1.12)	-1.66(-1.69,-1.62)	204
Gabon	53.10(50.70-55.46)	111	40.65(38.89-42.35)	117	-0.95(-0.99,-0.90)	-0.76(-0.79,-0.72)	-1.07(-1.11,-1.04)	-0.93(-0.95,-0.90)	198
Burundi	27.28(26.19-28.36)	182	26.50(25.49-27.49)	173	-0.07(-0.09,-0.05)	0.00(-0.07,0.06)	-0.23(-0.29,-0.16)	-0.11(-0.14,-0.08)	4
Comoros	26.69(25.66-27.77)	189	24.36(23.41-25.30)	181	-0.44(-0.46,-0.41)	-0.21(-0.24,-0.17)	-0.33(-0.34,-0.31)	-0.32(-0.33,-0.30)	48
Djibouti	26.80(25.80-27.89)	185	23.96(23.08-24.87)	186	-0.28(-0.31,-0.25)	-0.48(-0.51,-0.45)	-0.41(-0.44,-0.38)	-0.39(-0.41,-0.37)	67
Eritrea	29.31(28.15-30.46)	175	25.98(25.02-27.02)	177	-0.80(-0.88,-0.72)	-0.14(-0.17,-0.11)	-0.36(-0.38,-0.33)	-0.42(-0.45,-0.39)	78
Ethiopia	28.67(27.65-29.68)	178	25.59(24.70-26.50)	178	-0.34(-0.43,-0.26)	-0.26(-0.30,-0.23)	-0.60(-0.62,-0.58)	-0.40(-0.43,-0.37)	71
Kenya	24.36(23.49-25.24)	195	22.19(21.39-23.00)	194	-0.48(-0.50,-0.46)	-0.06(-0.10,-0.02)	-0.42(-0.44,-0.41)	-0.33(-0.35,-0.31)	50
Madagascar	28.36(27.26-29.52)	180	26.39(25.45-27.39)	175	-0.17(-0.21,-0.12)	-0.17(-0.21,-0.14)	-0.44(-0.47,-0.41)	-0.26(-0.28,-0.23)	37
Malawi	29.07(27.96-30.24)	176	26.32(25.27-27.38)	176	-0.30(-0.48,-0.11)	-0.28(-0.37,-0.19)	-0.54(-0.58,-0.50)	-0.37(-0.44,-0.30)	60
Mauritius	26.18(25.17-27.15)	191	22.62(21.70-23.54)	190	-0.56(-0.59,-0.53)	-0.52(-0.53,-0.52)	-0.43(-0.47,-0.39)	-0.50(-0.52,-0.48)	107
Mozambique	31.66(30.46-32.91)	171	27.95(26.95-29.04)	168	-0.40(-0.49,-0.31)	-0.32(-0.40,-0.23)	-0.57(-0.60,-0.55)	-0.45(-0.49,-0.40)	93
Rwanda	24.30(22.94-25.67)	196	21.77(20.63-22.92)	195	-0.35(-0.42,-0.29)	-0.25(-0.31,-0.19)	-0.52(-0.54,-0.51)	-0.39(-0.42,-0.36)	68
Seychelles	23.38(22.47-24.34)	199	20.68(19.83-21.47)	198	-0.66(-0.69,-0.64)	-0.30(-0.32,-0.28)	-0.32(-0.35,-0.30)	-0.42(-0.44,-0.41)	79
Somalia	31.49(30.27-32.72)	172	31.41(30.24-32.59)	155	-0.11(-0.17,-0.05)	0.15(0.09,0.20)	-0.05(-0.07,-0.04)	-0.02(-0.04,0.01)	2
United Republic of Tanzania	27.07(25.52-28.61)	184	24.24(22.96-25.56)	183	-0.43(-0.50,-0.36)	-0.22(-0.25,-0.19)	-0.53(-0.55,-0.52)	-0.39(-0.41,-0.36)	69
Uganda	27.86(26.84-28.93)	181	24.02(23.12-24.95)	184	-0.36(-0.51,-0.20)	-0.63(-0.65,-0.61)	-0.54(-0.63,-0.45)	-0.52(-0.57,-0.46)	111
Zambia	26.75(25.76-27.79)	187	23.67(22.81-24.55)	187	-0.28(-0.34,-0.23)	-0.36(-0.52,-0.19)	-0.65(-0.81,-0.49)	-0.44(-0.51,-0.36)	89
Botswana	37.97(36.52-39.47)	144	31.67(30.41-33.02)	151	-0.95(-0.99,-0.90)	-0.66(-0.79,-0.54)	-0.25(-0.31,-0.20)	-0.64(-0.69,-0.59)	152

Lesotho	43.53(41.74-45.34)	125	37.16(35.66-38.64)	125	-0.86(-1.00,-0.72)	-0.35(-0.43,-0.28)	-0.52(-0.54,-0.49)	-0.56(-0.62,-0.51)	122
Namibia	37.52(35.97-39.08)	147	32.26(30.95-33.59)	147	-0.72(-0.89,-0.55)	-0.36(-0.45,-0.28)	-0.59(-0.63,-0.56)	-0.55(-0.61,-0.48)	121
South Africa	35.96(34.75-37.23)	154	32.06(30.91-33.19)	149	-0.51(-0.58,-0.45)	-0.40(-0.64,-0.16)	-0.33(-0.42,-0.24)	-0.43(-0.52,-0.34)	85
Eswatini	39.97(38.36-41.61)	133	34.14(32.74-35.60)	138	-0.82(-0.87,-0.77)	-0.45(-0.57,-0.33)	-0.38(-0.43,-0.33)	-0.57(-0.62,-0.52)	127
Zimbabwe	38.34(36.85-39.93)	140	35.82(34.34-37.36)	128	-0.65(-0.70,-0.61)	0.35(0.30,0.40)	-0.48(-0.53,-0.42)	-0.25(-0.28,-0.23)	36
Benin	36.70(35.39-38.06)	152	31.00(29.85-32.16)	159	-0.99(-1.37,-0.60)	-0.33(-0.54,-0.12)	-0.63(-0.70,-0.56)	-0.62(-0.77,-0.48)	144
Burkina Faso	39.43(37.94-40.91)	136	33.98(32.74-35.25)	140	-0.83(-1.22,-0.43)	-0.36(-0.59,-0.12)	-0.55(-0.62,-0.47)	-0.56(-0.72,-0.40)	123
Cameroon	33.25(32.04-34.50)	166	27.79(26.77-28.83)	169	-0.89(-1.00,-0.79)	-0.46(-0.90,-0.02)	-0.62(-0.73,-0.52)	-0.67(-0.81,-0.52)	161
Cabo Verde	35.61(34.26-36.89)	158	28.52(27.47-29.61)	167	-1.12(-1.58,-0.65)	-0.67(-0.73,-0.62)	-0.67(-0.73,-0.62)	-0.81(-0.96,-0.66)	184
Chad	40.64(39.17-42.18)	130	34.83(33.51-36.08)	134	-0.73(-1.01,-0.45)	-0.26(-0.59,0.08)	-0.68(-0.75,-0.61)	-0.58(-0.73,-0.44)	133
C??te d'Ivoire	34.95(33.66-36.27)	161	29.60(28.52-30.71)	164	-1.03(-1.27,-0.79)	-0.23(-0.35,-0.10)	-0.62(-0.67,-0.57)	-0.60(-0.69,-0.51)	138
Gambia	37.53(36.15-38.92)	146	31.09(29.91-32.24)	157	-1.03(-1.41,-0.65)	-0.46(-0.67,-0.25)	-0.65(-0.72,-0.58)	-0.69(-0.84,-0.55)	168
Ghana	39.47(37.64-41.36)	135	32.96(31.41-34.49)	143	-0.72(-1.04,-0.39)	-0.43(-0.81,-0.05)	-0.79(-0.87,-0.71)	-0.67(-0.84,-0.50)	162
Guinea	37.92(36.50-39.27)	145	32.46(31.28-33.65)	145	-0.93(-1.30,-0.57)	-0.33(-0.53,-0.12)	-0.50(-0.56,-0.44)	-0.57(-0.71,-0.42)	128
Guinea-Bissau	38.04(36.65-39.42)	143	32.08(30.90-33.21)	148	-1.01(-1.37,-0.64)	-0.36(-0.57,-0.15)	-0.59(-0.65,-0.53)	-0.63(-0.77,-0.49)	150
Liberia	35.53(34.25-36.86)	159	30.59(29.49-31.72)	160	-0.23(-0.40,-0.05)	-0.75(-0.79,-0.72)	-0.57(-0.87,-0.26)	-0.53(-0.64,-0.42)	116
Mali	34.78(32.96-36.54)	163	28.69(27.19-30.16)	166	-0.80(-0.91,-0.68)	-0.73(-1.14,-0.32)	-0.60(-0.76,-0.45)	-0.73(-0.88,-0.58)	174
Mauritania	35.82(34.51-37.18)	156	29.99(28.83-31.11)	162	-1.00(-1.12,-0.89)	-0.41(-0.91,0.08)	-0.56(-0.68,-0.45)	-0.67(-0.83,-0.51)	163
Niger	41.67(40.14-43.18)	129	37.20(35.84-38.61)	124	-0.73(-0.93,-0.54)	-0.05(-0.28,0.18)	-0.41(-0.46,-0.37)	-0.43(-0.53,-0.33)	86
Nigeria	44.40(42.85-45.94)	122	33.38(32.21-34.54)	141	-0.57(-0.61,-0.53)	-1.04(-1.07,-1.01)	-1.30(-1.38,-1.23)	-0.99(-1.02,-0.96)	200
Sao Tome and Principe	32.75(31.59-33.91)	168	26.88(25.89-27.87)	171	-0.83(-1.15,-0.52)	-0.49(-0.86,-0.13)	-0.79(-0.86,-0.72)	-0.73(-0.90,-0.57)	175
Senegal	37.00(35.61-38.38)	150	31.57(30.41-32.75)	153	-0.93(-1.29,-0.57)	-0.30(-0.50,-0.10)	-0.60(-0.66,-0.53)	-0.59(-0.72,-0.45)	136
Sierra Leone	36.79(35.47-38.16)	151	31.64(30.48-32.83)	152	-0.65(-0.90,-0.40)	-0.28(-0.58,0.03)	-0.71(-0.77,-0.65)	-0.57(-0.70,-0.44)	129
Togo	34.88(33.63-36.19)	162	29.76(28.68-30.89)	163	-0.91(-1.13,-0.68)	-0.13(-0.39,0.14)	-0.64(-0.69,-0.58)	-0.59(-0.71,-0.47)	137
American Samoa	35.96(34.49-37.46)	155	31.86(30.60-33.15)	150	-0.48(-0.64,-0.32)	-0.29(-0.33,-0.26)	-0.51(-0.74,-0.29)	-0.42(-0.51,-0.33)	80
Bermuda	56.54(54.25-58.88)	103	51.18(49.15-53.31)	94	-0.31(-0.34,-0.28)	-0.44(-0.46,-0.42)	-0.24(-0.26,-0.21)	-0.34(-0.36,-0.32)	52
Cook Islands	35.73(34.22-37.20)	157	31.08(29.83-32.32)	158	-0.52(-0.58,-0.46)	-0.43(-0.46,-0.41)	-0.53(-0.56,-0.50)	-0.49(-0.51,-0.46)	106
Greenland	116.63(113.04-120.46)	27	104.62(101.11-108.13)	27	-0.27(-0.29,-0.24)	-0.54(-0.55,-0.53)	-0.29(-0.35,-0.23)	-0.38(-0.40,-0.36)	66
Guam	32.96(31.62-34.29)	167	29.14(27.91-30.31)	165	-0.53(-0.60,-0.46)	-0.41(-0.48,-0.35)	-0.37(-0.42,-0.32)	-0.44(-0.48,-0.41)	90
Monaco	212.91(205.91-220.32)	18	204.47(197.34-211.67)	18	-0.17(-0.20,-0.14)	-0.12(-0.13,-0.12)	-0.12(-0.13,-0.12)	-0.14(-0.15,-0.13)	7
Nauru	35.23(33.75-36.62)	160	31.45(30.20-32.72)	154	-0.09(-0.12,-0.07)	-0.18(-0.25,-0.11)	-0.86(-1.03,-0.69)	-0.37(-0.44,-0.30)	61
Niue	37.30(35.76-38.87)	149	32.39(31.12-33.75)	146	-0.53(-0.57,-0.50)	-0.51(-0.52,-0.51)	-0.40(-0.44,-0.36)	-0.48(-0.50,-0.47)	104
Northern Mariana Islands	33.59(32.22-35.08)	165	31.14(29.94-32.41)	156	-0.55(-0.64,-0.46)	-0.08(-0.13,-0.04)	-0.19(-0.26,-0.12)	-0.26(-0.30,-0.22)	38
Palau	33.92(32.51-35.35)	164	30.27(28.96-31.54)	161	-0.47(-0.52,-0.41)	-0.33(-0.34,-0.31)	-0.39(-0.50,-0.28)	-0.39(-0.43,-0.35)	70
Puerto Rico	52.86(50.71-55.09)	113	47.58(45.66-49.54)	105	-0.38(-0.39,-0.37)	-0.40(-0.40,-0.40)	-0.31(-0.32,-0.29)	-0.36(-0.37,-0.36)	57
Saint Kitts and Nevis	56.34(54.02-58.72)	105	49.87(47.91-51.88)	102	-0.55(-0.56,-0.55)	-0.38(-0.39,-0.38)	-0.30(-0.31,-0.29)	-0.42(-0.42,-0.41)	81
San Marino	217.46(210.13-225.23)	17	207.65(200.21-215.07)	14	-0.26(-0.27,-0.24)	-0.12(-0.12,-0.12)	-0.09(-0.11,-0.07)	-0.16(-0.17,-0.15)	10
Tokelau	38.26(36.73-39.81)	141	32.50(31.18-33.84)	144	-0.51(-0.52,-0.50)	-0.55(-0.56,-0.53)	-0.62(-0.67,-0.58)	-0.56(-0.57,-0.54)	124

Tuvalu	39.16(37.57-40.74)	139	34.06(32.61-35.44)	139	-0.66(-0.69,-0.64)	-0.34(-0.35,-0.33)	-0.45(-0.51,-0.39)	-0.47(-0.49,-0.45)	100
United States Virgin Islands	53.53(51.37-55.78)	110	46.89(45.04-48.87)	108	-0.57(-0.60,-0.53)	-0.55(-0.57,-0.54)	-0.23(-0.25,-0.20)	-0.45(-0.47,-0.44)	94
South Sudan	26.71(25.67-27.78)	188	25.25(24.33-26.23)	180	-0.19(-0.25,-0.14)	-0.07(-0.13,-0.02)	-0.31(-0.32,-0.29)	-0.20(-0.23,-0.18)	19
Sudan	74.87(71.78-78.08)	51	56.57(54.42-58.88)	76	-0.91(-0.91,-0.90)	-0.98(-0.98,-0.97)	-0.99(-1.00,-0.98)	-0.96(-0.97,-0.96)	199
Atopic dermatitis									
China	409.40(393.09-424.47)	71	411.13(394.14-427.17)	71	-0.10(-0.12,-0.08)	0.06(0.05,0.07)	0.08(0.04,0.12)	0.01(0.00,0.03)	24
Democratic People's Republic of Korea	466.50(436.16-496.44)	51	456.72(427.20-485.76)	52	-0.03(-0.05,-0.02)	-0.13(-0.13,-0.12)	-0.05(-0.06,-0.05)	-0.07(-0.08,-0.07)	195
Taiwan (Province of China)	520.79(484.33-556.34)	14	529.40(495.43-566.74)	13	-0.12(-0.14,-0.11)	0.14(0.14,0.15)	0.14(0.12,0.15)	0.06(0.05,0.07)	4
Cambodia	521.30(488.43-556.85)	13	510.76(478.78-544.45)	17	-0.08(-0.08,-0.07)	-0.07(-0.07,-0.07)	-0.06(-0.06,-0.06)	-0.07(-0.07,-0.07)	196
Indonesia	565.43(541.84-589.05)	11	565.75(540.52-589.69)	11	-0.02(-0.02,-0.02)	0.04(0.03,0.05)	-0.01(-0.01,0.00)	0.00(0.00,0.00)	43
Lao People's Democratic Republic	515.04(482.64-549.23)	17	508.33(476.44-542.24)	21	-0.07(-0.07,-0.07)	-0.05(-0.06,-0.05)	-0.02(-0.02,-0.02)	-0.05(-0.05,-0.04)	191
Malaysia	508.51(476.69-542.57)	22	502.59(471.29-536.34)	24	-0.04(-0.04,-0.04)	-0.06(-0.06,-0.06)	-0.02(-0.03,-0.02)	-0.04(-0.04,-0.04)	187
Maldives	505.69(473.81-539.82)	25	475.36(446.00-506.79)	47	0.04(0.03,0.05)	-0.17(-0.23,-0.10)	-0.51(-0.54,-0.48)	-0.22(-0.24,-0.20)	202
Myanmar	511.12(479.00-545.09)	19	514.72(482.33-548.91)	15	0.02(0.02,0.03)	0.03(0.03,0.03)	0.02(0.02,0.02)	0.02(0.02,0.03)	15
Philippines	515.38(493.87-535.59)	16	512.85(491.60-532.99)	16	-0.02(-0.02,-0.01)	-0.02(-0.02,-0.02)	-0.02(-0.02,-0.01)	-0.02(-0.02,-0.02)	171
Sri Lanka	469.65(438.46-500.66)	50	470.57(437.88-502.92)	50	0.04(0.04,0.04)	-0.02(-0.03,-0.01)	-0.01(-0.01,-0.01)	0.01(0.00,0.01)	25
Thailand	510.66(478.64-544.59)	20	510.01(477.94-543.84)	18	0.00(0.00,0.01)	-0.03(-0.05,-0.02)	0.00(-0.01,0.02)	-0.01(-0.02,0.01)	127
Timor-Leste	504.12(472.49-537.71)	27	508.70(476.81-542.68)	19	0.03(0.03,0.04)	0.01(0.01,0.02)	0.05(0.05,0.06)	0.03(0.03,0.04)	7
Viet Nam	516.15(483.62-550.33)	15	508.25(476.47-541.93)	22	-0.09(-0.09,-0.09)	-0.04(-0.04,-0.04)	-0.03(-0.03,-0.03)	-0.05(-0.05,-0.05)	192
Fiji	486.76(453.33-519.44)	34	485.73(452.41-518.34)	36	-0.03(-0.03,-0.02)	0.00(0.00,0.00)	0.01(0.01,0.01)	-0.01(-0.01,-0.01)	128
Kiribati	490.92(457.28-523.74)	31	492.08(458.42-525.02)	29	0.02(0.02,0.02)	-0.02(-0.02,-0.02)	0.03(0.02,0.03)	0.01(0.01,0.01)	26
Marshall Islands	485.21(452.04-517.78)	38	484.86(451.44-517.47)	39	0.00(0.00,0.00)	-0.02(-0.02,-0.01)	0.01(0.01,0.01)	0.00(0.00,0.00)	44
Micronesia (Federated States of)	485.60(452.40-518.40)	37	485.24(451.92-517.91)	38	0.04(0.04,0.04)	-0.03(-0.03,-0.03)	-0.02(-0.02,-0.02)	0.00(0.00,0.00)	45
Papua New Guinea	483.62(450.33-516.22)	43	484.41(451.09-517.00)	40	0.03(0.02,0.03)	-0.01(-0.01,0.00)	0.00(0.00,0.00)	0.01(0.00,0.01)	27
Samoa	481.18(447.94-514.02)	46	484.39(451.12-516.88)	41	0.01(0.01,0.02)	0.02(0.02,0.02)	0.03(0.03,0.03)	0.02(0.02,0.02)	16
Solomon Islands	484.55(451.16-517.15)	40	487.26(453.81-519.81)	35	0.01(0.00,0.01)	0.03(0.03,0.03)	0.02(0.02,0.02)	0.02(0.02,0.02)	17
Tonga	489.49(455.74-522.30)	32	490.68(456.98-523.45)	30	-0.05(-0.05,-0.05)	0.02(0.02,0.02)	0.06(0.05,0.06)	0.01(0.01,0.01)	28
Vanuatu	486.38(452.92-518.89)	35	488.69(455.11-521.34)	32	0.01(0.00,0.01)	0.02(0.02,0.02)	0.02(0.02,0.02)	0.02(0.02,0.02)	18
Armenia	612.86(542.09-693.46)	9	607.10(536.53-687.14)	7	-0.02(-0.02,-0.02)	-0.05(-0.06,-0.05)	-0.02(-0.02,-0.02)	-0.03(-0.03,-0.03)	182
Azerbaijan	613.39(542.52-693.94)	8	605.13(534.71-685.15)	8	-0.03(-0.03,-0.02)	-0.08(-0.08,-0.08)	-0.03(-0.03,-0.03)	-0.05(-0.05,-0.05)	193
Georgia	614.79(543.93-695.32)	3	602.47(527.48-684.79)	9	-0.22(-0.32,-0.11)	-0.14(-0.16,-0.13)	0.17(0.05,0.30)	-0.07(-0.12,-0.02)	197
Kazakhstan	615.00(543.89-695.52)	2	613.08(542.28-693.54)	2	-0.01(-0.01,0.00)	-0.01(-0.01,-0.01)	-0.02(-0.02,-0.01)	-0.01(-0.01,-0.01)	129
Kyrgyzstan	615.15(544.04-695.87)	1	612.27(541.45-692.76)	3	-0.03(-0.03,-0.02)	0.00(-0.01,0.00)	-0.02(-0.02,-0.02)	-0.02(-0.02,-0.02)	172
Mongolia	613.60(542.59-694.56)	7	613.48(542.50-694.09)	1	0.01(0.01,0.01)	-0.01(-0.01,0.00)	0.00(0.00,0.00)	0.00(0.00,0.00)	46
Tajikistan	613.74(542.79-694.38)	6	610.40(539.53-691.06)	5	-0.02(-0.02,-0.02)	-0.03(-0.03,-0.03)	-0.01(-0.01,0.00)	-0.02(-0.02,-0.02)	173
Turkmenistan	614.67(543.68-695.49)	4	608.33(537.26-688.60)	6	-0.05(-0.05,-0.05)	-0.03(-0.03,-0.03)	-0.03(-0.03,-0.02)	-0.04(-0.04,-0.04)	188
Uzbekistan	614.50(543.49-695.30)	5	610.76(540.04-691.21)	4	-0.02(-0.02,-0.02)	-0.02(-0.02,-0.02)	-0.03(-0.03,-0.03)	-0.02(-0.02,-0.02)	174
Albania	202.60(187.79-218.14)	136	203.10(188.37-218.66)	132	0.06(0.05,0.07)	-0.01(-0.02,-0.01)	-0.03(-0.03,-0.03)	0.01(0.00,0.01)	29

Bosnia and Herzegovina	203.63(188.86-219.16)	134	203.74(188.98-219.29)	126	0.01(0.00,0.03)	-0.01(-0.01,0.00)	0.01(0.01,0.01)	0.00(0.00,0.01)	47
Bulgaria	203.92(189.20-219.47)	130	203.33(188.54-218.87)	130	0.00(-0.01,0.00)	-0.01(-0.01,-0.01)	-0.01(-0.02,-0.01)	-0.01(-0.01,-0.01)	130
Croatia	203.84(189.01-219.37)	132	203.54(188.77-219.09)	127	0.00(0.00,0.01)	-0.01(-0.01,-0.01)	-0.01(-0.01,-0.01)	-0.01(-0.01,0.00)	131
Czechia	203.92(189.16-219.46)	129	203.37(188.61-218.91)	129	-0.01(-0.01,-0.01)	-0.01(-0.01,-0.01)	-0.01(-0.01,-0.01)	-0.01(-0.01,-0.01)	132
Hungary	251.14(229.60-276.35)	109	250.51(228.99-275.71)	109	0.11(0.11,0.12)	-0.01(-0.01,-0.01)	-0.13(-0.14,-0.12)	-0.01(-0.01,-0.01)	133
North Macedonia	203.59(188.83-219.16)	135	202.95(188.14-218.49)	135	0.00(-0.02,0.01)	-0.02(-0.02,-0.01)	-0.02(-0.02,-0.01)	-0.01(-0.02,-0.01)	134
Montenegro	203.69(188.91-219.23)	133	203.23(188.41-218.79)	131	0.00(0.00,0.00)	-0.01(-0.01,-0.01)	-0.01(-0.01,-0.01)	-0.01(-0.01,-0.01)	135
Poland	206.13(193.87-218.51)	123	188.66(179.61-198.37)	155	0.00(-0.01,0.00)	-0.97(-0.98,-0.96)	-0.01(-0.02,-0.01)	-0.31(-0.31,-0.30)	204
Romania	151.56(140.46-161.99)	198	152.70(141.65-163.28)	197	0.00(0.00,0.00)	0.09(0.09,0.10)	0.00(-0.01,0.00)	0.03(0.02,0.03)	8
Serbia	205.47(190.60-221.22)	127	202.99(188.28-218.55)	134	-0.02(-0.04,0.00)	-0.06(-0.06,-0.05)	-0.04(-0.05,-0.04)	-0.04(-0.05,-0.04)	189
Slovakia	204.20(189.47-219.75)	128	203.53(188.76-219.08)	128	-0.01(-0.01,-0.01)	-0.01(-0.02,-0.01)	-0.01(-0.01,-0.01)	-0.01(-0.01,-0.01)	136
Slovenia	203.87(189.05-219.41)	131	203.02(188.23-218.57)	133	-0.02(-0.02,-0.01)	-0.02(-0.02,-0.02)	-0.01(-0.01,-0.01)	-0.01(-0.02,-0.01)	137
Belarus	205.62(188.83-223.35)	125	204.70(187.93-222.44)	124	-0.03(-0.03,-0.03)	0.00(0.00,0.00)	-0.02(-0.02,-0.02)	-0.02(-0.02,-0.01)	175
Estonia	309.87(279.65-341.89)	80	308.14(277.96-340.12)	79	0.00(0.00,0.01)	-0.02(-0.03,-0.01)	-0.04(-0.04,-0.04)	-0.02(-0.03,-0.02)	176
Latvia	145.26(132.51-158.88)	199	144.60(132.73-158.76)	199	0.00(-0.02,0.01)	0.00(-0.01,0.00)	-0.04(-0.04,-0.03)	-0.02(-0.02,-0.01)	177
Lithuania	205.60(188.78-223.38)	126	204.72(187.95-222.46)	123	-0.01(-0.01,-0.01)	-0.01(-0.01,-0.01)	-0.02(-0.03,-0.02)	-0.01(-0.02,-0.01)	138
Republic of Moldova	205.93(189.10-223.72)	124	204.56(187.79-222.29)	125	-0.03(-0.03,-0.03)	-0.02(-0.02,-0.02)	-0.02(-0.02,-0.02)	-0.02(-0.02,-0.02)	178
Russian Federation	283.29(263.84-303.85)	82	285.67(267.06-304.75)	82	0.22(0.13,0.31)	-0.11(-0.19,-0.03)	-0.02(-0.08,0.04)	0.03(-0.02,0.08)	9
Ukraine	219.03(204.39-234.35)	113	218.21(203.62-233.48)	114	-0.02(-0.02,-0.02)	0.00(-0.01,0.00)	-0.01(-0.02,-0.01)	-0.01(-0.01,-0.01)	139
Brunei Darussalam	470.37(434.29-508.23)	49	471.57(435.38-509.53)	48	0.12(0.11,0.12)	-0.03(-0.04,-0.03)	-0.05(-0.05,-0.05)	0.01(0.01,0.01)	30
Japan	584.46(548.76-622.92)	10	582.49(545.52-618.63)	10	0.11(0.10,0.13)	-0.02(-0.03,-0.02)	-0.14(-0.16,-0.11)	-0.02(-0.02,-0.01)	179
Republic of Korea	473.22(436.19-513.63)	48	475.65(437.09-517.90)	46	-0.11(-0.16,-0.06)	0.00(-0.01,0.01)	0.15(0.11,0.19)	0.01(-0.01,0.03)	31
Singapore	443.45(402.82-485.32)	66	440.78(400.23-482.48)	67	-0.05(-0.11,0.00)	-0.08(-0.19,0.03)	0.02(-0.02,0.05)	-0.03(-0.07,0.01)	183
Australia	346.00(316.85-377.99)	76	345.27(316.71-375.89)	76	0.06(0.05,0.07)	0.00(0.00,0.00)	-0.07(-0.08,-0.07)	0.00(-0.01,0.00)	48
New Zealand	436.67(410.01-464.69)	67	441.34(415.11-468.95)	66	0.01(0.01,0.01)	0.11(0.10,0.12)	0.00(0.00,0.00)	0.04(0.04,0.04)	5
Andorra	444.06(410.47-480.28)	65	447.38(413.49-483.76)	64	0.04(0.02,0.06)	-0.01(-0.09,0.07)	0.04(-0.01,0.10)	0.03(-0.01,0.08)	10
Austria	448.91(415.19-485.16)	57	447.49(413.86-483.76)	63	0.00(-0.01,0.00)	0.00(0.00,0.00)	-0.03(-0.04,-0.02)	-0.01(-0.01,-0.01)	140
Belgium	448.39(414.69-484.68)	62	448.54(414.85-484.89)	57	0.01(0.01,0.01)	0.00(0.00,0.00)	0.00(0.00,0.00)	0.00(0.00,0.00)	49
Cyprus	433.70(392.83-478.90)	69	433.42(391.83-477.86)	69	0.00(-0.01,0.01)	0.00(-0.01,0.00)	-0.01(-0.01,-0.01)	0.00(0.00,0.00)	50
Denmark	514.75(468.16-568.39)	18	518.13(469.83-570.80)	14	0.08(0.07,0.08)	0.01(0.00,0.01)	-0.01(-0.01,-0.01)	0.02(0.02,0.03)	19
Finland	449.00(415.22-485.26)	56	448.00(414.22-484.30)	60	-0.01(-0.01,0.00)	-0.01(-0.01,-0.01)	-0.01(-0.01,-0.01)	-0.01(-0.01,-0.01)	141
France	550.81(505.10-603.10)	12	550.98(505.28-603.33)	12	0.08(0.08,0.08)	0.00(0.00,0.00)	-0.08(-0.09,-0.07)	0.00(0.00,0.00)	51
Germany	436.35(405.29-468.71)	68	438.81(404.55-475.13)	68	-0.02(-0.08,0.04)	-0.22(-0.39,-0.05)	0.37(0.24,0.49)	0.04(-0.03,0.12)	6
Greece	383.51(346.56-423.21)	75	386.17(348.70-428.00)	75	-0.05(-0.06,-0.04)	0.10(0.09,0.10)	0.02(0.02,0.02)	0.03(0.02,0.03)	11
Iceland	492.88(450.41-542.49)	30	490.56(446.14-541.64)	31	0.04(0.02,0.05)	-0.04(-0.05,-0.03)	-0.04(-0.05,-0.02)	-0.01(-0.02,-0.01)	142
Ireland	448.44(414.80-484.73)	61	449.04(415.42-485.43)	56	0.00(0.00,0.00)	0.01(0.01,0.01)	0.01(0.00,0.01)	0.00(0.00,0.01)	52
Israel	449.41(415.73-485.75)	55	448.50(414.85-484.83)	58	0.00(-0.01,0.00)	-0.01(-0.01,-0.01)	-0.01(-0.01,-0.01)	-0.01(-0.01,-0.01)	143
Italy	508.61(477.19-541.25)	21	507.70(476.31-540.37)	23	0.02(0.01,0.02)	-0.01(-0.01,-0.01)	-0.03(-0.03,-0.02)	-0.01(-0.01,0.00)	144

Luxembourg	448.69(414.96-484.90)	59	447.59(413.81-483.83)	61	-0.01(-0.01,-0.01)	-0.01(-0.02,0.00)	-0.01(-0.02,-0.01)	-0.01(-0.01,0.00)	145
Malta	448.76(415.06-485.04)	58	446.81(413.08-483.10)	65	-0.02(-0.02,-0.02)	-0.02(-0.02,-0.01)	-0.01(-0.01,-0.01)	-0.02(-0.02,-0.01)	180
Netherlands	448.56(414.82-484.85)	60	448.28(414.53-484.63)	59	0.00(-0.01,0.00)	0.00(0.00,0.00)	-0.01(-0.01,0.00)	0.00(0.00,0.00)	53
Norway	496.83(467.38-528.21)	28	496.97(466.25-529.40)	27	0.19(0.18,0.20)	0.05(0.03,0.06)	-0.23(-0.26,-0.21)	0.00(-0.01,0.01)	54
Portugal	449.85(416.10-486.22)	53	449.82(416.11-486.23)	55	-0.01(-0.01,-0.01)	0.00(0.00,0.01)	0.00(0.00,0.01)	0.00(0.00,0.00)	55
Spain	424.19(387.12-467.10)	70	423.70(386.64-466.65)	70	0.03(0.00,0.06)	0.00(-0.01,0.00)	-0.04(-0.06,-0.01)	0.00(-0.01,0.01)	56
Sweden	461.71(434.23-489.60)	52	460.84(433.49-488.76)	51	0.00(0.00,0.00)	-0.01(-0.01,-0.01)	-0.01(-0.01,-0.01)	-0.01(-0.01,-0.01)	146
Switzerland	448.20(414.46-484.47)	63	447.49(413.80-483.78)	62	0.01(0.00,0.01)	-0.01(-0.01,-0.01)	-0.02(-0.02,-0.02)	-0.01(-0.01,0.00)	147
United Kingdom	505.00(475.70-537.06)	26	497.67(467.37-529.30)	25	-0.30(-0.37,-0.23)	-0.06(-0.08,-0.04)	0.23(0.12,0.34)	-0.06(-0.10,-0.02)	194
Argentina	402.55(371.55-436.24)	72	401.98(371.10-435.57)	72	0.00(-0.01,0.00)	-0.01(-0.01,0.00)	0.00(-0.01,0.00)	0.00(-0.01,0.00)	57
Chile	402.29(371.48-435.86)	73	401.27(370.41-434.75)	74	-0.02(-0.02,-0.02)	0.00(0.00,0.00)	-0.01(-0.01,-0.01)	-0.01(-0.01,-0.01)	148
Uruguay	402.17(371.27-435.79)	74	401.85(371.14-435.34)	73	0.00(0.00,0.00)	-0.01(-0.01,0.00)	0.00(0.00,0.00)	0.00(0.00,0.00)	58
Canada	244.82(231.25-258.68)	111	244.94(231.38-258.76)	111	0.01(0.00,0.01)	0.00(0.00,0.00)	0.00(0.00,0.00)	0.00(0.00,0.00)	59
United States of America	319.96(302.88-337.36)	78	300.42(284.01-317.26)	80	0.94(0.78,1.11)	-1.72(-1.81,-1.64)	0.06(-0.03,0.14)	-0.22(-0.29,-0.15)	203
Antigua and Barbuda	274.48(251.96-296.42)	91	274.01(251.53-295.91)	94	0.01(0.01,0.01)	-0.02(-0.02,-0.02)	-0.01(-0.01,-0.01)	-0.01(-0.01,-0.01)	149
Bahamas	274.32(251.80-296.25)	94	274.42(251.91-296.33)	89	0.00(0.00,0.00)	0.01(0.01,0.01)	0.00(0.00,0.00)	0.00(0.00,0.00)	60
Barbados	274.34(251.79-296.27)	93	273.93(251.44-295.83)	96	-0.01(-0.01,0.00)	-0.01(-0.01,-0.01)	0.00(0.00,0.00)	-0.01(-0.01,0.00)	150
Belize	273.75(251.38-295.62)	105	273.89(251.47-295.75)	99	0.00(0.00,0.00)	0.00(0.00,0.00)	0.00(0.00,0.00)	0.00(0.00,0.00)	61
Cuba	273.58(251.18-295.45)	107	273.47(251.05-295.35)	106	0.00(0.00,0.00)	0.00(0.00,0.00)	0.00(0.00,0.00)	0.00(0.00,0.00)	62
Dominica	273.73(251.24-295.62)	106	273.55(251.20-295.46)	105	0.01(0.01,0.01)	-0.01(-0.01,-0.01)	0.00(0.00,0.00)	0.00(0.00,0.00)	63
Dominican Republic	274.25(251.80-296.12)	97	273.77(251.35-295.63)	102	-0.01(-0.01,-0.01)	-0.01(-0.01,-0.01)	0.00(0.00,0.00)	-0.01(-0.01,-0.01)	151
Grenada	274.25(251.71-296.18)	98	273.46(251.08-295.34)	107	0.02(0.01,0.02)	-0.03(-0.03,-0.03)	-0.02(-0.02,-0.02)	-0.01(-0.01,-0.01)	152
Guyana	274.31(251.84-296.20)	95	274.05(251.60-295.96)	93	-0.01(-0.01,-0.01)	0.00(0.00,0.00)	0.00(0.00,0.00)	0.00(0.00,0.00)	64
Haiti	274.69(252.21-296.62)	89	274.40(251.89-296.31)	90	-0.01(-0.01,-0.01)	0.00(0.00,0.00)	0.00(0.00,0.00)	0.00(0.00,0.00)	65
Jamaica	274.22(251.76-296.14)	99	273.90(251.47-295.78)	98	-0.01(-0.01,-0.01)	0.00(0.00,0.00)	0.00(0.00,0.00)	0.00(0.00,0.00)	66
Saint Lucia	274.53(252.00-296.46)	90	273.85(251.42-295.75)	101	-0.01(-0.01,-0.01)	-0.01(-0.01,-0.01)	-0.01(-0.01,0.00)	-0.01(-0.01,-0.01)	153
Saint Vincent and the Grenadines	274.10(251.62-296.01)	102	273.63(251.29-295.52)	104	-0.01(-0.01,-0.01)	-0.01(-0.01,-0.01)	0.00(0.00,0.00)	-0.01(-0.01,-0.01)	154
Suriname	274.16(251.68-296.07)	101	273.89(251.44-295.81)	100	0.00(0.00,0.00)	-0.01(-0.01,-0.01)	0.00(0.00,0.00)	0.00(0.00,0.00)	67
Trinidad and Tobago	274.00(251.57-295.91)	104	273.72(251.33-295.61)	103	0.00(-0.01,0.00)	0.00(0.00,0.00)	0.00(0.00,0.00)	0.00(0.00,0.00)	68
Bolivia (Plurinational State of)	263.65(247.70-280.37)	108	263.33(247.36-280.05)	108	-0.01(-0.01,-0.01)	0.00(0.00,0.00)	0.00(0.00,0.00)	0.00(0.00,0.00)	69
Ecuador	278.87(260.90-297.89)	83	278.96(261.28-297.37)	83	0.01(0.01,0.02)	0.00(0.00,0.00)	-0.01(-0.01,-0.01)	0.00(0.00,0.00)	70
Peru	247.70(232.49-263.53)	110	247.34(232.04-263.22)	110	-0.30(-0.31,-0.29)	-0.02(-0.02,-0.02)	0.34(0.31,0.37)	0.00(-0.01,0.01)	71
Colombia	215.71(199.74-232.69)	118	215.60(199.71-232.60)	121	0.00(0.00,0.00)	0.00(0.00,0.00)	0.00(0.00,0.00)	0.00(0.00,0.00)	72
Costa Rica	215.49(199.58-232.47)	121	215.76(199.83-232.74)	118	0.00(0.00,0.00)	0.01(0.01,0.01)	0.00(0.00,0.00)	0.00(0.00,0.00)	73
El Salvador	215.84(199.87-232.83)	116	215.96(200.07-232.89)	115	0.01(0.01,0.01)	0.00(0.00,0.00)	0.00(0.00,0.00)	0.00(0.00,0.00)	74
Guatemala	215.73(199.75-232.70)	117	215.86(199.95-232.84)	116	0.00(0.00,0.00)	0.00(0.00,0.00)	0.00(0.00,0.00)	0.00(0.00,0.00)	75
Honduras	215.66(199.70-232.65)	119	215.77(199.83-232.74)	117	0.00(0.00,0.00)	0.00(0.00,0.00)	0.00(0.00,0.00)	0.00(0.00,0.00)	76
Mexico	218.68(206.22-230.54)	114	218.53(206.10-230.37)	113	-0.05(-0.05,-0.04)	-0.01(-0.01,-0.01)	0.04(0.04,0.04)	0.00(-0.01,0.00)	77

Nicaragua	215.91(199.92-232.89)	115	215.67(199.77-232.68)	119	-0.01(-0.01,-0.01)	-0.01(-0.01,-0.01)	0.00(0.00,0.00)	0.00(0.00,0.00)	78
Panama	215.40(199.49-232.38)	122	215.39(199.52-232.38)	122	0.00(0.00,0.00)	0.00(0.00,0.00)	0.00(0.00,0.00)	0.00(0.00,0.00)	79
Venezuela (Bolivarian Republic of)	215.65(199.72-232.64)	120	215.62(199.69-232.63)	120	0.00(-0.01,0.00)	-0.01(-0.01,0.00)	0.01(0.01,0.01)	0.00(0.00,0.00)	80
Brazil	320.64(303.58-339.20)	77	320.08(303.60-337.42)	77	-0.05(-0.06,-0.03)	0.00(0.00,0.01)	0.02(0.02,0.03)	-0.01(-0.01,0.00)	155
Paraguay	319.63(295.01-344.69)	79	319.32(294.73-344.33)	78	0.00(0.00,0.00)	0.00(0.00,0.00)	0.00(0.00,0.00)	0.00(0.00,0.00)	81
Algeria	193.67(180.54-207.61)	144	193.52(180.36-207.49)	144	0.00(0.00,0.00)	0.00(0.00,0.00)	0.00(0.00,0.00)	0.00(0.00,0.00)	82
Bahrain	190.90(177.94-204.51)	153	190.71(177.66-204.38)	153	0.02(0.01,0.02)	-0.07(-0.08,-0.07)	0.04(0.03,0.05)	0.00(-0.01,0.00)	83
Egypt	140.10(128.98-151.54)	201	135.67(125.32-146.28)	203	-0.21(-0.21,-0.20)	-0.08(-0.08,-0.08)	-0.01(-0.01,-0.01)	-0.11(-0.11,-0.11)	200
Iran (Islamic Republic of)	197.25(186.63-208.22)	137	198.13(187.69-209.04)	136	0.00(-0.01,0.00)	0.05(0.04,0.07)	-0.01(-0.01,0.00)	0.01(0.01,0.02)	32
Iraq	193.50(177.71-209.75)	148	193.40(177.62-209.66)	146	-0.17(-0.19,-0.16)	0.00(0.00,0.00)	0.18(0.15,0.22)	0.00(-0.01,0.01)	84
Jordan	192.83(179.75-206.80)	150	192.38(179.31-206.19)	150	0.02(0.02,0.02)	-0.01(-0.01,-0.01)	-0.03(-0.03,-0.03)	-0.01(-0.01,-0.01)	156
Kuwait	190.87(177.85-204.55)	154	192.51(179.38-206.33)	149	-0.04(-0.05,-0.03)	0.05(0.04,0.05)	0.08(0.08,0.09)	0.03(0.03,0.04)	12
Lebanon	193.74(180.47-207.83)	142	193.80(180.61-207.83)	138	-0.01(-0.01,0.00)	0.02(0.01,0.02)	-0.01(-0.01,-0.01)	0.00(0.00,0.00)	85
Libya	192.75(179.68-206.59)	151	193.17(180.05-207.03)	148	0.03(0.03,0.03)	-0.01(-0.01,-0.01)	0.01(0.00,0.01)	0.01(0.01,0.01)	33
Morocco	194.07(180.87-208.06)	139	193.70(180.54-207.68)	140	0.01(0.01,0.01)	-0.01(-0.01,-0.01)	-0.02(-0.02,-0.01)	-0.01(-0.01,-0.01)	157
Palestine	193.96(180.78-207.94)	140	193.50(180.39-207.46)	145	-0.02(-0.02,-0.02)	0.00(0.00,0.00)	-0.01(-0.01,-0.01)	-0.01(-0.01,-0.01)	158
Oman	190.06(177.31-203.60)	155	189.26(176.58-202.94)	154	0.06(0.05,0.07)	0.00(-0.04,0.04)	-0.08(-0.12,-0.03)	-0.01(-0.04,0.01)	159
Qatar	187.70(174.93-201.19)	157	185.91(172.89-199.35)	157	0.05(0.04,0.06)	-0.13(-0.16,-0.09)	-0.01(-0.02,0.00)	-0.03(-0.04,-0.02)	184
Saudi Arabia	191.02(178.07-204.70)	152	190.78(177.84-204.42)	152	0.02(0.02,0.03)	-0.02(-0.02,-0.02)	-0.02(-0.02,-0.01)	0.00(-0.01,0.00)	86
Syrian Arab Republic	193.37(180.23-207.28)	149	194.73(181.37-208.77)	137	-0.01(-0.01,-0.01)	-0.01(-0.01,-0.01)	0.10(0.08,0.11)	0.02(0.02,0.03)	20
Tunisia	193.63(180.45-207.59)	146	193.73(180.51-207.68)	139	0.00(0.00,0.00)	0.00(0.00,0.00)	0.00(0.00,0.00)	0.00(0.00,0.00)	87
Turkey	296.59(274.07-321.34)	81	297.01(274.88-320.57)	81	0.12(0.07,0.17)	0.00(-0.01,0.01)	-0.12(-0.24,-0.01)	0.00(-0.04,0.04)	88
United Arab Emirates	188.25(175.51-201.90)	156	188.10(175.35-202.07)	156	-0.03(-0.04,-0.03)	-0.09(-0.10,-0.08)	0.10(0.10,0.11)	0.00(-0.01,0.00)	89
Yemen	193.73(180.54-207.68)	143	193.70(180.54-207.63)	141	-0.01(-0.01,-0.01)	0.00(0.00,0.00)	0.00(0.00,0.00)	0.00(0.00,0.00)	90
Afghanistan	194.60(181.21-208.67)	138	193.35(180.25-207.23)	147	-0.04(-0.05,-0.04)	-0.02(-0.03,0.00)	-0.01(-0.01,-0.01)	-0.02(-0.03,-0.02)	181
Bangladesh	276.02(252.49-304.69)	87	276.77(253.20-305.57)	87	0.00(0.00,0.00)	0.01(0.01,0.01)	0.01(0.01,0.01)	0.01(0.01,0.01)	34
Bhutan	275.97(252.17-304.54)	88	276.01(252.37-304.78)	88	-0.01(-0.01,-0.01)	0.00(0.00,0.00)	0.01(0.01,0.01)	0.00(0.00,0.00)	91
India	277.62(261.03-295.57)	84	277.90(261.29-295.88)	84	0.16(0.15,0.16)	0.00(0.00,0.00)	-0.16(-0.17,-0.14)	0.00(0.00,0.00)	92
Nepal	276.61(252.98-305.31)	86	277.35(253.73-306.12)	86	0.00(0.00,0.00)	0.01(0.01,0.02)	0.01(0.01,0.01)	0.01(0.01,0.01)	35
Pakistan	277.24(260.68-295.20)	85	277.69(261.12-295.70)	85	0.01(0.01,0.01)	0.01(0.01,0.01)	0.00(0.00,0.00)	0.01(0.01,0.01)	36
Angola	159.26(146.57-173.73)	187	159.69(146.96-174.22)	179	0.02(0.02,0.02)	0.01(0.01,0.01)	0.00(0.00,0.00)	0.01(0.01,0.01)	37
Central African Republic	159.54(146.83-174.03)	184	159.61(146.89-174.13)	181	0.00(0.00,0.00)	0.00(0.00,0.00)	0.01(0.00,0.01)	0.00(0.00,0.00)	93
Congo	159.69(146.96-174.20)	179	159.45(146.75-173.96)	184	0.00(0.00,0.00)	-0.01(-0.01,-0.01)	-0.01(-0.01,0.00)	-0.01(-0.01,-0.01)	160
Democratic Republic of the Congo	159.51(146.80-173.97)	186	159.40(146.68-173.90)	186	0.00(0.00,0.00)	0.00(0.00,0.00)	0.00(0.00,0.00)	0.00(0.00,0.00)	94
Equatorial Guinea	159.63(146.91-174.21)	180	158.46(145.75-172.91)	187	-0.02(-0.02,-0.02)	-0.04(-0.04,-0.04)	-0.02(-0.02,-0.01)	-0.03(-0.03,-0.02)	185
Gabon	159.53(146.79-174.04)	185	159.68(146.96-174.28)	180	0.01(0.01,0.01)	0.00(0.00,0.00)	0.00(0.00,0.00)	0.00(0.00,0.00)	95
Burundi	153.50(143.87-163.99)	189	153.19(143.49-163.60)	192	0.00(0.00,0.00)	-0.01(-0.02,-0.01)	-0.01(-0.01,-0.01)	-0.01(-0.01,-0.01)	161
Comoros	153.22(143.56-163.60)	193	153.13(143.42-163.45)	193	0.00(0.00,0.00)	0.00(0.00,0.00)	0.00(0.00,0.00)	0.00(0.00,0.00)	96

Djibouti	152.32(142.71-162.61)	197	152.23(142.61-162.47)	198	-0.01(-0.01,0.00)	-0.01(-0.01,-0.01)	0.01(0.01,0.01)	0.00(0.00,0.00)	97
Eritrea	153.28(143.61-163.62)	192	153.21(143.51-163.57)	191	0.00(0.00,0.00)	0.00(0.00,0.00)	0.00(0.00,0.00)	0.00(0.00,0.00)	98
Ethiopia	140.33(132.76-148.08)	200	143.15(135.72-151.40)	200	-0.09(-0.22,0.04)	0.00(-0.04,0.04)	0.35(0.26,0.43)	0.09(0.03,0.14)	2
Kenya	134.88(128.11-141.75)	203	141.10(133.07-148.93)	201	0.01(0.00,0.01)	0.49(0.49,0.50)	0.01(0.01,0.01)	0.16(0.16,0.16)	1
Madagascar	153.13(143.46-163.51)	194	153.11(143.42-163.45)	194	0.00(0.00,0.00)	0.00(0.00,0.00)	0.00(0.00,0.00)	0.00(0.00,0.00)	99
Malawi	153.40(143.74-163.85)	190	153.49(143.84-163.96)	189	0.00(0.00,0.01)	0.01(0.01,0.01)	0.00(0.00,0.00)	0.00(0.00,0.00)	100
Mauritius	508.28(476.32-542.01)	23	508.54(476.73-542.41)	20	0.02(0.02,0.03)	-0.01(-0.02,0.00)	-0.01(-0.01,-0.01)	0.00(0.00,0.00)	101
Mozambique	153.56(143.92-164.01)	188	153.57(143.92-164.03)	188	0.00(0.00,0.00)	0.00(0.00,0.00)	0.00(0.00,0.00)	0.00(0.00,0.00)	102
Rwanda	112.10(104.29-120.53)	204	112.07(104.24-120.48)	204	-0.27(-0.29,-0.25)	-0.02(-0.02,-0.01)	0.26(0.25,0.28)	-0.01(-0.01,0.00)	162
Seychelles	507.79(475.82-541.71)	24	497.15(466.22-529.98)	26	0.02(0.01,0.02)	-0.17(-0.17,-0.16)	-0.05(-0.07,-0.04)	-0.07(-0.08,-0.07)	198
Somalia	152.73(143.07-163.02)	195	153.05(143.38-163.37)	195	0.01(0.01,0.01)	0.01(0.01,0.01)	0.00(0.00,0.00)	0.01(0.01,0.01)	38
United Republic of Tanzania	193.89(181.80-207.72)	141	192.08(180.71-204.44)	151	0.01(0.00,0.01)	-0.07(-0.08,-0.06)	0.01(0.00,0.01)	-0.03(-0.03,-0.03)	186
Uganda	193.57(179.16-209.74)	147	193.58(179.14-209.74)	142	0.15(0.14,0.16)	0.00(0.00,0.00)	-0.16(-0.18,-0.14)	0.00(-0.01,0.00)	103
Zambia	153.29(143.65-163.76)	191	153.28(143.59-163.67)	190	0.00(0.00,0.00)	0.00(0.00,0.00)	0.00(0.00,0.00)	0.00(0.00,0.00)	104
Botswana	159.84(147.11-174.43)	178	159.48(146.76-174.01)	183	-0.01(-0.01,-0.01)	-0.01(-0.01,-0.01)	-0.01(-0.01,-0.01)	-0.01(-0.01,-0.01)	163
Lesotho	159.59(146.87-174.12)	182	159.56(146.82-174.10)	182	0.00(0.00,0.00)	0.00(0.00,0.00)	-0.01(-0.01,-0.01)	0.00(0.00,0.00)	105
Namibia	159.60(146.89-174.12)	181	159.71(146.98-174.21)	178	0.01(0.01,0.01)	0.00(0.00,0.00)	0.00(0.00,0.00)	0.00(0.00,0.00)	106
South Africa	161.13(151.69-171.07)	176	160.89(151.45-170.77)	176	0.00(0.00,0.00)	-0.01(-0.01,-0.01)	-0.01(-0.01,-0.01)	-0.01(-0.01,0.00)	164
Eswatini	159.92(147.19-174.52)	177	159.44(146.72-174.04)	185	0.00(0.00,0.00)	-0.01(-0.01,-0.01)	-0.02(-0.02,-0.02)	-0.01(-0.01,-0.01)	165
Zimbabwe	159.58(146.90-174.13)	183	159.79(147.05-174.30)	177	0.01(0.01,0.01)	0.01(0.01,0.01)	0.00(0.00,0.00)	0.00(0.00,0.01)	107
Benin	161.81(152.44-171.81)	169	161.81(152.45-171.82)	165	0.00(0.00,0.00)	0.00(0.00,0.00)	0.00(0.00,0.00)	0.00(0.00,0.00)	108
Burkina Faso	162.01(152.61-172.03)	162	161.92(152.55-171.92)	162	0.00(-0.01,0.00)	0.00(0.00,0.00)	0.00(0.00,0.00)	0.00(0.00,0.00)	109
Cameroon	178.01(167.61-189.66)	158	178.59(168.86-188.79)	158	0.04(0.04,0.04)	0.00(0.00,0.00)	0.00(0.00,0.00)	0.01(0.01,0.01)	39
Cabo Verde	162.29(152.90-172.33)	161	161.63(152.37-171.60)	171	-0.01(-0.01,-0.01)	-0.02(-0.02,-0.02)	-0.01(-0.02,-0.01)	-0.01(-0.01,-0.01)	166
Chad	161.99(152.60-172.03)	163	161.61(152.26-171.66)	172	-0.01(-0.01,-0.01)	-0.01(-0.01,-0.01)	-0.01(-0.01,-0.01)	-0.01(-0.01,-0.01)	167
C??te d'Ivoire	161.31(152.05-171.29)	175	161.19(151.89-171.16)	175	0.00(0.00,0.00)	-0.01(-0.01,-0.01)	0.00(0.00,0.00)	0.00(0.00,0.00)	110
Gambia	161.60(152.27-171.64)	174	161.76(152.40-171.78)	166	0.01(0.00,0.01)	0.01(0.01,0.01)	0.00(0.00,0.00)	0.00(0.00,0.00)	111
Ghana	137.40(129.97-145.56)	202	137.55(130.12-145.70)	202	-0.67(-0.75,-0.58)	-0.03(-0.06,-0.01)	0.66(0.51,0.80)	0.00(-0.06,0.05)	112
Guinea	161.78(152.40-171.80)	170	161.84(152.46-171.88)	163	0.01(0.01,0.01)	0.00(0.00,0.00)	0.00(-0.01,0.00)	0.00(0.00,0.00)	113
Guinea-Bissau	161.98(152.59-172.01)	164	161.93(152.54-171.93)	161	0.00(0.00,0.00)	0.00(0.00,0.00)	0.00(0.00,0.00)	0.00(0.00,0.00)	114
Liberia	161.81(152.42-171.85)	168	161.44(152.12-171.42)	174	-0.02(-0.02,-0.01)	0.00(-0.02,0.01)	0.00(0.00,0.00)	-0.01(-0.01,0.00)	168
Mali	169.79(158.37-181.94)	159	169.38(158.28-181.56)	159	-0.01(-0.01,-0.01)	-0.02(-0.02,-0.01)	0.00(0.00,0.00)	-0.01(-0.01,-0.01)	169
Mauritania	161.68(152.34-171.68)	172	161.72(152.36-171.74)	168	0.00(0.00,0.00)	0.00(0.00,0.00)	0.00(0.00,0.00)	0.00(0.00,0.00)	115
Niger	161.62(152.27-171.65)	173	161.71(152.37-171.71)	169	-0.01(-0.01,-0.01)	0.01(0.01,0.01)	0.00(0.00,0.00)	0.00(0.00,0.00)	116
Nigeria	162.70(154.09-171.93)	160	162.92(154.44-171.98)	160	-0.03(-0.03,-0.03)	0.02(0.02,0.02)	0.03(0.03,0.03)	0.00(0.00,0.01)	117
Sao Tome and Principe	161.74(152.38-171.74)	171	161.64(152.34-171.62)	170	0.00(0.00,0.00)	0.00(0.00,0.00)	-0.01(-0.01,-0.01)	0.00(0.00,0.00)	118
Senegal	161.96(152.61-172.01)	165	161.53(152.19-171.49)	173	-0.01(-0.01,-0.01)	-0.01(-0.01,-0.01)	0.00(0.00,0.00)	-0.01(-0.01,-0.01)	170
Sierra Leone	161.86(152.49-171.92)	167	161.75(152.40-171.76)	167	0.01(0.01,0.01)	-0.01(-0.01,0.00)	-0.01(-0.01,-0.01)	0.00(0.00,0.00)	119

Togo	161.95(152.57-171.95)	166	161.84(152.45-171.79)	164	-0.01(-0.01,-0.01)	0.00(0.00,0.00)	0.00(0.00,0.00)	0.00(0.00,0.00)	120
American Samoa	485.85(452.48-518.28)	36	488.04(454.57-520.74)	33	-0.01(-0.01,-0.01)	0.04(0.04,0.04)	0.02(0.02,0.02)	0.02(0.02,0.02)	21
Bermuda	274.28(251.78-296.22)	96	274.11(251.67-296.00)	92	0.01(0.00,0.01)	-0.01(-0.01,-0.01)	0.00(0.00,0.00)	0.00(0.00,0.00)	121
Cook Islands	483.79(450.55-516.41)	42	494.60(460.37-527.80)	28	0.04(0.03,0.05)	0.09(0.08,0.09)	0.10(0.09,0.11)	0.08(0.07,0.08)	3
Greenland	241.78(228.32-255.65)	112	243.56(229.92-257.32)	112	0.04(0.03,0.05)	0.02(0.01,0.04)	0.01(0.01,0.02)	0.03(0.02,0.03)	13
Guam	477.16(444.60-509.58)	47	482.11(448.87-514.64)	44	0.17(0.15,0.18)	-0.02(-0.03,0.00)	-0.06(-0.06,-0.05)	0.03(0.03,0.04)	14
Monaco	447.99(414.38-484.25)	64	450.31(416.33-486.91)	54	-0.03(-0.03,-0.02)	0.04(0.03,0.04)	0.04(0.02,0.07)	0.02(0.01,0.03)	22
Nauru	484.94(451.47-517.16)	39	487.95(454.40-520.67)	34	0.01(0.00,0.01)	0.03(0.03,0.03)	0.03(0.02,0.03)	0.02(0.02,0.02)	23
Niue	484.46(451.07-517.13)	41	485.67(452.46-518.12)	37	0.00(0.00,0.01)	-0.03(-0.03,-0.02)	0.05(0.04,0.06)	0.01(0.00,0.01)	40
Northern Mariana Islands	482.87(449.83-514.58)	45	482.66(449.60-514.81)	43	0.34(0.16,0.52)	-0.34(-0.58,-0.09)	-0.12(-0.15,-0.09)	0.00(-0.11,0.11)	122
Palau	483.43(450.20-516.14)	44	470.61(438.50-501.80)	49	-0.16(-0.19,-0.13)	0.12(0.10,0.13)	-0.23(-0.25,-0.21)	-0.10(-0.11,-0.08)	199
Puerto Rico	274.08(251.58-295.97)	103	273.93(251.44-295.81)	95	0.00(0.00,0.00)	0.00(0.00,0.00)	0.00(0.00,0.00)	0.00(0.00,0.00)	123
Saint Kitts and Nevis	274.20(251.72-296.11)	100	273.91(251.52-295.79)	97	0.00(0.00,0.00)	0.00(0.00,0.00)	0.00(0.00,0.00)	0.00(0.00,0.00)	124
San Marino	449.47(415.61-485.78)	54	450.67(416.97-487.20)	53	0.02(0.02,0.02)	0.01(0.01,0.01)	0.00(0.00,0.00)	0.01(0.01,0.01)	41
Tokelau	489.08(455.22-521.99)	33	484.15(450.94-516.55)	42	0.05(0.02,0.07)	-0.17(-0.26,-0.09)	-0.02(-0.06,0.01)	-0.04(-0.07,0.00)	190
Tuvalu	496.46(462.35-529.86)	29	480.93(448.01-513.58)	45	-0.12(-0.13,-0.12)	-0.13(-0.13,-0.13)	-0.07(-0.08,-0.07)	-0.11(-0.11,-0.11)	201
United States Virgin Islands	274.39(251.91-296.33)	92	274.20(251.69-296.11)	91	0.00(0.00,0.00)	-0.01(-0.01,-0.01)	0.00(0.00,0.00)	0.00(0.00,0.00)	125
South Sudan	152.42(142.71-162.68)	196	152.92(143.25-163.21)	196	0.00(0.00,0.00)	0.01(0.01,0.01)	0.03(0.02,0.03)	0.01(0.01,0.01)	42
Sudan	193.64(180.39-207.58)	145	193.58(180.30-207.63)	143	-0.01(-0.01,0.00)	0.00(0.00,0.00)	0.00(0.00,0.00)	0.00(0.00,0.00)	126
**Number of incident cases**									
Asthma									
China	4,490,559(3,363,512-6,071,201)	1	3,761,277(2,901,080-4,917,886)	3	-1.84(-3.28,-0.37)	-2.93(-3.80,-2.06)	4.52(3.02,6.03)	-0.28(-1.09,0.54)	139
Democratic People's Republic of Korea	118,674(91,746-154,172)	47	125,030(104,366-149,850)	60	1.66(1.38,1.94)	-0.42(-0.68,-0.16)	-0.76(-0.89,-0.63)	0.08(-0.06,0.22)	120
Taiwan (Province of China)	109,542(89,621-138,262)	50	93,573(77,453-114,413)	70	1.31(0.98,1.65)	-0.83(-0.92,-0.74)	-2.43(-3.21,-1.65)	-0.67(-0.94,-0.40)	160
Cambodia	48,263(36,532-62,561)	83	69,235(54,268-89,011)	82	0.43(0.36,0.50)	0.98(0.93,1.03)	2.48(2.12,2.84)	1.25(1.14,1.37)	59
Indonesia	1,144,668(911,938-1,458,820)	5	1,309,435(1,067,022-1,619,267)	6	-0.44(-0.89,0.00)	2.03(1.76,2.30)	-0.07(-0.26,0.12)	0.42(0.23,0.60)	106
Lao People's Democratic Republic	21,011(16,474-26,343)	126	26,664(21,626-33,304)	120	1.46(1.38,1.55)	0.31(0.28,0.34)	0.76(0.62,0.90)	0.80(0.75,0.86)	82
Malaysia	89,348(72,675-110,475)	55	125,336(100,251-162,124)	59	2.26(2.18,2.34)	1.32(1.28,1.35)	0.18(0.04,0.32)	1.18(1.13,1.24)	61
Maldives	1,807(1,399-2,359)	173	1,802(1,385-2,325)	174	-0.49(-0.71,-0.27)	-1.30(-1.42,-1.19)	2.03(1.80,2.25)	-0.02(-0.13,0.10)	125
Myanmar	163,560(135,057-200,507)	38	187,024(158,584-224,646)	42	0.07(0.01,0.14)	0.24(0.21,0.26)	1.17(1.10,1.24)	0.47(0.43,0.50)	101
Philippines	700,810(541,729-932,067)	9	880,783(690,740-1,142,589)	7	0.64(0.49,0.79)	0.80(0.78,0.82)	0.92(0.79,1.06)	0.79(0.73,0.85)	84
Sri Lanka	86,653(71,565-105,206)	58	119,666(102,098-140,634)	61	1.03(1.01,1.04)	1.16(1.12,1.19)	1.17(1.12,1.23)	1.12(1.10,1.14)	65
Thailand	305,923(251,864-375,467)	22	294,343(245,694-351,332)	25	0.29(0.25,0.33)	-0.80(-1.03,-0.56)	0.46(0.18,0.73)	-0.17(-0.31,-0.03)	133
Timor-Leste	5,259(4,112-6,728)	154	7,320(5,804-9,235)	153	0.81(0.65,0.97)	0.91(0.84,0.98)	1.81(1.65,1.98)	1.13(1.05,1.22)	64
Viet Nam	316,948(242,714-415,096)	21	419,716(345,209-519,094)	17	2.11(1.67,2.55)	-0.76(-1.02,-0.49)	1.59(1.40,1.79)	0.99(0.79,1.19)	73
Fiji	5,226(4,294-6,363)	155	4,774(3,952-5,864)	161	-0.12(-0.24,0.00)	-1.17(-1.27,-1.07)	0.47(0.27,0.66)	-0.31(-0.39,-0.22)	142
Kiribati	706(587-846)	187	829(700-989)	183	0.79(0.73,0.85)	0.41(0.39,0.43)	0.48(0.42,0.54)	0.54(0.52,0.57)	97
Marshall Islands	317(249-399)	193	294(230-369)	194	-0.66(-0.72,-0.60)	-0.93(-0.99,-0.86)	1.14(0.59,1.70)	-0.21(-0.38,-0.03)	135

Micronesia (Federated States of)	696(549-860)	188	533(410-671)	188	-1.18(-1.28,-1.08)	-1.78(-1.88,-1.67)	0.35(-0.49,1.21)	-0.93(-1.20,-0.66)	173
Papua New Guinea	36,551(29,776-44,187)	97	71,436(59,528-85,258)	80	3.24(3.19,3.30)	1.58(1.44,1.72)	2.21(2.15,2.27)	2.32(2.27,2.38)	26
Samoa	992(781-1,250)	180	1,104(840-1,436)	179	-0.79(-1.09,-0.49)	-0.19(-0.30,-0.08)	2.40(2.03,2.78)	0.45(0.27,0.62)	104
Solomon Islands	2,291(1,763-2,898)	167	4,079(3,155-5,349)	163	1.68(0.94,2.42)	1.11(0.86,1.35)	3.65(3.15,4.16)	2.13(1.81,2.44)	34
Tonga	740(571-972)	185	665(507-862)	185	-0.19(-0.68,0.30)	-0.82(-0.99,-0.64)	0.12(-0.27,0.51)	-0.29(-0.51,-0.06)	140
Vanuatu	949(744-1,195)	181	1,674(1,182-2,220)	175	0.88(0.77,0.98)	0.88(0.77,0.98)	4.98(2.70,7.30)	2.13(1.43,2.84)	33
Armenia	11,792(8,825-15,398)	141	9,170(6,954-11,748)	146	-1.67(-1.83,-1.51)	-1.58(-1.77,-1.39)	0.90(0.68,1.13)	-0.82(-0.94,-0.70)	168
Azerbaijan	27,166(20,647-35,719)	112	31,120(24,185-39,603)	111	0.18(0.02,0.34)	-0.51(-0.71,-0.31)	2.04(1.84,2.24)	0.52(0.40,0.63)	99
Georgia	18,200(15,030-22,577)	129	10,669(8,371-13,595)	143	-2.53(-3.08,-1.98)	-2.31(-2.52,-2.09)	-0.05(-0.59,0.49)	-1.71(-1.98,-1.44)	193
Kazakhstan	46,707(35,961-59,441)	85	51,667(39,763-66,586)	90	-2.68(-2.87,-2.49)	0.30(0.14,0.46)	3.68(3.41,3.95)	0.41(0.28,0.54)	107
Kyrgyzstan	22,756(17,759-29,523)	123	27,312(20,367-36,254)	118	-0.39(-0.50,-0.28)	0.06(0.00,0.12)	2.27(2.16,2.39)	0.61(0.55,0.67)	92
Mongolia	9,204(6,939-12,103)	144	12,167(9,209-16,024)	137	-0.41(-0.45,-0.36)	0.09(-0.09,0.27)	3.46(3.16,3.76)	0.99(0.87,1.11)	74
Tajikistan	24,037(17,713-31,916)	117	37,084(27,174-49,748)	102	0.79(0.67,0.91)	0.74(0.54,0.94)	3.16(2.94,3.38)	1.55(1.44,1.66)	52
Turkmenistan	18,128(13,972-23,927)	130	17,965(13,165-24,197)	134	-0.66(-0.87,-0.45)	-1.31(-1.49,-1.13)	2.13(1.86,2.40)	-0.02(-0.16,0.13)	124
Uzbekistan	121,609(93,738-156,959)	45	156,519(121,361-202,861)	48	1.27(1.15,1.39)	-0.39(-0.50,-0.27)	1.87(1.68,2.06)	0.86(0.76,0.95)	77
Albania	13,983(11,277-17,833)	138	11,128(9,289-13,443)	141	-1.43(-1.79,-1.06)	-0.64(-1.49,0.22)	-0.49(-2.17,1.23)	-0.84(-1.47,-0.21)	169
Bosnia and Herzegovina	27,549(22,584-33,810)	110	18,567(15,591-22,069)	133	-1.55(-1.92,-1.19)	-1.24(-1.47,-1.00)	-1.63(-1.69,-1.58)	-1.44(-1.59,-1.29)	187
Bulgaria	47,566(39,626-56,988)	84	29,693(24,391-35,437)	116	-2.16(-2.50,-1.82)	-1.59(-1.68,-1.49)	-0.89(-1.17,-0.61)	-1.60(-1.75,-1.44)	192
Croatia	33,293(27,579-39,628)	102	21,661(18,388-25,717)	126	-1.51(-1.68,-1.34)	-1.94(-2.02,-1.87)	-0.85(-1.03,-0.68)	-1.49(-1.58,-1.40)	189
Czechia	43,446(34,824-53,283)	91	40,127(32,774-48,307)	99	-1.30(-1.74,-0.85)	-0.41(-0.57,-0.24)	1.25(0.88,1.62)	-0.22(-0.43,-0.01)	136
Hungary	52,953(43,611-63,131)	78	37,786(31,130-45,481)	101	-1.26(-1.65,-0.86)	-1.11(-1.59,-0.63)	-1.21(-1.46,-0.96)	-1.24(-1.46,-1.01)	186
North Macedonia	16,818(13,381-20,945)	133	14,379(12,304-16,608)	135	0.27(0.06,0.48)	-1.99(-2.19,-1.80)	0.08(-0.15,0.32)	-0.63(-0.77,-0.50)	156
Montenegro	2,984(2,334-3,744)	163	2,844(2,285-3,542)	166	-0.53(-0.57,-0.50)	-0.53(-0.57,-0.50)	0.89(0.21,1.57)	-0.10(-0.31,0.11)	130
Poland	404,892(337,325-483,495)	16	248,710(204,209-303,393)	33	-1.23(-1.97,-0.48)	-2.62(-2.77,-2.46)	-1.22(-1.33,-1.11)	-1.75(-1.99,-1.50)	195
Romania	137,397(112,170-169,647)	42	96,468(78,601-118,131)	69	-0.97(-1.13,-0.81)	-1.56(-1.65,-1.47)	-1.20(-1.36,-1.03)	-1.24(-1.33,-1.15)	185
Serbia	42,740(35,775-51,420)	92	34,347(28,904-40,784)	108	-0.44(-0.99,0.11)	-1.25(-1.36,-1.15)	-0.62(-0.78,-0.46)	-0.81(-0.99,-0.63)	167
Slovakia	22,833(18,404-28,240)	122	19,981(16,346-24,301)	132	-0.74(-0.99,-0.50)	-0.61(-0.69,-0.53)	0.14(-0.07,0.35)	-0.44(-0.55,-0.33)	148
Slovenia	14,056(11,860-16,795)	137	11,279(9,385-13,555)	140	-1.06(-1.24,-0.88)	-1.08(-1.16,-0.99)	-0.02(-0.12,0.08)	-0.77(-0.84,-0.69)	165
Belarus	70,196(57,661-85,639)	68	42,427(34,444-52,940)	97	-2.20(-2.40,-2.00)	-3.09(-3.22,-2.97)	0.30(0.09,0.51)	-1.74(-1.86,-1.63)	194
Estonia	6,300(5,202-7,513)	149	3,679(2,963-4,523)	165	-2.68(-2.83,-2.54)	-4.17(-4.48,-3.86)	1.29(0.80,1.78)	-1.85(-2.05,-1.64)	198
Latvia	15,608(13,271-18,346)	135	7,692(6,238-9,424)	152	-3.47(-3.56,-3.37)	-2.52(-2.55,-2.49)	-1.06(-1.14,-0.98)	-2.40(-2.45,-2.36)	203
Lithuania	16,534(13,646-19,801)	134	9,591(7,695-11,731)	145	-2.17(-2.25,-2.09)	-2.49(-2.91,-2.06)	-0.89(-1.26,-0.51)	-1.87(-2.06,-1.68)	199
Republic of Moldova	23,650(18,894-30,030)	119	12,857(10,117-16,200)	136	-2.36(-2.51,-2.22)	-2.97(-3.05,-2.88)	-0.79(-0.98,-0.60)	-2.08(-2.17,-1.99)	200
Russian Federation	977,005(798,115-1,207,341)	7	507,709(393,295-649,532)	16	-2.71(-2.93,-2.50)	-3.84(-3.97,-3.71)	0.07(-0.25,0.40)	-2.24(-2.39,-2.10)	202
Ukraine	324,089(253,447-407,276)	20	171,318(130,593-221,689)	45	-2.63(-2.94,-2.31)	-3.92(-4.16,-3.68)	0.44(-0.07,0.95)	-2.13(-2.36,-1.90)	201
Brunei Darussalam	1,621(1,266-2,082)	176	2,225(1,794-2,741)	170	1.90(1.81,1.99)	0.41(0.31,0.51)	1.12(1.06,1.19)	1.09(1.03,1.14)	70
Japan	1,125,260(945,376-1,347,003)	6	561,688(467,115-676,760)	12	-2.48(-2.74,-2.22)	-4.47(-4.80,-4.15)	-0.05(-0.28,0.17)	-2.50(-2.67,-2.32)	204
Republic of Korea	225,485(186,171-274,992)	31	211,096(177,800-250,593)	39	-0.29(-1.10,0.53)	-0.48(-0.62,-0.33)	0.13(-0.10,0.36)	-0.23(-0.50,0.04)	137

Singapore	18,885(15,740-22,491)	128	20,940(17,119-25,927)	131	0.85(0.51,1.18)	-0.43(-0.60,-0.25)	1.05(0.92,1.19)	0.41(0.26,0.57)	110
Australia	107,870(85,156-130,818)	51	115,111(91,823-143,929)	62	1.40(1.01,1.79)	-2.12(-2.36,-1.88)	1.21(1.04,1.38)	0.20(0.04,0.36)	117
New Zealand	30,039(23,956-37,292)	106	24,801(19,022-31,981)	122	-0.99(-1.14,-0.83)	-0.67(-0.83,-0.51)	-0.13(-0.18,-0.08)	-0.67(-0.75,-0.59)	161
Andorra	237(193-288)	196	294(237-357)	195	1.02(-0.12,2.17)	1.40(-0.66,3.51)	-0.50(-0.81,-0.19)	0.56(-0.20,1.32)	94
Austria	37,442(31,564-44,051)	96	31,023(25,355-37,384)	112	-0.71(-0.90,-0.51)	-0.87(-0.93,-0.81)	-0.18(-0.51,0.14)	-0.65(-0.77,-0.52)	157
Belgium	51,973(44,031-61,440)	80	38,372(30,877-46,541)	100	-3.08(-3.60,-2.57)	0.51(0.16,0.86)	-0.48(-0.76,-0.20)	-1.17(-1.41,-0.94)	184
Cyprus	4,223(3,409-5,168)	159	5,904(4,824-7,170)	157	0.58(0.43,0.73)	1.36(1.34,1.39)	1.52(1.45,1.59)	1.17(1.11,1.22)	62
Denmark	24,837(21,105-29,236)	114	21,389(17,118-26,111)	129	0.10(-0.24,0.43)	-0.66(-0.76,-0.56)	-0.92(-1.67,-0.16)	-0.51(-0.77,-0.25)	151
Finland	21,654(17,981-25,795)	124	21,422(17,782-25,614)	128	0.62(0.44,0.79)	0.23(0.18,0.27)	-0.97(-1.25,-0.70)	-0.03(-0.13,0.08)	126
France	358,433(306,546-423,696)	18	296,162(244,156-357,363)	24	-1.48(-1.83,-1.13)	-0.63(-0.68,-0.59)	0.06(-0.24,0.36)	-0.68(-0.83,-0.53)	162
Germany	426,768(350,281-492,725)	14	246,835(201,445-300,847)	35	-4.79(-5.04,-4.55)	-0.13(-0.46,0.21)	-0.85(-1.26,-0.44)	-1.83(-2.03,-1.64)	197
Greece	45,512(37,481-54,777)	87	36,458(29,722-43,718)	103	-0.63(-0.69,-0.57)	-0.33(-0.40,-0.25)	-1.39(-1.57,-1.22)	-0.76(-0.83,-0.70)	164
Iceland	2,045(1,646-2,502)	172	2,045(1,644-2,531)	172	0.18(-0.19,0.54)	-0.58(-0.74,-0.42)	0.35(0.07,0.64)	0.00(-0.18,0.17)	123
Ireland	24,567(20,137-29,875)	115	22,572(17,932-27,554)	125	-1.27(-1.40,-1.14)	0.64(0.48,0.80)	-0.37(-1.10,0.37)	-0.33(-0.57,-0.09)	143
Israel	26,386(20,991-32,364)	113	40,691(31,597-50,555)	98	1.23(1.12,1.34)	1.42(1.38,1.46)	1.99(1.92,2.07)	1.50(1.45,1.55)	53
Italy	305,423(255,392-360,502)	23	207,135(166,868-249,393)	40	-1.47(-2.16,-0.77)	-2.04(-2.32,-1.76)	-0.65(-0.92,-0.39)	-1.45(-1.74,-1.15)	188
Luxembourg	2,126(1,751-2,793)	170	2,627(2,167-3,167)	168	1.03(0.88,1.18)	-0.04(-0.09,0.01)	1.34(1.06,1.62)	0.72(0.62,0.82)	87
Malta	2,268(1,908-2,665)	168	1,954(1,612-2,344)	173	-0.23(-0.40,-0.05)	-0.88(-0.91,-0.86)	-0.39(-0.58,-0.21)	-0.53(-0.61,-0.45)	153
Netherlands	49,068(41,428-57,168)	82	49,024(40,777-57,277)	93	0.26(0.21,0.30)	-1.36(-1.56,-1.16)	1.22(1.16,1.28)	0.00(-0.07,0.07)	122
Norway	35,454(29,475-42,022)	100	28,099(22,567-34,592)	117	-0.82(-1.57,-0.07)	-1.23(-1.34,-1.12)	-0.23(-0.60,0.13)	-0.79(-1.06,-0.53)	166
Portugal	70,880(58,660-86,166)	67	54,441(45,601-63,883)	86	-1.49(-2.01,-0.96)	0.45(0.15,0.76)	-1.52(-1.73,-1.32)	-1.01(-1.23,-0.79)	178
Spain	164,254(134,983-198,520)	37	173,292(142,545-207,590)	44	-1.26(-1.37,-1.16)	2.00(1.87,2.14)	-0.27(-0.62,0.09)	0.18(0.05,0.30)	118
Sweden	69,111(57,306-81,250)	69	52,430(42,260-64,149)	88	-3.29(-3.57,-3.00)	-1.39(-1.99,-0.80)	1.45(-0.09,3.00)	-1.15(-1.68,-0.62)	182
Switzerland	37,615(31,627-44,284)	95	36,015(29,402-43,182)	104	-0.36(-0.41,-0.31)	-1.02(-1.05,-0.99)	0.90(0.80,1.00)	-0.17(-0.21,-0.13)	134
United Kingdom	488,938(403,446-586,816)	11	415,532(338,537-506,097)	18	-1.17(-1.83,-0.50)	-0.49(-0.59,-0.38)	-0.12(-0.76,0.52)	-0.58(-0.88,-0.29)	154
Argentina	258,500(214,737-317,352)	30	326,712(263,862-406,380)	21	0.95(0.78,1.12)	0.97(0.93,1.02)	0.45(0.37,0.54)	0.81(0.74,0.87)	81
Chile	86,681(70,648-107,853)	57	105,914(86,981-129,139)	64	0.94(0.69,1.19)	-0.66(-0.89,-0.43)	2.02(1.53,2.51)	0.72(0.52,0.93)	85
Uruguay	23,194(19,260-28,100)	121	21,081(17,308-25,812)	130	-0.20(-0.36,-0.03)	-0.78(-0.90,-0.65)	0.07(-0.05,0.19)	-0.34(-0.42,-0.25)	144
Canada	201,721(164,225-248,004)	32	222,741(183,176-270,681)	36	0.47(0.09,0.85)	0.94(0.82,1.06)	-0.30(-0.93,0.33)	0.41(0.17,0.64)	108
United States of America	3,166,265(2,518,593-4,064,450)	3	4,143,124(3,475,553-4,948,182)	2	-2.24(-2.72,-1.77)	3.35(2.96,3.75)	1.44(1.14,1.75)	1.01(0.76,1.27)	72
Antigua and Barbuda	566(437-743)	189	611(474-769)	186	1.61(1.22,1.99)	-0.22(-0.33,-0.12)	-0.47(-0.74,-0.20)	0.32(0.16,0.48)	113
Bahamas	2,410(1,866-3,139)	165	2,711(2,100-3,430)	167	1.85(1.62,2.07)	0.22(0.01,0.44)	-0.55(-0.73,-0.37)	0.45(0.34,0.57)	103
Barbados	2,226(1,794-2,781)	169	2,186(1,771-2,699)	171	-0.43(-0.59,-0.28)	1.06(0.97,1.15)	-0.70(-0.76,-0.63)	-0.07(-0.13,-0.01)	129
Belize	2,423(1,841-3,105)	164	3,835(2,948-4,888)	164	1.31(0.81,1.81)	2.38(2.03,2.74)	1.24(1.07,1.41)	1.60(1.39,1.81)	51
Cuba	105,398(82,977-132,494)	52	80,767(64,553-99,384)	73	-0.99(-1.07,-0.92)	-1.36(-1.68,-1.05)	-0.55(-0.91,-0.19)	-0.95(-1.10,-0.79)	176
Dominica	790(621-1,002)	184	569(454-708)	187	-0.44(-0.51,-0.37)	-1.19(-1.44,-0.94)	-1.60(-1.74,-1.45)	-1.09(-1.18,-0.99)	180
Dominican Republic	64,526(49,923-81,246)	71	74,999(58,204-96,702)	77	-0.65(-0.86,-0.43)	1.07(0.81,1.34)	1.47(1.09,1.85)	0.64(0.47,0.82)	91
Grenada	1,052(795-1,363)	179	915(728-1,162)	181	0.39(0.23,0.56)	-0.77(-0.81,-0.72)	-1.07(-1.19,-0.96)	-0.48(-0.55,-0.41)	149

Guyana	8,881(6,955-11,276)	145	7,207(5,663-9,087)	154	-0.04(-0.20,0.12)	-1.91(-2.02,-1.80)	-0.06(-0.31,0.19)	-0.69(-0.80,-0.59)	163
Haiti	102,139(79,276-126,979)	53	150,751(119,070-188,223)	50	1.59(1.45,1.73)	1.05(1.01,1.09)	1.51(1.40,1.62)	1.36(1.30,1.42)	56
Jamaica	28,627(22,099-36,890)	108	21,612(16,953-27,391)	127	-0.10(-0.33,0.13)	-1.66(-1.77,-1.55)	-0.91(-1.08,-0.73)	-0.93(-1.04,-0.82)	174
Saint Lucia	1,693(1,315-2,153)	174	1,308(1,048-1,603)	178	0.29(0.18,0.41)	-1.53(-1.66,-1.41)	-1.29(-1.41,-1.17)	-0.88(-0.96,-0.79)	170
Saint Vincent and the Grenadines	1,205(926-1,563)	177	864(678-1,093)	182	-1.26(-1.29,-1.23)	-1.35(-1.39,-1.31)	-0.64(-0.94,-0.34)	-1.11(-1.20,-1.01)	181
Suriname	3,862(2,965-5,061)	160	4,616(3,572-5,908)	162	0.33(-0.03,0.68)	0.94(0.82,1.07)	0.61(0.51,0.71)	0.66(0.52,0.81)	89
Trinidad and Tobago	9,769(7,559-12,586)	143	8,338(6,447-10,735)	151	-1.89(-1.98,-1.81)	-0.89(-0.93,-0.85)	1.34(0.98,1.70)	-0.52(-0.63,-0.40)	152
Bolivia (Plurinational State of)	85,462(63,523-109,707)	60	106,608(79,183-144,003)	63	0.91(0.73,1.09)	-0.39(-0.48,-0.30)	2.06(1.84,2.28)	0.80(0.69,0.90)	83
Ecuador	115,768(85,198-151,654)	48	127,879(93,983-172,623)	58	-0.34(-0.74,0.07)	-0.22(-0.38,-0.06)	1.83(1.69,1.97)	0.36(0.19,0.52)	112
Peru	271,620(199,167-355,757)	27	249,734(185,262-337,734)	32	-0.38(-0.50,-0.25)	-2.15(-2.44,-1.86)	1.98(1.85,2.11)	-0.31(-0.45,-0.16)	141
Colombia	283,260(216,120-366,723)	25	256,255(193,451-332,665)	30	0.15(-0.15,0.44)	-0.59(-0.69,-0.49)	-0.45(-0.73,-0.17)	-0.36(-0.50,-0.22)	145
Costa Rica	35,127(26,816-44,964)	101	34,390(26,692-43,945)	107	2.25(2.16,2.34)	-2.07(-2.31,-1.84)	-0.22(-0.33,-0.12)	-0.06(-0.16,0.03)	128
El Salvador	80,440(60,576-103,848)	62	51,853(40,329-67,188)	89	-0.71(-0.90,-0.52)	-2.91(-3.04,-2.78)	-0.61(-0.74,-0.47)	-1.53(-1.63,-1.43)	190
Guatemala	100,926(75,835-128,430)	54	105,272(77,884-140,749)	66	0.52(0.23,0.82)	-1.08(-1.73,-0.42)	0.98(0.89,1.07)	0.11(-0.13,0.36)	119
Honduras	62,863(46,702-81,126)	72	73,991(55,648-97,341)	78	1.19(0.92,1.46)	-0.28(-0.35,-0.21)	0.98(0.70,1.27)	0.56(0.44,0.69)	95
Mexico	617,319(455,781-841,052)	10	606,000(433,053-811,132)	10	-1.01(-1.35,-0.68)	-0.09(-0.38,0.19)	1.30(1.21,1.39)	-0.04(-0.20,0.13)	127
Nicaragua	52,518(39,045-68,940)	79	44,738(33,360-59,048)	96	-0.57(-0.83,-0.31)	-0.78(-0.99,-0.57)	-0.30(-0.49,-0.10)	-0.60(-0.73,-0.47)	155
Panama	23,974(18,619-30,295)	118	30,412(23,119-39,435)	115	0.95(0.82,1.07)	0.35(0.27,0.43)	1.30(1.17,1.43)	0.83(0.76,0.90)	80
Venezuela (Bolivarian Republic of)	179,203(135,096-234,165)	35	169,708(128,442-218,919)	46	0.59(0.29,0.88)	-0.08(-0.17,0.01)	-1.22(-1.48,-0.95)	-0.27(-0.42,-0.13)	138
Brazil	1,797,924(1,305,007-2,425,965)	4	1,623,152(1,165,808-2,128,603)	4	-0.44(-0.68,-0.19)	-1.28(-1.34,-1.22)	0.77(0.49,1.04)	-0.39(-0.50,-0.27)	146
Paraguay	43,769(31,879-59,231)	89	63,305(46,892-84,222)	83	1.93(1.82,2.04)	0.95(0.92,0.99)	0.95(0.92,0.99)	1.26(1.22,1.30)	58
Algeria	146,771(114,676-189,342)	40	218,593(170,849-280,218)	37	0.72(0.66,0.78)	0.72(0.66,0.78)	2.78(1.87,3.71)	1.36(1.07,1.64)	55
Bahrain	3,234(2,538-4,109)	162	6,166(4,904-7,614)	156	1.95(1.81,2.09)	4.15(3.93,4.38)	0.76(0.61,0.91)	2.21(2.11,2.31)	29
Egypt	372,153(287,409-474,733)	17	566,303(441,856-720,245)	11	0.47(0.40,0.54)	1.44(1.37,1.51)	2.53(2.35,2.72)	1.47(1.39,1.54)	54
Iran (Islamic Republic of)	433,579(323,087-565,952)	13	415,328(327,426-524,205)	19	-0.05(-0.11,0.01)	-1.15(-1.21,-1.09)	0.92(0.69,1.15)	-0.13(-0.21,-0.06)	131
Iraq	145,551(110,802-186,951)	41	248,615(190,297-326,436)	34	3.47(3.13,3.82)	0.91(0.28,1.54)	1.30(1.19,1.41)	1.88(1.65,2.12)	43
Jordan	28,502(21,329-38,171)	109	76,042(57,525-98,883)	75	1.82(1.74,1.91)	3.85(3.34,4.37)	4.91(4.76,5.06)	3.47(3.28,3.65)	6
Kuwait	11,941(9,206-15,130)	140	23,469(18,102-29,633)	123	-0.67(-1.00,-0.34)	3.91(3.49,4.32)	3.87(3.67,4.07)	2.38(2.19,2.56)	23
Lebanon	24,091(18,971-30,902)	116	33,802(26,857-42,067)	110	0.19(0.08,0.30)	0.46(0.13,0.79)	2.85(2.01,3.70)	1.11(0.82,1.41)	66
Libya	30,503(22,889-39,126)	104	34,125(27,520-41,641)	109	0.36(0.27,0.45)	0.78(0.69,0.86)	0.12(-0.03,0.27)	0.41(0.35,0.48)	109
Morocco	127,525(99,425-160,957)	43	166,275(133,121-205,765)	47	1.17(1.11,1.24)	0.64(0.61,0.67)	1.03(0.93,1.12)	0.93(0.89,0.96)	76
Palestine	14,335(10,683-18,994)	136	30,541(22,825-40,046)	114	2.74(2.38,3.10)	3.05(2.97,3.13)	2.18(1.97,2.39)	2.68(2.55,2.82)	16
Oman	11,310(8,635-14,877)	142	25,413(19,975-32,469)	121	0.69(0.50,0.88)	2.29(1.62,2.96)	5.25(4.30,6.20)	2.77(2.40,3.14)	15
Qatar	2,356(1,795-3,106)	166	11,544(9,156-14,313)	139	2.52(2.35,2.69)	6.86(6.02,7.72)	7.58(7.27,7.90)	5.68(5.38,5.97)	1
Saudi Arabia	71,233(53,674-92,870)	66	132,867(105,713-165,421)	55	1.93(1.80,2.06)	2.55(1.97,3.13)	1.95(1.61,2.30)	2.11(1.90,2.33)	35
Syrian Arab Republic	85,909(66,729-110,565)	59	76,301(60,880-93,829)	74	-0.04(-0.51,0.44)	3.70(2.80,4.60)	-6.12(-7.76,-4.46)	-0.66(-1.31,0.00)	159
Tunisia	51,650(40,706-66,023)	81	60,869(49,618-74,973)	85	0.82(0.76,0.88)	-0.14(-0.21,-0.08)	1.11(0.94,1.28)	0.58(0.52,0.64)	93
Turkey	463,869(371,308-575,459)	12	515,559(435,559-611,836)	15	1.02(0.82,1.23)	-1.80(-2.36,-1.23)	2.46(2.22,2.71)	0.42(0.19,0.65)	105

United Arab Emirates	16,920(13,464-21,031)	132	62,279(50,671-76,301)	84	4.44(4.00,4.87)	8.84(7.94,9.76)	-0.42(-1.21,0.38)	4.43(3.96,4.89)	2
Yemen	114,730(88,261-148,175)	49	214,371(161,770-277,934)	38	2.09(2.00,2.18)	2.28(2.25,2.30)	2.19(2.05,2.32)	2.18(2.13,2.24)	31
Afghanistan	84,606(67,675-104,961)	61	281,631(217,451-363,785)	27	5.99(3.81,8.22)	3.40(3.26,3.54)	3.40(3.26,3.54)	4.20(3.52,4.88)	3
Bangladesh	264,877(220,069-322,695)	29	312,711(266,091-366,796)	22	1.26(1.03,1.49)	-0.43(-0.53,-0.33)	0.99(0.53,1.45)	0.53(0.37,0.70)	98
Bhutan	1,650(1,374-1,995)	175	1,483(1,276-1,752)	176	0.21(-0.50,0.93)	-0.96(-1.29,-0.64)	-0.37(-0.49,-0.25)	-0.41(-0.67,-0.15)	147
India	3,251,059(2,645,599-4,094,151)	2	4,533,398(3,732,737-5,478,018)	1	-3.16(-4.38,-1.92)	5.58(3.71,7.48)	0.74(-0.06,1.55)	0.99(0.24,1.74)	75
Nepal	35,563(29,868-42,996)	99	48,737(41,233-57,616)	94	1.38(1.27,1.48)	-0.16(-0.33,0.02)	2.28(1.94,2.63)	1.11(0.98,1.24)	68
Pakistan	348,466(276,260-442,692)	19	541,633(435,288-685,684)	13	2.50(1.08,3.94)	1.43(1.26,1.60)	1.43(1.26,1.60)	1.76(1.31,2.21)	48
Angola	89,170(69,442-112,095)	56	201,117(151,493-261,730)	41	3.39(3.37,3.42)	2.58(2.55,2.61)	2.55(2.47,2.63)	2.85(2.82,2.88)	14
Central African Republic	21,634(17,002-27,150)	125	34,817(27,207-44,222)	105	2.23(2.19,2.28)	1.69(1.64,1.74)	0.97(0.81,1.13)	1.65(1.60,1.71)	49
Congo	19,417(15,005-24,815)	127	34,497(26,347-44,766)	106	1.90(1.80,2.00)	1.70(1.58,1.82)	2.51(2.43,2.59)	2.00(1.94,2.07)	39
Democratic Republic of the Congo	275,152(206,903-355,149)	26	530,403(407,988-686,805)	14	2.06(2.03,2.09)	2.41(2.34,2.48)	2.44(2.37,2.50)	2.29(2.26,2.32)	27
Equatorial Guinea	3,421(2,598-4,363)	161	8,388(6,272-11,071)	150	3.69(3.47,3.91)	2.93(2.83,3.02)	2.94(2.73,3.15)	3.19(3.08,3.30)	9
Gabon	6,407(4,950-8,184)	148	8,875(6,767-11,466)	147	1.17(1.15,1.19)	0.71(0.69,0.72)	1.59(1.45,1.74)	1.14(1.09,1.18)	63
Burundi	60,962(46,800-78,156)	73	101,676(76,461-130,733)	67	0.40(0.18,0.62)	2.89(2.59,3.19)	2.30(1.57,3.05)	1.86(1.59,2.12)	45
Comoros	4,337(3,328-5,602)	158	4,947(3,835-6,291)	159	0.44(0.39,0.48)	0.27(0.25,0.29)	0.71(0.66,0.75)	0.46(0.43,0.48)	102
Djibouti	4,977(3,778-6,448)	156	8,674(6,777-11,283)	149	1.17(0.90,1.44)	1.96(1.66,2.27)	2.59(2.51,2.67)	1.95(1.82,2.09)	40
Eritrea	29,371(22,967-37,337)	107	52,549(40,268-67,740)	87	2.98(2.76,3.21)	1.85(1.72,1.98)	1.29(1.13,1.46)	2.05(1.92,2.18)	37
Ethiopia	408,660(308,529-542,179)	15	689,033(494,291-938,485)	8	1.45(1.40,1.49)	1.45(1.40,1.49)	2.76(2.47,3.05)	1.85(1.76,1.95)	47
Kenya	167,476(121,357-228,150)	36	280,521(205,184-377,311)	28	1.43(1.14,1.72)	1.71(1.57,1.86)	2.47(2.17,2.77)	1.85(1.69,2.01)	46
Madagascar	195,727(153,362-255,113)	34	273,651(213,853-344,796)	29	2.05(1.88,2.22)	0.21(0.01,0.40)	1.29(1.16,1.42)	1.10(1.00,1.21)	69
Malawi	80,387(60,369-104,319)	63	137,164(103,069-178,788)	54	0.54(0.12,0.95)	2.20(2.17,2.24)	2.77(2.63,2.91)	1.86(1.72,2.00)	44
Mauritius	6,436(5,210-7,887)	147	4,778(3,979-5,700)	160	0.05(-0.03,0.12)	-1.49(-1.52,-1.46)	-1.51(-1.63,-1.39)	-1.03(-1.08,-0.98)	179
Mozambique	120,725(92,612-156,418)	46	255,838(192,943-337,370)	31	2.79(2.19,3.39)	2.05(1.97,2.13)	3.02(2.90,3.13)	2.61(2.41,2.80)	17
Rwanda	122,363(95,242-159,159)	44	150,237(116,676-191,043)	51	-0.58(-3.54,2.48)	1.31(0.78,1.83)	0.22(-0.55,1.01)	0.38(-0.61,1.38)	111
Seychelles	312(247-396)	194	375(303-473)	191	0.07(-0.06,0.21)	0.50(0.47,0.53)	1.42(1.31,1.54)	0.65(0.60,0.71)	90
Somalia	76,901(57,992-98,036)	65	177,550(137,118-231,482)	43	3.00(2.81,3.19)	2.53(2.45,2.62)	3.45(3.37,3.54)	2.97(2.89,3.04)	10
United Republic of Tanzania	296,461(222,485-389,370)	24	612,808(460,356-799,475)	9	2.41(2.27,2.56)	3.46(3.31,3.61)	1.80(1.51,2.09)	2.53(2.41,2.65)	18
Uganda	198,022(149,950-262,233)	33	398,032(292,298-528,517)	20	2.87(2.82,2.91)	2.19(2.14,2.24)	2.40(2.33,2.47)	2.46(2.42,2.49)	20
Zambia	43,743(32,947-57,110)	90	97,999(72,167-129,647)	68	0.90(0.56,1.25)	3.08(2.92,3.23)	4.84(4.49,5.19)	2.91(2.72,3.09)	13
Botswana	5,508(4,222-6,982)	153	10,019(7,506-13,328)	144	1.88(1.55,2.21)	1.89(1.00,2.79)	2.21(1.96,2.45)	1.94(1.63,2.26)	41
Lesotho	4,832(3,769-6,091)	157	5,123(4,175-6,222)	158	-0.15(-0.33,0.02)	-0.39(-0.49,-0.28)	1.30(1.14,1.46)	0.22(0.13,0.32)	116
Namibia	5,942(4,641-7,543)	152	8,738(6,746-11,092)	148	2.17(1.85,2.48)	0.44(0.37,0.51)	1.66(1.51,1.81)	1.35(1.24,1.46)	57
South Africa	265,161(189,704-352,315)	28	302,856(216,857-402,989)	23	2.50(1.58,3.44)	-6.54(-9.21,-3.78)	8.11(6.84,9.39)	1.06(-0.01,2.16)	71
Eswatini	6,074(4,670-7,906)	150	7,142(5,569-8,859)	155	2.49(1.88,3.11)	-0.37(-0.43,-0.30)	-0.37(-0.43,-0.30)	0.51(0.32,0.71)	100
Zimbabwe	36,369(27,368-47,580)	98	51,492(40,122-65,729)	91	0.35(0.28,0.42)	0.70(0.56,0.83)	2.82(2.68,2.97)	1.24(1.16,1.32)	60
Benin	31,332(23,291-41,543)	103	72,418(52,917-96,381)	79	2.49(2.29,2.69)	2.79(2.73,2.85)	3.68(3.54,3.82)	2.95(2.86,3.04)	11
Burkina Faso	56,974(42,012-75,020)	75	131,075(95,954-177,974)	57	2.07(2.01,2.14)	3.07(2.90,3.24)	3.71(3.60,3.81)	2.92(2.85,2.98)	12

Cameroon	54,195(41,387-71,560)	77	140,046(102,838-188,093)	53	2.36(2.29,2.43)	4.18(4.08,4.28)	3.69(3.53,3.85)	3.37(3.30,3.43)	7
Cabo Verde	2,069(1,535-2,718)	171	2,413(1,853-3,146)	169	0.93(0.82,1.04)	-0.19(-0.30,-0.08)	1.03(0.89,1.18)	0.54(0.47,0.62)	96
Chad	30,051(22,466-39,400)	105	85,256(61,631-116,071)	71	2.67(2.61,2.73)	3.96(3.85,4.07)	4.49(4.37,4.61)	3.67(3.62,3.73)	4
C??te d'Ivoire	78,070(58,852-104,186)	64	155,984(117,239-206,990)	49	3.24(2.85,3.64)	1.70(1.56,1.85)	2.38(2.06,2.70)	2.44(2.26,2.63)	21
Gambia	6,019(4,441-8,039)	151	12,141(9,052-15,846)	138	2.14(2.00,2.28)	2.41(2.37,2.46)	2.94(2.81,3.07)	2.46(2.40,2.53)	19
Ghana	68,100(49,502-91,455)	70	131,817(98,899-172,299)	56	1.60(1.52,1.68)	2.47(2.38,2.56)	2.96(2.83,3.09)	2.34(2.28,2.40)	25
Guinea	43,848(33,516-58,527)	88	84,133(63,528-113,570)	72	2.88(2.70,3.07)	1.06(0.94,1.18)	2.94(2.73,3.15)	2.21(2.10,2.32)	28
Guinea-Bissau	6,749(5,164-8,734)	146	10,781(8,131-13,898)	142	1.37(1.25,1.48)	1.34(1.27,1.40)	2.24(2.14,2.34)	1.62(1.56,1.68)	50
Liberia	12,282(9,074-16,322)	139	23,331(17,080-31,164)	124	2.52(0.77,4.29)	2.04(1.91,2.17)	2.04(1.91,2.17)	2.19(1.64,2.74)	30
Mali	39,006(29,773-51,662)	94	105,829(78,936-142,381)	65	1.93(1.17,2.69)	3.85(3.75,3.96)	4.82(4.39,5.25)	3.55(3.27,3.83)	5
Mauritania	17,786(13,300-23,725)	131	30,696(23,255-40,683)	113	1.97(1.92,2.02)	1.93(1.91,1.96)	1.81(1.77,1.85)	1.89(1.87,1.91)	42
Niger	57,784(43,121-76,310)	74	145,970(106,775-198,158)	52	2.75(2.70,2.80)	3.25(3.15,3.35)	3.81(3.72,3.90)	3.24(3.19,3.29)	8
Nigeria	758,132(570,645-1,004,524)	8	1,524,454(1,132,369-2,056,074)	5	2.16(1.83,2.49)	3.18(2.86,3.50)	2.11(2.01,2.20)	2.39(2.24,2.55)	22
Sao Tome and Principe	1,082(832-1,390)	178	1,329(1,039-1,658)	177	1.50(1.18,1.83)	-0.09(-0.17,0.00)	0.67(0.58,0.77)	0.67(0.56,0.78)	88
Senegal	39,404(29,838-52,202)	93	70,251(52,236-93,382)	81	1.34(1.18,1.50)	1.99(1.93,2.05)	2.85(2.71,2.98)	2.03(1.95,2.11)	38
Sierra Leone	23,486(17,886-30,941)	120	45,585(34,248-60,200)	95	0.83(0.60,1.07)	3.23(2.90,3.55)	2.79(2.55,3.03)	2.35(2.18,2.53)	24
Togo	27,408(20,326-36,541)	111	50,610(37,927-66,537)	92	2.29(2.11,2.47)	1.71(1.54,1.87)	2.58(2.47,2.70)	2.15(2.06,2.25)	32
American Samoa	318(240-420)	192	314(235-407)	193	0.03(-0.15,0.21)	0.03(-0.15,0.21)	0.63(-1.85,3.17)	0.22(-0.57,1.00)	115
Bermuda	430(339-542)	190	349(275-430)	192	0.01(-0.09,0.11)	-0.48(-0.69,-0.27)	-1.57(-2.02,-1.11)	-0.66(-0.82,-0.49)	158
Cook Islands	131(100-174)	197	98(72-125)	199	-2.50(-2.88,-2.13)	-0.48(-0.67,-0.29)	0.31(-0.12,0.74)	-0.90(-1.12,-0.68)	171
Greenland	707(563-882)	186	513(428-625)	189	0.25(-0.46,0.97)	-2.43(-2.62,-2.24)	-1.08(-1.17,-0.98)	-1.17(-1.41,-0.94)	183
Guam	893(675-1,176)	183	940(704-1,224)	180	0.82(0.56,1.08)	-1.26(-1.62,-0.90)	1.37(0.87,1.87)	0.30(0.06,0.53)	114
Monaco	111(92-134)	198	136(111-164)	197	0.69(0.66,0.72)	0.72(0.66,0.79)	0.77(0.72,0.82)	0.72(0.69,0.75)	86
Nauru	68(52-88)	201	63(47-84)	202	-1.21(-1.29,-1.13)	-1.21(-1.29,-1.13)	2.28(0.65,3.94)	-0.14(-0.64,0.37)	132
Niue	15(11-20)	203	9(7-12)	203	-4.84(-5.68,-4.00)	-2.01(-2.32,-1.71)	2.24(1.54,2.95)	-1.60(-1.99,-1.20)	191
Northern Mariana Islands	276(214-354)	195	200(151-254)	196	2.02(1.66,2.38)	-2.46(-2.64,-2.29)	-2.88(-3.25,-2.51)	-1.01(-1.21,-0.81)	177
Palau	98(77-122)	200	82(66-100)	200	0.91(0.70,1.12)	-1.91(-2.10,-1.71)	-0.32(-0.69,0.04)	-0.48(-0.64,-0.31)	150
Puerto Rico	45,637(35,711-56,881)	86	26,855(21,738-33,087)	119	0.07(-0.11,0.25)	-1.96(-2.19,-1.72)	-3.50(-3.64,-3.35)	-1.79(-1.93,-1.66)	196
Saint Kitts and Nevis	428(325-552)	191	419(325-538)	190	0.10(0.05,0.15)	0.10(0.05,0.15)	-0.12(-0.80,0.56)	0.00(-0.22,0.22)	121
San Marino	99(80-122)	199	125(100-153)	198	0.92(0.82,1.02)	0.97(0.94,1.00)	0.59(0.55,0.63)	0.83(0.79,0.87)	79
Tokelau	12(8-16)	204	9(7-12)	204	-3.14(-4.65,-1.60)	-2.73(-3.42,-2.05)	2.99(2.44,3.54)	-0.95(-1.52,-0.38)	175
Tuvalu	53(42-66)	202	66(49-85)	201	-1.22(-1.43,-1.01)	-0.22(-0.30,-0.14)	4.28(3.34,5.22)	0.84(0.55,1.13)	78
United States Virgin Islands	895(693-1,168)	182	684(537-863)	184	-0.49(-0.54,-0.43)	-1.30(-1.33,-1.28)	-0.87(-1.07,-0.67)	-0.91(-0.98,-0.85)	172
South Sudan	55,084(42,534-71,416)	76	75,804(57,146-98,742)	76	1.34(1.11,1.58)	1.79(1.74,1.85)	0.04(-0.60,0.67)	1.11(0.89,1.32)	67
Sudan	155,407(114,968-203,710)	39	284,502(217,381-375,039)	26	2.97(2.84,3.09)	1.39(1.24,1.54)	2.10(1.93,2.28)	2.09(2.00,2.18)	36
Inflammatory bowel disease									
China	17,221(14,326-20,399)	2	51,462(43,933-60,474)	2	5.64(5.22,6.05)	3.22(3.10,3.34)	2.76(2.61,2.90)	3.82(3.68,3.96)	30
Democratic People's Republic of Korea	197(164-236)	75	387(326-463)	75	3.36(2.76,3.96)	1.95(1.92,1.98)	1.95(1.92,1.98)	2.38(2.20,2.57)	116

Taiwan (Province of China)	149(129-171)	85	470(420-526)	69	2.30(1.67,2.94)	7.86(6.32,9.43)	2.87(2.57,3.16)	4.19(3.67,4.73)	22
Cambodia	30(23-37)	138	79(64-96)	134	3.44(3.38,3.49)	3.82(3.57,4.07)	2.84(2.75,2.93)	3.38(3.29,3.46)	63
Indonesia	681(551-828)	39	1,504(1,248-1,808)	31	3.91(3.60,4.23)	2.34(2.28,2.39)	2.16(1.99,2.32)	2.77(2.65,2.88)	91
Lao People's Democratic Republic	12(10-15)	156	35(29-43)	151	3.66(3.60,3.72)	3.46(3.44,3.47)	3.70(3.64,3.77)	3.61(3.58,3.64)	42
Malaysia	72(61-83)	110	251(223-283)	96	6.80(6.70,6.90)	3.31(3.29,3.33)	3.04(2.99,3.10)	4.39(4.34,4.44)	15
Maldives	1(0-1)	190	3(2-3)	183	5.52(5.41,5.62)	4.70(4.61,4.79)	6.66(6.47,6.85)	5.63(5.55,5.71)	5
Myanmar	141(113-173)	86	306(253-378)	84	2.52(2.48,2.56)	2.69(2.65,2.72)	2.90(2.81,2.99)	2.70(2.67,2.73)	93
Philippines	242(197-292)	65	657(541-799)	56	4.36(4.26,4.47)	2.56(2.46,2.66)	3.69(3.37,4.01)	3.53(3.42,3.64)	49
Sri Lanka	107(88-131)	94	268(239-301)	90	5.83(5.59,6.07)	1.95(1.89,2.01)	2.31(2.24,2.37)	3.25(3.17,3.33)	74
Thailand	183(142-230)	80	373(309-455)	77	3.50(3.28,3.73)	1.97(1.89,2.04)	1.97(1.89,2.04)	2.49(2.41,2.58)	109
Timor-Leste	2(2-3)	178	6(5-7)	175	1.82(1.69,1.96)	4.27(4.22,4.31)	3.78(3.69,3.88)	3.26(3.20,3.32)	72
Viet Nam	402(316-499)	50	1,482(1,201-1,836)	33	8.84(8.38,9.30)	2.96(2.59,3.33)	2.37(2.31,2.43)	4.61(4.39,4.84)	10
Fiji	3(3-4)	170	6(5-8)	173	2.10(2.08,2.12)	2.59(2.54,2.63)	1.93(1.84,2.01)	2.23(2.20,2.27)	123
Kiribati	0(0-0)	195	1(0-1)	194	1.99(1.85,2.12)	2.98(2.73,3.23)	2.60(2.51,2.69)	2.52(2.42,2.61)	105
Marshall Islands	0(0-0)	198	0(0-0)	198	3.00(2.92,3.09)	2.80(2.71,2.88)	2.66(2.59,2.73)	2.83(2.78,2.89)	89
Micronesia (Federated States of)	0(0-0)	192	1(1-1)	193	1.85(1.74,1.97)	1.87(1.78,1.95)	1.65(1.57,1.73)	1.80(1.74,1.86)	145
Papua New Guinea	13(11-16)	152	43(36-53)	148	3.49(3.40,3.57)	4.43(4.35,4.51)	4.62(4.52,4.72)	4.20(4.15,4.26)	21
Samoa	1(0-1)	189	1(1-1)	191	0.59(0.41,0.76)	2.31(1.70,2.92)	3.46(3.07,3.85)	2.14(1.89,2.39)	129
Solomon Islands	1(1-1)	185	3(2-4)	182	3.98(3.93,4.02)	3.45(3.37,3.54)	3.39(3.35,3.43)	3.62(3.58,3.66)	41
Tonga	0(0-0)	193	1(0-1)	195	1.70(1.59,1.81)	2.21(2.07,2.36)	1.55(1.47,1.63)	1.89(1.81,1.96)	143
Vanuatu	1(0-1)	191	1(1-2)	189	3.30(3.19,3.41)	4.08(4.00,4.17)	3.55(3.47,3.62)	3.68(3.62,3.74)	38
Armenia	178(150-209)	81	237(200-283)	99	1.38(1.34,1.42)	1.14(1.07,1.21)	0.38(0.20,0.56)	0.98(0.92,1.05)	173
Azerbaijan	432(365-510)	49	805(677-965)	50	1.81(1.73,1.89)	2.72(2.66,2.77)	2.00(1.80,2.20)	2.18(2.11,2.25)	128
Georgia	381(325-449)	51	275(234-327)	89	-1.11(-1.45,-0.78)	-1.29(-1.35,-1.24)	-0.96(-1.29,-0.64)	-1.13(-1.28,-0.99)	202
Kazakhstan	922(782-1,089)	30	1,389(1,185-1,660)	35	0.50(0.39,0.61)	0.70(0.60,0.80)	3.10(2.83,3.37)	1.37(1.28,1.47)	162
Kyrgyzstan	222(189-260)	73	390(329-460)	74	2.10(1.89,2.31)	1.85(1.82,1.89)	1.85(1.82,1.89)	1.93(1.86,2.00)	141
Mongolia	98(82-116)	97	251(210-300)	97	2.93(2.85,3.01)	3.87(3.73,4.01)	2.99(2.86,3.12)	3.26(3.19,3.34)	73
Tajikistan	225(190-267)	72	518(439-612)	67	1.65(1.25,2.04)	3.68(3.20,4.16)	3.43(3.33,3.52)	2.87(2.67,3.08)	87
Turkmenistan	188(157-222)	79	383(324-451)	76	2.15(2.08,2.22)	3.15(2.83,3.47)	2.22(2.08,2.36)	2.47(2.36,2.59)	110
Uzbekistan	868(732-1,030)	33	2,213(1,878-2,642)	25	3.01(2.69,3.33)	3.63(3.47,3.78)	3.24(3.14,3.35)	3.33(3.18,3.48)	66
Albania	246(208-289)	64	291(249-341)	85	1.04(0.83,1.24)	1.02(0.80,1.23)	-0.02(-0.13,0.09)	0.56(0.40,0.72)	186
Bosnia and Herzegovina	345(301-395)	55	365(325-417)	78	0.26(-0.03,0.55)	0.88(0.73,1.02)	-0.84(-0.90,-0.79)	0.14(0.03,0.25)	193
Bulgaria	742(626-865)	38	851(729-996)	48	2.75(2.69,2.80)	-0.32(-0.48,-0.16)	-0.78(-0.82,-0.74)	0.48(0.41,0.55)	187
Croatia	527(493-562)	46	591(544-645)	64	0.03(-0.09,0.15)	0.50(0.46,0.54)	0.65(0.39,0.90)	0.40(0.31,0.49)	189
Czechia	543(463-656)	45	1,041(974-1,115)	44	2.67(2.41,2.94)	1.33(1.26,1.40)	2.80(2.24,3.37)	2.20(2.00,2.40)	126
Hungary	1,316(1,216-1,407)	28	2,268(2,160-2,384)	24	3.44(3.10,3.78)	2.00(1.81,2.19)	0.26(0.15,0.37)	1.93(1.79,2.07)	138
North Macedonia	194(165-227)	76	253(216-297)	95	1.32(1.20,1.45)	1.32(1.20,1.45)	0.26(-0.17,0.69)	0.99(0.83,1.15)	172
Montenegro	59(50-69)	118	67(57-79)	139	-0.13(-0.33,0.06)	1.09(0.97,1.22)	0.42(0.35,0.50)	0.45(0.37,0.53)	188

Poland	14,031(11,891-16,418)	5	5,805(5,141-6,573)	14	-5.05(-5.51,-4.60)	-2.46(-2.91,-2.01)	-0.50(-0.58,-0.41)	-3.03(-3.26,-2.80)	204
Romania	1,287(1,095-1,512)	29	1,187(1,085-1,305)	38	-1.92(-2.20,-1.65)	0.32(0.25,0.39)	0.63(0.35,0.91)	-0.28(-0.41,-0.15)	200
Serbia	909(776-1,060)	31	1,244(1,069-1,448)	37	3.37(2.96,3.79)	0.01(-0.09,0.11)	-0.21(-0.30,-0.12)	0.99(0.84,1.15)	171
Slovakia	566(501-637)	44	569(509-646)	65	0.77(0.54,0.99)	0.03(-0.08,0.14)	-0.62(-0.84,-0.39)	0.05(-0.07,0.17)	195
Slovenia	231(202-270)	71	277(245-316)	88	1.40(1.34,1.45)	0.71(0.68,0.74)	-0.26(-0.28,-0.23)	0.63(0.60,0.65)	185
Belarus	511(436-608)	48	649(552-774)	59	1.43(1.27,1.58)	0.19(0.07,0.30)	0.86(0.44,1.29)	0.78(0.63,0.93)	179
Estonia	62(55-70)	113	105(94-118)	126	2.54(2.19,2.88)	1.05(0.98,1.12)	2.04(1.35,2.73)	1.82(1.57,2.06)	144
Latvia	161(139-187)	83	154(135-179)	111	1.86(1.65,2.08)	-0.81(-0.91,-0.71)	-1.40(-1.56,-1.23)	-0.16(-0.26,-0.06)	199
Lithuania	233(204-267)	70	258(245-271)	94	1.19(1.09,1.29)	0.01(-0.07,0.08)	-0.18(-0.20,-0.17)	0.37(0.32,0.41)	190
Republic of Moldova	201(169-242)	74	194(179-210)	105	-1.81(-1.95,-1.66)	0.71(0.69,0.73)	0.58(0.52,0.63)	-0.12(-0.17,-0.07)	198
Russian Federation	13,522(11,622-15,867)	6	14,514(12,473-17,148)	7	1.08(0.84,1.33)	-0.07(-0.23,0.09)	-0.28(-0.32,-0.25)	0.23(0.13,0.33)	191
Ukraine	3,459(2,935-4,104)	14	3,626(3,093-4,258)	20	0.57(0.49,0.66)	0.03(-0.04,0.10)	-0.08(-0.27,0.11)	0.16(0.09,0.23)	192
Brunei Darussalam	13(10-15)	155	26(21-31)	155	3.03(2.97,3.09)	2.42(2.33,2.50)	1.92(1.86,1.98)	2.45(2.41,2.50)	111
Japan	16,383(13,833-19,567)	3	22,436(19,353-25,906)	4	3.72(2.95,4.49)	0.06(-0.03,0.15)	-0.32(-0.66,0.01)	1.06(0.80,1.32)	169
Republic of Korea	1,587(1,390-1,777)	22	4,407(4,220-4,589)	16	6.94(5.90,7.98)	2.03(1.99,2.07)	2.03(1.99,2.07)	3.53(3.22,3.85)	48
Singapore	123(101-147)	90	215(178-264)	101	2.12(1.68,2.56)	2.70(2.29,3.11)	0.93(0.57,1.29)	1.94(1.68,2.20)	137
Australia	2,213(1,884-2,621)	17	5,354(4,766-6,020)	15	5.51(5.12,5.90)	1.95(1.90,2.00)	2.09(2.02,2.15)	3.09(2.97,3.21)	78
New Zealand	766(663-876)	37	1,092(974-1,230)	43	1.50(1.40,1.60)	1.43(1.41,1.44)	0.78(0.69,0.86)	1.23(1.18,1.27)	164
Andorra	5(5-6)	166	9(8-11)	170	3.75(2.14,5.38)	2.68(2.38,2.97)	-0.10(-1.47,1.30)	1.90(1.16,2.65)	142
Austria	1,618(1,446-1,789)	21	1,940(1,749-2,182)	27	0.73(0.62,0.83)	1.38(1.11,1.64)	-0.25(-1.06,0.58)	0.66(0.39,0.94)	183
Belgium	829(787-875)	35	1,651(1,451-1,918)	30	1.19(0.95,1.42)	0.89(0.83,0.95)	5.58(4.78,6.38)	2.42(2.16,2.67)	114
Cyprus	32(28-38)	135	91(83-101)	132	5.56(5.40,5.72)	3.44(3.39,3.49)	1.94(1.84,2.04)	3.63(3.57,3.70)	40
Denmark	1,373(1,281-1,472)	24	1,334(1,247-1,419)	36	-1.33(-1.42,-1.25)	0.69(0.67,0.70)	0.34(0.23,0.44)	-0.08(-0.12,-0.04)	197
Finland	790(760-819)	36	1,500(1,345-1,652)	32	0.84(0.78,0.89)	1.70(1.60,1.81)	4.33(3.92,4.74)	2.22(2.09,2.35)	124
France	6,784(6,568-6,990)	12	8,431(7,970-8,812)	11	0.83(0.81,0.85)	0.83(0.81,0.85)	0.66(0.14,1.18)	0.78(0.61,0.94)	180
Germany	9,760(8,592-11,204)	10	16,746(14,805-18,956)	5	4.20(3.59,4.82)	0.39(0.24,0.54)	1.54(1.32,1.75)	1.94(1.73,2.16)	135
Greece	908(785-1,045)	32	911(849-975)	47	1.07(0.77,1.38)	-0.52(-0.87,-0.18)	-0.55(-0.63,-0.47)	0.01(-0.14,0.15)	196
Iceland	30(25-34)	139	65(56-73)	141	5.12(4.88,5.36)	2.11(2.04,2.19)	1.08(0.80,1.37)	2.65(2.51,2.78)	97
Ireland	351(298-411)	54	437(382-504)	71	0.90(0.70,1.11)	-0.08(-0.44,0.29)	1.42(1.36,1.47)	0.81(0.67,0.95)	178
Israel	298(258-342)	60	652(599-721)	58	4.59(4.40,4.79)	1.11(0.99,1.23)	2.31(2.25,2.37)	2.76(2.68,2.84)	92
Italy	12,109(10,641-13,753)	8	12,194(10,759-13,882)	10	-0.87(-1.10,-0.63)	1.83(1.56,2.09)	-0.90(-1.10,-0.70)	0.13(-0.02,0.27)	194
Luxembourg	84(73-97)	102	111(98-128)	125	-0.57(-0.68,-0.46)	1.13(0.88,1.38)	2.31(1.90,2.72)	0.96(0.80,1.12)	175
Malta	32(27-37)	137	44(38-51)	147	2.00(1.89,2.10)	1.01(0.96,1.06)	0.39(0.29,0.49)	1.13(1.08,1.19)	168
Netherlands	2,180(2,070-2,291)	18	1,460(1,269-1,692)	34	1.02(0.97,1.08)	-0.14(-0.29,0.01)	-5.30(-6.10,-4.50)	-1.43(-1.70,-1.16)	203
Norway	1,362(1,163-1,594)	25	2,113(1,834-2,432)	26	1.51(1.26,1.77)	1.97(1.77,2.18)	1.05(0.92,1.19)	1.56(1.44,1.68)	155
Portugal	607(533-697)	41	1,112(1,025-1,208)	41	5.04(4.70,5.38)	1.23(1.15,1.30)	0.14(-0.10,0.38)	2.10(1.96,2.24)	131
Spain	4,177(4,025-4,320)	13	6,798(6,558-7,062)	12	2.71(2.62,2.81)	2.06(2.04,2.09)	0.41(0.30,0.52)	1.66(1.62,1.71)	149
Sweden	2,296(2,012-2,617)	16	3,173(2,814-3,640)	22	0.78(0.58,0.97)	1.37(1.29,1.45)	1.23(1.02,1.44)	1.15(1.04,1.25)	167

Switzerland	853(755-975)	34	1,108(993-1,265)	42	1.45(1.38,1.51)	0.67(0.61,0.73)	0.68(0.65,0.72)	0.91(0.87,0.94)	176
United Kingdom	12,548(11,094-14,290)	7	16,688(14,854-19,052)	6	1.05(0.91,1.19)	1.23(1.20,1.26)	0.61(0.53,0.68)	0.98(0.93,1.03)	174
Argentina	524(437-621)	47	831(695-986)	49	1.56(1.51,1.60)	1.50(1.48,1.52)	1.72(1.66,1.77)	1.59(1.56,1.62)	152
Chile	314(267-361)	58	485(420-562)	68	3.47(3.17,3.76)	1.92(1.86,1.98)	-0.70(-1.03,-0.37)	1.53(1.39,1.68)	158
Uruguay	41(35-49)	128	66(55-78)	140	2.44(2.27,2.61)	1.14(1.09,1.18)	1.29(0.89,1.69)	1.59(1.45,1.72)	150
Canada	11,089(10,767-11,409)	9	14,206(13,697-14,688)	8	1.15(1.08,1.23)	1.85(1.83,1.88)	-0.57(-0.71,-0.42)	0.88(0.83,0.93)	177
United States of America	96,513(83,182-112,507)	1	85,388(77,956-94,653)	1	-4.46(-4.98,-3.95)	1.69(1.57,1.81)	1.13(1.04,1.21)	-0.45(-0.62,-0.27)	201
Antigua and Barbuda	1(1-1)	184	3(2-3)	184	4.49(4.35,4.63)	1.39(1.33,1.45)	2.77(2.70,2.84)	2.92(2.87,2.98)	83
Bahamas	5(4-6)	167	14(11-17)	166	4.17(4.06,4.28)	2.27(1.98,2.57)	3.89(3.75,4.02)	3.45(3.33,3.57)	55
Barbados	11(9-13)	159	18(15-22)	160	2.45(2.24,2.65)	0.07(-0.06,0.20)	2.44(2.34,2.53)	1.71(1.62,1.80)	148
Belize	3(2-3)	173	10(9-13)	169	5.01(4.76,5.25)	3.76(3.66,3.87)	5.00(4.91,5.09)	4.57(4.49,4.66)	11
Cuba	235(199-285)	69	359(302-430)	79	2.77(2.63,2.91)	-0.17(-0.35,0.01)	2.08(1.89,2.26)	1.52(1.42,1.62)	159
Dominica	1(1-2)	183	2(2-3)	186	3.51(3.40,3.61)	0.92(0.71,1.14)	1.55(1.44,1.65)	1.93(1.83,2.02)	139
Dominican Republic	117(96-139)	92	289(241-349)	86	4.09(3.98,4.21)	1.97(1.92,2.02)	3.42(3.35,3.48)	3.16(3.11,3.21)	77
Grenada	2(1-2)	181	3(3-4)	181	4.78(4.36,5.21)	1.45(1.25,1.65)	1.87(1.64,2.10)	2.70(2.53,2.88)	94
Guyana	14(12-17)	150	22(18-26)	158	2.14(1.89,2.39)	-0.31(-0.42,-0.20)	2.63(2.50,2.75)	1.45(1.35,1.55)	160
Haiti	96(80-116)	99	263(218-324)	93	4.20(4.05,4.36)	2.34(2.28,2.40)	4.16(4.08,4.24)	3.57(3.50,3.63)	45
Jamaica	38(32-46)	130	76(64-90)	136	3.12(2.69,3.55)	1.01(0.78,1.24)	3.31(3.04,3.57)	2.44(2.24,2.63)	112
Saint Lucia	2(2-3)	175	5(5-7)	176	3.77(3.59,3.94)	1.44(0.83,2.05)	3.37(3.13,3.61)	2.81(2.58,3.03)	90
Saint Vincent and the Grenadines	2(2-2)	180	4(3-4)	179	3.76(3.65,3.87)	0.49(0.44,0.55)	2.44(2.39,2.50)	2.19(2.15,2.24)	127
Suriname	8(6-9)	161	17(14-20)	161	3.38(3.14,3.62)	2.23(2.06,2.40)	2.89(2.74,3.04)	2.84(2.73,2.96)	88
Trinidad and Tobago	27(23-33)	141	52(44-64)	146	4.20(4.14,4.26)	0.58(0.47,0.70)	2.43(2.37,2.49)	2.27(2.22,2.32)	119
Bolivia (Plurinational State of)	79(67-93)	106	197(167-237)	103	4.37(4.25,4.49)	1.52(1.36,1.68)	4.06(3.61,4.50)	3.27(3.11,3.42)	71
Ecuador	120(105-136)	91	323(285-363)	82	6.24(6.13,6.36)	0.18(0.06,0.29)	4.07(3.74,4.40)	3.44(3.33,3.56)	56
Peru	274(228-325)	61	608(514-723)	62	3.56(3.13,3.99)	0.56(0.35,0.76)	4.53(4.31,4.76)	2.87(2.69,3.04)	86
Colombia	598(505-707)	42	1,129(960-1,327)	39	2.61(2.36,2.86)	0.93(0.79,1.06)	3.36(3.22,3.50)	2.26(2.16,2.37)	120
Costa Rica	63(53-74)	112	120(103-141)	124	3.23(3.02,3.43)	0.93(0.81,1.05)	2.46(2.37,2.54)	2.26(2.17,2.34)	121
El Salvador	101(85-120)	96	148(126-173)	112	1.87(1.68,2.05)	0.28(0.20,0.36)	1.82(1.73,1.92)	1.36(1.28,1.44)	163
Guatemala	130(110-154)	87	358(302-422)	80	3.59(3.40,3.79)	2.57(2.45,2.69)	4.38(4.29,4.47)	3.58(3.50,3.66)	44
Honduras	73(61-86)	109	182(153-215)	108	3.38(3.16,3.60)	2.04(1.90,2.17)	4.07(3.97,4.17)	3.24(3.14,3.33)	75
Mexico	2,540(2,184-2,963)	15	3,944(3,432-4,538)	17	1.08(0.72,1.43)	0.90(0.69,1.11)	2.68(2.54,2.83)	1.59(1.44,1.73)	153
Nicaragua	60(50-72)	116	124(106-147)	120	3.03(2.70,3.35)	2.05(1.68,2.41)	2.32(2.24,2.39)	2.51(2.35,2.67)	108
Panama	50(43-58)	124	92(79-108)	131	2.53(2.33,2.73)	0.73(0.06,1.40)	3.41(3.02,3.80)	2.22(1.95,2.49)	125
Venezuela (Bolivarian Republic of)	352(305-401)	53	655(555-767)	57	3.24(2.97,3.51)	0.67(0.04,1.29)	1.94(1.46,2.43)	2.01(1.73,2.29)	132
Brazil	7,717(6,763-8,849)	11	12,705(11,142-14,618)	9	1.94(1.80,2.08)	1.82(1.23,2.41)	1.42(1.27,1.58)	1.72(1.53,1.92)	147
Paraguay	109(95-126)	93	222(190-261)	100	5.77(5.59,5.96)	-0.53(-1.31,0.26)	2.21(2.01,2.41)	2.55(2.29,2.82)	103
Algeria	577(508-648)	43	1,657(1,373-2,043)	29	5.12(5.07,5.17)	2.95(2.75,3.15)	2.84(2.75,2.94)	3.68(3.61,3.76)	37
Bahrain	13(11-15)	151	58(48-72)	144	3.95(3.58,4.32)	7.99(7.72,8.25)	3.35(3.06,3.64)	5.19(5.00,5.38)	6

Egypt	1,624(1,415-1,814)	20	3,833(3,168-4,689)	19	4.23(4.02,4.43)	1.26(1.03,1.49)	3.25(3.17,3.34)	2.95(2.85,3.06)	81
Iran (Islamic Republic of)	1,347(1,111-1,654)	27	3,167(2,599-3,894)	23	4.02(3.76,4.29)	2.84(2.59,3.10)	2.04(1.97,2.11)	3.05(2.92,3.18)	79
Iraq	240(202-282)	68	984(810-1,191)	45	5.09(4.94,5.23)	4.71(4.17,5.24)	4.62(4.31,4.93)	4.88(4.66,5.10)	8
Jordan	130(114-147)	88	779(649-917)	51	6.29(6.03,6.55)	6.14(5.65,6.63)	6.81(6.30,7.32)	6.36(6.08,6.63)	3
Kuwait	61(57-66)	114	195(161-239)	104	1.46(0.90,2.02)	5.69(5.06,6.32)	4.79(4.01,5.58)	4.10(3.72,4.48)	26
Lebanon	65(54-80)	111	194(161-238)	106	4.24(3.77,4.72)	3.66(3.55,3.77)	3.57(2.61,4.53)	3.81(3.48,4.15)	31
Libya	51(42-62)	122	178(146-220)	109	6.09(5.91,6.27)	2.84(2.38,3.30)	4.73(3.86,5.59)	4.45(4.12,4.78)	13
Morocco	300(252-354)	59	599(508-712)	63	2.76(2.64,2.87)	2.01(1.90,2.12)	2.45(2.42,2.48)	2.41(2.36,2.46)	115
Palestine	29(24-35)	140	122(101-145)	122	7.26(7.11,7.40)	3.97(3.66,4.29)	4.00(3.82,4.19)	5.14(5.01,5.27)	7
Oman	38(31-46)	129	143(115-173)	116	3.96(3.07,4.86)	2.60(1.98,3.22)	6.83(5.52,8.16)	4.64(3.99,5.28)	9
Qatar	13(10-16)	153	125(102-152)	119	4.11(3.89,4.33)	13.79(13.07,14.50)	6.51(6.23,6.78)	8.24(7.99,8.49)	1
Saudi Arabia	341(287-404)	56	1,122(929-1,353)	40	4.84(4.64,5.03)	3.38(3.29,3.47)	4.24(3.83,4.66)	4.17(4.02,4.33)	24
Syrian Arab Republic	241(200-289)	67	449(376-544)	70	4.08(3.72,4.44)	2.93(2.80,3.06)	-0.24(-0.68,0.19)	2.14(1.94,2.35)	130
Tunisia	168(139-202)	82	349(288-426)	81	3.39(3.32,3.45)	2.61(2.43,2.79)	1.55(1.48,1.63)	2.56(2.49,2.63)	102
Turkey	2,084(1,859-2,316)	19	5,961(5,119-6,893)	13	5.14(4.83,5.44)	1.75(1.42,2.09)	4.44(4.05,4.82)	3.65(3.43,3.88)	39
United Arab Emirates	58(47-71)	119	520(410-651)	66	8.72(8.48,8.97)	13.25(12.57,13.93)	2.55(2.39,2.71)	7.89(7.66,8.11)	2
Yemen	192(157-230)	77	647(527-793)	60	4.27(4.16,4.38)	4.68(4.60,4.76)	3.88(3.78,3.97)	4.30(4.24,4.36)	18
Afghanistan	192(159-228)	78	684(557-828)	55	4.92(3.91,5.94)	4.59(4.49,4.68)	4.59(4.49,4.68)	4.53(4.14,4.92)	12
Bangladesh	1,355(1,105-1,656)	26	3,404(2,788-4,136)	21	3.94(3.86,4.03)	2.89(2.69,3.08)	2.70(2.57,2.83)	3.22(3.13,3.30)	76
Bhutan	7(6-9)	162	14(12-17)	165	2.57(2.10,3.05)	1.39(1.15,1.62)	2.88(2.65,3.12)	2.30(2.10,2.49)	118
India	15,360(12,631-18,815)	4	31,775(26,433-38,972)	3	3.02(2.84,3.19)	3.35(3.31,3.39)	1.03(0.56,1.50)	2.52(2.36,2.68)	106
Nepal	242(198-301)	66	624(515-764)	61	3.68(3.55,3.82)	3.20(3.16,3.25)	3.08(2.96,3.19)	3.34(3.28,3.40)	65
Pakistan	1,474(1,218-1,792)	23	3,888(3,193-4,838)	18	3.15(3.05,3.24)	3.24(3.16,3.32)	3.75(3.72,3.78)	3.42(3.38,3.46)	60
Angola	75(61-92)	108	263(219-315)	92	4.11(4.06,4.17)	4.52(4.50,4.54)	4.66(4.61,4.71)	4.45(4.42,4.47)	14
Central African Republic	21(17-25)	144	42(35-51)	150	2.70(2.67,2.73)	2.70(2.67,2.73)	2.11(1.82,2.40)	2.52(2.42,2.61)	107
Congo	18(15-22)	145	60(50-72)	143	4.99(4.82,5.16)	3.56(3.18,3.94)	4.10(3.82,4.38)	4.26(4.09,4.43)	19
Democratic Republic of the Congo	256(214-314)	63	745(616-916)	53	3.39(3.27,3.52)	3.20(3.04,3.36)	4.91(4.81,5.01)	3.78(3.70,3.87)	32
Equatorial Guinea	3(2-3)	174	15(13-19)	162	4.45(4.38,4.52)	7.70(7.57,7.82)	5.98(5.85,6.11)	6.01(5.94,6.07)	4
Gabon	9(7-11)	160	23(19-28)	157	3.60(3.56,3.65)	3.10(3.06,3.14)	3.56(3.47,3.65)	3.43(3.39,3.46)	58
Burundi	34(28-41)	134	79(64-95)	135	1.35(1.16,1.55)	4.22(3.94,4.50)	3.33(3.05,3.60)	2.96(2.82,3.10)	80
Comoros	3(3-4)	171	7(6-9)	172	3.32(3.23,3.41)	2.46(2.38,2.55)	3.16(3.09,3.23)	2.94(2.89,2.99)	82
Djibouti	3(2-4)	172	10(9-13)	168	4.85(4.29,5.41)	4.46(4.37,4.55)	3.71(3.60,3.82)	4.35(4.16,4.54)	16
Eritrea	18(15-21)	146	54(44-65)	145	4.64(4.50,4.79)	4.05(3.95,4.15)	3.31(3.15,3.46)	4.00(3.90,4.10)	27
Ethiopia	270(224-325)	62	710(590-855)	54	2.86(2.75,2.98)	3.20(2.92,3.49)	4.14(4.10,4.18)	3.39(3.29,3.49)	62
Kenya	124(103-149)	89	427(357-512)	72	3.75(3.59,3.91)	3.86(3.79,3.93)	5.45(5.17,5.72)	4.32(4.21,4.43)	17
Madagascar	79(66-96)	105	207(172-252)	102	3.02(2.96,3.08)	3.73(3.62,3.85)	3.40(3.35,3.44)	3.38(3.34,3.43)	64
Malawi	60(50-74)	115	139(114-170)	117	1.49(1.00,1.98)	2.99(2.92,3.06)	4.14(4.07,4.21)	2.89(2.73,3.05)	84
Mauritius	5(4-6)	168	9(7-10)	171	2.50(2.40,2.60)	1.79(1.76,1.82)	1.71(1.69,1.73)	1.99(1.95,2.04)	133

Mozambique	76(63-93)	107	192(158-230)	107	3.25(2.87,3.63)	3.26(3.21,3.31)	3.36(3.32,3.40)	3.29(3.16,3.41)	69
Rwanda	45(37-55)	125	102(84-124)	127	0.52(-2.17,3.29)	4.08(3.50,4.65)	3.07(2.41,3.73)	2.61(1.70,3.53)	100
Seychelles	0(0-0)	194	1(1-1)	192	3.07(3.02,3.12)	2.36(2.30,2.43)	2.42(2.14,2.70)	2.62(2.52,2.71)	99
Somalia	37(30-44)	132	100(81-124)	128	3.49(3.28,3.69)	3.72(3.68,3.76)	3.54(3.38,3.69)	3.57(3.48,3.66)	46
United Republic of Tanzania	156(128-188)	84	421(348-511)	73	2.96(2.92,2.99)	3.40(3.38,3.42)	4.06(4.02,4.10)	3.49(3.47,3.51)	50
Uganda	97(79-119)	98	281(232-342)	87	3.17(3.07,3.27)	3.91(3.67,4.14)	4.13(4.08,4.19)	3.74(3.66,3.83)	34
Zambia	53(44-64)	121	143(117-174)	115	2.27(2.17,2.37)	4.28(4.13,4.42)	3.64(3.54,3.73)	3.44(3.37,3.51)	57
Botswana	12(10-14)	157	31(26-39)	153	3.56(3.42,3.71)	3.38(3.06,3.71)	3.64(3.42,3.86)	3.56(3.42,3.70)	47
Lesotho	15(13-19)	148	25(20-30)	156	1.85(1.70,2.00)	0.84(0.72,0.95)	2.05(2.01,2.09)	1.59(1.52,1.66)	151
Namibia	13(11-15)	154	27(23-33)	154	3.76(3.73,3.79)	2.21(2.16,2.26)	2.04(1.98,2.10)	2.69(2.66,2.72)	95
South Africa	376(317-452)	52	746(636-891)	52	3.13(3.01,3.26)	1.61(1.39,1.84)	2.49(2.41,2.57)	2.44(2.35,2.53)	113
Eswatini	6(5-8)	164	13(11-16)	167	3.74(3.52,3.96)	1.48(1.36,1.61)	2.42(2.34,2.50)	2.56(2.46,2.65)	101
Zimbabwe	81(67-99)	103	146(120-178)	113	2.93(2.59,3.28)	0.08(-0.06,0.23)	3.09(2.96,3.21)	1.99(1.87,2.12)	134
Benin	32(26-39)	136	97(80-116)	129	3.29(3.22,3.35)	3.63(3.58,3.67)	4.97(4.73,5.21)	3.93(3.86,4.01)	28
Burkina Faso	60(50-72)	117	159(132-194)	110	2.28(2.19,2.36)	3.43(3.10,3.76)	4.49(4.25,4.73)	3.41(3.27,3.54)	61
Cameroon	80(66-97)	104	265(219-322)	91	4.32(4.13,4.50)	3.65(3.46,3.84)	4.51(4.21,4.81)	4.15(4.01,4.29)	25
Cabo Verde	2(2-3)	176	6(5-8)	174	3.49(3.46,3.53)	3.04(2.99,3.09)	3.98(3.76,4.20)	3.48(3.41,3.56)	52
Chad	35(29-43)	133	97(80-116)	130	2.66(2.57,2.74)	2.89(2.67,3.10)	5.05(4.66,5.44)	3.48(3.33,3.63)	53
C??te d'Ivoire	85(70-103)	101	242(198-298)	98	4.91(4.56,5.27)	2.35(2.18,2.52)	4.06(3.87,4.25)	3.73(3.58,3.87)	35
Gambia	6(5-7)	165	19(16-24)	159	4.08(4.04,4.12)	4.08(4.04,4.12)	4.44(4.09,4.79)	4.19(4.08,4.31)	23
Ghana	104(86-124)	95	307(255-370)	83	3.88(3.65,4.11)	3.22(3.06,3.38)	4.17(3.92,4.41)	3.75(3.61,3.89)	33
Guinea	42(35-52)	127	91(75-109)	133	2.80(2.68,2.91)	1.57(1.50,1.63)	3.79(3.54,4.03)	2.63(2.55,2.72)	98
Guinea-Bissau	7(5-8)	163	15(12-18)	164	2.37(2.35,2.39)	2.30(2.24,2.35)	4.15(4.07,4.24)	2.89(2.86,2.93)	85
Liberia	15(12-18)	149	43(36-52)	149	2.93(1.90,3.96)	3.22(1.41,5.07)	5.17(4.94,5.40)	3.84(3.16,4.52)	29
Mali	53(45-66)	120	143(120-172)	114	2.98(2.88,3.08)	2.67(2.56,2.78)	4.98(4.73,5.23)	3.49(3.40,3.58)	51
Mauritania	16(13-19)	147	34(28-41)	152	1.16(0.68,1.64)	3.19(3.12,3.27)	3.57(3.26,3.87)	2.67(2.49,2.86)	96
Niger	42(35-51)	126	122(101-148)	121	3.74(3.69,3.80)	2.73(2.66,2.81)	4.75(4.57,4.93)	3.70(3.63,3.76)	36
Nigeria	634(536-771)	40	1,713(1,440-2,066)	28	2.97(2.90,3.04)	2.67(2.25,3.10)	5.03(4.59,5.47)	3.47(3.22,3.71)	54
Sao Tome and Principe	1(1-1)	187	2(2-2)	188	2.15(2.10,2.19)	2.47(2.34,2.60)	5.43(4.99,5.87)	3.31(3.15,3.46)	67
Senegal	51(42-62)	123	132(109-161)	118	3.21(3.17,3.26)	2.69(2.60,2.77)	4.14(3.81,4.48)	3.30(3.19,3.41)	68
Sierra Leone	27(23-33)	142	67(55-81)	138	1.33(1.02,1.63)	3.08(2.45,3.71)	5.16(4.90,5.42)	3.28(3.03,3.54)	70
Togo	22(18-27)	143	74(62-90)	137	5.20(4.98,5.43)	3.06(2.93,3.19)	4.63(4.10,5.17)	4.21(4.02,4.40)	20
American Samoa	0(0-0)	197	0(0-0)	197	2.97(2.81,3.12)	1.48(1.34,1.63)	1.37(1.15,1.59)	1.93(1.82,2.05)	140
Bermuda	2(1-2)	182	2(2-3)	185	2.78(2.45,3.10)	0.02(-0.18,0.21)	1.74(1.60,1.88)	1.58(1.44,1.71)	154
Cook Islands	0(0-0)	199	0(0-0)	200	1.49(1.46,1.51)	2.13(2.03,2.22)	1.82(1.68,1.95)	1.79(1.74,1.85)	146
Greenland	11(9-14)	158	15(13-18)	163	1.86(1.48,2.24)	1.16(0.97,1.35)	0.16(0.03,0.29)	1.06(0.91,1.21)	170
Guam	1(1-1)	188	1(1-2)	190	2.39(2.31,2.47)	2.26(2.09,2.44)	1.96(1.87,2.06)	2.24(2.16,2.31)	122
Monaco	3(3-4)	169	4(4-5)	177	1.08(1.05,1.10)	0.55(0.49,0.61)	0.29(0.24,0.34)	0.65(0.62,0.67)	184

Nauru	0(0-0)	202	0(0-0)	202	2.46(1.69,3.23)	0.90(0.81,0.98)	0.90(0.81,0.98)	1.38(1.13,1.62)	161
Niue	0(0-0)	203	0(0-0)	203	0.09(0.01,0.17)	0.24(0.17,0.32)	2.02(1.94,2.10)	0.76(0.71,0.81)	181
Northern Mariana Islands	0(0-0)	196	0(0-0)	196	4.94(3.82,6.06)	-1.52(-2.76,-0.26)	-0.50(-0.73,-0.26)	1.20(0.63,1.76)	165
Palau	0(0-0)	200	0(0-0)	199	4.75(4.66,4.83)	1.14(0.93,1.34)	1.96(1.87,2.05)	2.54(2.44,2.65)	104
Puerto Rico	87(79-94)	100	122(108-137)	123	2.80(2.64,2.96)	-0.51(-0.70,-0.32)	1.32(0.81,1.83)	1.19(1.01,1.37)	166
Saint Kitts and Nevis	1(1-1)	186	2(2-2)	187	4.62(4.36,4.88)	1.77(1.63,1.91)	3.86(3.71,4.00)	3.42(3.31,3.53)	59
San Marino	2(2-3)	179	3(3-4)	180	2.41(2.32,2.51)	1.16(1.14,1.19)	1.02(0.94,1.10)	1.56(1.51,1.60)	156
Tokelau	0(0-0)	204	0(0-0)	204	-0.01(-0.16,0.14)	-1.99(-2.75,-1.22)	4.00(3.82,4.17)	0.73(0.47,0.98)	182
Tuvalu	0(0-0)	201	0(0-0)	201	1.64(1.63,1.66)	2.58(2.53,2.63)	2.66(2.57,2.75)	2.32(2.29,2.36)	117
United States Virgin Islands	2(2-3)	177	4(3-4)	178	3.63(3.50,3.77)	0.12(-0.02,0.26)	0.83(0.23,1.44)	1.54(1.34,1.74)	157
South Sudan	37(31-45)	131	63(53-76)	142	2.16(2.05,2.28)	2.81(2.66,2.97)	0.70(0.15,1.24)	1.94(1.75,2.13)	136
Sudan	334(275-400)	57	934(775-1,114)	46	3.67(3.53,3.81)	3.12(2.99,3.24)	4.03(3.88,4.19)	3.58(3.48,3.67)	43
Multiple sclerosis									
China	2,005(1,619-2,432)	5	2,967(2,423-3,547)	3	1.43(1.40,1.46)	1.11(1.07,1.15)	1.50(1.42,1.59)	1.34(1.31,1.37)	128
Democratic People's Republic of Korea	61(49-74)	64	85(69-102)	75	1.17(1.09,1.25)	1.22(1.14,1.30)	1.18(1.11,1.25)	1.19(1.14,1.24)	136
Taiwan (Province of China)	34(30-38)	88	67(58-77)	92	1.18(1.07,1.28)	4.38(4.14,4.61)	1.79(1.46,2.12)	2.30(2.17,2.43)	88
Cambodia	14(11-17)	123	28(23-35)	122	2.57(2.53,2.62)	2.55(2.53,2.57)	2.16(2.14,2.18)	2.44(2.42,2.45)	81
Indonesia	255(198-313)	28	427(343-517)	32	2.14(2.10,2.17)	1.70(1.67,1.72)	1.57(1.56,1.59)	1.80(1.79,1.81)	112
Lao People's Democratic Republic	7(6-9)	142	14(12-18)	140	2.56(2.52,2.59)	2.44(2.27,2.60)	2.39(2.35,2.43)	2.46(2.41,2.52)	80
Malaysia	19(15-23)	113	40(31-48)	111	3.75(3.71,3.79)	2.44(2.36,2.53)	1.64(1.58,1.70)	2.62(2.58,2.66)	72
Maldives	0(0-0)	190	1(0-1)	180	3.43(3.30,3.56)	3.74(3.71,3.77)	4.41(4.32,4.49)	3.87(3.82,3.93)	19
Myanmar	91(73-110)	60	139(112-167)	60	1.81(1.79,1.84)	1.34(1.32,1.35)	1.26(1.15,1.37)	1.47(1.44,1.51)	123
Philippines	97(76-119)	59	201(161-245)	49	2.73(2.70,2.75)	2.51(2.44,2.57)	2.47(2.43,2.50)	2.58(2.55,2.60)	73
Sri Lanka	25(20-31)	100	37(31-44)	115	1.98(1.91,2.05)	1.23(1.12,1.35)	0.73(0.69,0.77)	1.32(1.27,1.36)	129
Thailand	100(79-123)	57	151(123-182)	53	1.73(1.57,1.89)	1.64(1.25,2.03)	0.99(0.92,1.06)	1.43(1.29,1.57)	125
Timor-Leste	1(1-1)	171	2(1-2)	170	1.55(1.49,1.61)	2.18(2.09,2.26)	2.91(2.89,2.92)	2.21(2.18,2.24)	95
Viet Nam	113(90-138)	53	206(166-248)	47	2.53(2.48,2.59)	2.20(2.16,2.24)	1.55(1.52,1.58)	2.10(2.07,2.12)	103
Fiji	1(1-1)	167	2(1-2)	171	1.11(1.08,1.14)	1.29(1.28,1.30)	0.82(0.77,0.86)	1.07(1.06,1.09)	142
Kiribati	0(0-0)	195	0(0-0)	194	2.22(2.07,2.37)	2.34(2.22,2.46)	0.64(0.50,0.78)	1.76(1.67,1.85)	117
Marshall Islands	0(0-0)	198	0(0-0)	198	2.75(2.70,2.79)	1.36(1.28,1.44)	0.82(0.77,0.86)	1.62(1.58,1.66)	120
Micronesia (Federated States of)	0(0-0)	194	0(0-0)	195	1.43(1.30,1.55)	0.33(0.26,0.40)	0.06(0.03,0.08)	0.58(0.54,0.63)	160
Papua New Guinea	4(3-5)	150	11(8-13)	147	2.97(2.92,3.02)	3.54(3.49,3.59)	3.30(3.26,3.34)	3.29(3.26,3.31)	37
Samoa	0(0-0)	187	0(0-0)	189	1.11(1.07,1.16)	0.92(0.90,0.94)	1.52(1.49,1.55)	1.18(1.16,1.20)	137
Solomon Islands	0(0-0)	179	1(1-1)	175	3.52(3.44,3.60)	2.52(2.43,2.61)	1.88(1.79,1.97)	2.63(2.57,2.68)	71
Tonga	0(0-0)	192	0(0-0)	193	0.62(0.59,0.66)	0.76(0.74,0.78)	0.24(0.22,0.26)	0.54(0.53,0.56)	161
Vanuatu	0(0-0)	188	0(0-1)	184	2.58(2.55,2.62)	3.03(3.02,3.05)	2.49(2.47,2.50)	2.70(2.69,2.72)	68
Armenia	37(31-42)	83	46(40-53)	104	1.71(1.54,1.89)	0.87(0.69,1.05)	-0.15(-0.20,-0.09)	0.83(0.74,0.91)	152
Azerbaijan	56(46-66)	70	105(87-124)	70	1.48(1.23,1.72)	2.93(2.54,3.32)	2.33(2.18,2.48)	2.25(2.10,2.41)	91

Georgia	50(42-59)	74	43(36-50)	109	-0.57(-1.16,0.03)	-0.68(-0.76,-0.60)	-0.20(-0.39,0.00)	-0.50(-0.69,-0.30)	189
Kazakhstan	416(372-460)	18	551(491-617)	25	0.59(0.44,0.75)	2.09(1.96,2.23)	0.01(-0.15,0.17)	0.96(0.86,1.06)	150
Kyrgyzstan	33(27-39)	90	57(46-67)	97	1.08(0.84,1.33)	2.15(2.03,2.28)	2.60(2.51,2.69)	1.96(1.85,2.07)	106
Mongolia	18(14-21)	119	38(31-45)	113	1.34(1.05,1.63)	3.12(2.96,3.27)	3.46(2.94,3.98)	2.66(2.45,2.87)	70
Tajikistan	32(26-39)	91	76(62-91)	85	1.52(1.22,1.82)	3.59(3.45,3.73)	4.04(3.95,4.13)	3.06(2.93,3.20)	48
Turkmenistan	43(37-48)	81	84(75-94)	78	2.91(2.81,3.00)	2.89(2.82,2.95)	1.25(1.19,1.31)	2.38(2.33,2.42)	85
Uzbekistan	203(172-236)	34	352(293-414)	33	2.23(2.04,2.43)	2.58(2.26,2.90)	0.82(0.69,0.95)	1.88(1.75,2.00)	111
Albania	115(105-125)	52	86(80-93)	74	1.11(0.99,1.24)	-0.46(-0.59,-0.34)	-3.61(-4.60,-2.60)	-0.98(-1.31,-0.65)	196
Bosnia and Herzegovina	56(48-64)	71	38(33-42)	114	-1.31(-1.66,-0.95)	-0.89(-1.05,-0.73)	-2.22(-2.28,-2.15)	-1.44(-1.57,-1.31)	201
Bulgaria	197(181-216)	36	141(127-156)	58	-0.05(-0.12,0.01)	-0.73(-0.81,-0.66)	-2.89(-3.56,-2.22)	-1.20(-1.42,-0.99)	198
Croatia	50(44-56)	75	49(43-55)	101	0.39(0.37,0.42)	-0.09(-0.16,-0.01)	-0.60(-0.62,-0.58)	-0.10(-0.13,-0.07)	185
Czechia	167(150-184)	40	140(123-157)	59	0.10(0.05,0.16)	-0.61(-0.68,-0.54)	-1.33(-1.42,-1.25)	-0.62(-0.66,-0.58)	193
Hungary	203(187-219)	33	130(114-146)	62	0.00(-0.06,0.07)	-0.50(-0.60,-0.40)	-4.22(-4.69,-3.76)	-1.51(-1.67,-1.35)	203
North Macedonia	28(24-31)	96	37(32-41)	117	1.52(1.35,1.69)	1.37(1.22,1.52)	0.14(-0.22,0.50)	1.00(0.83,1.16)	146
Montenegro	13(11-14)	124	13(12-14)	143	0.93(0.88,0.98)	0.23(-0.02,0.47)	-1.01(-1.11,-0.91)	0.05(-0.03,0.14)	181
Poland	976(846-1,103)	13	817(725-908)	16	-0.24(-0.46,-0.03)	-0.38(-0.46,-0.30)	-1.18(-1.22,-1.14)	-0.60(-0.67,-0.52)	191
Romania	203(175-232)	35	144(123-169)	56	-0.35(-0.36,-0.33)	-1.31(-1.33,-1.29)	-1.90(-1.95,-1.85)	-1.16(-1.18,-1.14)	197
Serbia	149(134-163)	42	150(136-163)	54	1.03(0.51,1.55)	-0.42(-0.57,-0.27)	-0.64(-0.69,-0.59)	0.01(-0.20,0.22)	182
Slovakia	57(48-65)	69	63(55-72)	93	0.85(0.77,0.93)	0.32(0.17,0.46)	0.01(-0.07,0.08)	0.39(0.32,0.46)	170
Slovenia	34(31-38)	87	34(30-37)	119	0.95(0.92,0.98)	0.00(-0.07,0.06)	-1.12(-1.17,-1.08)	-0.05(-0.08,-0.02)	184
Belarus	99(83-115)	58	84(71-97)	77	-0.13(-0.16,-0.10)	-0.38(-0.47,-0.28)	-1.12(-1.29,-0.95)	-0.53(-0.60,-0.47)	190
Estonia	20(17-22)	112	13(12-15)	141	-1.89(-2.04,-1.75)	-0.67(-0.81,-0.54)	-1.70(-1.82,-1.58)	-1.37(-1.45,-1.28)	200
Latvia	36(32-40)	84	22(19-25)	129	-1.45(-1.49,-1.41)	-1.04(-1.07,-1.00)	-2.48(-2.56,-2.40)	-1.67(-1.71,-1.64)	204
Lithuania	48(42-54)	77	31(27-35)	121	-0.68(-0.78,-0.58)	-1.58(-1.65,-1.50)	-2.25(-2.31,-2.19)	-1.51(-1.56,-1.46)	202
Republic of Moldova	30(24-36)	93	26(22-30)	124	-0.35(-0.69,-0.01)	-0.42(-0.51,-0.32)	-0.68(-0.79,-0.56)	-0.48(-0.59,-0.36)	188
Russian Federation	1,764(1,486-2,026)	6	1,339(1,130-1,547)	12	-0.68(-0.74,-0.61)	-0.35(-0.38,-0.33)	-1.92(-2.05,-1.78)	-0.94(-0.99,-0.89)	195
Ukraine	925(800-1,047)	14	636(548-725)	19	-0.84(-1.01,-0.67)	-0.40(-0.50,-0.31)	-2.91(-3.46,-2.35)	-1.32(-1.51,-1.14)	199
Brunei Darussalam	0(0-0)	180	1(1-1)	176	4.11(3.99,4.24)	2.32(2.16,2.48)	0.83(0.67,0.99)	2.40(2.31,2.49)	83
Japan	467(380-563)	17	496(408-590)	29	0.76(0.66,0.87)	-0.26(-0.29,-0.23)	0.13(0.05,0.21)	0.23(0.18,0.28)	176
Republic of Korea	184(144-225)	37	222(181-270)	44	0.57(0.53,0.61)	0.62(0.61,0.63)	0.77(0.71,0.82)	0.65(0.63,0.68)	157
Singapore	4(3-5)	149	8(7-10)	151	4.17(3.95,4.40)	2.16(1.56,2.76)	0.01(-0.26,0.28)	2.20(1.97,2.43)	98
Australia	282(252-313)	27	532(466-600)	26	4.54(3.87,5.21)	1.51(1.39,1.63)	0.78(0.35,1.21)	2.21(1.96,2.46)	92
New Zealand	55(46-62)	73	60(52-69)	96	0.74(0.59,0.90)	0.64(0.56,0.73)	-0.35(-0.45,-0.26)	0.35(0.27,0.42)	172
Andorra	1(1-1)	166	2(2-2)	169	2.36(0.65,4.10)	2.37(0.80,3.97)	-1.44(-1.94,-0.93)	0.95(0.18,1.74)	151
Austria	179(154-204)	39	241(211-272)	40	1.50(1.36,1.63)	0.59(0.44,0.75)	1.21(1.07,1.36)	1.06(0.97,1.16)	143
Belgium	254(227-280)	29	285(249-323)	37	0.33(0.28,0.38)	0.57(0.55,0.59)	0.27(0.22,0.31)	0.39(0.37,0.41)	171
Cyprus	10(9-11)	132	22(19-26)	128	2.34(2.25,2.43)	4.14(3.93,4.36)	2.08(1.94,2.22)	2.90(2.81,3.00)	58
Denmark	215(199-232)	32	222(200-247)	43	0.34(0.29,0.40)	-0.21(-0.25,-0.16)	0.21(0.13,0.28)	0.09(0.06,0.12)	179

Finland	133(119-145)	46	144(132-156)	57	0.36(0.18,0.54)	-0.40(-0.50,-0.30)	0.90(0.36,1.44)	0.24(0.06,0.42)	174
France	1,373(1,192-1,587)	7	1,629(1,457-1,803)	8	1.33(1.16,1.49)	0.51(0.48,0.54)	-0.01(-0.05,0.03)	0.60(0.55,0.66)	158
Germany	2,305(2,031-2,607)	3	2,425(2,125-2,755)	5	0.70(0.62,0.77)	-0.36(-0.40,-0.31)	0.28(0.23,0.34)	0.18(0.14,0.21)	177
Greece	108(94-121)	55	123(106-140)	63	1.94(1.88,2.00)	0.28(0.18,0.38)	-0.79(-0.85,-0.73)	0.46(0.42,0.51)	165
Iceland	10(10-11)	131	13(11-15)	144	0.77(0.66,0.89)	1.65(1.36,1.95)	0.01(-0.16,0.18)	0.79(0.67,0.90)	155
Ireland	145(128-161)	43	197(168-225)	50	2.09(1.90,2.28)	2.83(2.73,2.93)	-1.58(-1.96,-1.20)	1.12(0.98,1.27)	140
Israel	55(45-65)	72	110(92-128)	68	2.67(2.64,2.70)	2.47(2.45,2.50)	2.06(1.98,2.14)	2.41(2.38,2.43)	82
Italy	1,292(1,109-1,487)	8	1,424(1,204-1,658)	11	-0.15(-0.21,-0.08)	1.09(0.99,1.20)	-0.09(-0.44,0.25)	0.32(0.20,0.44)	173
Luxembourg	12(11-14)	127	20(17-23)	132	1.61(1.54,1.68)	1.30(1.22,1.37)	2.20(2.14,2.25)	1.70(1.66,1.74)	118
Malta	3(3-3)	156	4(4-5)	158	1.00(0.99,1.01)	0.69(0.65,0.73)	1.73(1.65,1.80)	1.11(1.08,1.14)	141
Netherlands	504(451-569)	16	503(441-569)	28	0.39(0.26,0.53)	-0.08(-0.11,-0.05)	-0.34(-0.38,-0.31)	-0.01(-0.06,0.04)	183
Norway	153(134-175)	41	264(225-303)	39	2.05(1.88,2.22)	1.41(1.20,1.63)	2.44(2.35,2.54)	1.94(1.83,2.04)	107
Portugal	123(103-142)	50	118(107-131)	65	0.72(0.68,0.76)	-0.15(-0.23,-0.08)	-0.98(-1.02,-0.94)	-0.12(-0.15,-0.09)	186
Spain	739(663-822)	15	1,073(973-1,172)	13	2.86(2.18,3.55)	1.98(1.58,2.38)	-0.49(-0.69,-0.29)	1.34(1.07,1.61)	127
Sweden	382(333-435)	20	508(445-574)	27	0.89(0.78,0.99)	1.00(0.90,1.10)	1.08(1.01,1.15)	1.00(0.94,1.05)	147
Switzerland	242(219-270)	30	287(253-322)	36	0.53(0.49,0.57)	0.61(0.59,0.62)	0.58(0.54,0.62)	0.58(0.56,0.60)	159
United Kingdom	2,006(1,777-2,233)	4	2,873(2,535-3,227)	4	1.64(1.43,1.85)	1.01(0.83,1.20)	1.21(1.15,1.26)	1.29(1.20,1.38)	131
Argentina	309(259-356)	23	466(392-538)	31	1.62(1.61,1.63)	1.57(1.56,1.58)	1.08(1.00,1.17)	1.43(1.40,1.46)	124
Chile	108(87-131)	54	158(129-188)	51	1.04(1.01,1.06)	1.21(1.18,1.24)	1.67(1.44,1.90)	1.30(1.22,1.37)	130
Uruguay	28(24-33)	95	33(28-38)	120	0.90(0.84,0.96)	0.50(0.47,0.53)	0.04(-0.03,0.10)	0.48(0.45,0.51)	164
Canada	1,262(1,231-1,297)	9	1,591(1,548-1,638)	9	0.62(0.54,0.70)	0.28(0.25,0.31)	1.56(1.48,1.64)	0.79(0.75,0.83)	154
United States of America	9,344(8,082-10,656)	1	10,465(9,519-11,369)	1	-0.30(-0.40,-0.20)	0.75(0.72,0.78)	0.67(0.60,0.73)	0.40(0.36,0.44)	169
Antigua and Barbuda	0(0-0)	181	1(1-1)	177	5.48(5.42,5.53)	2.07(1.91,2.22)	0.02(-0.03,0.06)	2.51(2.46,2.56)	77
Bahamas	1(1-2)	165	2(2-3)	163	2.87(2.82,2.92)	1.88(1.81,1.95)	1.24(1.21,1.26)	1.98(1.95,2.01)	105
Barbados	2(1-2)	164	2(2-2)	166	2.30(2.22,2.38)	1.22(1.18,1.26)	0.17(0.12,0.22)	1.22(1.19,1.26)	134
Belize	1(0-1)	174	2(1-2)	173	3.69(3.67,3.71)	4.19(4.16,4.22)	3.77(3.69,3.85)	3.89(3.86,3.92)	17
Cuba	59(48-70)	66	68(58-79)	91	1.99(1.85,2.14)	0.11(0.06,0.16)	-0.51(-0.53,-0.49)	0.50(0.45,0.55)	162
Dominica	0(0-0)	186	0(0-0)	190	0.41(0.37,0.46)	0.39(0.37,0.41)	0.64(0.60,0.69)	0.48(0.45,0.50)	163
Dominican Republic	23(18-29)	106	44(35-53)	108	2.39(2.28,2.49)	2.04(2.00,2.08)	2.23(2.19,2.27)	2.21(2.17,2.25)	93
Grenada	0(0-0)	182	1(0-1)	181	4.62(4.40,4.85)	1.22(0.99,1.45)	-0.57(-0.63,-0.52)	1.76(1.66,1.87)	115
Guyana	2(1-2)	162	2(2-3)	167	0.79(0.70,0.87)	0.29(0.27,0.31)	1.15(1.05,1.25)	0.71(0.67,0.75)	156
Haiti	24(19-29)	103	61(49-73)	95	3.43(3.38,3.47)	3.51(3.46,3.56)	2.89(2.84,2.95)	3.26(3.22,3.31)	39
Jamaica	8(6-9)	140	12(9-14)	145	1.91(1.85,1.97)	1.22(1.18,1.25)	1.30(1.03,1.56)	1.48(1.39,1.57)	121
Saint Lucia	0(0-1)	178	1(1-1)	178	2.86(2.81,2.92)	2.02(1.97,2.07)	0.94(0.90,0.98)	1.94(1.91,1.97)	108
Saint Vincent and the Grenadines	0(0-0)	184	0(0-1)	187	1.54(1.48,1.61)	1.01(0.94,1.07)	0.54(0.51,0.57)	1.02(0.99,1.05)	145
Suriname	1(1-1)	172	2(1-2)	172	2.85(2.80,2.91)	2.60(2.57,2.63)	0.78(0.74,0.81)	2.10(2.08,2.13)	102
Trinidad and Tobago	3(3-4)	154	5(4-6)	156	2.13(2.07,2.20)	1.39(1.33,1.45)	0.32(0.28,0.36)	1.29(1.26,1.32)	132
Bolivia (Plurinational State of)	19(15-23)	115	47(37-56)	103	3.79(3.78,3.81)	3.37(3.34,3.41)	2.49(2.47,2.50)	3.20(3.19,3.22)	46

Ecuador	18(14-22)	118	45(36-53)	107	4.11(4.04,4.18)	3.06(3.02,3.10)	2.53(2.43,2.62)	3.21(3.17,3.26)	44
Peru	49(39-60)	76	115(92-136)	66	3.58(3.43,3.72)	2.82(2.77,2.87)	2.64(2.60,2.68)	3.00(2.94,3.06)	53
Colombia	57(45-69)	68	114(91-136)	67	3.91(3.79,4.04)	2.21(2.05,2.37)	1.05(0.99,1.11)	2.36(2.29,2.44)	86
Costa Rica	8(6-10)	138	18(14-21)	138	4.91(4.72,5.10)	2.69(2.53,2.85)	0.57(0.41,0.73)	2.72(2.60,2.83)	66
El Salvador	11(8-13)	130	18(14-21)	139	2.25(2.14,2.35)	1.59(1.57,1.62)	1.48(1.47,1.50)	1.76(1.73,1.80)	114
Guatemala	16(13-20)	122	55(44-67)	98	4.46(4.42,4.50)	4.56(4.54,4.59)	3.87(3.82,3.93)	4.31(4.28,4.33)	11
Honduras	9(7-11)	136	27(21-33)	123	3.93(3.87,3.99)	3.97(3.94,3.99)	3.41(3.39,3.44)	3.77(3.75,3.79)	21
Mexico	301(242-362)	25	700(578-818)	17	4.39(4.33,4.46)	2.79(2.75,2.84)	1.71(1.66,1.75)	2.95(2.92,2.99)	55
Nicaragua	7(6-9)	143	18(14-21)	136	4.36(4.29,4.43)	3.08(3.03,3.13)	2.26(2.21,2.31)	3.23(3.19,3.27)	42
Panama	5(4-6)	147	10(8-12)	148	3.46(3.38,3.53)	2.50(2.42,2.59)	1.64(1.60,1.69)	2.51(2.47,2.56)	78
Venezuela (Bolivarian Republic of)	40(32-48)	82	83(68-98)	81	4.42(4.07,4.76)	2.98(2.78,3.18)	-0.07(-0.41,0.27)	2.46(2.27,2.65)	79
Brazil	1,020(840-1,198)	11	1,923(1,603-2,265)	6	2.74(2.36,3.12)	2.31(2.23,2.40)	1.43(0.90,1.96)	2.17(1.96,2.38)	99
Paraguay	21(16-25)	111	45(36-54)	106	2.32(2.29,2.35)	2.67(2.64,2.70)	3.05(2.78,3.32)	2.68(2.60,2.77)	69
Algeria	287(235-347)	26	684(567-810)	18	4.18(4.10,4.26)	3.15(3.10,3.20)	1.75(1.68,1.83)	3.03(2.98,3.07)	49
Bahrain	6(4-7)	145	21(17-26)	130	3.46(3.30,3.63)	9.27(9.00,9.54)	1.63(1.21,2.05)	4.67(4.48,4.86)	7
Egypt	353(282-423)	21	818(658-986)	15	3.09(2.97,3.22)	2.93(2.90,2.96)	2.85(2.80,2.90)	2.96(2.91,3.00)	54
Iran (Islamic Republic of)	1,069(903-1,245)	10	1,913(1,643-2,191)	7	1.11(0.74,1.49)	3.50(3.26,3.74)	1.46(1.29,1.64)	2.13(1.96,2.29)	101
Iraq	180(143-219)	38	607(481-749)	21	4.95(4.15,5.76)	3.47(2.39,4.57)	4.77(4.58,4.96)	4.40(3.96,4.84)	10
Jordan	58(51-66)	67	202(163-241)	48	3.31(3.04,3.59)	3.57(1.11,6.09)	5.84(5.22,6.46)	4.26(3.45,5.07)	14
Kuwait	25(20-30)	102	101(82-120)	72	3.27(2.86,3.68)	6.36(6.17,6.55)	4.99(4.67,5.30)	4.96(4.77,5.16)	5
Lebanon	43(36-52)	80	102(84-121)	71	2.83(2.81,2.85)	2.83(2.81,2.85)	3.39(3.10,3.67)	3.00(2.91,3.09)	52
Libya	44(36-53)	79	119(101-140)	64	4.80(4.53,5.07)	4.21(4.06,4.35)	1.35(1.18,1.52)	3.48(3.36,3.61)	27
Morocco	301(245-362)	24	565(471-673)	24	2.73(2.69,2.76)	2.06(2.02,2.10)	1.76(1.72,1.81)	2.20(2.18,2.22)	96
Palestine	22(18-27)	109	74(60-90)	87	4.92(4.90,4.94)	4.39(4.33,4.45)	3.48(3.43,3.53)	4.27(4.24,4.30)	12
Oman	19(15-22)	116	82(67-100)	82	3.88(3.70,4.06)	3.88(3.70,4.06)	7.10(5.90,8.32)	5.23(4.76,5.70)	4
Qatar	7(6-9)	141	85(73-99)	76	7.11(6.04,8.18)	13.02(12.60,13.44)	6.02(5.60,6.45)	8.82(8.41,9.23)	1
Saudi Arabia	131(103-160)	48	487(387-593)	30	4.13(3.92,4.35)	5.08(5.00,5.17)	4.74(4.60,4.88)	4.67(4.58,4.77)	8
Syrian Arab Republic	140(110-172)	44	210(172-253)	46	3.93(3.80,4.06)	3.93(3.80,4.06)	-4.03(-4.87,-3.18)	1.39(1.10,1.68)	126
Tunisia	116(95-140)	51	214(177-252)	45	3.34(3.28,3.40)	2.07(1.99,2.15)	0.98(0.88,1.08)	2.14(2.09,2.19)	100
Turkey	1,017(972-1,063)	12	1,562(1,484-1,647)	10	2.20(2.10,2.31)	1.13(1.06,1.20)	1.16(0.73,1.59)	1.47(1.33,1.61)	122
United Arab Emirates	25(21-31)	99	152(120-190)	52	6.01(5.49,6.53)	13.70(11.88,15.55)	0.94(0.49,1.40)	6.58(5.99,7.16)	2
Yemen	80(63-98)	61	268(216-324)	38	4.34(4.29,4.39)	4.72(4.69,4.74)	3.69(3.63,3.75)	4.26(4.23,4.29)	13
Afghanistan	133(111-158)	47	588(490-704)	23	7.13(5.61,8.68)	4.69(4.58,4.81)	4.69(4.58,4.81)	5.28(4.72,5.84)	3
Bangladesh	327(258-395)	22	616(497-748)	20	2.38(2.35,2.42)	2.21(2.20,2.23)	2.02(1.96,2.08)	2.20(2.18,2.23)	97
Bhutan	2(2-3)	160	3(3-4)	160	0.79(0.28,1.31)	2.29(2.13,2.45)	1.80(1.55,2.05)	1.70(1.49,1.91)	119
India	2,756(2,197-3,351)	2	5,732(4,618-6,867)	2	2.48(2.45,2.51)	2.70(2.69,2.72)	2.49(2.43,2.56)	2.56(2.54,2.59)	75
Nepal	67(54-81)	63	132(106-161)	61	2.24(2.17,2.31)	2.41(2.39,2.42)	2.48(2.31,2.65)	2.38(2.32,2.44)	84
Pakistan	407(327-492)	19	963(769-1,160)	14	2.49(2.42,2.56)	3.10(3.05,3.15)	3.45(3.30,3.60)	3.02(2.97,3.08)	50

Angola	23(18-28)	108	69(53-85)	90	3.31(3.28,3.33)	4.04(3.98,4.09)	4.26(4.24,4.28)	3.88(3.86,3.90)	18
Central African Republic	5(4-6)	146	11(9-14)	146	2.65(2.62,2.68)	2.72(2.62,2.82)	2.14(1.82,2.46)	2.53(2.40,2.67)	76
Congo	4(3-5)	151	10(8-12)	149	3.47(3.46,3.49)	3.44(3.42,3.46)	2.88(2.83,2.93)	3.27(3.24,3.30)	38
Democratic Republic of the Congo	59(46-74)	65	148(115-185)	55	2.73(2.68,2.77)	3.27(3.24,3.29)	3.69(3.65,3.73)	3.23(3.21,3.25)	43
Equatorial Guinea	1(1-1)	173	2(2-3)	164	3.95(3.89,4.00)	4.99(4.98,5.01)	4.80(4.76,4.84)	4.58(4.56,4.61)	9
Gabon	2(1-2)	163	3(3-4)	159	2.93(2.91,2.94)	2.76(2.74,2.79)	2.44(2.41,2.48)	2.71(2.70,2.73)	67
Burundi	9(7-11)	135	20(16-26)	131	0.82(0.48,1.16)	3.91(3.77,4.04)	3.91(3.77,4.04)	2.83(2.69,2.97)	59
Comoros	1(1-1)	170	2(2-2)	168	2.29(2.24,2.34)	2.17(2.15,2.19)	2.33(2.28,2.38)	2.26(2.23,2.28)	89
Djibouti	1(1-1)	168	3(3-4)	161	3.36(2.95,3.77)	4.52(4.47,4.57)	3.91(3.72,4.11)	3.97(3.83,4.12)	16
Eritrea	8(6-10)	139	20(16-25)	133	3.59(3.47,3.71)	3.53(3.43,3.63)	3.08(2.83,3.33)	3.39(3.28,3.51)	31
Ethiopia	102(80-127)	56	226(176-283)	42	2.25(2.19,2.30)	2.50(2.45,2.55)	3.66(3.61,3.71)	2.78(2.75,2.82)	60
Kenya	36(28-45)	85	96(74-119)	73	3.46(3.45,3.47)	3.38(3.37,3.38)	3.56(3.54,3.58)	3.46(3.45,3.47)	28
Madagascar	33(26-41)	89	84(65-103)	79	2.60(2.55,2.65)	3.21(3.15,3.26)	3.92(3.85,3.99)	3.24(3.20,3.28)	41
Malawi	22(17-27)	110	46(36-57)	105	1.10(0.55,1.65)	2.58(2.51,2.65)	3.94(3.85,4.02)	2.57(2.39,2.75)	74
Mauritius	2(2-3)	159	3(3-4)	162	1.42(1.40,1.44)	1.07(1.00,1.14)	1.08(1.01,1.15)	1.19(1.16,1.23)	135
Mozambique	35(28-43)	86	82(64-101)	83	2.82(2.17,3.47)	2.59(2.50,2.68)	3.40(3.32,3.49)	2.93(2.72,3.14)	56
Rwanda	11(9-14)	128	23(18-29)	127	0.53(-1.91,3.02)	3.06(2.88,3.23)	3.06(2.88,3.23)	2.26(1.48,3.06)	90
Seychelles	0(0-0)	193	0(0-0)	192	3.02(2.99,3.05)	1.91(1.87,1.94)	0.43(0.17,0.68)	1.79(1.71,1.88)	113
Somalia	13(10-16)	125	37(28-47)	116	3.57(3.39,3.76)	3.66(3.59,3.73)	4.28(4.19,4.38)	3.82(3.74,3.89)	20
United Republic of Tanzania	45(35-56)	78	109(85-137)	69	2.92(2.88,2.96)	2.92(2.90,2.94)	3.41(3.38,3.44)	3.08(3.06,3.09)	47
Uganda	24(18-30)	104	62(47-77)	94	2.95(2.91,2.99)	3.29(3.27,3.30)	3.74(3.69,3.78)	3.32(3.30,3.34)	35
Zambia	19(15-23)	114	48(37-59)	102	1.94(1.90,1.98)	3.56(3.41,3.71)	4.27(4.25,4.29)	3.26(3.21,3.32)	40
Botswana	3(3-4)	153	8(6-10)	152	3.24(3.17,3.30)	2.69(2.65,2.74)	2.90(2.83,2.96)	2.93(2.89,2.97)	57
Lesotho	6(5-7)	144	9(7-11)	150	1.11(0.99,1.22)	0.69(0.58,0.79)	2.09(1.97,2.21)	1.27(1.19,1.34)	133
Namibia	4(3-5)	152	7(6-9)	154	2.83(2.76,2.91)	1.88(1.81,1.94)	2.32(2.31,2.34)	2.34(2.31,2.38)	87
South Africa	135(107-165)	45	233(185-279)	41	2.00(1.95,2.05)	1.74(1.70,1.77)	1.97(1.87,2.06)	1.90(1.86,1.93)	110
Eswatini	2(2-3)	158	4(3-5)	157	2.72(2.55,2.90)	1.24(1.14,1.35)	2.10(2.04,2.16)	2.02(1.95,2.10)	104
Zimbabwe	24(19-30)	105	41(32-51)	110	2.13(2.04,2.22)	1.26(1.19,1.33)	2.45(2.36,2.54)	1.91(1.86,1.97)	109
Benin	12(10-15)	126	36(28-44)	118	3.66(3.64,3.67)	3.81(3.78,3.84)	3.74(3.67,3.80)	3.74(3.71,3.76)	22
Burkina Faso	26(21-32)	97	69(55-85)	89	2.50(2.40,2.60)	3.83(3.81,3.85)	3.83(3.81,3.85)	3.39(3.35,3.43)	32
Cameroon	26(20-32)	98	83(65-102)	80	4.20(4.09,4.31)	4.36(4.28,4.44)	3.70(3.60,3.80)	4.10(4.04,4.16)	15
Cabo Verde	1(1-1)	169	2(2-3)	165	3.04(3.01,3.08)	2.95(2.87,3.03)	2.25(2.22,2.29)	2.77(2.74,2.80)	62
Chad	18(14-22)	117	50(39-61)	100	2.86(2.85,2.88)	3.59(3.57,3.62)	4.21(4.19,4.24)	3.55(3.54,3.56)	25
C??te d'Ivoire	32(25-39)	92	79(62-97)	84	4.21(4.10,4.31)	3.15(3.09,3.21)	2.30(2.26,2.35)	3.21(3.16,3.25)	45
Gambia	3(2-4)	157	8(6-10)	153	3.82(3.72,3.91)	3.29(3.26,3.31)	3.46(3.44,3.48)	3.51(3.48,3.54)	26
Ghana	78(65-90)	62	296(263-333)	35	5.81(5.72,5.90)	4.35(4.30,4.39)	4.04(3.98,4.10)	4.71(4.67,4.75)	6
Guinea	17(14-21)	120	38(31-47)	112	2.63(2.50,2.76)	2.64(2.49,2.79)	3.14(3.09,3.19)	2.77(2.69,2.85)	61
Guinea-Bissau	3(2-4)	155	7(5-8)	155	2.33(2.30,2.36)	2.99(2.96,3.02)	2.92(2.83,3.02)	2.74(2.71,2.77)	64

Liberia	5(4-6)	148	13(10-16)	142	3.65(1.38,5.97)	4.03(3.70,4.36)	3.15(2.48,3.83)	3.64(2.88,4.40)	24
Mali	29(23-34)	94	75(60-91)	86	2.17(2.07,2.27)	3.72(3.69,3.76)	4.18(4.11,4.24)	3.35(3.30,3.39)	34
Mauritania	8(6-10)	137	18(14-21)	137	2.65(2.64,2.65)	2.73(2.71,2.74)	2.85(2.82,2.87)	2.73(2.72,2.74)	65
Niger	25(20-31)	101	72(57-87)	88	3.34(3.30,3.38)	3.52(3.50,3.53)	4.15(4.12,4.18)	3.67(3.65,3.68)	23
Nigeria	229(182-282)	31	599(474-736)	22	3.14(3.10,3.19)	3.44(3.42,3.46)	3.52(3.43,3.61)	3.37(3.34,3.41)	33
Sao Tome and Principe	0(0-0)	189	0(0-1)	185	2.23(2.19,2.27)	3.14(3.12,3.16)	2.87(2.84,2.89)	2.75(2.73,2.78)	63
Senegal	23(18-28)	107	54(43-66)	99	3.38(3.36,3.40)	2.93(2.89,2.97)	2.76(2.71,2.80)	3.02(3.00,3.04)	51
Sierra Leone	9(7-12)	133	24(19-30)	126	1.52(1.19,1.85)	4.26(4.12,4.40)	3.80(3.75,3.84)	3.30(3.16,3.43)	36
Togo	9(7-11)	134	25(20-30)	125	3.76(3.70,3.81)	3.49(3.44,3.54)	2.99(2.96,3.03)	3.42(3.39,3.45)	30
American Samoa	0(0-0)	197	0(0-0)	196	2.25(2.17,2.33)	0.23(0.21,0.26)	0.53(0.49,0.57)	0.99(0.96,1.03)	148
Bermuda	0(0-1)	177	0(0-1)	188	0.77(0.69,0.85)	0.15(0.08,0.22)	-0.59(-0.64,-0.54)	0.11(0.07,0.15)	178
Cook Islands	0(0-0)	199	0(0-0)	199	0.56(0.51,0.61)	0.65(0.53,0.76)	0.27(0.19,0.34)	0.46(0.42,0.51)	166
Greenland	2(1-2)	161	1(1-2)	174	-4.81(-4.96,-4.65)	0.55(0.24,0.86)	1.87(1.38,2.37)	-0.86(-1.05,-0.67)	194
Guam	0(0-0)	191	0(0-0)	191	1.64(1.57,1.71)	0.78(0.76,0.80)	0.72(0.71,0.74)	1.04(1.01,1.07)	144
Monaco	0(0-1)	175	1(1-1)	179	0.77(0.71,0.82)	0.81(0.79,0.83)	0.80(0.73,0.86)	0.79(0.76,0.82)	153
Nauru	0(0-0)	202	0(0-0)	202	1.13(1.09,1.18)	0.00(-0.17,0.18)	0.10(-0.02,0.23)	0.41(0.34,0.48)	168
Niue	0(0-0)	203	0(0-0)	203	-0.86(-0.91,-0.81)	-1.62(-1.77,-1.47)	0.69(0.66,0.73)	-0.62(-0.68,-0.57)	192
Northern Mariana Islands	0(0-0)	196	0(0-0)	197	4.94(4.00,5.88)	-3.28(-4.28,-2.28)	-2.12(-2.31,-1.92)	0.08(-0.38,0.55)	180
Palau	0(0-0)	200	0(0-0)	200	3.68(3.57,3.79)	0.01(-0.11,0.14)	0.06(-0.05,0.17)	1.16(1.09,1.24)	139
Puerto Rico	16(14-19)	121	19(16-22)	134	1.93(1.84,2.02)	0.23(0.18,0.28)	-0.86(-0.98,-0.74)	0.42(0.37,0.48)	167
Saint Kitts and Nevis	0(0-0)	185	1(0-1)	182	4.01(3.93,4.09)	2.42(2.04,2.81)	0.20(0.05,0.36)	2.21(2.07,2.35)	94
San Marino	0(0-0)	183	0(0-1)	186	1.03(0.88,1.18)	1.01(0.93,1.09)	1.46(1.39,1.53)	1.16(1.10,1.23)	138
Tokelau	0(0-0)	204	0(0-0)	204	-0.32(-0.58,-0.06)	-2.12(-2.40,-1.83)	1.30(1.21,1.40)	-0.28(-0.43,-0.14)	187
Tuvalu	0(0-0)	201	0(0-0)	201	0.42(0.37,0.46)	1.14(1.08,1.20)	1.38(1.37,1.40)	0.98(0.96,1.01)	149
United States Virgin Islands	0(0-1)	176	0(0-1)	183	1.19(1.13,1.25)	0.10(-0.03,0.23)	-0.52(-0.54,-0.49)	0.24(0.20,0.29)	175
South Sudan	11(8-13)	129	18(14-22)	135	1.93(1.86,2.00)	2.64(2.58,2.69)	0.60(0.26,0.95)	1.76(1.65,1.87)	116
Sudan	131(103-160)	49	352(282-426)	34	3.74(3.63,3.86)	3.06(2.89,3.23)	3.63(3.49,3.76)	3.45(3.36,3.54)	29
Rheumatoid arthritis									
China	125,073(112,795-138,598)	1	222,509(198,118-247,768)	2	2.49(2.48,2.50)	2.49(2.48,2.50)	0.93(0.70,1.17)	2.00(1.93,2.08)	127
Democratic People's Republic of Korea	2,232(1,979-2,522)	35	3,109(2,732-3,529)	44	1.11(0.99,1.23)	1.24(1.14,1.35)	1.06(0.97,1.15)	1.13(1.06,1.19)	168
Taiwan (Province of China)	2,013(1,965-2,077)	40	3,459(3,410-3,508)	43	2.24(2.22,2.25)	2.14(2.12,2.15)	1.26(1.24,1.28)	1.88(1.87,1.89)	134
Cambodia	337(291-395)	103	832(729-962)	95	2.95(2.84,3.06)	3.50(3.41,3.59)	3.04(3.02,3.07)	3.17(3.11,3.22)	75
Indonesia	6,621(5,909-7,473)	15	11,396(10,134-12,825)	15	2.13(2.08,2.18)	1.86(1.82,1.89)	1.70(1.67,1.73)	1.89(1.87,1.92)	133
Lao People's Democratic Republic	137(118-159)	131	337(293-394)	130	2.88(2.86,2.91)	3.42(3.37,3.46)	3.14(3.12,3.16)	3.15(3.13,3.16)	77
Malaysia	638(545-751)	71	1,576(1,340-1,856)	70	4.21(4.05,4.36)	2.98(2.84,3.12)	2.27(2.23,2.31)	3.17(3.10,3.25)	76
Maldives	8(7-9)	178	31(26-37)	173	4.37(4.14,4.61)	5.50(5.38,5.62)	4.79(4.58,5.01)	4.94(4.81,5.06)	9
Myanmar	1,717(1,486-1,990)	44	3,502(3,095-3,992)	41	2.17(2.14,2.20)	2.65(2.64,2.67)	2.61(2.59,2.64)	2.49(2.47,2.50)	109
Philippines	2,983(2,668-3,336)	28	6,048(5,412-6,784)	29	2.88(2.81,2.96)	2.53(2.42,2.64)	2.07(1.80,2.35)	2.51(2.41,2.61)	107

Sri Lanka	662(563-784)	70	1,136(979-1,322)	82	1.77(1.69,1.84)	2.16(2.14,2.18)	1.70(1.66,1.74)	1.87(1.84,1.90)	135
Thailand	4,346(3,806-5,054)	20	9,036(7,841-10,379)	20	3.10(2.75,3.46)	3.57(3.47,3.66)	0.74(-0.36,1.84)	2.54(2.17,2.91)	104
Timor-Leste	25(21-30)	164	53(46-62)	166	1.92(1.87,1.97)	2.83(2.55,3.10)	3.07(2.97,3.18)	2.61(2.51,2.70)	100
Viet Nam	2,548(2,187-2,987)	31	6,149(5,286-7,110)	28	3.19(3.16,3.23)	3.33(3.28,3.37)	2.73(2.70,2.76)	3.10(3.08,3.12)	79
Fiji	27(23-33)	162	38(32-45)	172	1.23(1.08,1.39)	1.03(0.97,1.08)	1.25(1.12,1.39)	1.17(1.10,1.25)	163
Kiribati	2(2-3)	196	4(3-5)	195	1.87(1.76,1.98)	2.41(2.35,2.46)	1.87(1.74,2.00)	2.04(1.98,2.11)	126
Marshall Islands	2(1-2)	198	3(2-3)	197	2.63(2.59,2.67)	1.58(1.56,1.60)	1.34(1.30,1.38)	1.83(1.81,1.85)	137
Micronesia (Federated States of)	4(3-4)	189	5(4-6)	191	1.99(1.93,2.05)	0.63(0.59,0.66)	0.55(0.51,0.59)	1.03(1.00,1.06)	172
Papua New Guinea	130(111-155)	135	353(302-415)	128	3.52(3.47,3.57)	3.44(3.38,3.49)	3.50(3.49,3.51)	3.49(3.47,3.52)	56
Samoa	6(5-8)	182	10(8-11)	184	1.69(1.54,1.85)	0.63(0.59,0.68)	1.91(1.82,2.01)	1.41(1.34,1.47)	153
Solomon Islands	13(11-15)	173	31(27-36)	174	4.20(4.04,4.36)	2.36(2.19,2.53)	2.54(2.45,2.62)	3.02(2.90,3.15)	82
Tonga	3(3-4)	190	4(4-5)	193	1.36(1.30,1.41)	0.55(0.49,0.60)	0.34(0.18,0.49)	0.74(0.68,0.80)	181
Vanuatu	5(4-6)	186	12(10-14)	180	3.16(3.09,3.24)	2.90(2.61,3.20)	2.56(2.48,2.64)	2.87(2.78,2.97)	87
Armenia	205(179-236)	119	277(243-315)	136	1.18(0.96,1.40)	1.04(0.96,1.13)	1.03(0.83,1.23)	1.09(0.98,1.19)	170
Azerbaijan	382(323-452)	97	794(674-931)	97	1.55(1.28,1.81)	3.09(2.64,3.53)	3.11(2.95,3.28)	2.60(2.42,2.77)	101
Georgia	411(346-477)	90	314(265-364)	133	-1.56(-1.75,-1.36)	-1.17(-1.21,-1.13)	0.06(-0.49,0.61)	-0.91(-1.09,-0.73)	204
Kazakhstan	2,702(2,466-2,987)	30	4,822(4,374-5,335)	36	-0.21(-0.33,-0.10)	2.41(2.30,2.53)	3.92(3.82,4.02)	2.06(1.99,2.13)	125
Kyrgyzstan	536(475-603)	79	1,147(1,019-1,300)	81	1.98(1.88,2.09)	2.85(2.79,2.90)	3.13(3.09,3.17)	2.67(2.62,2.71)	98
Mongolia	124(107-145)	136	334(286-389)	131	2.58(2.41,2.75)	4.75(4.67,4.84)	3.06(2.97,3.14)	3.50(3.43,3.57)	53
Tajikistan	383(335-442)	95	962(838-1,117)	87	2.30(2.26,2.35)	3.72(3.69,3.76)	3.58(3.55,3.62)	3.22(3.19,3.25)	74
Turkmenistan	171(146-203)	123	355(308-409)	127	2.50(2.31,2.70)	2.39(2.21,2.57)	2.76(2.71,2.81)	2.58(2.49,2.67)	102
Uzbekistan	3,036(2,681-3,426)	27	7,167(6,392-7,983)	24	3.08(3.04,3.12)	3.27(3.13,3.41)	2.64(2.42,2.85)	3.02(2.93,3.11)	81
Albania	185(158-215)	121	277(241-314)	137	1.95(1.63,2.27)	1.34(0.99,1.70)	1.17(1.03,1.32)	1.41(1.16,1.66)	151
Bosnia and Herzegovina	490(436-544)	84	562(495-629)	110	0.31(0.01,0.62)	0.86(0.62,1.10)	-0.15(-0.24,-0.06)	0.47(0.33,0.61)	190
Bulgaria	576(497-658)	75	540(468-621)	113	-0.74(-0.87,-0.61)	-0.07(-0.15,0.01)	0.30(0.12,0.48)	-0.18(-0.27,-0.09)	200
Croatia	589(522-661)	74	702(621-793)	101	0.52(0.49,0.55)	0.62(0.59,0.65)	0.65(0.45,0.85)	0.60(0.53,0.66)	186
Czechia	877(756-1,002)	62	1,178(1,009-1,355)	79	1.22(1.12,1.31)	0.95(0.82,1.07)	1.06(0.72,1.40)	1.07(0.95,1.19)	171
Hungary	1,222(1,113-1,340)	50	1,389(1,256-1,532)	75	1.15(0.90,1.40)	0.59(0.49,0.68)	-0.63(-0.84,-0.43)	0.40(0.28,0.52)	192
North Macedonia	118(101-137)	138	181(155-208)	149	1.40(0.44,2.36)	1.56(1.49,1.64)	1.56(1.49,1.64)	1.51(1.21,1.82)	146
Montenegro	45(39-52)	155	60(52-69)	163	0.50(0.39,0.61)	1.24(1.21,1.27)	1.24(1.21,1.27)	1.01(0.97,1.05)	173
Poland	7,830(7,005-8,682)	13	7,813(6,942-8,685)	22	1.29(1.17,1.41)	0.86(0.72,1.00)	-2.09(-3.16,-1.02)	0.06(-0.29,0.41)	197
Romania	1,479(1,258-1,712)	48	1,691(1,440-1,943)	65	0.67(0.50,0.84)	0.24(0.18,0.30)	0.53(0.39,0.66)	0.49(0.41,0.57)	189
Serbia	717(655-783)	68	928(817-1,040)	92	0.47(0.36,0.59)	0.63(0.49,0.77)	1.73(1.32,2.15)	0.92(0.76,1.08)	175
Slovakia	332(287-382)	104	473(406-542)	117	1.42(1.36,1.49)	1.11(1.07,1.16)	1.21(0.87,1.55)	1.25(1.14,1.36)	160
Slovenia	280(248-316)	111	434(384-488)	119	1.53(1.49,1.57)	2.08(2.04,2.12)	0.91(0.79,1.04)	1.53(1.48,1.57)	144
Belarus	530(455-615)	80	655(564-759)	102	0.28(0.10,0.46)	0.80(0.73,0.88)	1.20(1.12,1.29)	0.77(0.70,0.85)	179
Estonia	241(219-261)	112	257(235-279)	141	-0.60(-0.78,-0.42)	1.42(1.31,1.54)	-0.45(-0.66,-0.23)	0.20(0.09,0.31)	194
Latvia	359(322-398)	100	316(285-350)	132	-0.83(-0.98,-0.68)	0.35(0.24,0.46)	-0.96(-1.06,-0.86)	-0.41(-0.49,-0.34)	203

Lithuania	519(475-571)	82	472(425-519)	118	0.47(0.30,0.64)	-0.49(-0.55,-0.42)	-1.13(-1.23,-1.04)	-0.31(-0.39,-0.24)	202
Republic of Moldova	233(202-269)	114	261(226-300)	140	0.09(-0.08,0.26)	0.29(0.21,0.36)	0.88(0.79,0.97)	0.43(0.36,0.50)	191
Russian Federation	12,644(11,333-14,137)	9	13,117(11,729-14,661)	10	0.57(0.56,0.59)	0.02(0.00,0.04)	-0.26(-0.29,-0.22)	0.12(0.11,0.13)	196
Ukraine	2,900(2,577-3,246)	29	2,691(2,382-3,046)	48	0.11(0.00,0.22)	-0.41(-0.49,-0.33)	-0.40(-0.81,0.01)	-0.25(-0.38,-0.11)	201
Brunei Darussalam	18(16-22)	171	50(44-58)	168	4.58(4.50,4.65)	3.47(3.31,3.62)	2.38(2.29,2.47)	3.49(3.42,3.55)	54
Japan	24,652(22,071-27,440)	4	26,450(23,575-29,571)	8	1.08(0.90,1.26)	0.73(0.57,0.89)	-1.41(-1.78,-1.03)	0.18(0.02,0.34)	195
Republic of Korea	4,503(4,065-5,000)	19	10,055(9,083-11,055)	18	3.15(3.10,3.20)	3.05(2.99,3.11)	2.03(1.88,2.18)	2.76(2.70,2.83)	94
Singapore	222(189-261)	117	599(505-697)	107	4.03(3.85,4.22)	3.82(2.96,4.69)	2.64(2.40,2.89)	3.57(3.28,3.86)	45
Australia	3,399(3,062-3,788)	24	6,609(5,898-7,383)	25	2.68(2.47,2.89)	2.96(2.83,3.09)	1.06(0.80,1.32)	2.27(2.14,2.40)	121
New Zealand	848(754-949)	63	1,360(1,206-1,515)	76	1.73(1.61,1.86)	2.25(2.21,2.30)	0.84(0.67,1.00)	1.63(1.56,1.70)	141
Andorra	9(8-10)	175	19(16-22)	177	3.57(3.39,3.75)	3.57(3.39,3.75)	0.88(-0.53,2.30)	2.50(1.86,3.15)	108
Austria	1,096(986-1,219)	57	1,445(1,297-1,605)	73	1.32(1.02,1.62)	1.37(1.22,1.52)	-0.05(-0.33,0.23)	0.92(0.76,1.07)	176
Belgium	1,542(1,390-1,707)	46	2,144(1,926-2,384)	55	1.21(1.11,1.31)	1.43(1.40,1.46)	0.68(0.58,0.78)	1.13(1.08,1.18)	166
Cyprus	140(125-157)	130	368(328-417)	122	3.14(3.04,3.23)	4.54(4.43,4.64)	2.37(2.09,2.66)	3.38(3.28,3.48)	64
Denmark	1,176(1,082-1,287)	53	1,778(1,618-1,960)	63	1.01(0.93,1.08)	1.87(1.82,1.92)	1.44(1.38,1.50)	1.46(1.42,1.50)	148
Finland	1,534(1,421-1,660)	47	2,238(2,055-2,440)	53	2.21(2.13,2.30)	2.21(2.13,2.30)	-0.55(-1.05,-0.06)	1.30(1.12,1.49)	158
France	7,696(6,950-8,538)	14	11,644(10,370-13,077)	14	1.59(1.52,1.65)	1.87(1.81,1.94)	0.75(0.65,0.85)	1.43(1.38,1.47)	149
Germany	10,581(9,466-11,815)	10	13,103(11,573-14,682)	11	1.49(1.42,1.56)	0.87(0.80,0.94)	-0.31(-0.48,-0.15)	0.70(0.63,0.77)	182
Greece	1,211(1,078-1,350)	51	1,423(1,262-1,615)	74	1.28(1.21,1.35)	0.65(0.45,0.85)	0.00(-0.25,0.25)	0.62(0.51,0.73)	185
Iceland	32(28-36)	161	54(48-60)	165	2.15(2.05,2.26)	2.41(2.35,2.47)	0.87(0.74,1.01)	1.81(1.75,1.88)	139
Ireland	923(839-1,007)	60	1,895(1,697-2,099)	61	2.51(2.27,2.75)	3.67(3.50,3.84)	1.07(0.68,1.46)	2.47(2.30,2.65)	110
Israel	457(403-523)	88	1,097(967-1,255)	83	3.48(3.44,3.52)	3.09(2.97,3.22)	2.69(2.58,2.80)	3.08(3.02,3.14)	80
Italy	10,212(9,159-11,376)	11	12,477(11,196-13,935)	12	0.58(0.55,0.61)	0.86(0.84,0.89)	0.64(0.62,0.66)	0.70(0.69,0.72)	183
Luxembourg	48(43-54)	154	94(83-106)	159	2.13(2.03,2.23)	2.51(2.49,2.53)	2.16(2.05,2.27)	2.28(2.24,2.33)	120
Malta	42(37-48)	157	63(57-70)	161	2.21(2.17,2.25)	1.69(1.65,1.72)	0.34(0.08,0.61)	1.43(1.35,1.52)	150
Netherlands	3,683(3,336-4,094)	23	5,101(4,580-5,703)	35	1.62(1.53,1.72)	2.26(2.23,2.30)	-0.77(-1.12,-0.41)	1.11(1.00,1.23)	169
Norway	1,156(1,031-1,289)	55	1,612(1,430-1,802)	67	0.26(0.09,0.43)	1.89(1.81,1.97)	1.19(0.48,1.91)	1.17(0.93,1.40)	164
Portugal	1,095(992-1,215)	58	1,642(1,472-1,821)	66	2.12(2.09,2.15)	1.67(1.63,1.71)	0.36(0.26,0.46)	1.40(1.36,1.44)	154
Spain	4,974(4,611-5,384)	18	8,040(7,400-8,693)	21	2.01(1.97,2.04)	2.37(2.36,2.39)	0.62(0.56,0.68)	1.67(1.64,1.70)	140
Sweden	2,370(2,191-2,572)	33	3,079(2,828-3,345)	45	0.66(0.62,0.70)	0.89(0.87,0.90)	1.12(1.07,1.18)	0.90(0.88,0.93)	177
Switzerland	1,374(1,230-1,535)	49	2,139(1,921-2,406)	56	1.58(1.53,1.63)	1.79(1.78,1.81)	1.17(0.99,1.36)	1.53(1.47,1.59)	145
United Kingdom	17,971(16,307-19,800)	5	21,101(19,159-23,236)	9	0.46(0.24,0.68)	1.02(0.95,1.09)	-0.04(-0.28,0.21)	0.52(0.41,0.63)	188
Argentina	3,135(2,779-3,554)	26	5,588(5,005-6,263)	33	2.51(2.36,2.66)	2.10(2.03,2.16)	1.28(1.09,1.47)	1.97(1.89,2.05)	129
Chile	1,741(1,551-1,966)	43	4,569(4,123-5,066)	40	4.13(4.11,4.16)	3.53(3.44,3.62)	2.52(2.47,2.57)	3.40(3.36,3.43)	63
Uruguay	339(302-380)	102	509(455-571)	114	1.65(1.56,1.74)	1.31(1.26,1.37)	1.18(1.15,1.22)	1.40(1.36,1.43)	155
Canada	5,388(5,155-5,605)	17	11,667(11,143-12,147)	13	3.58(3.43,3.74)	2.51(2.47,2.56)	2.06(2.03,2.08)	2.71(2.65,2.76)	97
United States of America	51,420(47,776-55,594)	3	92,848(85,088-101,348)	3	1.89(1.85,1.93)	2.59(2.57,2.61)	1.67(1.61,1.74)	2.07(2.04,2.09)	124
Antigua and Barbuda	6(5-7)	184	12(10-13)	178	3.47(3.28,3.66)	2.63(2.41,2.86)	1.47(1.39,1.54)	2.53(2.43,2.63)	106

Bahamas	23(20-27)	166	47(41-54)	170	3.32(3.21,3.42)	2.54(2.46,2.61)	1.43(1.38,1.48)	2.43(2.38,2.47)	112
Barbados	34(31-39)	160	55(49-61)	164	1.51(1.46,1.55)	2.06(2.01,2.11)	1.19(1.02,1.37)	1.60(1.54,1.66)	142
Belize	14(12-16)	172	47(40-54)	169	4.37(4.29,4.45)	4.97(4.55,5.39)	3.69(3.60,3.78)	4.32(4.19,4.46)	18
Cuba	1,101(951-1,268)	56	1,610(1,418-1,818)	68	2.04(2.03,2.06)	1.39(1.28,1.49)	0.55(0.53,0.56)	1.32(1.29,1.35)	157
Dominica	7(6-8)	179	9(8-10)	186	1.01(0.98,1.05)	0.56(0.52,0.60)	0.31(0.26,0.36)	0.63(0.60,0.65)	184
Dominican Republic	398(332-474)	92	878(751-1,041)	93	3.43(3.41,3.45)	2.89(2.86,2.92)	2.02(1.98,2.07)	2.77(2.75,2.78)	93
Grenada	5(4-6)	187	9(8-10)	185	3.66(3.21,4.10)	1.64(1.54,1.74)	0.93(0.66,1.19)	2.08(1.91,2.25)	123
Guyana	43(36-51)	156	60(52-70)	162	1.10(0.95,1.26)	0.65(0.58,0.72)	2.03(1.84,2.21)	1.22(1.13,1.30)	161
Haiti	452(393-517)	89	1,237(1,080-1,417)	78	3.78(3.65,3.91)	3.74(3.57,3.91)	3.11(3.04,3.18)	3.55(3.47,3.63)	47
Jamaica	160(137-187)	127	276(240-319)	138	2.58(2.53,2.62)	2.02(1.98,2.07)	1.24(1.09,1.39)	1.93(1.87,1.99)	132
Saint Lucia	12(10-13)	174	25(23-28)	175	3.78(3.75,3.81)	2.77(2.73,2.80)	1.64(1.58,1.70)	2.73(2.71,2.76)	95
Saint Vincent and the Grenadines	7(6-8)	180	10(9-12)	182	1.40(1.34,1.46)	1.65(1.60,1.69)	1.21(1.13,1.30)	1.41(1.37,1.45)	152
Suriname	22(19-26)	169	46(39-53)	171	2.40(2.19,2.61)	3.08(2.99,3.17)	2.11(2.03,2.19)	2.55(2.46,2.64)	103
Trinidad and Tobago	132(117-150)	132	232(208-259)	145	2.61(2.58,2.64)	2.27(2.13,2.41)	1.03(0.91,1.14)	1.97(1.91,2.03)	128
Bolivia (Plurinational State of)	488(430-558)	86	1,564(1,397-1,779)	71	4.57(4.41,4.73)	4.39(4.27,4.52)	3.30(3.22,3.37)	4.09(4.01,4.16)	24
Ecuador	965(852-1,100)	59	3,060(2,733-3,459)	46	5.20(5.14,5.25)	3.67(3.58,3.76)	3.38(3.34,3.43)	4.06(4.02,4.09)	26
Peru	1,585(1,363-1,855)	45	4,594(4,032-5,279)	39	3.86(3.48,4.24)	3.77(3.68,3.85)	3.66(3.59,3.74)	3.79(3.64,3.94)	33
Colombia	3,147(2,744-3,615)	25	7,175(6,431-8,026)	23	4.21(4.15,4.27)	2.83(2.79,2.86)	1.44(1.21,1.66)	2.87(2.79,2.94)	86
Costa Rica	396(350-452)	93	1,093(979-1,219)	84	4.62(4.57,4.68)	3.59(3.47,3.71)	2.58(2.52,2.63)	3.59(3.55,3.64)	42
El Salvador	379(327-443)	98	741(647-852)	100	2.89(2.79,2.99)	2.34(2.29,2.38)	1.71(1.63,1.78)	2.34(2.29,2.38)	116
Guatemala	570(498-660)	76	2,271(1,998-2,612)	51	5.51(5.31,5.70)	4.98(4.84,5.13)	4.14(4.06,4.22)	4.88(4.80,4.97)	11
Honduras	594(530-669)	73	2,092(1,878-2,331)	59	4.74(4.60,4.88)	4.68(4.63,4.73)	3.81(3.73,3.88)	4.42(4.36,4.48)	16
Mexico	16,228(14,706-17,906)	7	32,459(29,222-35,828)	4	2.94(2.76,3.13)	2.03(1.61,2.46)	2.29(2.19,2.40)	2.42(2.27,2.57)	114
Nicaragua	318(276-371)	106	941(826-1,071)	90	4.33(4.17,4.49)	3.96(3.92,4.01)	3.00(2.92,3.08)	3.80(3.73,3.87)	32
Panama	239(211-273)	113	605(537-684)	106	3.64(3.60,3.67)	2.92(2.85,2.98)	3.16(3.10,3.22)	3.25(3.22,3.28)	73
Venezuela (Bolivarian Republic of)	2,445(2,286-2,615)	32	6,421(5,986-6,839)	26	4.25(4.11,4.38)	3.52(3.40,3.64)	2.23(2.07,2.40)	3.35(3.26,3.43)	68
Brazil	16,562(14,840-18,555)	6	27,907(24,836-31,327)	7	2.37(2.32,2.43)	1.72(1.61,1.84)	1.43(1.40,1.46)	1.85(1.81,1.89)	136
Paraguay	332(285-390)	105	953(826-1,093)	89	4.50(4.23,4.76)	3.21(3.16,3.26)	3.56(3.45,3.67)	3.72(3.63,3.81)	37
Algeria	834(709-979)	64	2,294(1,983-2,670)	50	3.59(3.46,3.71)	3.59(3.53,3.65)	3.58(3.45,3.70)	3.58(3.51,3.64)	44
Bahrain	27(23-33)	163	155(132-182)	153	4.51(4.33,4.69)	9.33(9.16,9.51)	4.59(4.47,4.71)	6.26(6.15,6.37)	3
Egypt	1,847(1,557-2,205)	41	4,763(4,071-5,605)	37	3.53(3.36,3.71)	3.00(2.95,3.06)	3.49(3.34,3.64)	3.35(3.26,3.43)	67
Iran (Islamic Republic of)	2,314(2,071-2,597)	34	4,684(4,128-5,303)	38	2.97(2.72,3.23)	2.57(2.50,2.63)	1.81(1.74,1.88)	2.46(2.37,2.56)	111
Iraq	568(483-680)	77	2,057(1,767-2,419)	60	4.33(4.25,4.42)	4.46(4.32,4.61)	4.65(4.30,4.99)	4.51(4.33,4.69)	14
Jordan	118(98-144)	137	586(499-693)	108	3.65(3.53,3.78)	6.23(6.14,6.32)	7.16(6.68,7.64)	5.66(5.50,5.83)	4
Kuwait	77(64-95)	145	307(254-373)	134	1.14(0.93,1.34)	6.20(6.11,6.29)	7.30(7.11,7.48)	4.92(4.82,5.02)	10
Lebanon	115(98-135)	139	289(246-341)	135	2.53(2.42,2.65)	3.12(3.08,3.17)	4.17(3.84,4.51)	3.26(3.15,3.37)	71
Libya	131(109-157)	134	356(297-426)	126	3.48(3.35,3.60)	4.44(4.37,4.50)	2.72(2.64,2.81)	3.54(3.49,3.60)	48
Morocco	783(663-925)	66	1,717(1,475-2,013)	64	2.68(2.60,2.77)	2.70(2.65,2.75)	2.92(2.83,3.00)	2.77(2.72,2.82)	92

Palestine	63(53-75)	149	222(190-264)	146	4.68(4.62,4.75)	4.62(4.55,4.70)	4.04(4.00,4.09)	4.46(4.42,4.50)	15
Oman	57(48-69)	150	239(199-295)	144	4.21(3.82,4.60)	3.53(3.20,3.86)	7.21(6.33,8.09)	5.15(4.78,5.52)	8
Qatar	19(15-23)	170	185(151-233)	147	4.29(4.08,4.50)	12.66(12.37,12.95)	7.51(7.29,7.73)	8.32(8.16,8.48)	1
Saudi Arabia	488(404-593)	85	2,111(1,757-2,575)	58	4.60(4.50,4.70)	5.23(5.20,5.25)	5.74(5.61,5.87)	5.18(5.13,5.24)	7
Syrian Arab Republic	382(319-458)	96	741(628-871)	99	4.43(4.33,4.54)	4.20(3.58,4.82)	-1.76(-2.31,-1.21)	2.36(2.03,2.70)	115
Tunisia	287(243-341)	108	633(541-737)	103	3.08(2.89,3.27)	2.74(2.67,2.81)	2.45(2.40,2.50)	2.77(2.69,2.84)	91
Turkey	4,128(3,709-4,604)	21	10,701(9,656-11,831)	17	3.45(3.36,3.53)	3.86(3.79,3.93)	2.59(2.49,2.69)	3.33(3.28,3.39)	69
United Arab Emirates	70(58-87)	146	571(455-718)	109	7.08(7.02,7.14)	12.04(11.68,12.41)	4.06(3.93,4.19)	7.53(7.41,7.65)	2
Yemen	314(265-378)	107	1,019(865-1,212)	86	3.85(3.80,3.91)	4.44(4.42,4.46)	4.09(3.94,4.25)	4.15(4.10,4.20)	20
Afghanistan	355(307-416)	101	1,338(1,138-1,586)	77	4.59(3.49,5.71)	5.04(4.94,5.13)	5.04(4.94,5.13)	4.76(4.34,5.18)	12
Bangladesh	9,631(8,558-10,937)	12	30,926(27,483-34,943)	6	3.10(3.00,3.20)	4.63(4.57,4.70)	4.49(4.40,4.58)	4.10(4.04,4.15)	23
Bhutan	52(46-60)	153	139(124-158)	155	2.88(2.53,3.24)	4.17(4.11,4.23)	3.17(2.94,3.40)	3.46(3.32,3.59)	57
India	95,123(85,809-104,899)	2	222,904(201,191-246,448)	1	2.99(2.94,3.04)	3.00(2.98,3.02)	2.91(2.86,2.97)	2.97(2.94,3.00)	83
Nepal	1,794(1,592-2,020)	42	5,413(4,853-6,031)	34	3.71(3.62,3.79)	4.09(4.05,4.13)	3.80(3.71,3.88)	3.88(3.84,3.93)	31
Pakistan	14,547(13,314-15,947)	8	31,583(28,656-34,669)	5	1.94(1.88,2.01)	2.79(2.75,2.83)	3.46(3.39,3.53)	2.73(2.70,2.77)	96
Angola	618(547-709)	72	2,263(1,997-2,608)	52	3.74(3.46,4.02)	5.29(5.21,5.37)	4.51(4.33,4.70)	4.53(4.41,4.66)	13
Central African Republic	181(160-207)	122	375(332-426)	121	2.57(2.49,2.65)	3.10(3.07,3.14)	1.82(1.64,2.00)	2.54(2.47,2.60)	105
Congo	157(140-179)	128	503(440-576)	115	3.08(2.96,3.20)	5.19(4.92,5.47)	4.02(3.81,4.22)	4.08(3.96,4.20)	25
Democratic Republic of the Congo	2,173(1,916-2,484)	36	5,912(5,183-6,752)	30	2.81(2.69,2.93)	3.65(3.56,3.74)	3.99(3.87,4.11)	3.49(3.43,3.56)	55
Equatorial Guinea	23(21-26)	167	111(97-128)	157	4.53(4.30,4.75)	6.88(6.73,7.03)	4.80(4.51,5.09)	5.49(5.34,5.63)	5
Gabon	68(61-77)	147	184(163-211)	148	2.94(2.80,3.09)	3.80(3.70,3.90)	3.50(3.36,3.65)	3.44(3.35,3.52)	61
Burundi	402(359-451)	91	1,047(935-1,180)	85	1.87(1.50,2.24)	4.97(4.75,5.19)	3.30(3.14,3.47)	3.36(3.19,3.53)	66
Comoros	40(36-44)	158	106(95-118)	158	2.76(2.65,2.87)	4.06(3.98,4.14)	3.33(3.25,3.41)	3.41(3.35,3.47)	62
Djibouti	38(34-44)	159	180(159-204)	150	4.39(4.07,4.71)	6.24(6.20,6.28)	5.58(5.46,5.70)	5.46(5.35,5.57)	6
Eritrea	223(200-250)	116	743(661-840)	98	4.20(3.96,4.43)	4.86(3.93,5.80)	3.90(3.78,4.02)	4.30(4.00,4.60)	19
Ethiopia	4,084(3,666-4,532)	22	9,327(8,370-10,359)	19	2.57(2.44,2.70)	2.65(2.56,2.75)	3.31(3.13,3.49)	2.86(2.77,2.95)	88
Kenya	2,098(1,900-2,324)	39	5,880(5,272-6,551)	32	3.36(3.35,3.37)	3.57(3.56,3.59)	3.94(3.86,4.01)	3.62(3.60,3.64)	41
Madagascar	913(816-1,021)	61	2,609(2,330-2,961)	49	2.99(2.78,3.21)	4.28(4.01,4.55)	3.66(3.60,3.71)	3.65(3.53,3.76)	40
Malawi	797(713-895)	65	1,848(1,656-2,074)	62	1.32(1.19,1.45)	2.96(2.87,3.05)	4.37(4.21,4.54)	2.90(2.81,2.99)	85
Mauritius	64(55-76)	148	116(100-134)	156	3.02(2.91,3.14)	1.78(1.73,1.82)	1.59(1.55,1.62)	2.10(2.06,2.14)	122
Mozambique	1,165(1,046-1,302)	54	2,854(2,554-3,220)	47	2.81(2.63,2.99)	3.27(3.25,3.29)	3.31(2.97,3.66)	3.14(3.02,3.26)	78
Rwanda	554(495-619)	78	1,589(1,416-1,789)	69	0.38(-2.16,2.98)	5.32(4.97,5.66)	4.33(3.47,5.20)	3.46(2.58,4.34)	58
Seychelles	3(2-3)	194	5(4-6)	190	2.25(2.14,2.37)	2.46(2.39,2.54)	2.20(2.07,2.32)	2.31(2.24,2.38)	118
Somalia	775(683-870)	67	2,126(1,872-2,400)	57	3.40(3.23,3.56)	3.35(3.23,3.46)	3.92(3.80,4.03)	3.53(3.45,3.62)	50
United Republic of Tanzania	2,169(1,926-2,440)	37	6,169(5,481-6,963)	27	3.04(2.97,3.12)	3.88(3.83,3.94)	4.06(3.93,4.19)	3.67(3.62,3.73)	38
Uganda	1,201(1,073-1,349)	52	3,461(3,091-3,935)	42	2.29(1.94,2.65)	4.28(4.22,4.33)	4.50(4.17,4.84)	3.73(3.57,3.88)	36
Zambia	677(610-760)	69	2,179(1,960-2,438)	54	1.65(1.44,1.85)	4.57(4.38,4.75)	6.10(6.00,6.20)	4.11(4.00,4.22)	21
Botswana	114(101-130)	140	363(318-416)	124	4.59(4.40,4.79)	3.41(3.22,3.61)	4.00(3.73,4.27)	3.99(3.85,4.13)	29

Lesotho	171(152-192)	124	264(232-302)	139	1.48(1.34,1.63)	0.79(0.72,0.85)	2.27(2.20,2.35)	1.50(1.44,1.56)	147
Namibia	132(117-150)	133	343(305-387)	129	3.55(3.41,3.70)	3.03(2.87,3.19)	3.34(3.14,3.54)	3.33(3.22,3.43)	70
South Africa	6,276(5,652-6,962)	16	11,129(9,972-12,452)	16	2.57(2.45,2.68)	2.01(1.93,2.08)	1.22(1.05,1.40)	1.96(1.88,2.03)	130
Eswatini	78(69-89)	144	156(139-177)	151	3.54(3.10,3.98)	1.14(0.98,1.30)	2.74(2.61,2.86)	2.42(2.27,2.58)	113
Zimbabwe	519(450-606)	83	877(757-1,025)	94	1.66(1.53,1.79)	0.47(0.34,0.59)	3.56(3.44,3.67)	1.82(1.73,1.91)	138
Benin	113(98-131)	141	356(308-417)	125	3.80(3.78,3.83)	4.16(4.10,4.22)	4.21(4.16,4.26)	4.04(4.02,4.07)	27
Burkina Faso	230(201-265)	115	629(543-732)	104	2.87(2.81,2.93)	3.86(3.80,3.92)	3.84(3.74,3.94)	3.54(3.50,3.59)	49
Cameroon	282(243-328)	110	960(832-1,124)	88	3.87(3.78,3.97)	4.39(4.28,4.51)	4.74(4.63,4.85)	4.33(4.26,4.40)	17
Cabo Verde	8(7-9)	177	22(19-26)	176	3.26(3.19,3.34)	3.40(3.37,3.43)	3.84(3.79,3.89)	3.51(3.48,3.54)	52
Chad	144(125-166)	129	383(332-442)	120	2.65(2.63,2.68)	3.47(3.44,3.50)	4.30(4.22,4.37)	3.45(3.42,3.47)	59
C??te d'Ivoire	287(245-336)	109	816(699-959)	96	4.30(4.26,4.35)	3.28(3.24,3.32)	3.53(3.45,3.61)	3.66(3.63,3.70)	39
Gambia	23(19-27)	168	66(58-78)	160	4.28(4.24,4.31)	3.50(3.48,3.52)	3.59(3.56,3.63)	3.77(3.75,3.79)	35
Ghana	370(317-437)	99	1,152(988-1,355)	80	4.07(4.02,4.13)	3.65(3.60,3.70)	4.36(4.22,4.50)	4.01(3.95,4.06)	28
Guinea	170(148-194)	126	365(316-426)	123	2.95(2.83,3.07)	2.19(2.15,2.23)	2.90(2.76,3.04)	2.64(2.58,2.71)	99
Guinea-Bissau	24(21-28)	165	53(46-62)	167	2.21(2.20,2.23)	2.92(2.89,2.94)	3.31(3.28,3.35)	2.80(2.78,2.82)	90
Liberia	56(49-64)	152	155(133-184)	152	2.70(2.37,3.02)	4.29(3.94,4.64)	3.57(3.36,3.78)	3.52(3.35,3.70)	51
Mali	220(191-253)	118	611(528-712)	105	2.98(2.89,3.07)	3.77(3.67,3.87)	4.00(3.88,4.11)	3.59(3.52,3.66)	43
Mauritania	57(50-65)	151	151(132-174)	154	3.21(3.16,3.25)	3.32(3.27,3.36)	3.82(3.78,3.85)	3.44(3.41,3.46)	60
Niger	170(147-197)	125	547(474-636)	112	3.87(3.82,3.91)	4.35(4.31,4.40)	4.10(4.02,4.17)	4.11(4.08,4.14)	22
Nigeria	2,151(1,927-2,399)	38	5,884(5,262-6,601)	31	3.23(3.21,3.26)	3.16(3.14,3.19)	4.43(4.11,4.75)	3.56(3.46,3.66)	46
Sao Tome and Principe	3(3-4)	191	7(6-9)	189	1.82(1.78,1.86)	2.90(2.87,2.94)	4.18(4.12,4.24)	2.96(2.93,2.99)	84
Senegal	189(163-219)	120	492(429-573)	116	3.62(3.60,3.64)	3.19(3.14,3.24)	3.36(3.24,3.49)	3.38(3.33,3.42)	65
Sierra Leone	97(85-112)	142	242(209-284)	143	1.61(1.47,1.75)	3.73(3.51,3.94)	4.34(4.18,4.50)	3.26(3.14,3.37)	72
Togo	82(70-96)	143	250(213-293)	142	3.70(3.64,3.76)	3.89(3.81,3.96)	4.27(4.19,4.34)	3.94(3.90,3.99)	30
American Samoa	2(2-2)	197	3(2-3)	196	2.82(2.69,2.95)	-0.18(-0.33,-0.02)	0.22(0.12,0.33)	0.89(0.80,0.98)	178
Bermuda	6(5-7)	183	10(9-11)	183	2.24(2.14,2.35)	1.76(1.70,1.83)	0.66(0.60,0.72)	1.59(1.55,1.64)	143
Cook Islands	1(1-1)	199	1(1-1)	200	0.88(0.79,0.96)	0.65(0.37,0.93)	0.31(0.16,0.46)	0.57(0.46,0.68)	187
Greenland	5(5-6)	185	11(9-12)	181	2.80(2.77,2.83)	2.25(2.23,2.28)	1.89(1.72,2.05)	2.32(2.26,2.37)	117
Guam	6(5-8)	181	8(7-10)	188	1.91(1.79,2.02)	0.18(0.15,0.21)	0.63(0.55,0.71)	0.93(0.88,0.97)	174
Monaco	3(3-3)	192	4(4-5)	194	0.73(0.54,0.92)	1.32(1.24,1.41)	1.47(1.22,1.71)	1.17(1.05,1.29)	162
Nauru	0(0-0)	201	0(0-1)	202	0.85(0.78,0.93)	-0.33(-0.39,-0.26)	1.94(1.66,2.21)	0.74(0.65,0.83)	180
Niue	0(0-0)	203	0(0-0)	203	-0.32(-0.42,-0.21)	-1.29(-1.37,-1.21)	1.30(1.17,1.43)	-0.15(-0.22,-0.09)	199
Northern Mariana Islands	2(2-3)	195	2(2-3)	198	5.99(4.52,7.49)	-4.19(-4.63,-3.75)	-2.44(-3.14,-1.74)	-0.05(-0.63,0.53)	198
Palau	1(1-1)	200	1(1-1)	199	3.77(3.59,3.95)	-0.10(-0.24,0.05)	0.00(-0.18,0.17)	1.13(1.02,1.24)	167
Puerto Rico	391(344-459)	94	560(490-635)	111	2.21(2.17,2.25)	1.46(1.35,1.56)	0.09(-0.02,0.20)	1.26(1.20,1.31)	159
Saint Kitts and Nevis	4(3-4)	188	8(7-10)	187	3.42(3.23,3.61)	3.27(3.20,3.34)	1.77(1.65,1.89)	2.84(2.76,2.93)	89
San Marino	3(2-3)	193	5(4-6)	192	1.61(1.32,1.90)	2.12(2.01,2.23)	2.16(1.83,2.50)	1.95(1.79,2.12)	131
Tokelau	0(0-0)	204	0(0-0)	204	0.36(-0.03,0.74)	-1.90(-2.33,-1.46)	2.21(2.07,2.34)	0.34(0.13,0.56)	193

Tuvalu	0(0-0)	202	0(0-1)	201	0.90(0.86,0.95)	1.29(1.27,1.32)	2.00(1.95,2.06)	1.39(1.37,1.42)	156
United States Virgin Islands	9(7-10)	176	12(10-14)	179	1.92(1.88,1.96)	1.17(1.05,1.29)	0.41(0.31,0.51)	1.16(1.10,1.21)	165
South Sudan	484(432-550)	87	933(827-1,062)	91	1.67(1.55,1.79)	3.08(3.03,3.12)	2.02(1.68,2.36)	2.31(2.20,2.43)	119
Sudan	523(443-621)	81	1,542(1,317-1,829)	72	3.29(3.25,3.34)	3.46(3.38,3.54)	4.63(4.36,4.91)	3.77(3.68,3.87)	34
Psoriasis									
China	784,605(756,135-813,047)	1	892,906(859,645-924,276)	1	0.88(0.80,0.95)	0.47(0.46,0.48)	0.04(0.02,0.05)	0.46(0.43,0.48)	148
Democratic People's Republic of Korea	16,106(15,424-16,801)	37	18,993(18,187-19,767)	45	1.24(1.18,1.29)	0.52(0.49,0.54)	0.02(0.01,0.04)	0.57(0.55,0.59)	140
Taiwan (Province of China)	12,906(12,374-13,462)	46	14,068(13,426-14,653)	52	0.64(0.62,0.66)	0.30(0.27,0.34)	-0.11(-0.15,-0.07)	0.29(0.27,0.31)	155
Cambodia	2,655(2,546-2,768)	100	4,215(4,048-4,395)	98	1.87(1.84,1.89)	1.37(1.34,1.40)	1.56(1.49,1.62)	1.60(1.58,1.62)	72
Indonesia	23,160(22,248-24,050)	26	34,475(33,118-35,772)	22	1.59(1.54,1.64)	1.29(1.12,1.46)	1.32(1.02,1.62)	1.39(1.27,1.50)	86
Lao People's Democratic Republic	1,127(1,082-1,173)	134	1,812(1,740-1,885)	132	2.12(2.11,2.14)	1.54(1.50,1.57)	1.29(1.24,1.35)	1.65(1.63,1.67)	69
Malaysia	3,625(3,477-3,769)	87	6,234(5,979-6,493)	83	2.69(2.64,2.75)	1.70(1.55,1.85)	1.30(1.28,1.32)	1.89(1.84,1.95)	57
Maldives	51(49-53)	184	115(110-121)	176	1.90(1.86,1.95)	2.36(2.31,2.41)	4.49(4.45,4.52)	2.88(2.85,2.90)	11
Myanmar	11,465(11,016-11,948)	52	14,468(13,896-15,026)	51	1.18(1.12,1.24)	0.46(0.41,0.51)	0.72(0.69,0.75)	0.79(0.76,0.82)	126
Philippines	14,330(13,826-14,865)	42	25,748(24,810-26,703)	29	2.28(2.24,2.31)	2.52(2.50,2.54)	1.25(1.17,1.34)	2.05(2.02,2.08)	47
Sri Lanka	6,841(6,555-7,153)	66	8,196(7,838-8,565)	69	0.72(0.69,0.76)	0.82(0.81,0.84)	0.35(0.29,0.41)	0.63(0.60,0.65)	135
Thailand	13,679(13,113-14,293)	43	18,373(17,574-19,165)	47	1.08(1.04,1.13)	1.33(1.01,1.65)	0.63(0.57,0.69)	1.00(0.90,1.11)	110
Timor-Leste	199(191-207)	166	297(285-309)	166	1.65(1.59,1.71)	0.49(0.29,0.68)	1.85(1.79,1.91)	1.36(1.29,1.42)	88
Viet Nam	17,077(16,389-17,824)	34	24,566(23,509-25,523)	32	1.53(1.52,1.55)	1.18(1.17,1.20)	1.07(0.98,1.15)	1.27(1.24,1.29)	99
Fiji	263(252-276)	162	312(299-325)	163	0.60(0.57,0.64)	0.87(0.84,0.89)	0.20(0.17,0.23)	0.58(0.56,0.60)	138
Kiribati	25(24-26)	193	40(38-42)	189	1.39(1.32,1.46)	2.22(2.17,2.28)	1.22(1.17,1.28)	1.65(1.61,1.69)	68
Marshall Islands	15(14-16)	196	19(18-20)	196	1.30(1.28,1.32)	0.67(0.63,0.70)	0.57(0.53,0.62)	0.85(0.83,0.87)	121
Micronesia (Federated States of)	34(32-35)	190	34(32-35)	193	0.37(0.32,0.42)	-0.24(-0.25,-0.23)	-0.15(-0.21,-0.10)	-0.01(-0.03,0.02)	170
Papua New Guinea	1,555(1,490-1,622)	119	3,524(3,382-3,678)	107	2.67(2.60,2.74)	3.14(3.13,3.16)	2.70(2.68,2.73)	2.86(2.83,2.88)	12
Samoa	53(51-55)	183	70(68-73)	181	0.94(0.89,0.99)	0.92(0.89,0.95)	1.10(1.07,1.13)	0.99(0.97,1.01)	112
Solomon Islands	126(121-132)	169	231(222-241)	168	2.46(2.45,2.46)	2.24(2.22,2.26)	1.64(1.61,1.67)	2.12(2.11,2.13)	44
Tonga	33(32-35)	191	34(32-35)	192	0.14(0.08,0.20)	0.53(0.47,0.60)	-0.62(-0.70,-0.55)	0.03(-0.03,0.09)	168
Vanuatu	55(53-58)	181	103(98-108)	177	2.16(2.13,2.19)	2.48(2.44,2.51)	1.82(1.78,1.85)	2.16(2.14,2.19)	42
Armenia	2,462(2,351-2,567)	102	1,941(1,851-2,031)	129	-0.43(-0.49,-0.37)	-1.55(-1.62,-1.48)	-0.31(-0.36,-0.26)	-0.82(-0.85,-0.78)	191
Azerbaijan	4,896(4,675-5,113)	76	6,606(6,297-6,907)	80	1.39(1.36,1.43)	0.41(0.13,0.70)	1.31(1.26,1.36)	1.06(0.97,1.15)	107
Georgia	3,886(3,712-4,056)	82	2,377(2,268-2,484)	118	-1.42(-1.49,-1.34)	-2.36(-2.39,-2.33)	-1.19(-1.22,-1.16)	-1.68(-1.71,-1.65)	204
Kazakhstan	11,705(11,192-12,199)	51	11,534(11,012-12,029)	59	-1.23(-1.41,-1.05)	-0.08(-0.12,-0.03)	1.16(1.11,1.22)	-0.05(-0.11,0.01)	173
Kyrgyzstan	3,103(2,965-3,243)	95	4,183(3,997-4,370)	100	0.95(0.88,1.01)	0.73(0.68,0.78)	1.50(1.46,1.54)	1.04(1.01,1.08)	108
Mongolia	1,514(1,449-1,581)	121	2,210(2,113-2,302)	123	1.11(1.07,1.16)	1.00(0.95,1.05)	1.92(1.89,1.96)	1.32(1.30,1.35)	93
Tajikistan	3,674(3,515-3,838)	86	6,039(5,788-6,298)	87	1.62(1.53,1.70)	1.49(1.43,1.54)	2.00(1.97,2.03)	1.73(1.69,1.76)	63
Turkmenistan	2,412(2,309-2,517)	104	2,979(2,851-3,115)	109	1.11(1.03,1.20)	0.40(0.34,0.47)	0.74(0.68,0.80)	0.72(0.68,0.77)	130
Uzbekistan	14,527(13,902-15,160)	40	21,107(20,180-22,041)	39	1.60(1.57,1.64)	0.98(0.94,1.01)	1.38(1.35,1.40)	1.30(1.28,1.32)	96
Albania	2,358(2,265-2,450)	107	1,814(1,743-1,884)	131	0.05(-0.11,0.21)	-1.68(-1.84,-1.52)	-0.78(-0.89,-0.67)	-0.90(-1.03,-0.77)	195

Bosnia and Herzegovina	3,610(3,473-3,749)	88	2,269(2,174-2,358)	120	-1.72(-2.00,-1.43)	-1.63(-1.93,-1.34)	-1.55(-1.77,-1.32)	-1.62(-1.79,-1.46)	203
Bulgaria	6,684(6,419-6,938)	67	4,552(4,366-4,734)	96	-1.39(-1.47,-1.32)	-1.46(-1.55,-1.38)	-0.97(-1.02,-0.91)	-1.29(-1.34,-1.25)	200
Croatia	3,674(3,527-3,824)	85	2,744(2,635-2,853)	113	-1.09(-1.28,-0.90)	-1.38(-1.64,-1.12)	-0.50(-0.57,-0.43)	-0.98(-1.08,-0.88)	196
Czechia	7,673(7,378-7,957)	63	6,795(6,517-7,069)	77	-0.65(-0.73,-0.56)	-0.48(-0.73,-0.23)	-0.08(-0.14,-0.02)	-0.38(-0.47,-0.30)	187
Hungary	7,939(7,616-8,224)	62	6,317(6,055-6,548)	82	-0.87(-0.94,-0.79)	-0.95(-1.14,-0.76)	-0.48(-0.54,-0.41)	-0.75(-0.82,-0.68)	189
North Macedonia	1,468(1,413-1,519)	123	1,422(1,363-1,476)	138	0.18(-0.60,0.95)	-0.47(-0.63,-0.31)	0.09(-0.14,0.31)	-0.10(-0.35,0.16)	176
Montenegro	433(416-448)	154	392(377-408)	160	0.22(0.14,0.29)	-0.81(-0.90,-0.73)	-0.26(-0.32,-0.20)	-0.32(-0.37,-0.27)	185
Poland	29,206(28,137-30,311)	20	28,151(27,328-28,936)	25	-0.15(-0.19,-0.11)	-0.03(-0.16,0.10)	-0.18(-0.21,-0.14)	-0.12(-0.16,-0.08)	177
Romania	16,954(16,323-17,555)	35	12,055(11,615-12,533)	57	-0.79(-0.84,-0.75)	-1.65(-1.82,-1.48)	-0.91(-0.97,-0.84)	-1.14(-1.21,-1.08)	199
Serbia	7,233(6,955-7,503)	65	5,654(5,437-5,877)	90	-0.38(-0.77,0.01)	-1.37(-1.67,-1.08)	-0.65(-0.87,-0.43)	-0.84(-1.02,-0.66)	192
Slovakia	3,900(3,761-4,042)	81	3,526(3,388-3,661)	106	-0.35(-0.41,-0.29)	-0.61(-0.75,-0.46)	-0.05(-0.10,0.01)	-0.32(-0.37,-0.27)	186
Slovenia	1,412(1,358-1,466)	124	1,305(1,252-1,356)	142	-0.28(-0.29,-0.27)	-0.51(-0.55,-0.47)	0.03(-0.04,0.10)	-0.27(-0.30,-0.25)	183
Belarus	8,076(7,754-8,369)	60	6,510(6,251-6,781)	81	-0.48(-0.51,-0.45)	-1.36(-1.45,-1.28)	-0.29(-0.32,-0.25)	-0.74(-0.77,-0.71)	188
Estonia	1,175(1,129-1,220)	132	848(814-879)	147	-1.74(-1.82,-1.65)	-1.27(-1.39,-1.16)	-0.38(-0.46,-0.30)	-1.14(-1.20,-1.08)	198
Latvia	1,975(1,900-2,052)	112	1,246(1,195-1,297)	143	-1.43(-1.48,-1.38)	-1.97(-2.04,-1.91)	-1.22(-1.26,-1.18)	-1.58(-1.61,-1.54)	202
Lithuania	2,680(2,574-2,778)	99	1,791(1,717-1,863)	133	-0.79(-0.84,-0.74)	-1.96(-2.03,-1.88)	-1.31(-1.36,-1.26)	-1.39(-1.43,-1.35)	201
Republic of Moldova	3,244(3,125-3,362)	94	2,553(2,448-2,653)	116	-0.46(-0.48,-0.45)	-1.33(-1.39,-1.26)	-0.58(-0.61,-0.56)	-0.81(-0.84,-0.79)	190
Russian Federation	105,532(101,563-109,520)	9	96,948(93,468-100,400)	10	-0.40(-0.44,-0.35)	-0.68(-1.04,-0.32)	0.11(0.02,0.20)	-0.31(-0.43,-0.19)	184
Ukraine	38,843(37,424-40,324)	15	30,080(28,899-31,319)	24	-0.72(-0.75,-0.69)	-1.40(-1.65,-1.15)	-0.51(-0.56,-0.46)	-0.86(-0.94,-0.78)	194
Brunei Darussalam	89(86-93)	175	161(154-168)	174	2.68(2.65,2.71)	1.92(1.90,1.93)	1.53(1.50,1.56)	2.04(2.03,2.06)	49
Japan	55,544(53,578-57,514)	11	57,045(54,998-58,992)	15	0.48(0.46,0.50)	0.12(0.07,0.17)	-0.32(-0.36,-0.27)	0.10(0.08,0.12)	164
Republic of Korea	19,576(18,790-20,437)	29	24,621(23,555-25,646)	31	0.50(0.48,0.52)	0.71(0.66,0.75)	1.15(1.09,1.22)	0.80(0.77,0.83)	124
Singapore	1,126(1,080-1,174)	135	2,164(2,074-2,250)	125	2.98(2.76,3.21)	2.10(1.49,2.71)	1.69(1.39,2.00)	2.32(2.08,2.55)	34
Australia	26,593(25,522-27,670)	22	36,165(34,649-37,716)	20	1.26(1.22,1.30)	1.16(1.12,1.20)	0.74(0.68,0.79)	1.06(1.03,1.09)	106
New Zealand	5,904(5,695-6,104)	73	7,387(7,116-7,647)	71	0.97(0.92,1.03)	1.22(1.17,1.26)	0.24(0.21,0.27)	0.79(0.76,0.81)	125
Andorra	124(119-128)	170	193(186-201)	171	1.42(0.92,1.93)	2.52(1.27,3.79)	0.24(-1.00,1.50)	1.25(0.57,1.92)	100
Austria	18,745(18,076-19,420)	31	20,770(19,961-21,523)	41	0.20(0.17,0.23)	0.34(0.22,0.45)	0.55(0.50,0.60)	0.35(0.31,0.40)	152
Belgium	24,611(23,699-25,517)	25	26,452(25,453-27,481)	28	0.06(0.06,0.07)	0.38(0.36,0.39)	0.31(0.30,0.32)	0.25(0.25,0.26)	157
Cyprus	1,815(1,749-1,877)	116	2,924(2,811-3,032)	110	1.33(1.31,1.34)	1.85(1.80,1.89)	1.79(1.76,1.81)	1.66(1.64,1.67)	67
Denmark	12,448(11,992-12,905)	48	13,386(12,870-13,875)	55	0.27(0.25,0.29)	0.21(0.20,0.21)	0.29(0.29,0.29)	0.25(0.25,0.26)	156
Finland	13,102(12,649-13,559)	45	13,501(13,010-13,980)	54	0.26(0.25,0.26)	0.09(0.09,0.10)	-0.04(-0.05,-0.03)	0.10(0.10,0.11)	165
France	160,279(154,148-166,526)	5	172,843(166,210-179,240)	5	0.36(0.33,0.38)	0.43(0.38,0.47)	-0.04(-0.11,0.03)	0.25(0.22,0.28)	158
Germany	162,807(156,861-168,814)	4	167,515(161,352-173,618)	6	0.29(0.08,0.51)	-0.20(-0.29,-0.11)	0.09(-0.54,0.72)	0.04(-0.17,0.25)	167
Greece	25,418(24,479-26,360)	24	23,753(22,836-24,627)	34	0.43(0.37,0.49)	-0.22(-0.29,-0.16)	-0.91(-0.95,-0.87)	-0.24(-0.28,-0.21)	181
Iceland	630(607-652)	146	833(802-862)	148	0.91(0.88,0.94)	1.29(1.15,1.43)	0.78(0.68,0.88)	0.97(0.91,1.03)	114
Ireland	8,591(8,295-8,880)	57	11,346(10,944-11,771)	60	0.60(0.47,0.74)	1.66(1.62,1.70)	0.53(0.47,0.59)	0.98(0.93,1.03)	113
Israel	10,556(10,194-10,927)	53	19,158(18,527-19,854)	44	2.48(2.45,2.50)	2.04(2.02,2.05)	1.70(1.69,1.72)	2.07(2.06,2.09)	46
Italy	110,243(106,453-113,988)	8	109,807(105,647-114,073)	8	0.01(-0.06,0.08)	0.48(0.45,0.51)	-0.64(-0.80,-0.49)	-0.01(-0.07,0.04)	169

Luxembourg	902(870-934)	140	1,427(1,374-1,476)	137	1.10(1.06,1.14)	1.37(1.35,1.40)	2.36(2.34,2.38)	1.59(1.58,1.61)	73
Malta	891(860-924)	141	996(958-1,032)	145	0.68(0.66,0.70)	0.46(0.45,0.48)	0.01(-0.01,0.03)	0.39(0.38,0.40)	149
Netherlands	35,699(34,429-36,965)	19	39,506(37,991-41,047)	19	0.58(0.57,0.59)	0.33(0.32,0.33)	0.14(0.13,0.16)	0.35(0.34,0.36)	151
Norway	7,564(7,318-7,813)	64	9,355(9,038-9,670)	66	0.46(0.45,0.47)	0.74(0.72,0.77)	0.99(0.97,1.02)	0.74(0.72,0.75)	129
Portugal	25,800(24,865-26,716)	23	25,564(24,604-26,526)	30	0.17(0.14,0.21)	0.10(0.08,0.12)	-0.37(-0.39,-0.35)	-0.02(-0.04,-0.01)	171
Spain	90,544(86,844-94,095)	10	104,133(99,854-108,476)	9	0.33(0.29,0.38)	1.42(1.38,1.47)	-0.34(-0.45,-0.22)	0.50(0.44,0.55)	145
Sweden	16,171(15,582-16,725)	36	18,918(18,240-19,571)	46	0.33(0.29,0.36)	0.51(0.48,0.55)	0.78(0.76,0.79)	0.53(0.52,0.55)	142
Switzerland	15,449(14,891-15,997)	38	19,321(18,587-20,062)	42	0.56(0.54,0.58)	0.78(0.72,0.85)	0.94(0.88,1.00)	0.76(0.73,0.79)	128
United Kingdom	130,679(126,486-134,745)	7	144,237(139,636-148,769)	7	0.16(0.14,0.17)	0.45(0.43,0.47)	0.42(0.40,0.44)	0.35(0.34,0.36)	153
Argentina	36,166(34,666-37,684)	17	47,387(45,347-49,524)	17	0.72(0.69,0.74)	1.12(1.11,1.13)	0.98(0.96,1.00)	0.93(0.92,0.95)	117
Chile	14,419(13,782-15,057)	41	19,225(18,381-20,058)	43	1.19(1.16,1.22)	0.94(0.93,0.95)	0.87(0.78,0.96)	1.00(0.97,1.03)	109
Uruguay	3,432(3,291-3,578)	91	3,596(3,445-3,758)	104	0.26(0.23,0.29)	0.16(0.15,0.17)	0.06(0.05,0.08)	0.16(0.14,0.17)	160
Canada	27,254(26,311-28,143)	21	34,709(33,461-35,993)	21	0.82(0.78,0.86)	0.88(0.86,0.90)	0.82(0.78,0.85)	0.84(0.82,0.86)	122
United States of America	274,815(266,032-283,181)	3	324,492(313,901-334,530)	3	0.83(0.73,0.92)	0.25(0.14,0.36)	0.67(0.65,0.69)	0.58(0.53,0.63)	139
Antigua and Barbuda	32(31-34)	192	48(46-50)	188	2.30(2.19,2.41)	1.32(1.21,1.42)	0.57(0.53,0.60)	1.41(1.36,1.46)	84
Bahamas	131(126-137)	168	200(191-208)	170	2.07(2.03,2.12)	1.39(1.37,1.41)	0.99(0.94,1.05)	1.47(1.45,1.50)	79
Barbados	132(127-137)	167	161(154-168)	173	0.29(0.22,0.35)	1.03(1.00,1.05)	0.79(0.74,0.84)	0.71(0.68,0.74)	131
Belize	109(104-113)	173	225(216-235)	169	1.99(1.90,2.07)	2.95(2.93,2.97)	2.62(2.58,2.67)	2.55(2.52,2.58)	22
Cuba	6,293(6,037-6,577)	70	6,896(6,572-7,209)	76	0.96(0.92,0.99)	0.17(0.15,0.19)	-0.19(-0.22,-0.17)	0.32(0.30,0.33)	154
Dominica	40(38-41)	187	37(35-38)	191	-0.59(-0.60,-0.58)	-0.26(-0.27,-0.25)	0.03(0.01,0.04)	-0.27(-0.28,-0.27)	182
Dominican Republic	4,275(4,088-4,467)	78	6,164(5,902-6,431)	86	2.09(2.00,2.18)	0.67(0.60,0.75)	1.06(1.01,1.11)	1.28(1.23,1.33)	97
Grenada	48(46-50)	185	57(54-59)	184	1.30(1.21,1.39)	0.69(0.56,0.81)	-0.28(-0.31,-0.25)	0.59(0.54,0.64)	136
Guyana	410(392-429)	155	401(383-418)	159	-0.14(-0.17,-0.11)	-0.23(-0.26,-0.19)	0.24(0.14,0.34)	-0.05(-0.09,-0.01)	172
Haiti	4,175(4,005-4,349)	79	7,689(7,367-8,012)	70	2.19(2.18,2.21)	2.19(2.18,2.21)	2.06(1.94,2.18)	2.13(2.08,2.19)	43
Jamaica	1,307(1,253-1,362)	127	1,523(1,457-1,592)	135	0.88(0.79,0.98)	0.37(0.36,0.39)	0.30(0.28,0.31)	0.53(0.50,0.56)	143
Saint Lucia	75(72-78)	176	99(94-103)	178	1.02(1.01,1.04)	1.10(1.08,1.12)	0.78(0.76,0.81)	0.97(0.96,0.99)	115
Saint Vincent and the Grenadines	61(59-64)	179	64(61-67)	182	-0.15(-0.16,-0.13)	0.30(0.27,0.34)	0.34(0.31,0.36)	0.16(0.15,0.18)	161
Suriname	206(198-216)	165	303(291-316)	165	1.42(1.36,1.48)	1.77(1.73,1.80)	0.76(0.72,0.81)	1.34(1.31,1.37)	90
Trinidad and Tobago	609(584-636)	148	718(687-749)	154	0.78(0.75,0.80)	0.43(0.42,0.45)	0.49(0.45,0.53)	0.56(0.55,0.58)	141
Bolivia (Plurinational State of)	6,392(6,131-6,672)	69	10,806(10,336-11,272)	61	2.04(2.02,2.07)	1.75(1.70,1.79)	1.65(1.58,1.71)	1.83(1.80,1.86)	59
Ecuador	8,308(7,944-8,692)	58	13,354(12,779-13,967)	56	1.88(1.76,1.99)	1.59(1.52,1.66)	1.45(1.40,1.49)	1.64(1.60,1.69)	70
Peru	19,050(18,164-19,900)	30	27,942(26,655-29,131)	26	1.50(1.43,1.57)	1.13(1.11,1.16)	1.39(1.20,1.58)	1.33(1.26,1.39)	92
Colombia	6,574(6,312-6,837)	68	9,471(9,106-9,861)	65	2.00(1.92,2.07)	1.20(1.16,1.24)	0.70(0.62,0.78)	1.28(1.24,1.32)	98
Costa Rica	602(578-626)	149	938(899-974)	146	2.38(2.34,2.42)	1.36(1.33,1.38)	0.90(0.85,0.96)	1.53(1.51,1.56)	76
El Salvador	1,174(1,130-1,222)	133	1,313(1,260-1,369)	141	0.70(0.63,0.77)	0.08(0.07,0.10)	0.42(0.40,0.43)	0.39(0.37,0.41)	150
Guatemala	1,897(1,821-1,978)	115	3,742(3,588-3,906)	101	2.40(2.36,2.44)	2.50(2.49,2.52)	2.19(2.13,2.26)	2.37(2.35,2.40)	31
Honduras	1,088(1,042-1,136)	136	2,128(2,050-2,223)	127	2.27(2.22,2.32)	2.44(2.43,2.46)	2.33(2.29,2.38)	2.34(2.32,2.36)	33
Mexico	18,444(17,794-19,178)	32	26,783(25,781-27,818)	27	1.52(1.41,1.63)	1.34(1.31,1.36)	1.05(1.01,1.10)	1.31(1.27,1.35)	94

Nicaragua	864(831-902)	142	1,397(1,341-1,454)	139	2.06(2.02,2.10)	1.56(1.54,1.57)	1.42(1.36,1.48)	1.67(1.64,1.70)	66
Panama	469(451-488)	152	786(755-815)	150	1.89(1.75,2.04)	1.84(1.67,2.00)	1.72(1.69,1.76)	1.81(1.73,1.88)	60
Venezuela (Bolivarian Republic of)	3,731(3,581-3,878)	83	5,812(5,578-6,044)	88	2.00(1.84,2.15)	2.10(1.89,2.31)	0.33(0.10,0.57)	1.51(1.39,1.63)	77
Brazil	130,999(126,494-135,573)	6	196,399(189,359-202,875)	4	1.61(1.60,1.62)	1.50(1.48,1.52)	1.12(1.06,1.17)	1.41(1.39,1.43)	83
Paraguay	3,554(3,397-3,711)	90	6,172(5,902-6,455)	85	2.37(2.32,2.42)	1.92(1.91,1.94)	1.48(1.38,1.58)	1.92(1.89,1.96)	55
Algeria	15,261(14,634-15,902)	39	23,015(22,050-23,987)	37	1.58(1.55,1.61)	1.35(1.33,1.36)	1.36(1.31,1.40)	1.42(1.40,1.44)	81
Bahrain	298(284-312)	158	827(788-865)	149	1.95(1.88,2.03)	6.78(6.68,6.88)	1.85(1.59,2.11)	3.58(3.49,3.67)	4
Egypt	21,844(20,908-22,743)	27	32,864(31,645-34,097)	23	1.12(1.07,1.16)	1.77(1.75,1.80)	1.30(1.26,1.34)	1.42(1.40,1.45)	82
Iran (Islamic Republic of)	37,217(35,895-38,552)	16	48,309(46,547-50,098)	16	0.87(0.60,1.15)	0.85(0.39,1.31)	1.05(0.99,1.10)	0.94(0.77,1.11)	116
Iraq	12,168(11,639-12,696)	50	24,332(23,393-25,350)	33	3.94(3.06,4.82)	1.75(1.62,1.88)	1.75(1.62,1.88)	2.46(2.17,2.76)	27
Jordan	2,118(2,023-2,212)	108	5,801(5,570-6,036)	89	1.99(1.48,2.49)	3.78(3.67,3.89)	5.01(4.90,5.12)	3.56(3.37,3.74)	5
Kuwait	952(909-999)	139	2,264(2,169-2,365)	121	0.60(0.34,0.86)	3.95(3.85,4.05)	4.49(4.25,4.74)	3.03(2.90,3.16)	10
Lebanon	2,082(1,998-2,168)	110	2,785(2,678-2,900)	112	0.83(0.79,0.88)	0.44(0.40,0.47)	1.86(1.71,2.02)	1.00(0.95,1.05)	111
Libya	2,400(2,303-2,500)	105	3,566(3,415-3,723)	105	0.79(0.66,0.91)	1.74(1.68,1.79)	1.60(1.55,1.65)	1.40(1.34,1.45)	85
Morocco	17,474(16,774-18,249)	33	22,227(21,324-23,124)	38	1.26(1.20,1.32)	0.77(0.76,0.79)	0.50(0.48,0.53)	0.84(0.82,0.86)	123
Palestine	1,355(1,301-1,414)	125	2,587(2,487-2,694)	115	2.85(2.75,2.94)	2.47(2.37,2.56)	1.45(1.39,1.50)	2.26(2.20,2.31)	37
Oman	1,178(1,128-1,230)	131	2,234(2,120-2,343)	122	-0.12(-0.30,0.05)	1.14(0.40,1.89)	5.85(4.09,7.64)	2.22(1.61,2.85)	39
Qatar	263(250-276)	163	1,458(1,383-1,536)	136	1.97(1.85,2.10)	10.85(10.46,11.24)	5.34(5.17,5.51)	6.14(6.00,6.28)	1
Saudi Arabia	10,252(9,807-10,729)	54	18,070(17,237-18,853)	48	0.99(0.95,1.04)	2.13(1.89,2.37)	2.74(2.63,2.85)	1.95(1.87,2.04)	53
Syrian Arab Republic	8,111(7,754-8,447)	59	8,321(7,973-8,668)	67	1.86(1.76,1.97)	1.86(1.76,1.97)	-3.87(-4.63,-3.11)	0.05(-0.21,0.30)	166
Tunisia	5,403(5,168-5,628)	74	6,757(6,485-7,022)	79	1.05(1.02,1.08)	0.68(0.67,0.70)	0.60(0.56,0.64)	0.77(0.75,0.79)	127
Turkey	53,717(51,480-56,046)	12	65,172(62,562-67,837)	12	0.92(0.86,0.99)	0.47(0.44,0.50)	0.63(0.46,0.80)	0.66(0.60,0.72)	134
United Arab Emirates	1,046(997-1,101)	138	4,793(4,543-5,056)	95	4.61(4.55,4.67)	10.15(9.80,10.50)	1.85(1.54,2.17)	5.37(5.21,5.54)	2
Yemen	9,088(8,728-9,493)	55	16,968(16,269-17,688)	50	2.24(2.20,2.27)	1.95(1.93,1.97)	2.36(2.33,2.40)	2.17(2.15,2.19)	41
Afghanistan	8,719(8,373-9,083)	56	23,306(22,374-24,298)	36	5.63(4.76,6.50)	2.58(2.51,2.66)	2.58(2.51,2.66)	3.48(3.15,3.80)	6
Bangladesh	45,289(43,561-47,173)	14	62,114(59,617-64,412)	14	1.20(1.11,1.28)	1.29(1.25,1.33)	0.82(0.80,0.84)	1.10(1.07,1.14)	104
Bhutan	266(256-277)	161	308(297-321)	164	0.17(-0.36,0.70)	0.95(0.83,1.07)	0.34(-0.02,0.70)	0.53(0.30,0.76)	144
India	388,949(375,495-403,587)	2	635,346(613,080-657,984)	2	2.21(2.19,2.23)	1.75(1.72,1.77)	1.13(1.08,1.17)	1.70(1.68,1.72)	64
Nepal	7,975(7,691-8,282)	61	10,310(9,936-10,714)	62	1.43(1.36,1.50)	0.66(0.59,0.72)	0.70(0.60,0.81)	0.90(0.85,0.95)	119
Pakistan	48,627(46,956-50,473)	13	84,769(81,758-88,026)	11	1.77(1.74,1.80)	2.04(2.03,2.05)	2.01(1.98,2.03)	1.93(1.92,1.95)	54
Angola	6,150(5,882-6,453)	71	13,975(13,363-14,615)	53	2.85(2.82,2.87)	3.24(3.18,3.29)	2.50(2.46,2.53)	2.86(2.84,2.88)	13
Central African Republic	1,642(1,572-1,720)	118	2,811(2,690-2,945)	111	2.31(2.28,2.35)	2.02(1.97,2.07)	1.21(1.06,1.35)	1.86(1.81,1.91)	58
Congo	1,201(1,147-1,260)	129	2,170(2,073-2,270)	124	2.04(2.02,2.07)	2.28(2.23,2.32)	1.84(1.81,1.88)	2.05(2.03,2.07)	48
Democratic Republic of the Congo	21,175(20,227-22,144)	28	41,824(39,992-43,691)	18	2.72(2.66,2.78)	2.78(2.69,2.86)	1.54(1.50,1.59)	2.37(2.33,2.41)	30
Equatorial Guinea	255(244-267)	164	520(495-546)	158	2.59(2.55,2.63)	2.36(2.35,2.38)	2.50(2.43,2.57)	2.47(2.44,2.50)	26
Gabon	483(461-505)	150	684(654-715)	155	1.39(1.37,1.41)	1.28(1.26,1.30)	0.91(0.75,1.07)	1.20(1.14,1.25)	103
Burundi	1,286(1,234-1,343)	128	2,716(2,610-2,830)	114	1.18(0.94,1.42)	3.80(3.45,4.15)	3.00(2.75,3.25)	2.61(2.46,2.77)	19
Comoros	106(102-111)	174	165(159-172)	172	1.59(1.52,1.67)	1.61(1.55,1.67)	1.39(1.37,1.42)	1.53(1.49,1.56)	75

Djibouti	110(105-114)	171	270(259-281)	167	2.88(2.41,3.35)	3.45(3.34,3.56)	3.06(2.82,3.29)	3.16(2.99,3.34)	8
Eritrea	745(715-777)	143	1,546(1,485-1,614)	134	2.67(1.52,3.84)	3.25(2.86,3.64)	1.92(1.74,2.10)	2.63(2.24,3.02)	17
Ethiopia	12,471(12,006-12,969)	47	23,712(22,854-24,668)	35	2.50(2.32,2.67)	2.11(2.09,2.13)	2.11(2.09,2.13)	2.23(2.17,2.28)	38
Kenya	4,691(4,528-4,881)	77	9,999(9,644-10,397)	63	2.62(2.57,2.67)	2.90(2.80,2.99)	2.36(2.29,2.44)	2.62(2.58,2.67)	18
Madagascar	2,897(2,780-3,021)	97	6,225(5,988-6,484)	84	2.64(2.59,2.70)	2.88(2.85,2.91)	2.46(2.42,2.50)	2.67(2.64,2.69)	16
Malawi	2,363(2,271-2,465)	106	4,189(4,019-4,357)	99	1.12(0.69,1.54)	2.14(2.08,2.19)	2.65(2.55,2.75)	1.98(1.84,2.12)	51
Mauritius	272(261-282)	160	331(316-345)	162	1.07(1.01,1.12)	0.64(0.60,0.68)	0.31(0.29,0.34)	0.68(0.65,0.70)	133
Mozambique	3,594(3,451-3,737)	89	6,996(6,719-7,291)	74	2.49(2.04,2.94)	2.25(2.22,2.28)	2.25(2.22,2.28)	2.32(2.18,2.46)	35
Rwanda	1,470(1,380-1,560)	122	2,484(2,349-2,629)	117	0.12(-2.38,2.68)	2.32(2.15,2.50)	2.32(2.15,2.50)	1.63(0.83,2.44)	71
Seychelles	16(15-16)	195	23(22-24)	195	1.05(0.98,1.11)	1.64(1.63,1.66)	1.29(1.26,1.32)	1.35(1.32,1.37)	89
Somalia	1,920(1,844-2,002)	113	5,394(5,184-5,628)	91	3.76(3.63,3.88)	3.71(3.53,3.89)	3.59(3.46,3.72)	3.67(3.57,3.77)	3
United Republic of Tanzania	5,932(5,573-6,298)	72	11,920(11,240-12,660)	58	2.45(2.36,2.54)	2.52(2.48,2.57)	2.28(2.26,2.30)	2.42(2.39,2.46)	28
Uganda	4,000(3,838-4,179)	80	8,265(7,941-8,613)	68	2.95(2.89,3.01)	2.37(2.34,2.40)	2.32(2.22,2.43)	2.53(2.49,2.58)	23
Zambia	1,774(1,697-1,851)	117	3,722(3,573-3,876)	102	1.87(1.79,1.94)	3.01(2.95,3.08)	2.91(2.89,2.93)	2.57(2.54,2.61)	20
Botswana	436(419-455)	153	724(694-757)	153	2.02(1.81,2.23)	1.33(1.30,1.36)	1.97(1.92,2.01)	1.76(1.70,1.83)	62
Lesotho	709(681-738)	144	745(715-777)	151	0.24(0.13,0.36)	-0.29(-0.33,-0.24)	0.50(0.46,0.53)	0.15(0.10,0.19)	162
Namibia	470(450-491)	151	728(697-759)	152	2.16(2.09,2.23)	1.21(1.15,1.27)	1.18(1.13,1.23)	1.50(1.46,1.54)	78
South Africa	12,278(11,850-12,765)	49	17,912(17,255-18,579)	49	1.91(1.84,1.97)	1.01(0.94,1.09)	1.05(1.00,1.09)	1.30(1.26,1.34)	95
Eswatini	277(265-291)	159	362(347-378)	161	1.78(1.70,1.85)	0.37(0.31,0.43)	0.60(0.55,0.66)	0.89(0.85,0.93)	120
Zimbabwe	3,427(3,274-3,584)	92	4,886(4,664-5,107)	93	1.04(0.93,1.15)	1.27(1.25,1.30)	1.38(1.36,1.41)	1.21(1.17,1.25)	102
Benin	1,515(1,455-1,578)	120	3,413(3,275-3,550)	108	2.36(2.08,2.64)	3.23(2.91,3.56)	2.88(2.82,2.95)	2.79(2.65,2.94)	15
Burkina Faso	3,261(3,135-3,394)	93	6,759(6,493-7,029)	78	1.63(1.51,1.74)	2.88(2.36,3.39)	3.01(2.89,3.12)	2.49(2.32,2.66)	24
Cameroon	2,997(2,887-3,121)	96	7,196(6,922-7,493)	73	2.81(2.72,2.91)	3.58(3.37,3.79)	2.80(2.74,2.85)	3.04(2.97,3.12)	9
Cabo Verde	110(105-114)	172	158(153-165)	175	1.46(1.14,1.77)	1.30(0.73,1.87)	1.05(0.97,1.12)	1.24(1.03,1.45)	101
Chad	2,112(2,031-2,198)	109	4,844(4,645-5,043)	94	2.29(2.17,2.41)	3.15(2.81,3.49)	3.19(3.04,3.34)	2.84(2.70,2.98)	14
C??te d'Ivoire	3,680(3,539-3,840)	84	6,979(6,708-7,254)	75	2.53(2.28,2.78)	2.31(2.19,2.44)	1.73(1.68,1.78)	2.19(2.10,2.29)	40
Gambia	319(307-333)	157	620(596-646)	156	2.31(2.04,2.58)	2.41(2.10,2.73)	2.18(2.12,2.24)	2.26(2.12,2.40)	36
Ghana	5,224(4,972-5,513)	75	9,664(9,184-10,160)	64	1.82(1.41,2.23)	2.42(2.27,2.57)	2.02(1.96,2.08)	2.10(1.96,2.25)	45
Guinea	2,069(1,987-2,149)	111	3,598(3,463-3,747)	103	1.77(1.50,2.03)	1.76(1.62,1.90)	2.32(2.18,2.46)	1.89(1.78,2.01)	56
Guinea-Bissau	331(318-344)	156	542(521-564)	157	1.18(0.76,1.60)	1.90(1.85,1.95)	1.90(1.85,1.95)	1.68(1.54,1.81)	65
Liberia	619(596-644)	147	1,334(1,284-1,386)	140	3.08(1.30,4.90)	2.73(2.48,2.98)	1.89(1.38,2.40)	2.57(1.99,3.17)	21
Mali	2,625(2,481-2,771)	101	5,376(5,061-5,695)	92	1.65(1.55,1.74)	2.92(2.69,3.14)	3.00(2.88,3.13)	2.49(2.38,2.59)	25
Mauritania	648(623-674)	145	1,089(1,047-1,133)	144	1.35(1.04,1.66)	2.19(2.04,2.35)	1.68(1.62,1.74)	1.77(1.65,1.88)	61
Niger	2,860(2,744-2,975)	98	7,343(7,053-7,658)	72	2.59(2.23,2.96)	3.71(3.57,3.84)	3.40(3.35,3.46)	3.26(3.14,3.39)	7
Nigeria	35,937(34,687-37,281)	18	64,049(61,790-66,559)	13	2.51(2.49,2.54)	2.07(2.02,2.12)	1.47(1.39,1.55)	2.00(1.97,2.04)	50
Sao Tome and Principe	35(33-36)	189	51(49-53)	187	0.65(0.30,1.00)	1.65(1.61,1.70)	1.65(1.61,1.70)	1.34(1.23,1.46)	91
Senegal	2,430(2,332-2,531)	103	4,312(4,149-4,483)	97	1.88(1.59,2.16)	2.23(2.08,2.37)	1.74(1.68,1.79)	1.96(1.86,2.07)	52
Sierra Leone	1,194(1,150-1,239)	130	2,344(2,249-2,439)	119	0.96(0.75,1.17)	3.47(3.24,3.69)	2.45(2.35,2.55)	2.38(2.24,2.51)	29

Togo	1,086(1,043-1,134)	137	2,152(2,071-2,241)	126	2.20(2.02,2.39)	2.82(2.65,2.99)	2.15(2.10,2.20)	2.36(2.27,2.44)	32
American Samoa	15(14-16)	197	17(17-18)	197	2.10(2.01,2.18)	-0.02(-0.10,0.05)	-0.48(-0.67,-0.29)	0.49(0.42,0.56)	147
Bermuda	35(34-37)	188	37(36-39)	190	0.65(0.61,0.68)	0.19(0.16,0.22)	-0.26(-0.30,-0.23)	0.19(0.17,0.21)	159
Cook Islands	6(6-6)	199	6(6-6)	200	0.06(0.02,0.11)	0.17(0.11,0.23)	-0.44(-0.50,-0.38)	-0.07(-0.11,-0.04)	174
Greenland	66(64-69)	178	64(61-66)	183	0.00(-0.09,0.09)	-0.38(-0.42,-0.33)	-0.05(-0.12,0.02)	-0.15(-0.19,-0.11)	179
Guam	42(40-44)	186	51(49-54)	186	1.49(1.30,1.68)	0.44(0.42,0.47)	0.21(0.16,0.25)	0.69(0.63,0.75)	132
Monaco	70(67-72)	177	83(80-86)	179	0.64(0.62,0.67)	0.67(0.66,0.68)	0.42(0.40,0.44)	0.58(0.57,0.59)	137
Nauru	3(3-3)	202	3(3-3)	202	0.56(0.51,0.60)	-0.27(-0.40,-0.14)	-0.57(-0.70,-0.44)	-0.10(-0.16,-0.04)	175
Niue	1(1-1)	203	1(1-1)	203	-1.35(-1.41,-1.28)	-2.19(-2.38,-2.00)	0.22(0.18,0.26)	-1.13(-1.20,-1.07)	197
Northern Mariana Islands	14(14-15)	198	15(15-16)	198	3.92(3.05,4.80)	-2.04(-2.18,-1.90)	-2.04(-2.18,-1.90)	0.14(-0.17,0.45)	163
Palau	5(5-5)	200	6(6-7)	199	3.17(3.13,3.22)	-0.46(-0.58,-0.33)	0.14(0.08,0.20)	0.93(0.88,0.98)	118
Puerto Rico	1,904(1,824-1,984)	114	1,830(1,751-1,908)	130	0.68(0.63,0.74)	-0.23(-0.28,-0.17)	-0.88(-0.96,-0.79)	-0.15(-0.19,-0.11)	178
Saint Kitts and Nevis	22(21-23)	194	32(31-34)	194	0.93(0.79,1.08)	1.80(1.78,1.83)	1.36(1.30,1.42)	1.39(1.34,1.45)	87
San Marino	54(52-55)	182	73(70-76)	180	1.32(1.29,1.34)	1.19(1.18,1.20)	0.76(0.74,0.78)	1.09(1.08,1.10)	105
Tokelau	1(1-1)	204	0(0-0)	204	-1.11(-1.36,-0.86)	-2.69(-2.93,-2.45)	1.02(0.91,1.12)	-0.85(-0.98,-0.72)	193
Tuvalu	3(3-4)	201	4(4-4)	201	-0.10(-0.15,-0.05)	0.76(0.73,0.79)	0.82(0.78,0.86)	0.50(0.47,0.52)	146
United States Virgin Islands	57(55-59)	180	54(51-56)	185	0.43(0.42,0.45)	-0.40(-0.44,-0.36)	-0.61(-0.64,-0.58)	-0.19(-0.21,-0.17)	180
South Sudan	1,335(1,281-1,400)	126	2,020(1,941-2,103)	128	1.82(1.71,1.94)	2.47(2.37,2.57)	-0.12(-0.49,0.25)	1.44(1.31,1.57)	80
Sudan	13,214(12,688-13,747)	44	20,787(19,974-21,670)	40	2.00(1.77,2.23)	1.26(1.22,1.30)	1.55(1.43,1.67)	1.58(1.50,1.66)	74
Atopic dermatitis									
China	4,805,479(4,598,356-4,998,115)	1	5,837,355(5,576,377-6,077,542)	1	0.61(0.59,0.63)	0.64(0.63,0.64)	0.78(0.77,0.80)	0.67(0.66,0.68)	107
Democratic People's Republic of Korea	99,857(93,265-106,506)	30	118,601(110,586-126,671)	32	1.23(1.17,1.29)	0.26(0.25,0.27)	0.37(0.36,0.38)	0.59(0.57,0.61)	115
Taiwan (Province of China)	103,478(95,736-110,985)	27	124,411(115,945-134,657)	31	0.85(0.81,0.88)	0.53(0.44,0.62)	0.53(0.44,0.61)	0.64(0.60,0.69)	111
Cambodia	55,975(52,132-59,935)	44	84,983(79,381-91,030)	42	1.55(1.51,1.59)	1.38(1.36,1.39)	1.44(1.41,1.47)	1.45(1.43,1.47)	68
Indonesia	1,052,089(1,004,552-1,099,115)	3	1,437,343(1,371,678-1,499,105)	3	1.36(1.35,1.37)	1.18(1.17,1.20)	0.70(0.67,0.73)	1.08(1.07,1.09)	80
Lao People's Democratic Republic	21,938(20,454-23,499)	84	36,529(34,100-39,204)	77	2.46(2.42,2.50)	1.55(1.52,1.57)	1.32(1.31,1.33)	1.78(1.76,1.79)	59
Malaysia	91,240(85,163-97,712)	32	155,471(145,241-166,594)	27	2.89(2.86,2.92)	1.57(1.54,1.60)	1.15(1.12,1.18)	1.85(1.83,1.88)	52
Maldives	1,182(1,101-1,267)	164	2,276(2,117-2,447)	159	2.07(1.99,2.15)	2.08(2.04,2.12)	2.69(2.60,2.79)	2.27(2.23,2.32)	35
Myanmar	213,749(199,507-228,977)	18	279,249(260,906-298,835)	16	1.10(1.04,1.15)	0.87(0.86,0.88)	0.83(0.82,0.83)	0.93(0.91,0.95)	87
Philippines	333,115(317,751-348,439)	11	577,429(552,006-601,063)	7	2.27(2.25,2.28)	1.83(1.83,1.84)	1.65(1.64,1.67)	1.92(1.91,1.92)	49
Sri Lanka	80,620(74,875-86,134)	34	100,917(93,817-108,104)	35	0.83(0.82,0.85)	0.92(0.90,0.95)	0.58(0.55,0.61)	0.78(0.77,0.79)	99
Thailand	288,447(268,696-309,920)	12	349,286(326,139-375,377)	12	0.89(0.73,1.06)	0.81(0.31,1.30)	0.32(0.23,0.41)	0.65(0.48,0.82)	109
Timor-Leste	4,102(3,813-4,387)	141	6,871(6,404-7,374)	135	1.51(1.47,1.54)	1.82(1.78,1.87)	2.08(2.07,2.09)	1.79(1.77,1.81)	57
Viet Nam	356,466(333,297-382,099)	10	482,241(449,509-517,448)	9	1.27(1.23,1.31)	1.12(1.10,1.14)	0.75(0.72,0.79)	1.05(1.03,1.07)	81
Fiji	3,735(3,465-4,003)	145	4,401(4,093-4,711)	146	0.49(0.46,0.52)	0.74(0.73,0.75)	0.45(0.43,0.48)	0.57(0.55,0.58)	116
Kiribati	377(350-404)	182	590(548-631)	179	1.37(1.32,1.41)	1.88(1.81,1.94)	1.34(1.30,1.38)	1.55(1.51,1.58)	65
Marshall Islands	232(214-250)	189	274(254-294)	185	1.04(1.00,1.08)	0.49(0.47,0.50)	0.24(0.23,0.25)	0.57(0.56,0.59)	117
Micronesia (Federated States of)	525(485-565)	179	489(455-524)	182	0.36(0.32,0.41)	-0.61(-0.62,-0.59)	-0.50(-0.53,-0.47)	-0.26(-0.28,-0.24)	167

Papua New Guinea	20,439(18,937-21,916)	89	49,151(45,580-52,645)	67	3.17(3.15,3.18)	3.03(3.00,3.06)	3.03(3.00,3.06)	3.08(3.07,3.09)	12
Samoa	798(739-857)	172	1,015(945-1,088)	174	0.45(0.43,0.48)	0.61(0.60,0.63)	1.46(1.45,1.48)	0.83(0.82,0.84)	94
Solomon Islands	1,733(1,607-1,867)	159	3,272(3,037-3,508)	152	2.81(2.77,2.85)	2.14(2.11,2.16)	1.69(1.66,1.71)	2.21(2.19,2.23)	39
Tonga	483(448-518)	180	508(472-542)	180	0.40(0.38,0.42)	0.54(0.52,0.57)	-0.47(-0.49,-0.44)	0.18(0.16,0.19)	146
Vanuatu	773(715-832)	173	1,462(1,358-1,565)	168	2.32(2.31,2.33)	2.40(2.40,2.41)	1.91(1.89,1.93)	2.22(2.22,2.23)	38
Armenia	22,421(19,657-25,480)	82	14,960(13,353-16,798)	114	-2.65(-2.94,-2.35)	-1.31(-1.39,-1.24)	-0.16(-0.43,0.11)	-1.39(-1.52,-1.25)	198
Azerbaijan	51,261(44,771-58,478)	48	53,286(47,396-59,856)	62	-0.35(-0.54,-0.16)	-0.03(-0.10,0.04)	0.77(0.60,0.95)	0.13(0.04,0.22)	150
Georgia	31,314(27,792-35,249)	65	17,994(15,983-20,190)	107	-3.49(-3.58,-3.40)	-2.07(-2.35,-1.80)	-0.31(-0.60,-0.03)	-1.94(-2.08,-1.81)	204
Kazakhstan	109,568(96,164-124,607)	26	113,437(100,091-128,506)	33	-2.87(-2.98,-2.76)	1.06(0.72,1.40)	2.14(1.90,2.39)	0.08(-0.06,0.22)	153
Kyrgyzstan	34,348(29,987-39,413)	59	44,146(38,786-50,380)	69	-0.17(-0.40,0.06)	0.58(0.51,0.64)	2.18(1.98,2.38)	0.88(0.78,0.98)	91
Mongolia	17,715(15,417-20,381)	96	22,556(19,790-25,698)	98	-1.27(-1.42,-1.11)	0.36(-0.30,1.02)	2.99(2.39,3.59)	0.72(0.43,1.01)	105
Tajikistan	46,994(40,746-54,189)	52	67,192(58,784-76,699)	51	0.63(0.49,0.76)	0.93(0.90,0.97)	2.14(2.01,2.27)	1.24(1.17,1.30)	73
Turkmenistan	30,290(26,325-34,836)	66	32,511(28,612-37,012)	82	-0.37(-0.63,-0.12)	-0.17(-0.55,0.22)	1.44(1.33,1.55)	0.27(0.12,0.43)	145
Uzbekistan	174,342(151,337-200,571)	22	216,921(190,780-246,369)	20	0.44(0.32,0.56)	0.39(0.34,0.45)	1.41(1.28,1.54)	0.75(0.68,0.81)	103
Albania	7,313(6,733-7,932)	128	4,510(4,239-4,790)	144	-0.81(-1.04,-0.58)	-2.98(-3.19,-2.77)	-0.74(-0.87,-0.62)	-1.68(-1.86,-1.51)	202
Bosnia and Herzegovina	8,414(7,850-8,994)	123	5,088(4,808-5,374)	140	-2.21(-2.92,-1.50)	-1.41(-1.70,-1.13)	-1.75(-1.83,-1.66)	-1.76(-2.01,-1.51)	203
Bulgaria	14,882(13,992-15,798)	104	10,679(10,110-11,271)	124	-2.40(-2.57,-2.24)	-0.34(-0.40,-0.29)	-0.87(-0.93,-0.81)	-1.15(-1.21,-1.09)	193
Croatia	8,337(7,821-8,853)	124	6,492(6,138-6,847)	137	-1.13(-1.22,-1.04)	-0.75(-0.92,-0.58)	-0.68(-0.92,-0.44)	-0.83(-0.96,-0.70)	189
Czechia	17,694(16,616-18,786)	97	17,109(16,131-18,109)	108	-1.42(-1.62,-1.22)	0.70(0.16,1.23)	0.06(-0.17,0.30)	-0.21(-0.41,0.00)	166
Hungary	21,397(19,941-23,032)	86	18,187(17,069-19,401)	106	-0.74(-0.94,-0.53)	-0.29(-0.44,-0.14)	-0.61(-0.71,-0.51)	-0.58(-0.67,-0.48)	182
North Macedonia	3,895(3,628-4,178)	143	3,428(3,226-3,631)	151	-0.60(-1.32,0.12)	-0.45(-0.66,-0.23)	-0.05(-0.11,0.01)	-0.47(-0.76,-0.18)	177
Montenegro	1,197(1,116-1,284)	163	1,017(958-1,078)	173	-0.62(-0.64,-0.61)	-0.58(-0.60,-0.56)	-0.50(-0.54,-0.47)	-0.57(-0.58,-0.55)	180
Poland	70,764(66,965-74,602)	37	56,802(54,785-58,845)	59	-1.37(-1.42,-1.33)	-0.91(-1.01,-0.81)	-0.11(-0.20,-0.03)	-0.78(-0.84,-0.73)	187
Romania	32,524(30,420-34,546)	62	23,037(21,867-24,190)	96	-1.98(-2.17,-1.79)	-0.80(-1.05,-0.55)	-1.04(-1.18,-0.91)	-1.24(-1.36,-1.11)	197
Serbia	16,737(15,675-17,824)	100	14,080(13,292-14,908)	116	-0.66(-1.38,0.06)	-0.60(-0.65,-0.55)	-0.60(-0.65,-0.55)	-0.62(-0.85,-0.39)	183
Slovakia	9,801(9,157-10,462)	118	8,659(8,162-9,158)	131	-1.18(-1.27,-1.10)	-0.31(-0.43,-0.19)	0.02(-0.15,0.19)	-0.49(-0.57,-0.40)	178
Slovenia	3,375(3,163-3,585)	149	3,246(3,066-3,429)	153	-1.00(-1.05,-0.94)	0.22(0.16,0.28)	0.27(0.20,0.34)	-0.13(-0.17,-0.09)	161
Belarus	19,542(18,052-21,103)	91	15,807(14,703-16,901)	112	-2.07(-2.16,-1.98)	-0.56(-0.72,-0.41)	0.34(0.09,0.59)	-0.77(-0.87,-0.67)	186
Estonia	4,417(4,035-4,819)	139	3,225(2,977-3,467)	154	-3.47(-3.74,-3.21)	0.01(-0.32,0.34)	-0.35(-0.60,-0.10)	-1.20(-1.39,-1.02)	195
Latvia	3,435(3,161-3,737)	148	2,161(2,023-2,318)	161	-2.97(-3.25,-2.68)	-1.01(-1.08,-0.95)	-0.60(-0.76,-0.45)	-1.59(-1.70,-1.48)	200
Lithuania	6,803(6,290-7,347)	130	4,419(4,136-4,701)	145	-1.58(-1.74,-1.43)	-1.88(-2.04,-1.72)	-0.93(-0.97,-0.89)	-1.47(-1.55,-1.40)	199
Republic of Moldova	9,099(8,355-9,899)	121	5,684(5,329-6,060)	139	-2.63(-2.70,-2.56)	-1.30(-1.50,-1.10)	-1.01(-1.12,-0.89)	-1.65(-1.73,-1.57)	201
Russian Federation	385,802(361,309-411,043)	9	344,268(325,625-362,601)	13	-2.20(-2.41,-1.99)	0.09(-0.01,0.19)	0.68(0.36,0.99)	-0.42(-0.55,-0.29)	173
Ukraine	100,488(94,500-106,516)	29	72,197(68,609-75,746)	48	-2.32(-2.38,-2.26)	-0.65(-0.82,-0.49)	-0.48(-0.58,-0.38)	-1.15(-1.22,-1.08)	194
Brunei Darussalam	1,400(1,282-1,528)	161	1,742(1,613-1,879)	166	1.68(1.62,1.74)	0.18(0.08,0.27)	0.58(0.53,0.63)	0.77(0.73,0.82)	102
Japan	566,304(537,627-595,411)	5	542,674(516,730-565,971)	8	-0.05(-0.11,0.01)	-0.03(-0.06,-0.01)	-0.38(-0.42,-0.34)	-0.14(-0.17,-0.11)	163
Republic of Korea	184,102(169,867-199,186)	21	183,563(171,269-196,193)	23	0.19(0.09,0.29)	-0.86(-1.00,-0.72)	0.68(0.64,0.73)	0.00(-0.06,0.07)	156
Singapore	10,867(9,938-11,819)	116	18,806(17,364-20,240)	104	2.90(2.63,3.18)	0.90(0.76,1.05)	1.99(1.61,2.37)	1.90(1.73,2.07)	51

Australia	51,558(47,536-55,973)	47	71,104(66,026-76,547)	49	0.92(0.86,0.98)	1.19(1.15,1.22)	1.24(1.19,1.28)	1.12(1.09,1.15)	76
New Zealand	13,512(12,749-14,298)	109	16,847(15,972-17,746)	109	0.84(0.69,0.99)	1.09(1.02,1.15)	0.33(0.26,0.40)	0.75(0.69,0.81)	104
Andorra	182(170-195)	192	258(243-273)	188	2.21(1.49,2.93)	2.15(1.50,2.80)	-0.72(-0.91,-0.53)	1.11(0.79,1.43)	77
Austria	28,356(26,545-30,172)	67	31,336(29,461-33,242)	83	0.18(0.12,0.23)	0.08(0.06,0.11)	0.89(0.85,0.92)	0.35(0.32,0.37)	136
Belgium	36,737(34,367-39,091)	57	41,696(39,150-44,308)	72	0.23(0.11,0.36)	0.60(0.55,0.65)	0.46(0.37,0.54)	0.44(0.38,0.49)	133
Cyprus	3,095(2,808-3,401)	150	4,533(4,176-4,914)	143	0.81(0.74,0.87)	1.29(1.26,1.33)	1.90(1.85,1.95)	1.32(1.29,1.36)	72
Denmark	21,008(19,468-22,788)	88	23,986(22,094-25,927)	94	1.10(1.01,1.19)	0.10(0.07,0.13)	0.28(0.08,0.47)	0.46(0.40,0.53)	130
Finland	18,696(17,460-19,925)	94	19,679(18,496-20,868)	102	0.17(0.11,0.24)	0.30(0.27,0.33)	0.08(0.03,0.14)	0.18(0.15,0.21)	147
France	272,473(252,533-295,269)	14	298,929(278,135-322,482)	14	0.12(-0.01,0.26)	0.70(0.68,0.73)	0.09(0.05,0.14)	0.33(0.29,0.38)	140
Germany	280,057(262,407-297,299)	13	291,026(272,995-309,179)	15	-0.27(-0.38,-0.17)	-0.46(-0.70,-0.22)	1.18(0.91,1.45)	0.14(0.01,0.27)	148
Greece	31,777(29,373-34,521)	64	30,651(28,354-32,938)	85	0.17(0.16,0.19)	0.32(0.29,0.35)	-0.94(-1.01,-0.87)	-0.12(-0.14,-0.09)	159
Iceland	1,151(1,057-1,257)	165	1,408(1,302-1,527)	170	0.84(0.72,0.96)	0.84(0.78,0.89)	0.40(0.33,0.47)	0.70(0.64,0.75)	106
Ireland	15,011(13,936-16,167)	103	18,831(17,589-20,092)	103	-0.09(-0.17,-0.01)	2.00(1.92,2.07)	0.32(0.24,0.39)	0.82(0.77,0.87)	95
Israel	22,975(21,220-24,905)	78	42,504(39,328-45,919)	71	2.43(2.41,2.46)	1.97(1.91,2.03)	1.98(1.88,2.09)	2.14(2.09,2.19)	43
Italy	218,741(208,284-229,412)	17	227,737(217,176-237,869)	19	-0.07(-0.12,-0.03)	0.83(0.79,0.88)	-0.42(-0.60,-0.25)	0.13(0.07,0.19)	151
Luxembourg	1,391(1,301-1,482)	162	2,175(2,040-2,309)	160	1.79(1.71,1.86)	0.96(0.79,1.14)	2.07(2.00,2.14)	1.57(1.49,1.66)	64
Malta	1,489(1,384-1,599)	160	1,556(1,462-1,651)	167	-0.08(-0.21,0.06)	-0.24(-0.33,-0.15)	0.73(0.70,0.77)	0.14(0.09,0.19)	149
Netherlands	55,232(51,572-58,879)	45	61,156(57,404-65,015)	57	0.73(0.59,0.87)	0.14(0.01,0.28)	0.14(0.07,0.20)	0.34(0.27,0.40)	138
Norway	17,720(16,834-18,673)	95	21,372(20,336-22,449)	100	0.92(0.81,1.03)	0.47(0.41,0.52)	0.55(0.42,0.67)	0.65(0.59,0.71)	110
Portugal	37,029(34,605-39,497)	56	35,835(33,789-37,903)	78	0.05(-0.06,0.15)	0.14(0.05,0.24)	-0.63(-0.70,-0.56)	-0.12(-0.17,-0.06)	160
Spain	130,818(120,316-141,792)	25	148,717(137,993-159,751)	28	-0.15(-0.22,-0.08)	1.99(1.92,2.06)	-0.59(-0.76,-0.42)	0.47(0.39,0.55)	128
Sweden	33,540(31,861-35,191)	60	39,247(37,386-41,088)	75	-0.12(-0.34,0.09)	0.84(0.80,0.88)	1.13(1.09,1.17)	0.56(0.48,0.64)	118
Switzerland	24,890(23,311-26,489)	75	31,138(29,254-33,028)	84	0.53(0.47,0.59)	0.49(0.36,0.63)	1.35(1.33,1.38)	0.78(0.74,0.83)	100
United Kingdom	246,061(234,216-258,973)	15	274,246(260,489-287,965)	17	-0.12(-0.15,-0.09)	0.48(0.42,0.55)	0.66(0.49,0.84)	0.35(0.29,0.42)	137
Argentina	136,814(126,113-148,371)	24	164,407(153,085-177,167)	25	0.57(0.54,0.60)	0.57(0.54,0.60)	0.72(0.55,0.88)	0.63(0.58,0.69)	113
Chile	55,193(50,814-60,062)	46	62,049(57,821-66,609)	55	0.26(0.09,0.43)	0.12(0.07,0.16)	0.82(0.63,1.02)	0.40(0.31,0.48)	135
Uruguay	12,036(11,165-12,993)	110	12,066(11,261-12,941)	120	0.43(0.40,0.46)	-0.50(-0.56,-0.45)	0.21(0.18,0.23)	0.01(-0.02,0.03)	155
Canada	60,890(57,865-63,809)	41	79,873(76,540-83,389)	46	0.84(0.76,0.92)	0.94(0.90,0.97)	1.06(1.01,1.12)	0.94(0.91,0.98)	86
United States of America	755,408(719,413-790,114)	4	888,977(852,130-925,837)	4	1.45(1.34,1.57)	-0.40(-0.47,-0.33)	0.60(0.54,0.67)	0.55(0.49,0.61)	120
Antigua and Barbuda	173(159-187)	193	195(181-209)	192	1.93(1.84,2.02)	-0.11(-0.19,-0.03)	-0.62(-0.63,-0.60)	0.42(0.38,0.45)	134
Bahamas	709(651-768)	175	845(785-904)	176	2.05(1.83,2.27)	0.50(0.40,0.61)	-0.60(-0.76,-0.44)	0.64(0.54,0.73)	112
Barbados	631(583-679)	178	629(589-671)	178	-0.45(-0.52,-0.38)	0.47(0.35,0.59)	-0.22(-0.28,-0.15)	-0.03(-0.09,0.03)	157
Belize	647(588-708)	177	1,097(1,010-1,186)	172	1.56(1.54,1.58)	2.66(2.62,2.70)	1.34(1.32,1.37)	1.85(1.82,1.87)	53
Cuba	26,961(24,843-29,034)	72	24,048(22,476-25,628)	93	-0.40(-0.49,-0.30)	-0.74(-1.39,-0.09)	-0.20(-0.52,0.12)	-0.42(-0.66,-0.19)	174
Dominica	217(199-235)	191	157(146-168)	193	-1.01(-1.04,-0.98)	-1.20(-1.21,-1.19)	-1.11(-1.14,-1.09)	-1.12(-1.13,-1.10)	192
Dominican Republic	23,465(21,324-25,576)	76	29,693(27,285-32,070)	87	1.36(1.31,1.41)	0.20(0.16,0.23)	0.94(0.91,0.97)	0.81(0.78,0.83)	96
Grenada	271(247-295)	186	241(224-259)	189	0.30(0.17,0.42)	-0.50(-0.70,-0.30)	-1.08(-1.10,-1.05)	-0.41(-0.48,-0.33)	172
Guyana	2,427(2,212-2,643)	153	2,040(1,879-2,201)	164	-0.17(-0.26,-0.09)	-1.62(-1.72,-1.53)	0.17(-0.04,0.38)	-0.57(-0.65,-0.49)	181

Haiti	22,311(20,250-24,413)	83	37,543(34,271-40,801)	76	1.89(1.78,2.00)	2.03(1.95,2.12)	1.57(1.52,1.61)	1.82(1.77,1.87)	56
Jamaica	7,149(6,535-7,771)	129	6,551(6,066-7,021)	136	0.33(0.25,0.41)	-0.68(-0.72,-0.64)	-0.52(-0.54,-0.50)	-0.29(-0.33,-0.26)	169
Saint Lucia	442(402-482)	181	376(350-403)	183	-0.07(-0.10,-0.05)	-0.81(-0.82,-0.80)	-0.77(-0.79,-0.76)	-0.56(-0.57,-0.55)	179
Saint Vincent and the Grenadines	341(311-371)	184	267(248-287)	187	-0.91(-0.92,-0.89)	-0.96(-1.00,-0.91)	-0.64(-0.65,-0.63)	-0.83(-0.85,-0.82)	190
Suriname	1,142(1,045-1,240)	166	1,451(1,341-1,561)	169	0.63(0.52,0.74)	1.32(1.27,1.37)	0.48(0.38,0.57)	0.85(0.80,0.91)	93
Trinidad and Tobago	3,548(3,240-3,852)	147	3,123(2,908-3,342)	155	-1.33(-1.37,-1.29)	0.00(-0.04,0.04)	-0.04(-0.08,0.01)	-0.44(-0.46,-0.41)	175
Bolivia (Plurinational State of)	21,407(19,918-22,998)	85	34,834(32,634-37,199)	81	2.06(2.00,2.12)	1.11(1.06,1.15)	2.03(1.90,2.15)	1.69(1.64,1.73)	62
Ecuador	32,844(30,486-35,345)	61	48,695(45,541-51,955)	68	1.52(1.47,1.57)	1.28(1.25,1.30)	1.33(1.29,1.38)	1.38(1.35,1.41)	70
Peru	63,015(58,522-67,776)	40	81,098(76,096-86,242)	45	0.47(0.44,0.50)	0.47(0.44,0.50)	1.87(1.61,2.13)	0.90(0.82,0.99)	89
Colombia	80,351(73,843-87,322)	35	92,590(86,178-99,095)	37	1.58(1.49,1.68)	0.13(0.07,0.20)	-0.10(-0.21,0.02)	0.50(0.44,0.55)	125
Costa Rica	7,673(7,054-8,349)	126	8,887(8,283-9,504)	128	1.26(1.12,1.41)	0.00(-0.12,0.13)	0.28(0.25,0.32)	0.52(0.45,0.58)	124
El Salvador	13,727(12,582-14,936)	108	13,148(12,197-14,148)	118	0.86(0.78,0.94)	-1.34(-1.40,-1.27)	0.04(0.00,0.08)	-0.15(-0.19,-0.11)	164
Guatemala	23,419(21,311-25,620)	77	41,149(37,973-44,549)	73	2.88(2.84,2.92)	1.70(1.63,1.76)	1.35(1.30,1.40)	1.96(1.93,2.00)	48
Honduras	13,812(12,593-15,124)	107	22,992(21,210-24,894)	97	2.04(2.02,2.06)	1.58(1.56,1.61)	1.70(1.69,1.71)	1.77(1.76,1.78)	60
Mexico	223,237(209,132-237,501)	16	254,711(240,728-267,820)	18	0.94(0.91,0.97)	0.31(0.29,0.33)	0.13(0.11,0.15)	0.46(0.44,0.48)	131
Nicaragua	11,439(10,443-12,530)	114	14,394(13,307-15,555)	115	1.04(0.99,1.08)	0.59(0.57,0.61)	0.75(0.70,0.80)	0.79(0.76,0.81)	97
Panama	5,731(5,274-6,216)	133	8,755(8,116-9,426)	129	1.51(1.44,1.58)	1.24(1.07,1.42)	1.77(1.65,1.89)	1.51(1.43,1.58)	66
Venezuela (Bolivarian Republic of)	47,542(43,655-51,703)	51	55,470(51,593-59,439)	60	1.30(0.89,1.71)	0.96(0.82,1.09)	-0.95(-1.27,-0.64)	0.46(0.27,0.64)	132
Brazil	516,640(487,448-548,724)	6	603,400(575,850-632,485)	6	0.53(0.49,0.57)	0.63(0.61,0.65)	0.44(0.39,0.48)	0.54(0.52,0.56)	123
Paraguay	16,152(14,724-17,647)	101	21,495(19,860-23,179)	99	1.40(1.28,1.52)	0.88(0.86,0.89)	0.72(0.67,0.77)	0.99(0.95,1.03)	85
Algeria	59,366(55,003-64,012)	42	82,659(76,892-88,643)	44	0.97(0.92,1.03)	1.15(1.13,1.17)	1.34(1.29,1.39)	1.15(1.12,1.18)	75
Bahrain	1,097(1,016-1,179)	167	2,277(2,108-2,453)	158	1.76(1.68,1.85)	5.55(5.40,5.70)	0.46(0.22,0.69)	2.52(2.41,2.62)	25
Egypt	93,660(85,503-102,135)	31	144,105(132,649-156,308)	29	0.98(0.93,1.03)	2.01(1.81,2.21)	1.48(1.40,1.55)	1.49(1.42,1.56)	67
Iran (Islamic Republic of)	144,877(135,875-154,077)	23	159,855(151,455-168,668)	26	-0.36(-0.48,-0.23)	0.63(0.59,0.67)	0.68(0.55,0.81)	0.34(0.28,0.41)	139
Iraq	43,866(39,969-48,054)	53	88,559(81,120-96,443)	40	4.01(3.51,4.53)	1.50(0.56,2.45)	2.06(1.90,2.21)	2.52(2.17,2.87)	26
Jordan	9,184(8,508-9,927)	120	23,720(22,083-25,507)	95	1.84(1.34,2.34)	4.10(3.16,5.05)	4.35(4.19,4.50)	3.38(3.04,3.73)	9
Kuwait	3,800(3,528-4,085)	144	7,827(7,233-8,416)	132	-0.08(-0.44,0.28)	3.85(3.70,4.01)	3.72(3.39,4.05)	2.55(2.37,2.73)	22
Lebanon	7,377(6,841-7,943)	127	9,948(9,249-10,671)	125	0.10(0.02,0.18)	0.63(0.47,0.79)	2.40(1.89,2.91)	1.00(0.83,1.17)	83
Libya	10,261(9,508-11,095)	117	11,877(11,022-12,753)	121	0.77(0.69,0.84)	0.95(0.91,0.98)	-0.23(-0.31,-0.15)	0.50(0.46,0.54)	126
Morocco	57,429(53,317-61,891)	43	67,474(62,802-72,470)	50	1.09(1.05,1.12)	0.70(0.68,0.72)	-0.09(-0.13,-0.06)	0.56(0.54,0.59)	119
Palestine	5,370(4,956-5,823)	135	10,963(10,175-11,811)	122	3.71(3.66,3.76)	2.66(2.61,2.71)	1.14(1.07,1.20)	2.49(2.46,2.53)	28
Oman	4,633(4,287-5,000)	138	8,698(8,013-9,382)	130	0.62(0.49,0.75)	1.31(0.79,1.84)	4.74(3.58,5.92)	2.17(1.75,2.59)	41
Qatar	908(838-979)	171	4,683(4,297-5,082)	141	1.99(1.84,2.14)	9.43(8.98,9.88)	5.95(5.72,6.18)	5.85(5.68,6.02)	1
Saudi Arabia	38,011(35,250-40,994)	55	63,185(58,368-68,010)	54	1.88(1.81,1.95)	1.71(1.68,1.74)	1.71(1.65,1.77)	1.76(1.73,1.80)	61
Syrian Arab Republic	32,246(29,806-34,871)	63	26,772(24,870-28,848)	91	2.09(1.95,2.23)	1.76(0.94,2.58)	-6.23(-6.97,-5.49)	-0.72(-1.17,-0.27)	184
Tunisia	18,845(17,486-20,315)	93	20,492(19,082-21,970)	101	0.54(0.50,0.57)	0.28(0.27,0.30)	0.07(0.04,0.10)	0.29(0.27,0.31)	144
Turkey	200,110(184,725-217,088)	19	207,399(192,007-224,118)	21	0.89(0.73,1.04)	-0.36(-0.50,-0.23)	-0.05(-0.16,0.06)	0.13(0.05,0.22)	152
United Arab Emirates	4,006(3,698-4,320)	142	13,726(12,484-15,040)	117	4.20(4.15,4.24)	9.65(9.37,9.93)	-0.23(-0.48,0.01)	4.36(4.23,4.49)	3

Yemen	36,342(33,504-39,513)	58	72,702(67,418-78,377)	47	2.60(2.56,2.64)	3.04(2.98,3.09)	1.61(1.52,1.70)	2.42(2.38,2.45)	30
Afghanistan	27,448(25,426-29,667)	70	95,041(87,931-102,778)	36	7.36(5.91,8.83)	3.22(3.12,3.33)	3.22(3.12,3.33)	4.43(3.96,4.91)	2
Bangladesh	425,466(386,391-472,996)	8	433,543(394,804-478,184)	11	0.72(0.64,0.79)	0.03(0.01,0.05)	-0.48(-0.50,-0.45)	0.07(0.04,0.09)	154
Bhutan	2,243(2,045-2,494)	156	1,971(1,799-2,168)	165	-0.42(-1.12,0.27)	-0.07(-0.18,0.05)	-0.90(-1.21,-0.59)	-0.44(-0.68,-0.19)	176
India	2,988,495(2,798,828-3,193,453)	2	3,739,095(3,518,140-3,973,898)	2	1.36(1.32,1.40)	0.96(0.93,0.99)	-0.02(-0.05,0.00)	0.78(0.76,0.80)	101
Nepal	74,007(67,365-82,068)	36	88,697(80,789-98,054)	39	1.63(1.59,1.68)	0.46(0.40,0.52)	-0.19(-0.21,-0.17)	0.62(0.60,0.65)	114
Pakistan	439,012(409,842-470,057)	7	782,596(732,900-836,309)	5	2.22(2.19,2.25)	2.38(2.36,2.40)	1.44(1.39,1.48)	2.02(2.00,2.04)	46
Angola	22,798(20,662-25,199)	79	65,851(59,692-72,837)	52	3.82(3.79,3.85)	4.19(4.15,4.23)	3.15(3.02,3.28)	3.74(3.70,3.79)	5
Central African Republic	5,961(5,408-6,590)	132	10,840(9,872-11,962)	123	2.72(2.64,2.79)	2.22(2.16,2.28)	1.23(1.03,1.44)	2.07(2.00,2.14)	45
Congo	5,267(4,793-5,813)	136	9,907(9,040-10,878)	126	2.01(1.97,2.04)	2.77(2.72,2.81)	1.76(1.63,1.90)	2.20(2.15,2.25)	40
Democratic Republic of the Congo	87,241(78,991-96,497)	33	180,701(164,303-199,307)	24	2.66(2.64,2.68)	2.80(2.78,2.83)	2.11(2.03,2.18)	2.54(2.51,2.56)	23
Equatorial Guinea	992(898-1,101)	169	2,686(2,449-2,956)	157	4.49(4.47,4.51)	3.98(3.92,4.03)	1.97(1.87,2.06)	3.50(3.46,3.54)	8
Gabon	2,066(1,880-2,277)	157	3,045(2,789-3,336)	156	1.99(1.96,2.03)	1.20(1.18,1.23)	0.84(0.82,0.87)	1.34(1.33,1.36)	71
Burundi	12,028(11,185-13,052)	111	24,576(22,846-26,644)	92	1.06(0.82,1.30)	3.58(3.25,3.91)	2.98(2.65,3.30)	2.53(2.36,2.70)	24
Comoros	959(892-1,039)	170	1,164(1,087-1,249)	171	1.17(1.13,1.21)	0.66(0.64,0.69)	0.17(0.14,0.19)	0.67(0.65,0.69)	108
Djibouti	1,007(937-1,090)	168	2,083(1,943-2,241)	162	1.87(0.88,2.86)	3.22(3.14,3.30)	2.45(2.15,2.74)	2.56(2.23,2.89)	21
Eritrea	6,351(5,907-6,886)	131	12,363(11,498-13,349)	119	3.39(3.24,3.55)	2.65(2.53,2.77)	1.18(0.97,1.38)	2.40(2.27,2.52)	31
Ethiopia	100,804(94,457-107,200)	28	197,185(185,000-210,531)	22	2.62(2.44,2.81)	2.08(2.02,2.13)	2.37(2.26,2.49)	2.37(2.29,2.44)	32
Kenya	43,383(40,788-46,095)	54	82,707(77,444-87,639)	43	2.39(2.37,2.40)	3.02(3.00,3.04)	1.39(1.35,1.43)	2.25(2.23,2.27)	36
Madagascar	24,965(23,226-27,058)	74	50,681(47,139-54,742)	64	2.86(2.83,2.88)	2.64(2.58,2.70)	1.96(1.88,2.03)	2.49(2.46,2.52)	29
Malawi	20,086(18,682-21,747)	90	35,210(32,733-38,148)	80	1.28(0.84,1.71)	2.66(2.60,2.71)	1.83(1.72,1.94)	1.97(1.83,2.11)	47
Mauritius	5,532(5,163-5,943)	134	6,355(5,938-6,826)	138	0.98(0.96,0.99)	0.40(0.35,0.44)	0.07(0.07,0.08)	0.47(0.46,0.49)	129
Mozambique	27,086(25,167-29,360)	71	61,648(57,246-66,835)	56	3.38(3.08,3.69)	2.82(2.79,2.85)	2.50(2.41,2.58)	2.89(2.80,2.99)	15
Rwanda	11,241(10,325-12,292)	115	16,648(15,394-18,041)	111	-0.28(-2.94,2.45)	1.75(1.57,1.93)	1.75(1.57,1.93)	1.11(0.26,1.97)	78
Seychelles	374(350-401)	183	499(467-535)	181	0.95(0.94,0.96)	1.06(1.06,1.07)	0.97(0.96,0.98)	1.00(0.99,1.00)	84
Somalia	15,030(13,936-16,318)	102	42,802(39,808-46,374)	70	3.98(3.77,4.18)	3.81(3.73,3.88)	3.41(3.30,3.51)	3.71(3.63,3.80)	6
United Republic of Tanzania	69,568(64,567-75,156)	38	142,350(132,767-152,611)	30	2.75(2.73,2.76)	2.49(2.37,2.61)	2.26(2.19,2.33)	2.50(2.46,2.55)	27
Uganda	47,800(43,754-52,387)	50	106,965(97,816-117,333)	34	3.80(3.72,3.88)	2.84(2.77,2.91)	1.77(1.69,1.86)	2.80(2.75,2.85)	17
Zambia	16,855(15,680-18,260)	99	35,549(33,056-38,442)	79	2.33(2.31,2.35)	2.88(2.84,2.92)	2.58(2.56,2.61)	2.61(2.59,2.63)	20
Botswana	2,685(2,439-2,961)	152	3,807(3,495-4,162)	149	1.59(1.55,1.64)	0.99(0.97,1.02)	1.06(1.00,1.12)	1.21(1.18,1.24)	74
Lesotho	3,637(3,312-4,003)	146	3,478(3,187-3,805)	150	0.20(0.11,0.29)	-0.56(-0.59,-0.54)	-0.04(-0.05,-0.02)	-0.15(-0.18,-0.12)	165
Namibia	2,892(2,635-3,191)	151	4,322(3,955-4,739)	147	2.31(2.26,2.37)	0.82(0.79,0.85)	1.05(1.02,1.07)	1.39(1.37,1.41)	69
South Africa	67,701(63,469-72,186)	39	86,399(81,393-91,661)	41	1.47(1.38,1.56)	0.54(0.45,0.64)	0.59(0.55,0.63)	0.86(0.80,0.92)	92
Eswatini	1,805(1,637-1,999)	158	2,067(1,889-2,268)	163	1.54(1.42,1.66)	0.01(-0.02,0.03)	-0.07(-0.08,-0.06)	0.48(0.43,0.52)	127
Zimbabwe	22,566(20,489-24,949)	80	29,304(26,713-32,207)	88	0.73(0.62,0.84)	0.83(0.81,0.85)	1.14(1.03,1.25)	0.90(0.85,0.96)	90
Benin	11,685(10,882-12,501)	113	28,140(26,321-30,059)	89	2.92(2.90,2.94)	3.28(3.26,3.30)	2.99(2.91,3.07)	3.07(3.04,3.10)	13
Burkina Faso	22,460(20,963-24,017)	81	50,609(47,328-54,069)	65	2.45(2.43,2.47)	3.05(3.04,3.06)	3.01(3.00,3.03)	2.84(2.83,2.85)	16
Cameroon	26,277(24,559-28,254)	73	64,746(60,812-68,894)	53	3.33(3.28,3.37)	3.98(3.94,4.01)	2.18(2.10,2.27)	3.17(3.14,3.21)	10

Cabo Verde	762(713-813)	174	894(843-949)	175	1.49(1.40,1.57)	0.15(0.13,0.17)	0.02(-0.03,0.07)	0.55(0.52,0.59)	121
Chad	14,341(13,372-15,364)	105	39,627(36,932-42,410)	74	3.41(3.39,3.43)	3.75(3.74,3.76)	3.56(3.55,3.56)	3.57(3.56,3.58)	7
C??te d'Ivoire	28,071(26,210-30,021)	69	53,464(50,138-57,048)	61	2.88(2.83,2.93)	2.21(2.18,2.25)	1.64(1.60,1.68)	2.25(2.22,2.27)	37
Gambia	2,305(2,152-2,467)	154	4,537(4,260-4,844)	142	2.84(2.74,2.93)	2.55(2.53,2.57)	1.66(1.54,1.78)	2.36(2.31,2.41)	33
Ghana	28,164(26,367-30,158)	68	49,431(46,548-52,620)	66	0.96(0.59,1.34)	2.35(2.31,2.39)	2.35(2.31,2.39)	1.92(1.80,2.04)	50
Guinea	14,242(13,297-15,237)	106	27,654(25,891-29,529)	90	2.56(2.18,2.95)	2.08(1.92,2.24)	2.49(2.44,2.54)	2.32(2.17,2.48)	34
Guinea-Bissau	2,298(2,148-2,456)	155	3,906(3,665-4,170)	148	1.92(1.87,1.97)	1.98(1.97,2.00)	1.63(1.60,1.67)	1.85(1.83,1.87)	54
Liberia	4,247(3,980-4,536)	140	9,178(8,625-9,779)	127	3.84(2.23,5.47)	3.16(2.78,3.55)	0.84(0.28,1.41)	2.65(2.10,3.20)	18
Mali	21,208(19,591-23,087)	87	52,301(48,395-56,682)	63	2.38(2.25,2.51)	3.61(3.55,3.67)	3.39(3.35,3.43)	3.15(3.10,3.20)	11
Mauritania	4,649(4,345-4,969)	137	7,790(7,318-8,304)	133	2.04(2.01,2.07)	2.30(2.26,2.35)	1.04(0.97,1.12)	1.79(1.76,1.83)	58
Niger	19,346(18,029-20,725)	92	57,108(53,182-61,151)	58	3.57(3.47,3.68)	4.23(4.21,4.24)	3.57(3.54,3.59)	3.80(3.76,3.83)	4
Nigeria	197,113(184,703-210,390)	20	452,274(424,739-482,293)	10	3.18(3.16,3.19)	3.33(3.30,3.37)	2.24(2.19,2.29)	2.92(2.90,2.94)	14
Sao Tome and Principe	268(251-287)	187	368(346-392)	184	1.31(1.26,1.36)	1.69(1.62,1.76)	0.27(0.16,0.39)	1.09(1.03,1.14)	79
Senegal	17,676(16,511-18,897)	98	30,015(28,189-32,011)	86	1.81(1.78,1.85)	2.24(2.18,2.29)	1.37(1.24,1.51)	1.83(1.78,1.87)	55
Sierra Leone	8,007(7,487-8,552)	125	16,689(15,670-17,809)	110	1.42(0.92,1.92)	3.66(3.46,3.86)	2.49(2.41,2.56)	2.64(2.45,2.82)	19
Togo	8,531(7,967-9,120)	122	15,645(14,685-16,694)	113	2.24(2.21,2.27)	2.48(2.44,2.52)	1.52(1.40,1.64)	2.10(2.06,2.15)	44
American Samoa	244(226-261)	188	268(249-287)	186	1.84(1.72,1.96)	-0.45(-0.49,-0.41)	-0.41(-0.51,-0.31)	0.31(0.25,0.36)	142
Bermuda	139(129-149)	194	129(121-137)	196	0.27(0.23,0.32)	-0.08(-0.11,-0.06)	-1.11(-1.13,-1.08)	-0.29(-0.31,-0.27)	170
Cook Islands	93(87-100)	198	88(82-94)	199	-0.32(-0.42,-0.21)	0.02(-0.26,0.29)	-0.43(-0.61,-0.25)	-0.28(-0.39,-0.16)	168
Greenland	130(122-137)	195	125(119-131)	197	0.09(-0.11,0.29)	-0.41(-0.49,-0.33)	0.01(-0.19,0.22)	-0.13(-0.23,-0.03)	162
Guam	655(607-701)	176	823(768-879)	177	1.79(1.75,1.83)	0.13(0.10,0.15)	0.47(0.45,0.48)	0.79(0.77,0.80)	98
Monaco	101(95-107)	197	131(124-140)	195	1.23(1.15,1.31)	0.68(0.63,0.72)	0.87(0.83,0.90)	0.93(0.90,0.96)	88
Nauru	52(48-56)	201	52(48-56)	202	0.22(0.18,0.25)	-0.25(-0.27,-0.23)	-0.04(-0.07,-0.01)	-0.03(-0.05,-0.01)	158
Niue	11(11-12)	203	8(7-9)	203	-1.83(-1.87,-1.79)	-2.09(-2.17,-2.00)	0.32(0.29,0.35)	-1.23(-1.26,-1.20)	196
Northern Mariana Islands	218(201-235)	190	199(185-215)	191	4.96(4.23,5.69)	-3.96(-4.17,-3.75)	-2.73(-3.10,-2.36)	-0.39(-0.69,-0.09)	171
Palau	74(69-79)	200	82(76-88)	200	2.25(2.16,2.35)	-0.48(-0.56,-0.41)	-0.65(-0.73,-0.56)	0.32(0.26,0.37)	141
Puerto Rico	9,463(8,739-10,200)	119	7,033(6,593-7,486)	134	0.14(0.02,0.26)	-1.12(-1.19,-1.04)	-2.17(-2.29,-2.06)	-1.04(-1.11,-0.97)	191
Saint Kitts and Nevis	123(112-133)	196	133(124-143)	194	0.15(0.11,0.20)	0.59(0.57,0.61)	0.12(0.07,0.17)	0.30(0.27,0.32)	143
San Marino	87(81-93)	199	118(110-125)	198	1.81(1.79,1.83)	1.06(1.00,1.11)	0.24(0.22,0.26)	1.05(1.03,1.07)	82
Tokelau	9(8-9)	204	7(7-7)	204	-0.84(-1.07,-0.61)	-3.13(-3.29,-2.98)	1.32(1.25,1.40)	-0.80(-0.89,-0.70)	188
Tuvalu	48(45-51)	202	56(52-60)	201	0.00(-0.02,0.01)	0.74(0.71,0.77)	0.90(0.90,0.91)	0.55(0.54,0.56)	122
United States Virgin Islands	295(270-319)	185	238(221-254)	190	-0.21(-0.23,-0.20)	-0.97(-0.98,-0.97)	-1.01(-1.02,-1.00)	-0.74(-0.74,-0.73)	185
South Sudan	11,788(10,966-12,754)	112	18,513(17,194-20,032)	105	2.57(2.53,2.61)	2.57(2.53,2.61)	-0.57(-1.20,0.06)	1.58(1.38,1.78)	63
Sudan	49,558(45,899-53,520)	49	92,193(85,488-99,360)	38	3.17(2.90,3.43)	1.97(1.88,2.07)	1.48(1.39,1.56)	2.17(2.08,2.26)	42
